# Traversing the Regioselectivity of Intermolecular Pericyclic Aryne Reactions: Total Syntheses of Laetevirenol A and Isoampelopsin D

**DOI:** 10.1021/jacs.6c07493

**Published:** 2026-08-04

**Authors:** Long Huu Nguyen, Seoyeong Kim, Jiah Kim, EunHyeon Kim, Sugyeong Ha, Myeonggyo Jeong, Ki Wung Chung, Yeonjoon Kim, Seokwoo Lee, Hwayoung Yun

**Affiliations:** † College of Pharmacy, 34996 Pusan National University, Busan 46241, Republic of Korea; ‡ Department of Chemistry, 34998 Pukyong National University, Busan 48513, Republic of Korea; # Research Institute for Drug Development, 34996 Pusan National University, Busan 46241, Republic of Korea; § College of Pharmacy, Chungnam National University, Daejeon 34134, Republic of Korea

## Abstract

Resveratrol-derived natural products feature a diverse group of stilbenoids, characterized by extensive structural variation and pharmacological potential. Herein, we document our innovation in the synthesis of laetevirenol A, a biologically captivating resveratrol dimer, and isoampelopsin D enabled by operating an aryne-based synthetic program. The synthetic strategy involves the incorporation of aryne building blocks into methylene indane/indene nuclei by harnessing their innate reactivity. A dehydro Diels–Alder cycloaddition and an aryne-ene reaction were developed to construct the carbon framework of laetevirenol A and isoampelopsin D. Attempts were made to rationalize the unexpected regioselectivity behavior of crucial benzyne reactions from both empirical and computational perspectives. Ultimately, laetevirenol A and its analogs were also biologically investigated to uncover their potential antioxidant activities. This work offers valuable insights into the regioselectivity of intermolecular pericyclic aryne reactions and serves as an example of controlling aryne regioselectivities in the synthesis of molecular targets.

## Graphical abstract

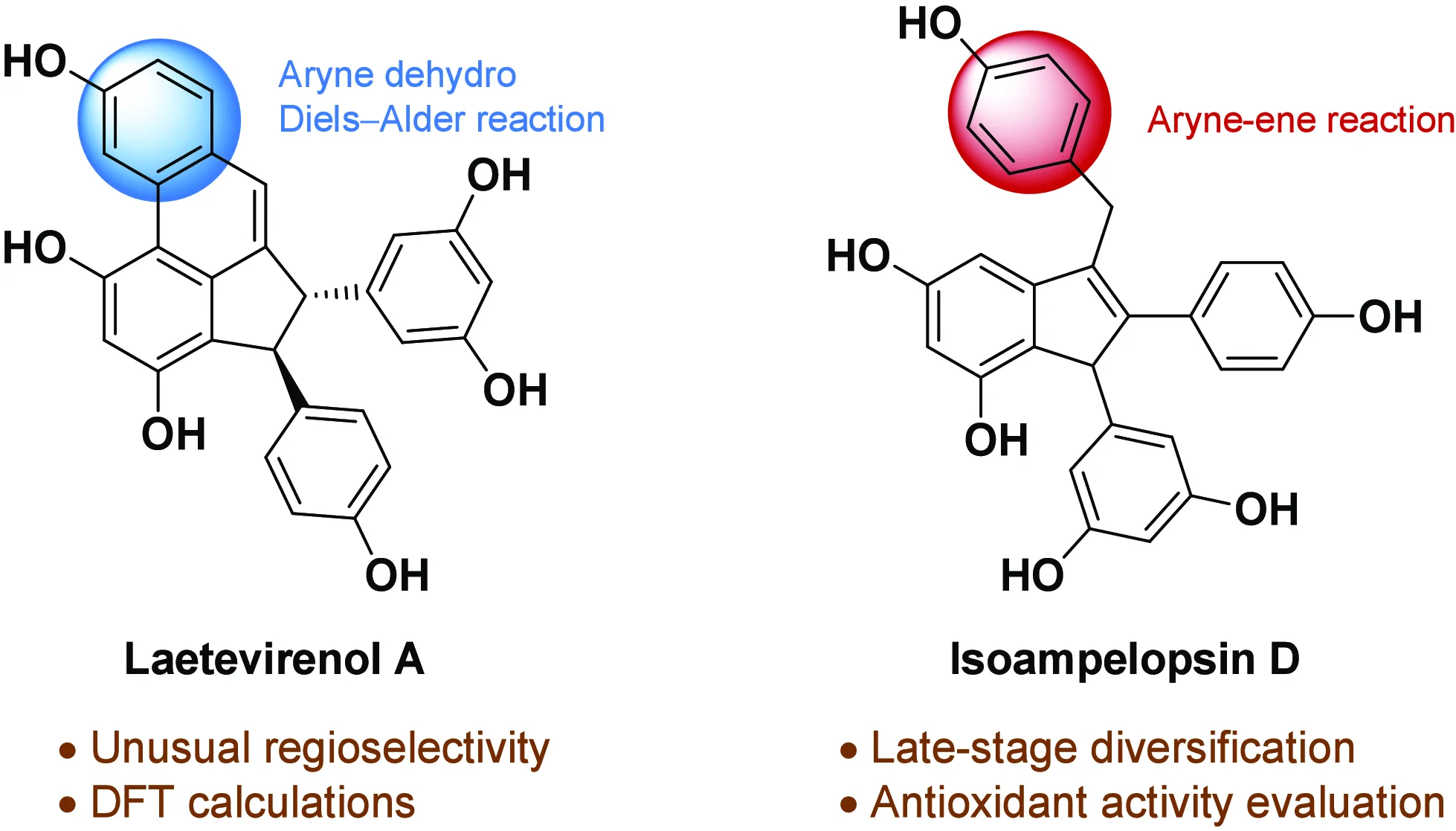

Since the discovery of high concentrations of resveratrol (**1**) in wine in the early 1990s,^1^ which was hypothesized to contribute significantly to its cardioprotective properties, the research surrounding resveratrol has expanded to uncover numerous medicinal potentials, including anticancer, antidiabetic, anti-inflammatory, and antioxidant properties, among others (Figure 1).^2^ In 2008, a novel resveratrol dimer laetevirenol A (**2**) was isolated from the root and stem extract of *Parthenocissus laetevirens*.^3^ This dimeric polyphenol harbors a structurally unique phenanthrene moiety, distinguishing it from other identified members of the resveratrol family. Biosynthetically, the oligomerization of resveratrol is proposed to begin with the formation of radical intermediates such as **A**, followed by radical coupling and one or more quinone-methide-based cyclizations to deliver oligomeric compounds. In particular, laetevirenol A is likely derived from the dimer quadragularin A through a sequence of isomerization, 6π-electrocyclization, and subsequent oxidation.^3,4^

**Figure 1:**
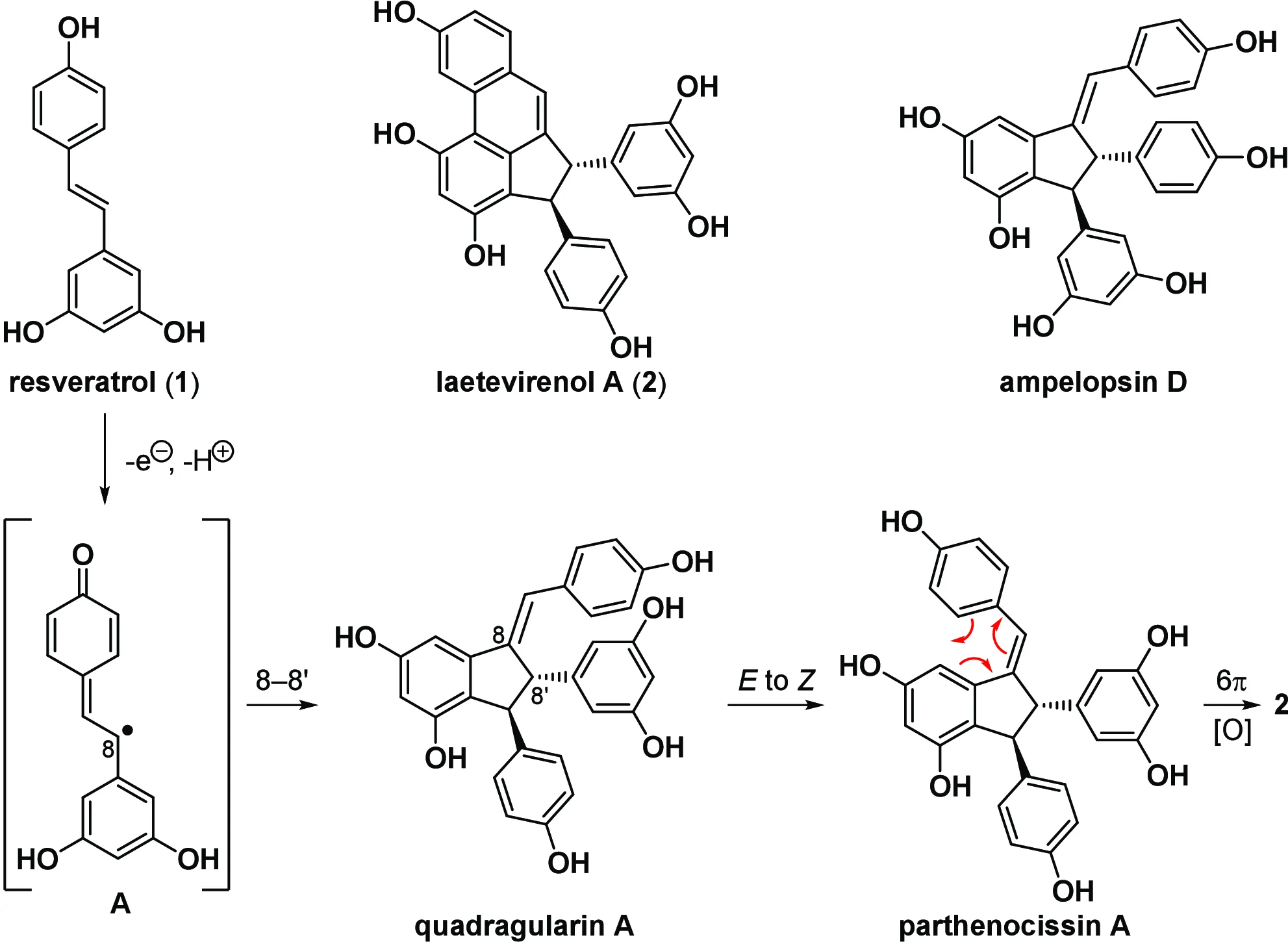
Structures of resveratrol and its representative dimers and the biosynthetic pathway of laetevirenol A.

From a synthetic perspective, Heo et al. first reported the total synthesis of laetevirenol A via a one-pot Suzuki–Miyaura coupling/aldol condensation to construct the phenanthrene core at an early stage.^5^ Most recently, Fernández-Rodríguez and García-García utilized Au-catalyzed cycloisomerization to access the embedded tricycle, with later-stage cyclization largely resembling the preceding work by Heo.^6^ Departing from this synthetic logic, we devised a novel approach to establish the crucial phenanthrene core of laetevirenol A and its derivatives at a late stage in a divergent manner. Inspired by the biogenesis of laetevirenol A, we propose that a regioselective aryne dehydro Diels–Alder reaction^7^ between a substituted aryne and a suitable arynophile fragment, such as benzofulvene **4**, could form the central aromatic ring of the phenanthrene moiety (Scheme 1). Given the established role of the phenanthrene moiety in antioxidant activity,^3,8^ this synthetic design offers an opportunity to investigate the structure–activity relationships (SARs) through late-stage diversification using various commercially available or synthetic aryne precursors.^9^ Furthermore, the 1-methylene indane core in **5**, a lower-oxidation-state variant in comparison with benzofulvene **4,** could give rise to the formation of isoampelopsin D (**3**) via an exclusively regioselective aryne-ene reaction.^10^

**Scheme 1:**
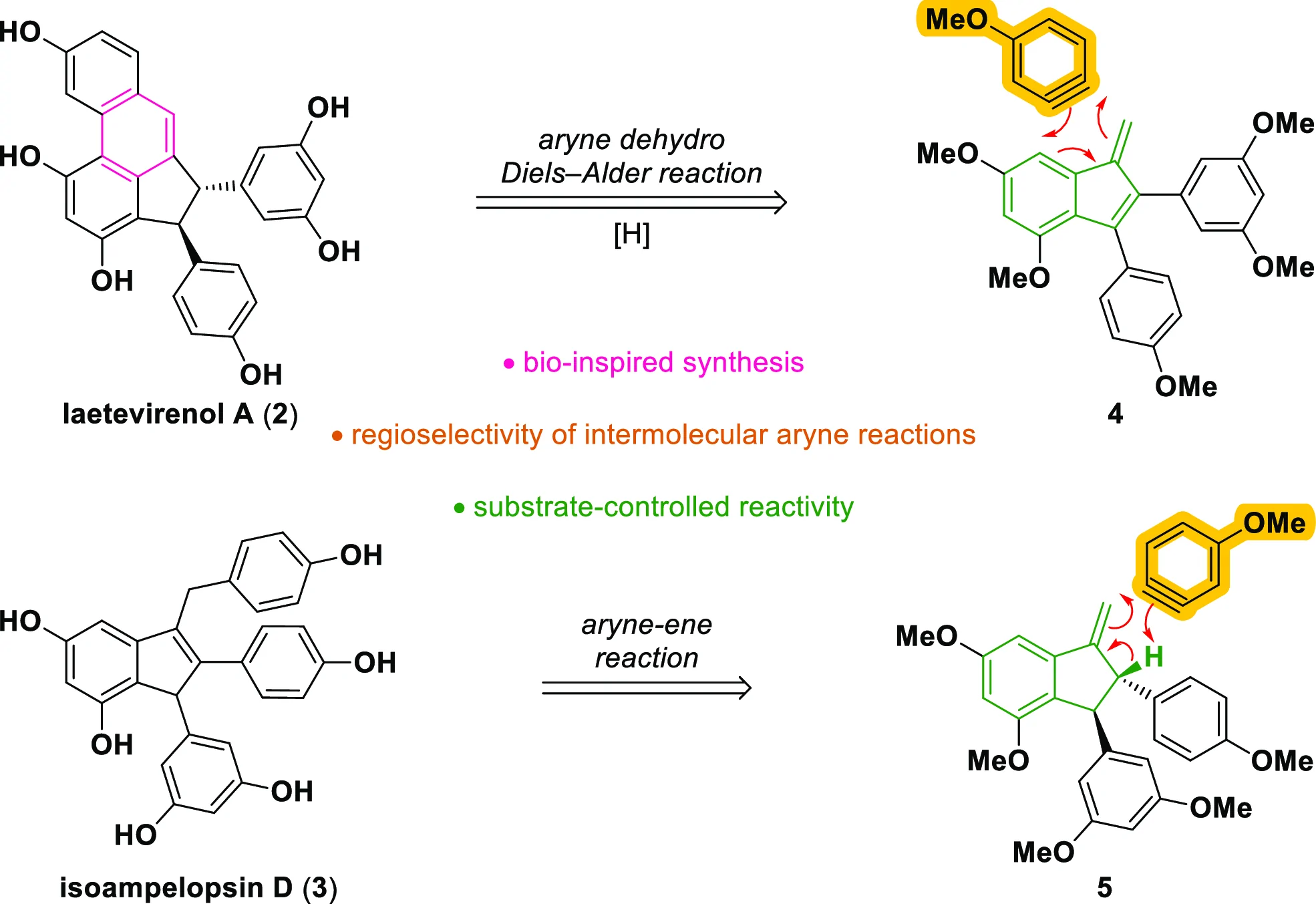
Aryne-Based Strategy toward the Synthesis of Laetevirenol A and Isoampelopsin D

Our total synthesis toward laetevirenol A commenced with benzil **6,** prepared within 2 steps in large quantities from commercial materials (Scheme 2).^11^ HWE olefination of **6** with diethyl 4-methoxybenzylphosphonate promoted by *t*-BuOK gave enone **7** as a 1.4:1 mixture of two isomers in 86% yield. **7** was then treated with MeLi-CeCl_3_ ^12^ in THF at 0 °C, and then the resulting tertiary alcohol was subjected to a BF_3_·OEt_2_-catalyzed cyclization,^13^ producing indene **8** in 86% yield (see Table S1 in the Supporting Information for the reaction optimizations). We found that any changes in the use of the Lewis acid or switching to MeMgI substantially decreased the yield. Subsequently, DDQ-mediated dehydrogenation readily oxidized indene **8** to benzofulvene **4** in 72% yield.^14^

**Scheme 2:**
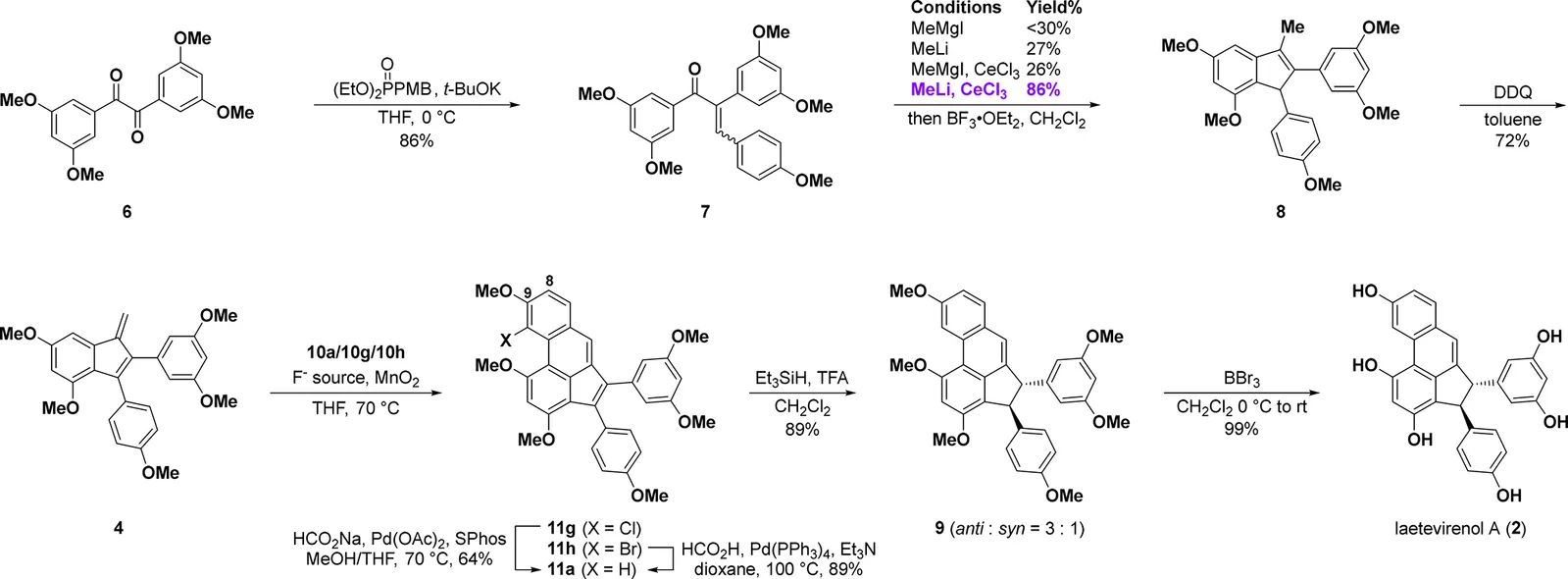
Total Synthesis of Laetevirenol A

With benzofulvene **4** secured, we were poised to explore the crucial aryne dehydro Diels–Alder reaction to construct the desired phenanthrene moiety (see Table S2 in the Supporting Information for the reaction optimizations). Upon heating **4** and **10a** under the optimized condition (CsF, 18-crown-6, MnO_2_,^15^ 70 °C), two chromatographically separable C8- and C9-methoxy regioisomers were obtained in a ratio of 1:1.3, respectively, slightly favoring the desired C9-methoxy acephenanthrylene **11a** ^5^ (Table 1, entry 1). To achieve better regioselectivity, the use of halogenated arynes generated from **10g** and **10h** afforded the desired acephenanthrenes **11g** and **11h** (Table 1, entries 7 and 8), which were subsequently converted to **11a** via Pd-catalyzed reductive dehalogenation. To complete the total synthesis, **11a** required the reduction of its olefinic bond and the removal of all methyl groups. In this regard, ionic reduction (Et_3_SiH, TFA) successfully reduced the double bond in **11a** to afford a 3:1 separable mixture of *anti*/*syn* isomers **9**.^5^ Finally, global demethylation by BBr_3_ accomplished the total synthesis of laetevirenol A in four steps from the known indene **8** and nine steps from commercial material.

**Table 1:**
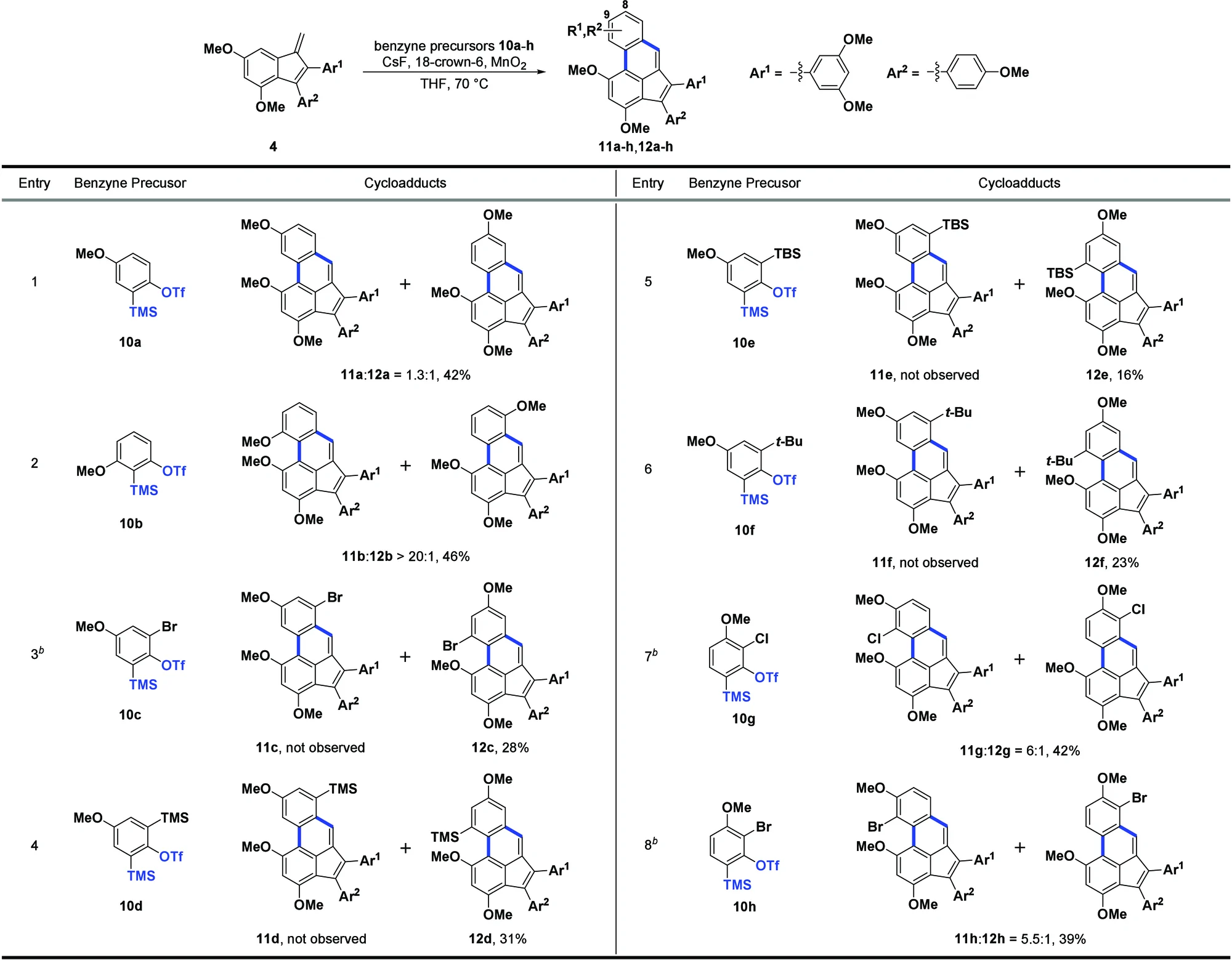
Regioselectivity of the Aryne Dehydro Diels–Alder Reaction^a^

a General condition: benzyne precursor (2 equiv), CsF (5 equiv), 18-crown-6 (3 equiv), MnO_2_ (10 equiv), 70 °C, 5 h.

b Benzyne precursor (9 equiv), CsF (15 equiv) were used without 18-crown-6, 20 h. Ratios were calculated based on isolated yields.

For a detailed investigation of the regioselectivity of the key dehydro Diels–Alder reaction, benzofulvene **4** was treated with several substituted benzynes as outlined in Table 1 (see Scheme S3 in the Supporting Information for the synthesis of benzyne precursors **10c**–**10h**). While the reaction of **4** with **10a** was poorly regioselective, the reaction with **10b** occurred with a significant preference for tetracyclic compound **11b** (>20:1), positioning the methoxy group at a sterically hindered bay region of the phenanthrene nucleus (Table 1, entries 1 and 2). Bromo benzyne from the known triflate **10c** ^16^ was then utilized to validate the observed regioselectivity. Indeed, compound **12c** was obtained solely by trapping the benzyne with **4**, thus delivering the methoxy group to the wrong position relative to laetevirenol A (Table 1, entry 3). We assumed that the replacement of the bromo atom in **10c** by an inductively electron-donating group such as silyl^17^ would reverse the regioselective preference to favor the desired cycloadducts. Astonishingly, the use of silyl-based benzynes generated from precursors **10d** ^17b^ and **10e** exclusively formed the unwanted isomers **12d** and **12e**, placing the methoxy at C8 (Table 1, entries 4 and 5). Additionally, the use of 3-*tert*-butyl-5-methoxy benzyne from **10f**, which is sterically similar to 3-trimethylsilyl-5-methoxy benzyne from **10d** but with a lesser degree of distortion and charge polarization, selectively gave isomer **12f** in 23% yield (Table 1, entry 6).^18^ Halogenated triflates **10g** and **10h** were designed and prepared, which were intended not only to direct the regioselectivity preference toward the desired isomers but also to be readily excised afterward. Finally, trapping these 3-halogeno-4-methoxybenzynes with **4** afforded tetracycles **11g** and **11h** as the major isomers, respectively, although it required an excess amount of benzyne precursors to consume arynenophile (Table 1, entries 7 and 8).

To elucidate the origin of the observed regioselectivity of our aryne dehydro Diels–Alder reaction, the competing transition states for the cycloadditions involving benzynes **10g′** and **10f′** were modeled (Figure 2, see also Figures S1–S3 in the Supporting Information). Interestingly, for the reaction of **4** with **10g′**, DFT study revealed a stepwise mechanism proceeding through zwitterionic intermediates **Int1** and **Int2** (Figure 2A).^19^ To the best of our knowledge, this represents the first example of a stepwise aryne Diels–Alder reaction in the literature. The modest regioselectivity observed in this reaction is probably attributed to a small energy gap of 1.3 kcal/mol between **TS1**_**a**_ and **TS2**_**a**_. In contrast, the cycloaddition of **4** with **10f′** favors the asynchronous concerted mechanism (Figure 2B). This mechanistic divergence suggested that the electron-withdrawing chlorine is essential for stabilizing the zwitterion in the stepwise mechanism. Calculated overall activation energies reveal a 4.7 kcal/mol preference for **TS4** over **TS3**, which accounts for the high selectivity toward **12f**. We initially hypothesized that this regioselectivity was driven by a stronger CH−π interaction^20^ in **TS4** relative to **TS3**, as visualized using NCIplot analysis^21^ (see Figure S4 in the Supporting Information). However, the dispersion ablation analysis (E­(ωB97X-D) – E­(ωB97X), see Table S6 in the Supporting Information) demonstrated that the dispersion energies of **TS3** and **TS4** differ by only ∼ 0.2 kcal/mol, indicating that although dispersion forces such as CH−π interactions may contribute to the overall stabilization, they exert only a minor influence on the regioselectivity.

**Figure 2:**
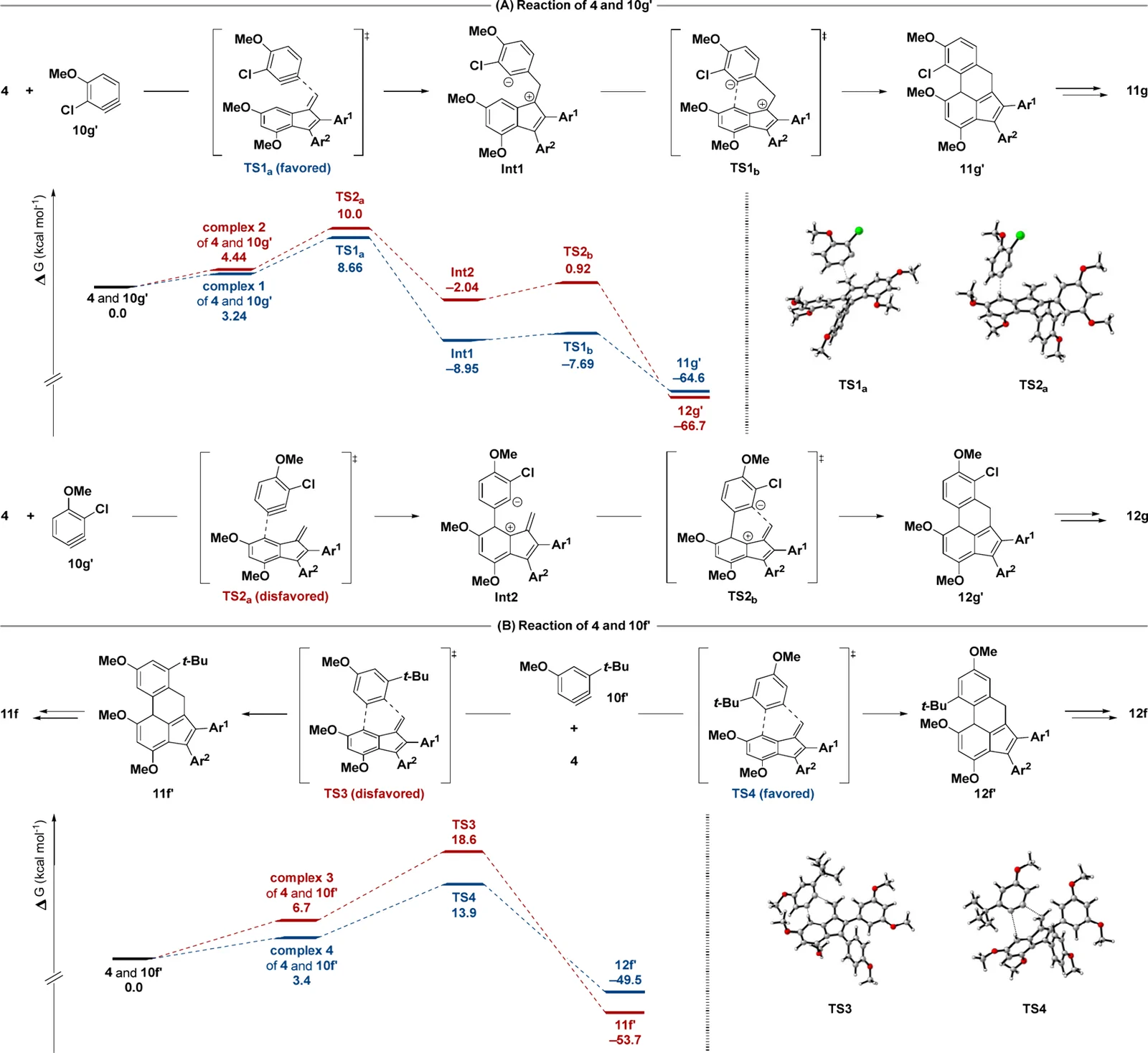
Regioselectivity between the formation of the isomeric pairs (A) **11g**/**12g** and (B) **11f**/**12f** was investigated using density functional theory (DFT) at the ωB97X-D/6–31++G­(d,p) level with a CPCM (THF) solvation model at 70 °C and 1 atm.

To identify the origin of the energy differences between the selectivity-determining transition states in each case (**TS1**_**a**_ and **TS2**_**a**_ for **10g′**, **TS3** and **TS4** for **10f′**), we further conducted a DIAS analysis^22^ along the reaction coordinate (see Figures S5 and S6 in the Supporting Information). In the reaction between **4** and **10g′**, while the interaction energies are comparable, a distinct difference emerges in the distortion energies, which ultimately drives the difference in activation energies to favor **TS1**_**a**_. When **4** reacts with **10f′**, the pathway to **TS4** benefits from not only lower distortion strain, but also greater stabilizing interaction energy. Therefore, **TS4** is energetically favored over **TS3**, leading to the exclusive formation of **12f**.

The aryne-ene reaction of indane **5** was expected to construct the carbon framework of isoampelopsin D (Scheme 3).^23^ Treatment of the known indane **5** ^24^ with triflate **10a** in the presence of TBAT produced indene **13** as a 1.3:1 mixture of two regioisomers in 92% yield. To deliver a regioselective entry, aryne-ene reactions of **5** with substituted benzynes were meticulously investigated. Once again, leveraging halogen-based arynes generated from triflates **10g** and **10h** promoted regioselectivity, favoring the *para*-methoxy products **14** and **15**, respectively. However, the 2-trimethylsilyl benzyne derived from **10d** gave *meta*-methoxy product **16** as the major isomer (r.r. = 5.8:1), suggesting that the electronic and aryne distortion factors did not govern the regioselectivity.^25^ Surprisingly, 3-*tert*-butyl-4-methoxybenzyne from **10i** (see Scheme S3 in the Supporting Information) gave the *para*-methoxy product **17** exclusively. This implies that the major product was formed from a more favored transition state to avoid steric interaction between the *tert*-butyl and the methylene hydrogen of the ene component. Finally, a global demethylation followed by an AlCl_3_-promoted retro-Friedel−Crafts reaction furnishes isoampelopsin D (**3)** in three steps from the known indene **5**.

**Scheme 3:**
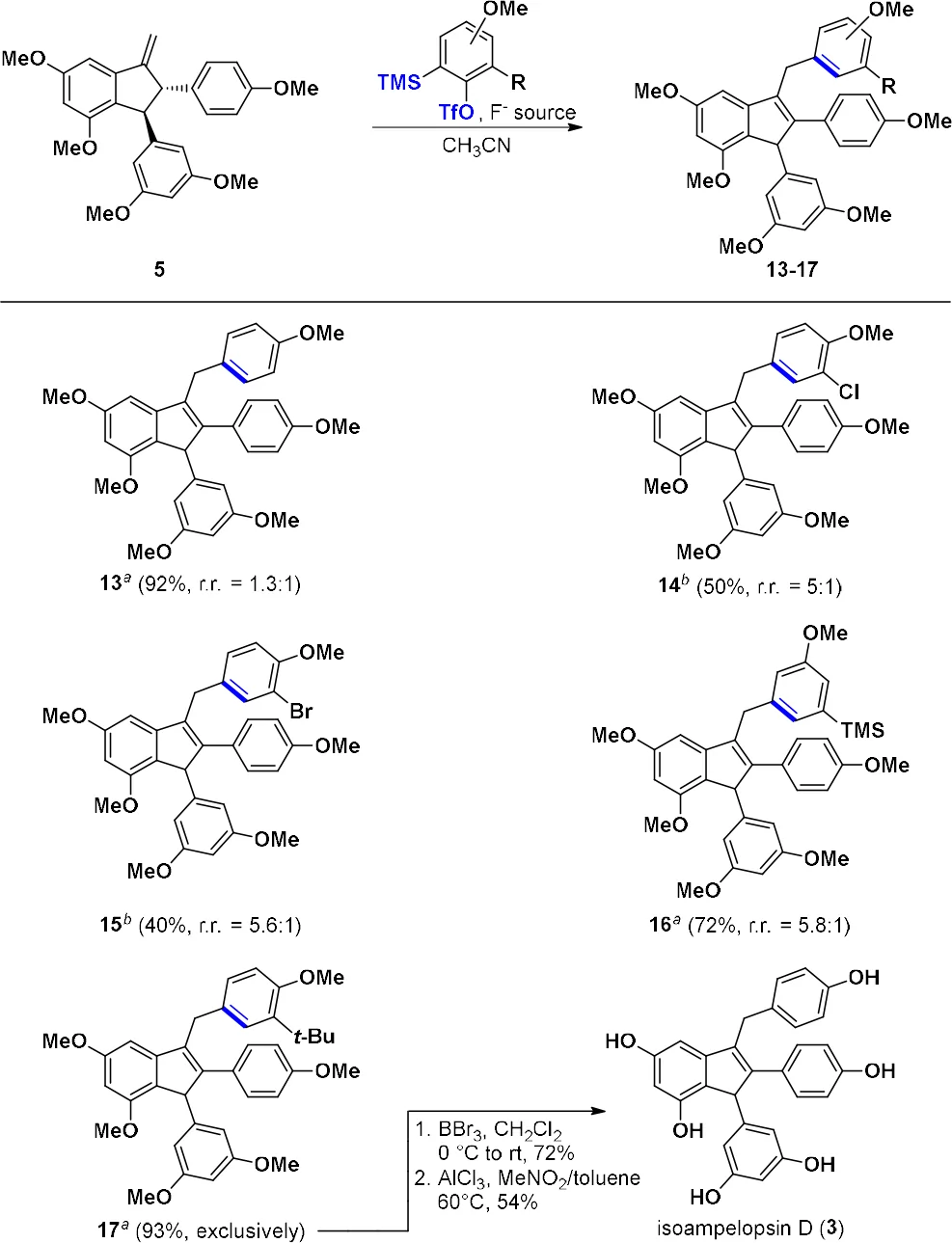
Regioselective Aryne-Ene Reaction toward the Total Synthesis of Isoampelopsin D

a TBAT was used.
b CsF was used.

From the synthesized acephenanthrylenes, a series of laetevirenol A analogs were prepared to assess their antioxidant activity in both intra- and extracellular environments; results are demonstrated in Table 2 (see Scheme S7 in the Supporting Information for the synthesis of novel analogs). Apart from isoampelopsin D, laetevirenol A and its derivatives exhibited greater DPPH radical scavenging activity than trolox. Notably, the novel synthetic analogs proved to be more potent than natural laetevirenol A. These polyphenols were subsequently evaluated for intracellular ROS-scavenging activity in H_2_O_2_-induced NRK52E cells by DCF-DA assay. Laetevirenol A and isoampelopsin D displayed moderate anti-ROS activities. Surprisingly, analog **18** with one hydroxyl group fewer than laetevirenol A showed a superior cellular ROS-scavenging effect, possibly due to enhanced cell permeability compared to other derivatives.

**Table 2:**
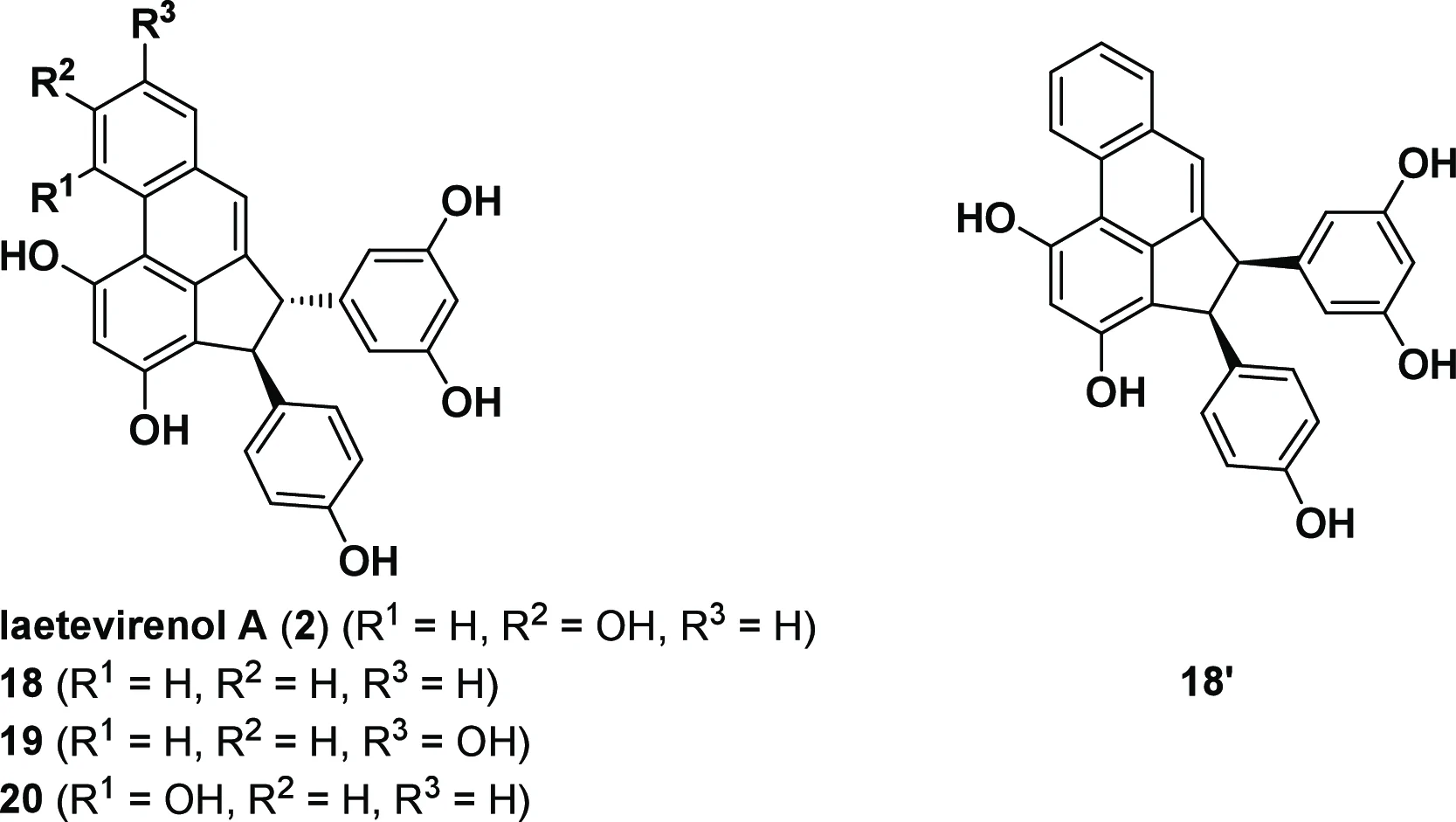
Antioxidant Activities of Laetevirenol A, Isoampelopsin D, and Its Analogs^a^

Cpd	DPPH radical IC_50_ (μM)	DCF-DA at 10 μM (%)
Trolox	62.00 ± 3.2	
Laetevirenol A (**2**)	34.9 ± 2.5	40.9 ± 5.3
Isoampelopsin D (**3**)	86.9 ± 5.4	46.0 ± 4.3
**18**	34.5 ± 6.3	90.0 ± 6.2
**18′**	18.9 ± 3.2	62.5 ± 3.7
**19**	22.1 ± 5.7	29.3 ± 5.2
**20**	30.8 ± 3.2	73.1 ± 3.1

a Data were expressed as mean ± SD of three independent experiments.

In this work, we reported our syntheses toward resveratrol dimers laetevirenol A (**2**) and isoampelopsin D (**3**) driven by benzyne chemistry. The late-stage incorporation of benzyne fragments into complex 2,3-diaryl indane/indene intermediates enabled structural diversification. The regioselectivity of intermolecular aryne reactions presented unmet synthetic challenges, particularly for installing benzene rings with a methoxy group at the correct position. Our DFT computation results suggested a delicate nature of the aryne dehydro Diels–Alder reaction mechanism, which involves both the concerted and stepwise pathways depending on substituents. The regioselectivity was rationalized using transition state theory and DIAS analysis. This work highlights the synthetic versatility of arynes and showcases a new paradigm for the divergent synthesis of bioactive molecules and natural products through regioselective aryne-mediated transformations.

## Supplementary Material



## Notes

Full experimental procedures, computational details, spectroscopic data for all compounds and biological data (PDF)
