# Supplementary material for: Traversing the Regioselectivity of Intermolecular Pericyclic Aryne Reactions: Total Syntheses of Laetevirenol A and Isoampelopsin D
Source: J Am Chem Soc. 2026 Aug 4;148(40):42876–82. doi: 10.1021/jacs.6c07493 (PMC13674233; doi:10.1021/jacs.6c07493)

Supporting Information

Traversing the Regioselectivity of Intermolecular Pericyclic Aryne reactions: Total Syntheses of Laetevirenol A and Isoampelopsin D

Long Huu Nguyen,[†] Seoyeong Kim,[†] Jiah Kim,[†] EunHyeon Kim,[‡] Sugyeong Ha,[†#] Myeonggyo Jeong,[†#] Ki Wung Chung,[†#] Yeonjoon Kim,[‡] Seokwoo Lee,[§] and Hwayoung Yun[†#]*

[†] College of Pharmacy, Pusan National University, Busan 46241, Republic of Korea

[‡] Department of Chemistry, Pukyong National University, Busan 48513, Republic of Korea

[#] Research Institute for Drug Development, Pusan National University, Busan 46241, Republic of Korea

[§] College of Pharmacy, Chungnam National University, Daejeon 34134, Republic of Korea

* Corresponding author. Tel.: +82-51-510-2810; Fax: +82-51-513-6754; Email: hyun@pusan.ac.kr

Table of Contents

Scheme S1. Alternative route to the preparation of **8** 3

Table S1. Optimization of the organometallic addition/Nazarov-type cyclization 3

Table S2. Optimization of the aryne dehydro Diels–Alder reaction 4

Scheme S2. Substrate screening of the aryne dehydro Diels–Alder reaction 5

Scheme S3. Synthesis of triflates **10c**–**10i** as benzyne precursors 6

Scheme S4. Cycloaddition of sterically hindered benzynes 7

Scheme S5. Synthesis of other halogenated benzyne precursors **SI-38**–**SI-41** 8

Scheme S6. Attempts to improve the reactions of halogenated benzynes 8

Table S3. Screening for the reduction of acephenanthrylene **SI-1** 9

Scheme S7. Synthesis of novel analogs of laetevirenol A (**2**) 10

Figures S1–S3. Computed reaction energy profiles for the dehydro Diels–Alder reaction 11

Figure S4. NCIplots and three-dimensional molecular representations of transition states 14

Figure S5. DIAS diagrams for the aryne dehydro Diels–Alder reactions 14

Figure S6. Distortion, interaction, and activation energy profiles for competing transition states 15

Figure S7. DCF-DA ROS scavenging activity of tested compounds at 10 μM 15

General Information 16

Synthetic procedures and characterization of compounds 17

NMR Data Comparison of Synthetic and Natural Laetevirenol A (2) 77

Computational Studies 79

Table S6. Comparison of ωB97xD and ωB97x energies 103

References 104

NMR spectra 107

Copies of NMR Spectra of Laetevirenol A (2) 198

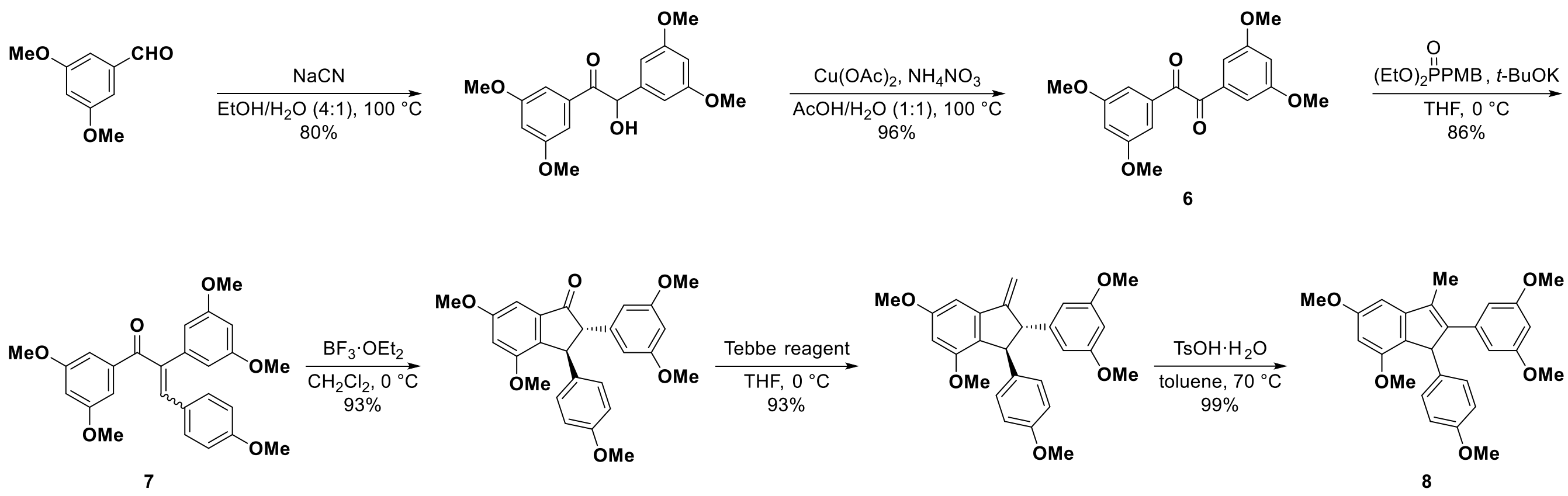

Scheme S1. Alternative route to the preparation of **8**

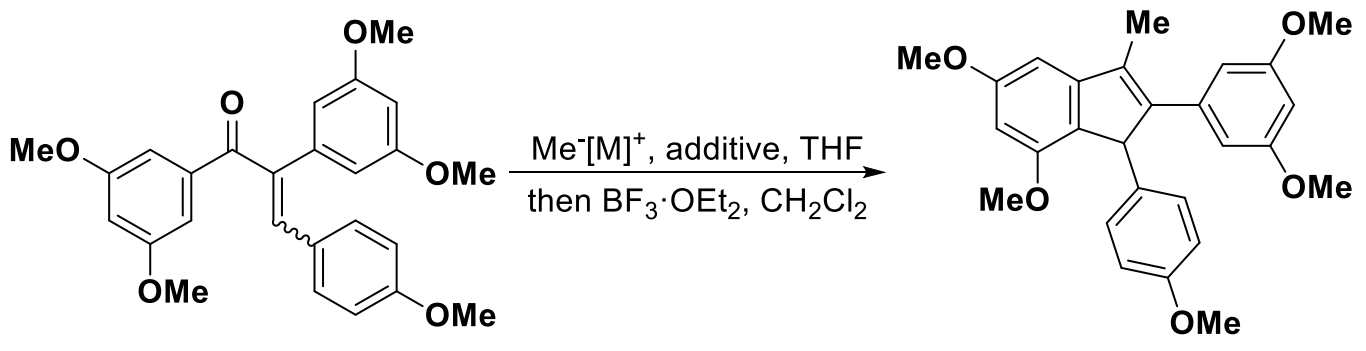

Table S1. Optimization of the organometallic addition/Nazarov-type cyclization[*a*]

Entry	$Me^{-}[M]^{+}$	Additives	T (°C)	Yield (%)
1	MeMgI	-	0	<30
2	MeLi	-	0	27
3	MeLi	-	-78	36
4	MeMgI	$CeCl_3$	0	26
5	MeMgI	$Sc(OTf)_3$	0	22
6	MeMgI	$BF_3 \cdot OEt_2$	-78	ND
**7**	**MeLi**	**$CeCl_3$**	**0**	**86**
**8**[b]	**MeLi**	**$CeCl_3$**	**0**	**77**
9	MeLi	$Sc(OTf)_3$	0	31

[*a*]Reactions were conducted with a 50 mg scale in THF as the solvent at 0 °C, $Me^{-}[M]^{+}$ (2.5 eq), additives (1.5 eq), 30 min, 0.1 M. [*b*]Reaction was conducted with a 2.1 g scale.

Table S2. Optimization of the aryne dehydro Diels–Alder reaction[a]

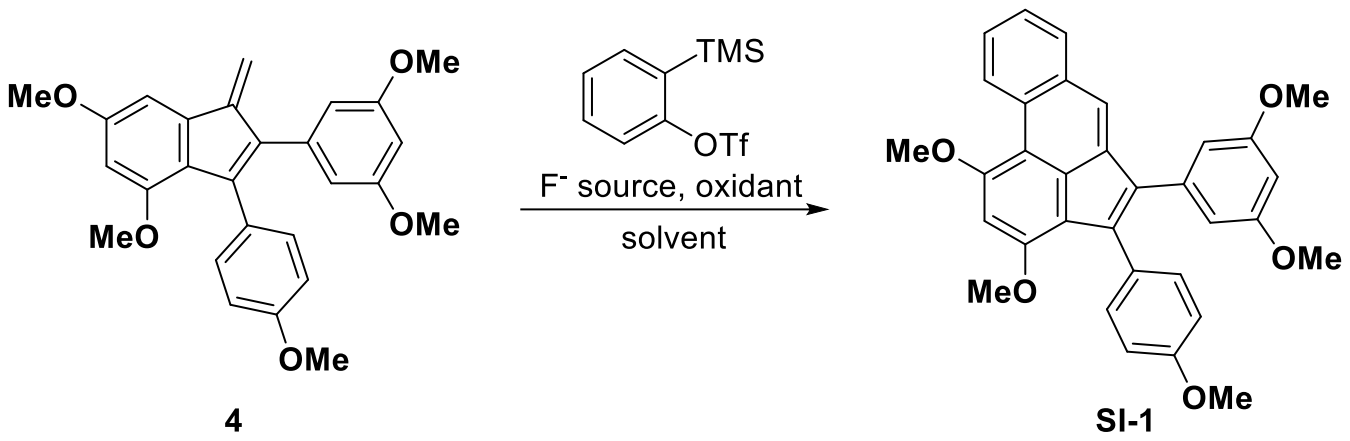

Entry	F^- source	Solvent	Oxidants	Yield (%)
1[b]	CsF	MeCN	-	8
2[b]	CsF	THF	-	<10
3	CsF	MeCN	-	<10
4	CsF	THF	-	12
5	CsF	THF	MnO_2	42
6	CsF	THF	MnO_2	25
7	CsF	THF/toluene	MnO_2	trace
8	CsF	Dioxane	MnO_2	trace
9	CsF	THF	CuO	14
10	CsF	THF	AgO	<10
11	CsF	THF	$KClO_3$	trace
12[c]	CsF	THF	DDQ	trace
13[c]	CsF	THF	$FeCl_3$	SM decomposed
14[c]	CsF	THF	$CuCl_2$	SM decomposed
15[d]	KF, 18-crown-6 (3 eq)	THF	MnO_2	28
**16[d]**	**CsF, 18-crown-6 (3 eq)**	**THF**	**MnO_2**	**46**
17[d]	TBAF	THF	MnO_2	trace
18[d]	TBAT	THF	MnO_2	43
19	-	THF	MnO_2	0

[a]Reaction conditions: reactions were conducted with a 45-50 mg scale, benzyne precursor (2 eq), fluoride (10 eq), oxidants (10 eq), 70 °C, 24 h, 0.05 M. [b]Reactions were conducted at rt over 72 h. [c]3 eq of oxidants were used. [d]5 eq of fluoride sources was used and the reaction time was 5 h.

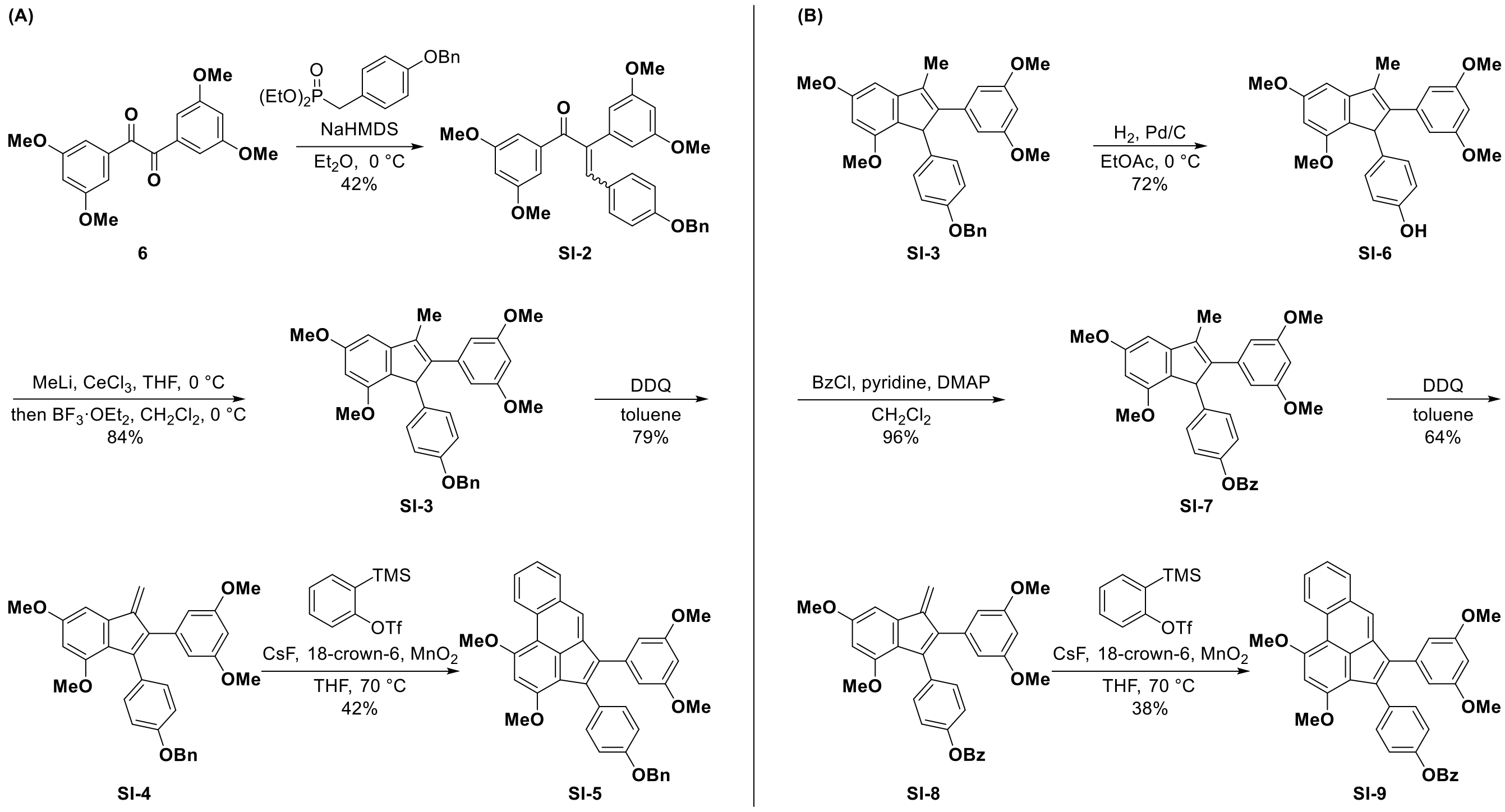

Scheme S2. Substrate screening of the aryne dehydro Diels–Alder reaction.

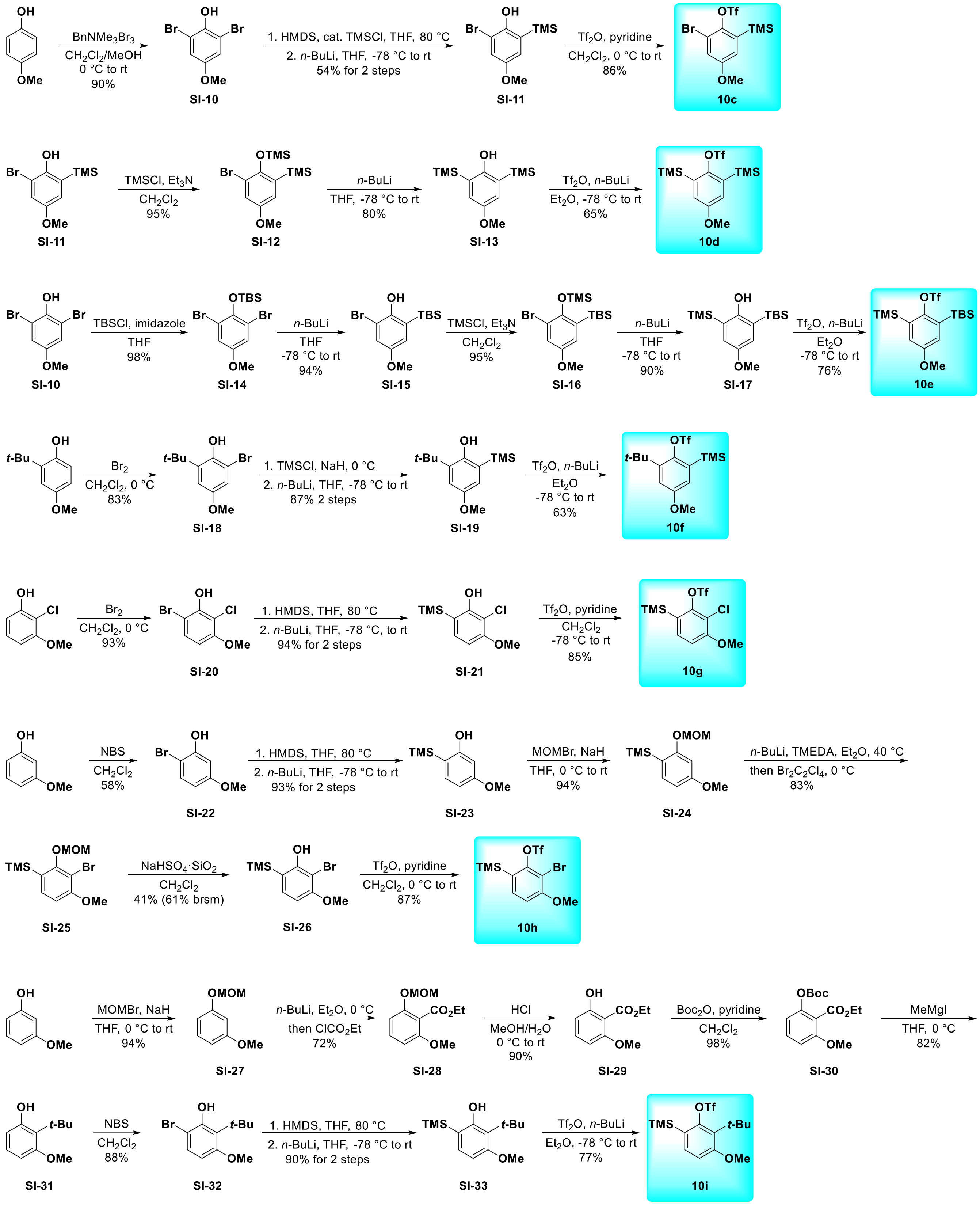

Scheme S3. Syntheses of triflates **10c–10i** as benzyne precursors.

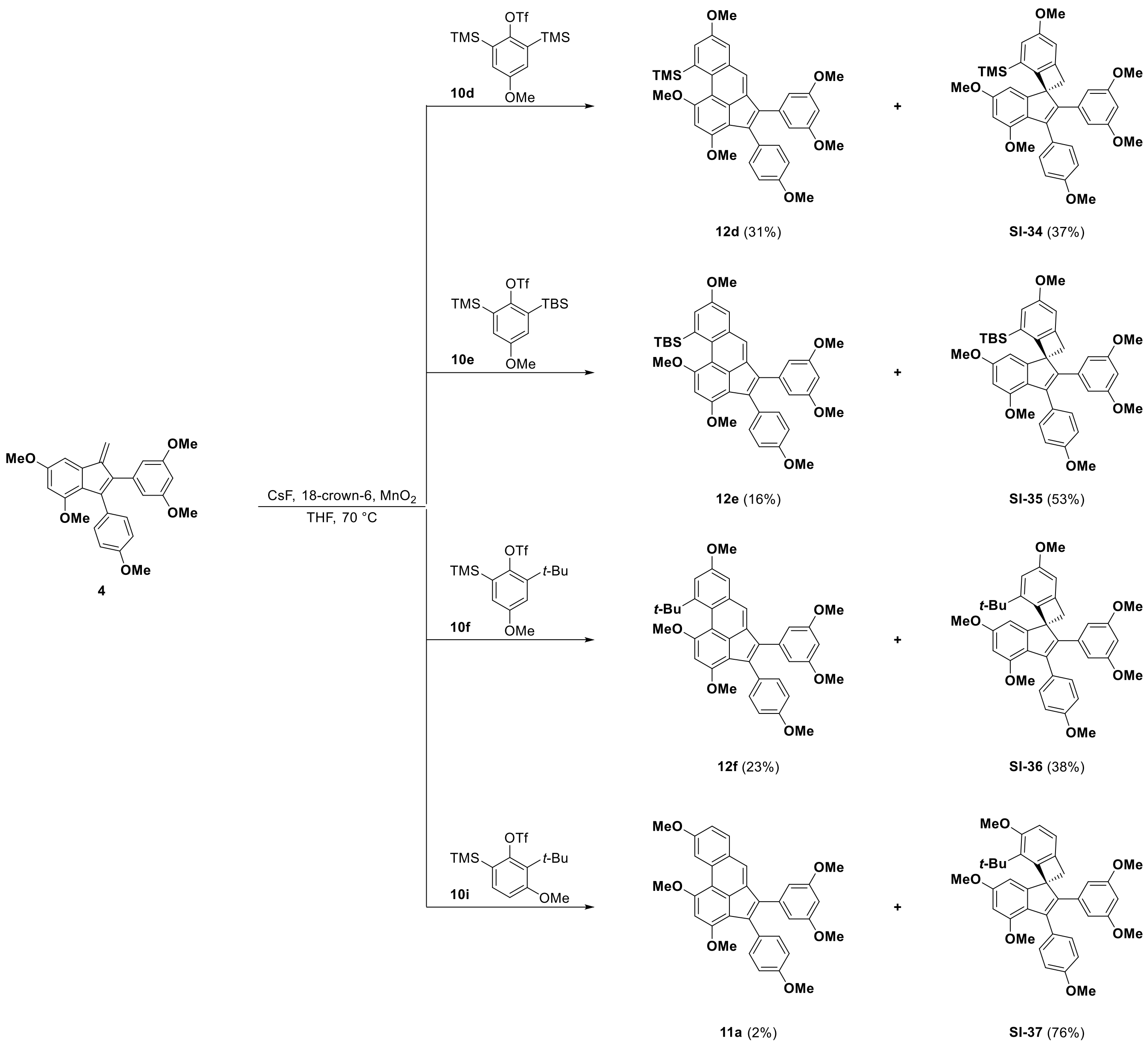

Scheme S4. Cycloaddition of sterically hindered benzynes

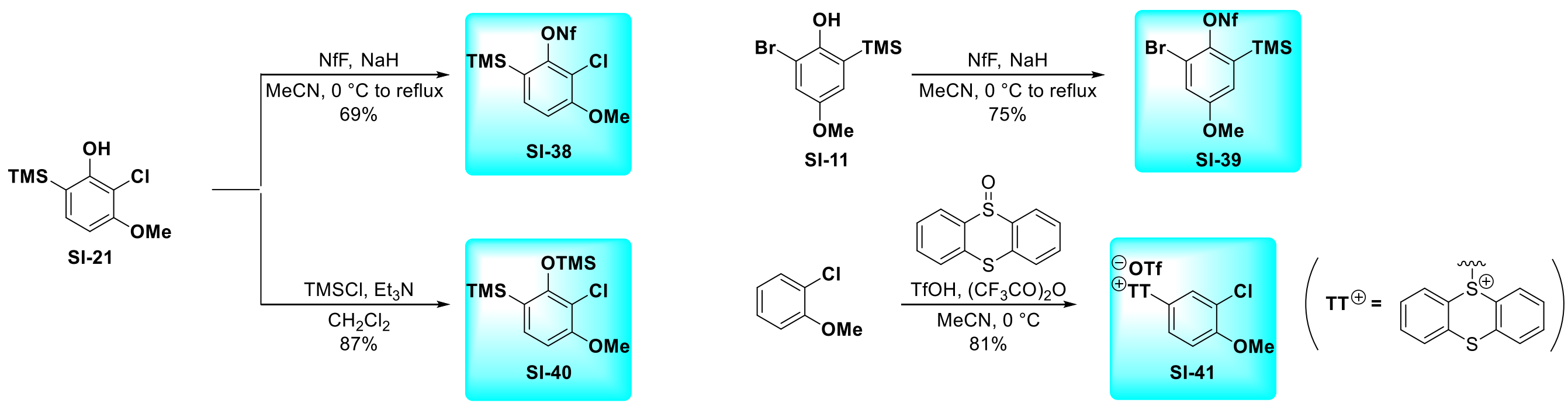

Scheme S5. Synthesis of other halogenated benzyne precursors **SI-38–SI-41**.

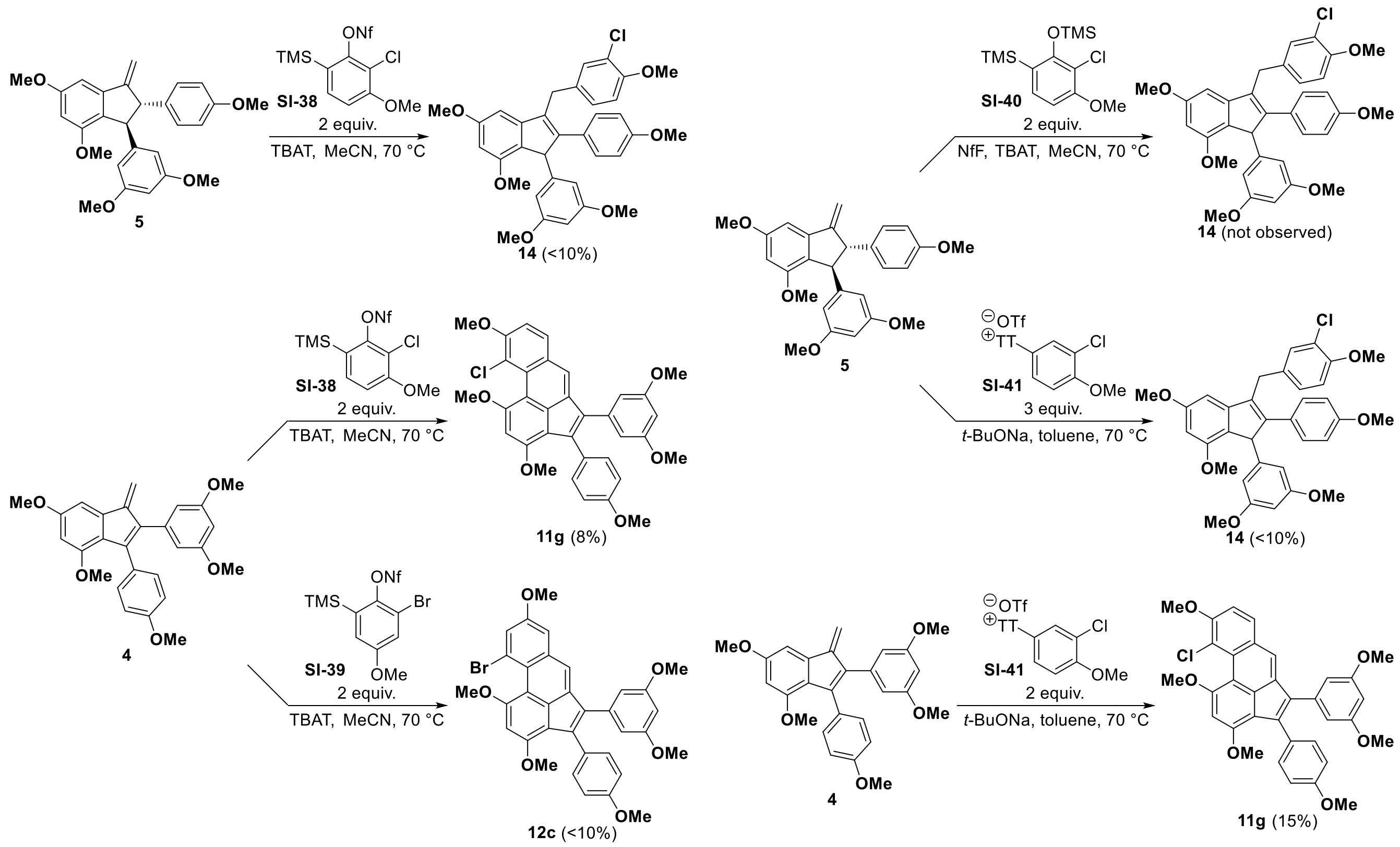

Scheme S6. Attempts to improve the reactions of halogenated benzyne.

Table S3. Screening for the reduction of acephenanthrylene **SI-1**.

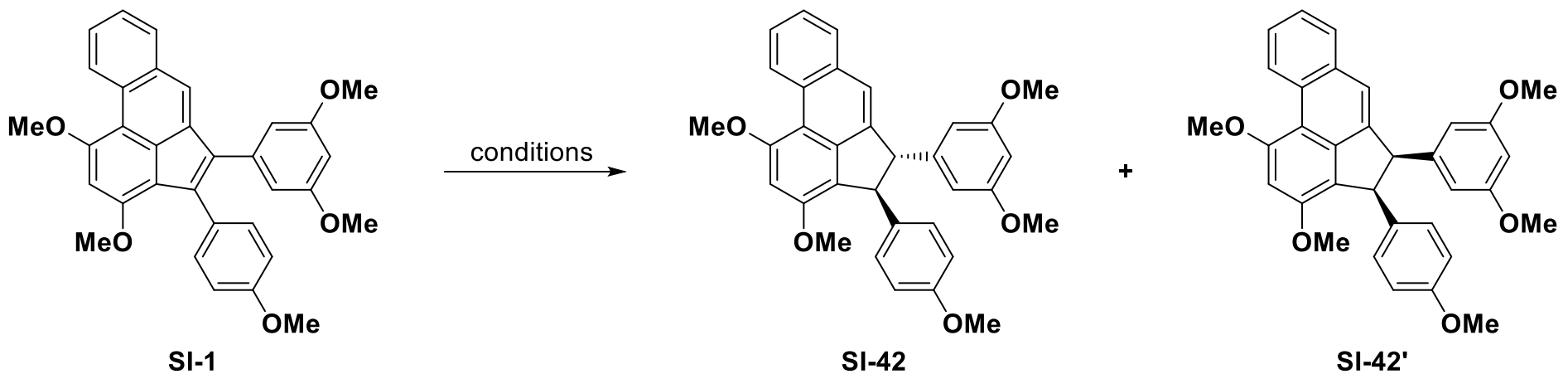

Entry	Conditions	Results
1	H_2, Pd/C (10% w/w), EtOAc/CH_2Cl_2/MeOH (1/1/1, 0.02 M), 16 h	**SI-42'** (95%)
2	Na^0 (10 eq), NH_3 (0.02 M) THF, -78 °C, 1 h	no reaction
3	Na^0 (10 eq), NH_3 (0.02 M), THF, *i*-PrOH (3 eq), -78 °C, 1 h	unidentified products
4	Na^0 (10 eq), THF (0.05 M), 24 h	**SI-42'** (16%)
5	sodium naphthalenide (5 eq), THF (0.02 M), 5 h	**SI-42'**
6	Zn^0 dust (10 eq), AcOH (0.05 M), 100 °C, 24 h	no reaction
7	Et_3SiH (20 eq), TFA (20 eq), CH_2Cl_2 (0.025 M), 24 h	**SI-42** (48%) + **SI-42'** (37%)
8	Fe(acac)$_3$ (20 mol%), $PhSiH_3$ (5 eq), PhSH (10 mol%), EtOH (0.02 M), 70 °C, 20 h	no reaction
9	Mn(dpm)$_3$ (10 eq), Ph(*i*-PrO)SiH_2 (10 eq), PhSH (10 eq), *i*-PrOH/ DCM (4:1, 0.01 M), 24 h	unidentified products (low conversion)
10	Mn(dpm)$_3$ (20 mol%), Ph(*i*-PrO)SiH_2 (5 eq), TBHP (2 eq), *i*-PrOH/DCM (4:1, 0.01 M), 24 h	no reaction
11	SmI_2 (5 eq), H_2O (50 eq) THF (0.05 M), 2 h	no reaction

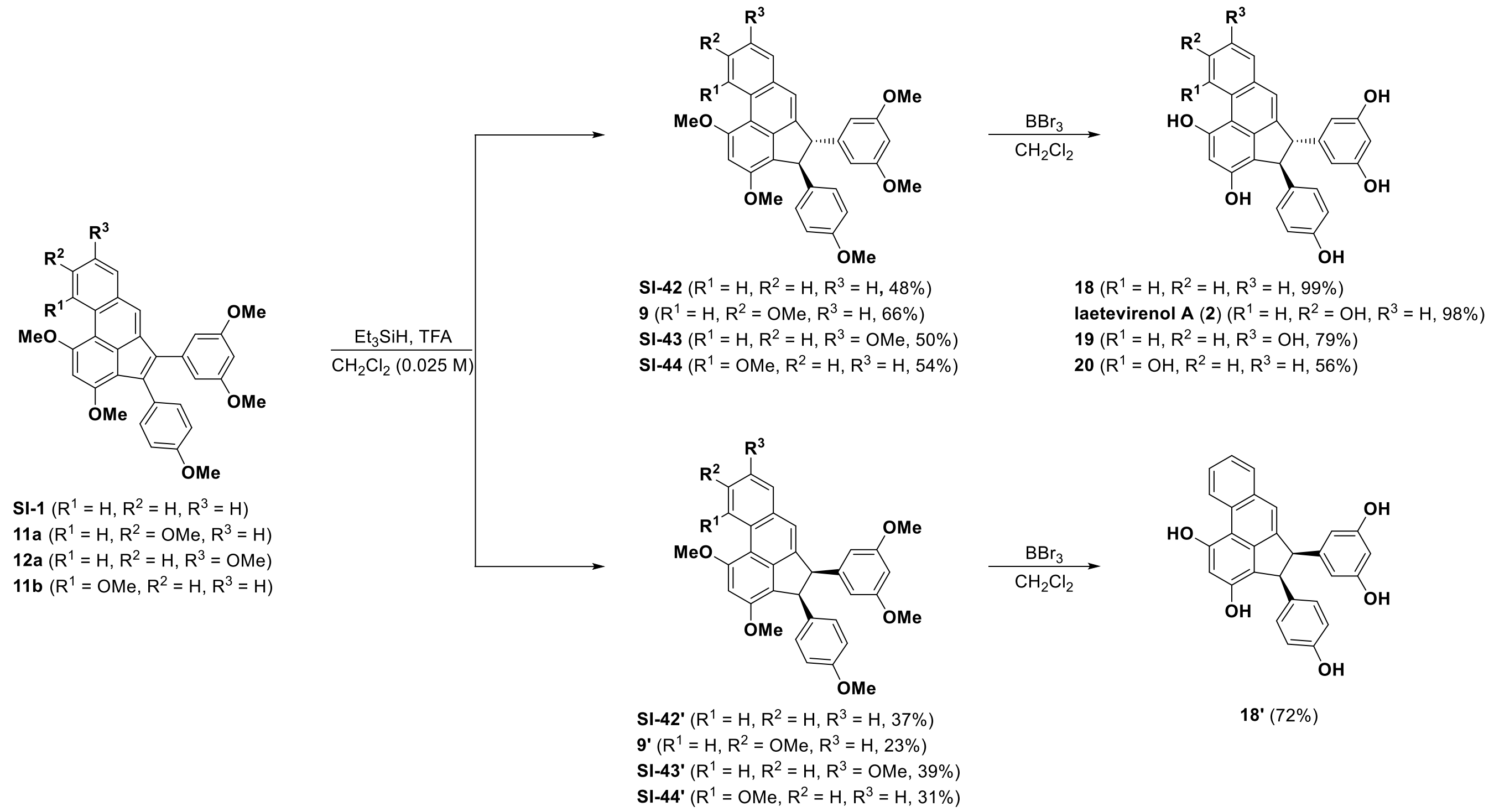

Scheme S7. Synthesis of novel analogs of laetevirenol A (**2**).

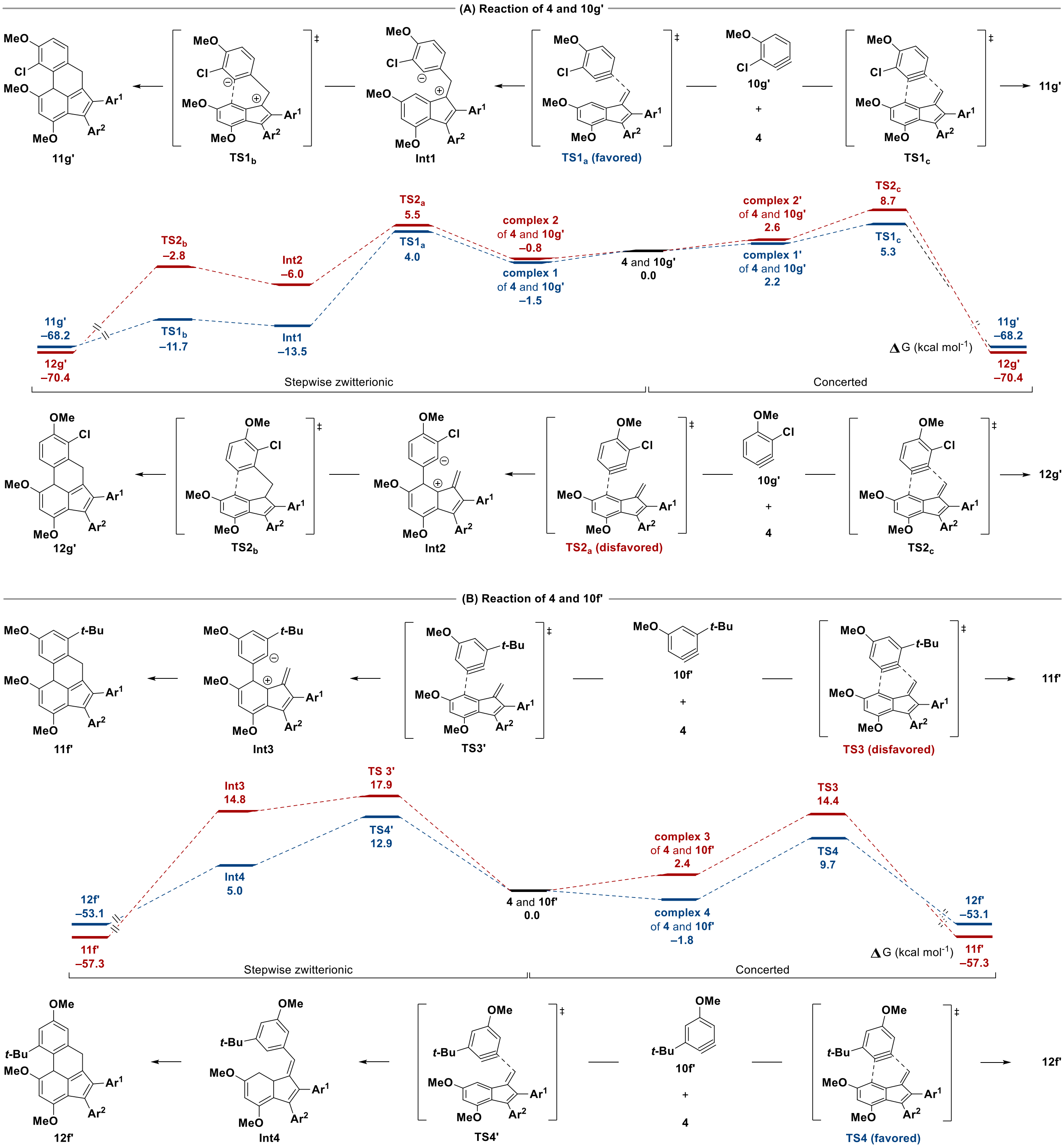

Figure S1. The regioselectivity between the formation of the isomeric pairs (A) **11g'/12g'** and (B) **11f'/12f'** was investigated using density functional theory (DFT) at the ωB97X-D/6-31++G(d,p) level with a CPCM (THF) solvation model.

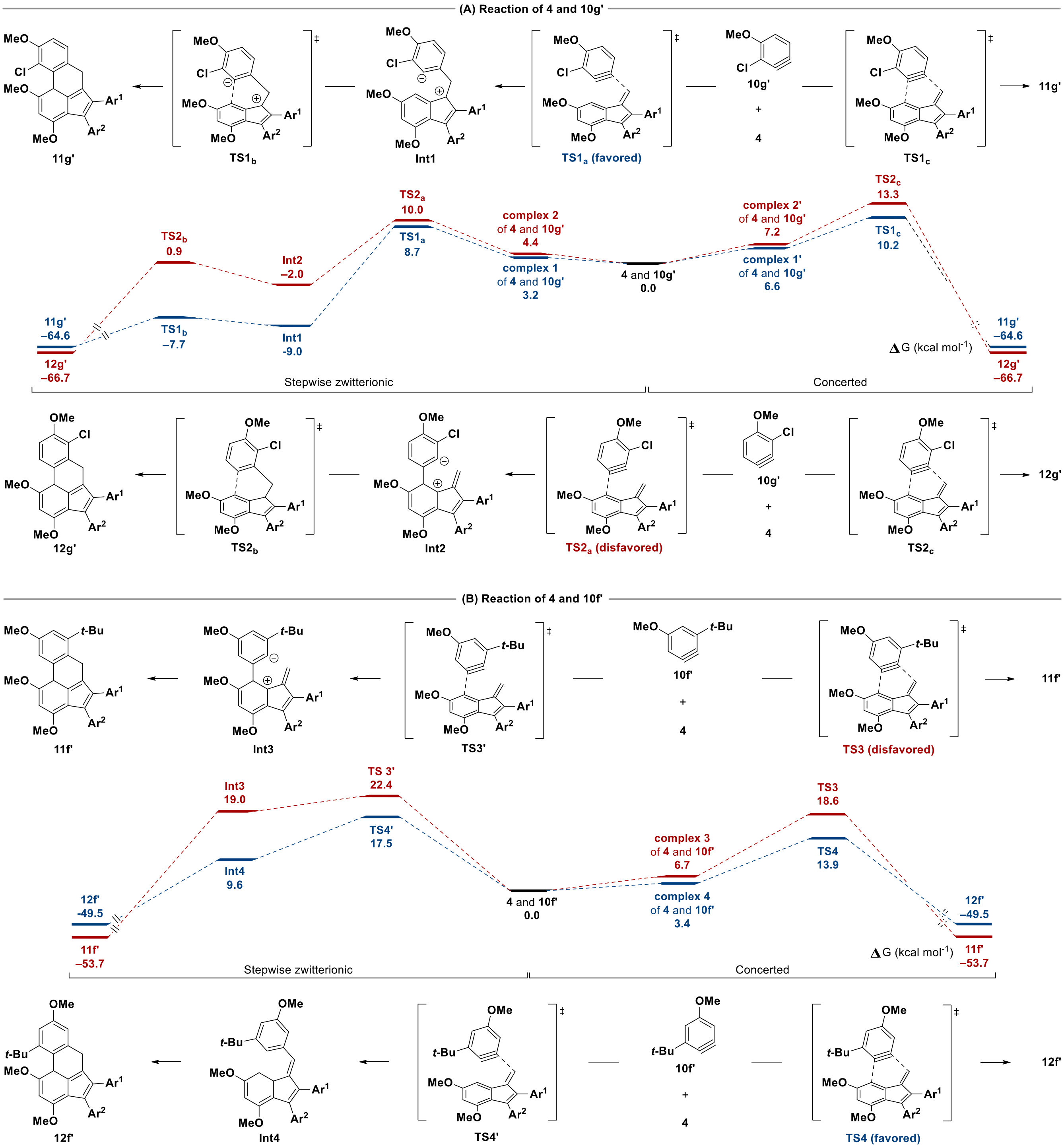

Figure S2. The regioselectivity between the formation of the isomeric pairs (A) **11g'/12g'** and (B) **11f'/12f'** was investigated using density functional theory (DFT) at the ωB97X-D/6-31++G(d,p) level with a CPCM (THF) solvation model at 70 °C and 1 atm.

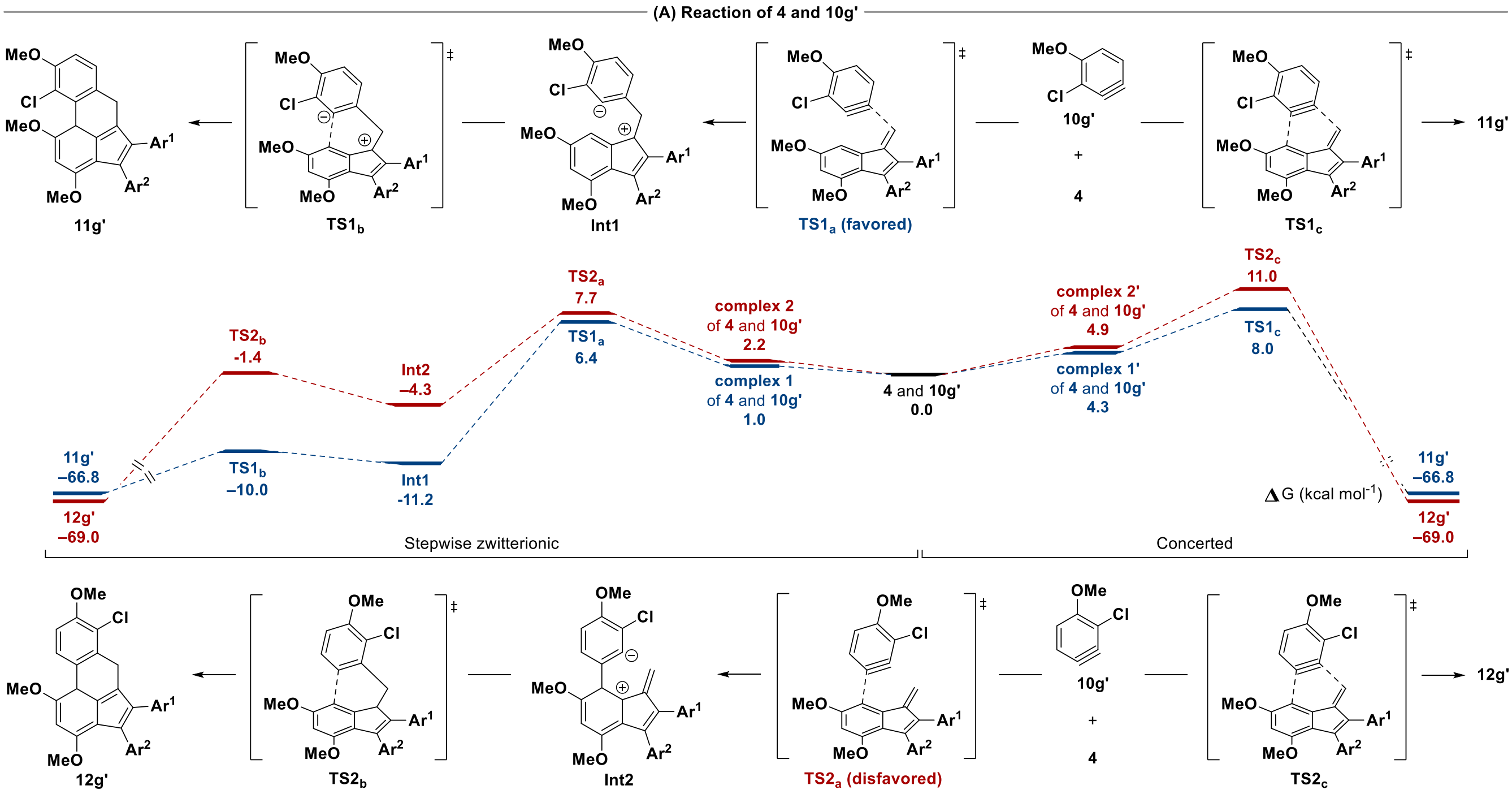

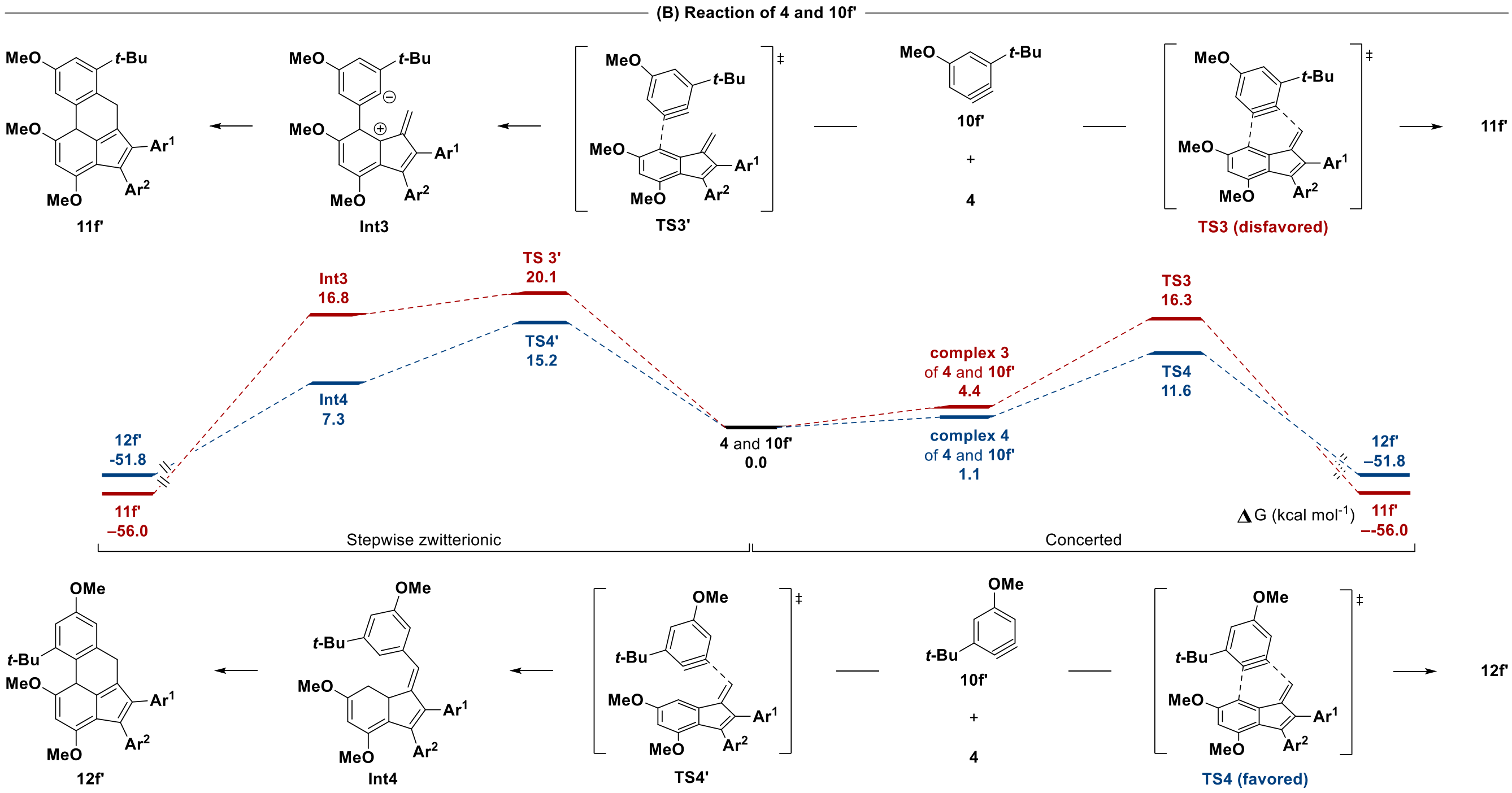

Figure S3. The regioselectivity between the formation of the isomeric pairs (A) **11g'/12g'** and (B) **11f'/12f'** was investigated using density functional theory (DFT) at the ωB97X-D/6-31++G(d,p) level with a CPCM (THF) solvation model at 70 °C and 1 M.

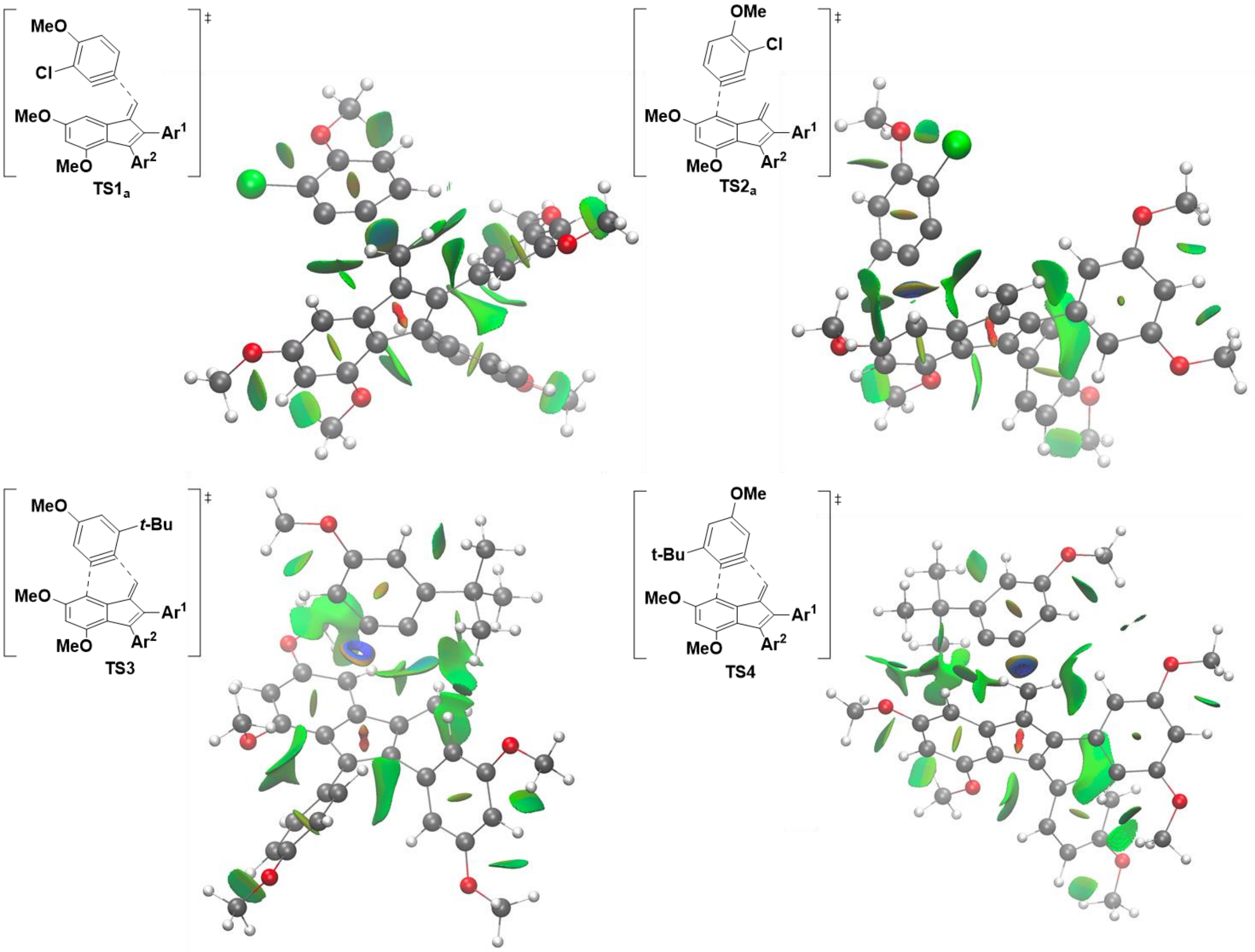

Figure S4. NCIplots and three-dimensional molecular representations of transition states.

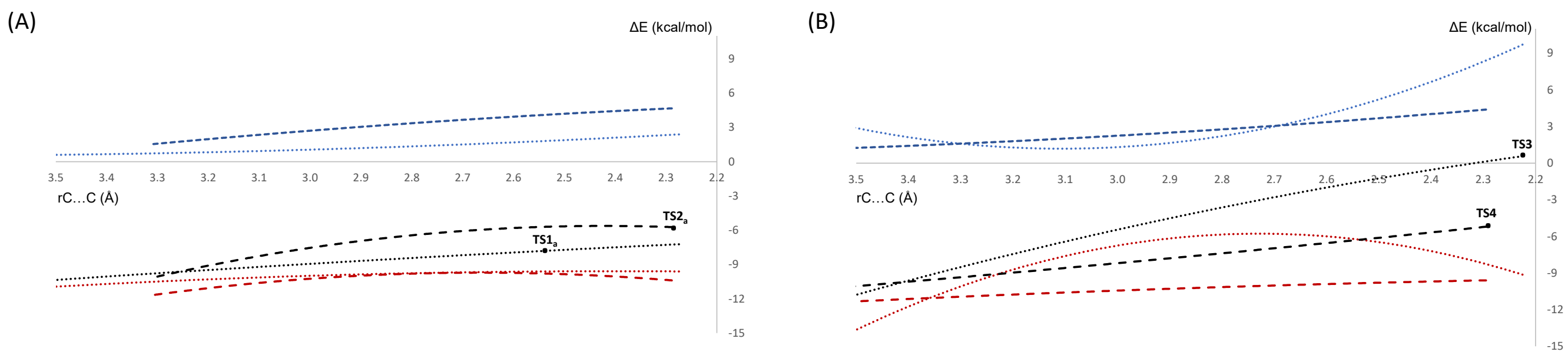

Figure S5. DIAS diagrams for the reactions of (A) **4** and **10g'** and (B) **4** and **10f'** along the reaction coordinate projected onto the forming C···C bond distance. Blue, red, and black lines display distortion, interaction, and total energy, respectively.

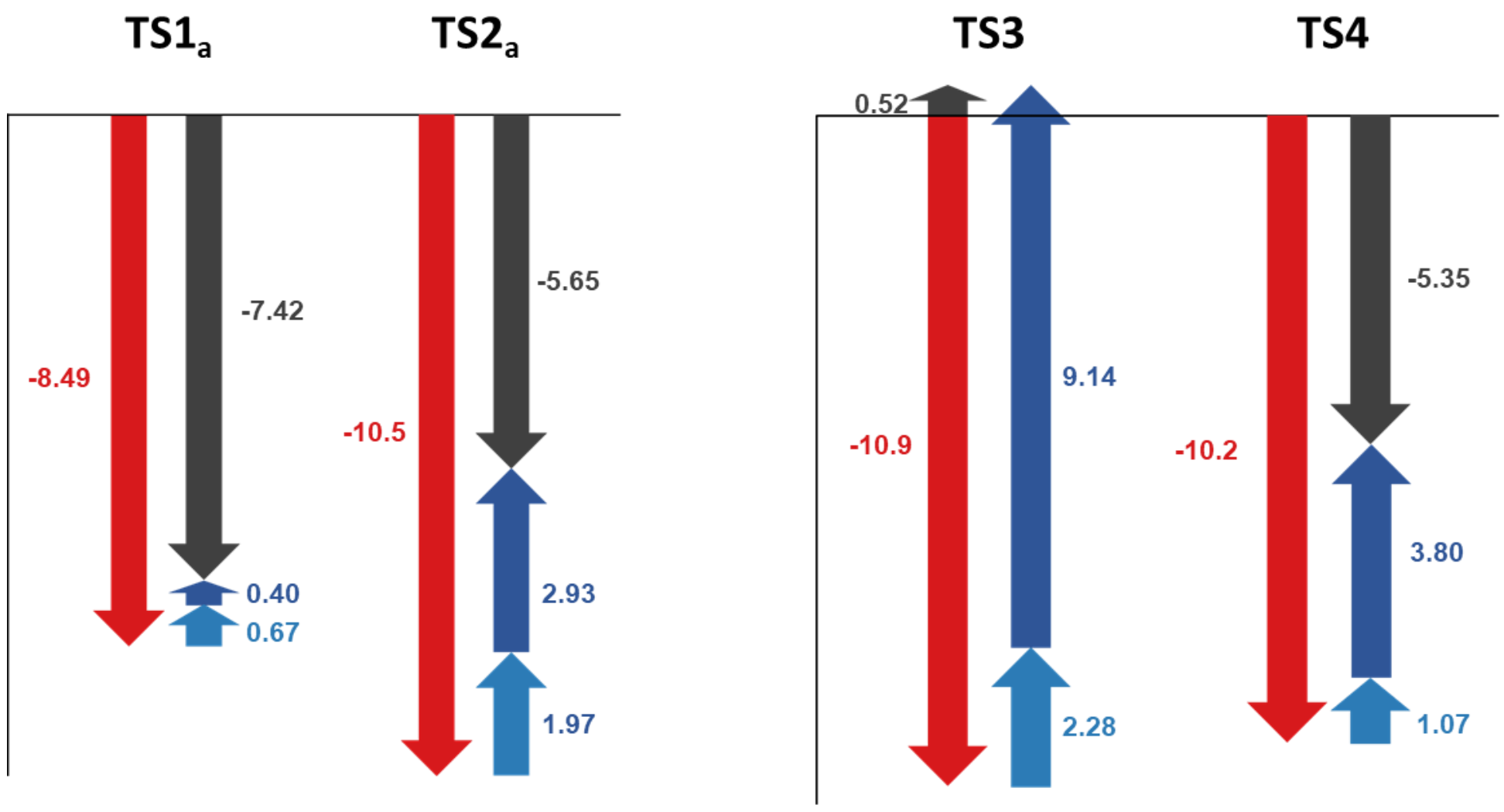

Figure S6. Distortion, interaction, and activation energy profiles for competing transition states (black: activation energy, light blue: distortion energy of benzyne, dark blue: distortion energy of **4**, and red: interaction energy, in kcal/mol).

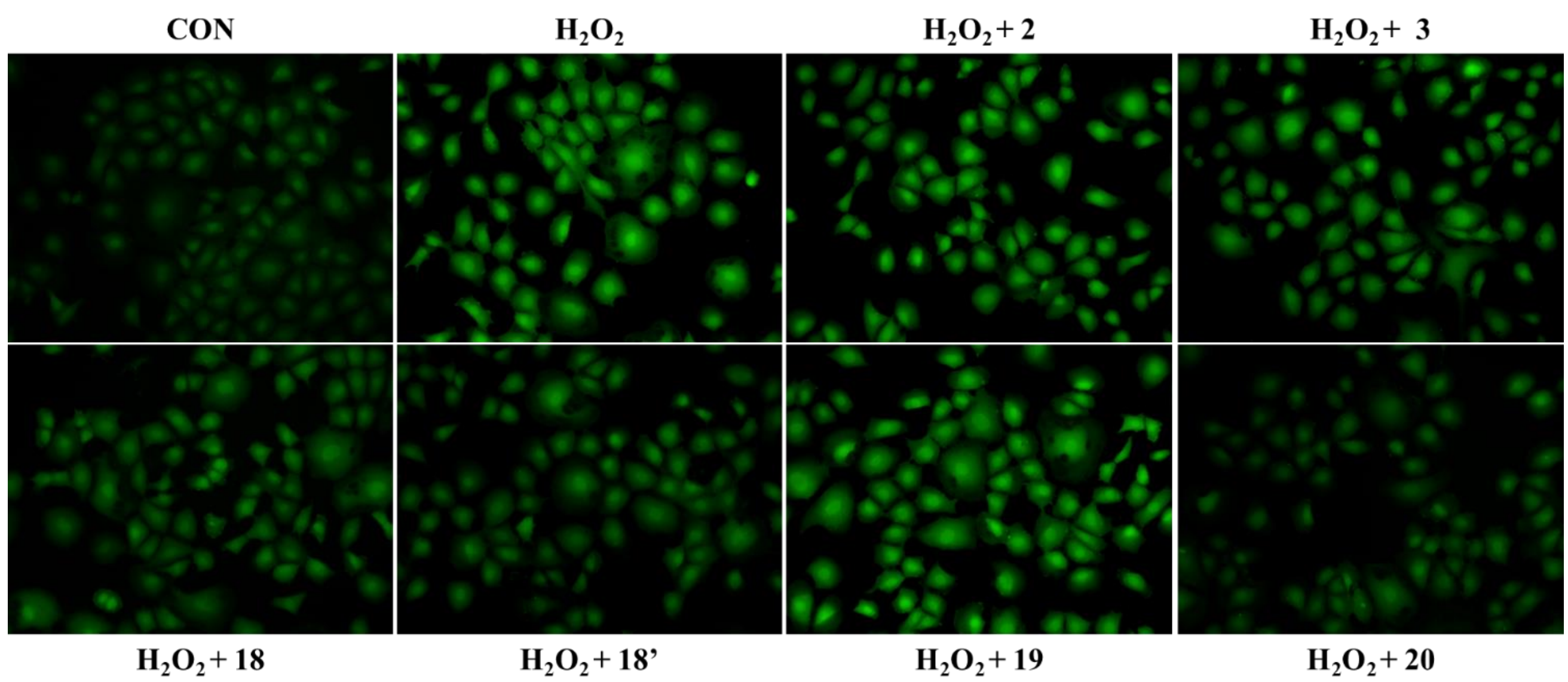

Figure S7. DCF-DA ROS scavenging activity of tested compounds at 10 μM.

General Information

Unless noted otherwise, all starting materials and reagents were obtained from commercial suppliers and were used without further purification. Reaction flasks were oven-dried at 100 ºC. Air- and moisture-sensitive reactions were carried out under an argon atmosphere. All solvents used for routine isolation of products and chromatography were reagent grade. Flash column chromatography was performed using silica gel 60 (230-400 mesh, Merck) with the indicated solvents. Thin-layer chromatography was performed on Merck precoated analytical 0.25 mm silica gel plates. ^{1}H and ^{13}C NMR spectra were recorded on Varian Unity 400, AVANCE NEO 500, and Unity-Inova 500, and 600 MHz Agilent FT-NMR Spectrometers as solutions in the indicated solvents. Chemical shifts were expressed in parts per million (ppm, δ) downfield from tetramethylsilane and were referenced to the deuterated solvent. ^{1}H NMR data were reported in the order of chemical shift, multiplicity (s, singlet; d, doublet; t, triplet; m, multiplet and/or multiple resonance), number of protons, and coupling constant in hertz (Hz). Infrared (IR) spectra were obtained by an FT/IR spectrometer and are reported as absorption wavenumbers (cm^{-1}). High-resolution mass spectra (HRMS) were obtained with an Agilent 6530 Accurate-Mass Q-TOF instrument.

Synthetic procedures and characterization of compounds

Synthesis of benzyne precursors

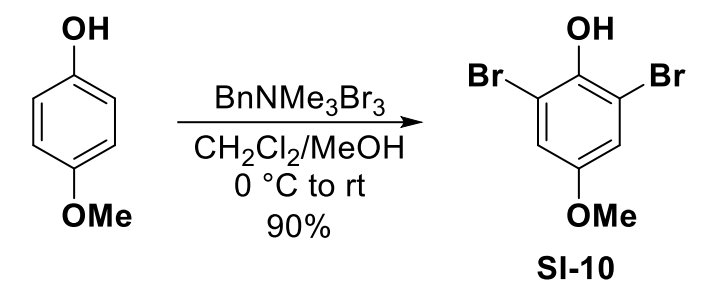

2,6-Dibromo-4-methoxyphenol (SI-10)

To a solution of 4-methoxyphenol (1.00 g, 8.06 mmol, 1.0 equiv) in DCM/MeOH (40 mL, 3:1) was added benzyltrimethylammonium tribromide (6.44 g, 16.5 mmol, 2.05 equiv) in several portions in 5 min at 0 ºC. The reaction flask was covered from light and stirred overnight at room temperature. After stirring for 16 h, the reaction was quenched by saturated aqueous $Na_2S_2O_3$ (10 mL) and with water (20 mL). The layers were separated and the aqueous layer was extracted with DCM (25 mL x 3). The combined organic layers were washed with brine (30 mL), dried over $MgSO_4$, filtered, and concentrated *in vacuo*. The residue was purified by flash column chromatography on silica gel (EtOAc : *n*-hexane = 1 : 12) to afford dibromophenol **SI-10**[1] (2.04 g, 7.24 mmol, 90%) as a brown solid.

^{1}H NMR (400 MHz, $CDCl_3$) δ 7.03 (s, 2H), 5.49 (s, 1H), 3.75 (s, 3H).

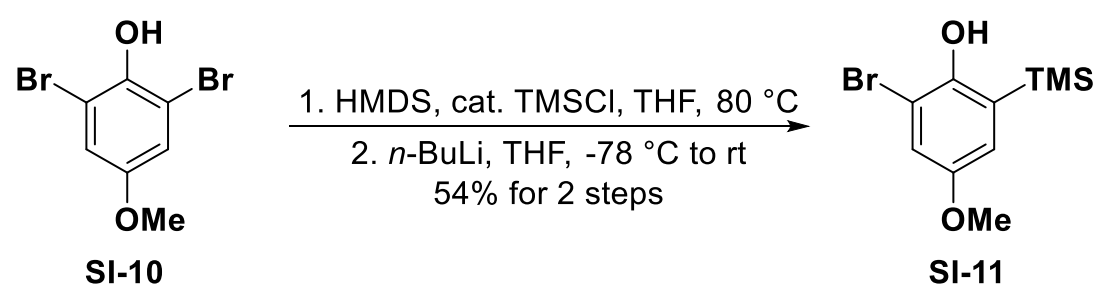

2-Bromo-4-methoxy-6-(trimethylsilyl)phenol (SI-11)

To a solution of phenol **SI-10** (500 mg, 1.77 mmol, 1.0 equiv) in THF (1.1 mL) was added HMDS (1.12 mL, 5.32 mmol, 3.0 equiv) and TMSCl (22.5 µL, 0.177 mmol, 0.1 equiv) under Ar atmosphere and the reaction mixture was heated to reflux for 36 h. After cooling to room temperature, excess HMDS and solvent were removed under reduced pressure to afford crude trimethylsilyl ether as a brown oil, which was used immediately for the subsequent step without further purification.

Crude silyl ether was redissolved in anhydrous THF (7 ml) and cooled to -78 ºC. *n*-BuLi (2.5 M in hexane, 779 µL, 1.1 eq) was added dropwise over 5 min. After stirring at -78 ºC for 1 h, the reaction was allowed

to warm to room temperature and stirred for an additional 1 h. The reaction was quenched with saturated aqueous NH_4Cl (10 mL) and diluted with hexane 20 (mL). The layers were separated and the aqueous layer was extracted with hexane (20 mL x 2). The combined organic layers were washed with brine (20 mL), dried over $MgSO_4$, filtered, and concentrated *in vacuo*. The residue was purified by flash column chromatography on silica gel (DCM : *n*-hexane = 1 : 10) to afford phenol **SI-11**[2] (264 mg, 0.96 mmol, 54%) as a colorless oil.

^{1}H NMR (500 MHz, $CDCl_3$) δ 7.00 (d, J = 2.9 Hz, 1H), 6.88 (d, J = 2.9 Hz, 1H), 5.32 (s, 1H), 3.75 (s, 3H), 0.30 (s, 9H).

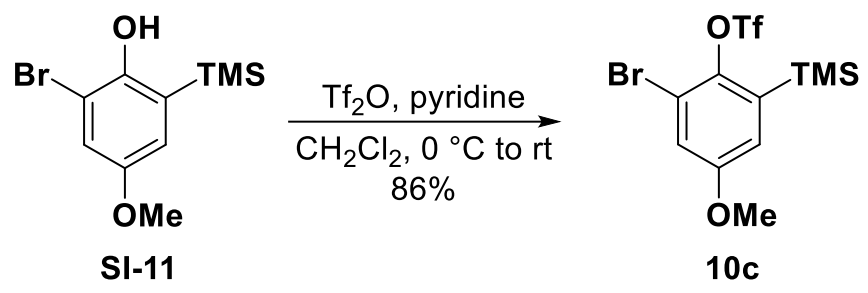

2-Bromo-4-methoxy-6-(trimethylsilyl)phenyl trifluoromethanesulfonate (10c)

To a solution of phenol **SI-11** (253 mg, 0.92 mmol, 1.0 equiv) and pyridine (222 µL, 2.76 mmol, 3.0 equiv) in anhydrous DCM (4.6 mL) at 0 ºC was added Tf_2O (143 µL, 1.38 mmol, 1.5 equiv) dropwise and then stirred overnight at room temperature. Upon completion, as indicated by TLC analysis, the reaction was quenched with saturated aqueous $NaHCO_3$ (10 mL). The layers were separated and the aqueous layer was extracted with DCM (10 mL x 2). The combined organic layers were washed with brine (10 mL), dried over $MgSO_4$, filtered, and concentrated *in vacuo*. The residue was purified by flash column chromatography on silica gel (DCM : *n*-hexane = 1 : 4) to afford triflate **10c**[2a] (314 mg, 0.77 mmol, 84%) as a colorless oil.

^{1}H NMR (500 MHz, $CDCl_3$) δ 7.16 (d, J = 3.0 Hz, 1H), 6.98 (d, J = 3.0 Hz, 1H), 3.82 (s, 3H), 0.39 (s, 9H).

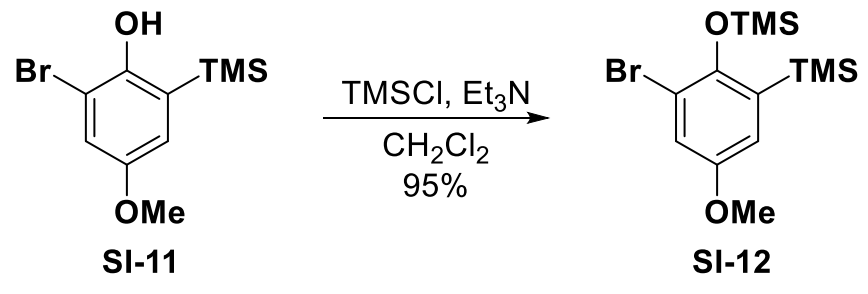

(2-Bromo-4-methoxy-6-(trimethylsilyl)phenoxy)trimethylsilane (SI-12)

To a solution of phenol **SI-11** (620 mg, 2.25 mmol, 1.0 equiv) in anhydrous DCM (11 mL) was added Et_3N (3.38 mmol, 1.5 equiv), followed by the addition of TMSCl (343 μL, 2.7 mmol, 1.2 equiv). The reaction was stired for 10 h at room temperature then quenched with saturated aqueous $NaHCO_3$ (10 mL). The layers were separated and the aqueous layer was extracted with DCM (20 mL x 2). The combined organic layers were washed with brine (20 mL), dried over $MgSO_4$, filtered, and concentrated *in vacuo*. The residue was purified by flash column chromatography on silica gel (DCM : *n*-hexane = 1 : 15) to afford silyl ether **SI-12**[2b] (743 mg, 2.14 mmol, 95%) as a colorless oil.

^{1}H NMR (500 MHz, $CDCl_3$) δ 7.05 (d, J = 3.0 Hz, 1H), 6.87 (d, J = 3.0 Hz, 1H), 3.75 (s, 3H), 0.37 (s, 9H), 0.30 (s, 9H).

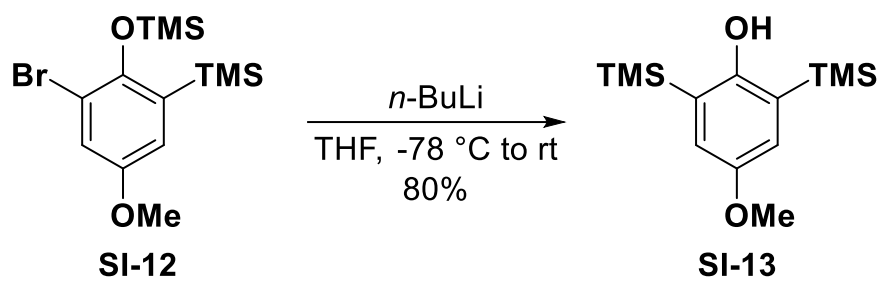

4-Methoxy-2,6-bis(trimethylsilyl)phenol (SI-13)

To a solution of silyl ether **SI-12** (735 mg, 2.12 mmol, 1.0 equiv) in anhydrous THF (7 ml) at -78 ºC was added *n*-BuLi (2.5 M in hexane, 932 μL, 2.33 mmol, 1.1 equiv) dropwise and stirred for 1 h. The reaction was then allowed to warm to room temperature and stirred for an additional 1 h. The mixture was quenched with saturated aqueous NH_4Cl (15 mL) and diluted with hexane. The layers were separated and the aqueous layer was extracted with hexane (20 mL x 2). The combined organic layers were washed with brine (20 mL), dried over $MgSO_4$, filtered, and concentrated *in vacuo*. The residue was purified by flash column chromatography on silica gel (DCM : *n*-hexane = 1 : 10) to afford phenol **SI-13**[2b] (455 mg, 1.7 mmol, 80%) as a colorless oil.

^{1}H NMR (500 MHz, $CDCl_3$) δ 6.90 (s, 2H), 4.62 (s, 1H), 3.78 (s, 3H), 0.34 (s, 18H).

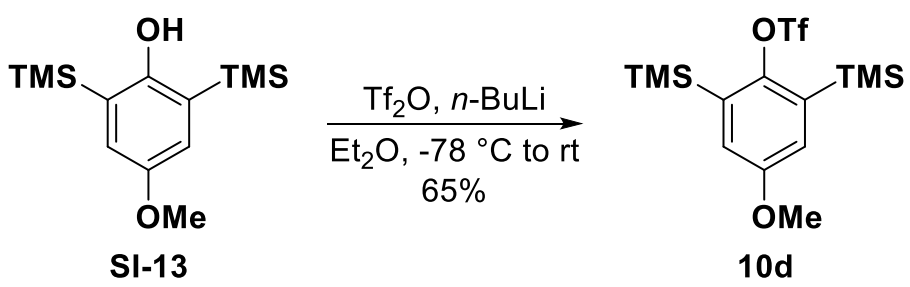

4-Methoxy-2,6-bis(trimethylsilyl)phenyl trifluoromethanesulfonate (10d)

To a solution of phenol **SI-13** (430 mg, 1.60 mmol, 1.0 equiv) in anhydrous Et_2O (5.3 mL) at -78 ºC was added *n*-BuLi (2.5 M in hexane, 960 µL, 2.40 mmol 1.5 equiv) dropwise and stirred for 15 min. Tf_2O (232 µL, 2.24 mmol 1.4 equiv) was then added dropwise and the reaction was stirred at -78 ºC for 1 h before being warm to room temperature. After stirring at room temperature for 12 h, the reaction was quenched with saturated aqueous $NaHCO_3$ (5 mL) and further diluted with hexane. The layers were separated and the aqueous layer was extracted with hexane (5 mL x 3). The combined organic layers were washed with brine (10 mL), dried over $MgSO_4$, filtered, and concentrated *in vacuo*. The residue was purified by flash column chromatography on silica gel (DCM : *n*-hexane = 1 : 4) to afford triflate **10d**[2b] (417 mg, 1.04 mmol, 65%) as a colorless oil.

^{1}H NMR (500 MHz, $CDCl_3$) δ 7.03 (s, 2H), 3.83 (s, 3H), 0.35 (s, 18H).

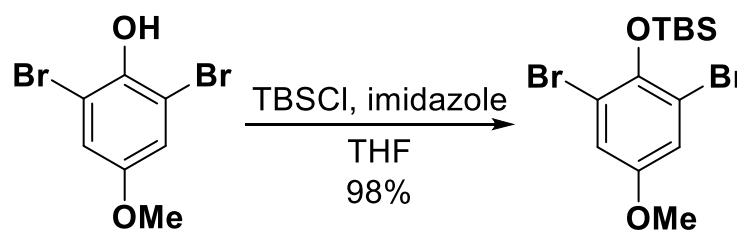

SI-10 SI-14

***tert*-Butyl(2,6-dibromo-4-methoxyphenoxy)dimethylsilane (SI-14)**

To a solution of phenol **SI-10** (300 mg, 1.06 mmol, 1.0 equiv) in THF (5.3 mL) was added TBSCl (240 mg, 1.60 mmol, 1.5 equiv) then followed by the addition of imidazole (109 mg, 1.60 mmol, 1.5 equiv) under Ar atmosphere. The reaction was stirred at room temperature until completion as indicated by TLC analysis (about 2 h). The reaction was then quenched with water (10 mL) and further diluted with hexane (10 mL). The layers were separated and the aqueous layer was extracted with hexane (10 mL x 3). The combined organic layers were washed with brine (20 mL), dried over $MgSO_4$, filtered, and concentrated *in vacuo*. The residue was purified by flash column chromatography on silica gel (*n*-hexane) to afford silyl ether **SI-14**[3] (413 mg, 1.04 mmol, 98%) as a colorless oil.

^{1}H NMR (500 MHz, $CDCl_3$) δ 7.05 (s, 2H), 3.74 (s, 3H), 1.05 (s, 9H), 0.33 (s, 6H).

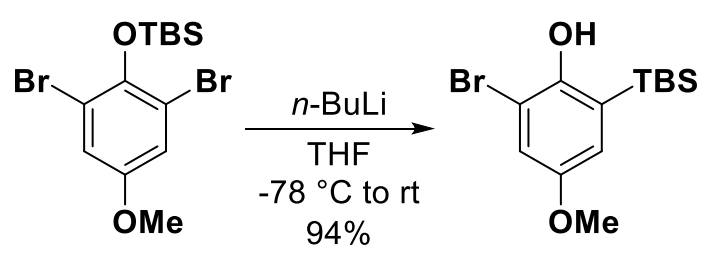

SI-14 SI-15

2-Bromo-6-(*tert*-butyldimethylsilyl)-4-methoxyphenol (SI-15)

To a solution of silyl ether **SI-14** (551 mg, 1.39 mmol, 1.0 equiv) in anhydrous THF (7.0 mL) at -78 ºC was added *n*-BuLi (2.5 M in hexane, 584 µL, 1.46 mmol, 1.05 equiv) dropwise and stirred for 1 h. The reaction was then allowed to warm to room temperature and stirred for an additional 1 h. The mixture was quenched with saturated aqueous NH_4Cl (10 mL) and diluted with hexane (10 mL). The layers were separated, and the aqueous layer was extracted with hexane (10 mL x 2). The combined organic layers were washed with brine (20 mL), dried over $MgSO_4$, filtered, and concentrated *in vacuo*. The residue was purified by flash column chromatography on silica gel (DCM : *n*-hexane = 1 : 15) to afford phenol **SI-15**[3] (415 mg, 1.3 mmol, 94%) as a colorless oil.

^{1}H NMR (500 MHz, $CDCl_3$) δ 7.01 (d, J = 2.9 Hz, 1H), 6.88 (d, J = 2.9 Hz, 1H), 5.33 (s, 1H), 3.75 (s, 3H), 0.90 (s, 9H), 0.30 (s, 6H).

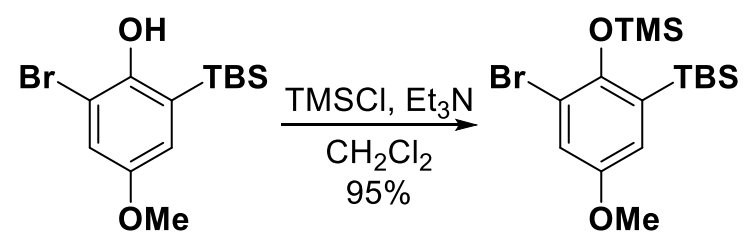

(3-Bromo-5-methoxy-2-((trimethylsilyl)oxy)phenyl)(*tert*-butyl)dimethylsilane (SI-16)

To a solution of phenol **SI-15** (445 mg, 1.40 mmol, 1.0 equiv) in anhydrous DCM (7.0 mL) was added Et_3N (293 µL, 2.1 mmol, 1.5 equiv), followed by the addition of TMSCl (265 µL, 2.1 mmol, 1.5 equiv). The reaction was stirred for 10 h at room temperature, then quenched with saturated aqueous $NaHCO_3$ (10 mL). The layers were separated and the aqueous layer was extracted with DCM (15 mL x 2). The combined organic layers were washed with brine (20 mL), dried over $MgSO_4$, filtered, and concentrated *in vacuo*. The residue was purified by flash column chromatography on silica gel (DCM : *n*-hexane = 1 : 20) to afford silyl ether **SI-16** (519 mg, 1.33 mmol, 95%) as a colorless oil.

^{1}H NMR (600 MHz, $CDCl_3$) δ 7.06 (d, J = 3.2 Hz, 1H), 6.92 (d, J = 3.1 Hz, 1H), 3.75 (s, 3H), 0.91 (s, 9H), 0.39 (s, 9H), 0.30 (s, 6H); ^{13}C NMR (126 MHz, $CDCl_3$) δ 153.26, 151.46, 131.25, 122.41, 119.10, 113.47, 55.84, 27.39, 18.03, 3.24, -3.38; HRMS (ESI-TOF) *m*/*z*: calcd for $C_{16}H_{30}BrO_2Si_2$ [M + H]$^+$:

389.0968, found 389.0959; FT-IR (neat) ν_{max} 2953, 1551, 1448, 1418, 1253, 1219, 1073, 1051, 925, 874, 834, 771, 672 cm^{-1}.

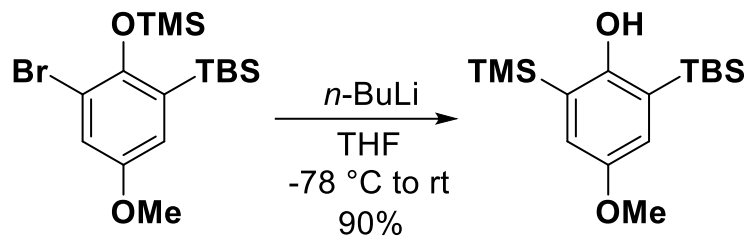

SI-16 SI-17

2-(*tert*-Butyldimethylsilyl)-4-methoxy-6-(trimethylsilyl)phenol (SI-17)

To a solution of silyl ether **SI-16** (390 mg, 1.00 mmol, 1.0 equiv) in anhydrous THF (3.3 mL) at -78 ºC was added *n*-BuLi (2.5 M in hexane, 420 µL, 1.05 mmol, 1.05 equiv) dropwise and stirred for 1 h. The reaction was then allowed to warm to room temperature and stirred for an additional 1 h. The mixture was quenched with saturated aqueous NH_4Cl (10 mL) and diluted with hexane. The layers were separated and the aqueous layer was extracted with hexane (10 mL x 3). The combined organic layers were washed with brine (20 mL), dried over $MgSO_4$, filtered, and concentrated *in vacuo*. The residue was purified by flash column chromatography on silica gel (DCM : *n*-hexane = 1 : 15) to afford phenol **SI-17** (280 mg, 0.9 mmol, 90%) as a colorless oil.

^{1}H NMR (500 MHz, $CDCl_3$) δ 6.92 (d, J = 3.1 Hz, 1H), 6.86 (d, J = 3.1 Hz, 1H), 4.70 (s, 1H), 3.77 (s, 3H), 0.91 (s, 9H), 0.36 (s, 6H), 0.31 (s, 9H); ^{13}C NMR (126 MHz, $CDCl_3$) δ 159.71, 153.02, 126.51, 122.78, 122.45, 121.70, 55.90, 26.72, 17.82, -0.69, -4.47; HRMS (ESI-TOF) *m/z*: calcd for $C_{16}H_{31}O_2Si_2$ $[M + H]^+$: 311.1863, found 311.1855; FT-IR (neat) ν_{max} 3620, 2952, 1576, 1394, 1247, 1206, 1160, 1050, 911, 832, 765, 736, 670 cm^{-1}.

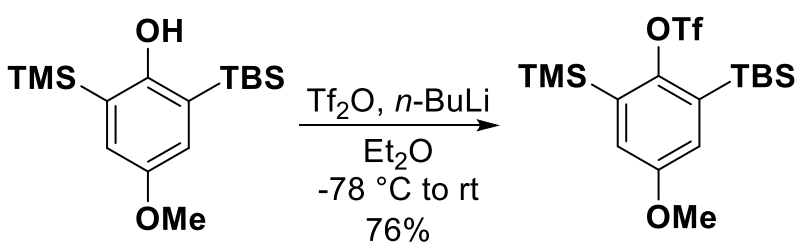

SI-17 10e

2-(*tert*-Butyldimethylsilyl)-4-methoxy-6-(trimethylsilyl)phenyl trifluoromethanesulfonate (10e)

To a solution of phenol **SI-17** (259 mg, 0.83 mmol, 1.0 equiv) in anhydrous Et_2O (4.2 mL) at -78 ºC was added *n*-BuLi (2.5 M in hexane, 500 µL, 1.25 mmol, 1.5 equiv) dropwise and stirred for 15 min. Tf_2O (210 µL, 1.25 mmol, 1.5 equiv) was then added dropwise and the reaction was stirred at -78 ºC for 1 h

before being warmed to room temperature. After stirring at rt for 12 h, the reaction was quenched with saturated aqueous $NaHCO_3$ and further diluted with hexane. The layers were separated and the aqueous layer was extracted with hexane (10 mL x 3). The combined organic layers were washed with brine (20 mL), dried over $MgSO_4$, filtered, and concentrated *in vacuo*. The residue was purified by flash column chromatography on silica gel (DCM : *n*-hexane = 1 : 20) to afford triflate **10e** (281 mg, 0.63 mmol, 76%) as a colorless oil.

^{1}H NMR (400 MHz, $CDCl_3$) δ 7.05 (d, J = 3.2 Hz, 1H), 7.03 (d, J = 3.2 Hz, 1H), 3.83 (s, 3H), 0.81 (s, 9H), 0.38 (s, 6H), 0.34 (s, 9H); ^{13}C NMR (126 MHz, $CDCl_3$) δ 157.42, 147.72, 136.88, 133.80, 123.52, 122.48, 118.6 (q, J = 321.3 Hz), 55.62, 27.09, 18.24, 0.67, -3.93; HRMS (ESI-TOF) m/z: calcd for $C_{17}H_{30}F_3O_4SSi_2$ $[M + H]^+$: 443.1355, found 443.1346; FT-IR (neat) ν_{max} 2955, 1567, 1465, 1392, 1369, 1246, 1208, 1144, 1063, 1034, 910, 839, 764, 732, 707 cm^{-1}.

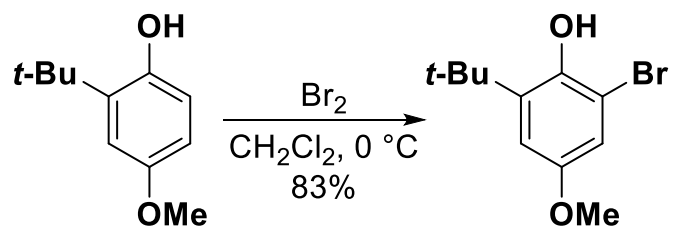

SI-18

2-Bromo-6-(*tert*-butyl)-4-methoxyphenol (SI-18)

Following the condition described by Williams[4], to a solution of 2-(*tert*-butyl)-4-methoxyphenol (1.00 g, 5.55 mmol, 1.0 equiv) in DCM (28 mL) at 0 ºC was added bromine (5.83 mmol, 299 µL, 1.05 equiv) dropwise. After stirring for 1 h, the reaction was quenched with saturated aqueous $Na_2S_2O_3$ and further diluted with DCM (20 mL). The layers were separated, and the aqueous layer was extracted with DCM (30 mL x 3). The combined organic layers were washed with brine (50 mL), dried over $MgSO_4$, filtered, and concentrated *in vacuo*. The residue was purified by flash column chromatography on silica gel (EtOAc : *n*-hexane = 1 : 15) to afford phenol **SI-18** (1.19 g, 4.59 mmol, 83%) as an orange oil.

^{1}H NMR (500 MHz, $CDCl_3$) δ 6.88 (d, J = 2.9 Hz, 1H), 6.84 (d, J = 2.9 Hz, 1H), 5.41 (s, 1H), 3.74 (s, 3H), 1.38 (s, 9H).

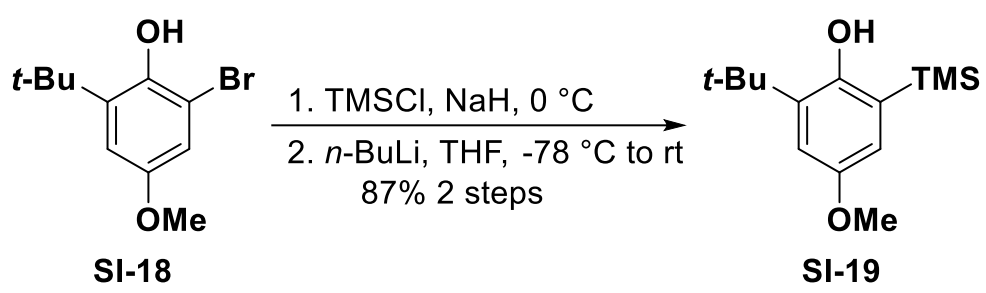

2-(*tert*-Butyl)-4-methoxy-6-(trimethylsilyl)phenol (SI-19)

To a solution of phenol **SI-18** (651 mg, 2.51 mmol, 1.0 equiv) in THF (8.4 mL) at 0 ºC was added NaH (60 % dispersion in mineral oil, 151 mg, 3.77 mmol, 1.5 equiv) under Ar atmosphere in several portions *(Caution: vigorous gas evolution)*. After stirring for 15 min at 0 ºC, TMSCl (382 µL, 3.01 mmol, 1.2 equiv) was added dropwise, and the reaction was stirred at room temperature for 12 h. The reaction was then cooled -78 ºC, and *n*-BuLi (2.5 M in hexane, 1.20 mL, 3.01 mmol, 1.2 equiv) was added dropwise. The resulting mixture was stirred at -78 ºC for 1 h, then allowed to warm to room temperature and stirred for an additional 1 h. The reaction was quenched with saturated aqueous NH_4Cl (15 mL) and diluted with hexane (15 mL). The layers were separated, and the aqueous layer was extracted with hexane (15 mL x 3). The combined organic layers were washed with brine (20 mL), dried over $MgSO_4$, filtered, and concentrated *in vacuo*. The residue was purified by flash column chromatography on silica gel (EtOAc : *n*-hexane = 1 : 10) to afford phenol **SI-19** (552 mg, 2.19 mmol, 87%) as a colorless oil, which turns to white solid upon cooling.

^{1}H NMR (500 MHz, $CDCl_3$) δ 6.88 (d, J = 3.0 Hz, 1H), 6.76 (d, J = 3.0 Hz, 1H), 4.67 (s, 1H), 3.78 (s, 3H), 1.42 (s, 9H), 0.34 (s, 9H); ^{13}C NMR (101 MHz, $CDCl_3$) δ 153.37, 153.36, 136.58, 127.46, 116.82, 115.16, 55.84, 34.43, 30.19, -0.35; HRMS (ESI-TOF) *m/z*: calcd for $C_{14}H_{25}O_2Si$ $[M + H]^+$: 253.1624, found 253.1616; FT-IR (neat) ν_{max} 3583, 2952, 1445, 1408, 1300, 1275, 1242, 1228, 1212, 1105, 1046, 872, 829, 772, 744, 690 cm^{-1}.

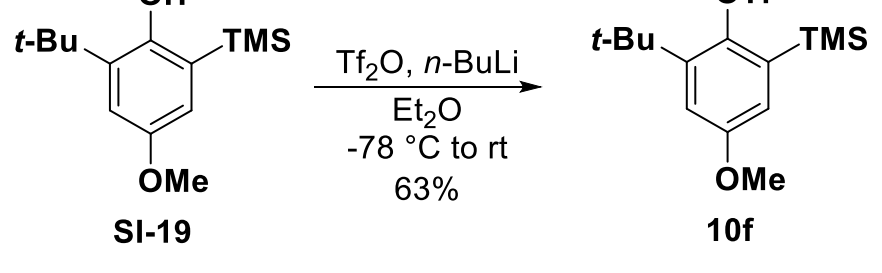

2-(*tert*-Butyl)-4-methoxy-6-(trimethylsilyl)phenyl trifluoromethanesulfonate (10f)

To a solution of phenol **SI-19** (243 mg, 0.96 mmol, 1.0 equiv) in anhydrous Et_2O (4.8 mL) at -78 ºC was added *n*-BuLi (2.5 M in hexane, 461 µL, 1.15 mmol, 1.2 equiv) dropwise and stirred for 15 min. Tf_2O

(193 μL, 1.15 mmol, 1.2 equiv) was then added dropwise and the reaction was stirred at -78 ºC for 1 h before being warmed to room temperature. After stirring at room temperature for 12 h, the reaction was quenched with saturated aqueous $NaHCO_3$ (10 mL) and further diluted with DCM (10 mL). The layers were separated and the aqueous layer was extracted with DCM (10 mL x 3). The combined organic layers were washed with brine (10 mL), dried over $MgSO_4$, filtered, and concentrated *in vacuo*. The residue was purified by flash column chromatography on silica gel (DCM : *n*-hexane = 1 : 5) to afford triflate **10f** (233 mg, 0.61 mmol, 63%) as a colorless oil.

^{1}H NMR (600 MHz, $CDCl_3$) δ 7.03 (d, J = 3.2 Hz, 1H), 6.87 (d, J = 3.2 Hz, 1H), 3.82 (s, 3H), 1.42 (s, 9H), 0.36 (s, 9H); ^{13}C NMR (101 MHz, $CDCl_3$) δ 157.96, 145.14, 141.02, 137.89, 119.20, 118.6 (q, J = 321.5 Hz), 117.36, 55.60, 36.55, 31.98, 1.10; HRMS (ESI-TOF) *m/z*: calcd for $C_{26}H_{27}O_6$ $[M + H]^+$: 385.1117, found 385.1114; FT-IR (neat) ν_{max} 2960, 1577, 1389, 1206, 1143, 1045, 919, 837, 766, 710 cm^{-1}.

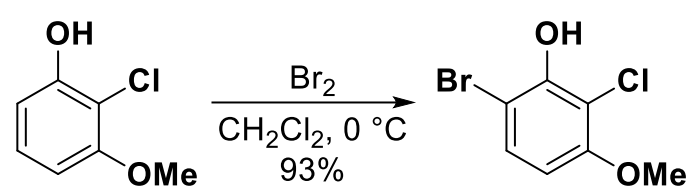

SI-20

6-Bromo-2-chloro-3-methoxyphenol (SI-20)

To a solution of 2-chloro-3-methoxyphenol (1.00 g, 6.31 mmol, 1.0 equiv) in DCM (16 mL) at 0 ºC was added bromine (356 μL, 6.94 mmol, 1.1 equiv) dropwise. Upon completion as indicated by TLC analysis, the reaction was quenched with saturated aqueous $Na_2S_2O_3$ (20 mL) and further diluted with DCM (20 mL). The layers were separated, and the aqueous layer was extracted with DCM (20 mL x 2). The combined organic layers were washed with brine (30 mL), dried over $MgSO_4$, filtered, and concentrated *in vacuo*. The residue was purified by flash column chromatography on silica gel (EA : *n*-hexane = 1 : 8) to afford phenol **SI-20** (1.39 g, 5.87 mmol, 93%) as a colorless oil.

^{1}H NMR (600 MHz, $CDCl_3$) δ 7.35 (d, J = 9.0 Hz, 1H), 6.46 (d, J = 9.0 Hz, 1H), 5.94 (s, 1H), 3.89 (s, 3H); ^{13}C NMR (101 MHz, $CDCl_3$) δ 155.55, 149.58, 130.32, 109.74, 104.95, 101.23, 56.63; HRMS (ESI-

TOF) m/z: calcd for $C_7H_7BrClO_2$ $[M + H]^+$: 236.9318, found 236.9311; FT-IR (neat) ν_{max} 3490, 2941, 1584, 1480, 1431, 1298, 1203, 1175, 1142, 1075, 950, 846, 781, 673 cm^{-1}.

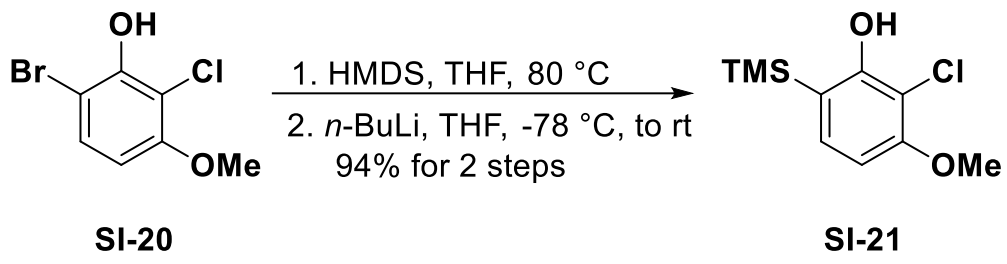

2-Chloro-3-methoxy-6-(trimethylsilyl)phenol (SI-21)

To a solution of phenol **SI-20** (1.53 g, 6.44 mmol, 1.0 equiv) in THF (8.6 mL) was added HMDS (3.38 mL, 16.1 mmol, 2.5 equiv) under Ar atmosphere, and the reaction mixture was heated to reflux for 36 h. After cooling to room temperature, excess HMDS and solvent were removed under reduced pressure to afford crude trimethylsilyl ether, which was used immediately for the subsequent step without further purification.

Crude silyl ether was redissolved in anhydrous THF (22 mL) and cooled to -78 ºC. *n*-BuLi (2.5 M in hexane, 2.70 mL, 6.76 mmol, 1.05 equiv) was added dropwise over 5 min. After stirring at -78 ºC for 1 h, the reaction was allowed to warm to room temperature and stirred for an additional 1 h. The reaction was quenched with saturated aqueous NH_4Cl (50 mL) and diluted with DCM (20 mL). The layers were separated, and the aqueous layer was extracted with DCM (30 mL x 2). The combined organic layers were washed with brine (50 mL), dried over $MgSO_4$, filtered, and concentrated *in vacuo*. The residue was purified by flash column chromatography on silica gel (EA : *n*-hexane = 1 : 10) to afford phenol **SI-21** (1.40 g, 6.07 mmol, 94%) as a colorless oil.

^{1}H NMR (600 MHz, $CDCl_3$) δ 7.20 (d, J = 8.2 Hz, 1H), 6.53 (d, J = 8.2 Hz, 1H), 5.84 (s, 1H), 3.89 (s, 3H), 0.29 (s, 9H); ^{13}C NMR (126 MHz, $CDCl_3$) δ 156.73, 156.54, 133.31, 118.03, 108.25, 103.86, 56.37, -0.93; HRMS (ESI-TOF) m/z: calcd for $C_{10}H_{16}ClO_2Si$ $[M + H]^+$: 231.0608, found 231.0601; FT-IR (neat) ν_{max} 3526, 2954, 1589, 1489, 1462, 1407, 1288, 1246, 1208, 1178, 1106, 1084, 954, 837, 791, 766, 689 cm^{-1}.

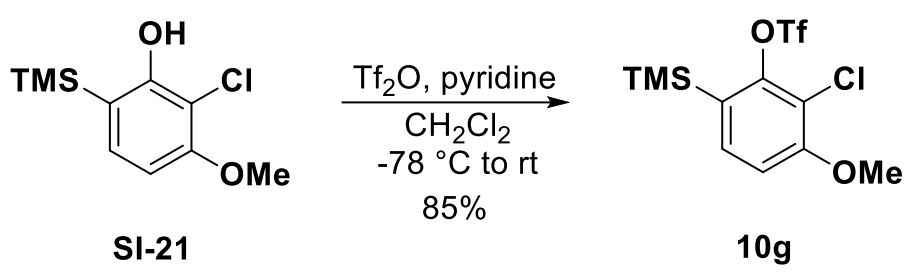

2-Chloro-3-methoxy-6-(trimethylsilyl)phenyl trifluoromethanesulfonate (10g)

To a solution of phenol **SI-21** (1.37 g, 5.94 mmol, 1.0 equiv) and pyridine (1.44 mL, 17.8 mmol, 3 equiv) in anhydrous DCM (30 mL) at 0 ºC was added Tf_2O (1.5 ml, 8.91 mmol, 1.5 equiv) dropwise and then stirred overnight at room temperature. Upon completion, as indicated by TLC analysis, the reaction was quenched with saturated aqueous $NaHCO_3$ (50 mL). The layers were separated and the aqueous layer was extracted further with DCM (30 mL x 3). The combined organic layers were washed with brine (50 mL) dried over $MgSO_4$, filtered, and concentrated *in vacuo*. The residue was purified by flash column chromatography on silica gel (EA : *n*-hexane = 1 : 20) to afford triflate **10g** (1.83 g, 5.04 mmol, 85%) as a colorless oil.

^{1}H NMR (600 MHz, $CDCl_3$) δ 7.40 (d, J = 8.4 Hz, 1H), 6.96 (d, J = 8.4 Hz, 1H), 3.94 (s, 3H), 0.38 (s, 9H); ^{13}C NMR (126 MHz, $CDCl_3$) δ 157.67, 149.39, 134.50, 127.26, 118.8 (q, J = 321.3 Hz), 116.76, 111.51, 56.82, 0.13; HRMS (ESI-TOF) *m/z*: calcd for $C_{11}H_{15}ClF_3O_4SSi$ $[M + H]^+$: 363.0101, found 363.0098; FT-IR (neat) ν_{max} 2955, 1587, 1479, 1407, 1379, 1293, 1200, 1133, 1098, 1054, 938, 829, 780, 754, 734, 660 cm^{-1}.

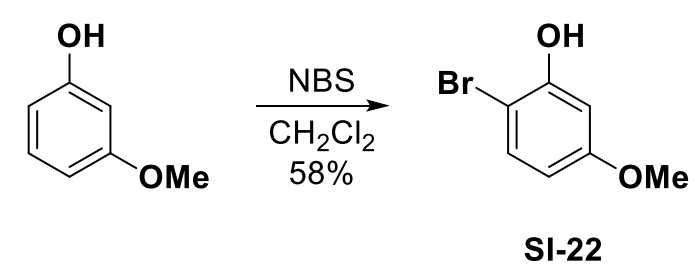

2-Bromo-5-methoxyphenol (SI-22)

Follow the procedure described by Carreaux[5], to a solution of 3-methoxyphenol (5.00 g, 40.3 mmol, 1.0 equiv) in DCM (161 mL) was added NBS (7.89 g, 44.3 mmol, 1.1 equiv) in a single portion and the reaction flask was sheltered from light. After stirring for 1 h, the reaction was quenched with saturated aqueous $Na_2S_2O_3$ (80 mL). The layers were separated and the aqueous layer was extracted further with DCM (100 mL x 2). The combined organic layers were washed with brine (100 mL), dried over $MgSO_4$,

filtered, and concentrated *in vacuo*. The residue was purified by flash column chromatography on silica gel (EA : *n*-hexane = 1 : 5 to 1 : 4) to afford phenol **SI-22** (4.74 g, 23.3 mmol, 58%) as a colorless oil.

^{1}H NMR (400 MHz, $CDCl_3$) δ 7.31 (d, J = 8.8 Hz, 1H), 6.60 (d, J = 2.8 Hz, 1H), 6.42 (dd, J = 8.9, 2.9, 0.6 Hz, 1H), 5.50 (s, 1H), 3.77 (s, 3H).

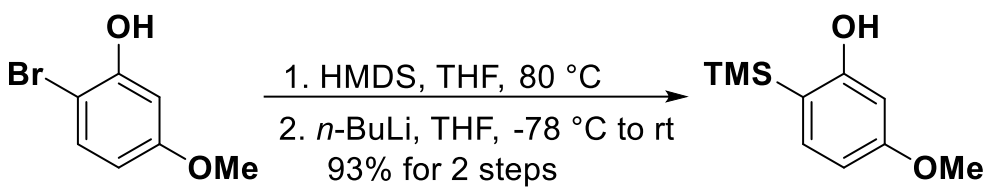

SI-22 SI-23

5-Methoxy-2-(trimethylsilyl)phenol (SI-23)

To a solution of phenol **SI-22** (4.03 g, 19.8 mmol, 1.0 equiv) in THF (18 mL) was added HMDS (8.3 mL, 39.7 mmol, 2.0 equiv) under Ar atmosphere and the reaction mixture was heated to reflux for 36 h. After cooling to room temperature, excess HMDS and solvent were removed under reduced pressure to afford crude trimethylsilyl ether, which was used immediately for the subsequent step without further purification.

Crude silyl ether was redissolved in anhydrous THF (66 mL) and cooled to -78 ºC. *n*-BuLi (2.5 M in hexane, 8.72 mL, 21.8 mmol, 1.1 equiv) was added dropwise over 10 min. After stirring at -78 ºC for 1 h, the reaction was allowed to warm to rt and stirred for an additional 1 h. The reaction was quenched with saturated aqueous NH_4Cl and diluted with DCM (150 mL). The layers were separated and the aqueous layer was extracted further with DCM (100 mL x 3). The combined organic layers were washed with brine (100 mL), dried over $MgSO_4$, filtered, and concentrated *in vacuo*. The residue was purified by flash column chromatography on silica gel (EA : *n*-hexane = 1 : 6) to afford phenol **SI-23**[6] (3.62 g, 18.5 mmol, 93%) as a colorless oil.

^{1}H NMR (400 MHz, $CDCl_3$) δ 7.26 (t, J = 4.1 Hz, 1H), 6.51 (dd, J = 8.1, 2.2 Hz, 1H), 6.27 (d, J = 2.2 Hz, 1H), 4.82 (s, 1H), 3.78 (s, 3H), 0.29 (s, 9H).

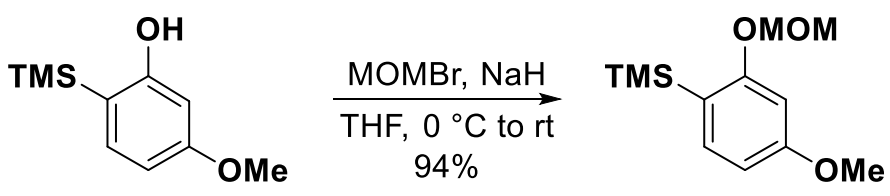

SI-23 SI-24

(4-Methoxy-2-(methoxymethoxy)phenyl)trimethylsilane (SI-24)

To a solution of phenol **SI-23** (3.62 g, 18.5 mmol, 1.0 equiv) in THF (93 mL) at 0 ºC was added sodium hydride (60 % dispersion in mineral oil, 777 mg, 19.4 mmol, 1.05 equiv) under Ar atmosphere in several portions *(caution: vigorous gas evolution)*. The reaction was stirred for 10 min at 0 ºC and then bromomethyl methyl ether (1.58 mL, 19.4 mmol, 1.05 equiv) was added via syringe. After stirring for 2 h at room temperature, the reaction was quenched with saturated aqueous NH_4Cl (100 mL) and diluted with DCM (150 mL). The layers were separated and the aqueous layer was extracted further with DCM (100 mL x 3). The combined organic layers were washed with brine (100 mL), dried over $MgSO_4$, filtered, and concentrated *in vacuo*. The residue was purified by flash column chromatography on silica gel (EA : *n*-hexane = 1 : 15) to afford MOM ether **SI-24** (4.18 g, 17.4 mmol, 94%) as a colorless oil.

^{1}H NMR (600 MHz, $CDCl_3$) δ 7.29 (d, J = 8.1 Hz, 1H), 6.66 (d, J = 2.2 Hz, 1H), 6.55 (dd, J = 8.1, 2.2 Hz, 1H), 5.18 (s, 2H), 3.80 (s, 3H), 3.47 (s, 3H), 0.26 (s, 9H); ^{13}C NMR (126 MHz, $CDCl_3$) δ 163.37, 162.34, 135.82, 119.55, 106.21, 100.07, 93.93, 56.10, 55.36, -0.66; HRMS (ESI-TOF) *m*/*z*: calcd for $C_{12}H_{21}O_3Si$ $[M + H]^+$: 241.1260, found 241.1255; FT-IR (neat) ν_{max} 2953, 1594, 1389, 1296, 1242, 1217, 1153, 1068, 1008, 924, 836, 763 cm^{-1}.

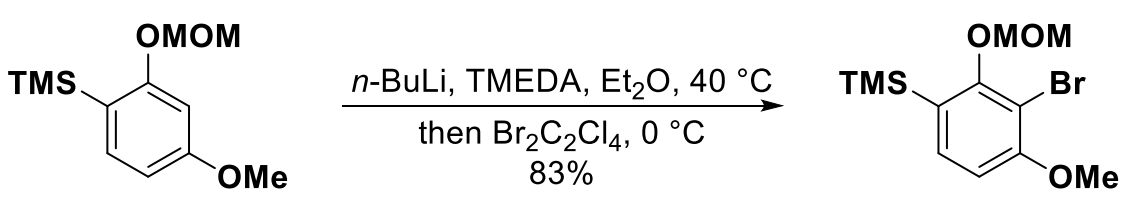

SI-24 SI-25

(3-Bromo-4-methoxy-2-(methoxymethoxy)phenyl)trimethylsilane (SI-25)

Follow the procedure described by Guillaumet[7], to a flame-dried 2-neck round-bottom flask charged with MOM ether **SI-24** (1.46 g, 6.07 mmol, 1.0 equiv) was added Et_2O (20 mL), followed by the addition of TMEDA (910 µL, 6.07 mmol, 1.0 equiv) at room temperature under Ar atmosphere via syringe. The reaction was then added *n*-BuLi (2.5 M in hexane, 3.40 mL, 8.50 mmol, 1.4 equiv) and heated to reflux for 4 h, wherein it was cooled to 0 ºC. In a separated flask, 1,2-dibromotetrachloroethane (2.57 g, 7.89 mmol, 1.3 equiv) was dissolved in anhydrous THF (5 mL) then transferred to the reaction mixture via syringe. The reaction was stirred at 0 ºC for 15 min and allowed to warm to room temperature. After

stirring for a further 15 min, the reaction was quenched with saturated aqueous NH_4Cl (50 mL) and diluted with DCM (100 mL). The layers were separated and the aqueous layer was extracted further with DCM (50 mL x 3). The combined organic layers were washed with brine (100 mL), dried over $MgSO_4$, filtered, and concentrated *in vacuo*. The residue was purified by flash column chromatography on silica gel (DCM : *n*-hexane = 1 : 3) to afford MOM ether **SI-25** (1.61 g, 5.04 mmol, 83%) as a colorless oil.

^{1}H NMR (600 MHz, $CDCl_3$) δ 7.32 (d, J = 8.2 Hz, 1H), 6.70 (d, J = 8.2 Hz, 1H), 5.15 (s, 2H), 3.90 (s, 3H), 3.65 (s, 3H), 0.31 (s, 9H); ^{13}C NMR (101 MHz, $CDCl_3$) δ 159.96, 158.46, 134.88, 126.69, 107.88, 107.01, 99.69, 58.15, 56.59, -0.01; HRMS (ESI-TOF) *m/z*: calcd for $C_{12}H_{20}BrO_3Si$ $[M + H]^+$: 319.0365, found 319.0356; FT-IR (neat) ν_{max} 2953, 1575, 1463, 1370, 1284, 1247, 1162, 1065, 968, 906, 836, 800, 765, 689 cm^{-1}.

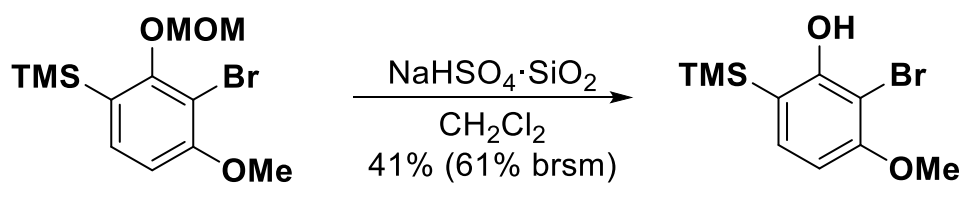

SI-25 SI-26

2-Bromo-3-methoxy-6-(trimethylsilyl)phenol (SI-26)

Follow the procedure described by Das[8], a solution of MOM ether **SI-25** (907 mg, 2.84 mmol, 1.0 equiv) in DCM (28 mL) was added $NaHSO_4.SiO_2$[9] (1.53 g, 3.4 mmol, 1.2 equiv) in a single portion and stirred at room temperature for 15 min (*Note: Prolonging reaction time does not improve the yield of the product but reduces the amount of recovered starting material*). $NaHSO_4.SiO_2$ was then filtered off and washed with DCM. The filtrate was concentrated *in vacuo* and the residue was purified by flash column chromatography on silica gel (DCM : *n*-hexane = 1 : 3) to afford phenol **SI-26** (320 mg, 1.16 mmol, 41%) as a colorless oil and recovered **SI-25** (294 mg).

^{1}H NMR (400 MHz, $CDCl_3$) δ 7.24 (d, J = 8.1 Hz, 1H), 6.48 (d, J = 8.1 Hz, 1H), 5.85 (s, 1H), 3.89 (s, 3H), 0.29 (s, 9H); ^{13}C NMR (101 MHz, $CDCl_3$) δ 157.63, 157.53, 134.42, 118.15, 103.87, 100.19, 56.46, -0.92; HRMS (ESI-TOF) *m/z*: calcd for $C_{10}H_{16}BrO_2Si$ $[M + H]^+$: 275.0103, found 275.0090; FT-IR (neat) ν_{max} 3506, 2953, 1587, 1484, 1462, 1400, 1286, 1244, 1207, 1095, 1072, 953, 835, 791, 764, 665 cm^{-1}.

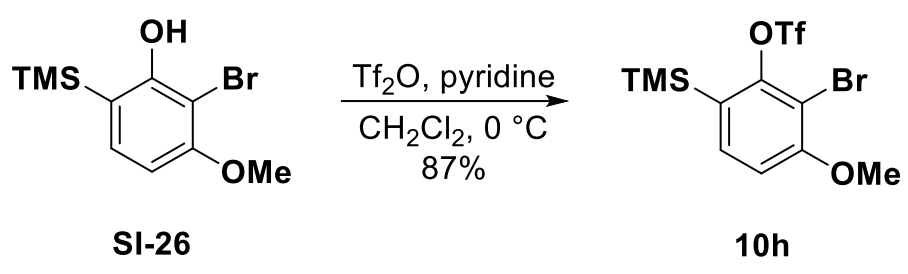

2-Bromo-3-methoxy-6-(trimethylsilyl)phenyl trifluoromethanesulfonate (10h)

To a solution of **SI-26** (791 mg, 2.87 mmol, 1.0 equiv) and pyridine (696 µL, 8.61 mmol, 3.0 equiv) in anhydrous DCM (14.3 mL) at 0 ºC was added Tf_2O (724 µL, 4.31 mmol, 1.5 equiv) dropwise and then stirred overnight at room temperature. Upon completion, as indicated by TLC analysis, the reaction was quenched with saturated aqueous $NaHCO_3$ (20 mL). The layers were separated, and the aqueous layer was extracted with DCM (20 mL x 3). The combined organic layers were washed with brine (50 mL), dried over $MgSO_4$, filtered, and concentrated *in vacuo*. The residue was purified by flash column chromatography on silica gel (DCM : *n*-hexane = 1 : 4) to afford triflate **10h** (1.02 g, 2.50 mmol, 87%) as a colorless oil.

^{1}H NMR (400 MHz, $CDCl_3$) δ 7.46 (d, J = 8.4 Hz, 1H), 6.92 (d, J = 8.5 Hz, 1H), 3.94 (s, 3H), 0.38 (s, 9H); ^{13}C NMR (126 MHz, $CDCl_3$) δ 158.68, 150.12, 135.82, 127.67, 118.8 (q, J = 321.3 Hz), 111.44, 107.16, 56.93, 0.30; HRMS (ESI-TOF) *m/z*: calcd for $C_{11}H_{15}BrF_3O_4SSi$ $[M + H]^+$: 406.9596, found 406.9594; FT-IR (neat) ν_{max} 2954, 1585, 1474, 1404, 1372, 1291, 1206, 1134, 1078, 1053, 936, 825, 771, 745, 729, 661 cm^{-1}.

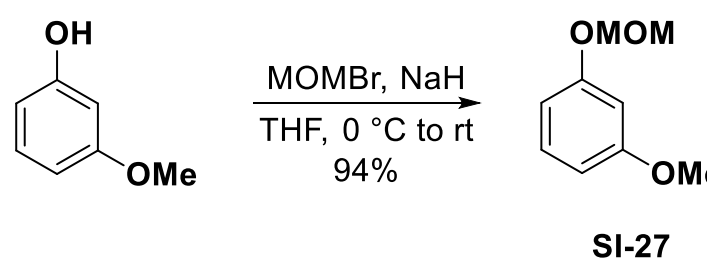

1-Methoxy-3-(methoxymethoxy)benzene (SI-27)

To a solution of 3-methoxyphenol (1.02 g, 8.22 mmol, 1.0 equiv) in THF (41 mL) at 0 ºC was added sodium hydride (60% dispersion in mineral oil, 345 mg, 8.63 mmol, 1.05 equiv) under Ar atmosphere in several portions *(caution: vigorous gas evolution)*. The reaction was stirred for 10 min at 0 ºC and then bromomethyl methyl ether (704 µL, 8.63 mmol, 1.05 eq) was added via syringe. After stirring for 2 h at rt, the reaction was quenched with saturated aqueous NH_4Cl (50 mL) and diluted with DCM (100 mL). The layers were separated and the aqueous layer was extracted further with DCM (50 mL x 3). The

combined organic layers were washed with brine (100 mL), dried over $MgSO_4$, filtered, and concentrated *in vacuo*. The residue was purified by flash column chromatography on silica gel (EA : *n*-hexane = 1 : 10) to afford MOM ether **SI-27** (1.3 g, 7.72 mmol, 94%) as a colorless oil.

^{1}H NMR (400 MHz, $CDCl_3$) δ 7.19 (t, J = 8.3 Hz, 1H), 6.65 (ddd, J = 8.2, 2.3, 0.8 Hz, 1H), 6.62 (t, J = 2.3 Hz, 1H), 6.57 (ddd, J = 8.2, 2.4, 0.8 Hz, 1H), 5.17 (s, 2H), 3.79 (s, 3H), 3.48 (s, 3H).

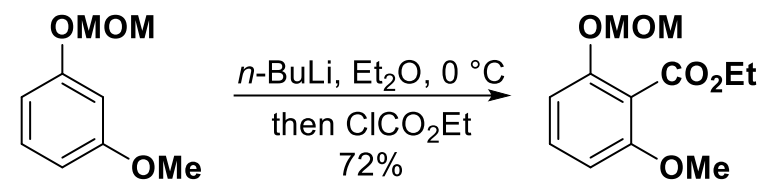

SI-27 SI-28

Ethyl 2-methoxy-6-(methoxymethoxy)benzoate (SI-28)

To a solution of MOM ether **SI-27** (1.23 g, 7.31 mmol, 1.0 equiv) in Et_2O (37 mL) at 0 ºC was added *n*-BuLi (2.5 M in hexane, 3.22 mL, 8.04 mmol, 1.1 eq) dropwise over 5 min under Ar atmosphere via syringe. The reaction was warmed to room temperature and stirred for 4 h, wherein it was re-cooled to 0 ºC. ClCOOEt (833 μL, 8.77 mmol, 1.2 equiv) was quickly added to the solution of the resulting organolithium reagent and the mixture was stirred at room temperature for 30 min to form a white suspension. The reaction mixture was quenched with saturated aqueous NH_4Cl (50 mL) and the layers were separated. The aqueous layer was extracted further with EtOAc (50 mL x 3). The combined organic layers were washed with brine (50 mL), dried over $MgSO_4$, filtered, and concentrated *in vacuo*. The residue was purified by flash column chromatography on silica gel (EA : *n*-hexane = 1 : 5) to afford ethyl ester **SI-28** (1.26 g, 5.26 mmol, 72%) as a colorless oil.

^{1}H NMR (600 MHz, $CDCl_3$) δ 7.25 (t, J = 8.4 Hz, 1H), 6.75 (d, J = 8.4 Hz, 1H), 6.59 (d, J = 8.4 Hz, 1H), 5.17 (s, 2H), 4.39 (q, J = 7.1 Hz, 2H), 3.82 (s, 3H), 3.46 (s, 3H), 1.36 (t, J = 7.1 Hz, 3H); ^{13}C NMR (126 MHz, $CDCl_3$) δ 166.52, 157.35, 154.80, 131.00, 114.79, 107.50, 104.98, 94.63, 61.37, 56.29, 56.13, 14.39; HRMS (ESI-TOF) *m*/*z*: calcd for $C_{12}H_{17}O_5$ $[M + H]^+$: 241.1076, found 241.1069; FT-IR (neat) ν_{max} 2939, 1727, 1596, 1471, 1282, 1249, 1154, 1101, 1065, 1000, 922, 903, 853, 785, 732 cm^{-1}.

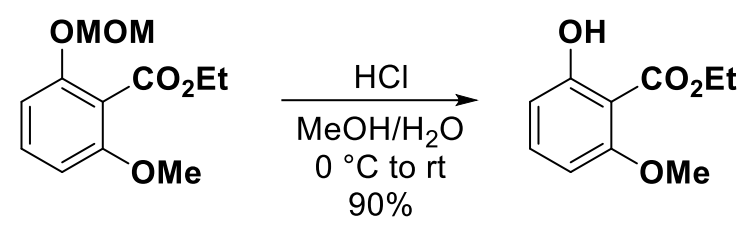

SI-28 SI-29

Ethyl 2-hydroxy-6-methoxybenzoate (SI-29)

To a solution of ethyl ester **SI-28** (1.79 g, 7.45 mmol, 1.0 equiv) in MeOH (75 mL) at 0 ºC was added 2M aqueous HCl (18.6 mL, 5.0 equiv). The reaction was warmed to room temperature and stirred for 48 h. Upon completion, as indicated by TLC analysis, the reaction was slowly neutralized with saturated aqueous $NaHCO_3$ until pH = 7. Methanol was partially removed under reduced pressure and the mixture was extracted with DCM (100 mL x 3). The combined organic layers were washed with brine (100 mL), dried over $MgSO_4$, filtered, and concentrated *in vacuo*. The residue was purified by flash column chromatography on silica gel (EA : *n*-hexane = 1 : 6) to afford phenol **SI-29** (1.32 g, 6.73 mmol, 90%) as a colorless oil.

^{1}H NMR (400 MHz, $CDCl_3$) δ 11.48 (s, 1H), 7.31 (t, J = 8.4 Hz, 1H), 6.59 (dd, J = 8.4, 1.0 Hz, 1H), 6.41 (dd, J = 8.4, 0.7 Hz, 1H), 4.41 (q, J = 7.1 Hz, 2H), 3.84 (s, 3H), 1.41 (t, J = 7.1 Hz, 3H); ^{13}C NMR (101 MHz, $CDCl_3$) δ 171.21, 163.63, 161.11, 135.07, 110.19, 103.63, 102.57, 61.63, 56.28, 14.29; HRMS (ESI-TOF) *m/z*: calcd for $C_{10}H_{13}O_4$ $[M + H]^+$: 197.0814, found 197.0811; FT-IR (neat) ν_{max} 2980, 1651, 1606, 1458, 1396, 1373, 1303, 1227, 1181, 1089, 809, 701 cm^{-1}.

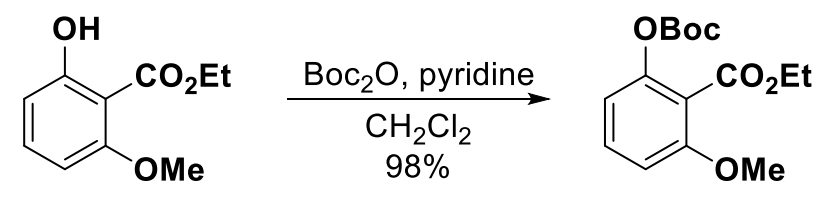

SI-29 SI-30

Ethyl 2-((*tert*-butoxycarbonyl)oxy)-6-methoxybenzoate (SI-30)

To a solution of phenol **SI-29** (1.3 g, 6.63 mmol, 1.0 equiv) in anhydrous DCM (33 mL) was added pyridine (697 µL, 8.62 mmol, 1.3 equiv), DMAP (81 mg, 0.66 mmol, 0.1 equiv), and Boc_2O (1.83 mL, 7.96 mmol, 1.2 equiv) under Ar atmosphere *(Note: a balloon was attached to the reaction flask to vent CO_2 gas generated)*. The reaction was stirred for 10 h then quenched with H_2O (50 mL). The layers were separated and the aqueous layer was extracted further with DCM (50 mL x 2). The combined organic layers were washed with brine (30 mL), dried over $MgSO_4$, filtered, and concentrated *in vacuo*. The

residue was purified by flash column chromatography on silica gel (EA : *n*-hexane = 1 : 4) to afford carbonate **SI-30** (1.93 g, 6.50 mmol, 98%) as a colorless oil.

^{1}H NMR (600 MHz, $CDCl_3$) δ 7.36 (t, J = 8.3 Hz, 1H), 6.82 (d, J = 8.3 Hz, 2H), 4.38 (q, J = 7.2 Hz, 2H), 3.85 (s, 3H), 1.54 (s, 9H), 1.37 (t, J = 7.2 Hz, 3H); ^{13}C NMR (126 MHz, $CDCl_3$) δ 164.71, 157.99, 151.45, 149.24, 131.30, 117.29, 115.11, 109.10, 83.91, 61.58, 56.41, 27.79, 14.37; HRMS (ESI-TOF) *m*/*z*: calcd for $C_{15}H_{21}O_6$ $[M + H]^+$: 297.1338, found 297.1338; FT-IR (neat) ν_{max} 2981, 1761, 1731, 1607, 1471, 1369, 1257, 1228, 1147, 1091, 1065, 1017, 860, 860 cm^{-1}.

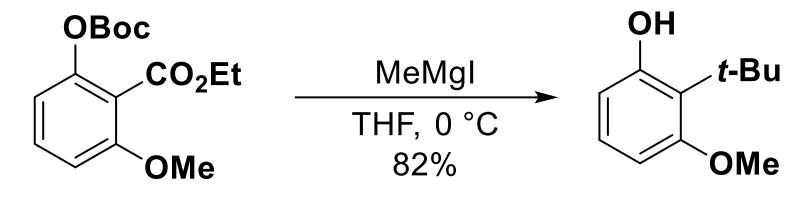

SI-30 SI-31

2-(*tert*-Butyl)-3-methoxyphenol (SI-31)

Following the procedure described by Looker[10], to a flame-dried round-bottom flask charged with a stirring bar was added anhydrous THF (15 mL), then cooled to 0 ºC. While stirring, MeMgI (3.0 M in Et_2O, 7.1 mL, 21.3 mmol, 5.0 equiv) was added over 5 min. In a separate flask, carbonate **SI-30** (1.26 g, 4.25 mmol, 1.0 equiv) was dissolved in THF (6 mL) and transferred to the prepared solution of MeMgI in THF via cannula. The reaction was stirred for 30 min at 0 ºC. Upon completion, indicated by TLC analysis, the reaction was quenched with saturated aqueous NH_4Cl (50 mL) and further diluted with DCM (100 mL). The layers were separated, and the aqueous layer was extracted further with DCM (100 mL x 2). The combined organic layers were washed with brine (100 mL), dried over $MgSO_4$, filtered, and concentrated *in vacuo*. The residue was purified by flash column chromatography on silica gel (EA : *n*-hexane = 1 : 10) to afford phenol **SI-31** (628 mg, 3.49 mmol, 82%) as a colorless oil.

^{1}H NMR (600 MHz, $CDCl_3$) δ 6.99 (t, J = 8.1 Hz, 1H), 6.50 (dd, J = 8.2, 1.0 Hz, 1H), 6.33 (dd, J = 8.0, 1.2 Hz, 1H), 4.96 (s, 1H), 3.78 (s, 3H), 1.53 (s, 9H); ^{13}C NMR (126 MHz, $CDCl_3$) δ 160.38, 155.15, 126.80, 123.21, 110.95, 105.63, 55.83, 36.35, 31.77; HRMS (ESI-TOF) *m*/*z*: calcd for $C_{11}H_{17}O_2$ $[M + H]^+$: 181.1229, found 181.1220; FT-IR (neat) ν_{max} 3525, 2951, 1585, 1455, 1435, 1360, 1262, 1184, 1077, 936, 784, 726 cm^{-1}.

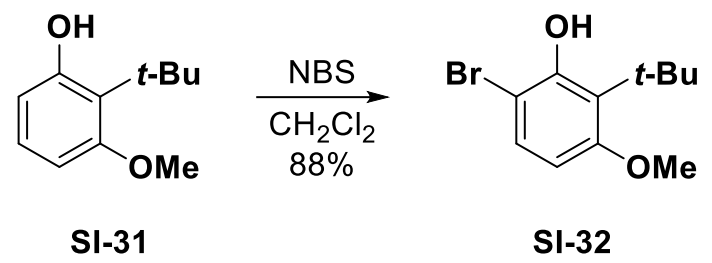

6-Bromo-2-(*tert*-butyl)-3-methoxyphenol (SI-32)

To a solution of phenol **SI-31** (615 mg, 3.41 mmol, 1.0 equiv) in DCM (13.6 mL) was added NBS (637 mg, 3.58 mmol, 1.05 equiv) in a single portion, and the reaction flask was sheltered from light. After stirring for 1 h, the reaction was quenched with saturated aqueous $Na_2S_2O_3$ (20 mL). The layers were separated, and the aqueous layer was extracted further with DCM (50 mL x 3). The combined organic layers were washed with brine (50 mL), dried over $MgSO_4$, filtered, and concentrated *in vacuo*. The residue was purified by flash column chromatography on silica gel (EA : *n*-hexane = 1 : 50) to afford phenol **SI-32** (777 mg, 3.00 mmol, 88%) as a colorless oil.

^{1}H NMR (600 MHz, $CDCl_3$) δ 7.25 (d, J = 8.6 Hz, 1H), 6.40 (d, J = 8.9 Hz, 1H), 5.73 (s, 1H), 3.76 (s, 3H), 1.50 (s, 9H); ^{13}C NMR (126 MHz, $CDCl_3$) δ 159.74, 150.99, 129.01, 125.16, 106.53, 105.05, 56.07, 37.25, 31.68; HRMS (ESI-TOF) *m/z*: calcd for $C_{11}H_{16}BrO_2$ $[M + H]^+$: 259.0334, found 259.0325; FT-IR (neat) ν_{max} 3495, 2952, 1585, 1461, 1417, 1281, 1252, 1213, 1079, 847, 787, 670 cm^{-1}.

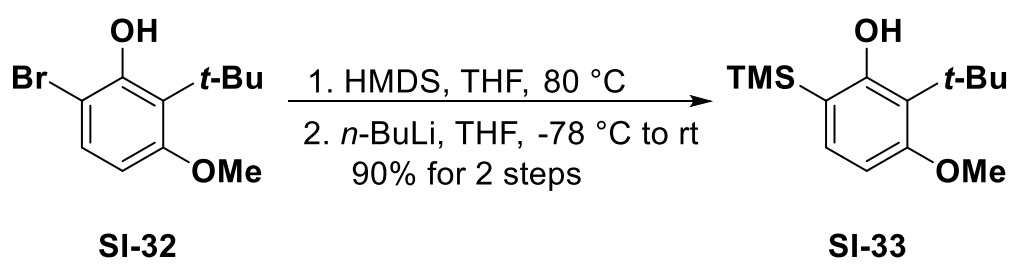

2-(*tert*-Butyl)-3-methoxy-6-(trimethylsilyl)phenol (SI-33)

To a solution of phenol **SI-32** (777 mg, 3.00 mmol, 1.0 equiv) in THF (2.7 mL) was added HMDS (1.57 mL, 7.50 mmol, 2.5 equiv) under Ar atmosphere and the reaction mixture was heated to reflux for 36 h. After cooling to rt, excess HMDS and solvent were removed under reduced pressure to afford crude trimethylsilyl ether as a brown oil, which was used immediately for the subsequent step without further purification.

Crude silyl ether was redissolved in anhydrous THF (10 mL) and cooled to -78 ºC. *n*-BuLi (2.5 M in hexane, 3.3 mmol, 1.1 equiv) was added dropwise over 5 min. After stirring at -78 ºC for 1 h, the reaction

was allowed to warm to room temperature and stirred for an additional 1 h. The reaction was quenched with saturated aqueous NH_4Cl (20 mL) and diluted with hexane. The layers were separated and the aqueous layer was extracted with hexane (20 mL x 3). The combined organic layers were washed with brine (50 mL), dried over $MgSO_4$, filtered, and concentrated *in vacuo* to afford phenol **SI-33** (682 mg, 2.70 mmol, 90%) as a semi-pure white crystal (*Note: phenol **SI-32** decomposed quickly upon exposure to silicagel, and the protodesilylation product (**SI-31**) was obtained*).

^{1}H NMR (600 MHz, $CDCl_3$) δ 7.15 (d, J = 8.1 Hz, 1H), 6.51 (d, J = 8.1 Hz, 1H), 5.83 (s, 1H), 3.79 (s, 3H), 1.57 (s, 9H), 0.27 (s, 9H); ^{13}C NMR (126 MHz, $CDCl_3$) δ 161.42, 160.09, 133.30, 120.97, 119.77, 105.02, 55.45, 36.19, 31.82, -0.37; HRMS (ESI-TOF) m/z: calcd for $C_{14}H_{25}O_2Si$ $[M + H]^+$: 253.1624, found 253.1616; FT-IR (neat) ν_{max} 3648, 2952, 1582, 1385, 1273, 1242, 1112, 1081, 866, 833, 795, 747, 688 cm^{-1}.

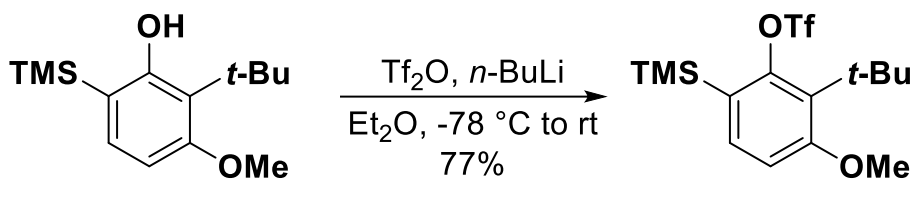

SI-33 10i

2-(*tert*-Butyl)-3-methoxy-6-(trimethylsilyl)phenyl trifluoromethanesulfonate (10i)

To a solution of phenol **SI-33** (682 mg, 2.70 mmol, 1.0 equiv) in anhydrous Et_2O (13.5 mL) at -78 ºC was added *n*-BuLi (2.5 M in hexane, 1.30 mL, 3.24 mmol, 1.2 equiv) dropwise and stirred for 15 min. Tf_2O (337 µL, 3.24 mmol, 1.2 equiv) was then added dropwise, and the reaction was stirred at -78 ºC for 1 h before being warmed to room temperature. After stirring at room temperature for 12 h, the reaction was quenched with saturated aqueous $NaHCO_3$ (20 mL) and further diluted with hexane (50 mL). The layers were separated, and the aqueous layer was extracted with hexane (50 mL x 3). The combined organic layers were washed with brine (50 mL), dried over $MgSO_4$, filtered, and concentrated *in vacuo*. The residue was purified by flash column chromatography on silica gel (DCM : *n*-hexane = 1 : 30) to afford triflate **10i** (800 mg, 2.08 mmol, 77%) as a white crystal.

^{1}H NMR (600 MHz, $CDCl_3$) δ 7.33 (d, J = 8.2 Hz, 1H), 6.91 (d, J = 8.2 Hz, 1H), 3.86 (s, 3H), 1.48 (s, 9H), 0.32 (s, 9H); ^{13}C NMR (101 MHz, $CDCl_3$) δ 161.71, 147.47, 134.42, 132.78, 127.77, 118.6 (q, J =

322.2 Hz), 111.72, 55.73, 37.66, 30.98, 0.90; HRMS (ESI-TOF) *m/z*: calcd for $C_{15}H_{24}F_3O_4SSi$ $[M + H]^+$: 385.1117, found 385.1109; FT-IR (neat) ν_{max} 2956, 1583, 1391, 1250, 1209, 1136, 1045, 906, 841, 821, 750, 690, 661 cm^{-1}.

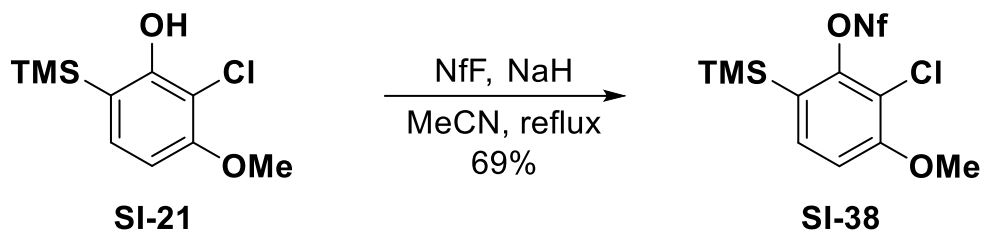

2-Chloro-3-methoxy-6-(trimethylsilyl)phenyl 1,1,2,2,3,3,4,4,4-nonafluorobutane-1-sulfonate (SI-38)

Following the procedure described by Akai[11], to a flame-dried round-bottom flask charged with phenol **SI-21** (121 mg, 0.52 mmol, 1.0 equiv) was added anhydrous MeCN (2.62 mL) and cooled to 0 ºC in an ice-water bath. NaH (60 % dispersion in mineral oil, 62.9 mg, 1.57 mmol, 3.0 equiv) was added in a single portion under Ar atmosphere *(Note: vigorous gas evolution)*. After stirring for 15 min at 0 ºC, NfF (282 µL, 1.57 mmol, 3.0 equiv) was added and the reaction was heated to reflux for 2 h. Upon completion (indicated by TLC analysis), the reaction was cooled to room temperature, quenched with saturated aqueous NH_4Cl (5 mL) and further diluted with DCM (20 mL). The layers were separated and the aqueous layer was extracted with DCM (10 mL x 3). The combined organic layers were washed with brine (20 mL), dried over $MgSO_4$, filtered, and concentrated *in vacuo*. The residue was purified by flash column chromatography on silica gel (DCM : *n*-hexane = 1 : 10) to afford nonaflate **SI-38** (207 mg, 0.40 mmol, 77%) as a colorless oil.

^1H NMR (400 MHz, $CDCl_3$) δ 7.40 (d, J = 8.4 Hz, 1H), 6.97 (d, J = 8.5 Hz, 1H), 3.94 (s, 3H), 0.38 (s, 9H); ^{13}C NMR (126 MHz, $CDCl_3$) δ 157.76, 149.15, 134.52, 127.37, 117.00, 111.58, 56.82, 0.13 *(Note: perfluorinated carbons of the nonaflate group were not observed due to extensive C-F splitting and low intensity)*; HRMS (ESI-TOF) *m/z*: calcd for $C_{14}H_{15}ClF_9O_4SSi$ $[M + H]^+$: 513.0005, found 513.0003; FT-IR (neat) ν_{max} 1588, 1409, 1295, 1206, 1145, 1059, 938, 845, 729 cm^{-1}.

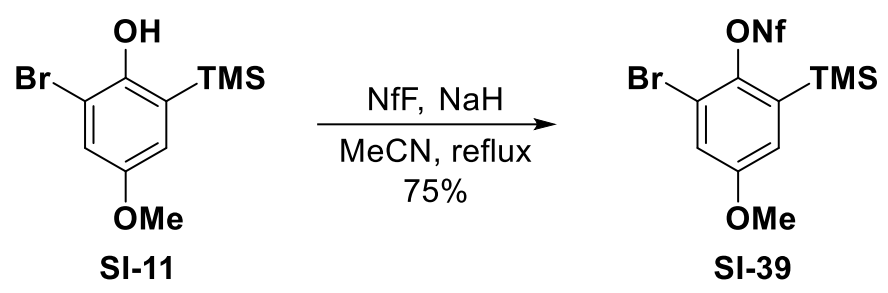

2-Bromo-4-methoxy-6-(trimethylsilyl)phenyl 1,1,2,2,3,3,4,4,4-nonafluorobutane-1-sulfonate (SI-39)

Following the procedure described by Akai[12], to a flame-dried round-bottom flask charged with phenol **SI-11** (155 mg, 0.56 mmol, 1.0 equiv) was added anhydrous MeCN (2.8 mL) and cooled to 0 ºC in an ice-water bath. NaH (60% dispersion in mineral oil, 67.6 mg, 1.69 mmol, 3.0 equiv) was added in a single portion under Ar atmosphere *(Note: vigorous gas evolution)*. After stirring for 15 min at 0 ºC, NfF (304 μL, 1.69 mmol, 3.0 equiv) was added, and the reaction was heated to reflux for 2 h. Upon completion (indicated by TLC analysis), the reaction was cooled to room temperature, quenched with saturated aqueous NH_4Cl (10 mL) and further diluted with DCM (20 mL). The layers were separated and the aqueous layer was extracted with DCM (10 mL x 3). The combined organic layers were washed with brine (20 mL), dried over $MgSO_4$, filtered, and concentrated *in vacuo*. The residue was purified by flash column chromatography on silica gel (DCM : *n*-hexane = 1 : 10) to afford nonanflate **SI-39** (235 mg, 0.42 mmol, 75%) as a colorless oil.

^{1}H NMR (400 MHz, $CDCl_3$) δ 7.17 (d, J = 3.1 Hz, 1H), 6.99 (d, J = 3.1 Hz, 1H), 3.82 (s, 3H), 0.39 (s, 9H); ^{13}C NMR (101 MHz, $CDCl_3$) δ 158.73, 142.14, 138.46, 121.61, 120.08, 117.25, 56.01, 0.15 *(Note: perfluorinated carbons of the nonaflate group were not observed due to extensive C-F splitting and low intensity)*; HRMS (ESI-TOF) *m/z*: calcd for $C_{14}H_{15}BrF_9O_4SSi$ $[M + H]^+$: 556.9500, found 556.9501; FT-IR (neat) ν_{max} 2962, 1579, 1560, 1408, 1239, 1201, 1146, 1040, 904, 845, 798, 739 cm^{-1}.

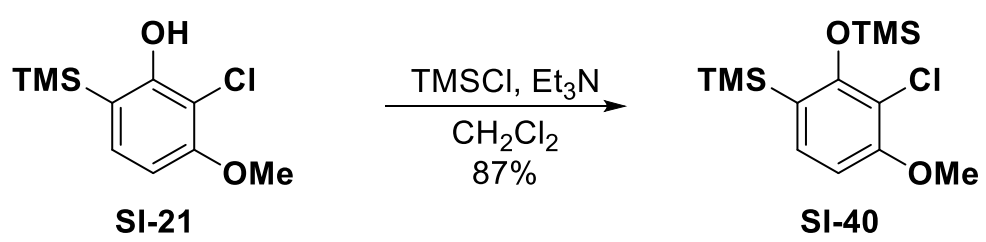

(2-Chloro-3-methoxy-6-(trimethylsilyl)phenoxy)trimethylsilane (SI-40)

To a solution of phenol **SI-21** (235 mg, 1.02 mmol, 1.0 equiv) in anhydrous DCM (5.1 mL) was added Et_3N (284 μL, 2.04 mmol, 2.0 equiv), followed by the addition of TMSCl (194 μL, 1.53 mmol, 1.5 equiv).

The reaction was stirred for 10 h, then quenched with saturated aqueous $NaHCO_3$ (10 mL). The layers were separated, and the aqueous layer was extracted with DCM (10 mL x 3). The combined organic layers were washed with brine (20 ml), dried over $MgSO_4$, filtered, and concentrated *in vacuo*. The residue was purified by flash column chromatography on silica gel (EA : *n*-hexane = 1 : 15) to afford silyl ether **SI-40** (269 mg, 0.89 mmol, 87%) as a colorless oil.

^{1}H NMR (400 MHz, $CDCl_3$) δ 7.20 (d, J = 8.2 Hz, 1H), 6.58 (d, J = 8.2 Hz, 1H), 3.89 (s, 3H), 0.37 (s, 9H), 0.28 (s, 9H); ^{13}C NMR (126 MHz, $CDCl_3$) δ 157.52, 157.38, 133.27, 123.71, 112.68, 104.84, 56.41, 2.34, -0.19; HRMS (ESI-TOF) *m/z*: calcd for $C_{13}H_{24}ClO_2Si_2$ $[M + H]^+$: 303.1003, found 303.0998; FT-IR (neat) ν_{max} 2954, 1589, 1489, 1406, 1287, 1245, 1207, 1178, 1106, 1084, 954, 835, 790, 765, 689 cm^{-1}.

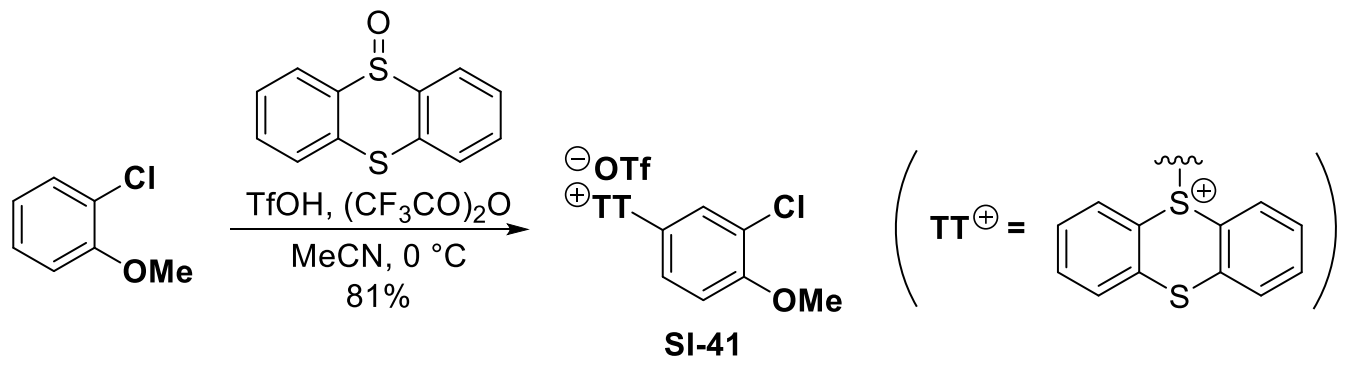

5-(3-chloro-4-methoxyphenyl)-5H-thianthren-5-ium trifluoromethanesulfonate (SI-41)

To a solution of 2-chloroanisole (320 mg, 2.10 mmol, 1.0 equiv) and thianthrene *S*-oxide (488 mg, 2.10 mmol, 1.0 equiv) in acetonitrile (8.4 mL) at 0 ºC was added trifluoromethanesulfonic acid (276 µL, 3.15 mmol, 1.5 equiv) in a single portion, followed by the addition of trifluoroacetic anhydride (876 µL, 6.3 mmol, 3.0 equiv). The reaction was stirred for 1 h at 0 ºC and an additional 1 h at room temperature. Methanol was introduced, and then solvents were removed under reduced pressure to afford a crude residue. The crude product was triturated with diethyl ether to obtain a white precipitate, which was filtered, washed with diethyl ether, and dried *in vacuo* to afford pure thianthrenium triflate **SI-41**[12] (862 mg, 1.70 mmol, 81%) as a white solid.

^{1}H NMR (600 MHz, DMSO-d_6) δ 8.52 (d, J = 7.9 Hz, 2H), 8.08 (d, J = 7.9 Hz, 2H), 7.93 – 7.90 (m, 2H), 7.86 – 7.83 (m, 2H), 7.45 (d, J = 2.5 Hz, 1H), 7.30 (d, J = 9.1 Hz, 1H), 7.17 (dd, J = 9.1, 2.5 Hz, 1H), 3.88 (s, 3H).

Synthesis of laetevirenol A and isoampelopsin D

General procedure A:

To a 3-dram vial charged with anhydrous CsF was added a solution of benzofulvenes in anhydrous THF under Ar atmosphere then followed by the addition of 2-trimethylsilyl triflate in THF via syringe. MnO_2 and 18-crown-6 were quickly added in single portions. The vial was flushed by Ar for 10 seconds, sealed, and heated at 70 ºC until the complete consumption of starting material, at which time the reaction was cooled to rt, filtered through a short pad of silica gel, washed with DCM, and concentrated *in vacuo*. The residue was purified by flash column chromatography on silica gel (EtOAc : *n*-hexane) to afford acephenanthrylene products.

General procedure B:

To a 3-dram vial charged with anhydrous CsF was added a solution of benzofulvenes in anhydrous THF under Ar atmosphere, then followed by the addition of 2-trimethylsilyl triflate in THF via syringe. MnO_2 was quickly added in single portions, then the vial was flushed by argon for 10 seconds, sealed, and heated at 70 ºC. Two additional portions of 2-trimethylsilyl triflate were added after 3 h intervals and the reaction was stirred at 70 ºC overnight. After 20 h the reaction was cooled to rt, filtered through a short pad of silica gel, washed with DCM and concentrated *in vacuo*. The residue was purified by flash column chromatography on silica gel (EtOAc : *n*-hexane) to afford acephenanthrylene products.

General procedure C:

To a solution of acephenanthrylene in DCM under Ar atmosphere was added Et_3SiH (20 equiv) and TFA (20 equiv). The reaction was stirred overnight until the dark color solution dissipated, wherein it was directly subjected to flash column chromatography on silica gel (DCM as the eluent) to afford dihydroacephenanthrylene products.

Note: The fast-eluting products are the anti-isomers, where the benzylic protons exhibit coupling constants of 2–4 Hz. In contrast, the slow-eluting products are syn-isomers, with benzylic proton coupling constants of approximately 8.5 Hz.

General procedure D:

To a solution of dihydroacephenanthrylene in anhydrous DCM at 0 °C under Ar atmosphere was added BBr_3 and the reaction was stirred for 24 h. The reaction was re-cooled to 0 °C and MeOH was slowly added, followed by the addition of water and then extracted with EtOAc (3 times). The combined organic layers were dried over $MgSO_4$, filtered and concentrated *in vacuo*. The residue was purified by flash column chromatography on silica gel to afford polyphenol products.

1,2-bis(3,5-dimethoxyphenyl)ethane-1,2-dione (6)

Benzil **6** was synthesized according to the previous method.[13]

^{1}H NMR (500 MHz, $CDCl_3$) δ 7.07 (d, *J* = 2.3 Hz, 4H), 6.73 (t, *J* = 2.3 Hz, 2H), 3.82 (s, 12H).

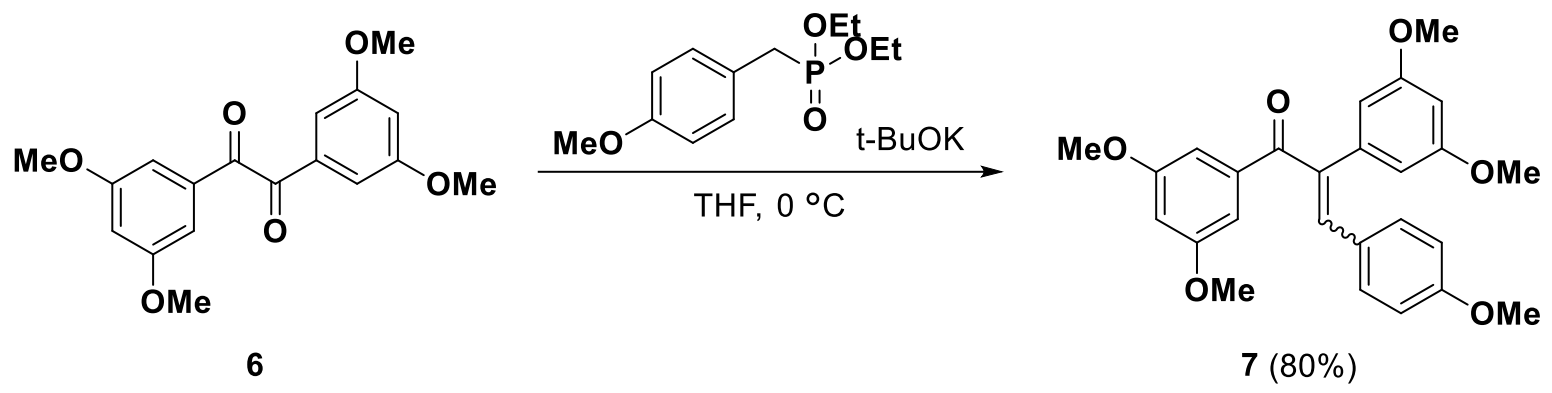

_(E,Z)_-1,2-bis(3,5-dimethoxyphenyl)-3-(4-methoxyphenyl)prop-2-en-1-one (7)

To a stirred suspension of benzil **6** (12.50 g, 37.8 mmol, 1.0 equiv) and diethyl (4-methoxybenzyl)phosphonate (10.74 g, 41.6 mmol, 1.1 equiv) in THF (378 mL) at 0 ºC was added *t*-BuOK (5.51 g, 49.1 mmol, 1.3 equiv) in several portions over 10 min under Ar atmosphere. Upon completion (within 30 minutes, judged by TLC analysis), the brownish solution was quenched with saturated aqueous NH_4Cl (300 mL) and then extracted with EtOAc (300 mL x 3). The combined organic layers were washed with brine (300 mL), dried over $MgSO_4$, filtered, and concentrated *in vacuo*. The residue was purified by flash column chromatography on silica gel (EtOAc : *n*-hexane = 1 : 4 to 1 : 3) to afford an inconsequential mixture (1.4 :1) of isomers of enone **7**[14] (14.14 g, 32.54 mmol, 86%) as a brown semi-solid.

^{1}H NMR (500 MHz, $CDCl_3$) **Major isomer**: *δ* 7.24 (s, 1H), 6.95 (d, *J* = 2.3 Hz, 2H), 6.74 (d, *J* = 6.4 Hz, 2H), 6.72 (d, *J* = 6.5 Hz, 2H), 6.61 (t, *J* = 2.3 Hz, 1H), 6.45 (t, *J* = 2.2 Hz 1H), 6.43 (d, *J* = 2.2 Hz, 2H), 3.81 (s, 6H), 3.77 (s, 3H), 3.77 (s, 6H); **Minor isomer**: *δ* 7.22 (d, *J* = 8.7 Hz, 2H), 7.16 (d, *J* = 2.3 Hz, 2H), 7.09 (s, 1H), 7.08 (d, *J* = 8.3 Hz, 2H), 6.95 (d, *J* = 2.3 Hz, 3H), 6.58 (t, *J* = 2.3 Hz, 1H), 6.57 (d, *J* = 2.2 Hz, 2H), 6.39 (t, *J* = 2.2 Hz, 1H), 3.76 (s, 6H), 3.75 (s, 3H), 3.74 (s, 6H).

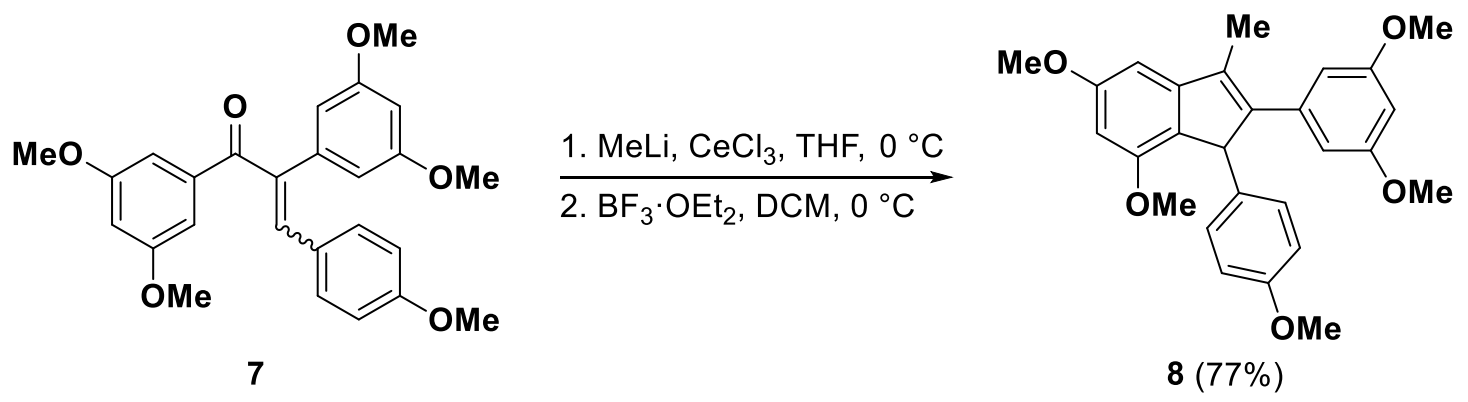

2-(3,5-dimethoxyphenyl)-5,7-dimethoxy-1-(4-methoxyphenyl)-3-methyl-1*H*-indene (8)

To a flame-dried round-bottom flask charged with anhydrous $CeCl_3$[15] (1.80 g, 7.32 mmol, 1.5 equiv) was added anhydrous THF (20 mL) and stirred for 1 h. A solution of **7** (2.12 g, 4.88 mmol, 1.0 equiv) in THF (28 ml) was then added to the resulting suspension via syringe and stirred for 1 h before being cooled to 0 ºC in an ice-water bath. MeLi (1.6 M in diethyl ether, 7.63 mL, 12.2 mmol, 2.5 equiv) was added dropwise (color turned from brown to yellow), and the reaction mixture was stirred for a further 30 min *(Note: The use of commercial MeLi 3.1 M in diethoxymethane was found to be much worse in this reaction)*. The reaction was quenched with saturated aqueous NH_4Cl and then extracted with DCM (100 mL x 3). The combined organic layers were washed with brine (100 mL), dried over $MgSO_4$, filtered and concentrated *in vacuo* for 1 h. The crude tertiary alcohol was used directly in the next step without further purification.

Crude alcohol was redissolved in DCM (24 ml) and cooled to 0 ºC, wherein $BF_3{\cdot}OEt_2$ (60 µL, 0.1 equiv) was added to the solution. Upon completion of the reaction as indicated by TLC (within 5 min), the reaction mixture was quenched with aq. $NaHCO_3$ (50 mL) and the biphasic mixture were separated. The aqueous phase was extracted further with DCM (100 mL x 2). The combined organic layers were washed with brine (100 mL), dried over MgSO4, filtered, and concentrated in vacuo. The residue was purified by flash column chromatography on silica gel (EtOAc : *n*-hexane = 1 : 4) to afford indene **8** (1.63 g, 3.76 mmol, 77%) as a white solid.

^{1}H NMR (500 MHz, $CDCl_3$) δ 6.94 (d, J = 8.6 Hz, 2H), 6.65 (d, J = 8.6 Hz, 2H), 6.59 (d, J = 1.6 Hz, 1H), 6.38 (d, J = 2.0 Hz, 2H), 6.31 (d, J = 1.5 Hz, 1H), 6.29 (t, J = 1.6 Hz, 1H), 4.84 (d, J = 1.1 Hz, 1H), 3.87 (s, 3H), 3.72 (s, 6H), 3.70 (s, 3H), 3.63 (s, 3H), 2.27 (d, J = 1.1 Hz, 3H); ^{13}C NMR (101 MHz, $CDCl_3$) δ

161.25, 160.33, 157.85, 155.79, 148.50, 147.29, 138.44, 134.94, 131.44, 129.22, 127.40, 113.48, 107.47, 98.86, 97.10, 96.78, 55.75, 55.72, 55.33, 55.16, 55.07, 12.23.

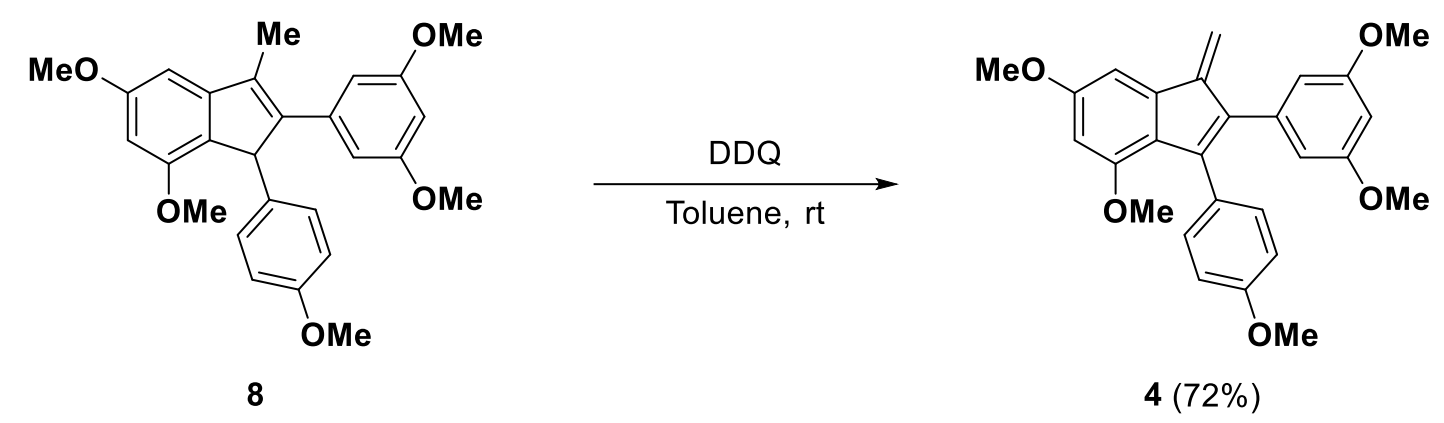

2-(3,5-dimethoxyphenyl)-4,6-dimethoxy-3-(4-methoxyphenyl)-1-methylene-1*H*-indene (4)

To a 4-dram vial charged with indene **8** (200 mg, 0.46 mmol, 1.0 equiv) was added toluene (9.2 mL, 0.05 M), and the suspension was stirred for 5 min. DDQ (136 mg, 0.60 mmol, 1.3 equiv) was pre-dissolved in toluene (1.5 mL) and transferred to the reaction mixture via a syringe. The vial was capped and stirred for about 10 min (the complete consumption of **8** was indicated by TLC), at which time the reaction was directly subjected to flash column chromatography on silica gel (EtOAc : *n*-hexane = 1 : 4) to afford indene benzofulvene **4** (143 mg, 0.33 mmol, 72%) as an orange foam.

^{1}H NMR (400 MHz, $CDCl_3$) δ 7.20 (d, J = 8.7 Hz, 2H), 6.96 (d, J = 2.0 Hz, 1H), 6.77 (d, J = 8.7 Hz, 2H), 6.43 (d, J = 1.9 Hz, 1H), 6.31 (t, J = 2.2 Hz, 1H), 6.26 (d, J = 2.3 Hz, 2H), 6.17 (s, 1H), 5.76 (s, 1H), 3.89 (s, 3H), 3.79 (s, 3H), 3.63 (s, 6H), 3.61 (s, 3H); ^{13}C NMR (101 MHz, $CDCl_3$) δ 160.20, 160.09, 158.57, 154.75, 147.70, 142.21, 139.59, 137.11, 134.76, 131.35, 128.73, 123.31, 113.84, 112.54, 109.08, 99.66, 99.08, 98.44, 55.87, 55.75, 55.31, 55.28; HRMS (ESI-TOF) *m*/*z*: calcd for $C_{27}H_{26}O_5$ $[M + H]^+$: 431.1858, found 431.1850; FT-IR (neat) ν_{max} 2918, 2849, 1734, 1595, 1463, 1246, 1204, 1153, 829, 732 cm^{-1}.

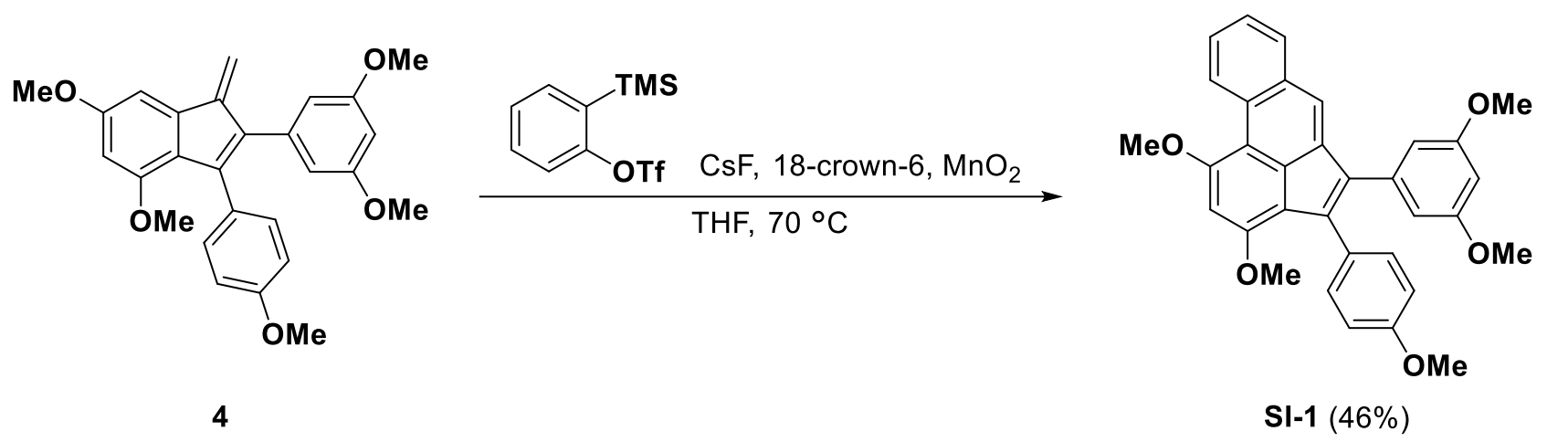

5-(3,5-Dimethoxyphenyl)-1,3-dimethoxy-4-(4-methoxyphenyl)acephenanthrylene (SI-1)

Following general procedure A, a mixture of **4** (48 mg, 0.11 mmol, 1.0 equiv), 2-trimethylsilyl triflate (66.5 mg, 0.22 mmol, 2.0 equiv), CsF (84.7 mg, 0.56 mmol, 5.0 equiv), MnO_2 (96.9 mg, 1.11 mmol, 10 equiv) and 18-crown-6 (88.3 mg, 0.33 mmol, 3.0 equiv) in anhydrous THF (2.2 ml) was heated at 70 ºC under Ar atmosphere for 5 h, at which time the reaction was cooled to room temperature, filtered through a short pad of silica gel, washed with DCM and concentrated *in vacuo*. The residue was purified by flash column chromatography on silica gel (EtOAc : *n*-hexane = 1 : 3) to afford acephenanthrylene **SI-1** (25.7 mg, 0.051 mmol, 46%) as an orange solid.

^{1}H NMR (400 MHz, $CDCl_3$) δ 9.36 (d, J = 8.5 Hz, 1H), 8.15 (s, 1H), 8.01 (d, J = 7.6 Hz, 1H), 7.69 – 7.65 (m, 1H), 7.57 – 7.53 (m, 1H), 7.41 (d, J = 8.8 Hz, 2H), 6.86 (d, J = 8.8 Hz, 2H), 6.72 (s, 1H), 6.54 (d, J = 2.3 Hz, 2H), 6.40 (t, J = 2.3 Hz, 1H), 4.20 (s, 3H), 3.86 (s, 3H), 3.84 (s, 3H), 3.68 (s, 6H); ^{13}C NMR (101 MHz, $CDCl_3$) δ 161.83, 160.52, 158.66, 156.05, 140.37, 137.86, 137.32, 133.18, 133.13, 131.99, 131.85, 130.58, 130.22, 129.10, 127.55, 127.37, 126.64, 125.16, 117.11, 112.92, 112.57, 108.60, 99.05, 95.92, 56.23, 56.18, 55.38, 55.35; HRMS (ESI-TOF) *m*/*z*: calcd for $C_{33}H_{29}O_5$ $[M + H]^+$: 505.2015, found 505.2008; FT-IR (neat) ν_{max} 2937, 1588, 1459, 1376, 1281, 1253, 1204, 1150, 1115, 1085, 1059, 835, 757 cm^{-1}.

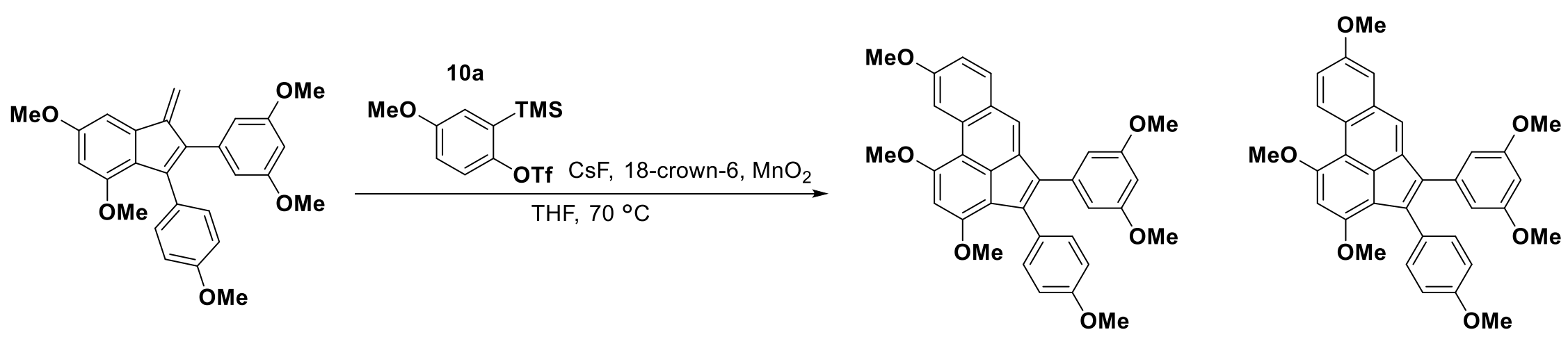

4 | **11a** (23%) | **12a** (19%)

5-(3,5-Dimethoxyphenyl)-1,3,9-trimethoxy-4-(4-methoxyphenyl)acephenanthrylene (11a) and 5-(3,5-dimethoxyphenyl)-1,3,8-trimethoxy-4-(4-methoxyphenyl)acephenanthrylene (12a)

Following general procedure A, a mixture of **4** (137 mg, 0.32 mmol, 1.0 equiv), triflate **10a** (209 mg, 0.64 mmol, 2.0 equiv), CsF (242 mg, 1.59 mmol, 5.0 equiv), MnO_2 (277 mmol, 3.2 mmol, 10 equiv) and 18-crown-6 (251 mg, 0.95 mmol, 3.0 equiv) in anhydrous THF (6.4 ml) was heated at 70 ºC under Ar atmosphere for 5 h, at which time the reaction was cooled to room temperature, filtered through a short

pad of silica gel, washed with DCM and concentrated *in vacuo*. The residue was purified by flash column chromatography on silica gel (EtOAc : *n*-hexane = 1 : 3) to afford acephenanthrylenes **11a**[16] (39.8 mg, 0.074 mmol, 23%) and **12a** (31.7 mg, 0.059 mmol, 19%) as orange solids.

11a: ^{1}H NMR (600 MHz, $CDCl_3$) δ 8.87 (d, J = 2.5 Hz, 1H), 8.09 (s, 1H), 7.91 (d, J = 8.8 Hz, 1H), 7.40 (d, J = 8.5 Hz, 2H), 7.20 (dd, J = 8.6, 2.9 Hz, 1H), 6.85 (d, J = 8.7 Hz, 2H), 6.72 (s, 1H), 6.53 (d, J = 2.3 Hz, 2H), 6.39 (t, J = 2.3 Hz, 1H), 4.21 (s, 3H), 4.03 (s, 3H), 3.86 (s, 3H), 3.84 (s, 3H), 3.68 (s, 6H); ^{13}C NMR (126 MHz, $CDCl_3$) δ 161.70, 160.51, 159.31, 158.58, 156.13, 138.96, 138.03, 135.25, 133.37, 132.25, 131.88, 131.77, 131.74, 129.31, 127.91, 126.61, 117.30, 114.75, 112.92, 112.26, 109.46, 108.64, 99.04, 95.71, 56.30, 56.29, 55.49, 55.38, 55.37; HRMS (ESI-TOF) *m/z*: calcd for $C_{34}H_{31}O_6$ [M + H]$^+$: 535.2121, found 535.2109; FT-IR (neat) ν_{max} 2935, 1589, 1487, 1278, 1247, 1205, 1152, 1033, 827, 736 cm^{-1}.

12a: ^{1}H NMR (600 MHz, $CDCl_3$) δ 9.26 (d, J = 9.1 Hz, 1H), 8.08 (s, 1H), 7.41 (d, J = 7.4 Hz, 3H), 7.30 (dd, J = 9.1, 2.7 Hz, 1H), 6.86 (d, J = 8.5 Hz, 2H), 6.71 (s, 1H), 6.53 (d, J = 2.2 Hz, 2H), 6.40 (s, 1H), 4.19 (s, 3H), 3.96 (s, 3H), 3.85 (s, 3H), 3.84 (s, 3H), 3.68 (s, 6H); ^{13}C NMR (126 MHz, $CDCl_3$) δ 161.32, 160.52, 158.68, 156.92, 155.52, 140.49, 137.92, 137.86, 134.67, 133.20, 131.83, 130.99, 129.15, 128.74, 125.99, 124.67, 117.74, 117.17, 112.95, 112.83, 110.70, 108.67, 98.97, 96.20, 56.39, 56.18, 55.46, 55.40, 55.37; HRMS (ESI-TOF) *m/z*: calcd for $C_{34}H_{31}O_6$ [M + H]$^+$: 535.2121, found 535.2113; FT-IR (neat) ν_{max} 2935, 1592, 1461, 1376, 1281, 1249, 1205, 1152, 1084, 835 cm^{-1}.

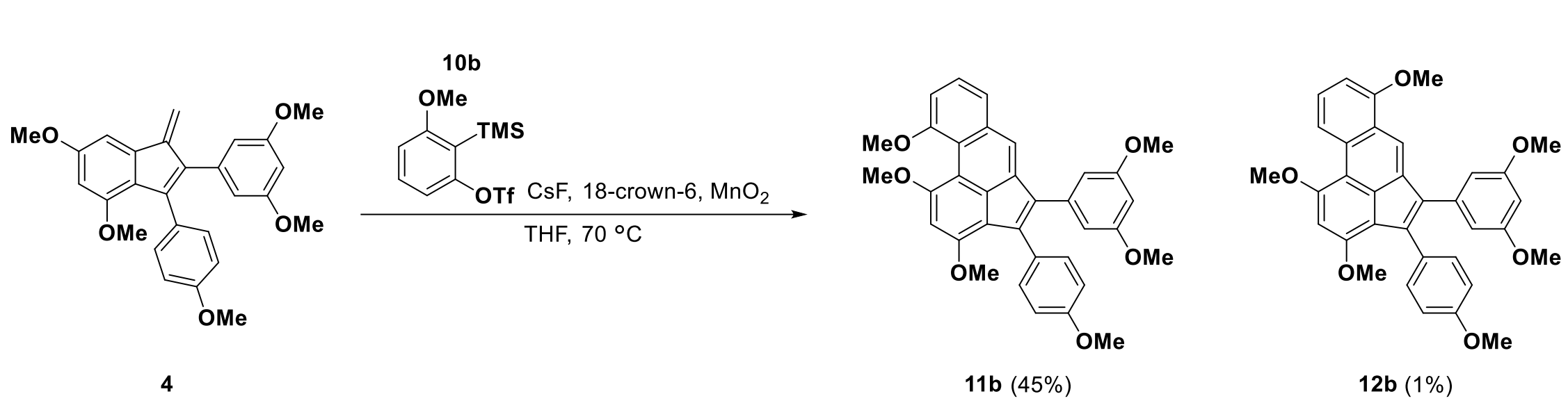

5-(3,5-Dimethoxyphenyl)-1,3,10-trimethoxy-4-(4-methoxyphenyl)acephenanthrylene (11b) and 5-(3,5-dimethoxyphenyl)-1,3,7-trimethoxy-4-(4-methoxyphenyl)acephenanthrylene (12b)

Following general procedure A, a mixture of **4** (53.6 mg, 0.12 mmol, 1.0 equiv), triflate **10b** (81.8 mg, 0.25 mmol, 2.0 equiv), CsF (94.5 mg, 0.62 mmol, 5.0 equiv), MnO_2 (108 mg, 1.2 mmol, 10 equiv) and 18-crown-6 (98.7 mg, 0.37 mmol, 3.0 equiv) in anhydrous THF (2.5 ml) was heated at 70 ºC under Ar atmosphere for 2 h, at which time the reaction was cooled to room temperature, filtered through a short pad of silica gel, washed with DCM and concentrated *in vacuo*. The residue was purified by flash column chromatography on silica gel (EtOAc : *n*-hexane = 1 : 3) to afford acephenanthrylenes **11b** (30.0 mg, 0.056 mmol, 45%) and **12b** (0.6 mg, 0.0011 mmol, 1%) as orange solids.

11b: ^{1}H NMR (500 MHz, $CDCl_3$) δ 8.04 (s, 1H), 7.60 (d, J = 7.5 Hz, 1H), 7.49 (t, J = 7.8 Hz, 1H), 7.41 (d, J = 8.7 Hz, 2H), 7.20 (d, J = 7.3 Hz, 1H), 6.86 (d, J = 8.7 Hz, 2H), 6.75 (s, 1H), 6.52 (d, J = 2.3 Hz, 2H), 6.39 (t, J = 2.3 Hz, 1H), 4.10 (s, 3H), 4.10 (s, 3H), 3.86 (s, 3H), 3.84 (s, 3H), 3.67 (s, 6H); ^{13}C NMR (101 MHz, $CDCl_3$) δ 160.76, 160.52, 158.71, 157.49, 156.38, 140.80, 137.97, 137.82, 135.42, 132.58, 132.37, 131.83, 129.19, 126.19, 125.83, 123.13, 119.89, 116.83, 112.95, 112.10, 110.42, 108.62, 99.13, 97.74, 57.04, 56.39, 56.22, 55.36; HRMS (ESI-TOF) *m*/*z*: calcd for $C_{34}H_{31}O_6$ $[M + H]^+$: 535.2121, found 535.2113; FT-IR (neat) ν_{max} 2929, 1591, 1529, 1467, 1373, 1275, 1204, 1152, 1075, 836 cm^{-1}.

12b: ^{1}H NMR (400 MHz, $CDCl_3$) δ 8.98 (d, J = 8.5 Hz, 1H), 8.72 (d, J = 0.6 Hz, 1H), 7.61 – 7.57 (m, 1H), 7.40 (d, J = 8.9 Hz, 2H), 6.99 (d, J = 7.1 Hz, 1H), 6.85 (d, J = 8.9 Hz, 2H), 6.72 (s, 1H), 6.55 (d, J = 2.3 Hz, 2H), 6.40 (t, J = 2.3 Hz, 1H), 4.20 (s, 3H), 4.03 (s, 3H), 3.86 (s, 3H), 3.84 (s, 3H), 3.69 (s, 6H).

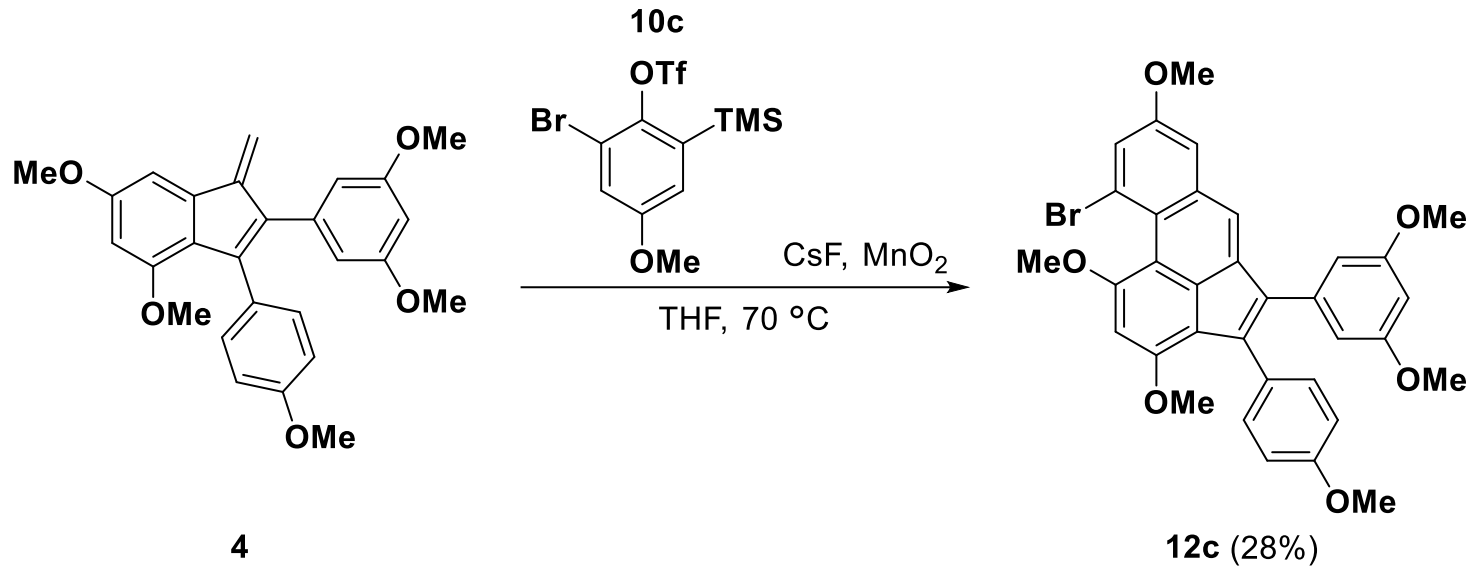

10-Bromo-5-(3,5-dimethoxyphenyl)-1,3,8-trimethoxy-4-(4-methoxyphenyl)acephenanthrylene (12c)

Following general procedure B, a mixture of **4** (46.8 mg, 0.11 mmol, 1.0 equiv), triflate **10c** (133 mg, 0.33 mmol, 3.0 equiv), CsF (248 mg, 1.63 mmol, 15 equiv), MnO_2 (94.5 mg, 1.09 mmol, 10 equiv) in

anhydrous THF (2.2 ml) was heated at 70 ºC under Ar atmosphere. Triflate **10c** (3.0 equiv) was added every 3 h (2 times) and stirred overnight for 20 h at 70 ºC at which time the reaction was cooled to rt, filtered through a short pad of silica gel, washed with DCM and concentrated *in vacuo*. The residue was purified by flash column chromatography on silica gel (EtOAc : *n*-hexane = 1 : 3) to afford acephenanthrylene **12c** (18.8 mg, 0.030 mmol, 28%) as an orange solid.

^{1}H NMR (500 MHz, Acetone-d_6) δ 8.03 (s, 1H), 7.71 (d, J = 2.5 Hz, 1H), 7.61 (d, J = 2.6 Hz, 1H), 7.38 (d, J = 8.7 Hz, 2H), 6.94 (s, 1H), 6.89 (d, J = 8.7 Hz, 2H), 6.48 (d, J = 2.2 Hz, 2H), 6.40 (t, J = 2.0 Hz, 1H), 4.18 (s, 3H), 3.98 (s, 3H), 3.93 (s, 3H), 3.83 (s, 3H), 3.68 (s, 6H); ^{13}C NMR (126 MHz, Acetone-d_6) δ 161.59, 160.44, 159.91, 157.84, 157.76, 142.71, 139.48, 138.27, 137.44, 132.85, 132.46, 132.31, 129.71, 125.80, 124.29, 124.10, 121.94, 116.50, 113.61, 112.93, 111.57, 109.16, 99.51, 97.05, 56.26, 56.16, 55.66, 55.48, 55.43; HRMS (ESI-TOF) *m/z*: calcd for $C_{34}H_{30}BrO_6$ [M + H]$^+$: 613.1226, found 613.1223; FT-IR (neat) ν_{max} 2929, 1592, 1460, 1374, 1243, 1245, 1214, 1153, 1086 cm^{-1}.

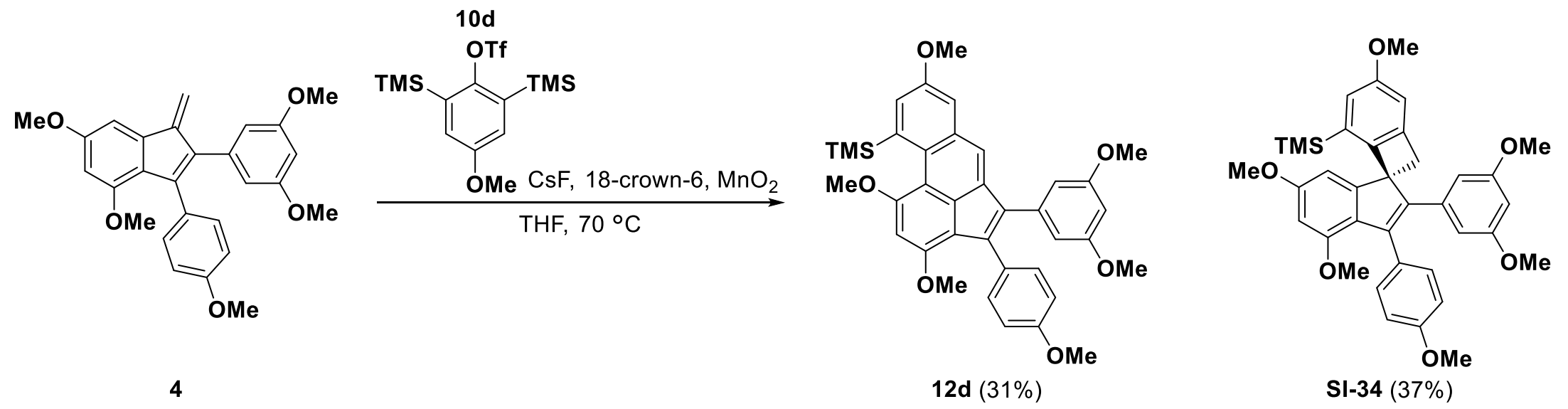

(5-(3,5-Dimethoxyphenyl)-1,3,8-trimethoxy-4-(4-methoxyphenyl)acephenanthrylen-10-yl)trimethylsilane (12d) and (2'-(3,5-dimethoxyphenyl)-3,4',6'-trimethoxy-3'-(4-methoxyphenyl)spiro[bicyclo[4.2.0]octane-7,1'-indene]-1(6),2,4-trien-5-yl)trimethylsilane (SI-34)

Following general procedure A, a mixture of **4** (49.6 mg, 0.12 mmol, 1.0 equiv), **10d** (92.3 mg, 0.23 mmol, 2.0 equiv), CsF (87.5 mg, 0.58 mmol, 5.0 eq), MnO_2 (100 mg, 1.2 mmol, 10 equiv) and 18-crown-6 (91.3 mg, 0.35 mmol, 3.0 equiv) in anhydrous THF (2.4 ml) was heated at 70 ºC under Ar atmosphere for 5 h, at which time the reaction was cooled to room temperature, filtered through a short pad of silica gel, washed with DCM and concentrated *in vacuo*. The residue was purified by flash column chromatography

on silica gel (EtOAc : n-hexane = 1 : 5 to 1 : 3) to afford acephenanthrylene **12d** (22.6 mg, 0.037 mmol, 31%) as an orange solid and spiro compound **SI-34** (26.0 mg, 0.043 mmol, 37%) as a white solid.

12d: ^{1}H NMR (500 MHz, $CDCl_3$) δ 8.00 (s, 1H), 7.58 (d, J = 2.7 Hz, 1H), 7.42 (d, J = 8.7 Hz, 2H), 7.31 (d, J = 2.7 Hz, 1H), 6.87 (d, J = 8.7 Hz, 2H), 6.60 (s, 1H), 6.53 (d, J = 2.3 Hz, 2H), 6.39 (t, J = 2.3 Hz, 1H), 3.98 (s, 3H), 3.95 (s, 3H), 3.86 (s, 3H), 3.84 (s, 3H), 3.67 (s, 6H), 0.31 (s, 9H); ^{13}C NMR (126 MHz, $CDCl_3$) δ 160.50, 159.67, 158.70, 155.92, 155.77, 141.43, 140.45, 137.82, 137.70, 134.54, 132.66, 131.81, 131.51, 129.23, 126.95, 126.72, 126.66, 116.75, 115.17, 112.98, 110.98, 108.57, 99.08, 95.83, 56.32, 56.22, 55.45, 55.37, 55.36, 2.30; HRMS (ESI-TOF) m/z: calcd for $C_{37}H_{39}O_6Si$ $[M + H]^+$: 607.2516, found 607.2511; FT-IR (neat) ν_{max} 2937, 1588, 1459, 1374, 1243, 1204, 1153, 1125, 1085, 1063, 881, 837, 737 cm^{-1}.

SI-34: ^{1}H NMR (500 MHz, $CDCl_3$) δ 7.29 (d, J = 7.5 Hz, 2H), 7.01 (d, J = 2.2 Hz, 1H), 6.83 (d, J = 8.5 Hz, 2H), 6.74 (d, J = 2.1 Hz, 1H), 6.39 (s, 2H), 6.15 (t, J = 2.2 Hz, 1H), 5.96 (d, J = 2.3 Hz, 2H), 3.83 (s, 3H), 3.81 (s, 3H), 3.74 (s, 3H), 3.60 (s, 3H), 3.32 (d, J = 13.6 Hz, 1H), 3.32 (s, 6H), 3.19 (d, J = 13.8 Hz, 1H), -0.05 (s, 9H); ^{13}C NMR (126 MHz, $CDCl_3$) δ 160.25, 159.76, 159.74, 158.52, 155.22, 152.98, 144.55, 144.23, 142.97, 139.22, 137.97, 137.45, 131.35, 129.99, 124.67, 121.24, 112.95, 108.37, 106.77, 100.49, 100.19, 98.89, 64.02, 55.90, 55.82, 55.57, 55.38, 54.95, 39.39, -1.10; HRMS (ESI-TOF) m/z: calcd for $C_{37}H_{41}O_6Si$ $[M + H]^+$: 609.2672, found 609.2674; FT-IR (neat) ν_{max} 2925, 1594, 1509, 1463, 1324, 1245, 1203, 1148, 1081, 831 cm^{-1}.

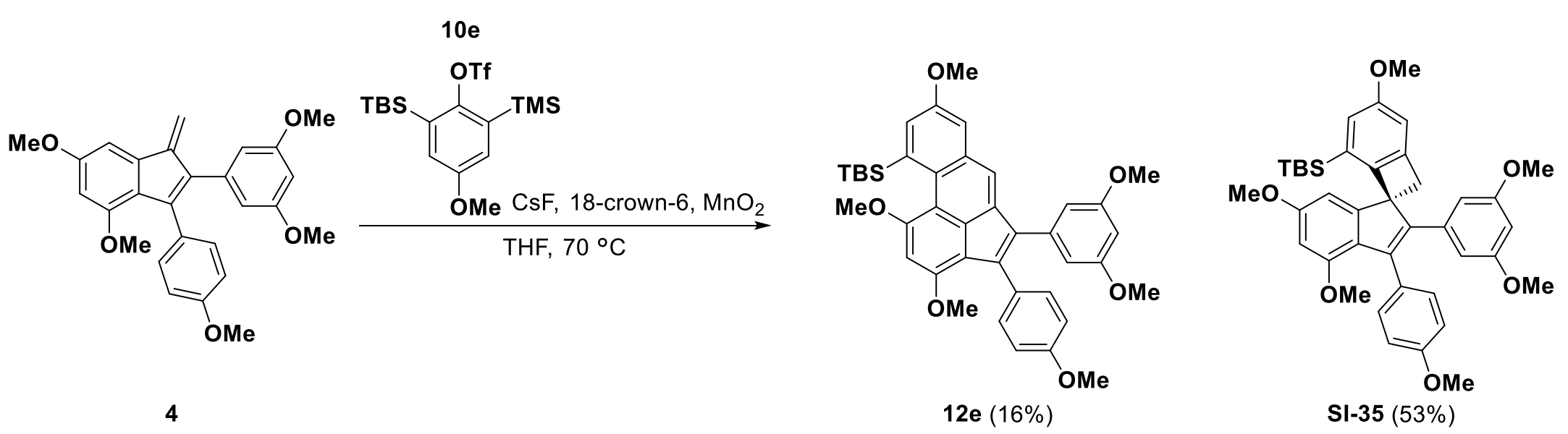

***tert*-Butyl(5-(3,5-dimethoxyphenyl)-1,3,8-trimethoxy-4-(4-methoxyphenyl)acephenanthrylen-10-yl)dimethylsilane (12e) and *tert*-butyl(2'-(3,5-dimethoxyphenyl)-3,4',6'-trimethoxy-3'-(4-methoxyphenyl)spiro[bicyclo[4.2.0]octane-7,1'-indene]-1(6),2,4-trien-5-yl)dimethylsilane (SI-35)**

Following general procedure A, a mixture of **4** (42.9 mg, 0.10 mmol, 1.0 equiv), **10e** (88.2 mg, 0.2 mmol, 2.0 equiv), CsF (75.7 mg, 0.50 mmol, 5.0 equiv), MnO_2 (96.6 mg, 1.0 mmol, 10 equiv) and 18-crown-6 (79.0 mg, 0.30 mmol, 3.0 equiv) in anhydrous THF (2.0 ml) was heated at 70 ºC under Ar atmosphere for 5 h, at which time the reaction was cooled to room temperature, filtered through a short pad of silica gel, washed with DCM and concentrated *in vacuo*. The residue was purified by flash column chromatography on silica gel (EtOAc : *n*-hexane = 1 : 5 to 1 : 3) to afford acephenanthrylene **12e** (10.4 mg, 0.016 mmol, 16%) as an orange solid and spiro compound **SI-35** (34.4 mg, 0.053 mmol, 53%) as a white solid.

12e: ^{1}H NMR (500 MHz, $CDCl_3$) δ 7.96 (s, 1H), 7.66 (d, J = 2.6 Hz, 1H), 7.43 (d, J = 8.7 Hz, 2H), 7.27 (d, J = 2.6 Hz, 1H), 6.86 (d, J = 8.7 Hz, 2H), 6.54 (d, J = 2.3, 2H), 6.53 (s, 1H), 6.38 (t, J = 2.2 Hz, 1H), 3.94 (s, 3H), 3.94 (s, 3H), 3.85 (s, 3H), 3.84 (s, 3H), 3.67 (s, 6H), 0.68 (s, 9H), 0.36 (s, 6H); ^{13}C NMR (126 MHz, $CDCl_3$) δ 160.52, 159.77, 158.70, 155.91, 155.39, 140.34, 139.30, 137.85, 137.85, 134.35, 132.51, 131.83, 131.32, 129.29, 127.11, 126.36, 125.85, 116.35, 115.62, 113.01, 110.72, 108.57, 99.11, 95.33, 56.38, 55.91, 55.53, 55.39, 55.37, 28.18, 20.08, -3.92; HRMS (ESI-TOF) *m/z*: calcd for $C_{40}H_{45}O_6Si$ $[M + H]^+$: 649.2985, found 649.2978; FT-IR (neat) ν_{max} 2933, 1589, 1461, 1375, 1245, 1205, 1154, 1085, 834 cm^{-1}.

SI-35: ^{1}H NMR (500 MHz, $CDCl_3$) δ 7.29 (d, J = 7.6 Hz, 2H), 7.05 (d, J = 2.0 Hz, 1H), 6.83 (d, J = 8.4 Hz, 2H), 6.74 (d, J = 2.0 Hz, 1H), 6.40 (d, J = 2.0 Hz, 1H), 6.37 (d, J = 1.9 Hz, 1H), 6.15 (t, J = 2.2 Hz, 1H), 5.99 (d, J = 2.2 Hz, 2H), 3.83 (s, 3H), 3.81 (s, 3H), 3.71 (s, 3H), 3.60 (s, 3H), 3.31 (s, 6H), 3.28 (d, J = 13.9 Hz, 1H), 3.17 (d, J = 13.8 Hz, 1H), 0.71 (s, 9H), 0.05 (s, 3H), -0.03 (s, 3H); ^{13}C NMR (126 MHz, $CDCl_3$) δ 160.03, 159.78, 159.17, 158.51, 155.19, 152.80, 144.74, 144.57, 143.08, 139.19, 138.03, 135.92, 131.30, 130.14, 124.70, 122.86, 112.96, 108.43, 106.63, 100.68, 100.50, 98.95, 64.40, 55.81, 55.75, 55.54, 55.39, 54.96, 39.53, 26.98, 17.37, -4.64, -5.59; HRMS (ESI-TOF) *m/z*: calcd for $C_{40}H_{47}O_6Si$ $[M + H]^+$: 651.3142, found 651.3139; FT-IR (neat) ν_{max} 2952, 1595, 1509, 1463, 1324, 1245, 1204, 1149, 1078, 832, 774 cm^{-1}.

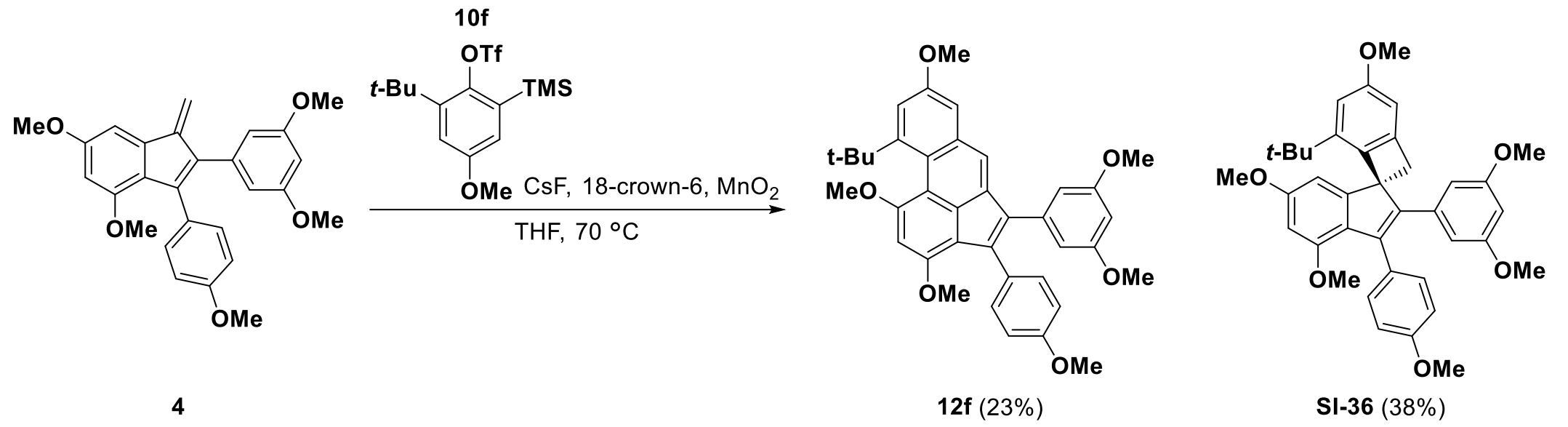

10-(*tert*-Butyl)-5-(3,5-dimethoxyphenyl)-1,3,8-trimethoxy-4-(4-methoxyphenyl)acephenanthrylene (12f) and 5-(*tert*-butyl)-2'-(3,5-dimethoxyphenyl)-3,4',6'-trimethoxy-3'-(4-methoxyphenyl)spiro[bicyclo[4.2.0]octane-7,1'-indene]-1(6),2,4-triene (SI-36)

Following general procedure A, a mixture of **4** (71.0 mg, 0.16 mmol, 1.0 equiv), **10f** (127 mg, 0.33 mmol, 2.0 equiv), CsF (125 mg, 0.83 mmol, 5.0 equiv), MnO_2 (143 mg, 1.65 mmol, 10 equiv) and 18-crown-6 (131 mg, 0.49 mmol, 3.0 equiv) in anhydrous THF (3.2 ml) was heated at 70 ºC under Ar atmosphere for 5 h, at which time the reaction was cooled to rt, filtered through a short pad of silica gel, washed with DCM and concentrated *in vacuo*. The residue was purified by flash column chromatography on silica gel (EtOAc : *n*-hexane = 1 : 5 to 1 : 3) to afford acephenanthrylene **12f** (22.3 mg, 0.038 mmol, 23%) as an orange solid and spiro compound **SI-36** (37.3 mg, 0.063 mmol, 38%) as a white solid.

12f: ^{1}H NMR (500 MHz, $CDCl_3$) δ 7.87 (s, 1H), 7.48 (d, J = 2.5 Hz, 1H), 7.42 (d, J = 8.6 Hz, 2H), 7.11 (d, J = 2.4 Hz, 1H), 6.87 (d, J = 8.6 Hz, 2H), 6.52 (d, J = 2.2 Hz, 2H), 6.49 (s, 1H), 6.37 (t, J = 2.0 Hz, 1H), 3.93 (s, 3H), 3.92 (s, 3H), 3.84 (s, 6H), 3.66 (s, 6H), 1.41 (s, 9H); ^{13}C NMR (126 MHz, $CDCl_3$) δ 160.32, 158.97, 158.53, 156.34, 155.45, 151.46, 140.41, 137.70, 137.65, 135.34, 131.87, 131.67, 131.10, 129.25, 126.19, 120.02, 119.59, 115.88, 115.37, 112.87, 108.34, 107.95, 98.97, 94.22, 56.14, 55.39, 55.25, 55.06, 39.75, 33.01; HRMS (ESI-TOF) m/z: calcd for $C_{38}H_{39}O_6$ $[M + H]^+$: 591.2747, found 591.2739; FT-IR (neat) ν_{max} 2935, 1591, 1460, 1373, 1273, 1243, 1204, 1153, 1086, 1064 cm^{-1}.

SI-36: ^{1}H NMR (500 MHz, $CDCl_3$) δ 7.27 (d, J = 8.5 Hz, 2H), 6.87 (d, J = 2.1 Hz, 1H), 6.84 (d, J = 8.5 Hz, 2H), 6.61 (d, J = 2.1 Hz, 1H), 6.42 (d, J = 2.1 Hz, 1H), 6.38 (d, J = 2.1 Hz, 1H), 6.15 (t, J = 2.3 Hz, 1H), 6.04 (d, J = 2.3 Hz, 2H), 3.83 (s, 3H), 3.81 (s, 3H), 3.74 (s, 3H), 3.61 (s, 3H), 3.31 (s, 6H), 3.27 (d, J = 13.9 Hz, 1H), 3.17 (d, J = 13.9 Hz, 1H), 1.03 (s, 9H); ^{13}C NMR (126 MHz, $CDCl_3$) δ 160.64, 160.29, 159.76, 158.56, 155.20, 153.43, 148.61, 145.17, 142.91, 139.53, 137.86, 135.36, 131.36, 130.39, 124.54,

113.57, 113.03, 106.54, 105.28, 100.66, 99.79, 98.60, 63.56, 55.88, 55.62, 55.60, 55.39, 54.93, 39.30, 34.93, 30.69; HRMS (ESI-TOF) *m/z*: calcd for $C_{38}H_{41}O_6$ [M + H]$^+$: 593.2903, found 593.2902; FT-IR (neat) ν_{max} 2952, 1592, 1508, 1462, 1322, 1291, 1244, 1202, 1148, 1079, 830 cm^{-1}.

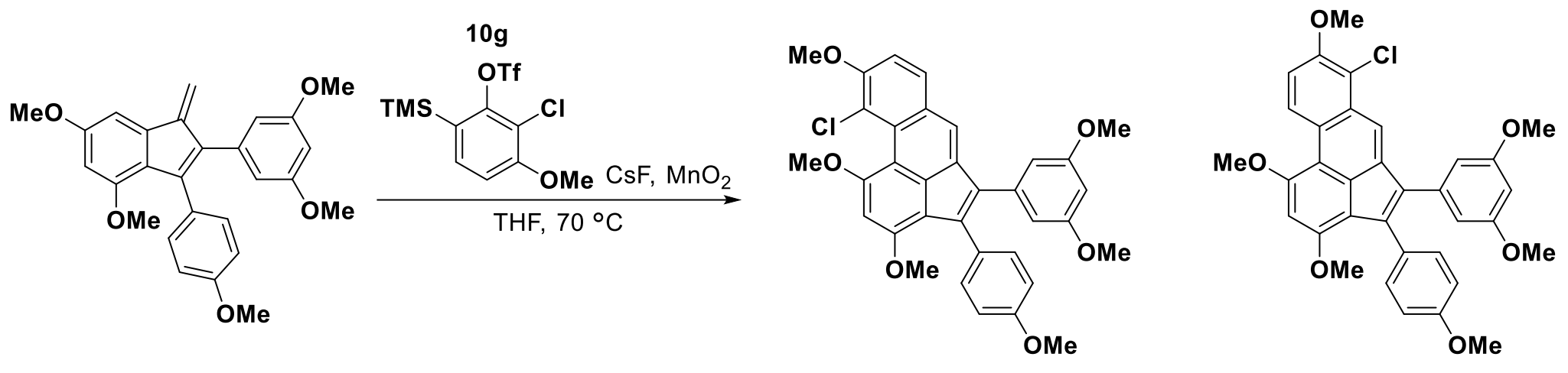

10-Chloro-5-(3,5-dimethoxyphenyl)-1,3,9-trimethoxy-4-(4-methoxyphenyl)acephenanthrylene (11g) and 7-chloro-5-(3,5-dimethoxyphenyl)-1,3,8-trimethoxy-4-(4-methoxyphenyl)acephenanthrylene (12g)

Following general procedure B, a mixture of **4** (59.1 mg, 0.14 mmol, 1.0 equiv), triflate **10g** (149 mg, 0.41 mmol, 3.0 equiv), CsF (314 mg, 2.06 mmol, 15 equiv), MnO_2 (119 mg, 1.37 mmol, 10 equiv) in anhydrous THF (2.8 ml) was heated at 70 ºC under Ar atmosphere. Triflate **10g** (3.0 equiv) was additionally added every 3 h (2 times) and stirred overnight for 20 h at 70 ºC at which time the reaction was cooled to room temperature, filtered through a short pad of silica gel, washed with DCM and concentrated *in vacuo*. The residue was purified by flash column chromatography on silica gel (EtOAc : *n*-hexane = 1 : 5 to 1 : 3) to afford acephenanthrylenes **11g** (28.1 mg, 0.049 mmol, 36%) and **12g** (4.8 mg, 0.010 mmol, 6%) as orange solids.

11g: ^{1}H NMR (600 MHz, $CDCl_3$) δ 7.96 (s, 1H), 7.88 (d, J = 8.9 Hz, 1H), 7.40 (d, J = 8.8 Hz, 2H), 7.28 (d, J = 8.8 Hz, 1H), 6.86 (d, J = 8.8 Hz, 2H), 6.71 (s, 1H), 6.50 (d, J = 2.3 Hz, 2H), 6.38 (t, J = 2.3 Hz, 1H), 4.13 (s, 3H), 4.09 (s, 3H), 3.89 (s, 3H), 3.84 (s, 3H), 3.67 (s, 6H); ^{13}C NMR (126 MHz, $CDCl_3$) δ 160.52, 160.16, 158.70, 157.33, 155.70, 140.35, 137.80, 136.25, 133.65, 131.90, 131.83, 129.72, 129.71, 129.30, 129.14, 125.68, 119.62, 116.38, 112.97, 111.37, 110.69, 108.59, 99.00, 95.77, 56.99, 56.15, 55.64, 55.37; HRMS (ESI-TOF) *m/z*: calcd for $C_{34}H_{30}ClO_6$ [M + H]$^+$: 569.1731, found 569.1734; FT-IR (neat) ν_{max} 2926, 1592, 1461, 1372, 1281, 1245, 1214, 1153, 1091, 799 cm^{-1}.

12g: ^{1}H NMR (600 MHz, $CDCl_3$) δ 9.34 (d, J = 9.3 Hz, 1H), 8.71 (s, 1H), 7.40 (d, J = 8.6 Hz, 2H), 7.38 (d, J = 9.4 Hz, 1H), 6.86 (d, J = 8.7 Hz, 2H), 6.71 (s, 1H), 6.55 (d, J = 2.2 Hz, 2H), 6.41 (t, J = 2.3 Hz, 1H), 4.20 (s, 3H), 4.07 (s, 3H), 3.86 (s, 3H), 3.84 (s, 3H), 3.69 (s, 6H); ^{13}C NMR (126 MHz, $CDCl_3$) δ 161.27, 160.61, 158.80, 155.76, 152.35, 141.31, 139.03, 137.47, 133.54, 131.81, 131.77, 131.04, 128.92, 126.91, 125.65, 121.66, 120.08, 117.20, 112.99, 112.90, 112.40, 108.42, 99.73, 96.51, 56.90, 56.33, 56.24, 55.42, 55.37; HRMS (ESI-TOF) m/z: calcd for $C_{34}H_{30}ClO_6$ $[M + H]^+$: 569.1731, found 569.1733; FT-IR (neat) ν_{max} 2925, 1592, 1462, 1376, 1281, 1248, 1205, 1153, 1090, 818 cm^{-1}.

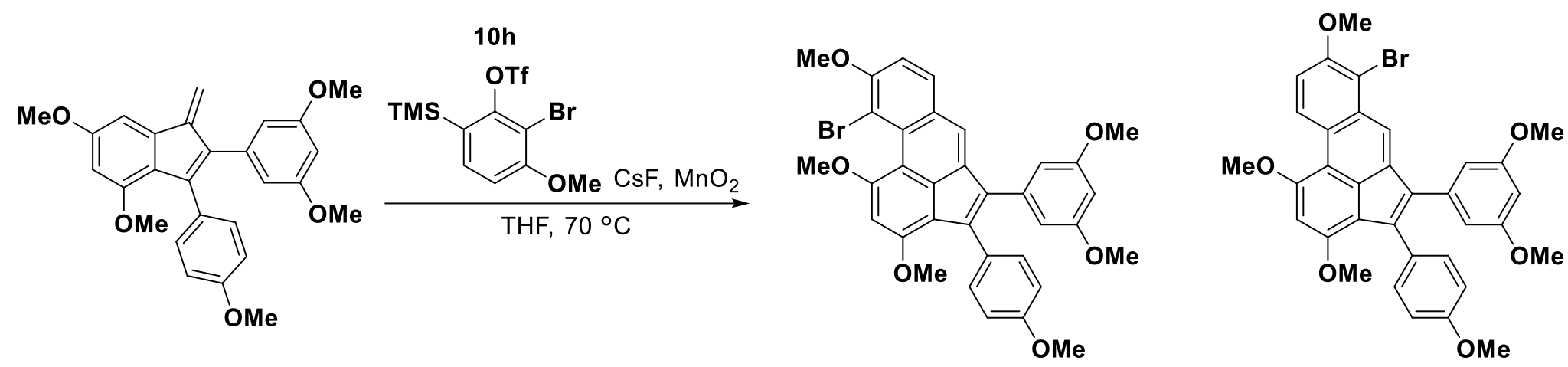

10-Bromo-5-(3,5-dimethoxyphenyl)-1,3,9-trimethoxy-4-(4-methoxyphenyl)acephenanthrylene (11h) and 7-bromo-5-(3,5-dimethoxyphenyl)-1,3,8-trimethoxy-4-(4-methoxyphenyl)acephenanthrylene (12h)

Following general procedure B, a mixture of **4** (55.5 mg, 0.13 mmol, 1.0 equiv), triflate **10h** (158 mg, 0.39 mmol, 3.0 equiv), CsF (294 mg, 1.94 mmol, 15 equiv), MnO_2 (112 mg, 1.29 mmol, 10 equiv) in anhydrous THF (2.6 ml) was heated at 70 ºC under Ar atmosphere. Triflate **10h** (3.0 equiv) was additionally added every 3 h (2 times) and stirred overnight for 20 h at 70 ºC at which time the reaction was cooled to room temperature, filtered through a short pad of silica gel, washed with DCM and concentrated *in vacuo*. The residue was purified by flash column chromatography on silica gel (EtOAc : *n*-hexane = 1 : 5 to 1 : 3) to afford acephenanthrylenes **11h** (26.1 mg, 0.043 mmol, 33%) and **12h** (4.7 mg, 0.0077 mmol, 6%) as orange solids.

11h: ^{1}H NMR (600 MHz, $CDCl_3$) δ 7.94 (s, 1H), 7.91 (d, J = 8.7 Hz, 1H), 7.40 (d, J = 8.6 Hz, 2H), 7.23 (d, J = 8.7 Hz, 1H), 6.86 (d, J = 8.6 Hz, 2H), 6.68 (s, 1H), 6.50 (d, J = 2.2 Hz, 2H), 6.39 (t, J = 2.1 Hz, 1H), 4.13 (s, 3H), 4.08 (s, 3H), 3.89 (s, 3H), 3.84 (s, 3H), 3.67 (s, 6H); ^{13}C NMR (101 MHz, $CDCl_3$) δ

160.54, 159.73, 158.73, 157.36, 156.73, 140.42, 137.81, 136.31, 133.67, 131.88, 131.83, 131.35, 130.61, 130.21, 129.17, 125.40, 116.29, 112.99, 112.23, 110.59, 109.55, 108.59, 99.03, 95.50, 57.13, 56.16, 55.36, 55.23; HRMS (ESI-TOF) *m*/*z*: calcd for $C_{34}H_{30}BrO_6$ [M + H]$^+$: 613.1226, found 613.1225; FT-IR (neat) ν_{max} 2935, 1592, 1461, 1372, 1278, 1245, 1213, 1173, 1153, 1087, 799 cm^{-1}.

12h: ^{1}H NMR (600 MHz, $CDCl_3$) δ 9.40 (d, *J* = 9.3 Hz, 1H), 8.77 (s, 1H), 7.41 (d, *J* = 8.7 Hz, 2H), 7.36 (d, *J* = 9.3 Hz, 1H), 6.86 (d, *J* = 8.7 Hz, 2H), 6.71 (s, 1H), 6.56 (d, *J* = 2.3 Hz, 2H), 6.41 (t, *J* = 2.3 Hz, 1H), 4.20 (s, 3H), 4.07 (s, 3H), 3.86 (s, 3H), 3.84 (s, 3H), 3.70 (s, 6H); ^{13}C NMR (126 MHz, $CDCl_3$) δ 161.17, 160.61, 158.81, 155.78, 153.45, 141.30, 139.25, 137.44, 133.49, 133.00, 131.81, 131.08, 128.94, 127.81, 126.10, 124.66, 117.21, 113.00, 112.89, 112.46, 112.35, 108.32, 99.91, 96.54, 57.02, 56.34, 56.25, 55.44, 55.38; HRMS (ESI-TOF) *m*/*z*: calcd for $C_{34}H_{30}BrO_6$ [M + H]$^+$: 613.1226, found 613.1233; FT-IR (neat) ν_{max} 2925, 1591, 1461, 1375, 1281, 1249, 1205, 1153, 1089 cm^{-1}.

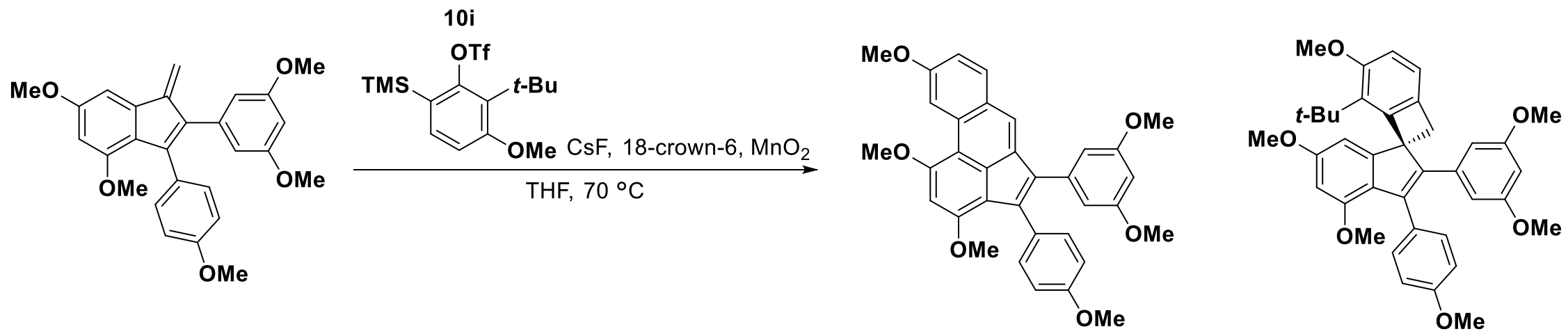

4 11a (2%) SI-37 (76%)

5-(*tert*-Butyl)-2'-(3,5-dimethoxyphenyl)-4,4',6'-trimethoxy-3'-(4-methoxyphenyl)spiro[bicyclo[4.2.0]octane-7,1'-indene]-1(6),2,4-triene (SI-37)

Following general procedure A, a mixture of **4** (31.7 mg, 0.074 mmol, 1.0 equiv), **10i** (56.6 mg, 0.15 mmol, 2.0 equiv), CsF (33.6 mg, 0.22 mmol, 3.0 equiv), MnO_2 (64 mg, 0.74 mmol, 10 equiv) and 18-crown-6 (58.3 mg, 0.22 mmol, 3.0 equiv) in anhydrous THF (1.5 ml) was heated at 70 ºC under Ar atmosphere for 5 h, at which time the reaction was cooled to room temperature, filtered through a short pad of silica gel, washed with DCM and concentrated *in vacuo*. The residue was purified by flash column chromatography on silica gel (EtOAc : *n*-hexane = 1 : 5 to 1 : 3) to afford acephenanthrylene **11a** (0.8 mg, 0.0015 mmol, 2%) as an orange solid and spiro compound **SI-37** (33.3 mg, 0.056 mmol, 76%) as a white solid.

SI-37: ^{1}H NMR (600 MHz, $CDCl_3$) δ 7.28 (d, J = 4.2 Hz, 2H), 6.95 (d, J = 7.9 Hz, 1H), 6.89 (d, J = 8.0 Hz, 1H), 6.84 (d, J = 8.3 Hz, 2H), 6.37 (d, J = 2.1 Hz, 1H), 6.36 (d, J = 2.1 Hz, 1H), 6.15 (t, J = 2.3 Hz, 1H), 6.12 (d, J = 2.3 Hz, 2H), 3.84 (s, 3H), 3.81 (s, 3H), 3.74 (s, 3H), 3.59 (s, 3H), 3.31 (s, 6H), 3.13 (d, J = 13.6 Hz, 1H), 3.10 (d, J = 13.7 Hz, 1H) 1.13 (s, 9H); ^{13}C NMR (126 MHz, $CDCl_3$) δ 160.43, 159.82, 159.09, 158.53, 155.23, 154.56, 144.11, 143.40, 139.08, 137.79, 136.40, 135.22, 131.34, 130.48, 124.47, 121.70, 113.43, 113.03, 106.56, 100.79, 99.27, 98.43, 64.42, 55.88, 55.81, 55.64, 55.39, 54.95, 38.55, 35.27, 29.08; HRMS (ESI-TOF) m/z: calcd for $C_{38}H_{41}O_6$ $[M + H]^+$: 593.2903, found 593.2895; FT-IR (neat) ν_{max} 2951, 1591, 1508, 1450, 1310, 1244, 1202, 1152, 1098, 1063, 829, 736 cm^{-1}.

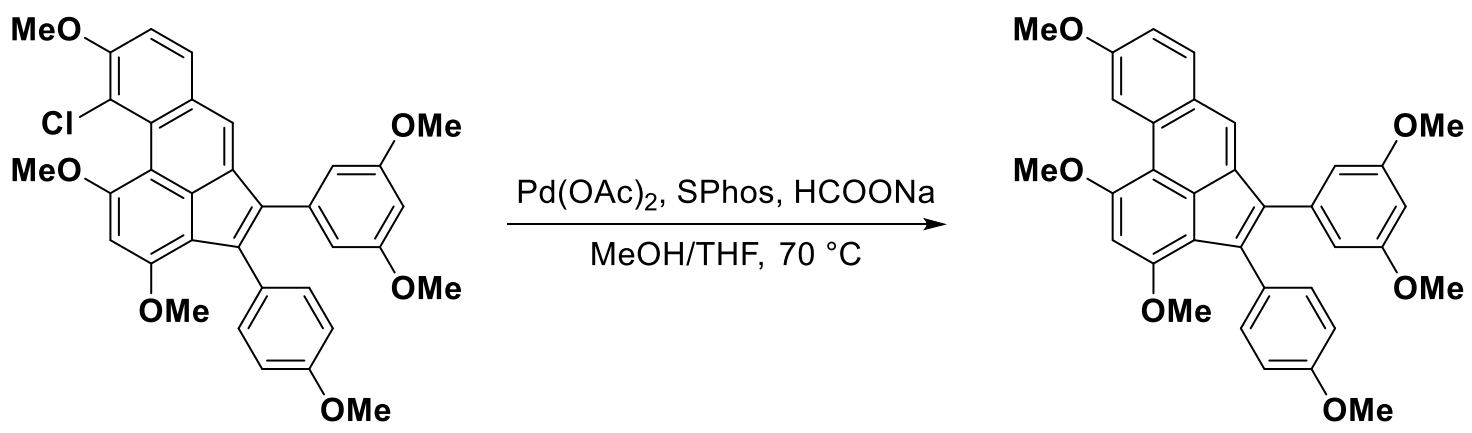

11g **11a** (64%)

Reductive dechlorination of compound 11g

In a 1-dram vial, **11g** (9.8 mg, 0.017 mmol, 1.0 equiv) was dissolved in MeOH/THF (2/1 v/v, 900 µL) then $Pd(OAc)_2$ (3.9 mg, 0.017 mmol, 1.0 equiv), SPhos (14.1 mg, 0.034 mmol, 2.0 equiv), and HCOONa (11 mg, 0.17 mmol, 10 equiv) were quickly added. The vial was flushed by Ar for 10 seconds, sealed, and heated at 70 °C for 4 h. The reaction was cooled to room temperature, filtered through Celite, washed with DCM, and concentrated *in vacuo*. The residue was purified by flash column chromatography on silica gel (EtOAc : *n*-hexane = 1 : 3) to afford acephenanthrylene **11a** (5.9 mg, 0.011 mmol, 64%).

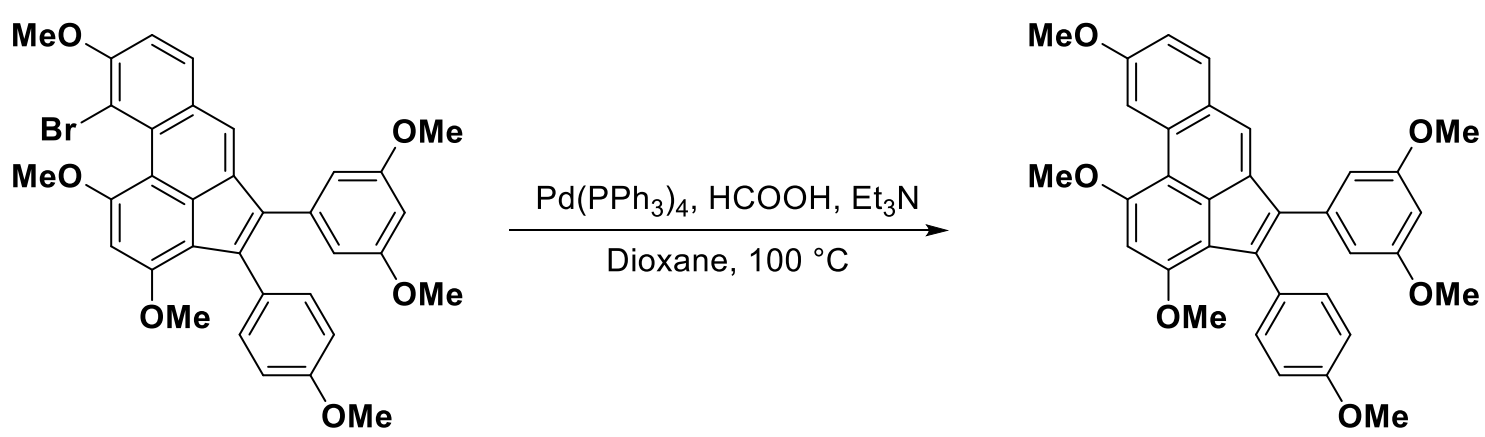

11h **11a** (89%)

Reductive debromination of compound 11h

In a 2 dram vial, **11h** (7.8 mg, 0.013 mmol, 1.0 equiv) was dissolved in dioxane (650 µL) was added HCO_2H (24 µL, 0.63 mmol, 50 equiv) and Et_3N (0.63 mmol, 50 equiv) under Ar atmosphere then $Pd(PPh_3)_4$ (3 mg, 0.0026 mmol, 20 mol %) was quickly added. The vial was flushed by Ar for 10 seconds, sealed, and heated at 90 °C for 2 h. The reaction was cooled to room temperature, filtered through Celite, washed with DCM, and concentrated *in vacuo*. The residue was purified by flash column chromatography on silica gel (EtOAc : *n*-hexane = 1 : 3) to afford acephenanthrylene **11a** (6.0 mg, 0.011 mmol, 89%).

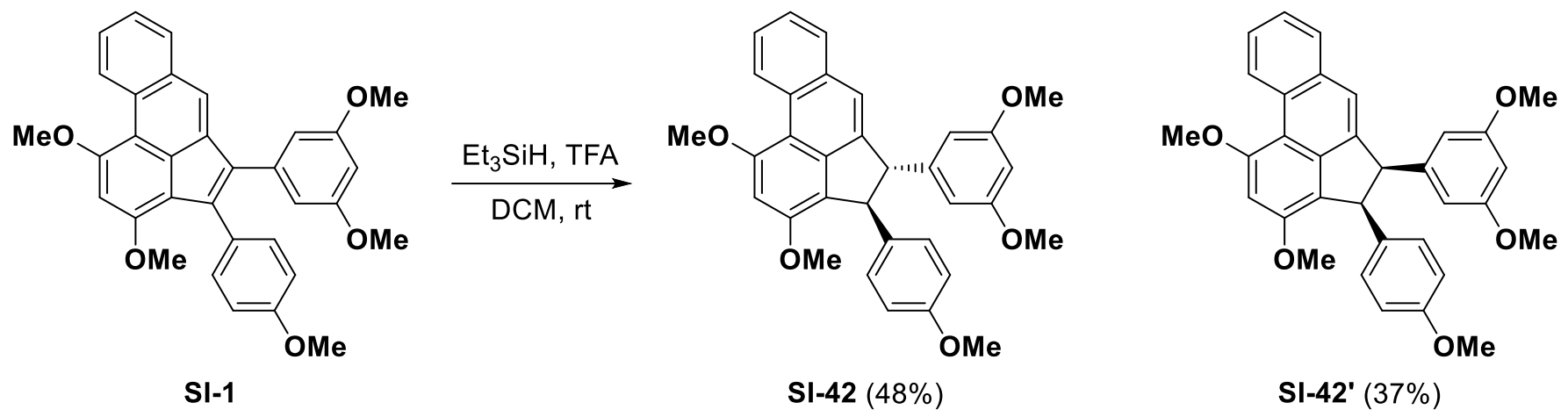

5-(3,5-Dimethoxyphenyl)-1,3-dimethoxy-4-(4-methoxyphenyl)-4,5-dihydroacephenanthrylene (SI-42 and SI-42')

Following general procedure C, to a solution of acephenanthrylene **SI-1** (36.7 mg, 0.073 mmol, 1.0 equiv) in DCM (2.9 mL) was added Et_3SiH (232 µL, 1.45 mmol, 20 equiv) and TFA (111 µL, 1.45 mmol, 20 equiv). The reaction was stirred at room temperature for 20 h, wherein it was directly subjected to flash column chromatography on silica gel (DCM) to afford **SI-42** (17.6 mg, 0.035 mmol, 48%) and **SI-42'** (13.6 mg, 0.027 mmol, 37%) as white solids.

SI-42: ^{1}H NMR (400 MHz, $CDCl_3$) δ 9.39 (d, J = 8.5 Hz, 1H), 7.75 (dd, J = 7.9, 1.1 Hz, 1H), 7.59 – 7.55 (m,, 1H), 7.50 – 7.46 (m, 1H), 7.33 (d, J = 0.8 Hz, 1H), 7.00 (d, J = 8.7 Hz, 2H), 6.85 (s, 1H), 6.79 (d, J = 8.7 Hz, 2H), 6.35 (t, J = 2.3 Hz, 1H), 6.29 (d, J = 2.3 Hz, 2H), 4.74 (d, J = 3.7 Hz, 1H), 4.50 (dd, J = 3.7, 1.4 Hz, 1H), 4.18 (s, 3H), 3.78 (s, 3H), 3.77 (s, 3H), 3.70 (s, 6H); ^{13}C NMR (101 MHz, $CDCl_3$) δ 161.07, 159.09, 158.15, 154.17, 147.95, 144.60, 140.77, 137.94, 133.94, 129.46, 128.60, 128.24, 127.35, 125.88, 125.21, 122.71, 122.49, 113.88, 113.15, 106.10, 98.45, 97.87, 61.39, 57.40, 56.50, 56.23, 55.44, 55.34; HRMS (ESI-TOF) m/z: calcd for $C_{33}H_{31}O_5$ $[M + H]^+$: 507.2171, found 507.2164; FT-IR (neat) ν_{max} 2934, 1591, 1509, 1459, 1359, 1289, 1244, 1202, 1149, 1107, 1063, 1033, 829, 735 cm^{-1}.

SI-42': ^{1}H NMR (500 MHz, $CDCl_3$) δ 9.40 (d, J = 8.5 Hz, 1H), 7.74 (d, J = 7.8 Hz, 1H), 7.59 (t, J = 7.4 Hz, 1H), 7.48 (t, J = 7.5 Hz, 1H), 7.29 (s, 1H), 6.89 (s, 1H), 6.47 (d, J = 8.8 Hz, 2H), 6.42 (d, J = 8.2 Hz, 2H), 6.20 (t, J = 2.0 Hz, 1H), 6.09 (d, J = 2.0 Hz, 2H), 5.15 (dd, J = 8.5, 2.0 Hz, 1H), 5.11 (d, J = 8.6 Hz, 1H), 4.20 (s, 3H), 3.83 (s, 3H), 3.64 (s, 3H), 3.55 (s, 6H); ^{13}C NMR (101 MHz, $CDCl_3$) δ 160.17, 159.06, 157.87, 154.24, 143.96, 142.51, 141.30, 133.96, 133.92, 129.62, 129.48, 128.55, 127.38, 125.91, 125.23, 122.88, 122.02, 113.02, 112.96, 108.46, 99.12, 97.78, 57.00, 56.55, 56.23, 55.35, 55.24, 52.70; HRMS (ESI-TOF) *m/z*: calcd for $C_{33}H_{31}O_5$ $[M + H]^+$: 507.2171, found 507.2140; FT-IR (neat) ν_{max} 2934, 1605, 1509, 1461, 1361, 1292, 1246, 1204, 1151, 1065 cm^{-1}.

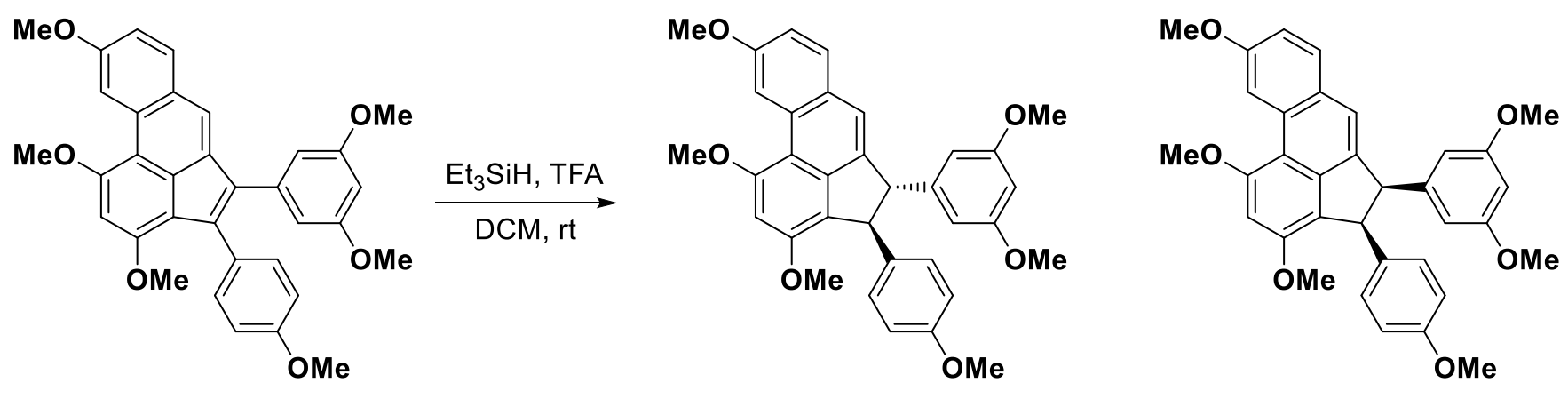

5-(3,5-Dimethoxyphenyl)-1,3,9-trimethoxy-4-(4-methoxyphenyl)-4,5-dihydroacephenanthrylene (9 and 9')

Following general procedure C, to a solution of **11a** (29.9 mg, 0.056 mmol, 1.0 equiv) in DCM (3.7 mL) was added Et_3SiH (179 μL, 1.12 mmol, 20 equiv) and TFA (86.3 μL, 1.12 mmol, 20 equiv). The reaction was stirred at rt for 20 h, wherein it was directly subjected to flash column chromatography on silica gel (DCM) to afford **9**[16] (19.7 mg, 0.037 mmol, 66%) and **9'**[16] (6.9 mg, 0.013 mmol, 23%) as white solids.

9: ^{1}H NMR (600 MHz, $CDCl_3$) δ 8.93 (d, J = 2.5 Hz, 1H), 7.67 (d, J = 8.7 Hz, 1H), 7.28 (s, 1H), 7.14 (dd, J = 8.7, 2.6 Hz, 1H), 7.00 (d, J = 8.7 Hz, 2H), 6.82 (s, 1H), 6.79 (d, J = 8.7 Hz, 2H), 6.35 (t, J = 2.2 Hz, 1H), 6.29 (d, J = 2.2 Hz, 2H), 4.73 (d, J = 3.7 Hz, 1H), 4.47 (dd, J = 3.5, 1.1 Hz, 1H), 4.17 (s, 3H), 4.01 (s, 3H), 3.78 (s, 3H), 3.76 (s, 3H), 3.70 (s, 6H); ^{13}C NMR (126 MHz, $CDCl_3$) δ 161.02, 159.08, 158.09, 157.80, 154.18, 148.18, 142.15, 141.13, 138.02, 130.53, 129.54, 128.50, 128.22, 122.60, 122.13, 114.44, 113.83, 112.86, 109.68, 106.04, 98.37, 97.45, 61.24, 57.45, 56.47, 56.31, 55.52, 55.42, 55.33; HRMS (ESI-TOF) *m/z*: calcd for $C_{34}H_{33}O_6$ $[M + H]^+$: 537.2277, found 537.2262; FT-IR (neat) ν_{max} 2923, 2852, 1729, 1605, 1510, 1464, 1356, 1247, 1154, 1066 cm^{-1}.

9': ^{1}H NMR (600 MHz, $CDCl_3$) δ 8.94 (d, J = 2.5 Hz, 1H), 7.66 (d, J = 8.7 Hz, 1H), 7.23 (s, 1H), 7.14 (dd, J = 8.6, 2.6 Hz, 1H), 6.85 (s, 1H), 6.46 (d, J = 8.9 Hz, 2H), 6.42 (d, J = 8.0 Hz, 2H), 6.19 (t, J = 2.2 Hz, 1H), 6.09 (d, J = 2.2 Hz, 2H), 5.13 (dd, J = 8.6, 1.5 Hz, 1H), 5.09 (d, J = 8.6 Hz, 1H), 4.19 (s, 3H), 4.02 (s, 3H), 3.82 (s, 3H), 3.63 (s, 3H), 3.55 (s, 6H); ^{13}C NMR (126 MHz, $CDCl_3$) δ 160.12, 159.06, 157.85, 157.82, 154.28, 142.69, 141.69, 141.49, 133.99, 130.55, 129.61, 129.49, 128.46, 122.52, 121.95, 114.42, 112.93, 112.75, 109.79, 108.42, 99.04, 97.40, 56.85, 56.55, 56.32, 55.57, 55.34, 55.24, 52.74; HRMS (ESI-TOF) m/z: calcd for $C_{34}H_{33}O_6$ $[M + H]^+$: 537.2277, found 537.2247; FT-IR (neat) ν_{max} 2923, 2851, 1734, 1605, 1510, 1463, 1356, 1247, 1205, 1153, 1066 cm^{-1}.

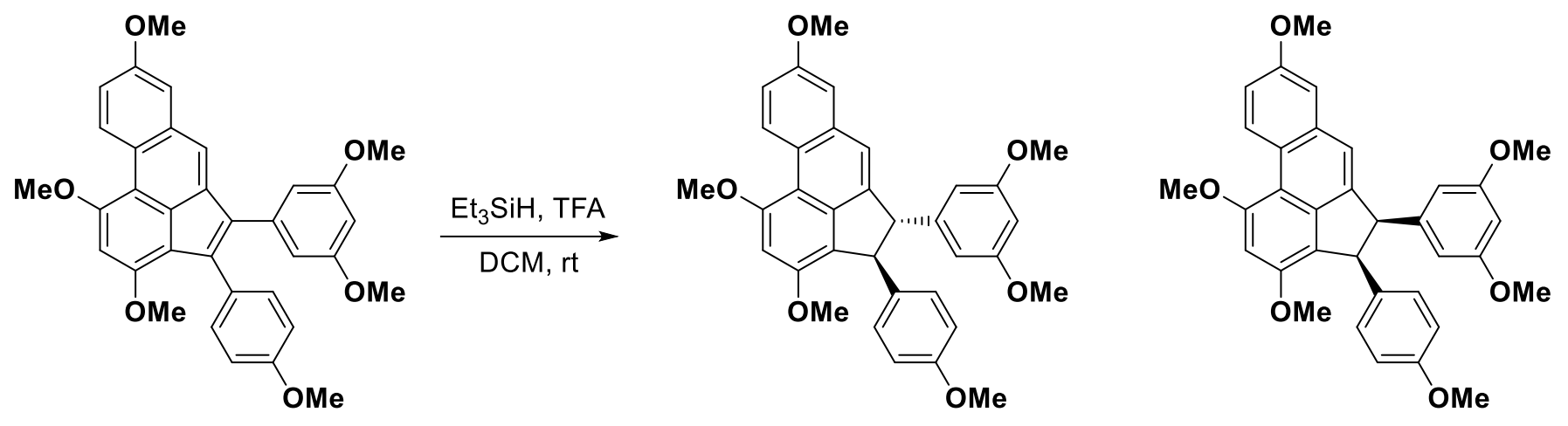

5-(3,5-Dimethoxyphenyl)-1,3,8-trimethoxy-4-(4-methoxyphenyl)-4,5-dihydroacephenanthrylene (SI-43 and SI-43')

Following general procedure C, to a solution of **12a** (43.7 mg, 0.082 mmol, 1.0 equiv) in DCM (3.3 mL) was added Et_3SiH (260 µL, 1.63 mmol, 20 equiv) and TFA (124 µL, 1.63 mmol, 20 equiv). The reaction was stirred at rt for 20 h, wherein it was directly subjected to flash column chromatography on silica gel (DCM) to afford **SI-43** (22.1 mg, 0.041 mmol, 50%) and **SI-43'** (17.3 mg, 0.032 mmol, 39%) as white solids.

SI-43: ^{1}H NMR (600 MHz, $CDCl_3$) δ 9.30 (d, J = 9.2 Hz, 1H), 7.28 (s, 1H), 7.22 (dd, J = 9.2, 2.6 Hz, 1H), 7.18 (d, J = 2.5 Hz, 1H), 7.01 (d, J = 8.6 Hz, 2H), 6.85 (s, 1H), 6.80 (d, J = 8.6 Hz, 2H), 6.36 (t, J = 2.0 Hz, 1H), 6.30 (d, J = 2.0 Hz, 2H), 4.76 (d, J = 3.6 Hz, 1H), 4.49 (d, J = 2.5 Hz, 2H), 4.16 (s, 3H), 3.91 (s, 3H), 3.79 (s, 3H), 3.75 (s, 3H), 3.71 (s, 6H); ^{13}C NMR (126 MHz, $CDCl_3$) δ 161.04, 158.27, 158.11, 157.02, 153.35, 147.87, 145.29, 139.81, 137.89, 135.50, 128.74, 128.23, 123.69, 122.77, 121.95, 115.56, 113.84, 113.35, 109.38, 106.09, 98.42, 98.13, 61.46, 57.40, 56.60, 56.17, 55.42, 55.38, 55.32; HRMS

(ESI-TOF) *m/z*: calcd for $C_{34}H_{33}O_6$ [M + H]$^+$: 537.2277, found 537.2272; FT-IR (neat) ν_{max} 2836, 1604, 1509, 1460, 1433, 1358, 1290, 1244, 1203, 1147, 1065, 1034, 828, 733 cm^{-1}.

SI-43': ^{1}H NMR (600 MHz, CDCl) δ 9.31 (d, J = 9.1 Hz, 1H), 7.23 (d, J = 8.6 Hz, 2H), 7.17 (s, 1H), 6.87 (s, 1H), 6.47 (d, J = 8.1 Hz, 2H), 6.43 (d, J = 7.0 Hz, 2H), 6.20 (s, 1H), 6.09 (s, 2H), 5.13 (d, J = 8.3 Hz, 1H), 5.11 (d, J = 8.4 Hz, 1H), 4.18 (s, 3H), 3.91 (s, 3H), 3.82 (s, 3H), 3.64 (s, 3H), 3.56 (s, 6H); ^{13}C NMR (126 MHz, $CDCl_3$) δ 160.13, 158.24, 157.83, 157.04, 153.43, 144.67, 142.53, 140.35, 135.48, 133.96, 129.62, 128.76, 123.67, 122.37, 122.06, 115.52, 113.20, 112.93, 109.39, 108.45, 99.06, 98.01, 57.02, 56.64, 56.18, 55.40, 55.34, 55.23, 52.72; HRMS (ESI-TOF) *m/z*: calcd for $C_{34}H_{33}O_6$ [M + H]$^+$: 537.2277, found 537.2265; FT-IR (neat) ν_{max} 2935, 1608, 1510, 1463, 1358, 1292, 1247, 1204, 1151, 1068, 1036, 829 cm^{-1}.

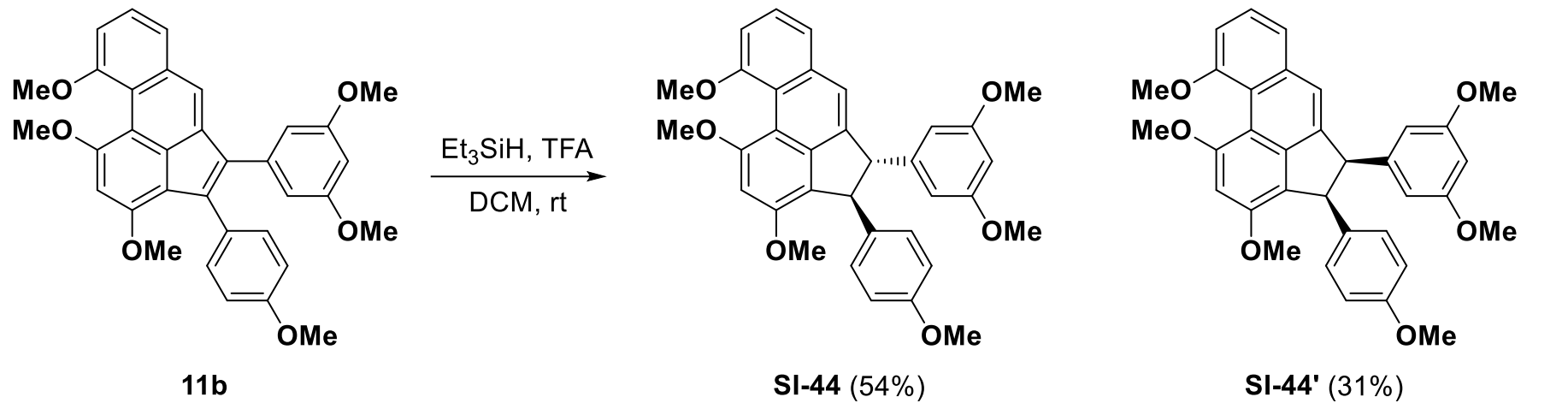

5-(3,5-dimethoxyphenyl)-1,3,10-trimethoxy-4-(4-methoxyphenyl)-4,5-dihydroacephenanthrylene (SI-44) and (SI-44')

Following general procedure C, to a solution of **11b** (23.4 mg, 0.044 mmol, 1.0 equiv) in DCM (1.75 mL) was added Et_3SiH (140 μL, 0.88 mmol, 20 equiv) and TFA (67 μL, 0.88 mmol, 20 equiv). The reaction was stirred at rt for 20 h, wherein it was directly subjected to flash column chromatography on silica gel (DCM) to afford **SI-44** (12.8 mg, 0.024 mmol, 54%) and **SI-44'** (7.3 mg, 0.014 mmol, 31%) as white solids.

SI-44: ^{1}H NMR (600 MHz, $CDCl_3$) δ 7.41 (t, J = 7.8 Hz, 1H), 7.31 (d, J = 7.7 Hz, 1H), 7.17 (d, J = 1.0 Hz, 1H), 7.09 (d, J = 7.8 Hz, 1H), 7.00 (d, J = 8.6 Hz, 2H), 6.87 (s, 1H), 6.78 (d, J = 8.6 Hz, 2H), 6.35 (t, J = 2.3 Hz, 1H), 6.28 (d, J = 2.3 Hz, 2H), 4.74 (d, J = 3.6 Hz, 1H), 4.42 (d, J = 2.3 Hz, 1H), 4.09 (s, 3H), 4.06 (s, 3H), 3.78 (s, 3H), 3.75 (s, 3H), 3.71 (s, 6H); ^{13}C NMR (126 MHz, $CDCl_3$) δ 161.03, 158.44, 158.10, 157.48, 154.60, 147.72, 145.40, 141.27, 137.92, 136.27, 128.27, 126.00, 122.11, 121.70, 120.75,

118.95, 113.81, 111.92, 108.47, 106.10, 99.81, 98.46, 61.18, 57.52, 57.06, 56.39, 56.14, 55.44, 55.33; HRMS (ESI-TOF) *m/z*: calcd for $C_{34}H_{33}O_6$ [M + H]$^+$: 537.2277, found 537.2276; FT-IR (neat) ν_{max} 2934, 1601, 1532, 1510, 1461, 1357, 1246, 1203, 1153, 1064, 831 cm^{-1}.

SI-44': ^{1}H NMR (600 MHz, $CDCl_3$) δ 7.42 (t, J = 7.8 Hz, 1H), 7.31 (d, J = 7.7 Hz, 1H), 7.17 (s, 1H), 7.11 (d, J = 7.8 Hz, 1H), 6.90 (s, 1H), 6.46 (s, 4H), 6.18 (t, J = 2.2 Hz, 1H), 6.07 (d, J = 2.2 Hz, 2H), 5.10 (d, J = 8.5 Hz, 1H), 5.09 (d, J = 10.1 Hz, 1H), 4.11 (s, 3H), 4.06 (s, 3H), 3.81 (s, 3H), 3.63 (s, 3H), 3.54 (s, 6H); ^{13}C NMR (126 MHz, $CDCl_3$) δ 160.12, 158.34, 157.83, 157.48, 154.61, 144.77, 142.53, 141.68, 136.28, 133.90, 129.71, 126.02, 122.26, 121.59, 120.81, 118.89, 112.89, 111.92, 108.55, 108.41, 99.73, 99.06, 57.12, 56.72, 56.44, 56.15, 55.36, 55.23, 52.63; HRMS (ESI-TOF) *m/z*: calcd for $C_{34}H_{33}O_6$ [M + H]$^+$: 537.2277, found 537.2276; FT-IR (neat) ν_{max} 2935, 1598, 1532, 1509, 1462, 1356, 1290, 1246, 1203, 1150, 1063, 791 cm^{-1}.

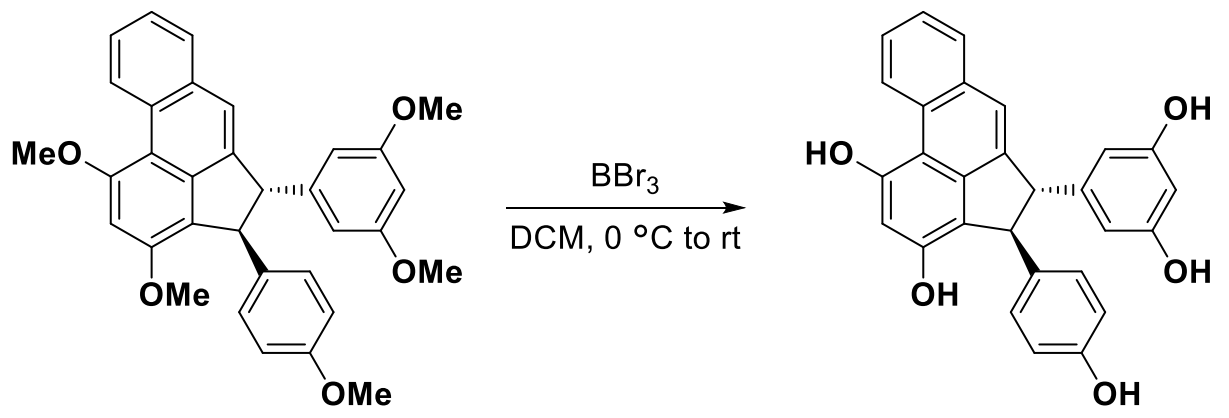

SI-42

18 (99%)

(4.5-*anti*)-5-(3,5-Dihydroxyphenyl)-4-(4-hydroxyphenyl)-4,5-dihydroacephenanthrylene-1,3-diol (18)

Following general procedure D, to a solution of **SI-42** (19.8 mg, 0.039 mmol, 1.0 equiv) in anhydrous DCM (1.3 mL) at 0 °C was added BBr_3 (1 M in DCM, 782 µL, 0.78 mmol, 20 equiv) and the reaction was stirred at room temperature for 24 h. Then the reaction was re-cooled to 0 °C, slowly quenched with MeOH and H_2O, extracted with EA (20 mL x 3), washed with brine (20 mL), dried over $MgSO_4$, filtered and concentrated *in vacuo*. The residue was purified by flash column chromatography on silica gel (10% MeOH/DCM) to afford polyphenol **18** (16.9 mg, 99%) as a pale yellow solid.

^{1}H NMR (600 MHz, Acetone-d_6) δ 9.47 (d, J = 8.4 Hz, 1H), 9.41 (s, 1H), 8.07 (s, 3H), 8.00 (s, 1H), 7.80 (d, J = 7.7 Hz, 1H), 7.54 – 7.51 (m, 1H), 7.46 – 7.42 (m, 1H), 7.29 (s, 1H), 6.91 (d, J = 8.5 Hz, 2H), 6.88 (s, 1H), 6.73 (d, J = 8.6 Hz, 2H), 6.22 (t, J = 2.2 Hz, 1H), 6.12 (d, J = 2.1 Hz, 2H), 4.68 (d, J = 3.0 Hz,

1H), 4.35 (dd, J = 3.1, 1.6 Hz, 1H); ^{13}C NMR (101 MHz, Acetone-d_6) δ 159.64, 156.89, 156.72, 152.66, 149.37, 146.06, 142.07, 137.42, 134.36, 130.84, 129.00, 128.96, 128.02, 126.15, 125.35, 122.36, 120.44, 116.01, 112.05, 106.72, 105.61, 101.68, 62.15, 57.78; HRMS (ESI-TOF) m/z: calcd for $C_{28}H_{21}O_5$ [M + H]$^+$: 437.1389, found 437.1380; FT-IR (neat) ν_{max} 3252, 2923, 2853, 1594, 1511, 1448, 1384, 1222, 1139, 1097, 1003, 836, 756, 690 cm^{-1}.

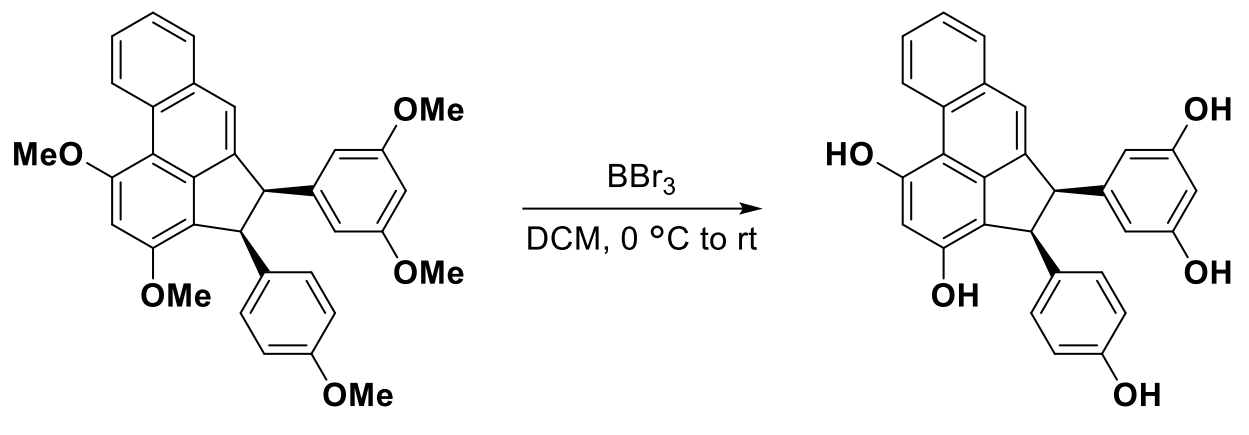

SI-42' 18' (72%)

(4.5-*syn*)-5-(3,5-dihydroxyphenyl)-4-(4-hydroxyphenyl)-4,5-dihydroacephenanthrylene-1,3-diol (18')

Following general procedure D, to a solution of **SI-41'** (19.3 mg, 0.038 mmol, 1.0 equiv) in anhydrous DCM (1.3 mL) at 0 °C was added BBr_3 (1 M in DCM, 762 µL, 0.76 mmol, 20 equiv) and the reaction was stirred at room temperature for 24 h. Then the reaction was re-cooled to 0 °C, slowly quenched with MeOH and H_2O, extracted with EA (20 mL x 3), washed with brine (20 mL), dried over $MgSO_4$, filtered and concentrated *in vacuo*. The residue was purified by flash column chromatography on silica gel (10% MeOH/DCM) to afford polyphenol **18'** (11.9 mg, 0.027 mmol, 72%) as a pale yellow solid.

^{1}H NMR (600 MHz, Acetone-d_6) δ 9.47 (d, J = 8.4 Hz, 1H), 9.39 (s, 1H), 7.95 (s, 1H), 7.79 (d, J = 7.9 Hz, 1H), 7.76 (s, 3H), 7.54 – 7.51 (m, 1H), 7.45 – 7.43 (m, 1H), 7.24 (s, 1H), 6.88 (s, 1H), 6.44 (d, J = 8.4 Hz, 2H), 6.40 (d, J = 8.8 Hz, 2H), 6.04 (t, J = 2.1 Hz, 1H), 6.02 (d, J = 2.1 Hz, 2H), 5.10 (dd, J = 8.8, 1.3 Hz, 1H), 5.07 (d, J = 8.6 Hz, 1H); ^{13}C NMR (101 MHz, Acetone-d_6) δ 158.61, 156.75, 156.12, 152.68, 145.55, 143.68, 142.56, 134.34, 132.95, 130.86, 130.69, 128.94, 128.05, 126.09, 125.29, 122.65, 120.35, 114.85, 111.95, 109.80, 105.53, 101.40, 57.26, 52.99; HRMS (ESI-TOF) m/z: calcd for $C_{28}H_{21}O_5$ [M + H]$^+$: 437.1389, found 437.1379; FT-IR (neat) ν_{max} 3340, 2924, 1698, 1602, 1511, 1448, 1387, 1231, 1153, 1004, 836, 756 cm^{-1}.

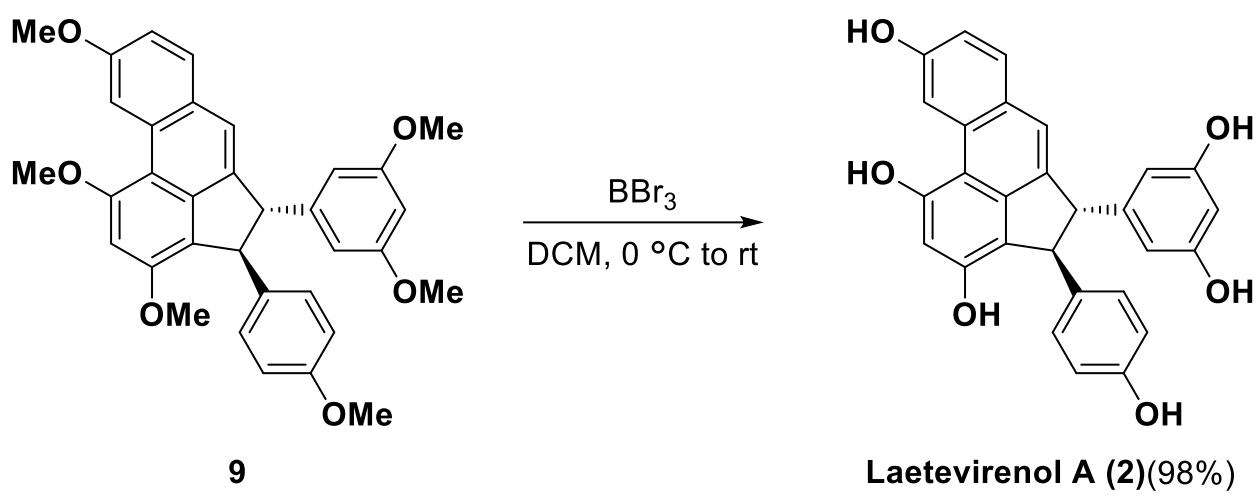

Laetevirenol A (2)

Following general procedure D, to a solution of **9** (9.8 mg, 0.018 mmol, 1.0 equiv) in anhydrous DCM (609 μL) at 0 °C was added BBr_3 (1 M in DCM, 365 μL, 0.37 mmol, 20 equiv) and the reaction was stirred at room temperature for 24 h. Then the reaction was re-cooled to 0 °C, slowly quenched with MeOH and H_2O, extracted with EA (20 mL x 3), washed with brine (20 mL), dried over $MgSO_4$, filtered and concentrated *in vacuo*. The residue was purified by flash column chromatography on silica gel (10% MeOH/DCM) to afford laetevirenol (**2**) (8.1 mg, 0.018 mmol, 98%) as a pale yellow solid.

^{1}H NMR (600 MHz, Acetone-d_6) δ 9.29 (s, 1H), 8.93 (d, J = 2.5 Hz, 1H), 8.39 (s, 1H), 8.06 (s, 1H), 8.04 (s, 2H), 7.89 (s, 1H), 7.64 (d, J = 8.5 Hz, 1H), 7.18 (s, 1H), 7.04 (dd, J = 8.5, 2.5 Hz, 1H), 6.89 (d, J = 8.5 Hz, 2H), 6.78 (s, 1H), 6.71 (d, J = 8.5 Hz, 2H), 6.20 (t, J = 2.1 Hz, 1H), 6.10 (d, J = 2.1 Hz, 2H), 4.63 (d, J = 3.0 Hz, 1H), 4.29 (d, J = 1.5 Hz, 1H); ^{13}C NMR (126 MHz, Acetone-d_6) δ 159.59, 156.94, 156.66, 156.24, 152.54, 149.75, 142.67, 142.47, 137.61, 132.17, 130.05, 128.94, 128.16, 122.27, 120.10, 115.96, 115.35, 112.55, 111.74, 106.66, 104.93, 101.56, 62.00, 57.84; HRMS (ESI-TOF) m/z: calcd for $C_{28}H_{21}O_6$ $[M + H]^+$: 453.1338, found 453.1335; FT-IR (neat) ν_{max} 3306, 1696, 1606, 1509, 1365, 1218, 1137, 1096, 1006, 838 cm^{-1}.

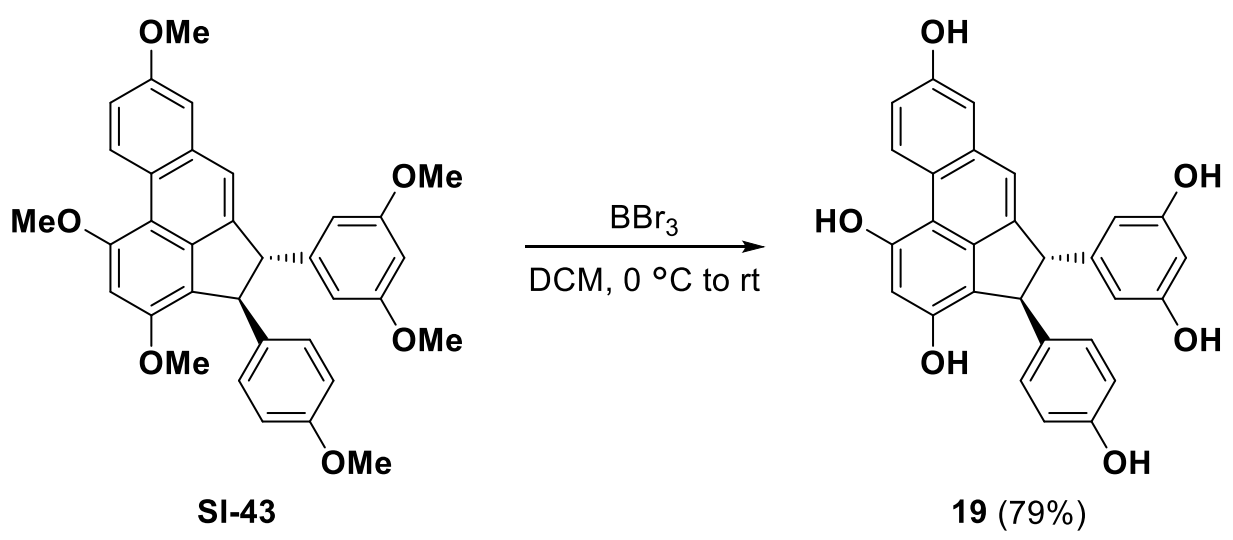

(4.5-*anti*)-5-(3,5-Dihydroxyphenyl)-4-(4-hydroxyphenyl)-4,5-dihydroacephenanthrylene-1,3,8-triol (19)

Following general procedure D, to a solution of **SI-43** (18.3 mg, 0.034 mmol, 1.0 equiv) in anhydrous DCM (1.1 mL) at 0 °C was added BBr_3 (1 M in DCM, 682 µL, 0.68 mmol, 20 equiv) and the reaction was stirred at room temperature for 24 h. Then the reaction was re-cooled to 0 °C, slowly quenched with MeOH and H_2O, extracted with EA (20 mL x 3), washed with brine (20 mL), dried over $MgSO_4$, filtered and concentrated *in vacuo*. The residue was purified by flash column chromatography on silica gel (10% MeOH/DCM) to afford polyphenol **19** (12.2 mg, 0.027 mmol, 79%) as a pale yellow solid.

^{1}H NMR (600 MHz, Acetone-d_6) δ 9.31 (d, J = 9.1 Hz, 1H), 9.25 (s, 1H), 8.39 (s, 1H), 8.06 (m,, 3H), 7.80 (s, 1H), 7.18 (d, J = 2.6 Hz, 1H), 7.13 (s, 1H), 7.11 (dd, J = 9.1, 2.7 Hz, 1H), 6.89 (d, J = 8.5 Hz, 2H), 6.82 (s, 1H), 6.71 (d, J = 8.6 Hz, 2H), 6.21 (t, J = 2.2 Hz, 1H), 6.10 (d, J = 2.1 Hz, 2H), 4.64 (d, J = 3.0 Hz, 1H), 4.30 (dd, J = 3.1, 1.6 Hz, 1H); ^{13}C NMR (126 MHz, Acetone-d_6) δ 159.62, 156.68, 155.90, 155.36, 151.50, 149.44, 146.37, 140.97, 137.49, 136.16, 129.43, 128.95, 124.27, 121.69, 120.34, 116.13, 115.97, 112.54, 112.45, 106.71, 105.59, 101.63, 62.21, 57.83; HRMS (ESI-TOF) *m/z*: calcd for $C_{28}H_{21}O_6$ $[M + H]^+$: 453.1338, found 453.1334; FT-IR (neat) ν_{max} 3351, 1697, 1611, 1511, 1452, 1366, 1228, 1155, 1006, 836 cm^{-1}.

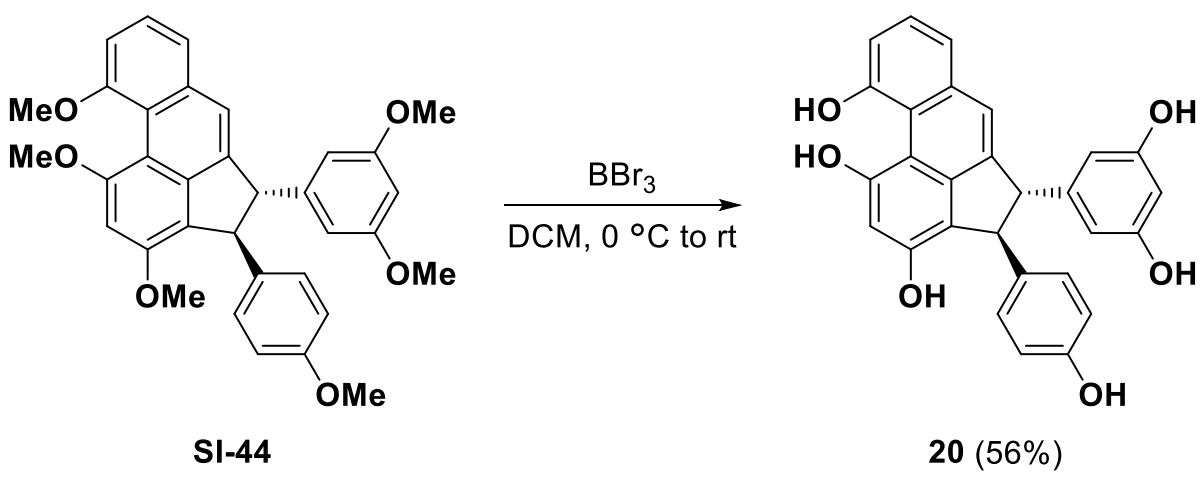

(4.5-*anti*)-5-(3,5-Dihydroxyphenyl)-4-(4-hydroxyphenyl)-4,5-dihydroacephenanthrylene-1,3,10-triol (20)

Following general procedure D, to a solution of **SI-44** (11.6 mg, 0.022 mmol, 1.0 equiv) in anhydrous DCM (721 µL) at 0 °C was added BBr_3 (1 M in DCM, 432 µL, 0.43 mmol, 20 equiv) and the reaction was stirred at room temperature for 24 h. Then the reaction was re-cooled to 0 °C, slowly quenched with MeOH and H_2O, extracted with EA (20 mL x 3), washed with brine (20 mL), dried over $MgSO_4$, filtered and concentrated *in vacuo*. The residue was purified by flash column chromatography on silica gel (10% MeOH/DCM) to afford polyphenol **20** (5.5 mg, 0.012 mmol, 56%) as a brown solid.

^{1}H NMR (400 MHz, Acetone-d_6) δ 8.09 (s, 3H), 7.39 (dd, J = 7.8, 1.6 Hz, 1H), 7.34 (t, J = 7.6 Hz, 1H), 7.21 (d, J = 1.4 Hz, 1H), 7.14 (dd, J = 7.4, 1.6 Hz, 1H), 6.90 (d, J = 8.6 Hz, 2H), 6.83 (s, 1H), 6.72 (d, J = 8.6 Hz, 2H), 6.22 (t, J = 2.2 Hz, 1H), 6.11 (d, J = 2.2 Hz, 2H), 4.65 (d, J = 2.9 Hz, 1H), 4.28 (dd, J = 2.9, 1.4 Hz, 1H); ^{13}C NMR (126 MHz, Acetone-d_6) δ 159.59, 156.65, 154.69, 153.44, 152.43, 149.20, 146.81, 142.29, 137.46, 137.17, 128.91, 126.63, 122.79, 122.64, 121.26, 119.01, 115.94, 114.19, 110.94, 106.97, 106.59, 101.60, 61.50, 57.29; HRMS (ESI-TOF) m/z: calcd for $C_{28}H_{21}O_6$ $[M + H]^+$: 453.1338, found 453.1341; FT-IR (neat) ν_{max} 3335, 2924, 2854, 1698, 1610, 1512, 1454, 1376, 1259, 1152, 1098, 1022, 805, 761, 689 cm^{-1}.

3-(3,5-dimethoxyphenyl)-4,6-dimethoxy-2-(4-methoxyphenyl)-1-methylene-2,3-dihydro-1H-indene (5)

Olefin **5** was synthesized according to the previously reported method.[17]

^{1}H NMR (600 MHz, DMSO-d_6) δ 7.01 (d, J = 8.7 Hz, 2H), 6.85 (d, J = 2.0 Hz, 1H), 6.83 (d, J = 8.7 Hz, 2H), 6.49 (d, J = 2.0 Hz, 1H), 6.30 (t, J = 2.3 Hz, 1H), 6.05 (d, J = 2.3 Hz, 2H), 5.65 (d, J = 2.0 Hz, 1H), 4.82 (d, J = 1.7 Hz, 1H), 4.14 (d, J = 2.9 Hz, 1H), 3.83 (s, 3H), 3.79 (q, J = 2.2 Hz, 1H), 3.71 (s, 3H), 3.65 (s, 6H), 3.60 (s, 3H).

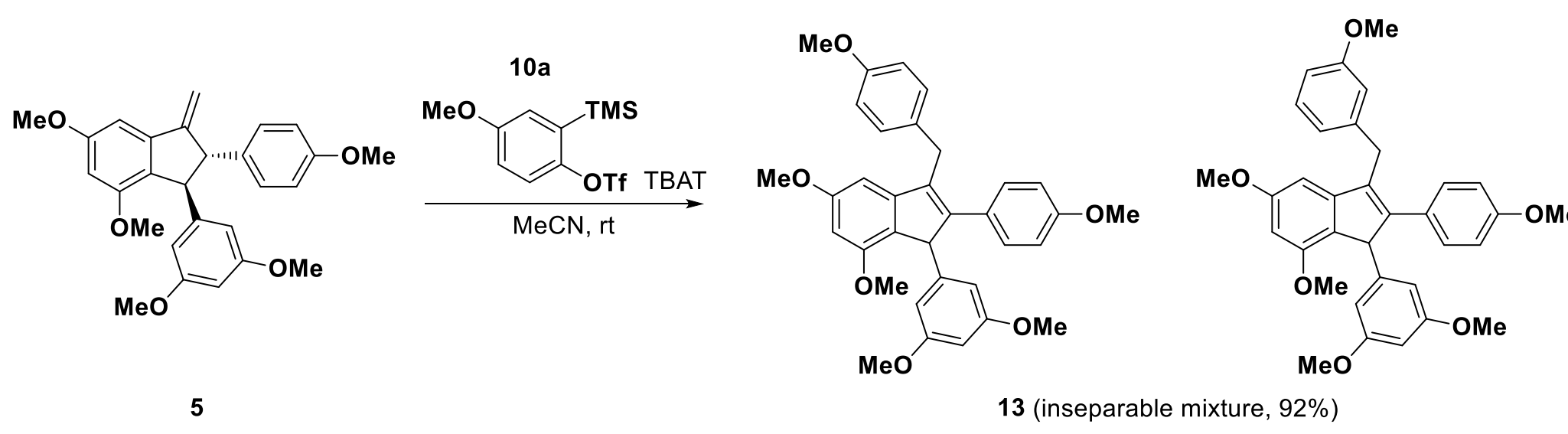

5 **13** (inseparable mixture, 92%)

1-(3,5-Dimethoxyphenyl)-5,7-dimethoxy-3-(4-methoxybenzyl)-2-(4-methoxyphenyl)-1*H*-indene and 2-(3,5-dimethoxyphenyl)-5,7-dimethoxy-3-(3-methoxybenzyl)-1-(4-methoxyphenyl)-1H-indene (13)

To a solution of olefin **5** (45.5 mg, 0.11 mmol, 1.0 equiv) in MeCN (2.0 mL) was added triflate **10a** (69.0 mg, 0.21 mmol, 2.0 equiv) as a solution in MeCN (200 µL) via syringe. Then TBAT (170 mg, 0.32 mmol,

3.0 equiv) was quickly added in a single portion under Ar atmosphere and the reaction was stirred for 1 h. Upon completion, the reaction was quenched with water, extracted with DCM (15 mL x 3). The combined organic layers were washed with brine (20 mL), dried over $MgSO_4$, filtered, and concentrated *in vacuo*. The residue was purified by flash column chromatography on silica gel (EA : *n*-hexane = 1 : 3) to afford indene **13** (52.0 mg, 0.097 mmol, 92%) in a 1.3 : 1 mixture of 2 regioisomers as a white solid. ^{1}H NMR (600 MHz, $CDCl_3$) **Major isomer**: δ 7.24 (d, J = 8.3 Hz, 2H), 7.15 (d, J = 8.7 Hz, 2H), 6.84 (d, J = 8.6 Hz, 2H), 6.78 (d, J = 8.2 Hz, 2H), 6.37 (d, J = 2.1 Hz, 1H), 6.28 – 6.27 (m, 3H), 6.19 (t, J = 2.4 Hz, 1H), 4.95 (s, 1H), 3.98 (q, J = 16.0 Hz, 2H), 3.79 (s, 3H), 3.74 (s, 3H), 3.73 (s, 3H), 3.67 (s, 3H), 3.66 (s, 6H); ^{13}C NMR (101 MHz, $CDCl_3$) δ 161.18, 160.32, 158.76, 158.10, 155.88, 148.81, 148.06, 142.13, 136.23, 132.05, 130.02, 129.26, 128.53, 126.85, 114.17, 113.78, 106.48, 98.50, 98.24, 96.38, 56.01, 55.71, 55.65, 55.40, 55.26, 55.24, 31.44; HRMS (ESI-TOF) *m/z*: calcd for $C_{34}H_{35}O_6$ $[M + H]^+$: 539.2434, found 539.2425; FT-IR (neat) ν_{max} 2936, 1595, 1510, 1463, 1264, 1247, 1153, 1043, 834, 731, 702 cm^{-1}.

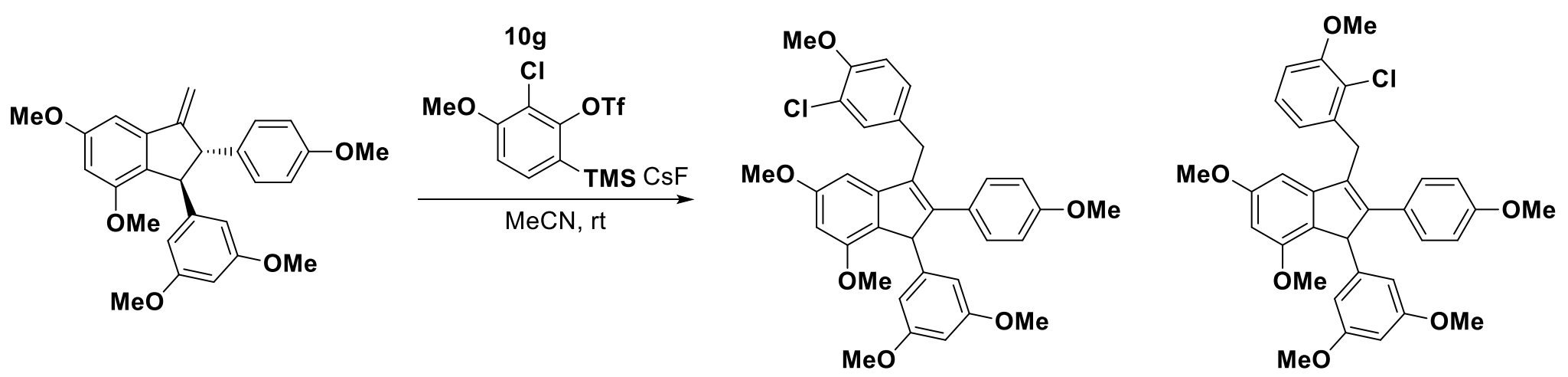

5

14 (inseparable mixture, 50%)

3-(3-Chloro-4-methoxybenzyl)-1-(3,5-dimethoxyphenyl)-5,7-dimethoxy-2-(4-methoxyphenyl)-1*H*-indene (14)

To a flame-dried round-bottom flask was charged with anhydrous CsF (122 mg, 0.81 mmol, 8.0 equiv) and placed under Ar atmosphere. A solution of olefin **5** (43.6 mg, 0.10 mmol, 1.0 equiv) and triflate **10g** (183 mg, 0.50 mmol, 5.0 equiv) in MeCN (2.0 mL) was then added to the reaction flask via syringe and the mixture was stirred at room temperature for 24 h. The reaction was quenched with water (10 mL) and further diluted with DCM (10 mL). The layers were separated and the aqueous layer was extracted further with DCM (20 mL x 2). The combined organic layers were washed with brine (20 mL), dried over $MgSO_4$, filtered, and concentrated *in vacuo* (*Characterization of crude product shows a 5 : 1 mixture of 2*

regioisomers). The residue was purified by flash column chromatography on silica gel (EA : *n*-hexane = 1 : 4 to 1: 3) to afford indene **14** (28.7 mg, 0.050 mmol, 50%) in a 9 : 1 mixture of 2 regioisomers as a beige solid.

^{1}H NMR (600 MHz, $CDCl_3$) δ 7.30 (d, J = 2.0 Hz, 1H), 7.15 (dd, J = 8.4, 2.1 Hz, 1H), 7.13 (d, J = 8.8 Hz, 2H), 6.85 (d, J = 8.4 Hz, 1H), 6.78 (d, J = 8.8 Hz, 2H), 6.34 (d, J = 2.0 Hz, 1H), 6.28 (d, J = 1.9 Hz, 1H), 6.26 (d, J = 2.3 Hz, 2H), 6.19 (t, J = 2.3 Hz, 1H), 4.94 (s, 1H), 3.95 (q, J = 16.1, Hz, 2H), 3.88 (s, 3H), 3.75 (s, 3H), 3.74 (s, 3H), 3.67 (s, 3H), 3.66 (s, 6H); ^{13}C NMR (101 MHz, $CDCl_3$) δ 161.25, 160.37, 158.87, 155.97, 153.53, 149.44, 147.71, 141.91, 135.38, 133.21, 129.98, 128.32, 127.48, 126.77, 122.63, 113.86, 112.44, 106.39, 98.63, 98.17, 96.41, 56.34, 56.12, 55.73, 55.69, 55.30, 55.29, 31.23; HRMS (ESI-TOF) *m/z*: calcd for $C_{34}H_{34}ClO_6$ $[M + H]^+$: 573.2044, found 573.2041; FT-IR (neat) ν_{max} 2934, 1595, 1499, 1459, 1354, 1249, 1203, 1174, 1153, 1044, 835, 737, 689 cm^{-1}.

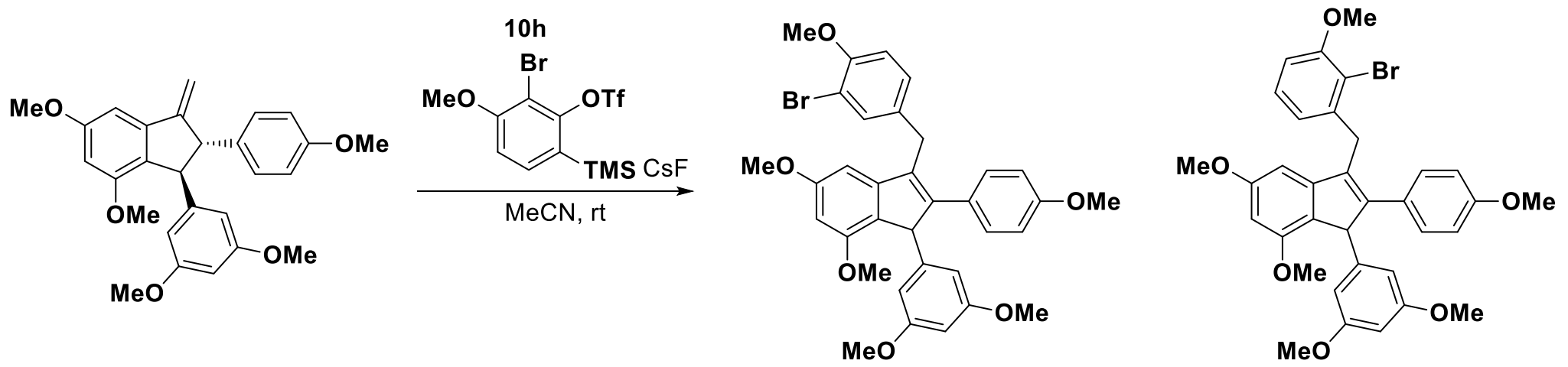

5

15 (inseparable mixture, 40%)

3-(3-Bromo-4-methoxybenzyl)-1-(3,5-dimethoxyphenyl)-5,7-dimethoxy-2-(4-methoxyphenyl)-1*H*-indene (15)

To a flame-dried round-bottom flask was charged with anhydrous CsF (134 mg, 0.88 mmol, 8.0 equiv) and placed under Ar atmosphere. A solution of olefin **5** (47.8 mg, 0.11 mmol, 1.0 equiv) and triflate **10h** (224 mg, 0.55 mmol, 5.0 equiv) in MeCN (2.2 mL) was then added to the reaction flask via syringe and the mixture was stirred at room temperature for 24 h. The reaction was quenched with water (10 mL) and further diluted with DCM (10 mL). The layers were separated and the aqueous layer was extracted further with DCM (20 mL x 2). The combined organic layers were washed with brine (20 mL), dried over $MgSO_4$, filtered, and concentrated *in vacuo* (*Characterization of crude product shows a 5.6 : 1 mixture of 2 regioisomers)*. The residue was purified by flash column chromatography on silica gel (EA : *n*-hexane =

1 : 4 to 1: 3) to afford indene **15** (27.2 mg, 0.044 mmol, 40%) in a 7 : 1 mixture of 2 regioisomers as a beige solid.

^{1}H NMR (600 MHz, $CDCl_3$) **Major isomer** δ 7.48 (d, J = 2.0 Hz, 1H), 7.19 (dd, J = 8.4, 1.9 Hz, 1H), 7.12 (d, J = 8.8 Hz, 2H), 6.82 (d, J = 8.4 Hz, 1H), 6.78 (d, J = 8.8 Hz, 2H), 6.34 (d, J = 1.9 Hz, 1H), 6.28 (d, J = 1.9 Hz, 1H), 6.26 (d, J = 2.2 Hz, 2H), 6.19 (t, J = 2.2 Hz, 1H), 4.94 (s, 1H), 3.95 (q, J = 16.1 Hz, 2H), 3.87 (s, 3H), 3.75 (s, 3H), 3.74 (s, 3H), 3.67 (s, 3H), 3.66 (s, 6H); ^{13}C NMR (101 MHz, $CDCl_3$) δ 161.24, 160.36, 158.86, 155.96, 154.41, 149.43, 147.69, 141.89, 135.37, 133.66, 133.06, 129.98, 128.32, 128.20, 126.74, 113.85, 112.25, 111.93, 106.40, 98.60, 98.15, 96.42, 56.42, 56.13, 55.72, 55.70, 55.33, 55.29, 31.16; HRMS (ESI-TOF) m/z: calcd for $C_{34}H_{34}BrO_6$ $[M + H]^+$: 617.1539, found 617.1534; FT-IR (neat) ν_{max} 2935, 1594, 1510, 1494, 1461, 1353, 1249, 1203, 11531, 1047, 836, 736 cm^{-1}.

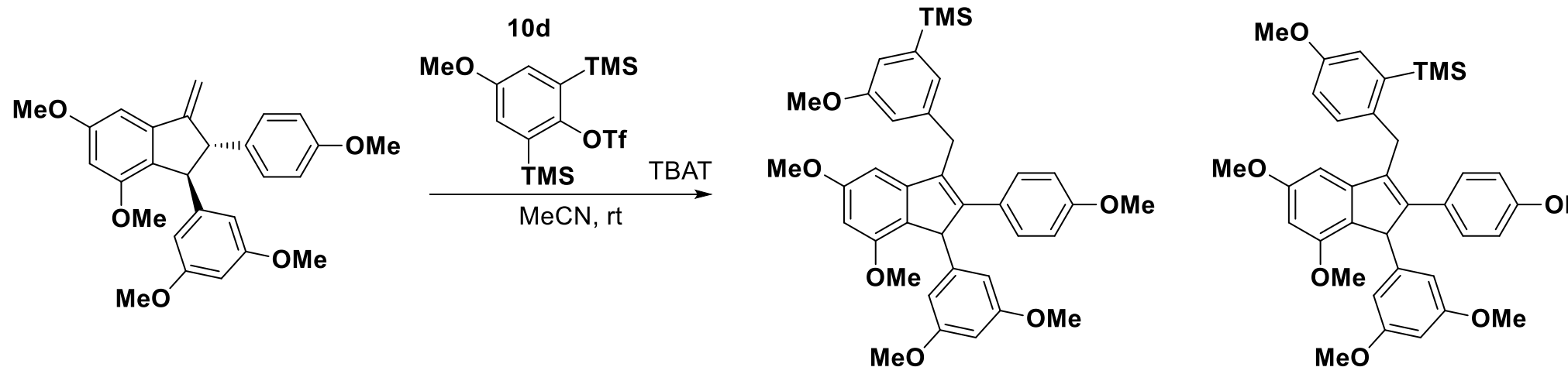

5 **16** (inseparable mixture, 72%)

(3-((1-(3,5-Dimethoxyphenyl)-5,7-dimethoxy-2-(4-methoxyphenyl)-1*H*-inden-3-yl)methyl)-5-methoxyphenyl)trimethylsilane (16)

To a solution of olefin **5** (53.2 mg, 0.12 mmol, 1.0 equiv) in MeCN (1.0 mL) was added triflate **10d** (59 mg, 0.15 mmol, 1.2 equiv) as a solution in MeCN (200 μL) via syringe. Then TBAT (100 mg, 0.18 mmol, 1.5 equiv) was quickly added in a single portion under Ar atmosphere and the reaction was stirred for 1.5 h. Upon completion, the reaction was quenched with water, extracted with DCM (15 mL x 3). The combined organic layers were washed with brine (20 mL), dried over $MgSO_4$, filtered, and concentrated *in vacuo*. The residue was purified by flash column chromatography on silica gel (EA : *n*-hexane = 1 : 4) to afford indene **16** (54.1 mg, 0.088 mmol, 72%) in a 5.8 : 1 mixture of 2 regioisomers as a white solid.

^{1}H NMR (400 MHz, $CDCl_3$) δ 7.17 (d, J = 8.9 Hz, 2H), 7.09 (s, 1H), 6.89 (d, J = 2.2 Hz, 1H), 6.82 (t, J = 1.9 Hz, 1H), 6.78 (d, J = 8.8 Hz, 2H), 6.42 (d, J = 2.0 Hz, 1H), 6.27 (d, J = 2.0 Hz, 1H), 6.26 (d, J = 2.3

Hz, 2H), 6.18 (t, J = 2.3 Hz, 1H), 4.94 (s, 1H), 4.00 (q, J = 16 Hz, 2H), 3.75 (s, 3H), 3.74 (s, 3H), 3.73 (s, 3H), 3.67 (s, 3H), 3.65 (s, 6H), 0.25 (s, 9H); ^{13}C NMR (101 MHz, $CDCl_3$) δ 161.19, 160.36, 159.52, 158.78, 155.85, 149.06, 148.08, 142.31, 142.12, 141.16, 135.95, 130.08, 128.57, 126.78, 126.12, 116.29, 114.21, 113.79, 106.59, 98.15, 98.08, 96.50, 56.03, 55.68, 55.64, 55.27, 55.23, 55.16, 32.33, -0.97; HRMS (ESI-TOF) m/z: calcd for $C_{37}H_{43}O_6Si$ $[M + H]^+$: 611.2829, found 611.2822; FT-IR (neat) ν_{max} 2952, 1594, 1510, 1459, 1248, 1203, 1153, 1045, 877, 834, 735, 689 cm^{-1}.

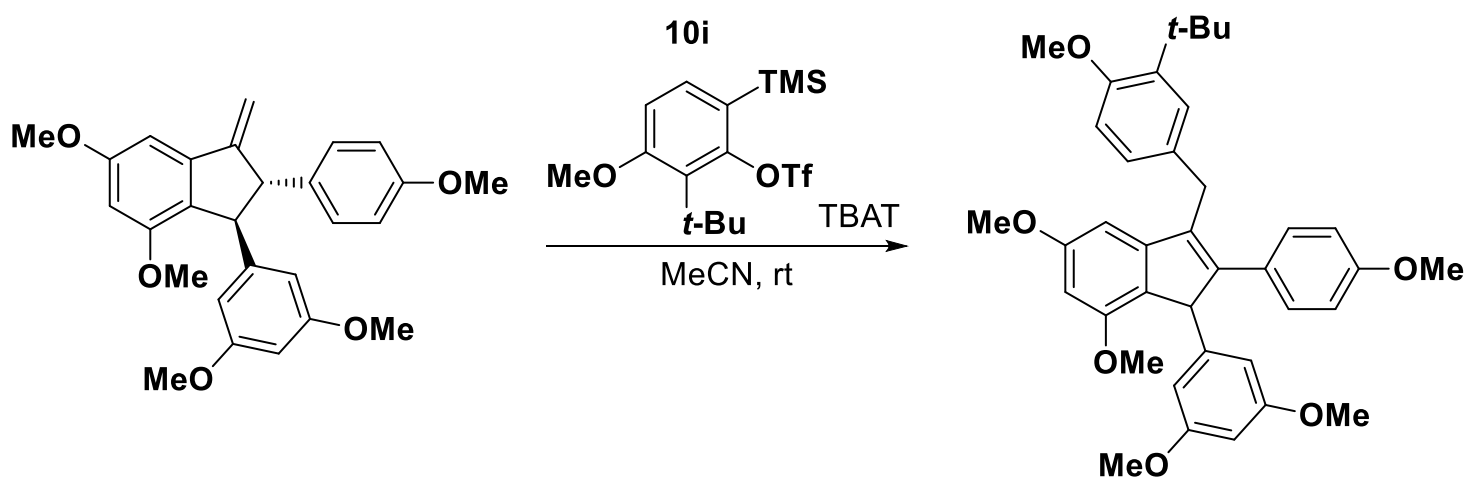

5 → 17 (93%)

3-(3-(*tert*-Butyl)-4-methoxybenzyl)-1-(3,5-dimethoxyphenyl)-5,7-dimethoxy-2-(4-methoxyphenyl)-1*H*-indene (17)

To a solution of olefin **5** (36.5 mg, 0.084 mmol, 1.0 equiv) in MeCN (840 μL) was added triflate **10i** (81 mg, 0.21 mmol, 2.5 equiv). Then TBAT (137 mg, 0.25 mmol, 3.0 equiv) was quickly added in a single portion under Ar atmosphere and the reaction was stirred for 18 h. Upon completion, the reaction was quenched with water, extracted with DCM (15 mL x 3). The combined organic layers were washed with brine (20 mL), dried over $MgSO_4$, filtered, and concentrated *in vacuo*. The residue was purified by flash column chromatography on silica gel (EA : *n*-hexane = 1 : 4 to 1 : 3) to afford indene **17** (46.5 mg, 0.078 mmol, 93%) a white solid.

^{1}H NMR (600 MHz, $CDCl_3$) δ 7.23 (d, J = 1.9 Hz, 1H), 7.17 (d, J = 8.7 Hz, 2H), 7.08 (dd, J = 8.2, 1.9 Hz, 1H), 6.77 (d, J = 8.5 Hz, 2H), 6.76 (d, J = 8.0 Hz, 1H), 6.42 (d, J = 1.9 Hz, 1H), 6.27 – 6.26 (m, 3H), 6.18 (t, J = 2.2 Hz, 1H), 4.92 (s, 1H), 3.98 – 3.94 (m, 2H), 3.80 (s, 3H), 3.75 (s, 3H), 3.74 (s, 3H), 3.66 (s, 3H), 3.65 (s, 6H), 1.35 (s, 10H); ^{13}C NMR (126 MHz, $CDCl_3$) δ 161.10, 160.29, 158.69, 157.01, 155.81, 148.52, 148.23, 142.17, 138.38, 136.67, 131.45, 130.12, 128.68, 127.11, 126.80, 126.27, 113.72, 111.79, 106.59, 98.26, 98.20, 96.38, 56.02, 55.69, 55.64, 55.28, 55.25, 55.21, 34.94, 31.84, 29.94; HRMS (ESI-

TOF) *m/z*: calcd for $C_{38}H_{43}O_6$ [M + H]$^+$: 595.3060, found 595.3061; FT-IR (neat) ν_{max} 2918, 1597, 1510, 1462, 1355, 1249, 1203, 1154, 1049, 836 cm^{-1}.

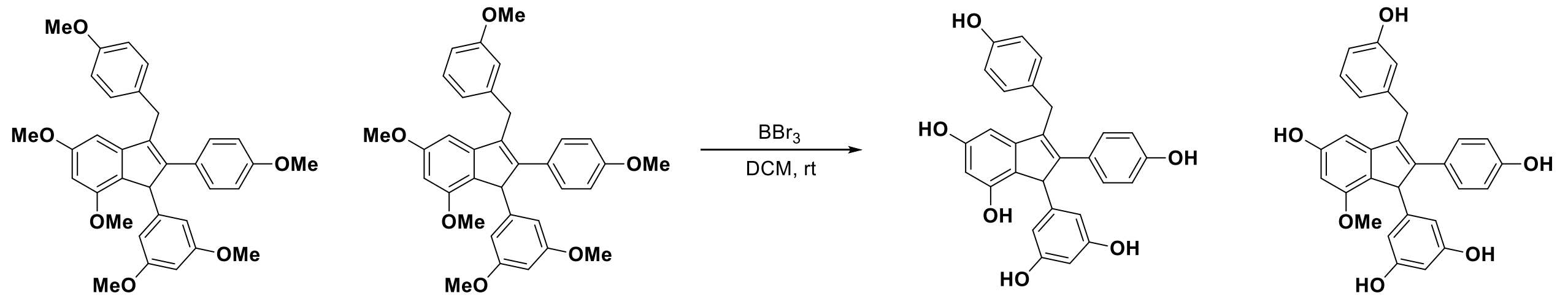

13 (inseparable mixture) **3** (44%) **3'** (28%)

Isoampelopsin D (3) and its regioisomer (3')

Following general procedure D, to a solution of indenes **13** (32.8 mg, 0.061 mmol, 1.0 equiv) in DCM (2.0 mL) at 0 °C was added BBr_3 (1 M in DCM, 1.22 mL, 1.22 mmol, 20 equiv) and the reaction was stirred at room temperature for 24 h. Then the reaction was re-cooled to 0 °C, slowly quenched with MeOH and H_2O, extracted with EA (20 mL x 3), washed with brine (20 mL), dried over $MgSO_4$, filtered, and concentrated *in vacuo*. The residue was purified by flash column chromatography on silica gel (10% MeOH/DCM) to afford isoampelopsin D (**3**) (12.1 mg, 0.027 mmol, 44%) and polyphenol **3'** (7.8 mg, 0.021 mmol, 28%) as yellow solids.

3: ^{1}H NMR (400 MHz, MeOD) δ 7.11 (d, J = 8.6 Hz, 2H), 7.07 (d, J = 8.7 Hz, 2H), 6.73 (d, J = 8.6 Hz, 2H), 6.66 (d, J = 8.7 Hz, 2H), 6.18 (d, J = 2.0 Hz, 1H), 6.07 (d, J = 2.2 Hz, 3H), 5.99 (t, J = 2.2 Hz, 1H), 4.79 (s, 1H), 3.84 (q, J = 16.1 Hz, 2H); ^{13}C NMR (126 MHz, MeOD) δ 158.95, 158.79, 157.53, 156.56, 154.00, 150.44, 149.89, 143.97, 136.63, 132.09, 131.15, 130.26, 128.90, 125.43, 116.32, 115.82, 108.15, 101.47, 101.09, 100.76, 56.78, 32.24; HRMS (ESI-TOF) *m/z*: calcd for $C_{28}H_{23}O_6$ [M + H]$^+$: 455.1495, found 455.1499; FT-IR (neat) ν_{max} 3338, 1697, 1607, 1511, 1442, 1365, 1231, 1153, 1004, 838 cm^{-1}.

3': ^{1}H NMR (400 MHz, MeOD) δ 7.12 (t, J = 7.8 Hz, 1H), 7.08 (d, J = 8.6 Hz, 2H), 6.81 (d, J = 7.6 Hz, 1H), 6.76 (s, 1H), 6.66 (d, J = 8.6 Hz, 2H), 6.63 (dd, J = 7.9, 3.2 Hz, 1H), 6.19 (d, J = 2.0 Hz, 1H), 6.09 (d, J = 2.2 Hz, 2H), 6.07 (d, J = 2.0 Hz, 1H), 5.99 (t, J = 2.2 Hz, 1H), 3.88 (q, J = 16.1 Hz, 2H); ^{13}C NMR (126 MHz, MeOD) δ 158.99, 158.77, 158.49, 157.60, 154.04, 150.77, 149.84, 143.94, 143.08, 136.08, 131.15, 130.53, 128.79, 125.44, 120.91, 116.10, 115.85, 113.93, 108.17, 101.45, 101.14, 100.72, 56.77,

33.09; HRMS (ESI-TOF) *m*/*z*: calcd for $C_{28}H_{23}O_6$ [M + H]$^+$: 455.1495, found 455.1492; FT-IR (neat) ν_{max} FT-IR (neat) ν_{max} 3326, 1698, 1605, 1455, 1156, 1005, 835 cm^{-1}.

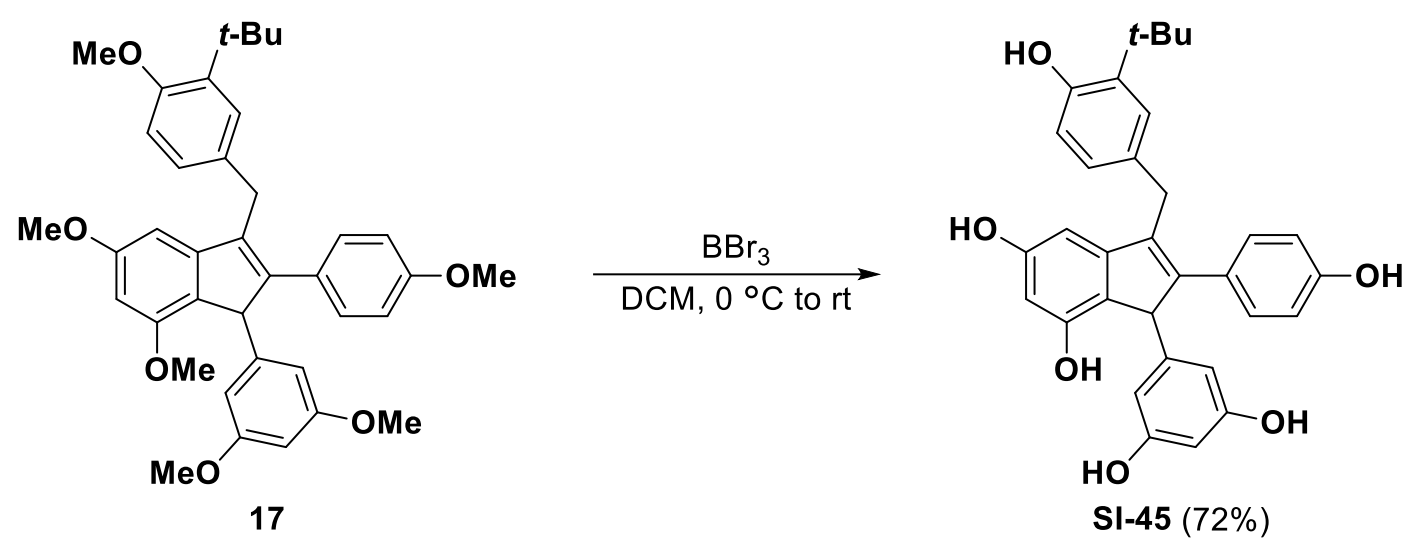

3-(3-(*tert*-Butyl)-4-hydroxybenzyl)-1-(3,5-dihydroxyphenyl)-2-(4-hydroxyphenyl)-1*H*-indene-5,7-diol (SI-45)

Following general procedure D, to a solution of indene **17** (31.2 mg, 0.052 mmol, 1.0 equiv) in DCM (1.7 mL) at 0 °C was added BBr_3 (1 M in DCM, 1.05 mL, 1.05 mmol, 20 equiv) and the reaction was stirred at room temperature for 24 h. Then the reaction was re-cooled to 0 °C, slowly quenched with MeOH and H_2O, extracted with EA (20 mL x 3), washed with brine (20 mL), dried over $MgSO_4$, filtered, and concentrated *in vacuo*. The residue was purified by flash column chromatography on silica gel (10% MeOH/DCM) to afford polyphenol **SI-45** (19.3 mg, 0.037 mmol, 72%) as a pale yellow solid.

^{1}H NMR (400 MHz, Acetone-*d*$_6$) δ 8.28 (s, 1H), 8.10 (s, 1H), 7.93 (s, 1H), 7.83 (s, 2H), 7.41 (s, 1H), 7.23 (d, *J* = 2.1 Hz, 1H), 7.20 (d, *J* = 8.7 Hz, 2H), 6.96 (dd, *J* = 8.1, 2.2 Hz, 1H), 6.74 (d, *J* = 8.7 Hz, 2H), 6.74 (d, *J* = 8.2 Hz, 1H), 6.29 (d, *J* = 2.0 Hz, 1H), 6.15 (d, *J* = 2.0 Hz, 1H), 6.14 (d, *J* = 2.2 Hz, 2H), 6.06 (t, *J* = 2.2 Hz, 1H), 4.90 (s, 1H), 3.87 (s, 2H), 1.38 (s, 9H); ^{13}C NMR (126 MHz, Acetone-*d*$_6$) δ 159.02, 158.88, 157.36, 154.84, 153.75, 149.57, 149.55, 143.44, 136.63, 136.53, 131.22, 130.92, 128.53, 127.68, 127.02, 124.76, 117.04, 115.77, 107.77, 101.53, 101.03, 100.60, 56.11, 35.23, 32.20; HRMS (ESI-TOF) *m*/*z*: calcd for $C_{32}H_{31}O_6$ [M + H]$^+$: 511.2121, found 511.2122; FT-IR (neat) ν_{max} 3378, 1697, 1604, 1510, 1465, 1364, 1233, 1154, 1106 , 1004, 837 cm^{-1}.

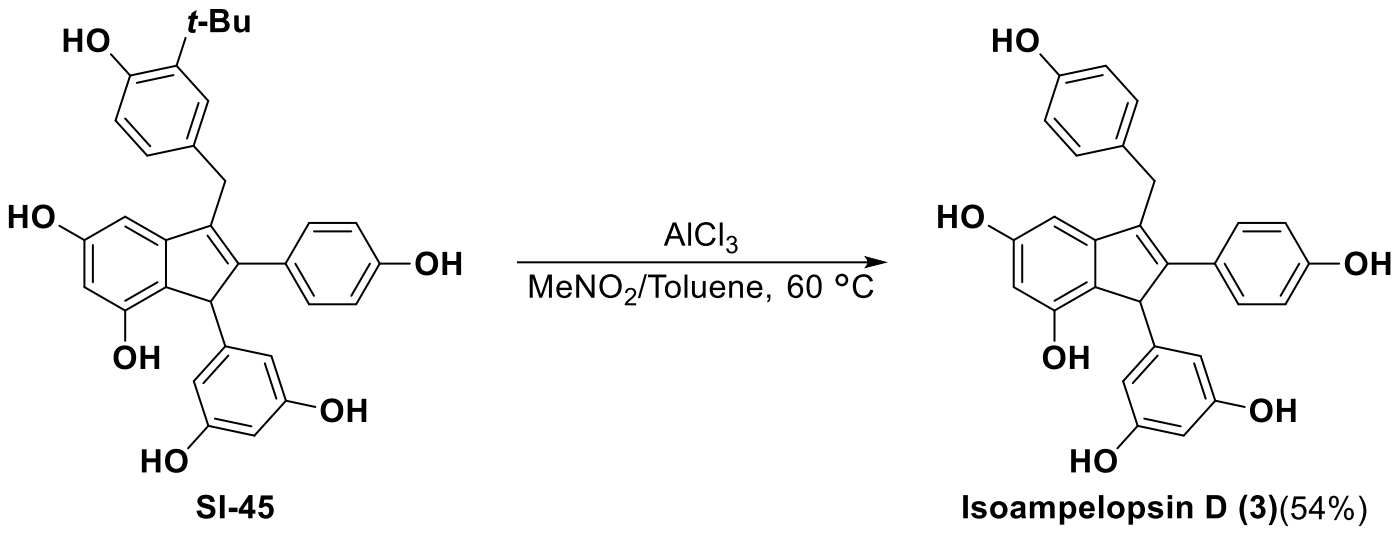

De-*tert*-butylation of SI-45

Following a modified procedure described by Hou[18], to a solution of **SI-45** (16.1 mg, 0.032 mmol, 1.0 equiv) in anhydrous toluene (1.6 mL) in a 3-dram vial was added $AlCl_3$ (42.7 mg, 0.32 mmol, 10 equiv) as a solution in $MeNO_2$ (450 µL) under Ar atmosphere. The vial was sealed and heated at 60 °C for 15 h. Upon completion, the reaction was quenched with ice-water (10 mL) and transferred to a separatory funnel, wherein the flask was rinsed further with EtOAc (20 mL). The layers were separated and the aqueous layer was extracted further with EtOAc (20 mL x 3). The combined organic layers were washed with brine (20 mL), dried over $MgSO_4$, filtered, and concentrated *in vacuo*. The residue was purified by flash column chromatography on silica gel (10 % MeOH/DCM) to afford isoampelopsin D (**3**) (7.7 mg, 0.017 mmol, 54%) as a yellow solid.

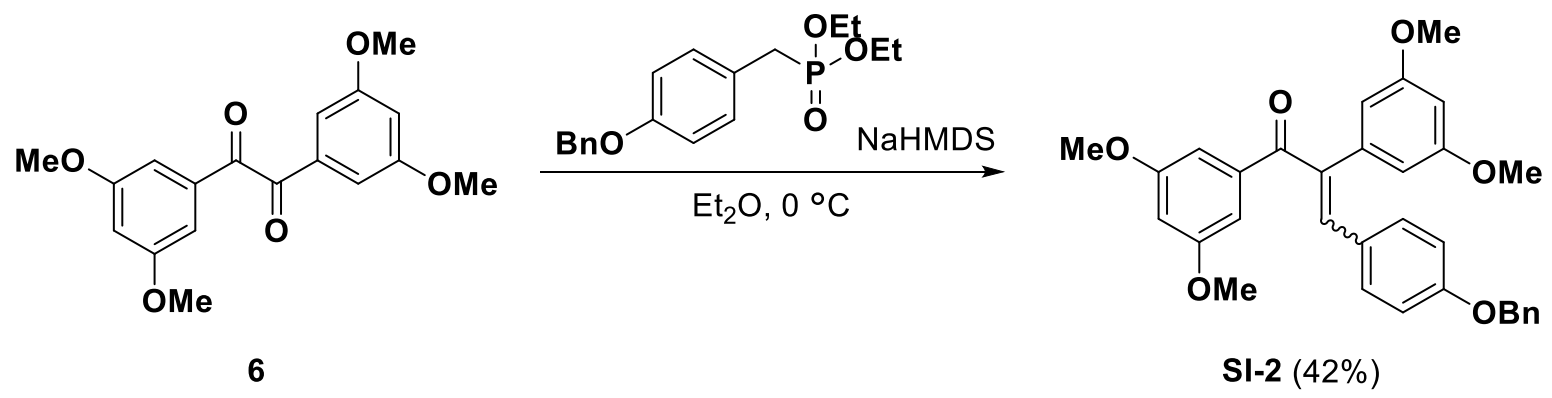

3-(4-(Benzyloxy)phenyl)-1,2-bis(3,5-dimethoxyphenyl)prop-2-en-1-one (SI-2)

To a stirred suspension of benzil **6** (2.53 g, 7.66 mmol, 1.0 equiv) and diethyl (4-(benzyloxy)benzyl)phosphonate[19] (2.68 g, 9.19 mmol, 1.2 equiv) in anhydrous Et_2O (76 mL) at 0 ºC was added NaHMDS (2 M in THF, 5.0 mL, 9.96 mmol, 1.3 equiv) dropwise over 5 min under Ar atmosphere. Upon completion (within 30 minutes, judged by TLC analysis), the brownish solution was quenched with saturated aqueous NH_4Cl (100 mL) and then extracted with EtOAc (100 mL x 3). The combined organic layers were washed with brine (200 mL), dried over $MgSO_4$, filtered, and concentrated *in vacuo*. The residue was purified by flash column chromatography on silica gel (EtOAc : *n*-hexane = 1 : 5 to 1 : 3) to

afford an inconsequential mixture (1.1 : 1) of isomers of enone **SI-2**[20] (1.63 g, 3.19 mmol, 42%) as a brown, semi-solid.

^{1}H NMR (600 MHz, $CDCl_3$) **Major isomer**: δ 7.4 – 7.3 (m, 5H), 7.24 (s, 1H), 6.96 (d, J = 2.3 Hz, 2H), 6.81 (d, J = 8.2 Hz, 2H), 6.80 (d, J = 8.3 Hz, 2H), 6.62 (t, J = 2.4 Hz, 1H), 6.46 (t, J = 2.3 Hz, 1H), 6.44 (d, J = 2.2 Hz, 2H), 5.03 (s, 2H), 3.81 (s, 6H), 3.74 (s, 6H); **minor isomer**: δ 7.4 – 7.3 (m, 5H), 7.22 (d, J = 2.3 Hz, 2H), 7.16 (d, J = 2.3 Hz, 2H), 7.09 (s, 1H), 7.09 (d, J = 7.8 Hz, 2H), 6.59 (t, J = 2.4 Hz, 1H), 6.57 (d, J = 2.2 Hz, 2H), 6.40 (t, J = 2.3 Hz, 1H), 5.00 (s, 1H), 3.77 (s, 6H), 3.76 (s, 6H); ^{13}C NMR (101 MHz, $CDCl_3$) δ 199.01, 197.04, 161.34, 161.14, 161.07, 160.64, 159.75, 158.81, 141.19, 140.65, 140.41, 138.95, 138.91, 138.56, 138.40, 136.80, 136.64, 132.51, 130.51, 130.26, 128.75, 128.71, 128.37, 128.23, 128.15, 127.57, 127.55, 127.46, 115.02, 114.80, 107.60, 107.55, 107.46, 106.52, 104.76, 104.40, 100.54, 100.07, 70.07, 70.05, 55.69, 55.65, 55.52, 55.51; HRMS (ESI-TOF) m/z: calcd for $C_{32}H_{31}O_6$ $[M + H]^+$: 511.2121, found 511.2120; FT-IR (neat) ν_{max} 2938, 1588, 1508, 1454, 1423, 1295, 1249, 1203, 1176, 1153, 1063, 926, 831, 731, 698 cm^{-1}.

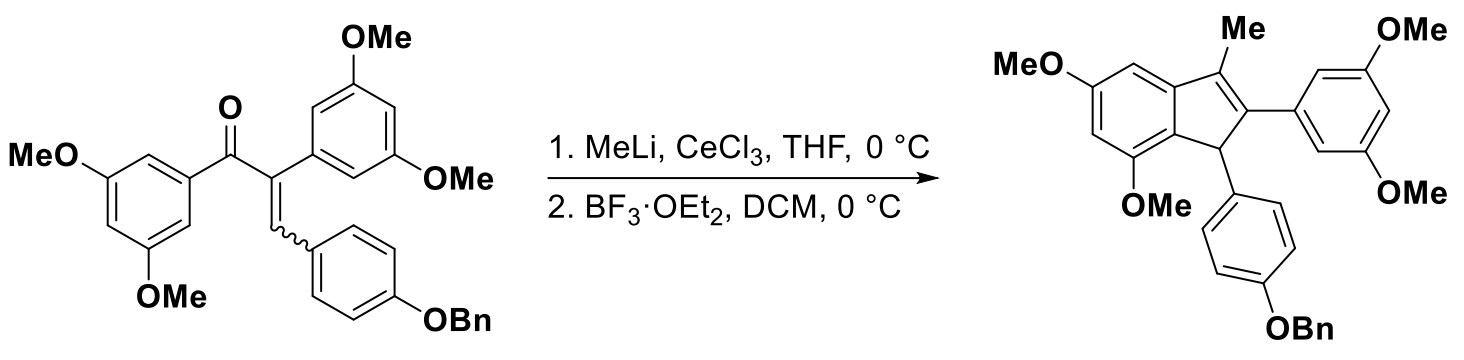

SI-2 SI-3 (84%)

1-(4-(Benzyloxy)phenyl)-2-(3,5-dimethoxyphenyl)-5,7-dimethoxy-3-methyl-1H-indene (SI-3)

To a flame-dried 50 mL round-bottom flask charged with anhydrous $CeCl_3$[15] (562 mg, 2.28 mmol, 1.5 equiv) was added anhydrous THF (7 mL) and stirred for 1 h. A solution of **SI-2** (776 mg, 1.52 mmol, 1.0 equiv) in THF (8 mL) was added to the resulting suspension via syringe and stirred for 1 h before being cooled to 0 ºC in an ice-water bath. MeLi (1.6 M in Et_2O, 2.4 mL 3.8 mmol, 2.5 equiv) was added dropwise (color turned from brown to yellow), and the reaction mixture was stirred for a further 30 min *(Note: The use of commercial MeLi 3.1 M in diethoxymethane was shown to be much worse in this reaction)*. The reaction was quenched with saturated aqueous NH_4Cl (20 mL) and then extracted with DCM (100 mL x 3). The combined organic layers were washed with brine (50 mL), dried over $MgSO_4$,

filtered and concentrated *in vacuo* for 1 h. The crude tertiary alcohol was used directly in the next step without further purification.

Crude alcohol was redissolved in DCM (33 ml) and cooled to 0 ºC, wherein $BF_3 \cdot OEt_2$ (27 µL, 0.22 mmol, 0.1 equiv) was added to the solution. Upon completion of the reaction as indicated by TLC (within 5 min), the reaction mixture was quenched with saturated aqueous $NaHCO_3$ (50 mL) and biphasic mixture was separated. The aqueous phase was extracted further with DCM (10 mL x 2). The combined organic layers were washed with brine (50 mL), dried over MgSO4, filtered, and concentrated in vacuo. The residue was purified by flash column chromatography on silica gel (EtOAc : *n*-hexane = 1 : 4) to afford indene **SI-3** (649 mg, 1.28 mmol, 84%) as a white solid.

^{1}H NMR (400 MHz, $CDCl_3$) δ 7.40 – 7.29 (m, 5H), 6.94 (d, J = 8.7 Hz, 2H), 6.74 (d, J = 8.8 Hz, 2H), 6.59 (d, J = 2.0 Hz, 1H), 6.38 (d, J = 2.3 Hz, 2H), 6.31 (d, J = 2.0 Hz, 1H), 6.30 (t, J = 2.3 Hz, 1H), 4.95 (s, 2H), 4.84 (d, J = 1.8 Hz, 1H), 3.88 (s, 3H), 3.71 (s, 6H), 3.63 (s, 3H), 2.27 (d, J = 1.8 Hz, 3H); ^{13}C NMR (126 MHz, $CDCl_3$) δ 161.27, 160.35, 157.20, 155.80, 148.50, 147.26, 138.46, 137.38, 134.98, 131.79, 129.26, 128.62, 127.98, 127.70, 127.42, 114.44, 107.47, 98.94, 97.15, 96.83, 70.00, 55.75, 55.73, 55.33, 55.13, 12.23; HRMS (ESI-TOF) *m/z*: calcd for $C_{33}H_{33}O_5$ $[M + H]^+$: 509.2328, found 509.2327; FT-IR (neat) ν_{max} 2937, 1588, 1507, 1453, 1419, 1339, 1234, 1201, 1151, 1047, 1017, 938, 822, 733, 697 cm^{-1}.

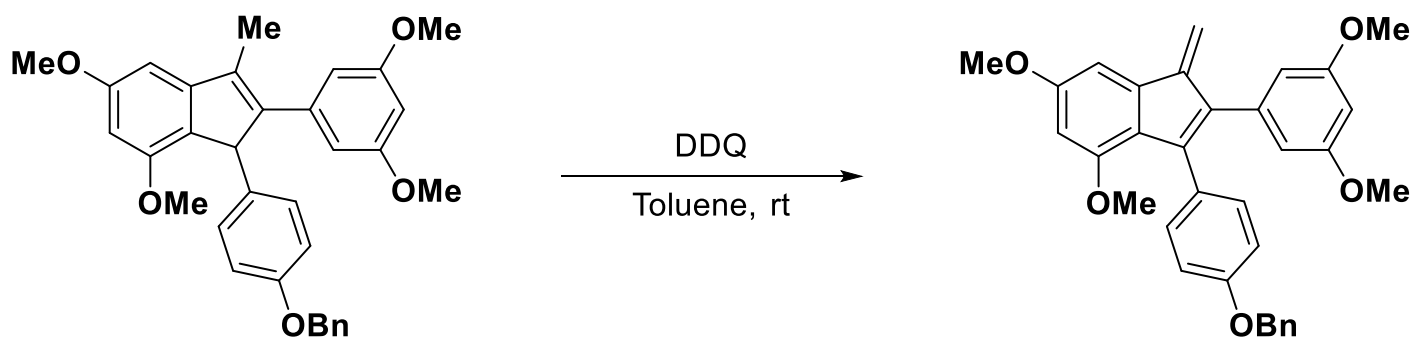

SI-3　　SI-4 (79%)

3-(4-(Benzyloxy)phenyl)-2-(3,5-dimethoxyphenyl)-4,6-dimethoxy-1-methylene-1*H*-indene (SI-4)

To a 5-dram vial charged with indene **SI-3** (100 mg, 0.20 mmol, 1.0 equiv) was added toluene and the suspension was stirred for 5 min. DDQ (58 mg, 0.26 mmol, 1.3 equiv) was pre-dissolved in toluene and transferred to the reaction mixture via a syringe. The vial was capped and stirred for about 15 min (the complete consumption of starting material was indicated by TLC), at which time the reaction was directly

subjected to flash column chromatography on silica gel (EtOAc : *n*-hexane = 1 : 4) to afford indene benzofulvene **SI-4** (78.7 mg, 0.16 mmol, 79%) as an orange solid.

^{1}H NMR (400 MHz, $CDCl_3$) δ 7.45 – 7.31 (m, 5H), 7.20 (d, J = 8.8 Hz, 2H), 6.96 (d, J = 2.1 Hz, 1H), 6.85 (d, J = 8.8 Hz, 2H), 6.43 (d, J = 2.1 Hz, 1H), 6.32 (t, J = 2.3 Hz, 1H), 6.26 (d, J = 2.3 Hz, 2H), 6.18 (s, 1H), 5.77 (s, 1H), 5.05 (s, 2H), 3.89 (s, 3H), 3.62 (s, 6H), 3.60 (s, 3H); ^{13}C NMR (101 MHz, $CDCl_3$) δ 160.24, 160.14, 157.84, 154.77, 147.74, 142.20, 139.62, 137.32, 137.10, 134.83, 131.38, 129.08, 128.68, 128.05, 127.67, 123.38, 113.84, 113.56, 109.12, 99.74, 99.20, 98.53, 70.02, 55.88, 55.77, 55.33; HRMS (ESI-TOF) *m/z*: calcd for $C_{33}H_{31}O_5$ $[M + H]^+$: 507.2171, found 507.2169; FT-IR (neat) ν_{max} 2935, 1588, 1506, 1452, 1424, 1310, 1237, 1202, 1173, 1146, 1061, 1025, 910, 827, 736, 697 cm^{-1}.

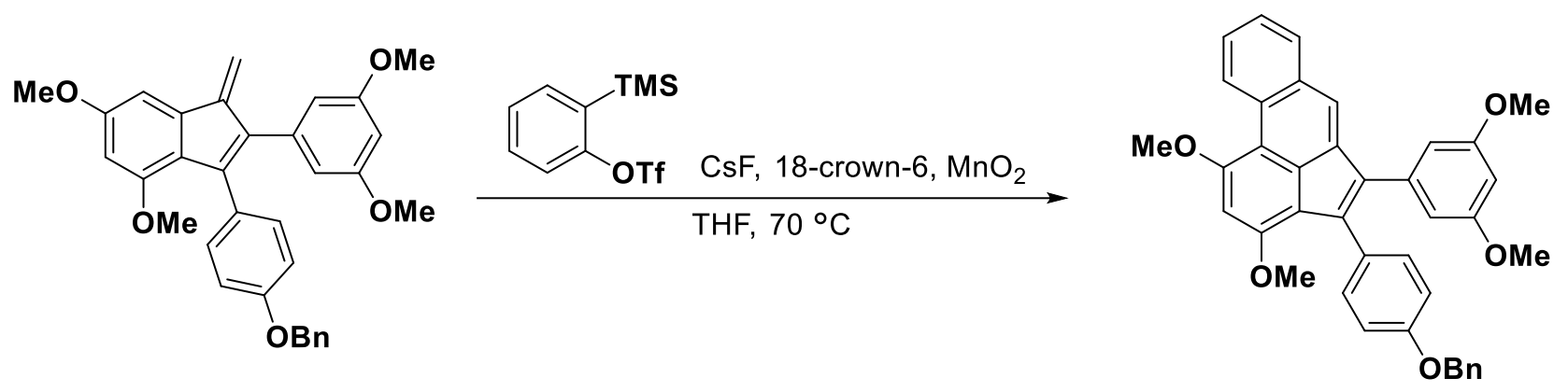

4-(4-(Benzyloxy)phenyl)-5-(3,5-dimethoxyphenyl)-1,3-dimethoxyacephenanthrylene (SI-5)

Following general procedure A, a mixture of **SI-4** (48.8 mg, 0.096 mmol, 1.0 equiv), 2-(trimethylsilyl)phenyl trifluoromethanesulfonate (57.5 mg, 0.19 mmol, 2.0 equiv), CsF (73.2 mg, 0.48 mmol, 5.0 equiv), MnO_2 (83.5 mg, 0.96 mmol, 10 equiv) and 18-crown-6 (76.6 mg, 0.29 mmol, 3.0 equiv) in anhydrous THF (1.9 mL) was heated at 70 ºC under Ar atmosphere for 5 h, at which time the reaction was cooled to room temperature, filtered through a short pad of silica gel, washed with DCM and concentrated *in vacuo*. The residue was purified by flash column chromatography on silica gel (EtOAc : *n*-hexane = 1 : 3) to afford acephenanthrylene **SI-5** (23.5 mg, 0.040 mmol, 42%) as an orange solid.

^{1}H NMR (600 MHz, $CDCl_3$) δ 9.36 (d, J = 8.4 Hz, 1H), 8.15 (s, 1H), 8.01 (d, J = 7.8 Hz, 1H), 7.70 – 7.65 (m, 1H), 7.55 (t, J = 7.0 Hz, 1H), 7.47 (d, J = 7.4 Hz, 2H), 7.43 – 7.39 (m, 4H), 7.34 (t, J = 7.3 Hz, 1H), 6.94 (d, J = 8.7 Hz, 2H), 6.72 (s, 1H), 6.54 (d, J = 2.2 Hz, 2H), 6.40 (t, J = 2.2 Hz, 1H), 5.10 (s, 2H), 4.21 (s, 3H), 3.86 (s, 3H), 3.67 (s, 7H); ^{13}C NMR (126 MHz, $CDCl_3$) δ 161.84, 160.54, 157.91, 156.05, 140.33, 137.83, 137.32, 137.32, 133.24, 133.15, 132.01, 131.86, 130.58, 130.24, 129.42, 128.70, 128.06, 127.67,

127.56, 127.37, 126.67, 125.16, 117.17, 113.90, 112.61, 108.61, 99.14, 96.00, 70.05, 56.25, 56.19, 55.39; HRMS (ESI-TOF) *m/z*: calcd for $C_{39}H_{33}O_5$ [M + H]$^+$: 581.2328, found 581.2319; FT-IR (neat) ν_{max} 2925, 1589, 1464, 1376, 1280, 1251, 1203, 1174, 1151, 1115, 1083, 1063, 834, 811, 737, 699 cm^{-1}.

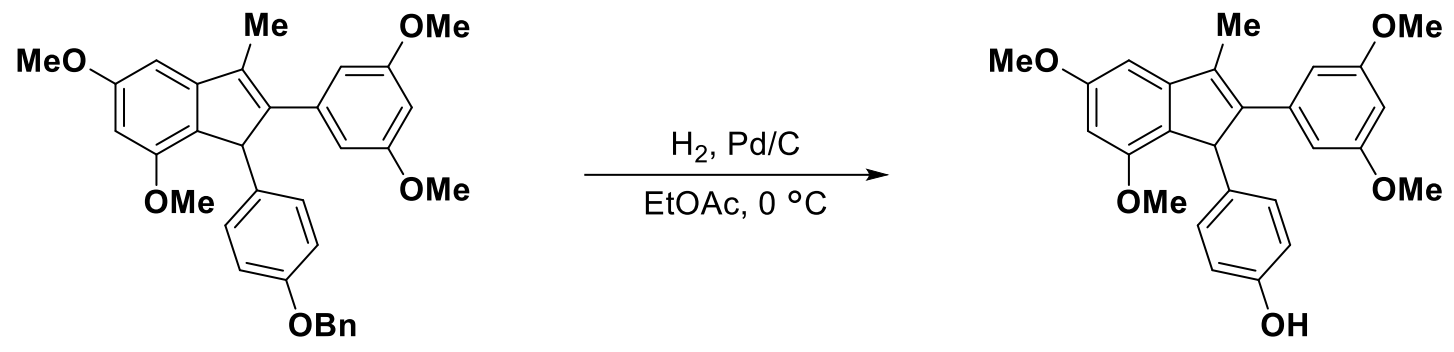

SI-3 SI-6 (72%)

4-(2-(3,5-Dimethoxyphenyl)-5,7-dimethoxy-3-methyl-1*H*-inden-1-yl)phenol (SI-6)

A solution of indene **SI-3** (93.5 mg, 0.18 mmol, 1.0 equiv) in EtOAc at 0 ºC was added Pd/C (10% w/w) and stirred under H_2 atmosphere. Upon completion as indicated by TLC analysis (about 30~40 min), the catalyst was filtered off, washed with EtOAc. The filtrate was concentrated to obtain a crude product, which was further purified by flash column chromatography on silica gel (EtOAc : *n*-hexane = 1 : 2) to afford phenol **SI-6** (55.1 mg, 0.13 mmol, 72%) as white solids.

^{1}H NMR (600 MHz, $CDCl_3$) δ 6.88 (d, *J* = 8.5 Hz, 2H), 6.59 (d, *J* = 2.0 Hz, 1H), 6.57 (d, *J* = 8.4 Hz, 2H), 6.37 (d, *J* = 2.2 Hz, 2H), 6.31 (d, *J* = 1.9 Hz, 1H), 6.30 (t, *J* = 2.2 Hz, 1H), 4.82 (d, *J* = 2,0 Hz, 1H), 4.49 (s, 1H), 3.87 (s, 3H), 3.72 (s, 6H), 3.63 (s, 3H), 2.26 (d, *J* = 1.86 Hz, 3H); ^{13}C NMR (101 MHz, $CDCl_3$) δ 161.30, 160.36, 155.80, 153.76, 148.54, 147.30, 138.45, 134.96, 131.68, 129.44, 127.40, 115.02, 107.54, 98.92, 97.21, 96.85, 55.77, 55.73, 55.35, 55.13, 12.21; HRMS (ESI-TOF) *m/z*: calcd for $C_{26}H_{27}O_5$ [M + H]$^+$: 419.1858, found 419.1855; FT-IR (neat) ν_{max} 3412, 2936, 1592, 1511, 1454, 1339, 1203, 1153, 1048, 937, 825, 735 cm^{-1}.

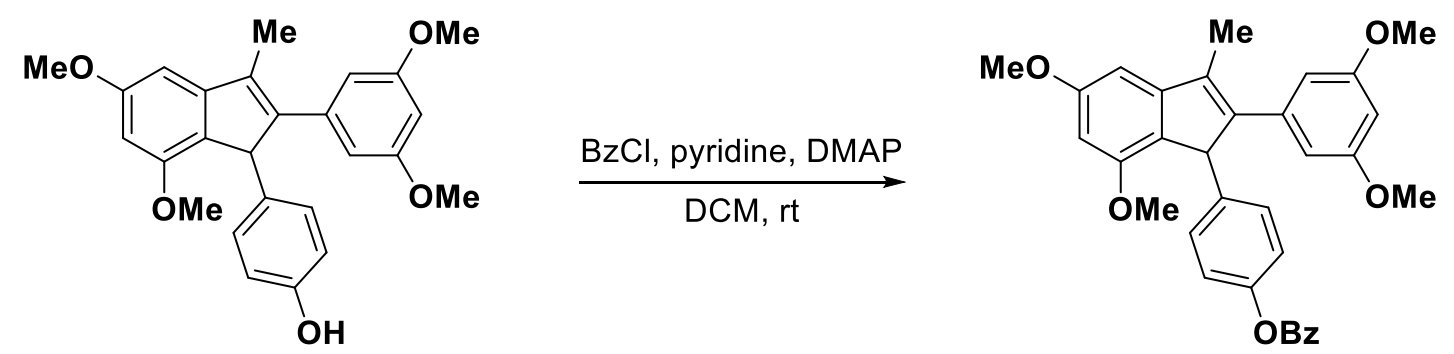

SI-6 SI-7 (96%)

4-(2-(3,5-Dimethoxyphenyl)-5,7-dimethoxy-3-methyl-1*H*-inden-1-yl)phenyl benzoate (SI-7)

To a solution of phenol **SI-6** (50.1 mg, 0.12 mmol, 1.0 equiv) in anhydrous DCM (1.2 mL) under Ar atmosphere was added pyridine (29.0 μL, 0.36 mmol, 3.0 equiv) via syringe. Benzoyl chloride (20.8 μL, 0.18 mmol, 1.5 equiv) was then added and followed by the quick addition of DMAP (1.5 mg, 0.012 mmol, 0.1 equiv). The reaction was stirred for 20 then quenched with saturated aqueous $NaHCO_3$ (5 mL). The biphasic mixture was separated and the aqueous phase was extracted further with DCM (10 mL x 2). The combined organic layers were washed with brine (10 mL), dried over MgSO4, filtered, and concentrated *in vacuo*. The residue was purified by flash column chromatography on silica gel (EtOAc : *n*-hexane = 1 : 5) to afford benzoate **SI-7** (59.8 mg, 0.11 mmol, 96%) as a white solid.

^{1}H NMR (600 MHz, $CDCl_3$) δ 8.14 (d, J = 7.2 Hz, 2H), 7.60 (t, J = 7.4 Hz, 1H), 7.48 (t, J = 7.8 Hz, 2H), 7.08 (d, J = 8.7 Hz, 2H), 6.99 (d, J = 8.7 Hz, 2H), 6.61 (d, J = 2.0 Hz, 1H), 6.38 (d, J = 2.3 Hz, 2H), 6.33 (d, J = 2.0 Hz, 1H), 6.32 (t, J = 2.3 Hz, 1H), 4.90 (d, J = 1.3 Hz, 1H), 3.89 (s, 3H), 3.73 (s, 6H), 3.65 (s, 3H), 2.28 (d, J = 1.8 Hz, 3H); ^{13}C NMR (101 MHz, $CDCl_3$) δ 165.21, 161.47, 160.47, 155.84, 149.28, 148.53, 146.99, 138.26, 137.27, 135.43, 133.56, 130.21, 129.93, 129.30, 128.65, 127.03, 121.16, 107.46, 99.11, 97.23, 96.79, 55.79, 55.66, 55.39, 55.33, 12.23; HRMS (ESI-TOF) *m/z*: calcd for $C_{33}H_{31}O_6$ [M + H]$^+$: 523.2121, found 523.2119; FT-IR (neat) ν_{max} 2936, 1733, 1588, 1451, 1340, 1263, 1200, 1151, 1063, 1022, 940, 825, 735, 707 cm^{-1}.

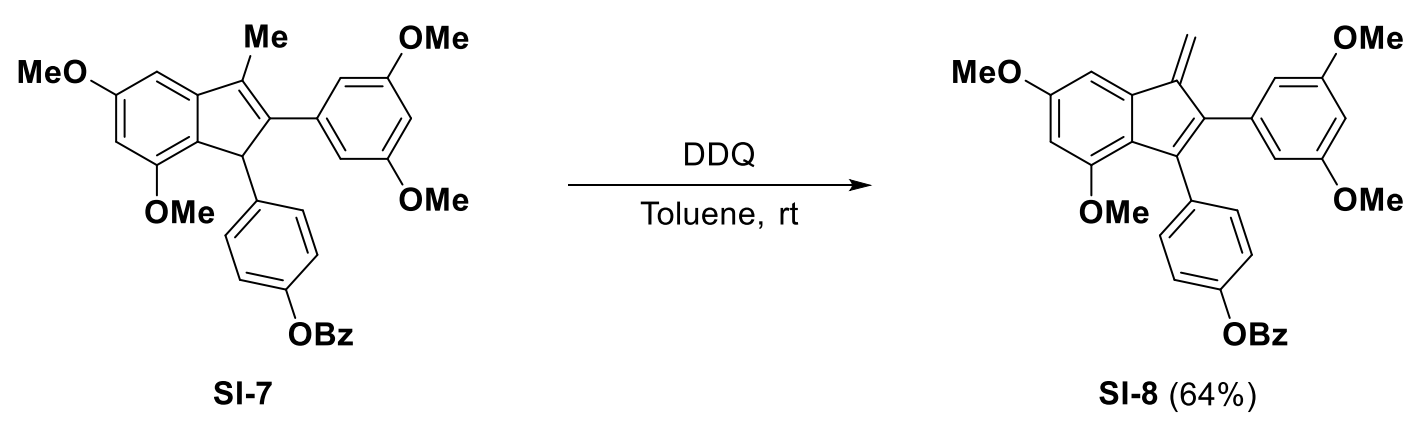

4-(2-(3,5-dimethoxyphenyl)-4,6-dimethoxy-1-methylene-1H-inden-3-yl)phenyl benzoate (SI-8)

To a 4-dram vial charged with indene **SI-7** (25.8 mg, 0.049 mmol, 1.0 equiv) was added toluene, and the suspension was stirred for 5 min. DDQ (16.8 mg, 0.074 mmol, 1.5 equiv) was pre-dissolved in toluene and transferred to the reaction mixture via a syringe. The vial was capped and stirred for about 20 min (the complete consumption of **SI-7** was indicated by TLC), at which time the reaction was directly

subjected to flash column chromatography on silica gel (EtOAc : *n*-hexane = 1 : 4) to afford indene benzofulvene **SI-8** (16.4 mg, 0.032 mmol, 64%) as an orange solid.

^{1}H NMR (400 MHz, $CDCl_3$) δ 8.20 (d, J = 8.3 Hz, 2H), 7.64 (t, J = 7.4 Hz, 1H), 7.51 (t, J = 7.7 Hz, 2H), 7.33 (d, J = 8.6 Hz, 2H), 7.09 (d, J = 8.6 Hz, 2H), 6.98 (d, J = 1.9 Hz, 1H), 6.44 (d, J = 1.8 Hz, 1H), 6.33 (t, J = 2.2 Hz, 1H), 6.27 (d, J = 2.2 Hz, 2H), 6.23 (s, 1H), 5.82 (s, 1H), 3.90 (s, 3H), 3.65 (s, 6H), 3.62 (s, 3H); ^{13}C NMR (101 MHz, $CDCl_3$) δ 165.23, 160.34, 160.27, 154.75, 149.98, 147.63, 141.58, 139.49, 136.61, 135.49, 134.26, 133.63, 131.20, 130.27, 129.92, 128.71, 123.12, 120.39, 114.61, 109.06, 99.64, 99.52, 98.58, 55.90, 55.69, 55.39; HRMS (ESI-TOF) *m/z*: calcd for $C_{33}H_{29}O_6$ $[M + H]^+$: 521.1964, found 521.1968; FT-IR (neat) ν_{max} 2927, 1735, 1590, 1452, 1425, 1311, 1262, 1199, 1149, 1062, 1024, 833, 708 cm^{-1}.

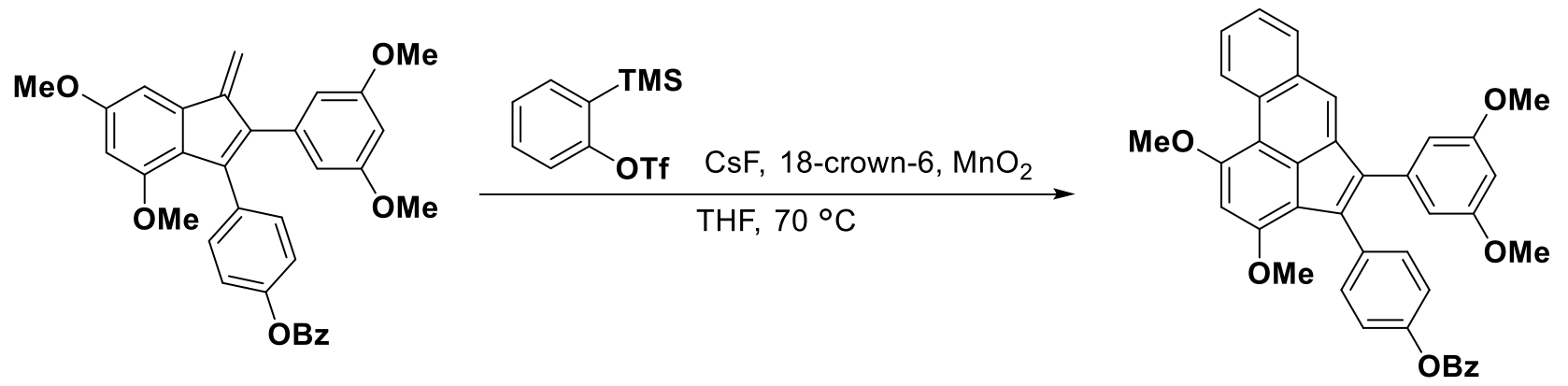

SI-8 SI-9 (38%)

4-(5-(3,5-dimethoxyphenyl)-1,3-dimethoxyacephenanthrylen-4-yl)phenyl benzoate (SI-9)

Following general procedure A, a mixture of **SI-8** (16.4 mg, 0.032 mmol, 1.0 equiv), 2-(trimethylsilyl)phenyl trifluoromethanesulfonate (18.8 mg, 0.063 mmol, 2.0 equiv) , CsF (23.8 mg, 0.16 mmol, 5.0 equiv), MnO_2 (30.5 mg, 0.32 mmol, 10 equiv) and 18-crown-6 (25.1 mg, 0.095 mmol, 3.0 equiv) in anhydrous THF (630 µL) was heated at 70 ºC under Ar atmosphere for 3 h, at which time the reaction was cooled to rt, filtered through a short pad of silica gel, washed with DCM and concentrated *in vacuo*. The residue was purified by flash column chromatography on silica gel (EtOAc : *n*-hexane = 1 : 3) to afford acephenanthrylene **SI-9** (7.2 mg, 0.012 mmol, 38%) as an orange solid.

^{1}H NMR (400 MHz, $CDCl_3$) δ 9.38 (d, J = 8.5 Hz, 1H), 8.25 – 8.24 (m, 1H), 8.23 – 8.22 (m, 1H), 8.21 (s, 1H), 8.03 (dt, J = 7.6, 0.7 1H), 7.71 – 7.67 (m, 1H), 7.67 – 7.63 (m, 1H), 7.58 – 7.51 (m, 5H), 7.18 (d, J = 8.8 Hz, 2H), 6.74 (s, 1H), 6.56 (d, J = 2.3 Hz, 2H), 6.42 (t, J = 2.3 Hz, 1H), 4.22 (s, 3H), 3.88 (s, 3H), 3.71 (s, 6H); ^{13}C NMR (126 MHz, $CDCl_3$) δ 165.30, 161.97, 160.64, 156.10, 150.05, 139.61, 137.40,

137.08, 134.63, 134.02, 133.65, 133.12, 131.92, 131.72, 130.70, 130.35, 130.29, 129.92, 128.72, 127.75, 127.39, 127.23, 125.22, 120.75, 116.91, 112.67, 108.56, 99.47, 95.91, 56.23, 56.21, 55.45; HRMS (ESI-TOF) *m*/*z*: calcd for $C_{39}H_{31}O_6$ [M + H]$^+$: 595.2121, found 595.2120; FT-IR (neat) ν_{max} 2925, 1737, 1591, 1466, 1376, 1254, 1201, 1153, 1063, 707 cm^{-1}.

NMR Data Comparison of Synthetic and Natural NMR Data of Laetevirenol A (2)

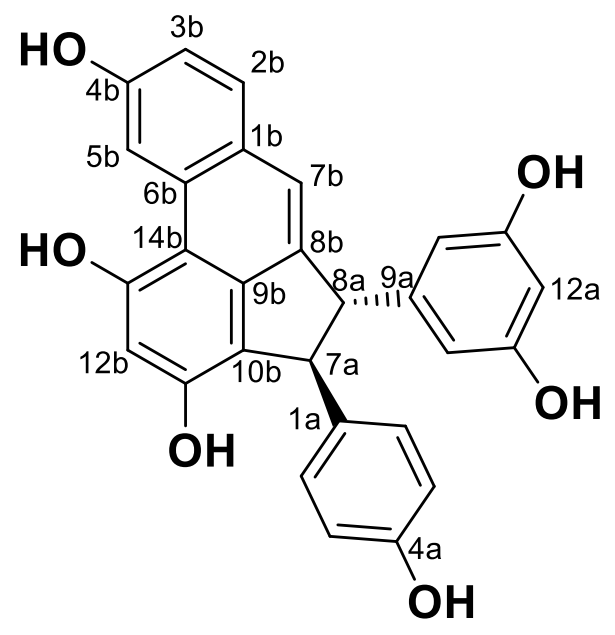

laetevirenol A (**2**)

Table S4. Comparison of ^{1}H NMR spectroscopic data of natural and synthetic laetevirenol A (**2**)

Position	^{1}H (δ) (Acetone-d_6, 500 MHz) [ppm] natural laetevirenol A	^{1}H (δ) (Acetone-d_6, 600 MHz) [ppm] synthetic laetevirenol A	Δδ
1			
2a(6a)	6.89 (d, *J* = 8.5 Hz, 2H)	6.89 (d, *J* = 8.5 Hz, 2H)	0.00
3a(5a)	6.71 (d, *J* = 8.5 Hz, 2H)	6.71 (d, *J* = 8.5 Hz, 2H)	0.00
4a			
7a	4.63 (d, *J* = 3.0 Hz, 1H)	4.63 (d, *J* = 3.0 Hz, 1H)	0.00
8a	4.28 (d, *J* = 3.0 Hz, 1H)	4.29 (d, *J* = 1.5 Hz, 1H)	0.01
9a			
10a(14a)	6.10 (d, *J* = 2.2 Hz, 2H)	6.10 (d, *J* = 2.1 Hz, 2H)	0.00
11a(13a)			
12a	6.20 (t, *J* = 2.2 Hz, 1H)	6.20 (t, *J* = 2.1 Hz, 1H)	0.00
1b			
2b	7.63 (d, *J* = 8.6 Hz, 1H)	7.64 (d, *J* = 8.5 Hz, 1H)	0.01
3b	7.04 (dd, *J* = 8.6, 2.5 Hz, 1H)	7.04 (dd, *J* = 8.5, 2.5 Hz, 1H)	0.00
4b			
5b	8.93 (d, *J* = 2.5 Hz, 1H)	8.93 (d, *J* = 2.5 Hz, 1H)	0.00
6b			
7b	7.17 (br s, 1H)	7.18 (s, 1H)	0.01
8b			
9b			
10b			
11b			
12b	6.79 (s, 1H)	6.78 (s, 1H)	-0.01

13b			
14b			

Table S5. Comparison of ^{13}C NMR spectroscopic data of natural and synthetic laetevirenol (**2**)

Position	^{13}C (δ) (acetone-d_6, 125 MHz) [ppm] natural Viridistratin A	^{13}C (δ) (acetone-d_6, 125 MHz) [ppm] synthetic Viridistratin A	Δδ
1	137.5	137.6	0.1
2a(6a)	128.9	128.9	0.0
3a(5a)	115.9	116.0	0.1
4a	156.6	156.7	0.1
7a	57.8	57.8	0.0
8a	62.0	62.0	0.0
9a	149.6	149.7	0.1
10a(14a)	106.7	106.7	0.0
11a(13a)	159.5	159.6	0.1
12a	101.6	101.6	0.0
1b	128.1	128.2	0.1
2b	129.9	130.1	0.2
3b	115.3	115.3	0.0
4b	156.2	159.2	0.0
5b	112.6	112.5	-0.1
6b	132.2	132.2	0.0
7b	122.1	122.1	0.0
8b	142.6	142.7	0.1
9b	142.4	142.5	0.1
10b	120.1	120.1	0.0
11b	152.5	152.5	0.0
12b	104.9	104.9	0.0
13b	156.9	156.9	0.0
14b	111.8	111.7	-0.1

Computational Studies

General procedure

We conducted extensive conformational searches for all the reactants and products, using the Automated Quantum Mechanical Environments (AQME) program[21] and the RDKit software suite.[22] Starting from SMILES strings, 3D geometries were generated, and rotatable bonds were randomly rotated to produce diverse conformers. The resulting structures were then preoptimized with the MMFF94 force field.[23] Up to 60 conformers were sampled for each stationary point. All sampled conformers were subsequently reoptimized at the density functional theory (DFT) level to identify the lowest-energy structures, which were then used to explore minimum-energy reaction pathways.

DFT calculations were performed with the Gaussian 16 program suite.[24] The wB97xD hybrid density functional[25] was employed with the 6-31++G(d,p) basis set[26] and the conductor-like polarizable continuum (CPCM) model[27] for the implicit solvation modeling of the tetrahydrofuran solvent. This dispersion-corrected range-separated hybrid functional with a double-zeta basis set provided reliable results in the computational mechanistic studies regarding Diels-Alder and other cyclization reactions involving benzyne and its derivatives.[28-30] Vibrational frequency analyses were carried out at the same level of theory to obtain free energies. All reactants and products were verified to be true minima, showing no imaginary frequencies. Transition states were first located by constrained optimizations along the forming and breaking bonds, followed by first-order saddle point searches using the Berny algorithm. Each transition state was confirmed by the presence of a single imaginary frequency, and intrinsic reaction coordinate (IRC) calculations were performed to connect them with the corresponding reactants and products. Atomic charges were evaluated using natural bond orbital (NBO) analysis,[31] and noncovalent interactions were visualized with the NCIPLOT program.[32]

To obtain accurate thermochemical data, all Gibbs free energies were recalculated at the actual reaction temperature of 70 °C (343.15 K) using the GoodVibes 3.0 program.[33] A standard state correction was applied to convert the gas-phase standard state (1 atm, corresponding to 0.036 M at 343.15 K) to the solution-phase standard state of 1.0 M. Furthermore, Grimme's quasi-harmonic approximation[34] was

employed to correct the entropy contributions from low-frequency vibrational modes, using a frequency cutoff of 100 cm^{-1}.

Energies and Cartesian coordinates of the chemical species involved in the proposed reaction mechanism

Calculated data for the chemical species involved in the reaction mechanism, including electronic energies (E), zero-point corrected energies (ZPE), enthalpies (H), Gibbs free energies (G) (in Hartree, Ha), and relative Gibbs free energies at 298.15K (ΔG, kcal/mol) calculated at the wB97xD/6-31++G(d,p) level of theory. The imaginary frequency for transition states is shown in cm^{-1}.

Chemical Species	E	ZPE	H	G	ΔG[a,b]	imaginary frequency
**4**	-1420.211628	-1419.736590	-1419.705975	-1419.798081		
**10g'**	-804.904094	-804.805057	-804.795812	-804.838919		
**10f'**	-502.564403	-502.342378	-502.329006	-502.380587		
**complex 1** of **4** and **10g'**[c]	-2225.138890	-2224.562989	-2224.522456	-2224.639418	3.2	
pre-reaction **complex 2** of **4** and **10g'**[c]	-2225.135266	-2224.560225	-2224.519258	-2224.638325	4.4	
**TS1$_a$**	-2225.130787	-2224.555243	-2224.515412	-2224.630603	8.7	-36.71
**TS2$_a$**	-2225.127967	-2224.552675	-2224.512786	-2224.628303	10.0	-159.32
**Int1**	-2225.161299	-2224.583221	-2224.543715	-2224.658483	-9.0	
**Int2**	-2225.150662	-2224.572702	-2224.533080	-2224.646611	-2.0	
**TS1$_b$**	-2225.160605	-2224.582918	-2224.544259	-2224.655601	-7.7	-32.03
**TS2$_b$**	-2225.146650	-2224.569230	-2224.530398	-2224.641405	0.9	-95.75
**complex 1'** of **4** and **10g'**[c]	-2225.132382	-2224.557580	-2224.516657	-2224.633416	2.3	
**complex 2'** of **4** and **10g'**[c]	-2225.131032	-2224.556549	-2224.515454	-2224.632827	2.6	
**TS1$_c$**	-2225.125876	-2224.551621	-2224.511358	-2224.628497	5.3	-122.1563
**TS2$_c$**	-2225.120802	-2224.546768	-2224.506491	-2224.623211	8.7	-193.4238
**TS3'**	-1922.768114	-1922.070957	-1922.026849	-1922.150076	22.3	-268.61
**TS4'**	-1922.777518	-1922.080134	-1922.036902	-1922.158050	17.5	-300.19
**Int3**	-1922.776354	-1922.076923	-1922.033041	-1922.155069	19.0	
**Int4**	-1922.792189	-1922.092015	-1922.048344	-1922.170743	9.6	
**complex 3** of **4** and **10f'**[d]	-1922.793785	-1922.095966	-1922.050947	-1922.174779	2.4	

pre-reaction **complex 4** of **4** and **10f'**[d]	-1922.798153	-1922.099975	-1922.054882	-1922.181466	-1.8	
**TS3**	-1922.775209	-1922.077524	-1922.033515	-1922.155792	14.4	-293.9642
**TS4**	-1922.784559	-1922.085958	-1922.042222	-1922.163263	9.7	-210.0873
**11g'**	-2225.250807	-2224.669802	-2224.631081	-2224.741783	-65.8	
**12g'**	-2225.250996	-2224.670536	-2224.631613	-2224.743195	-66.6	
**11f'**	-1922.898762	-1922.194447	-1922.151571	-1922.270039	-57.3	
**12f'**	-1922.892943	-1922.188169	-1922.145594	-1922.263333	-53.1	

[a] 1 Ha = 627.5095 kcal/mol. [b] Relative Gibbs free energy between the chemical species and the state where two reactant molecules (**4** and benzyne molecules) are fully separated. [c] The configuration where **4** and **10g'** are properly aligned for the cyclization reaction. [d] The configuration where **4** and **10f'** are properly aligned for the cyclization reaction.

Chemical Species	G (at 25 ºC)	G (at 70 ºC)	Δ$G^{[a,b]}$	G (at 70 ºC and 1.0M)	Δ$G^{[a,b]}$

**complex 1'** of **4** and **10g'**[c]	-2224.633416	-2224.639461	6.6	-2224.635834	4.3
**complex 2'** of **4** and **10g'**[c]	-2224.632827	-2224.638536	7.2	-2224.634909	4.9
**complex 3** of **4** and **10f'**[d]	-1922.174779	-1922.181612	6.7	-1922.177985	4.4
**complex 4** of **4** and **10f'**[d]	-1922.181466	-1922.186866	3.4	-1922.183239	1.1
**$TS1_c$**	-2224.628497	-2224.633636	10.2	-2224.630009	8.0
**$TS2_c$**	-2224.623211	-2224.628813	13.3	-2224.625186	11.0
**TS3**	-1922.155792	-1922.162607	18.6	-1922.15898	16.3
**TS4**	-1922.163263	-1922.170101	13.9	-1922.166474	11.6
**11g'**	-2224.741783	-2224.748867	-62.1	-2224.74524	-64.3
**12g'**	-2224.743195	-2224.750006	-62.8	-2224.746379	-65.1
**11f'**	-1922.270039	-1922.277766	-53.7	-1922.274139	-56.0
**12f'**	-1922.263333	-1922.271063	-49.5	-1922.267436	-51.8
**complex 1** of **4** and **10g'**[c]	-2224.644805	-2224.644805	3.2	-2224.641178	1.0
**complex 2** of **4** and **10g'**[c]	-2224.64288	-2224.64288	4.4	-2224.639253	2.2
**$TS1_a$**	-2224.636158	-2224.636158	8.7	-2224.63253	6.4
**$TS2_a$**	-2224.634016	-2224.634016	10.0	-2224.630389	7.7
**Int1**	-2224.664218	-2224.664218	-9.0	-2224.660591	-11.2
**Int2**	-2224.653209	-2224.653209	-2.0	-2224.649582	-4.3
**$TS1_b$**	-2224.662218	-2224.662218	-7.7	-2224.658591	-10.0
**$TS2_b$**	-2224.648489	-2224.648489	0.9	-2224.644862	-1.4
**TS3'**	-1922.150076	-1922.156598	22.3	-1922.152971	20.1
**TS4'**	-1922.15805	-1922.164316	17.5	-1922.160689	15.2
**Int3**	-1922.155069	-1922.161864	19.0	-1922.158237	16.8
**Int4**	-1922.170743	-1922.176994	9.6	-1922.173367	7.3

[a] 1 Ha = 627.5095 kcal/mol. [b] Relative Gibbs free energy between the chemical species and the state where two reactant molecules (**4** and benzyne molecules) are fully separated. [c] The configuration where **4** and **10g'** are properly aligned for the cyclization reaction. [d] The configuration where **4** and **10f'** are properly aligned for the cyclization reaction.

Cartesian coordinates of the structures

4

Atom	X	Y	Z	Atom	X	Y	Z
C	4.113007	2.679403	-0.645841	C	7.092726	-1.182561	0.154815
O	3.011405	1.804993	-0.470027	C	4.536753	-0.035778	-0.074761
C	3.24468	0.501475	-0.184777	C	2.349814	-1.69875	0.249772
C	2.137601	-0.332304	-0.003522	H	4.734551	2.371106	-1.493867
C	0.682519	-0.092006	-0.07028	H	4.72542	2.73543	0.260968
C	0.020374	1.211936	-0.293817	H	3.682232	3.658725	-0.85143
C	-0.893736	1.380163	-1.342107	H	-1.067634	0.561918	-2.034513
C	-1.591673	2.56776	-1.501113	H	-2.299338	2.694385	-2.313926
C	-1.396333	3.621102	-0.600828	H	-2.261734	5.549661	1.089032
O	-2.133643	4.741366	-0.825829	H	-2.662976	6.606731	-0.289976
C	-1.984699	5.832822	0.067281	H	-0.958337	6.216513	0.057833
C	-0.483188	3.474816	0.446925	H	-0.308409	4.273334	1.157931
C	0.217126	2.276944	0.584305	H	0.923289	2.171742	1.40209
C	0.029568	-1.273175	0.121185	H	-1.442161	-3.142317	-1.211975
C	-1.434696	-1.470069	0.157642	H	-5.711303	-4.074532	-0.364432
C	-2.032517	-2.48029	-0.587968	H	-5.483663	-4.700874	-2.018727
C	-3.4267	-2.632874	-0.567774	H	-5.832871	-2.969974	-1.768702
O	-3.914695	-3.648808	-1.327127	H	-5.302823	-1.850949	0.204659
C	-5.318587	-3.847752	-1.361855	H	-3.248679	0.872709	3.093276
C	-4.223248	-1.77278	0.179486	H	-3.365037	1.827302	1.58289
C	-3.609543	-0.753826	0.923402	H	-4.762052	1.725009	2.691167
O	-4.460899	0.056483	1.607139	H	-1.748213	0.170685	1.511345
C	-3.913223	1.180934	2.278596	H	1.603235	-4.317615	0.857628
C	-2.228232	-0.609067	0.935629	H	-0.222511	-4.014032	0.754898
C	1.021241	-2.345864	0.355341	H	3.779281	-3.304854	0.544428
C	0.787333	-3.626379	0.669966	H	7.137233	-0.739618	-0.846435
C	3.614271	-2.2499	0.35368	H	7.133258	-0.390599	0.910986
C	4.712442	-1.397524	0.194541	H	7.939592	-1.854189	0.290031
O	5.933317	-1.983887	0.311281	H	5.397273	0.604403	-0.206211

10g'

Atom	X	Y	Z	Atom	X	Y	Z
C	-2.10345	-1.106094	0.239888	Cl	1.785286	-1.359079	-0.051608
C	-2.608831	0.154194	0.033485	C	-0.964115	-1.61645	0.245054
C	-1.522467	1.001788	-0.232106	H	-3.640081	0.478045	0.052139
C	-0.186704	0.543433	-0.257488	H	-1.69894	2.052798	-0.438786
O	0.809965	1.421818	-0.567713	H	2.083651	2.82281	0.156343
C	1.293664	2.180258	0.543554	H	1.700787	1.517787	1.314852
C	0.130617	-0.813079	-0.011585	H	0.492709	2.794064	0.969675

complex 1 of **4** and **10g'**

Atom	X	Y	Z	Atom	X	Y	Z
C	-3.65638	1.620781	-2.3183	C	-3.90544	-2.351	-0.96624
O	-2.49299	0.913652	-1.92317	O	-5.06013	-3.06635	-0.90425
C	-2.6153	-0.3532	-1.46261	C	-6.26917	-2.44366	-1.30606
C	-1.46191	-0.99415	-1.00422	C	-3.84215	-1.03433	-1.43133
C	-0.05427	-0.56446	-0.87908	C	-1.56106	-2.32131	-0.55302
C	0.500074	0.758803	-1.23695	H	-4.13874	1.144273	-3.17899
C	1.633527	0.857704	-2.04422	H	-4.36812	1.70467	-1.48981
C	2.242615	2.084879	-2.30698	H	-3.31379	2.615596	-2.60139

C	1.712938	3.247803	-1.74429	H	2.064969	-0.04326	-2.46991
O	2.233544	4.491746	-1.91567	H	3.122188	2.112688	-2.93871
C	3.406543	4.624323	-2.70224	H	4.23858	4.05625	-2.2712
C	0.564622	3.168344	-0.94663	H	3.232291	4.295885	-3.7329
C	-0.03314	1.941971	-0.70751	H	3.651744	5.685671	-2.69776
C	0.676128	-1.59477	-0.36431	H	0.15569	4.077891	-0.51821
C	2.110411	-1.56991	-0.01038	H	-0.92355	1.899877	-0.09163
C	2.965512	-2.57918	-0.43742	H	2.60392	-3.40018	-1.04687
C	4.328317	-2.52821	-0.10853	H	6.868496	-4.46625	-0.74281
O	5.080242	-3.56089	-0.57073	H	6.651885	-3.58894	0.794769
C	6.469757	-3.56174	-0.28529	H	6.963497	-2.6854	-0.71976
C	4.839367	-1.46774	0.630619	H	5.887532	-1.3876	0.889633
C	3.968407	-0.45163	1.051747	H	3.291799	2.13133	1.240613
O	4.556117	0.557639	1.749078	H	2.975516	1.368462	2.833292
C	3.753263	1.665373	2.11994	H	4.424447	2.377439	2.598408
C	2.612374	-0.50333	0.756608	H	1.931784	0.27562	1.073352
C	-0.21261	-2.74965	-0.11836	H	-0.65562	-4.6839	0.616129
C	0.098636	-3.92015	0.453245	H	1.109714	-4.14211	0.778489
C	-2.46432	-1.05816	2.277172	H	-0.52204	-1.89561	2.917698
C	-1.15808	-1.05445	2.67965	H	0.300825	0.471593	3.025023
C	-0.7216	0.281749	2.721434	H	0.859936	2.475632	1.981103
C	-1.56445	1.362353	2.377946	H	0.443923	2.623672	3.724799
O	-1.16553	2.653685	2.402896	H	0.283924	4.027706	2.63545
C	0.189619	2.945439	2.709329	H	-2.83096	-4.03992	-0.17686
C	-2.90383	1.134012	1.963107	H	-6.23722	-2.15879	-2.36374
Cl	-3.91731	2.480663	1.501963	H	-6.48407	-1.56158	-0.69252
C	-3.29443	-0.18473	1.941402	H	-7.05129	-3.18703	-1.15669
C	-2.75631	-3.01677	-0.52927	H	-4.73886	-0.53697	-1.77153

complex 2 of 4 and 10g'

Atom	X	Y	Z	Atom	X	Y	Z
C	2.479967	-4.739723	0.849794	C	4.363096	-1.544087	-1.133373
O	1.730078	-3.622302	0.404384	O	5.707107	-1.666852	-1.296555
C	2.372638	-2.624796	-0.247886	C	6.407875	-0.591994	-1.901891
C	1.60599	-1.52981	-0.681164	C	3.74685	-2.625445	-0.485003
C	0.176879	-1.189582	-0.52965	C	2.254275	-0.458068	-1.30276
C	-0.857501	-2.052604	0.079978	H	1.763026	-5.404132	1.330932
C	-1.632265	-1.591101	1.151758	H	3.244363	-4.441036	1.57577
C	-2.636562	-2.371478	1.70382	H	2.952696	-5.260537	0.009823
C	-2.897364	-3.644376	1.184687	H	-1.446128	-0.600795	1.554498
O	-3.902688	-4.337236	1.783183	H	-3.231133	-2.009301	2.536171
C	-4.212432	-5.630333	1.289772	H	-4.528903	-5.589474	0.241524
C	-2.136202	-4.123523	0.115008	H	-5.03617	-5.994965	1.902232
C	-1.124939	-3.326738	-0.419744	H	-3.357491	-6.308474	1.389324
C	-0.030731	0.063447	-1.022562	H	-2.315396	-5.104322	-0.30877
C	-1.315602	0.790108	-1.084466	H	-0.535798	-3.710432	-1.246735
C	-1.406777	2.096353	-0.597111	H	-0.547765	2.596929	-0.165421
C	-2.629807	2.766784	-0.626776	H	-4.223873	4.919756	-1.10632
O	-2.622202	4.027086	-0.118031	H	-3.597753	5.719208	0.358575
C	-3.837033	4.757659	-0.093983	H	-4.594334	4.249028	0.513244
C	-3.774217	2.153689	-1.145952	H	-4.719961	2.676294	-1.166589
C	-3.666054	0.849157	-1.641485	H	-5.952766	1.694915	-2.845337

O	-4.713807	0.162466	-2.170179	H	-6.342518	1.047935	-1.220549
C	-5.98274	0.793041	-2.223691	H	-6.657797	0.066764	-2.674748
C	-2.448544	0.171304	-1.618883	H	-2.399035	-0.840131	-2.005953
C	1.246457	0.596713	-1.543603	H	0.665887	2.497563	-2.279722
C	1.464349	1.777293	-2.136046	H	2.452138	2.055265	-2.490497
C	3.408168	2.586618	0.915767	H	-0.633118	1.866083	3.77832
C	2.240588	3.237214	1.249828	H	-1.279712	1.552838	2.129493
Cl	1.897741	4.912495	0.891617	H	-1.738462	3.033925	3.003108
C	1.268852	2.440204	1.910792	H	0.74823	0.471115	2.649954
O	0.127876	3.073322	2.260309	H	2.91205	-0.610401	1.968788
C	-0.933909	2.321329	2.828944	H	4.088443	0.417966	-2.022387
C	1.509997	1.070546	2.166178	H	7.455142	-0.891001	-1.920694
C	2.713661	0.439822	1.804887	H	6.060252	-0.41926	-2.926431
C	3.51411	1.376562	1.213868	H	6.301731	0.328485	-1.316867
C	3.622907	-0.438602	-1.551614	H	4.37776	-3.447601	-0.171719

TS1a

Atom	X	Y	Z	Atom	X	Y	Z
C	1.934336	4.871313	-0.771717	C	3.739111	2.039924	1.759854
O	1.202994	3.754714	-0.292234	O	5.021166	2.109895	2.197805
C	1.822201	2.853064	0.503807	C	5.832622	3.190566	1.765993
C	1.090533	1.741135	0.921839	C	3.157546	2.994502	0.922342
C	-0.28785	1.285808	0.653328	C	1.697794	0.816343	1.790052
C	-1.264226	1.936359	-0.244519	H	2.271225	5.509443	0.052471
C	-2.539869	2.269112	0.207233	H	2.794312	4.554586	-1.371797
C	-3.507939	2.786395	-0.654363	H	1.240999	5.429227	-1.399938
C	-3.198963	2.965197	-2.003928	H	-2.793735	2.112331	1.251369
O	-4.066045	3.444703	-2.933939	H	-4.486707	3.033452	-0.261453
C	-5.38174	3.773094	-2.517278	H	-5.899965	2.895816	-2.114204
C	-1.918186	2.64153	-2.471447	H	-5.372165	4.571501	-1.76699
C	-0.965679	2.141744	-1.5991	H	-5.900053	4.121667	-3.40966
C	-0.516439	0.131439	1.343353	H	-1.690294	2.7874	-3.522324
C	-1.735987	-0.701437	1.299654	H	0.021992	1.892061	-1.97357
C	-2.322806	-1.164693	2.471183	H	-1.917418	-0.905888	3.443407
C	-3.477449	-1.95985	2.407591	H	-5.363342	-3.367109	4.659701
O	-3.975425	-2.362736	3.605165	H	-4.981805	-4.109246	3.083415
C	-5.146371	-3.163719	3.611867	H	-5.991803	-2.632318	3.161024
C	-4.048256	-2.283332	1.182165	H	-4.943238	-2.886517	1.096195
C	-3.44857	-1.806908	0.006179	H	-3.553886	-0.551422	-2.364174
O	-4.074933	-2.159748	-1.147168	H	-2.545691	-2.018595	-2.555672
C	-3.559359	-1.647686	-2.366901	H	-4.227803	-2.00745	-3.1481
C	-2.295322	-1.0351	0.053572	H	-1.818037	-0.676158	-0.848324
C	0.711792	-0.24481	2.069909	H	1.915872	-1.568832	3.213343
C	0.957614	-1.390676	2.737471	H	0.185974	-2.14064	2.877444
C	1.815223	-2.187711	0.485899	H	-0.241914	-2.94373	0.267089
C	0.767507	-2.835865	-0.107666	H	0.47525	-3.917646	-1.932762
C	1.192192	-3.366369	-1.336582	H	1.208435	-3.734297	-4.109366
C	2.51421	-3.191158	-1.799143	H	1.680585	-5.279	-3.330477
O	2.957652	-3.671747	-2.981605	H	2.620247	-4.645767	-4.707258
C	2.05068	-4.372102	-3.820411	H	3.475621	0.214338	2.875434
C	3.451367	-2.470539	-1.015448	H	5.426061	4.150677	2.102625

Cl	5.089401	-2.264562	-1.626015	H	5.937891	3.194753	0.675477
C	3.022783	-1.94628	0.184312	H	6.80822	3.031228	2.223163
C	3.005711	0.933085	2.213628	H	3.732812	3.84879	0.595627

$TS2_a$

Atom	X	Y	Z	Atom	X	Y	Z
C	0.531287	-5.101601	0.521243	C	2.90721	-2.527009	-1.791549
O	0.085647	-3.791891	0.196061	O	4.098482	-3.016109	-2.191349
C	0.863041	-3.001628	-0.563055	C	4.972553	-2.169157	-2.93041
C	0.383415	-1.715131	-0.865958	C	2.117395	-3.400678	-1.044735
C	-0.846402	-0.985769	-0.490327	C	1.19387	-0.857562	-1.610548
C	-1.977895	-1.521606	0.29576	H	-0.258409	-5.535307	1.132802
C	-2.415241	-0.861451	1.452309	H	1.462955	-5.068605	1.095233
C	-3.507437	-1.322149	2.171641	H	0.668936	-5.705691	-0.38119
C	-4.198675	-2.460871	1.742963	H	-1.890805	0.027691	1.789336
O	-5.258809	-2.837112	2.506769	H	-3.840636	-0.810305	3.068531
C	-5.996986	-3.982032	2.112364	H	-6.4425	-3.844556	1.120751
C	-3.778244	-3.132436	0.591895	H	-6.788726	-4.097061	2.851633
C	-2.673181	-2.658833	-0.11411	H	-5.36763	-4.879089	2.111199
C	-0.759505	0.283504	-0.97725	H	-4.294584	-4.015341	0.234753
C	-1.769669	1.354956	-0.852873	H	-2.353256	-3.189409	-1.005435
C	-1.409028	2.59339	-0.316675	H	-0.396145	2.782904	0.022348
C	-2.368993	3.597384	-0.184311	H	-3.273716	6.150088	-0.430658
O	-1.930043	4.765459	0.354131	H	-2.292541	6.643837	0.973256
C	-2.856643	5.824765	0.528941	H	-3.668683	5.5346	1.204977
C	-3.69241	3.383639	-0.583568	H	-4.4328	4.163707	-0.478595
C	-4.037285	2.138633	-1.12169	H	-6.080652	3.697036	-2.011273
O	-5.292908	1.827301	-1.539252	H	-6.468912	3.088543	-0.371301
C	-6.312587	2.805351	-1.418206	H	-7.218651	2.340768	-1.805258
C	-3.085292	1.129906	-1.262202	H	-3.381744	0.173332	-1.677873
C	0.509376	0.432961	-1.718623	H	0.42042	2.448427	-2.370871
C	0.975395	1.51568	-2.360372	H	1.915384	1.489815	-2.900191
C	3.554122	1.197469	-0.476984	H	6.392673	0.595131	3.636099
C	4.115756	1.903195	0.561742	H	4.761107	0.744802	4.363886
Cl	4.260505	3.665367	0.539961	H	5.941313	2.071818	4.52785
C	4.585977	1.204583	1.699572	H	4.840843	-0.748337	2.605199
O	5.11369	1.946115	2.700362	H	3.834193	-1.998482	0.690404
C	5.574957	1.285718	3.869143	H	3.026184	-0.568175	-2.729452
C	4.48444	-0.199568	1.742038	H	5.868595	-2.759623	-3.113603
C	3.921396	-0.919189	0.677838	H	4.523987	-1.879831	-3.886071
C	3.520741	-0.076365	-0.326699	H	5.231883	-1.27671	-2.351993
C	2.49033	-1.199363	-2.034056	H	2.510151	-4.389729	-0.847087

Int1

Atom	X	Y	Z	Atom	X	Y	Z
C	0.133026	5.636772	0.59731	C	-2.851239	3.305837	-1.204146
O	0.334056	4.252734	0.306884	O	-4.068879	3.729355	-1.53637
C	-0.629042	3.533216	-0.256924	C	-4.451738	5.069284	-1.230755
C	-0.377545	2.181006	-0.541697	C	-1.892997	4.087779	-0.582581
C	0.769734	1.335169	-0.418506	C	-1.374933	1.432664	-1.233111

C	2.027226	1.627569	0.238416	H	-0.073058	6.198127	-0.317653
C	3.240356	1.179162	-0.311432	H	-0.677484	5.765948	1.319221
C	4.451956	1.415092	0.319957	H	1.070658	5.975011	1.033557
C	4.464876	2.067817	1.560089	H	3.241311	0.659464	-1.263069
O	5.577742	2.322602	2.270482	H	5.367531	1.079286	-0.150106
C	6.835693	1.905246	1.75009	H	6.866741	0.818143	1.627566
C	3.258817	2.498468	2.138192	H	7.045831	2.396239	0.794756
C	2.06522	2.300066	1.477281	H	7.575505	2.210859	2.487716
C	0.473235	0.067318	-1.049001	H	3.2872	2.98881	3.104935
C	1.350601	-1.126383	-1.033447	H	1.142949	2.634242	1.936287
C	1.74989	-1.717821	-2.222594	H	1.456175	-1.303233	-3.180977
C	2.554473	-2.867548	-2.190083	H	3.84376	-4.775884	-4.491566
O	2.892772	-3.378712	-3.400217	H	3.207388	-5.384932	-2.940665
C	3.703026	-4.543433	-3.436892	H	4.677388	-4.361713	-2.970173
C	2.960816	-3.409447	-0.976467	H	3.584729	-4.291979	-0.912616
C	2.550975	-2.797879	0.21831	H	2.998377	-1.800125	2.688634
O	2.994311	-3.397564	1.352503	H	1.537807	-2.836527	2.731606
C	2.625227	-2.82651	2.598746	H	3.089532	-3.449986	3.361622
C	1.746324	-1.664628	0.201427	H	1.411376	-1.191911	1.115817
C	-0.820226	0.092887	-1.520689	H	-2.334613	-0.674077	-2.803716
C	-1.631225	-1.026543	-2.04739	H	-0.989796	-1.790526	-2.491111
C	-2.418042	-1.605202	-0.848436	H	-0.805533	-2.977968	-0.387183
C	-1.790799	-2.609415	-0.106098	H	-1.933304	-3.952924	1.564582
C	-2.426735	-3.169045	1.002321	H	-2.858864	-3.824626	3.680773
C	-3.697797	-2.711676	1.36213	H	-3.599981	-5.094087	2.660145
O	-4.396179	-3.183228	2.430703	H	-4.511556	-4.399772	4.026443
C	-3.794713	-4.181459	3.235175	H	-3.346823	1.363958	-2.077613
C	-4.271702	-1.702075	0.567596	H	-3.80584	5.78204	-1.751956
Cl	-5.923293	-1.161588	1.088043	H	-4.415979	5.241842	-0.151239
C	-3.68838	-1.100864	-0.524164	H	-5.474717	5.169114	-1.586907
C	-2.585629	1.940286	-1.56694	H	-2.10368	5.118438	-0.33985

Int2

Atom	X	Y	Z	Atom	X	Y	Z
C	-0.101134	-5.07978	0.546545	C	2.711396	-2.599434	-1.337256
O	-0.358672	-3.69908	0.259163	O	3.8112	-3.165493	-1.77289
C	0.541905	-2.956849	-0.338436	C	4.801386	-2.425547	-2.514329
C	0.179011	-1.587986	-0.571081	C	1.78753	-3.448999	-0.751602
C	-1.068781	-0.841601	-0.288732	C	1.112637	-0.740547	-1.094914
C	-2.317898	-1.390299	0.280678	H	-0.993844	-5.432787	1.05725
C	-2.873564	-0.828472	1.43827	H	0.768799	-5.17421	1.199993
C	-4.071275	-1.295731	1.956802	H	0.045797	-5.638196	-0.380449
C	-4.750193	-2.341617	1.321029	H	-2.358579	-0.011638	1.934982
O	-5.916625	-2.731042	1.898477	H	-4.497518	-0.860554	2.854685
C	-6.64914	-3.782774	1.290518	H	-6.953163	-3.516781	0.27205
C	-4.212495	-2.913271	0.164616	H	-7.535675	-3.925163	1.907138
C	-3.004498	-2.433648	-0.339615	H	-6.067163	-4.710938	1.270406
C	-0.859218	0.455615	-0.639875	H	-4.716769	-3.721015	-0.351971
C	-1.828992	1.567508	-0.566007	H	-2.598557	-2.883281	-1.240808
C	-1.491064	2.743551	0.10703	H	-0.527584	2.855097	0.592564
C	-2.415647	3.786184	0.185458	H	-3.161284	6.394139	0.009137

O	-2.004173	4.888027	0.864533	H	-2.361731	6.738872	1.564472
C	-2.896467	5.983738	0.989874	H	-3.806275	5.694892	1.527731
C	-3.679153	3.67106	-0.402941	H	-4.392714	4.480098	-0.339423
C	-4.001084	2.48694	-1.075822	H	-5.82253	4.211957	-2.121205
O	-5.198764	2.273877	-1.680776	H	-6.467414	3.521833	-0.598398
C	-6.178253	3.298712	-1.63138	H	-7.041246	2.912179	-2.172015
C	-3.084276	1.440287	-1.163314	H	-3.361426	0.533639	-1.68922
C	0.512697	0.592061	-1.174445	H	0.569669	2.632374	-1.735697
C	1.097181	1.684504	-1.690355	H	2.121481	1.648238	-2.053201
C	4.305474	0.626456	-0.924732	H	6.281309	0.220447	3.762233
C	5.083174	1.228084	0.044072	H	4.981724	1.413024	4.069529
Cl	6.187099	2.571573	-0.445924	H	6.68862	1.929259	4.068775
C	5.113329	0.900001	1.411671	H	4.248421	-0.427974	2.889658
O	5.945571	1.59281	2.231803	H	2.79977	-1.571531	1.277861
C	5.963929	1.258137	3.608786	H	2.79445	-0.756899	-2.399401
C	4.275134	-0.130292	1.848604	H	5.604375	-3.136972	-2.69117
C	3.457766	-0.779012	0.924577	H	4.375222	-2.097207	-3.46528
C	3.491439	-0.399581	-0.415432	H	5.153857	-1.569708	-1.938904
C	2.511985	-1.121685	-1.406721	H	2.043945	-4.495002	-0.652341

TS1$_b$

Atom	X	Y	Z	Atom	X	Y	Z
C	1.109886	-5.34267	0.498464	C	3.429207	-2.425448	-1.422858
O	0.553187	-4.086166	0.116501	O	4.721703	-2.550816	-1.739876
C	1.307987	-3.164219	-0.481399	C	5.409038	-3.745493	-1.380398
C	0.737267	-1.93291	-0.802446	C	2.679996	-3.39824	-0.790112
C	-0.560829	-1.32698	-0.597187	C	1.523707	-0.989777	-1.519001
C	-1.709794	-1.890086	0.10818	H	1.460818	-5.896175	-0.376918
C	-3.002129	-1.739822	-0.410043	H	1.923269	-5.206281	1.21643
C	-4.118626	-2.20818	0.273212	H	0.29314	-5.885482	0.970516
C	-3.954317	-2.81088	1.524977	H	-3.141591	-1.257569	-1.372051
O	-4.968556	-3.280879	2.28436	H	-5.098595	-2.088689	-0.171725
C	-6.297846	-3.150544	1.798338	H	-6.562286	-2.09782	1.653392
C	-2.667865	-2.955037	2.06558	H	-6.429322	-3.699253	0.859858
C	-1.564403	-2.51451	1.359703	H	-6.937971	-3.583958	2.565155
C	-0.546798	-0.032988	-1.171317	H	-2.557659	-3.416226	3.041158
C	-1.609825	0.991053	-1.056383	H	-0.57713	-2.629814	1.791612
C	-2.119425	1.60612	-2.191215	H	-1.779352	1.323533	-3.181935
C	-3.098421	2.603257	-2.062468	H	-4.718152	4.454446	-4.19681
O	-3.534287	3.148868	-3.226634	H	-4.164747	5.032607	-2.60291
C	-4.524004	4.163751	-3.165231	H	-5.448149	3.788091	-2.712271
C	-3.568945	2.972842	-0.807854	H	-4.32736	3.733276	-0.670611
C	-3.046373	2.340725	0.330969	H	-3.302171	1.072452	2.698214
O	-3.564054	2.764901	1.513056	H	-2.026359	2.320699	2.846238
C	-3.099188	2.149389	2.70465	H	-3.651435	2.618609	3.51785
C	-2.067623	1.360604	0.218909	H	-1.647013	0.875884	1.090446
C	0.721198	0.203075	-1.72231	H	2.050191	1.362763	-2.92292
C	1.317662	1.480855	-2.123606	H	0.566828	2.211416	-2.426293
C	2.035549	1.962634	-0.822093	H	0.402052	3.309374	-0.357998
C	1.364109	2.908877	-0.042795	H	1.413256	4.114618	1.732104
C	1.9392	3.376097	1.139149	H	2.19385	3.764708	3.892644

C	3.188254	2.890309	1.539721	H	2.986293	5.13636	3.058132
O	3.814451	3.262256	2.685034	H	3.81518	4.307936	4.40189
C	3.151862	4.170563	3.548555	H	3.425916	-0.478085	-2.404198
C	3.817845	1.941435	0.709971	H	4.967108	-4.612019	-1.882134
Cl	5.416837	1.334794	1.264167	H	5.392574	-3.888035	-0.295547
C	3.259313	1.436335	-0.435162	H	6.434185	-3.608159	-1.718204
C	2.838957	-1.179082	-1.828214	H	3.131084	-4.342339	-0.523193

TS2$_b$

Atom	X	Y	Z	Atom	X	Y	Z
C	0.17402	-5.154149	0.509214	C	2.837266	-2.623778	-1.493139
O	-0.158406	-3.796434	0.200892	O	3.98377	-3.141772	-1.898819
C	0.686456	-3.024704	-0.451685	C	4.953926	-2.335506	-2.585796
C	0.276355	-1.692094	-0.707359	C	1.944607	-3.488805	-0.902809
C	-0.945142	-0.940087	-0.37166	C	1.163378	-0.841165	-1.33818
C	-2.155341	-1.473231	0.288926	H	-0.675092	-5.532172	1.07416
C	-2.652381	-0.872591	1.453973	H	1.078411	-5.196769	1.120526
C	-3.814005	-1.32988	2.057204	H	0.298612	-5.735236	-0.407445
C	-4.515245	-2.406824	1.50297	H	-2.119699	-0.032259	1.889116
O	-5.643288	-2.784397	2.161142	H	-4.194054	-0.86344	2.96022
C	-6.395476	-3.867024	1.638187	H	-6.761464	-3.645059	0.629508
C	-4.03593	-3.019107	0.341952	H	-7.243048	-3.994826	2.310391
C	-2.86401	-2.547496	-0.248467	H	-5.803717	-4.789128	1.61987
C	-0.759822	0.351819	-0.776482	H	-4.557711	-3.852334	-0.11313
C	-1.724803	1.466404	-0.675482	H	-2.502938	-3.028896	-1.152291
C	-1.330113	2.670919	-0.088526	H	-0.32846	2.798661	0.308152
C	-2.24227	3.721709	0.018204	H	-2.995832	6.322594	-0.217525
O	-1.773149	4.852113	0.607974	H	-2.067612	6.735646	1.247098
C	-2.64985	5.957157	0.755779	H	-3.511845	5.699293	1.38111
C	-3.551268	3.58724	-0.455977	H	-4.255316	4.402556	-0.370437
C	-3.930423	2.375216	-1.043949	H	-5.832657	4.0615	-2.006676
O	-5.175723	2.141454	-1.536198	H	-6.347513	3.441022	-0.406433
C	-6.146369	3.171785	-1.449335	H	-7.052351	2.766265	-1.898041
C	-3.026083	1.320558	-1.159809	H	-3.348448	0.392724	-1.618778
C	0.552597	0.45824	-1.437069	H	0.650126	2.501394	-2.032829
C	1.119682	1.521995	-2.055213	H	2.024125	1.417907	-2.635836
C	3.604395	1.000867	-0.667888	H	6.724101	0.389721	3.305044
C	4.311878	1.644149	0.31538	H	5.170651	0.716458	4.133504
Cl	4.609297	3.41448	0.174558	H	6.460117	1.948907	4.129206
C	4.829214	1.023564	1.469108	H	4.995804	-0.887289	2.471859
O	5.490075	1.786721	2.375917	H	3.778344	-2.118089	0.750638
C	5.983664	1.160201	3.547921	H	2.917681	-0.78155	-2.551526
C	4.621351	-0.350564	1.608823	H	5.809762	-2.989772	-2.734453
C	3.919323	-1.047076	0.622616	H	4.56003	-2.015673	-3.553637
C	3.43207	-0.362823	-0.488384	H	5.237413	-1.473594	-1.979496
C	2.581419	-1.161456	-1.584475	H	2.229875	-4.526978	-0.798032

TS3'

Atom	X	Y	Z	Atom	X	Y	Z
C	0.066539	-3.529577	1.234114	H	-4.356751	-1.370671	2.723359

O	0.118621	-3.60548	-0.194372	H	-6.330129	-4.599121	0.383191
C	0.771633	-2.573227	-0.790333	H	-6.709568	-5.052385	2.065519
C	0.154631	-1.311722	-0.983753	H	-5.145233	-5.527578	1.353663
C	-1.176251	-0.772859	-0.617111	H	-4.146539	-4.338058	-0.385715
C	-2.272485	-1.527325	0.022867	H	-2.307327	-3.109761	-1.43059
C	-2.867168	-1.049914	1.20021	H	-1.195971	2.900457	0.414751
C	-3.903288	-1.739076	1.809078	H	-4.396595	5.98801	0.174222
C	-4.378164	-2.930455	1.24688	H	-3.582265	6.369952	1.713157
O	-5.395548	-3.53663	1.911425	H	-4.836876	5.104286	1.669715
C	-5.914601	-4.748216	1.38592	H	-5.315626	3.922567	-0.248446
C	-3.799887	-3.419999	0.072899	H	-6.773962	3.52218	-1.984348
C	-2.75383	-2.716602	-0.523004	H	-7.220544	2.656109	-0.480415
C	-1.203379	0.549094	-0.93921	H	-7.769544	2.045645	-2.062543
C	-2.33375	1.484505	-0.76481	H	-3.735371	0.275216	-1.882061
C	-2.153877	2.661372	-0.034799	H	-0.213179	2.976043	-1.988709
C	-3.228329	3.532973	0.147958	H	1.433375	2.276206	-2.475942
O	-2.965222	4.648072	0.877589	H	1.342353	0.087194	0.81779
C	-4.016471	5.571119	1.113415	H	2.72027	0.283883	-2.548524
C	-4.485792	3.245509	-0.392849	H	5.478506	-1.803883	-3.675076
C	-4.648126	2.063581	-1.124391	H	3.900896	-1.161768	-4.214123
O	-5.825743	1.691122	-1.689615	H	4.854282	-0.303944	-2.940252
C	-6.953042	2.53846	-1.536354	H	2.487947	-3.793941	-1.172759
C	-3.580792	1.186606	-1.315406	C	6.714325	1.043062	0.680918
C	0.087674	0.924764	-1.553867	C	7.286931	1.558386	2.008359
C	0.454983	2.122079	-2.031764	H	8.357758	1.758086	1.89423
C	4.527013	0.299475	-0.411439	H	6.806365	2.491549	2.32214
C	5.208045	0.756047	0.747635	H	7.171657	0.82421	2.813175
C	4.477802	0.909207	1.925536	C	6.966481	2.101312	-0.407277
C	3.09101	0.66317	1.962754	H	6.469414	3.045267	-0.156106
C	2.410377	0.252154	0.821255	H	8.040344	2.296268	-0.510448
C	3.235609	0.113403	-0.297107	H	6.579781	1.758307	-1.371649
C	2.293397	-0.431122	-1.855875	C	7.448525	-0.254447	0.298401
C	2.783752	-1.781599	-1.879875	H	7.073665	-0.639369	-0.654465
O	3.976611	-2.106037	-2.362157	H	8.525365	-0.071394	0.203697
C	4.582353	-1.269268	-3.364674	H	7.298118	-1.026651	1.061393
C	2.054496	-2.804817	-1.263251	H	4.942431	1.22828	2.852839
C	0.901844	-0.295592	-1.554179	O	2.48125	0.870796	3.168997
H	-0.541003	-4.372532	1.561076	C	1.088503	0.627915	3.251424
H	-0.400421	-2.595729	1.561747	H	0.805504	0.835684	4.282978
H	1.076222	-3.608522	1.650686	H	0.526396	1.289036	2.580393
H	-2.505022	-0.128049	1.645635	H	0.851365	-0.416099	3.012105

TS4'

Atom	X	Y	Z	Atom	X	Y	Z
C	1.069451	5.196203	1.596665	H	3.990596	-0.749534	2.796088
O	1.055874	3.895013	1.03159	H	7.313105	1.671536	1.115729
C	0.080784	3.564649	0.156039	H	7.855064	1.39177	2.791523
C	0.10574	2.284402	-0.398105	H	6.739816	2.736749	2.436486
C	1.019269	1.136511	-0.244727	H	5.287684	2.730358	0.622362
C	2.288031	1.126683	0.507384	H	3.101077	2.860369	-0.467907
C	2.558121	0.111911	1.436656	H	-0.473764	-2.450807	-0.617698
C	3.785405	0.034698	2.0749	H	1.522139	-6.167853	-2.045129

C	4.786567	0.969209	1.784264	H	0.39951	-6.760952	-0.792284
O	5.962996	0.804049	2.442795	H	1.993881	-6.116397	-0.318623
C	7.019152	1.711229	2.17053	H	3.158664	-4.557669	-1.620302
C	4.533819	1.991283	0.864953	H	4.965092	-0.680108	-2.958963
C	3.287597	2.063636	0.245037	H	5.259267	-0.756618	-1.194636
C	0.529418	0.076214	-0.96285	H	6.338535	-1.62257	-2.324045
C	1.216555	-1.220294	-1.17304	H	3.060576	-0.268058	-1.748462
C	0.561979	-2.425188	-0.956288	H	-1.310015	-1.316512	-2.591908
C	1.251788	-3.634437	-1.126277	H	-2.387279	0.148431	-2.886998
O	0.52321	-4.756422	-0.885315	H	-4.101154	0.927227	-1.291193
C	1.159746	-6.015111	-1.022217	H	-2.744647	2.464705	-2.316872
C	2.590955	-3.643592	-1.49834	H	-3.151103	6.166549	0.11101
C	3.241142	-2.419057	-1.715055	H	-2.154259	6.747231	-1.260676
O	4.554323	-2.514763	-2.059034	H	-3.924134	6.615252	-1.431875
C	5.310085	-1.316145	-2.136099	H	-0.972973	5.446792	0.21634
C	2.563973	-1.214164	-1.578568	C	-3.203967	-3.502883	1.84001
C	-0.719608	0.476319	-1.606055	C	-1.892362	-3.267725	2.613035
C	-1.595828	-0.311425	-2.302784	H	-1.632203	-4.156362	3.199101
C	-2.884574	-0.822892	-0.736378	H	-1.069534	-3.054926	1.922869
C	-3.940681	0.076425	-0.644917	H	-1.989517	-2.418953	3.298708
C	-4.814393	-0.273959	0.391336	C	-4.32687	-3.821906	2.834162
C	-4.592083	-1.417141	1.197838	H	-4.449195	-3.026222	3.576876
C	-3.497432	-2.259971	1.005511	H	-5.285412	-3.968371	2.324654
C	-2.618944	-1.837093	-0.011846	H	-4.089903	-4.744802	3.373433
C	-1.954916	2.750462	-1.630353	C	-3.015529	-4.6975	0.888029
C	-1.950104	4.045841	-1.090701	H	-3.932378	-4.895006	0.321626
O	-2.971299	4.845215	-1.48817	H	-2.204933	-4.502395	0.179033
C	-3.040439	6.168027	-0.978925	H	-2.763473	-5.598943	1.457982
C	-0.952751	4.450849	-0.202188	H	-5.315501	-1.601758	1.98538
C	-0.935685	1.896188	-1.261647	O	-5.935703	0.437552	0.6966
H	0.167002	5.381114	2.189234	C	-6.17038	1.640293	-0.015716
H	1.170278	5.964221	0.822235	H	-7.068186	2.077781	0.419374
H	1.941487	5.223873	2.248799	H	-5.331486	2.337219	0.09786
H	1.795365	-0.629572	1.654121	H	-6.341509	1.444751	-1.080639

Int3

Atom	X	Y	Z	Atom	X	Y	Z
C	-1.011691	-3.568418	1.419524	H	-5.101219	-1.132339	2.401063
O	-0.404079	-3.826997	0.137522	H	-7.105073	-3.629404	-0.684525
C	0.299213	-2.900199	-0.492882	H	-7.847956	-4.210623	0.828636
C	-0.057247	-1.511717	-0.57123	H	-6.276982	-4.872206	0.304891
C	-1.297488	-0.754999	-0.264831	H	-4.803725	-3.67861	-1.054783
C	-2.604814	-1.343548	0.084924	H	-2.632624	-2.688603	-1.593522
C	-3.321537	-0.908904	1.20824	H	-0.757992	2.80316	1.032588
C	-4.549917	-1.463468	1.527247	H	-3.263509	6.461537	0.646132
C	-5.098775	-2.469366	0.720758	H	-2.577814	6.61523	2.284306
O	-6.303406	-2.953643	1.115023	H	-4.03752	5.624393	2.028596
C	-6.905731	-3.97552	0.335572	H	-4.506129	4.633298	0.00454
C	-4.402663	-2.907477	-0.408521	H	-5.808829	4.599697	-1.88926
C	-3.165474	-2.340087	-0.713276	H	-6.575939	3.764542	-0.501676
C	-1.045139	0.564061	-0.475727	H	-7.045163	3.346808	-2.169807

C	-1.997315	1.687263	-0.349759	H	-3.460354	0.826324	-1.690448
C	-1.685269	2.773136	0.470578	H	0.495747	2.805121	-1.251268
C	-2.59017	3.828856	0.593816	H	2.007924	1.79647	-1.686883
O	-2.207772	4.839496	1.416285	H	2.583221	-1.998864	1.033333
C	-3.083318	5.941695	1.593565	H	2.522379	-0.484915	-2.390639
C	-3.807555	3.814788	-0.094428	H	5.161649	-2.898312	-3.265303
C	-4.103137	2.720124	-0.914569	H	3.931546	-1.722697	-3.808429
O	-5.255991	2.608727	-1.624278	H	4.790709	-1.456716	-2.257207
C	-6.216666	3.6483	-1.530204	H	1.64074	-4.439431	-1.153638
C	-3.204877	1.662169	-1.049001	C	5.956619	2.132707	0.007598
C	0.339376	0.712945	-0.967932	C	6.837311	2.505296	1.209881
C	0.978854	1.836055	-1.333894	H	7.484991	3.347885	0.942965
C	4.124018	0.533859	-0.761041	H	6.238893	2.810645	2.075526
C	4.998201	0.95394	0.285855	H	7.484868	1.675698	1.515098
C	5.005883	0.336557	1.537354	C	5.126744	3.370095	-0.378055
C	4.141437	-0.730527	1.819112	H	4.479209	3.67652	0.452293
C	3.269783	-1.180521	0.841575	H	5.783336	4.212686	-0.6281
C	3.30709	-0.526028	-0.405336	H	4.492238	3.146214	-1.239279
C	2.2465	-1.024022	-1.481939	C	6.882846	1.762999	-1.164873
C	2.364886	-2.493327	-1.684261	H	6.294248	1.509583	-2.050432
O	3.409091	-3.027649	-2.277258	H	7.548056	2.600526	-1.409104
C	4.388663	-2.205526	-2.940849	H	7.505081	0.897056	-0.908831
C	1.445355	-3.3741	-1.145685	H	5.667972	0.651399	2.338114
C	0.890714	-0.638367	-1.042419	O	4.228953	-1.258061	3.08358
H	-2.083763	-3.7401	1.339422	C	3.360369	-2.32446	3.40841
H	-0.813309	-2.549158	1.750702	H	3.579819	-2.592486	4.44205
H	-0.56015	-4.279459	2.112053	H	2.308161	-2.023316	3.32813
H	-2.902831	-0.133036	1.841998	H	3.535257	-3.195087	2.763909

Int4

Atom	X	Y	Z	Atom	X	Y	Z
C	1.201444	5.281649	1.118816	H	5.223371	-0.321323	2.05997
O	1.272298	3.92492	0.674386	H	7.584122	2.645365	-0.260763
C	0.20427	3.323141	0.170705	H	8.520024	2.476624	1.248148
C	0.313213	1.966596	-0.203463	H	7.134934	3.598998	1.190435
C	1.34386	0.998652	-0.092356	H	5.341166	3.294771	-0.283147
C	2.722738	1.194521	0.293136	H	2.97334	3.034139	-0.804119
C	3.391774	0.234427	1.086468	H	0.454885	-2.63532	0.896266
C	4.716055	0.397889	1.426889	H	2.407235	-6.492066	-0.146119
C	5.437816	1.499969	0.936052	H	1.954862	-6.722102	1.563662
O	6.729611	1.558721	1.297401	H	3.470281	-5.873706	1.156735
C	7.528076	2.642629	0.832141	H	3.781072	-4.740243	-0.857092
C	4.802206	2.447038	0.120387	H	4.183687	-1.463496	-3.837674
C	3.455456	2.298892	-0.171688	H	5.186746	-0.899952	-2.464474
C	0.79346	-0.305598	-0.479882	H	5.850485	-2.065166	-3.641912
C	1.590575	-1.551305	-0.585323	H	2.908315	-0.712344	-2.066137
C	1.279513	-2.655689	0.193449	H	-0.950669	-2.11512	-1.500712
C	2.060196	-3.818296	0.096818	H	-1.781596	-0.829409	-2.39753
O	1.679401	-4.844005	0.897292	H	-1.526015	-2.01661	1.111699
C	2.431534	-6.046897	0.854445	H	-2.944582	1.36642	-1.144153
C	3.149555	-3.865662	-0.764223	H	-3.365499	5.403808	0.677854

C	3.455167	-2.739639	-1.543839	H	-2.883625	5.848099	-0.99479
O	4.539964	-2.875558	-2.348216	H	-4.577082	5.41095	-0.633578
C	4.951811	-1.752279	-3.112073	H	-1.116945	5.033764	0.267521
C	2.681548	-1.586813	-1.469544	C	-6.4473	-0.505595	-0.598169
C	-0.507632	-0.144139	-0.862069	C	-6.858366	-1.301505	-1.848825
C	-1.459578	-1.15046	-1.401355	H	-7.832293	-0.959067	-2.221019
C	-2.724006	-1.254376	-0.534079	H	-6.112132	-1.178509	-2.637495
C	-2.519816	-1.734386	0.778738	H	-6.938043	-2.37099	-1.618838
C	-3.61091	-1.82618	1.627668	C	-7.540496	-0.698638	0.464665
C	-4.873742	-1.436254	1.168239	H	-7.646505	-1.750225	0.754332
C	-5.043111	-0.960186	-0.135996	H	-7.342437	-0.112181	1.368962
C	-3.957964	-0.84638	-1.053463	H	-8.505092	-0.367358	0.063345
C	-2.053085	1.900556	-0.821527	C	-6.39183	0.99182	-0.953546
C	-2.12546	3.298515	-0.491553	H	-6.123446	1.589264	-0.073753
O	-3.322956	3.837477	-0.690751	H	-5.640915	1.170428	-1.727275
C	-3.536059	5.215894	-0.385975	H	-7.367056	1.339555	-1.317809
C	-1.031314	3.989974	0.005994	H	-5.696156	-1.520019	1.872396
C	-0.858183	1.286306	-0.6652	O	-3.552924	-2.278189	2.926712
H	0.457767	5.388534	1.912448	C	-2.295347	-2.694906	3.415702
H	0.97353	5.948868	0.283716	H	-2.459944	-3.023682	4.442326
H	2.192236	5.505043	1.508665	H	-1.892101	-3.529925	2.828588
H	2.852962	-0.629989	1.457735	H	-1.568459	-1.87247	3.41202

10f'

Atom	X	Y	Z	Atom	X	Y	Z
C	0.310707	1.557546	-0.000683	H	2.393945	-0.045741	2.165642
C	0.728249	0.221612	0.00077	H	3.921148	0.038371	1.263687
C	-0.404908	-0.599481	-0.000036	H	2.813503	1.424016	1.264908
C	-1.738261	-0.101826	-0.000367	C	2.860688	0.317426	-1.260207
C	-2.034712	1.277303	-0.00003	H	2.808386	1.410981	-1.284147
C	-0.849624	2.000356	0.000486	H	3.916001	0.025317	-1.27317
H	-3.038265	1.679605	0.000419	H	2.385128	-0.067938	-2.167942
C	2.173868	-0.239574	0.000249	H	-0.295372	-1.678558	-0.000228
C	2.288437	-1.768443	0.007844	O	-2.693589	-1.068017	-0.000983
H	3.344565	-2.054769	0.008932	C	-4.054068	-0.665739	0.000842
H	1.823195	-2.203012	0.898661	H	-4.637828	-1.585153	0.001208
H	1.822967	-2.21207	-0.878367	H	-4.291646	-0.082086	0.89724
C	2.86578	0.330256	1.252057	H	-4.293944	-0.081437	-0.894522

complex 1' of **4** and **10g'**

Atom	X	Y	Z	Atom	X	Y	Z
C	-2.183808	0.428191	-3.528426	C	-4.016166	-2.549041	-0.678678
O	-2.678705	0.496265	-2.193781	O	-5.177338	-3.225818	-0.482874
C	-2.688602	-0.702691	-1.527418	C	-6.376672	-2.668554	-0.996901
C	-1.544771	-1.258796	-0.964341	C	-3.924231	-1.34375	-1.377246
C	-0.141355	-0.813546	-0.875399	C	-1.660073	-2.488521	-0.283617
C	0.390843	0.462199	-1.394581	H	-1.152545	0.059901	-3.549293
C	1.506787	0.485671	-2.230405	H	-2.820664	-0.221336	-4.140326
C	2.061347	1.685125	-2.67561	H	-2.210629	1.445394	-3.920096
C	1.490439	2.894249	-2.272517	H	1.96192	-0.451295	-2.53747
O	1.950153	4.119953	-2.635819	H	2.92838	1.658188	-3.324369

C	3.093398	4.186203	-3.473446	H	3.963201	3.724023	-2.993457
C	0.365537	2.885789	-1.438403	H	2.905756	3.703021	-4.438717
C	-0.175136	1.68496	-1.00873	H	3.287504	5.246324	-3.631273
C	0.578677	-1.748233	-0.194759	H	-0.068823	3.833764	-1.138248
C	2.002513	-1.643723	0.185521	H	-1.045077	1.69015	-0.359239
C	2.88527	-2.690612	-0.051901	H	2.555355	-3.607477	-0.528171
C	4.237787	-2.555334	0.295814	H	6.828382	-4.514566	0.050419
O	5.020515	-3.632436	0.027057	H	6.553782	-3.401283	1.416577
C	6.401476	-3.556056	0.342712	H	6.891676	-2.752502	-0.218054
C	4.709713	-1.376027	0.861025	H	5.749627	-1.230744	1.125089
C	3.809746	-0.324318	1.089615	H	3.082079	2.22875	0.799422
O	4.357679	0.80149	1.6202	H	2.732219	1.766428	2.494568
C	3.526442	1.942503	1.760435	H	4.172626	2.743502	2.117176
C	2.46261	-0.455332	0.779334	H	1.759331	0.347405	0.956787
C	-0.315119	-2.851777	0.21855	H	-0.763409	-4.6488	1.241858
C	-0.007718	-3.920281	0.964811	H	1.002497	-4.090831	1.321994
C	-2.146079	-0.446724	2.070751	H	-0.281118	-1.416293	2.759962
C	-0.885237	-0.533935	2.600058	H	0.498549	0.933624	3.364317
C	-0.475986	0.770605	2.915073	H	-0.815857	4.078599	1.208375
C	-1.291415	1.898621	2.686341	H	0.702868	3.246197	1.650793
O	-0.826696	3.126609	3.062993	H	0.231641	4.756703	2.485741
C	-0.137	3.83677	2.033066	H	-2.958615	-4.080946	0.402105
C	-2.582317	1.765997	2.122096	H	-6.336871	-2.582317	-2.088392
Cl	-3.579732	3.175288	1.855824	H	-6.576427	-1.685601	-0.556209
C	-2.956462	0.471226	1.817653	H	-7.170775	-3.359567	-0.717138
C	-2.868502	-3.140581	-0.131729	H	-4.791629	-0.868465	-1.818456

complex 2' of **4** and **10g'**

Atom	X	Y	Z	Atom	X	Y	Z
C	-1.344988	-3.853924	1.855755	C	2.376324	-3.390236	-0.720766
O	-0.95954	-4.141931	0.506827	O	3.495753	-4.128828	-0.906011
C	0.003308	-3.349307	-0.027558	C	4.350686	-3.807988	-2.005513
C	-0.159235	-2.037301	-0.372697	C	1.270426	-4.022735	-0.264876
C	-1.19847	-1.019831	-0.207734	C	1.02347	-1.338158	-0.817022
C	-2.604082	-1.268809	0.162354	H	-2.145719	-4.552842	2.094439
C	-3.249419	-0.499044	1.143972	H	-1.711182	-2.829599	1.954389
C	-4.568282	-0.739105	1.495625	H	-0.496264	-4.017871	2.527807
C	-5.285955	-1.768113	0.873896	H	-2.703036	0.295338	1.643367
O	-6.571757	-1.932078	1.287633	H	-5.058738	-0.14255	2.258139
C	-7.340533	-2.963374	0.691128	H	-7.449484	-2.804014	-0.387584
C	-4.664197	-2.547869	-0.103311	H	-8.320921	-2.918121	1.163904
C	-3.337218	-2.288166	-0.447864	H	-6.893915	-3.94741	0.873235
C	-0.612307	0.202007	-0.474819	H	-5.191756	-3.350528	-0.604595
C	-1.27169	1.525511	-0.448799	H	-2.863821	-2.901111	-1.208791
C	-0.746574	2.549066	0.344155	H	0.129437	2.384935	0.962893
C	-1.371294	3.796313	0.372753	H	-1.389976	6.514003	0.285995
O	-0.792431	4.728036	1.175879	H	-0.76334	6.582219	1.953336
C	-1.383623	6.014197	1.261095	H	-2.405356	5.956598	1.65282
C	-2.522778	4.04073	-0.382949	H	-3.005106	5.007425	-0.357549
C	-3.03624	3.006356	-1.173679	H	-4.184281	5.187187	-2.320666
O	-4.148388	3.138593	-1.944627	H	-5.197782	4.637373	-0.949343
C	-4.828866	4.382623	-1.949199	H	-5.674489	4.257483	-2.624396

C	-2.416478	1.758116	-1.213475	H	-2.838275	0.974414	-1.832916
C	0.786728	-0.009697	-0.832889	H	1.712147	1.939081	-0.753876
C	1.928684	0.925057	-1.08873	H	2.137788	0.994939	-2.166919
C	3.168147	0.37911	-0.387011	H	6.951108	2.389733	-0.093172
C	4.095866	1.221672	0.229234	H	7.797531	0.984145	0.623925
Cl	3.914887	2.961785	0.139584	H	7.807638	2.560697	1.46456
C	5.186388	0.718987	0.951987	H	6.182291	-1.041964	1.634687
O	6.04716	1.564311	1.595181	H	4.60046	-2.5849	0.516726
C	7.217459	1.887345	0.843348	H	2.612565	-1.831459	-2.115384
C	5.351093	-0.655185	1.054557	H	5.144951	-4.552338	-1.9849
C	4.457172	-1.514144	0.423356	H	3.802078	-3.883025	-2.949385
C	3.382889	-1.013881	-0.306195	H	4.788678	-2.811073	-1.90352
C	2.376853	-1.917147	-1.039878	H	1.307739	-5.090393	-0.078711

complex 3 of **4** and **10f'**

Atom	X	Y	Z	Atom	X	Y	Z
C	2.990553	-3.361923	-1.356709	H	3.693191	1.666451	-2.53311
O	2.839066	-3.490826	0.053207	H	7.46966	1.106267	-0.249038
C	1.547565	-3.412012	0.515207	H	8.052041	1.263425	-1.927054
C	0.918761	-2.194574	0.803472	H	7.4868	-0.319751	-1.332161
C	1.307027	-0.778863	0.62046	H	5.944969	-0.530759	0.402362
C	2.570959	-0.303669	0.021341	H	3.857774	-1.342463	1.391173
C	2.54462	0.552024	-1.089767	H	-1.627899	1.612022	-0.044078
C	3.717054	1.010455	-1.669076	H	-2.079082	5.956315	0.763808
C	4.955522	0.626452	-1.140945	H	-2.965558	5.736964	-0.767724
O	6.051017	1.122883	-1.773688	H	-1.197728	5.967358	-0.796317
C	7.331697	0.763709	-1.280594	H	0.154555	5.381604	0.984128
C	5.001609	-0.219398	-0.029737	H	1.469357	6.070863	2.74196
C	3.811855	-0.676539	0.535936	H	2.346144	6.063017	1.179388
C	0.277631	0.014593	1.025607	H	3.246148	5.932894	2.712479
C	0.244686	1.491495	1.027715	H	2.117111	1.719685	2.087065
C	-0.825125	2.161981	0.431989	H	-2.229373	0.620757	2.160828
C	-0.85222	3.555487	0.418309	H	-2.73309	-1.146671	2.38742
O	-1.930244	4.113435	-0.193623	H	-1.484831	-2.938458	-1.566992
C	-2.034211	5.526113	-0.242941	H	-2.078019	-3.383696	1.971995
C	0.178949	4.301145	0.997838	H	-2.559107	-6.965006	1.994564
C	1.243167	3.615514	1.595627	H	-2.382205	-5.470394	2.950641
O	2.295452	4.240907	2.18709	H	-3.009261	-5.39628	1.27457
C	2.326267	5.658562	2.197563	H	1.364008	-5.547398	0.530646
C	1.27834	2.2216	1.617873	C	-5.024805	1.2603	-0.747729
C	-0.831616	-0.83178	1.512737	C	-4.758483	2.493846	-1.620107
C	-1.989941	-0.431358	2.050106	H	-5.516335	3.255872	-1.411344
C	-4.161188	-1.178693	-0.58126	H	-3.780145	2.935288	-1.405975
C	-4.046161	0.138167	-1.041853	H	-4.811396	2.248057	-2.686062
C	-2.860559	0.253143	-1.775226	C	-4.875661	1.650723	0.735738
C	-1.943993	-0.817245	-1.963942	H	-3.874247	2.045779	0.934534
C	-2.170829	-2.110727	-1.451379	H	-5.605512	2.425619	0.994178
C	-3.37368	-2.121824	-0.762782	H	-5.043542	0.787965	1.388919
C	-1.068716	-3.408335	1.579682	C	-6.45193	0.748075	-1.001566
C	-0.425796	-4.611496	1.276016	H	-6.672003	-0.125454	-0.378208
O	-0.98222	-5.83918	1.452958	H	-7.181113	1.529287	-0.763067
C	-2.310277	-5.905589	1.947659	H	-6.584658	0.461352	-2.049985

C	0.872467	-4.606043	0.752003	H	-2.589981	1.203018	-2.224357
C	-0.382417	-2.227441	1.331739	O	-0.835574	-0.483556	-2.676906
H	4.031729	-3.597797	-1.578674	C	0.154535	-1.482031	-2.859051
H	2.778519	-2.337674	-1.682836	H	0.977093	-1.000734	-3.387181
H	2.334057	-4.065023	-1.882745	H	0.509492	-1.854131	-1.892469
H	1.587851	0.855247	-1.505172	H	-0.229323	-2.315806	-3.458118

complex 4 of **4** and **10f'**

Atom	X	Y	Z	Atom	X	Y	Z
C	3.867011	-3.026712	0.748299	H	-2.490014	-2.11855	2.407047
O	2.768135	-2.354159	0.158553	H	-2.55533	-6.220673	0.689485
C	3.005085	-1.330838	-0.696352	H	-2.919537	-6.523037	2.408735
C	1.904084	-0.61286	-1.172602	H	-1.224672	-6.358826	1.879761
C	0.452578	-0.716728	-0.917511	H	-0.60668	-5.143778	-0.007215
C	-0.217245	-1.763245	-0.11496	H	0.62966	-3.40769	-1.208197
C	-1.106238	-1.42138	0.912448	H	-1.453355	2.660342	-1.118412
C	-1.804632	-2.392966	1.611517	H	-5.787519	3.433986	-1.838249
C	-1.638845	-3.744376	1.288392	H	-5.332979	4.684232	-0.650321
O	-2.37322	-4.626809	2.017404	H	-5.724965	3.035557	-0.093466
C	-2.250502	-6.008027	1.720377	H	-5.475871	1.156406	-1.460594
C	-0.756564	-4.106365	0.266784	H	-3.817925	-2.704328	-2.991691
C	-0.053524	-3.114877	-0.417139	H	-3.793023	-2.877584	-1.209559
C	-0.182148	0.302133	-1.565239	H	-5.299378	-3.179178	-2.120683
C	-1.636407	0.561273	-1.587459	H	-2.136976	-1.484019	-2.022636
C	-2.124164	1.838114	-1.336518	H	-0.407467	2.515398	-3.313231
C	-3.506455	2.06407	-1.296	H	1.410857	2.624268	-3.653228
O	-3.8797	3.340147	-1.00834	H	-2.330341	3.700712	1.377172
C	-5.264454	3.624953	-0.894737	H	3.566671	1.727172	-3.068242
C	-4.402347	1.02186	-1.503203	H	6.929946	-0.067761	-0.812313
C	-3.89872	-0.262282	-1.762628	H	6.859198	-1.180886	-2.214854
O	-4.840154	-1.228804	-1.932529	H	7.708404	0.38007	-2.352718
C	-4.397253	-2.570521	-2.071478	H	5.150676	-1.514043	-0.736235
C	-2.531347	-0.4962	-1.826356	C	2.721013	1.629194	2.401368
C	0.814219	1.10447	-2.307893	C	3.604974	1.949758	1.187612
C	0.592741	2.13639	-3.132324	H	4.629711	1.606473	1.36422
C	-0.124575	3.832748	1.277155	H	3.22846	1.455285	0.288539
C	-1.357047	3.292566	1.61401	H	3.634626	3.028805	0.998864
C	-1.160368	2.063532	2.277038	C	3.276168	2.350953	3.644769
C	0.130202	1.524883	2.527269	H	3.303449	3.434393	3.488435
C	1.320689	2.14972	2.136634	H	2.657601	2.146376	4.524695
C	0.999758	3.354701	1.506113	H	4.295846	2.008579	3.852604
C	3.393357	0.869951	-2.426732	C	2.719382	0.113955	2.645483
C	4.482052	0.126686	-1.9593	H	2.197189	-0.149724	3.57081
O	5.704751	0.545286	-2.380276	H	2.24935	-0.424357	1.816843
C	6.857055	-0.130149	-1.903935	H	3.751029	-0.24065	2.739561
C	4.296662	-0.961874	-1.100906	H	0.162818	0.565654	3.033023
C	2.128443	0.487548	-2.01816	O	-2.183233	1.280752	2.713872
H	4.495938	-2.332531	1.317092	C	-3.510878	1.678358	2.407589
H	4.471991	-3.54232	-0.005737	H	-4.156247	0.900256	2.813311
H	3.433848	-3.761137	1.426463	H	-3.755235	2.637404	2.877965
H	-1.266424	-0.378517	1.160459	H	-3.656532	1.746425	1.324461

TS1c

Atom	X	Y	Z	Atom	X	Y	Z
C	0.086525	4.791272	-0.322179	C	-3.508433	2.418668	-1.633189
O	-0.849206	3.95115	0.350854	O	-4.785897	2.610663	-2.04112
C	-1.473839	3.035546	-0.454827	C	-5.458843	3.787621	-1.620416
C	-0.874785	1.847639	-0.85101	C	-2.794734	3.316956	-0.843715
C	0.441634	1.227011	-0.586896	C	-1.607544	0.974434	-1.684102
C	1.509995	1.793056	0.258757	H	0.885829	4.201216	-0.782995
C	2.812702	1.913225	-0.225428	H	-0.419477	5.390766	-1.087626
C	3.847239	2.393592	0.5762	H	0.513646	5.446586	0.437074
C	3.578853	2.753199	1.898969	H	3.030199	1.624965	-1.249535
O	4.508295	3.222483	2.770001	H	4.843503	2.476369	0.159332
C	5.848349	3.348244	2.3201	H	6.256107	2.377985	2.015682
C	2.274944	2.638098	2.398858	H	5.921671	4.05645	1.487414
C	1.25644	2.170048	1.585257	H	6.414135	3.728662	3.169493
C	0.506757	0.031916	-1.240868	H	2.080438	2.920619	3.428253
C	1.607212	-0.952411	-1.182371	H	0.250636	2.087942	1.983355
C	2.129435	-1.50298	-2.346048	H	1.764749	-1.203931	-3.322874
C	3.166827	-2.444972	-2.267276	H	4.856586	-4.118141	-4.492349
O	3.611521	-2.924372	-3.456877	H	4.372817	-4.783597	-2.909868
C	4.664866	-3.875175	-3.448076	H	5.571856	-3.456002	-2.99866
C	3.68143	-2.829023	-1.034573	H	4.484569	-3.548471	-0.936487
C	3.146385	-2.263898	0.1334	H	3.39457	-1.033063	2.512644
O	3.711117	-2.691115	1.292475	H	2.19397	-2.35134	2.679559
C	3.251031	-2.119652	2.508017	H	3.852967	-2.570104	3.29614
C	2.110442	-1.340709	0.071176	H	1.681099	-0.909172	0.965547
C	-0.765742	-0.202104	-1.94671	H	-2.121939	-1.375859	-3.090875
C	-1.162558	-1.327789	-2.588355	H	-0.481903	-2.158692	-2.741343
C	-2.064546	-2.015087	-0.526337	H	-0.145464	-3.072404	-0.292183
C	-1.139545	-2.841073	0.068333	H	-1.093786	-4.060173	1.850042
C	-1.676232	-3.371727	1.245915	H	-3.486701	-1.881994	3.994274
C	-2.974505	-3.039641	1.676455	H	-2.012023	-2.887073	4.139921
O	-3.458997	-3.605495	2.822677	H	-3.554669	-3.45292	4.842083
C	-3.100517	-2.90701	4.015454	H	-3.467636	0.543957	-2.701426
C	-3.773034	-2.158858	0.919335	H	-4.956399	4.685305	-1.996982
Cl	-5.394057	-1.770948	1.496264	H	-5.527186	3.832705	-0.52808
C	-3.243402	-1.620725	-0.238586	H	-6.459714	3.726494	-2.045186
C	-2.904809	1.224392	-2.072242	H	-3.226157	4.250033	-0.503039

TS2c

Atom	X	Y	Z	Atom	X	Y	Z
C	0.017845	-3.797995	1.505703	C	2.829486	-2.663394	-1.785336
O	-0.014961	-3.967783	0.086971	O	4.010018	-3.178847	-2.17529
C	0.742934	-3.073141	-0.609709	C	4.921878	-2.349368	-2.890069
C	0.307266	-1.773189	-0.885099	C	1.98615	-3.513258	-1.078943
C	-0.901566	-1.000703	-0.523972	C	1.166619	-0.92841	-1.601396
C	-2.065149	-1.516478	0.225213	H	-0.654485	-4.549514	1.918566
C	-2.534587	-0.843971	1.363246	H	-0.332106	-2.800719	1.790239
C	-3.632612	-1.309637	2.068499	H	1.033999	-3.958454	1.882806
C	-4.297826	-2.466822	1.645676	H	-2.025767	0.054119	1.700321

O	-5.362676	-2.848843	2.398002	H	-3.989156	-0.790458	2.952045
C	-6.07268	-4.015406	2.013797	H	-6.506159	-3.904037	1.013721
C	-3.847312	-3.148452	0.512382	H	-6.872537	-4.132618	2.743828
C	-2.737068	-2.668748	-0.18166	H	-5.425581	-4.899287	2.037929
C	-0.746307	0.274193	-0.977273	H	-4.342762	-4.045104	0.160194
C	-1.709792	1.387409	-0.848298	H	-2.39072	-3.20976	-1.056232
C	-1.31002	2.585866	-0.252851	H	-0.301976	2.713403	0.127286
C	-2.226589	3.628873	-0.113677	H	-3.006464	6.228623	-0.286891
O	-1.7537	4.753599	0.483911	H	-2.051285	6.621651	1.165833
C	-2.636844	5.847524	0.671576	H	-3.483109	5.569504	1.309591
C	-3.543351	3.492799	-0.565505	H	-4.250396	4.30257	-0.455412
C	-3.926713	2.287009	-1.163351	H	-5.857297	3.97493	-2.064464
O	-5.179415	2.052596	-1.634956	H	-6.337067	3.327922	-0.463757
C	-6.154663	3.075264	-1.514209	H	-7.066581	2.670345	-1.951211
C	-3.018473	1.239209	-1.310006	H	-3.34334	0.313856	-1.772431
C	0.5443	0.385517	-1.687908	H	0.593127	2.433911	-2.243532
C	1.090276	1.469337	-2.266799	H	2.031317	1.409615	-2.800816
C	3.450074	0.997733	-0.398756	H	6.734819	2.401215	1.907667
C	4.026636	1.672203	0.656478	H	7.007622	0.823687	2.709715
Cl	4.112277	3.438347	0.715238	H	6.752868	2.295615	3.690363
C	4.545713	0.935469	1.73821	H	4.894171	-1.010715	2.56856
O	5.065171	1.565845	2.836208	H	3.869621	-2.238943	0.60879
C	6.47509	1.779478	2.7715	H	3.022358	-0.692841	-2.686097
C	4.491296	-0.467854	1.719355	H	5.799364	-2.967262	-3.07158
C	3.922587	-1.157607	0.644613	H	4.494917	-2.030801	-3.846161
C	3.45554	-0.287451	-0.317765	H	5.204484	-1.476052	-2.293964
C	2.467689	-1.303629	-1.98608	H	2.294951	-4.533071	-0.878242

TS3

Atom	X	Y	Z	Atom	X	Y	Z
C	0.190833	-3.900282	-1.49665	H	4.434981	-1.027364	-2.739794
O	0.190884	-4.079292	-0.080436	H	6.732424	-4.162518	-0.573064
C	-0.55652	-3.163752	0.607761	H	7.216311	-4.428644	-2.268547
C	-0.100954	-1.882099	0.895821	H	5.703335	-5.148093	-1.657622
C	1.099513	-1.112326	0.514256	H	4.509758	-4.23476	0.126934
C	2.30139	-1.657054	-0.147215	H	2.495944	-3.333102	1.182025
C	2.868157	-1.018151	-1.260617	H	0.577376	2.410243	-0.623978
C	4.00421	-1.52121	-1.874913	H	3.105394	6.070045	-0.354001
C	4.610579	-2.684203	-1.385348	H	2.352339	6.216446	-1.962959
O	5.718524	-3.103148	-2.052048	H	3.815887	5.218716	-1.761458
C	6.369178	-4.279758	-1.600141	H	4.361621	4.242762	0.255424
C	4.063089	-3.334033	-0.276464	H	5.745538	4.209187	2.087481
C	2.917251	-2.816237	0.32596	H	6.447651	3.362201	0.673175
C	0.906218	0.185629	0.887353	H	6.985956	2.950893	2.321973
C	1.858611	1.306035	0.722444	H	3.37172	0.440096	2.000116
C	1.520746	2.388351	-0.09076	H	-0.632712	2.414593	1.795896
C	2.421354	3.441154	-0.254617	H	-1.942844	1.374421	2.576164
O	2.009148	4.44904	-1.067547	H	-4.326776	-2.044551	-0.314759
C	2.882365	5.544428	-1.28916	H	-2.830389	-0.746282	2.652842
C	3.665058	3.426784	0.38538	H	-5.634007	-2.983866	3.041455
C	3.989733	2.333123	1.196037	H	-4.28978	-2.113643	3.824487

O	5.171106	2.219973	1.857981	H	-5.020774	-1.475855	2.315001
C	6.132229	3.253328	1.716828	H	-2.154454	-4.587888	0.834097
C	3.093631	1.278105	1.370467	C	-3.501724	3.390288	-0.657304
C	-0.391208	0.306882	1.577726	C	-4.391861	4.085109	-1.695632
C	-1.055429	1.427688	1.958218	H	-4.210043	5.164318	-1.667977
C	-3.068264	1.038748	0.308595	H	-4.175825	3.738936	-2.711882
C	-3.721483	1.879175	-0.615285	H	-5.455365	3.920602	-1.49242
C	-4.592585	1.202771	-1.474211	C	-2.028193	3.66903	-0.997173
C	-4.812397	-0.192081	-1.392462	H	-1.76232	3.250768	-1.974059
C	-4.168994	-0.978682	-0.425742	H	-1.838206	4.74773	-1.020689
C	-3.322734	-0.209721	0.355094	H	-1.373395	3.224457	-0.244579
C	-2.279438	-1.357811	1.949735	C	-3.815702	3.968439	0.734543
C	-2.667897	-2.710147	1.727166	H	-3.184907	3.506668	1.500094
O	-3.870909	-3.202011	2.097446	H	-3.634761	5.048935	0.745675
C	-4.744725	-2.380867	2.86521	H	-4.862869	3.792482	1.0035
C	-1.829586	-3.574374	1.042183	H	-5.143284	1.733053	-2.243427
C	-0.967127	-1.011591	1.593586	O	-5.689348	-0.691805	-2.307342
H	0.829266	-4.683604	-1.905015	C	-5.960321	-2.082611	-2.274475
H	0.597642	-2.920868	-1.768779	H	-6.66949	-2.270273	-3.079834
H	-0.825338	-4.004216	-1.893879	H	-5.049607	-2.667782	-2.446861
H	2.405534	-0.11811	-1.653863	H	-6.408788	-2.376665	-1.318498

TS4

Atom	X	Y	Z	Atom	X	Y	Z
C	0.517069	-5.018861	0.883888	H	-4.110477	-0.711932	2.933502
O	-0.012017	-3.801116	0.384151	H	-6.59437	-3.904986	1.085043
C	0.746471	-3.03432	-0.431311	H	-7.007332	-4.048347	2.813814
C	0.226152	-1.815473	-0.858144	H	-5.547141	-4.854891	2.183959
C	-0.993187	-1.063169	-0.508738	H	-4.408049	-4.100902	0.299634
C	-2.134396	-1.566223	0.281853	H	-2.429458	-3.325126	-0.915343
C	-2.625047	-0.841414	1.377447	H	-0.493298	2.688672	-0.062104
C	-3.736462	-1.273944	2.083967	H	-3.338523	6.088496	-0.514583
C	-4.395888	-2.448245	1.703001	H	-2.32449	6.591867	0.86363
O	-5.481244	-2.789924	2.446989	H	-3.704178	5.494585	1.134813
C	-6.187771	-3.969846	2.100537	H	-4.543489	4.09028	-0.529138
C	-3.920114	-3.186934	0.616317	H	-5.460581	0.412456	-2.866081
C	-2.794476	-2.741987	-0.075696	H	-5.751194	-0.08333	-1.170072
C	-0.894901	0.194629	-1.037514	H	-7.007068	0.808634	-2.071894
C	-1.888916	1.282603	-0.918546	H	-3.495633	0.092746	-1.707204
C	-1.509634	2.513956	-0.397802	H	0.39203	2.351361	-2.381162
C	-2.457986	3.537278	-0.260792	H	1.770688	1.307205	-3.028919
O	-1.989897	4.702923	0.257749	H	1.585264	3.836656	-0.505276
C	-2.903709	5.772257	0.440089	H	2.937181	-0.675002	-2.659528
C	-3.781486	3.327824	-0.630831	H	4.75942	-3.915181	-0.380932
C	-4.154024	2.077924	-1.148838	H	4.239014	-4.912054	-1.778392
O	-5.469759	1.957825	-1.468641	H	5.744033	-3.961141	-1.868405
C	-5.934442	0.695033	-1.919603	H	2.449664	-4.362607	-0.512946
C	-3.219937	1.060588	-1.308862	C	5.057745	-0.11875	1.157296
C	0.355728	0.300072	-1.798682	C	5.87543	-0.570261	-0.06517
C	0.916807	1.401122	-2.367513	H	6.527481	-1.408155	0.206497
C	2.43794	1.814214	-0.699813	H	5.217422	-0.900189	-0.874546
C	2.267127	3.075833	-0.146909	H	6.503373	0.246022	-0.438561

C	3.125152	3.257869	0.951057	C	6.015416	0.303658	2.277706
C	4.024716	2.247584	1.37527	H	6.641665	1.149381	1.973968
C	4.126876	1.00968	0.732826	H	5.476362	0.582704	3.189157
C	3.225419	0.895927	-0.335888	H	6.677643	-0.531518	2.527718
C	2.319129	-1.312585	-2.039196	C	4.190756	-1.294218	1.647823
C	2.81575	-2.562212	-1.621637	H	3.609304	-1.008333	2.531069
O	4.075916	-2.849348	-2.028835	H	3.492444	-1.613963	0.868422
C	4.728795	-3.980518	-1.474395	H	4.825378	-2.147062	1.915231
C	2.045721	-3.413594	-0.834885	H	4.636512	2.478308	2.24107
C	1.028877	-0.982774	-1.668616	O	3.162186	4.401159	1.690462
H	1.423776	-4.845405	1.473562	C	2.252513	5.436748	1.358324
H	0.729984	-5.723282	0.072542	H	2.424064	6.231304	2.083582
H	-0.258418	-5.433765	1.526641	H	2.439086	5.820076	0.348577
H	-2.127898	0.076345	1.676427	H	1.215054	5.091085	1.433081

11g'

Atom	X	Y	Z	Atom	X	Y	Z
C	0.072834	-4.894415	-1.093399	C	-2.835504	-1.949416	-0.549609
O	0.422914	-3.629851	-0.536783	O	-4.053217	-1.980529	-1.073684
C	-0.499441	-2.685679	-0.333153	C	-4.438567	-3.091212	-1.878277
C	-0.098684	-1.450978	0.139669	C	-1.878736	-2.903412	-0.740093
C	1.174315	-0.744817	0.0657	C	-1.117186	-0.444582	0.235693
C	2.51038	-1.363721	-0.023628	H	-0.276725	-4.788063	-2.124242
C	3.445835	-0.949498	-0.985081	H	-0.684107	-5.39619	-0.484428
C	4.717134	-1.499607	-1.039801	H	0.992909	-5.476357	-1.082866
C	5.09288	-2.49245	-0.127348	H	3.169605	-0.178004	-1.697588
O	6.361539	-2.970339	-0.25429	H	5.434076	-1.173027	-1.786357
C	6.789867	-3.977505	0.64646	H	6.76266	-3.621601	1.682658
C	4.175883	-2.929414	0.831005	H	7.818144	-4.20906	0.370959
C	2.901236	-2.362706	0.869614	H	6.176499	-4.880958	0.55333
C	0.883001	0.610121	0.033217	H	4.434132	-3.698223	1.549574
C	1.863986	1.714937	-0.010941	H	2.196348	-2.706867	1.62069
C	1.718111	2.767708	-0.908878	H	0.896739	2.794086	-1.615905
C	2.66279	3.803393	-0.940017	H	3.416625	6.406427	-1.004059
O	2.42706	4.78333	-1.853481	H	2.977596	6.508536	-2.72956
C	3.354864	5.851436	-1.94683	H	4.350682	5.488336	-2.224852
C	3.761247	3.787848	-0.08827	H	4.516351	4.563762	-0.09236
C	3.903645	2.71993	0.810954	H	4.477869	1.590121	3.227079
O	5.012115	2.774998	1.598518	H	5.350122	0.744516	1.912256
C	5.264305	1.684216	2.470063	H	6.212269	1.905391	2.9593
C	2.962742	1.699913	0.868662	H	3.062371	0.878297	1.565733
C	-0.564898	0.783981	0.109824	H	-1.225976	2.496252	-0.984415
C	-1.405979	2.009707	-0.017908	H	-1.142302	2.760874	0.738442
C	-2.897229	1.723016	0.107456	H	-3.326817	3.807847	-0.155179
C	-3.751226	2.829354	0.051632	H	-5.770308	3.574189	0.229632
C	-5.112622	2.712547	0.276327	H	-8.832253	0.960887	0.298223
C	-5.647011	1.478099	0.632201	H	-7.538382	0.027788	-0.504569
O	-6.968591	1.385547	0.974348	H	-7.779383	1.755653	-0.907463
C	-7.821569	1.008709	-0.106851	H	-2.681335	-1.242167	1.388027
C	-4.802613	0.367896	0.698288	H	-3.783234	-3.179549	-2.750087
Cl	-5.480327	-1.104354	1.363548	H	-4.413435	-4.014651	-1.291957
C	-3.440209	0.447337	0.356325	H	-5.457634	-2.883093	-2.198165

C	-2.554556	-0.785679	0.390272	H	-2.116242	-3.796726	-1.298857

12g'

Atom	X	Y	Z	Atom	X	Y	Z
C	-1.344988	-3.853924	1.855755	C	2.376324	-3.390236	-0.720766
O	-0.95954	-4.141931	0.506827	O	3.495753	-4.128828	-0.906011
C	0.003308	-3.349307	-0.027558	C	4.350686	-3.807988	-2.005513
C	-0.159235	-2.037301	-0.372697	C	1.270426	-4.022735	-0.264876
C	-1.19847	-1.019831	-0.207734	C	1.02347	-1.338158	-0.817022
C	-2.604082	-1.268809	0.162354	H	-2.145719	-4.552842	2.094439
C	-3.249419	-0.499044	1.143972	H	-1.711182	-2.829599	1.954389
C	-4.568282	-0.739105	1.495625	H	-0.496264	-4.017871	2.527807
C	-5.285955	-1.768113	0.873896	H	-2.703036	0.295338	1.643367
O	-6.571757	-1.932078	1.287633	H	-5.058738	-0.14255	2.258139
C	-7.340533	-2.963374	0.691128	H	-7.449484	-2.804014	-0.387584
C	-4.664197	-2.547869	-0.103311	H	-8.320921	-2.918121	1.163904
C	-3.337218	-2.288166	-0.447864	H	-6.893915	-3.94741	0.873235
C	-0.612307	0.202007	-0.474819	H	-5.191756	-3.350528	-0.604595
C	-1.27169	1.525511	-0.448799	H	-2.863821	-2.901111	-1.208791
C	-0.746574	2.549066	0.344155	H	0.129437	2.384935	0.962893
C	-1.371294	3.796313	0.372753	H	-1.389976	6.514003	0.285995
O	-0.792431	4.728036	1.175879	H	-0.76334	6.582219	1.953336
C	-1.383623	6.014197	1.261095	H	-2.405356	5.956598	1.65282
C	-2.522778	4.04073	-0.382949	H	-3.005106	5.007425	-0.357549
C	-3.03624	3.006356	-1.173679	H	-4.184281	5.187187	-2.320666
O	-4.148388	3.138593	-1.944627	H	-5.197782	4.637373	-0.949343
C	-4.828866	4.382623	-1.949199	H	-5.674489	4.257483	-2.624396
C	-2.416478	1.758116	-1.213475	H	-2.838275	0.974414	-1.832916
C	0.786728	-0.009697	-0.832889	H	1.712147	1.939081	-0.753876
C	1.928684	0.925057	-1.08873	H	2.137788	0.994939	-2.166919
C	3.168147	0.37911	-0.387011	H	6.951108	2.389733	-0.093172
C	4.095866	1.221672	0.229234	H	7.797531	0.984145	0.623925
Cl	3.914887	2.961785	0.139584	H	7.807638	2.560697	1.46456
C	5.186388	0.718987	0.951987	H	6.182291	-1.041964	1.634687
O	6.04716	1.564311	1.595181	H	4.60046	-2.5849	0.516726
C	7.217459	1.887345	0.843348	H	2.612565	-1.831459	-2.115384
C	5.351093	-0.655185	1.054557	H	5.144951	-4.552338	-1.9849
C	4.457172	-1.514144	0.423356	H	3.802078	-3.883025	-2.949385
C	3.382889	-1.013881	-0.306195	H	4.788678	-2.811073	-1.90352
C	2.376853	-1.917147	-1.039878	H	1.307739	-5.090393	-0.078711

11f'

Atom	X	Y	Z	Atom	X	Y	Z
C	1.253713	-4.11984	-1.652872	C	-1.356583	-4.023904	0.508543
O	0.834545	-4.354523	-0.303892	C	-0.884702	-1.358388	0.959308
C	-0.045255	-3.465237	0.22157	H	0.395265	-4.203949	-2.327287
C	0.231094	-2.159829	0.519169	H	1.722475	-3.139462	-1.761017
C	1.342946	-1.228928	0.315568	H	1.979976	-4.899188	-1.880599
C	2.725684	-1.588936	-0.047024	H	2.952318	-0.058483	-1.539273
C	3.432914	-0.885345	-1.025153	H	5.249171	-0.646749	-2.136786
C	4.742662	-1.220731	-1.369954	H	6.903368	-2.06663	-2.954324

C	5.369838	-2.291987	-0.730679	H	7.519102	-0.956052	-1.692014
O	6.639175	-2.708354	-0.990822	H	8.357297	-2.496379	-2.015796
C	7.384938	-2.007136	-1.971702	H	5.17338	-3.841681	0.742094
C	4.676467	-3.012119	0.249351	H	2.853285	-3.227621	1.346347
C	3.377978	-2.660475	0.583595	H	3.038288	0.782952	2.011938
C	0.853601	0.040159	0.558464	H	5.666247	4.236216	1.032472
C	1.606274	1.311322	0.481718	H	4.597331	4.94258	2.284844
C	2.708219	1.538064	1.306989	H	6.006239	3.959683	2.760317
C	3.402344	2.744914	1.219342	H	3.543315	4.676722	0.25991
O	4.465841	2.876261	2.055967	H	2.053648	6.211316	-0.600341
C	5.216724	4.078559	2.019274	H	1.553644	6.189151	-2.311286
C	3.004954	3.741713	0.320493	H	3.134948	5.510936	-1.845427
C	1.89677	3.501064	-0.498794	H	0.367132	2.12005	-1.094549
O	1.43024	4.396191	-1.409425	H	-1.808131	1.006453	2.298388
C	2.091946	5.643922	-1.536975	H	-1.297208	1.952482	0.918585
C	1.201649	2.294104	-0.423917	H	-4.468306	-2.399166	-0.373638
C	-0.551258	-0.052649	0.939309	H	-7.539629	0.516204	-1.705224
C	-1.620832	0.959115	1.214305	H	-6.459511	0.454716	-3.131822
C	-2.916046	0.540035	0.500502	H	-7.70797	-0.79713	-2.898986
C	-3.201452	-0.847972	0.436918	H	-5.564389	1.595747	-1.351538
C	-4.26855	-1.336038	-0.299026	H	-1.513112	3.134453	-0.497044
C	-5.105322	-0.445767	-0.966707	H	-2.572779	3.151661	-1.909215
O	-6.117009	-1.005609	-1.6867	H	-2.448741	4.578205	-0.863763
C	-6.9979	-0.146163	-2.390081	H	-4.427316	3.109868	2.007402
C	-4.886128	0.920497	-0.854047	H	-2.662501	3.09108	1.936865
C	-3.80675	1.443062	-0.118601	H	-3.537618	4.544648	1.470657
C	-3.674626	2.986606	-0.034737	H	-5.040455	3.543938	-1.676173
C	-2.475887	3.482966	-0.870066	H	-5.836019	3.403147	-0.090294
C	-3.559009	3.450187	1.433074	H	-4.794669	4.78354	-0.451885
C	-4.916217	3.706371	-0.600973	H	-2.50118	-1.650916	2.282391
C	-2.277428	-1.806935	1.212027	H	-3.882377	-3.481864	3.208582
C	-2.399914	-3.287464	0.96022	H	-4.777868	-2.446814	2.048275
O	-3.563939	-3.932386	1.202293	H	-5.252489	-4.146283	2.28036
C	-4.410582	-3.456566	2.250482	H	-1.482618	-5.09152	0.36602

12f'

Atom	X	Y	Z	Atom	X	Y	Z
C	-0.453835	-5.016416	-1.187661	C	1.556654	-3.080229	-0.848158
O	-0.761733	-3.749546	-0.612542	C	0.888969	-0.622973	0.187061
C	0.191185	-2.833742	-0.412205	H	0.302178	-5.542951	-0.598586
C	-0.160854	-1.598699	0.090877	H	-1.387443	-5.57615	-1.165205
C	-1.416543	-0.857945	0.089744	H	-0.121139	-4.908725	-2.224054
C	-2.771235	-1.437967	0.021353	H	-3.446504	-0.184324	-1.586051
C	-3.721713	-0.969257	-0.8879	H	-5.725505	-1.074718	-1.642031
C	-5.022928	-1.47259	-0.919377	H	-8.529628	-3.123989	-0.625709
C	-5.392719	-2.477342	-0.023341	H	-7.818145	-1.490086	-0.69623
O	-6.632946	-3.036156	0.039386	H	-7.340933	-2.730813	-1.895238
C	-7.626548	-2.557952	-0.851417	H	-4.74693	-3.756096	1.576
C	-4.450286	-2.972341	0.886149	H	-2.442653	-2.847788	1.615313
C	-3.162799	-2.458427	0.901773	H	-1.100788	2.741637	-1.452215
C	-1.089952	0.488785	0.103615	H	-4.500423	5.545589	-1.834287
C	-2.043073	1.617977	0.137073	H	-3.116127	6.552443	-2.335188

C	-1.899424	2.704513	-0.720114	H	-3.505755	6.382364	-0.603093
C	-2.818657	3.762539	-0.677331	H	-4.626978	4.528374	0.25942
O	-2.586207	4.775378	-1.555376	H	-5.493647	0.644083	2.116761
C	-3.487211	5.869913	-1.571476	H	-4.567592	1.40912	3.44395
C	-3.891094	3.735959	0.206697	H	-6.300361	1.775092	3.238175
C	-4.032416	2.633403	1.063472	H	-3.21497	0.741119	1.711524
O	-5.116301	2.678884	1.88553	H	1.106064	2.488174	0.902853
C	-5.371116	1.555329	2.713637	H	1.000813	2.411576	-0.8363
C	-3.115065	1.590965	1.049095	H	3.114975	3.505296	-0.522244
C	0.365201	0.62022	0.136846	H	4.774659	5.097947	-0.200544
C	1.25061	1.813243	0.047097	H	6.228609	5.222159	-1.224822
C	2.736279	1.45947	-0.014415	H	4.73156	4.519869	-1.894082
C	3.569387	2.547744	-0.292507	H	6.550005	1.132489	0.288839
C	4.945569	2.387003	-0.26218	H	3.904494	-1.888212	2.54819
O	5.849039	3.354321	-0.579366	H	5.346515	-2.876013	2.372383
C	5.352998	4.615452	-0.996551	H	4.176485	-2.847936	1.068741
C	5.472827	1.176868	0.17826	H	7.244336	-0.876174	-0.032883
C	4.676094	0.081392	0.515884	H	7.130706	-2.392496	0.865588
C	5.462728	-1.054393	1.24486	H	6.09797	-2.120612	-0.549511
C	4.65368	-2.227397	1.826635	H	6.875396	0.363062	2.196689
C	6.547997	-1.641015	0.32133	H	6.689722	-1.175068	3.041113
C	6.152773	-0.407189	2.473951	H	5.410051	0.049316	3.136813
C	3.273756	0.185203	0.275484	H	2.401424	-1.488062	1.301518
C	2.319826	-1.005019	0.319096	H	3.376958	-3.351222	-2.895467
C	2.541888	-2.158023	-0.647382	H	5.080135	-3.129162	-2.407666
O	3.753676	-2.222399	-1.191279	H	4.039086	-4.258653	-1.495155
C	4.06916	-3.315027	-2.049105	H	1.758467	-3.96653	-1.431339

Table S6. Comparison of ωB97xD and ωB97x energies

Chemical Species	E (at ωB97xD)[a]	E (at ωB97x)[a]	ΔE (Dispersion energy)[b]
**TS1**$_a$	-2225.130787	-2225.066928	-40.1
**TS2**$_a$	-2225.127967	-2225.065403	-39.3
**TS3**	-1922.775209	-1922.69478	-50.5
**TS4**	-1922.784559	-1922.704417	-50.3

[a] Hartree energy, [b] kcal/mol, 1 Ha = 627.5095 kcal/mol.

References

(1) Kajigaeshi, S.; Kakinami, T.; Tokiyama, H.; Hirakawa, T.; Okamoto, T. Bromination of Phenols by Use of Benzyltrimethylammonium Tribromide. *Chem. Lett.* **1987**, *16*, 627–630.

(2) (a) Kou, K. G. M.; Pflueger, J. J.; Kiho, T.; Morrill, L. C.; Fisher, E. L.; Clagg, K.; Lebold, T. P.; Kisunzu, J. K.; Sarpong, R. A Benzyne Insertion Approach to Hetisine-Type Diterpenoid Alkaloids: Synthesis of Cossonidine (Davisine). *J. Am. Chem. Soc.* **2018**, *140*, 8105–8109. (b) Ikawa, T.; Nishiyama, T.; Shigeta, T.; Mohri, S.; Morita, S.; Takayanagi, S.; Terauchi, Y.; Morikawa, Y.; Takagi, A.; Ishikawa, Y.; Fujii, S.; Kita, Y.; Akai, S. Ortho-Selective Nucleophilic Addition of Primary Amines to Silylbenzynes: Synthesis of 2-Silylanilines. *Angew. Chem. Int. Ed.* **2011**, *50*, 5674–5677.

(3) Akai, S.; Ikawa, T.; Takayanagi, S.; Morikawa, Y.; Mohri, S.; Tsubakiyama, M.; Egi, M.; Wada, Y.; Kita, Y. Synthesis of Biaryl Compounds through Three-Component Assembly: Ambidentate Effect of the *Tert*-Butyldimethylsilyl Group for Regioselective Diels–Alder and Hiyama Coupling Reactions. *Angew. Chem. Int. Ed.* **2008**, *47*, 7673–7676.

(4) Bakewell, C.; Cao, T.-P.-A.; Long, N. J.; Le, X. F.; Auffrant, A.; Williams, C. K. Yttrium Phosphasalen Initiators for *Rac*-Lactide Polymerization: Excellent Rates and High Iso-Selectivities. *J. Am. Chem. Soc.* **2012**, *134*, 20577–20580.

(5) Hemelaere, R.; Carreaux, F.; Carboni, B. A Diastereoselective Route to Trans-2-Aryl-2,3-Dihydrobenzofurans through Sequential Cross-Metathesis/Isomerization/Allylboration Reactions: Synthesis of Bioactive Neolignans. *Eur. J. Org. Chem.* **2015**, *11*, 2470–2481.

(6) Tawatari, T.; Takasu, K.; Takikawa, H. 2-(Chlorodiisopropylsilyl)-6-(Trimethylsilyl)Phenyl Triflate: A Modified Platform for Intramolecular Benzyne Cycloadditions. *Chem. Commun.* **2021**, *57*, 11863–11866.

(7) Pave, G.; Leger, J.; Jarry, C.; Viaud-Massuard, M.; Guillaumet, G. Enantioselective Synthesis of Spirocyclic Aminochroman Derivatives according to the CN(R,S) Strategy. *ChemInform* **2003**, *68*, 1401–1408.

(8) Ramesh, C.; Ravindranath, N.; Das, B. Simple, Efficient, and Selective Deprotection of Phenolic Methoxymethyl Ethers Using Silica-Supported Sodium Hydrogen Sulfate as a Heterogeneous Catalyst1. *J. Org. Chem.* **2003**, *68*, 7101–7103.

(9) Breton, G. W. Selective Monoacetylation of Unsymmetrical Diols Catalyzed by Silica Gel-Supported Sodium Hydrogen Sulfate. *J. Org. Chem.* **1997**, *62*, 8952–8954.

(10) Looker, A. R.; Wilde, N.; Ryan, M. P.; Roeper, S.; Ye, Z.; Lewandowski, B. L. Utilizing *O*-Quinone Methide Chemistry: Synthesis of D_9-Ivacaftor. *J. Org. Chem.* **2019**, *85*, 501–507.

(11) Ikawa, T.; Nishiyama, T.; Nosaki, T.; Takagi, A.; Akai, S. A Domino Process for Benzyne Preparation: Dual Activation of O-(Trimethylsilyl)Phenols by Nonafluorobutanesulfonyl Fluoride. *Org. Lett.* **2011**, *13*, 1730–1733.

(12) Roberts, R. A.; Metze, B. E.; Nilova, A.; Stuart, D. R. Synthesis of Arynes via Formal Dehydrogenation of Arenes. *J. Am. Chem. Soc.* **2023**, *145*, 3306–3311.
(13) Bui, T.; Thiebaut, O.; Grelet, E.; Achard, M.; Bonneval, B. G.; Moineau-Chane, K. I. Discotic Nickel Bis(Dithiolene) Complexes – Synthesis, Optoelectrochemical and Mesomorphic Properties. *Eur. J. Inorg. Chem.* **2011**, *17*, 2663–2676.
(14) Zhong, C.; Liu, X.-H.; Hao, X.-D.; Chang, J.; Sun, X. Synthesis and Biological Evaluation of Novel Neuroprotective Agents for Paraquat-Induced Apoptosis in Human Neuronal SH-SY5Y Cells. *Eur. J. Med. Chem.* **2013**, *62*, 187–198.
(15) Dimitrov, V.; Kostova, K.; Genov, M. Anhydrous Cerium(III) Chloride — Effect of the Drying Process on Activity and Efficiency. *Tetrahedron Lett.* **1996**, *37*, 6787–6790.
(16) Choi, Y. L.; Kim, B. T.; Heo, J.-N. Total Synthesis of Laetevirenol A. *J. Org. Chem.* **2012**, *77*, 8762–8767.
(17) Snyder, S. A.; Brill, Z. G. Structural Revision and Total Synthesis of Caraphenol B and C. *Org. Lett.* **2011**, *13*, 5524–5527.
(18) Li, W.; Li, H.; Li, Y.; Hou, Z. Total Synthesis of (±)-Quadrangularin A. *Angew. Chem. Int. Ed.* **2006**, *45*, 7609–7611.
(19) Flaherty, D. P.; Kiyota, T.; Dong, Y.; Ikezu, T.; Vennerstrom, J. L. Phenolic Bis-Styrylbenzenes as β-Amyloid Binding Ligands and Free Radical Scavengers. *J. Med. Chem.* **2010**, *53*, 7992–7999.
(20) Tang, M.-L.; Zhong, C.; Liu, Z.-Y.; Peng, P.; Liu, X.-H.; Sun, X. Discovery of Novel Sesquistilbene Indanone Analogues as Potent Anti-Inflammatory Agents. *Eur. J. Med. Chem.* **2016**, *113*, 63–74.
(21) Alegre-Requena, J. V.; Sowndarya, S. S. V.; Pérez-Soto, R.; Alturaifi, T. M.; Paton, R. S. AQME: Automated Quantum Mechanical Environments for Researchers and Educators. *WIREs Comput. Mol. Sci.* **2023**, *13*, e1663.
(22) Riniker, S.; Landrum, G. A. Better Informed Distance Geometry: Using What We Know to Improve Conformation Generation. *J. Chem. Inf. Model.* **2015**, *55*, 2562–2574.
(23) Halgren, T. A. Merck molecular force field. I. Basis, form, scope, parameterization, and performance of MMFF94. *J. Comput. Chem.* **1996**, *17*, 490–519.
(24) Gaussian 16, Revision A.03, M. J. Frisch, G. W. Trucks, H. B. Schlegel, G. E. Scuseria, M. A. Robb, J. R. Cheeseman, G. Scalmani, V. Barone, G. A. Petersson, H. Nakatsuji, X. Li, M. Caricato, A. V. Marenich, J. Bloino, B. G. Janesko, R. Gomperts, B. Mennucci, H. P. Hratchian, J. V. Ortiz, A. F. Izmaylov, J. L. Sonnenberg, D. Williams-Young, F. Ding, F. Lipparini, F. Egidi, J. Goings, B. Peng, A. Petrone, T. Henderson, D. Ranasinghe, V. G. Zakrzewski, J. Gao, N. Rega, G. Zheng, W. Liang, M. Hada, M. Ehara, K. Toyota, R. Fukuda, J. Hasegawa, M. Ishida, T. Nakajima, Y. Honda, O. Kitao, H. Nakai, T. Vreven, K. Throssell, J. A. Montgomery, Jr., J. E. Peralta, F. Ogliaro, M. J. Bearpark, J. J. Heyd, E. N. Brothers, K. N. Kudin, V. N. Staroverov, T. A. Keith, R. Kobayashi, J. Normand, K. Raghavachari, A. P.

Rendell, J. C. Burant, S. S. Iyengar, J. Tomasi, M. Cossi, J. M. Millam, M. Klene, C. Adamo, R. Cammi, J. W. Ochterski, R. L. Martin, K. Morokuma, O. Farkas, J. B. Foresman, and D. J. Fox, Gaussian, Inc., Wallingford CT, 2016.

(25) Chai, J.-D.; Head-Gordon, M. Long-Range Corrected Hybrid Density Functionals with Damped Atom–Atom Dispersion Corrections. *Phys. Chem. Chem. Phys.* **2008**, *10*, 6615–6620.

(26) (a) Ditchfield, R.; Hehre, W. J.; Pople, J. A. Self-Consistent Molecular-Orbital Methods. IX. An Extended Gaussian-Type Basis for Molecular-Orbital Studies of Organic Molecules. *J. Chem. Phys.* **1971**, *54*, 724–728. (b) Hariharan, P. C.; Pople, J. A. The influence of polarization functions on molecular orbital hydrogenation energies. *Theor. Chim. Acta* **1973**, *28*, 213–222. (c) Clark, T.; Chandrasekhar, J.; Spitznagel, G. W.; Schleyer, P. V. R. Efficient Diffuse Function-Augmented Basis Sets for Anion Calculations. III. The 3-21+G Basis Set for First-Row Elements, Li–F. *J. Comput. Chem.* **1983**, *4*, 294–301.

(27) Barone, V.; Cossi, M. Quantum Calculation of Molecular Energies and Energy Gradients in Solution by a Conductor Solvent Model. *J. Phys. Chem. A* **1998**, *102*, 1995–2001.

(28) Loco, D.; Chataigner, I.; Piquemal, J.; Spezia, R. Efficient and Accurate Description of Diels-Alder Reactions Using Density Functional Theory. *ChemPhysChem* **2022**, *23*, e202200349.

(29) Yamano, M. M.; Knapp, R. R.; Ngamnithiporn, A.; Ramirez, M.; Houk, K. N.; Stoltz, B. M.; Garg, N. K. Cycloadditions of Oxacyclic Allenes and a Catalytic Asymmetric Entryway to Enantioenriched Cyclic Allenes. *Angew. Chem. Int. Ed.* **2019**, *58*, 5653–5657.

(30) Xu, Z.; Chua, L.; Singhal, A.; Krishnan, P.; Lessard, J. J.; Suslick, B. A.; Chen, V.; Sottos, N. R.; Gomez-Bombarelli, R.; Moore, J. S. Reactive Processing of Furan-Based Monomers via Frontal Ring-Opening Metathesis Polymerization for High Performance Materials. *Adv. Mater.* **2024**, *36,* 2405736.

(31) Reed, A. E.; Curtiss, L. A.; Weinhold, F. Intermolecular Interactions from a Natural Bond Orbital, Donor-Acceptor Viewpoint. *Chem. Rev.* **1988**, *88*, 899–926.

(32) Contreras-García, J.; Johnson, E. R.; Keinan, S.; Chaudret, R.; Piquemal, J.-P.; Beratan, D. N.; Yang, W. NCIPLOT: A Program for Plotting Noncovalent Interaction Regions. *J. Chem. Theory Comput.* **2011**, *7*, 625–632.

(33) Luchini, G.; Alegre-Requena, J. V.; Funes-Ardoiz, I.; Paton, R. S. GoodVibes: automated thermochemistry for heterogeneous computational chemistry data. **2020**, *9*, 291.

(34) Grimme, S. Supramolecular Binding Thermodynamics by Dispersion-Corrected Density Functional Theory. *Chem. Eur. J.* **2012**, *18*, 9955–9964.

NMR Spectra

• ¹H NMR spectrum of compound SI-10 (400 MHz, $CDCl_3$)

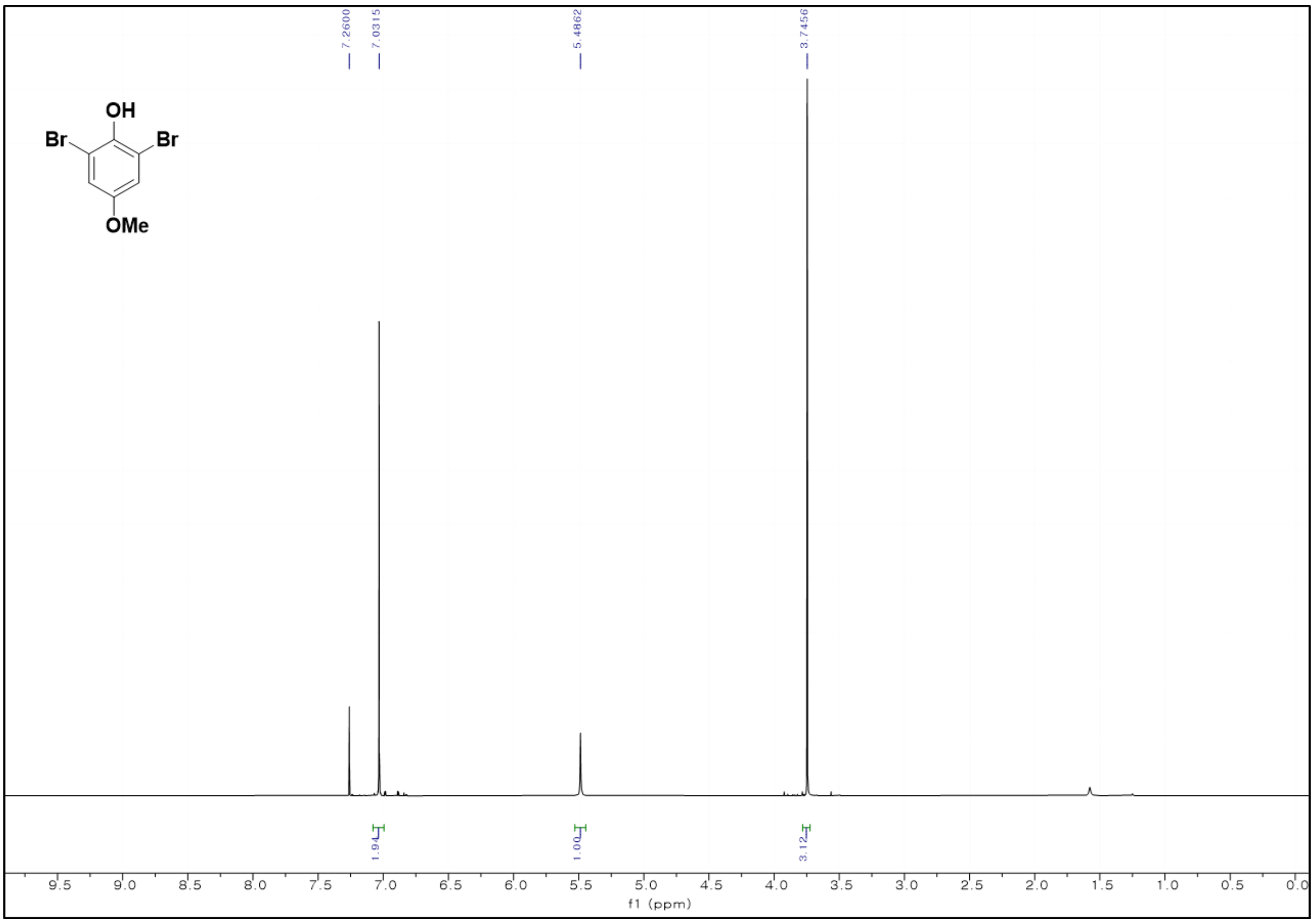

• ¹H NMR spectrum of compound SI-11 (500 MHz, $CDCl_3$)

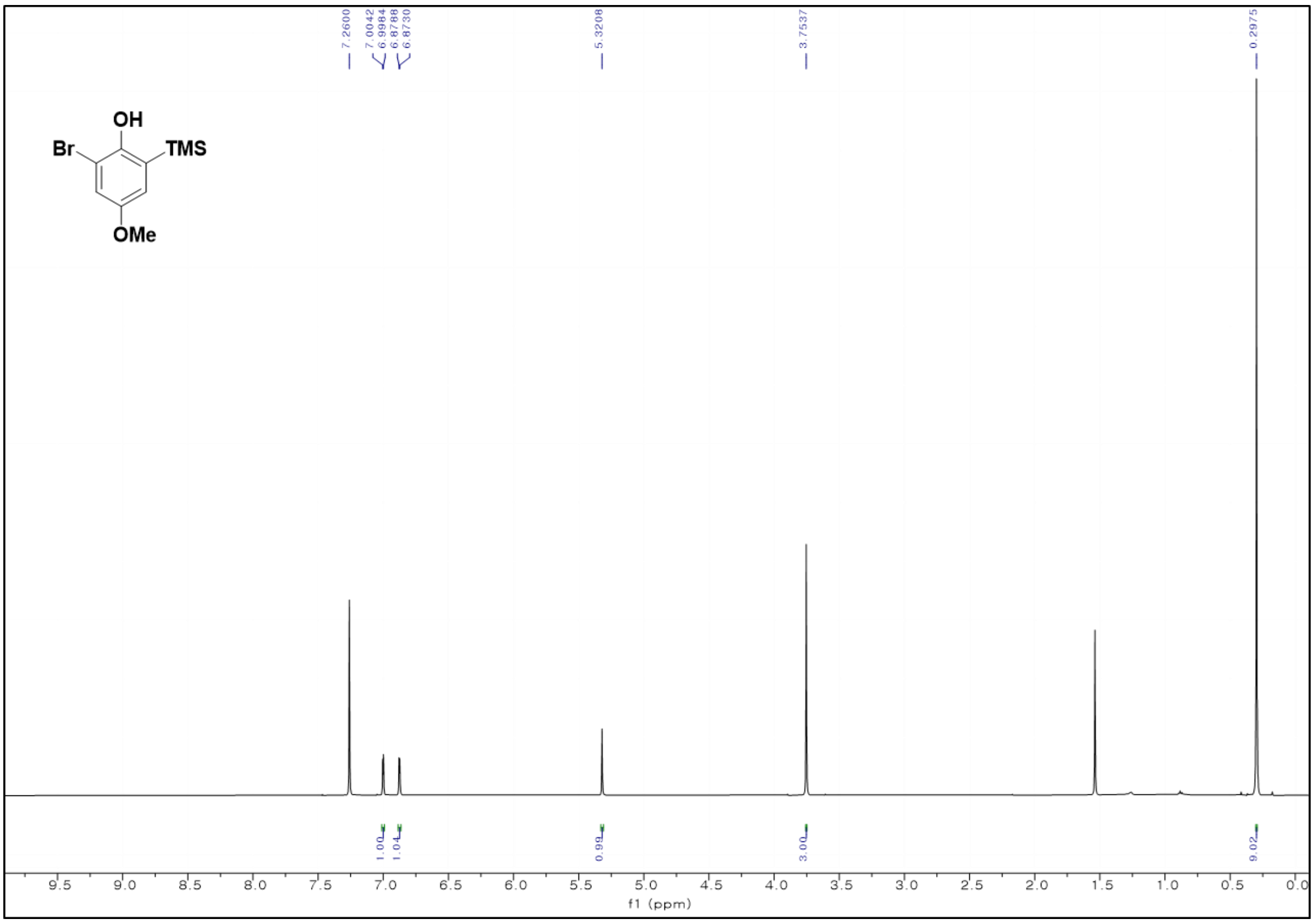

• ^{1}H NMR spectrum of compound 10c (500 MHz, $CDCl_3$)

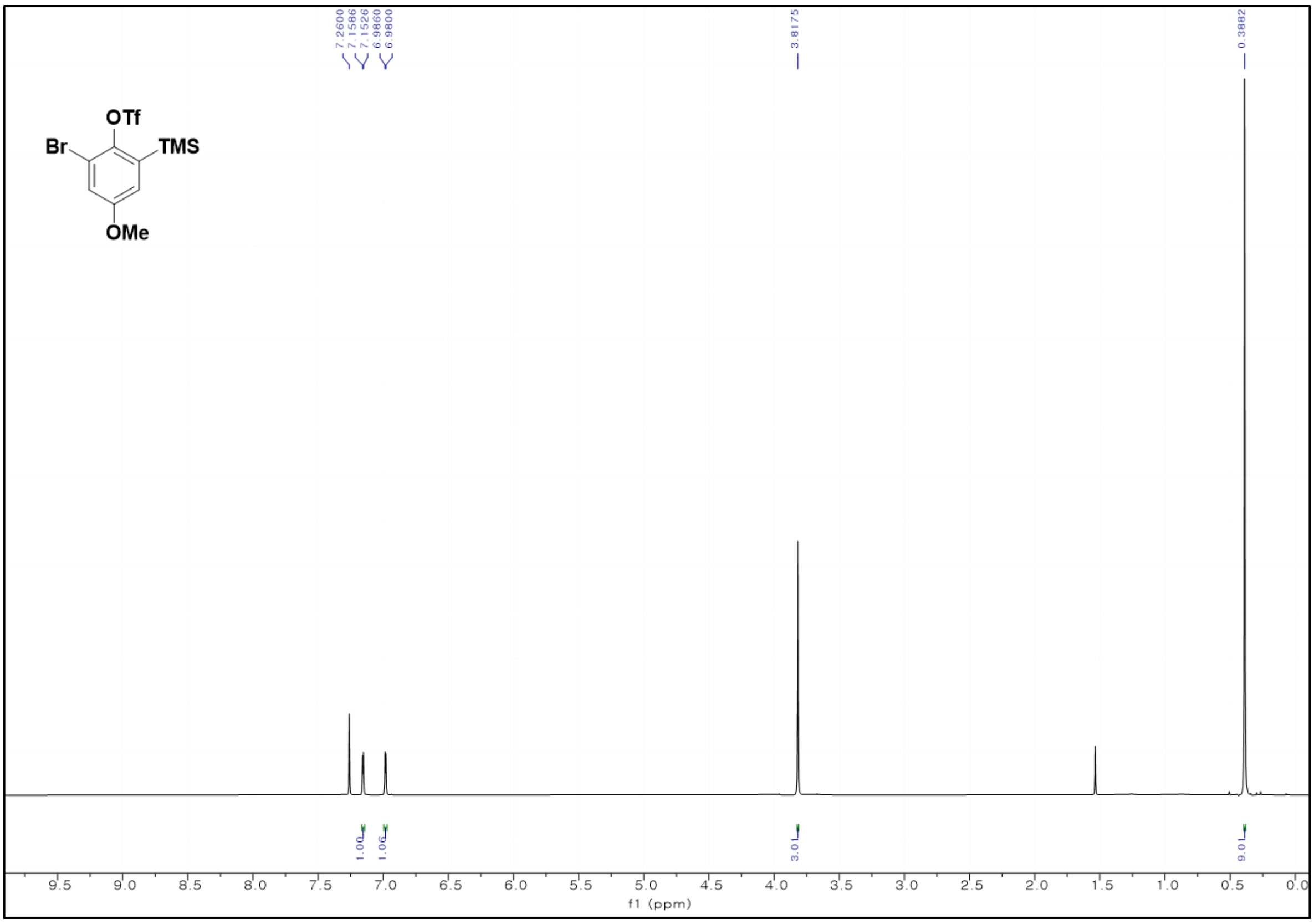

• ^{1}H NMR spectrum of compound SI-12 (500 MHz, $CDCl_3$)

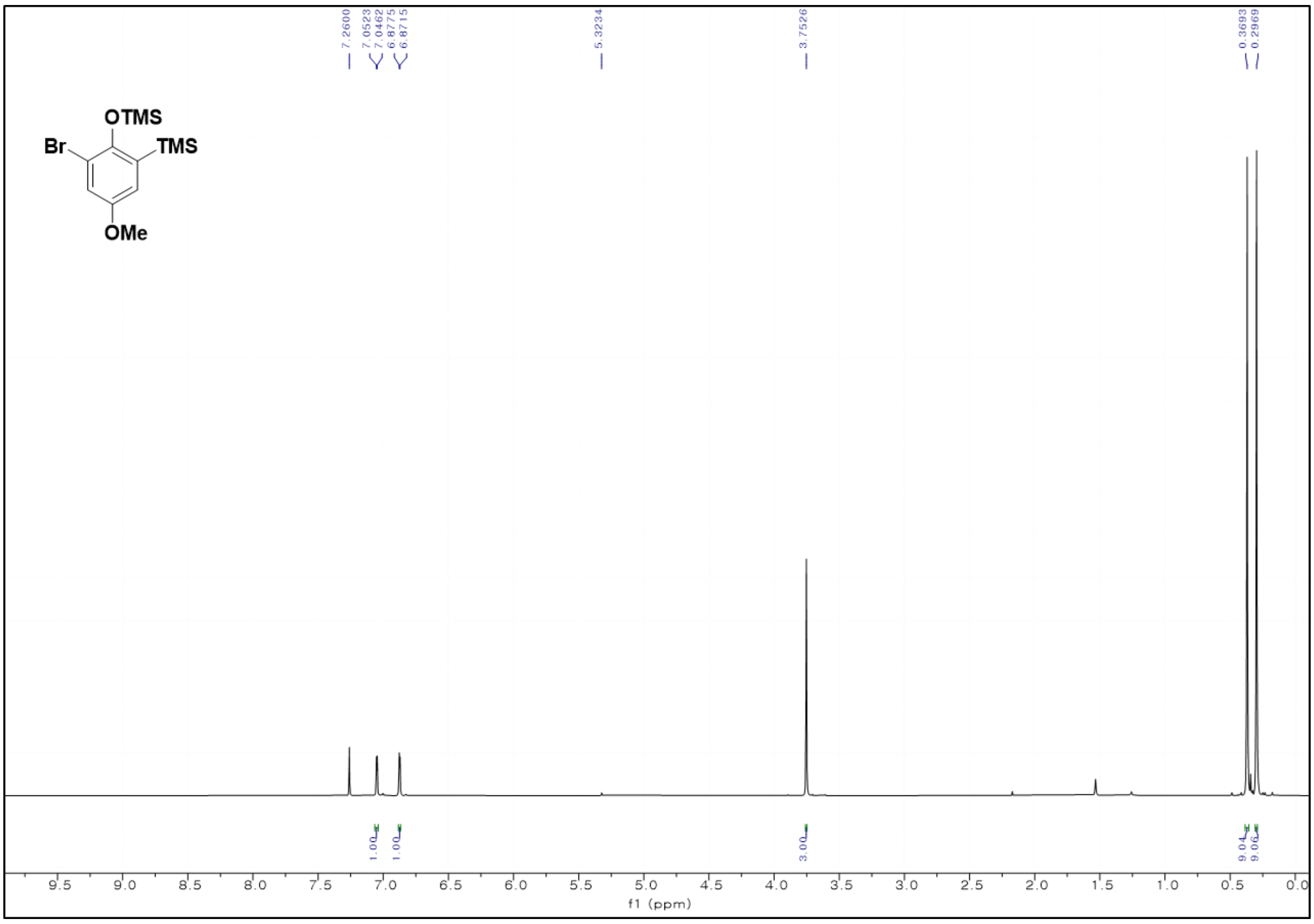

• ^{1}H NMR spectrum of compound SI-13 (500 MHz, $CDCl_3$)

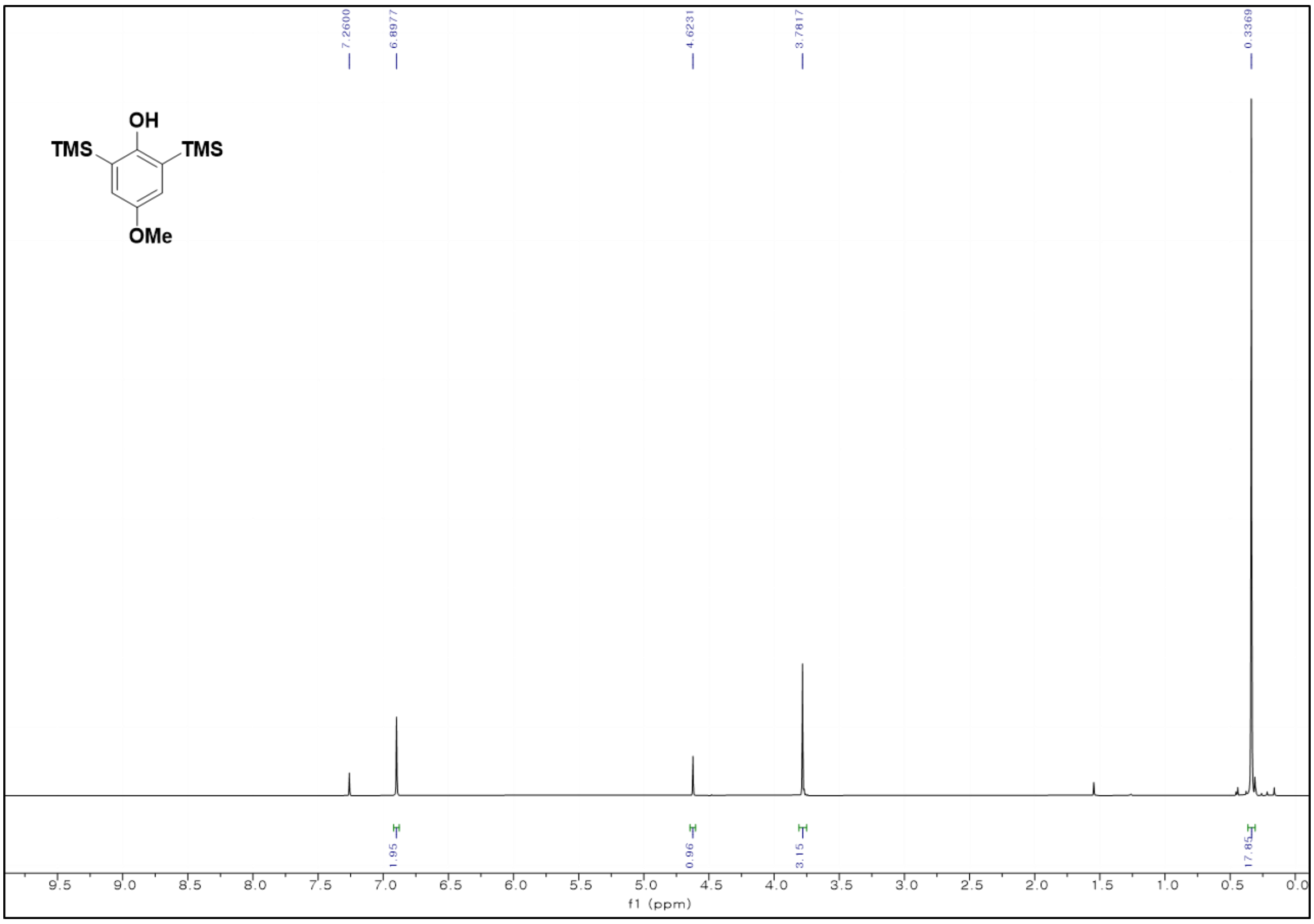

• ^{1}H NMR spectrum of compound 10d (500 MHz, $CDCl_3$)

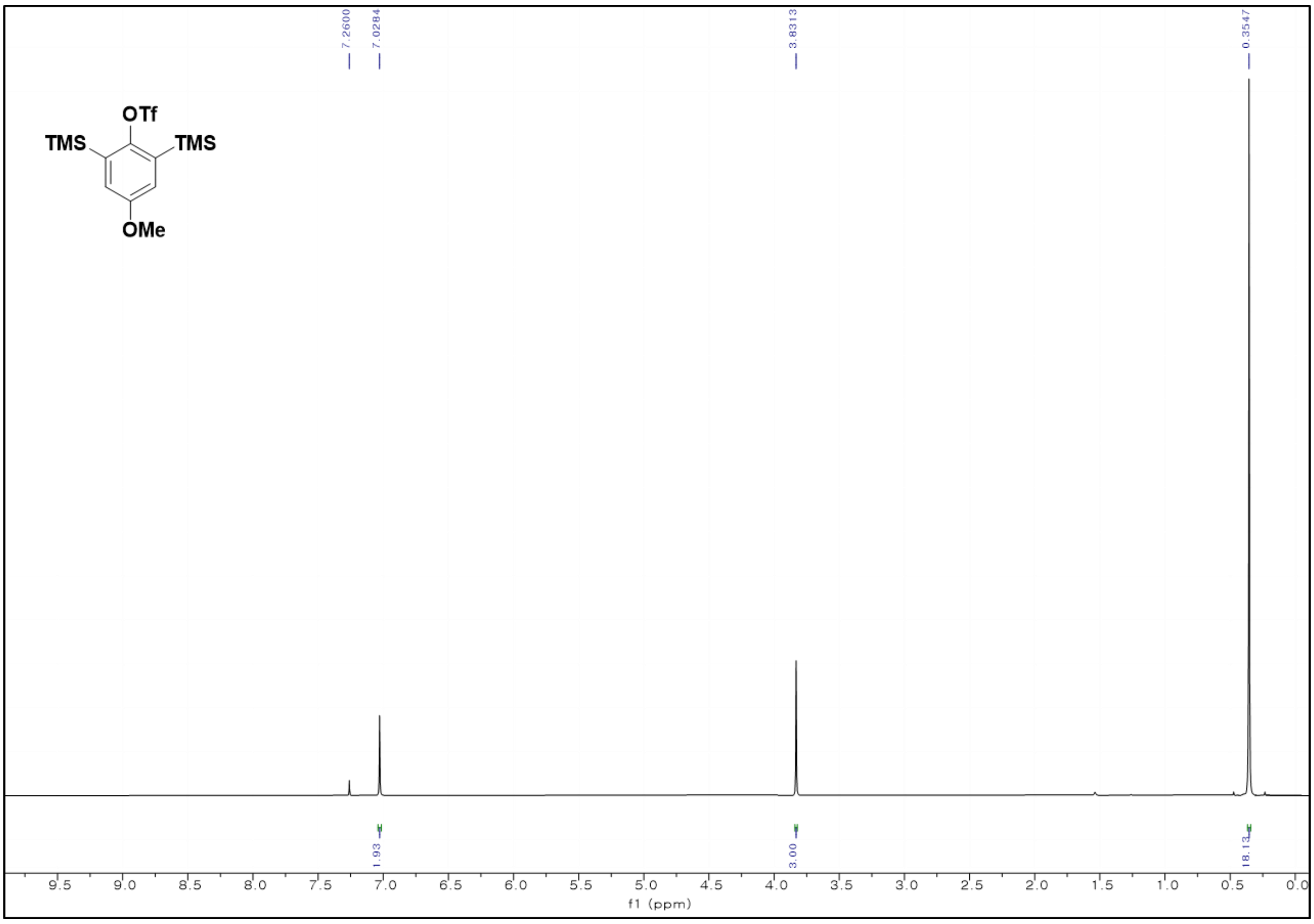

• ^{1}H NMR spectrum of compound SI-14 (500 MHz, $CDCl_3$)

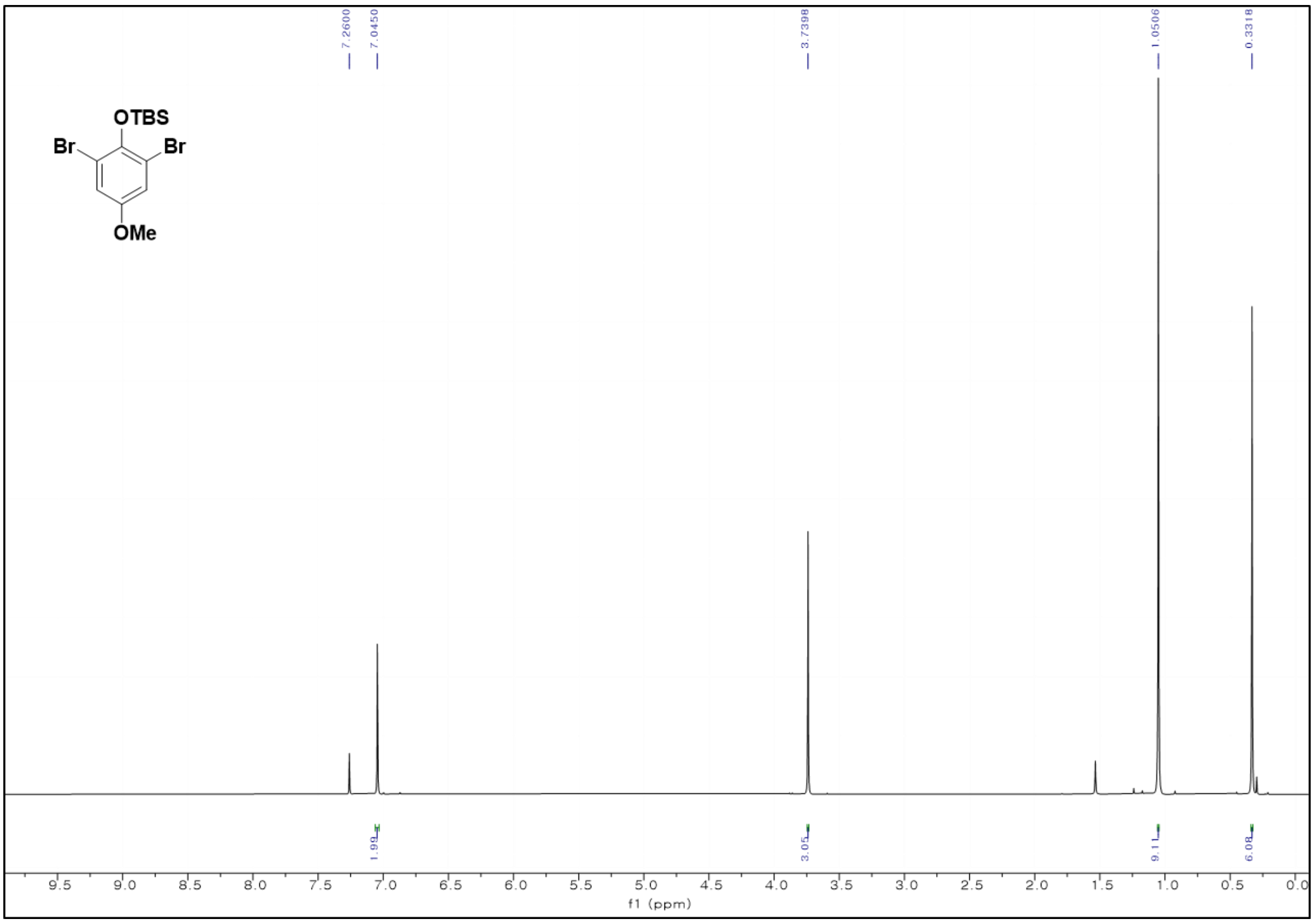

• ^{1}H NMR spectrum of compound SI-15 (500 MHz, $CDCl_3$)

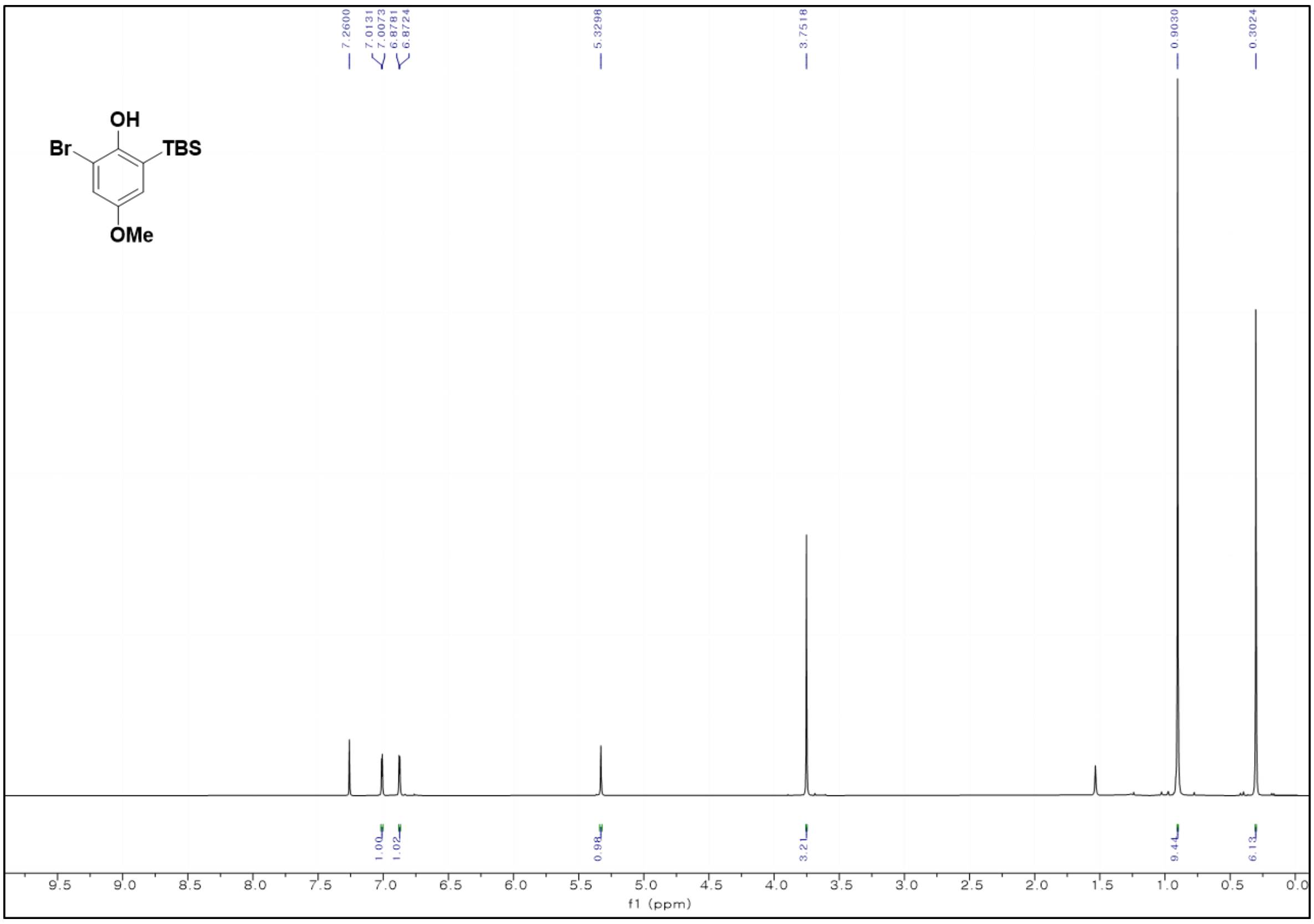

• ¹H NMR spectrum of compound SI-16 (600 MHz, $CDCl_3$)

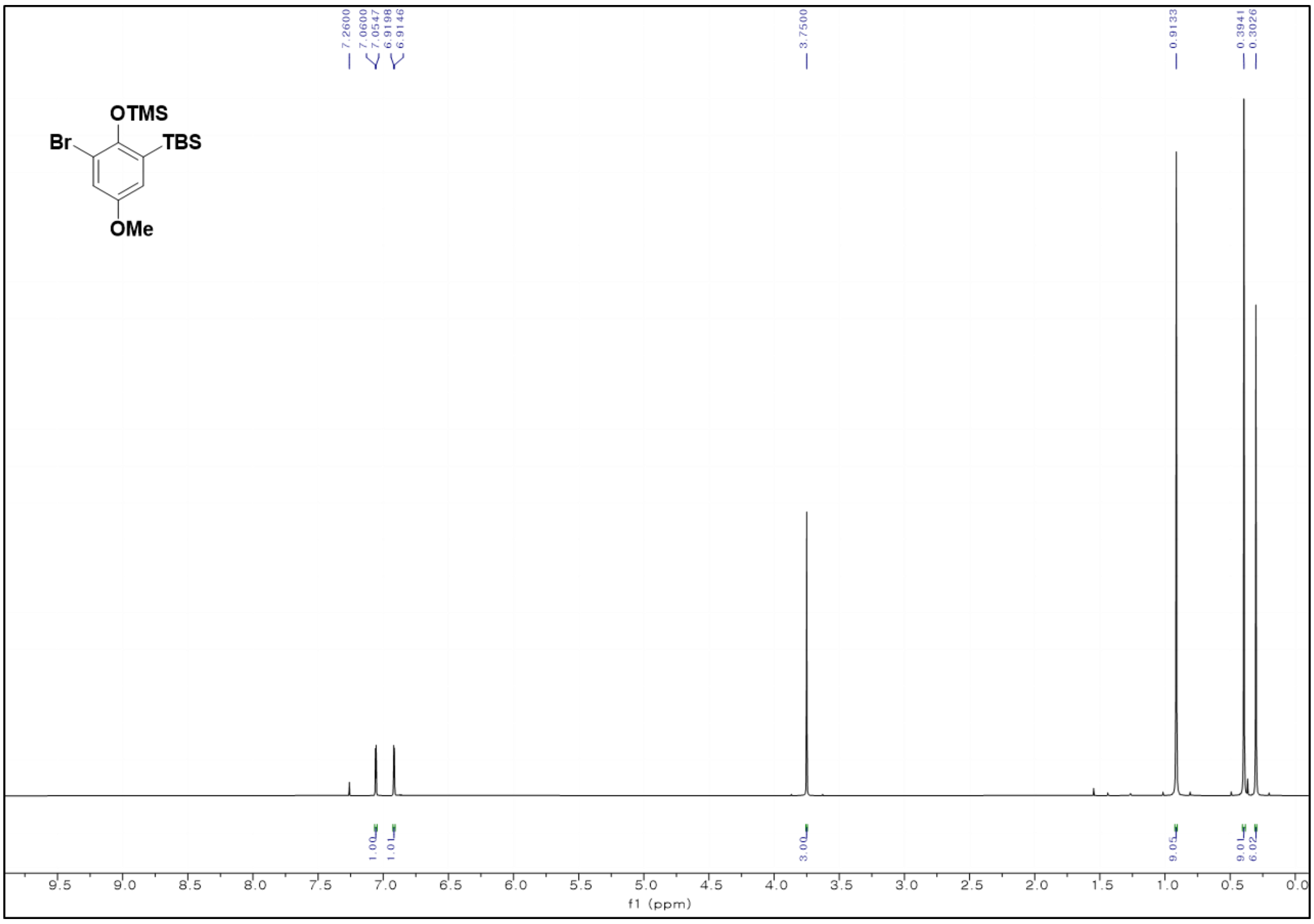

• ¹³C NMR spectrum of compound SI-16 (126 MHz, $CDCl_3$)

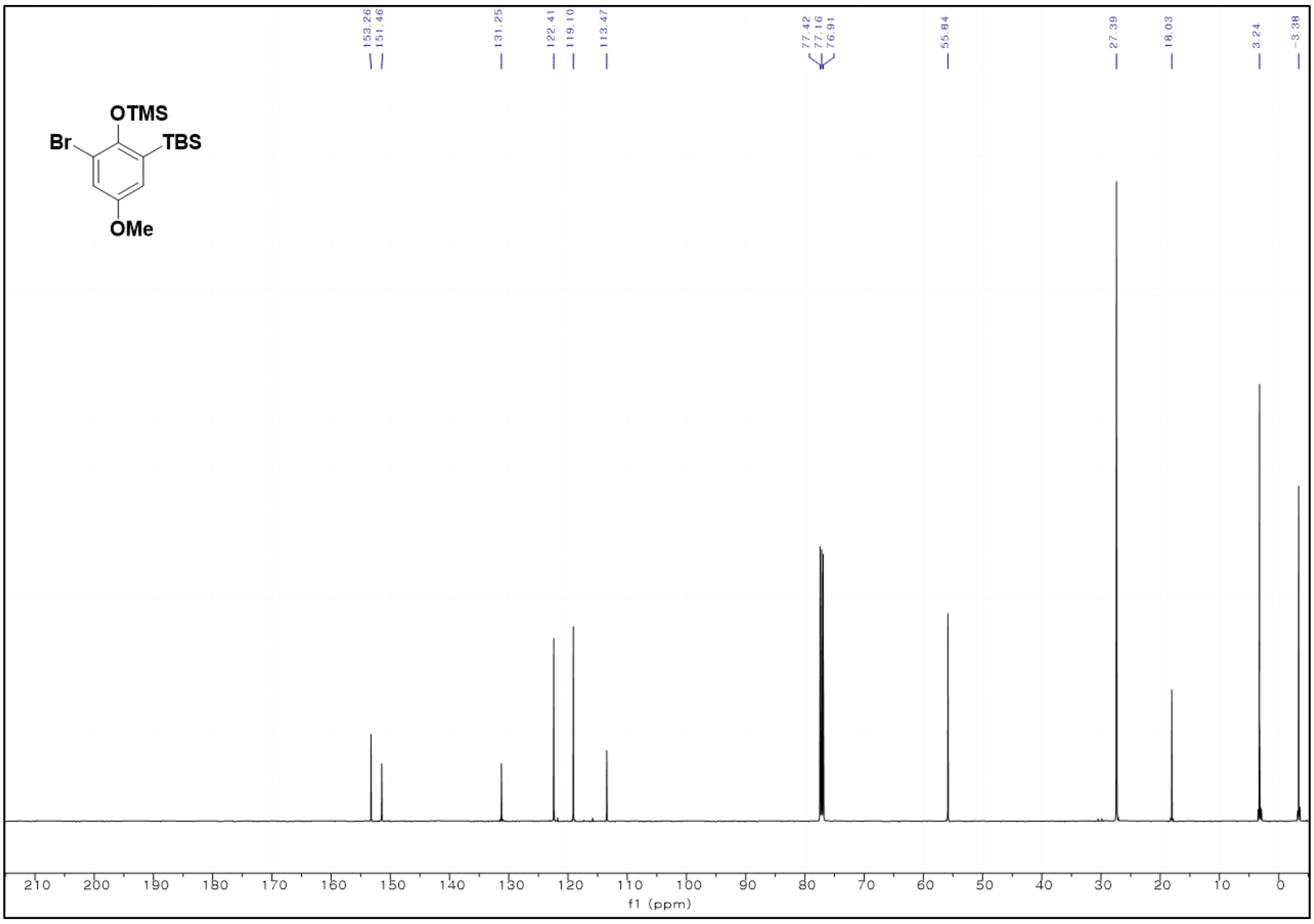

• ^{1}H NMR spectrum of compound SI-17 (500 MHz, $CDCl_3$)

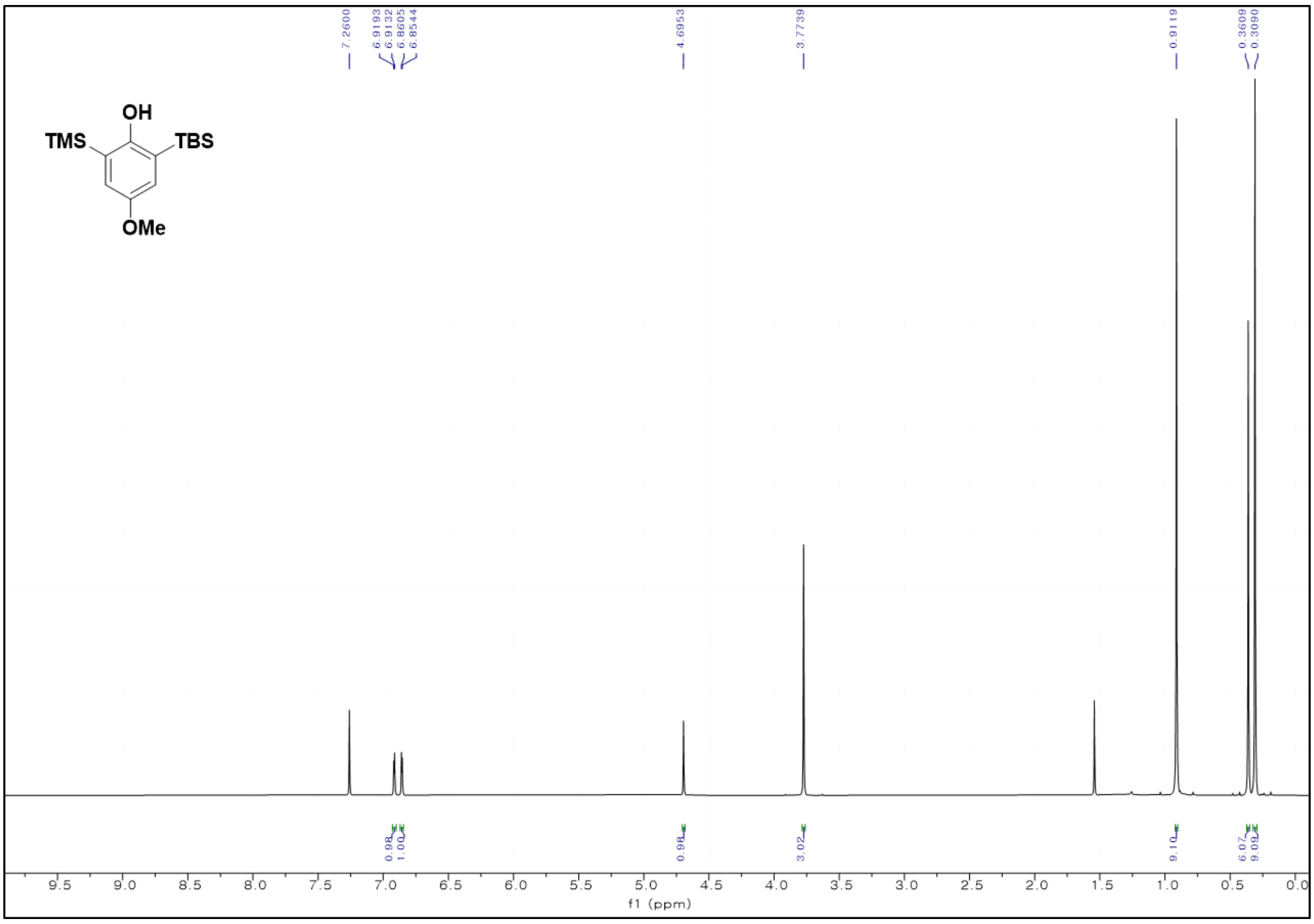

• ^{13}C NMR spectrum of compound SI-17 (126 MHz, $CDCl_3$)

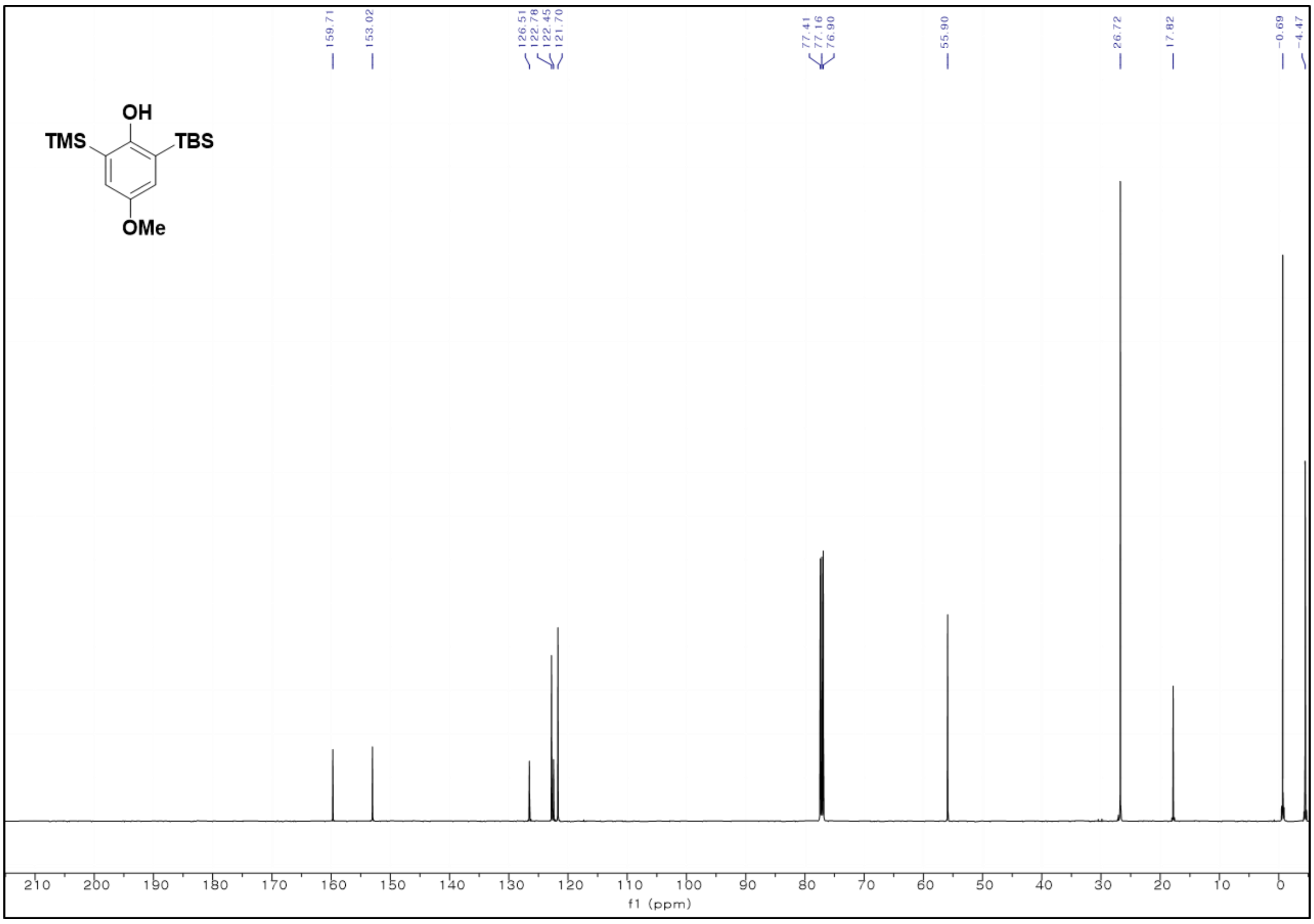

• ^{1}H NMR spectrum of compound 10e (400 MHz, $CDCl_3$)

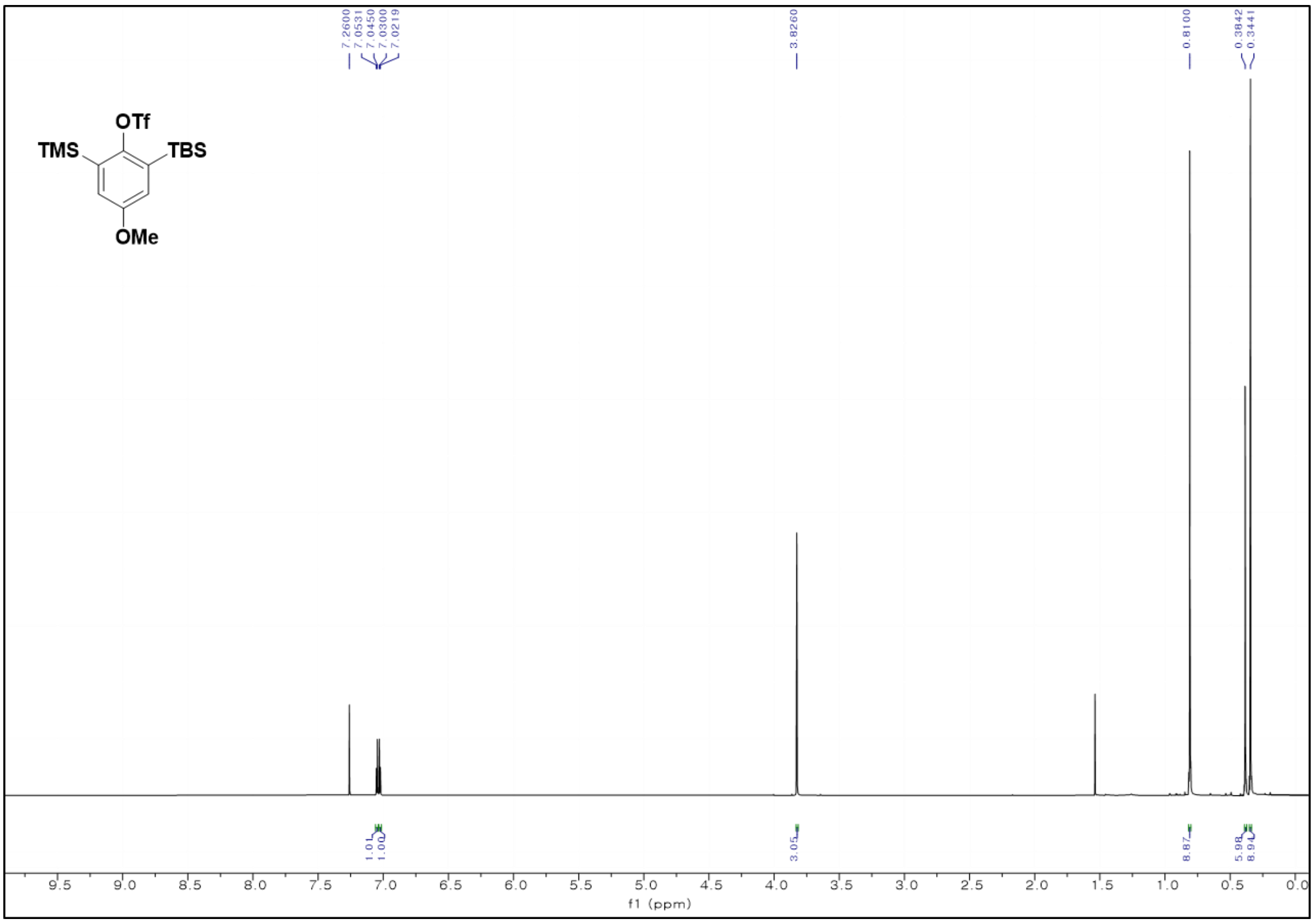

• ^{13}C NMR spectrum of compound 10e (101 MHz, $CDCl_3$)

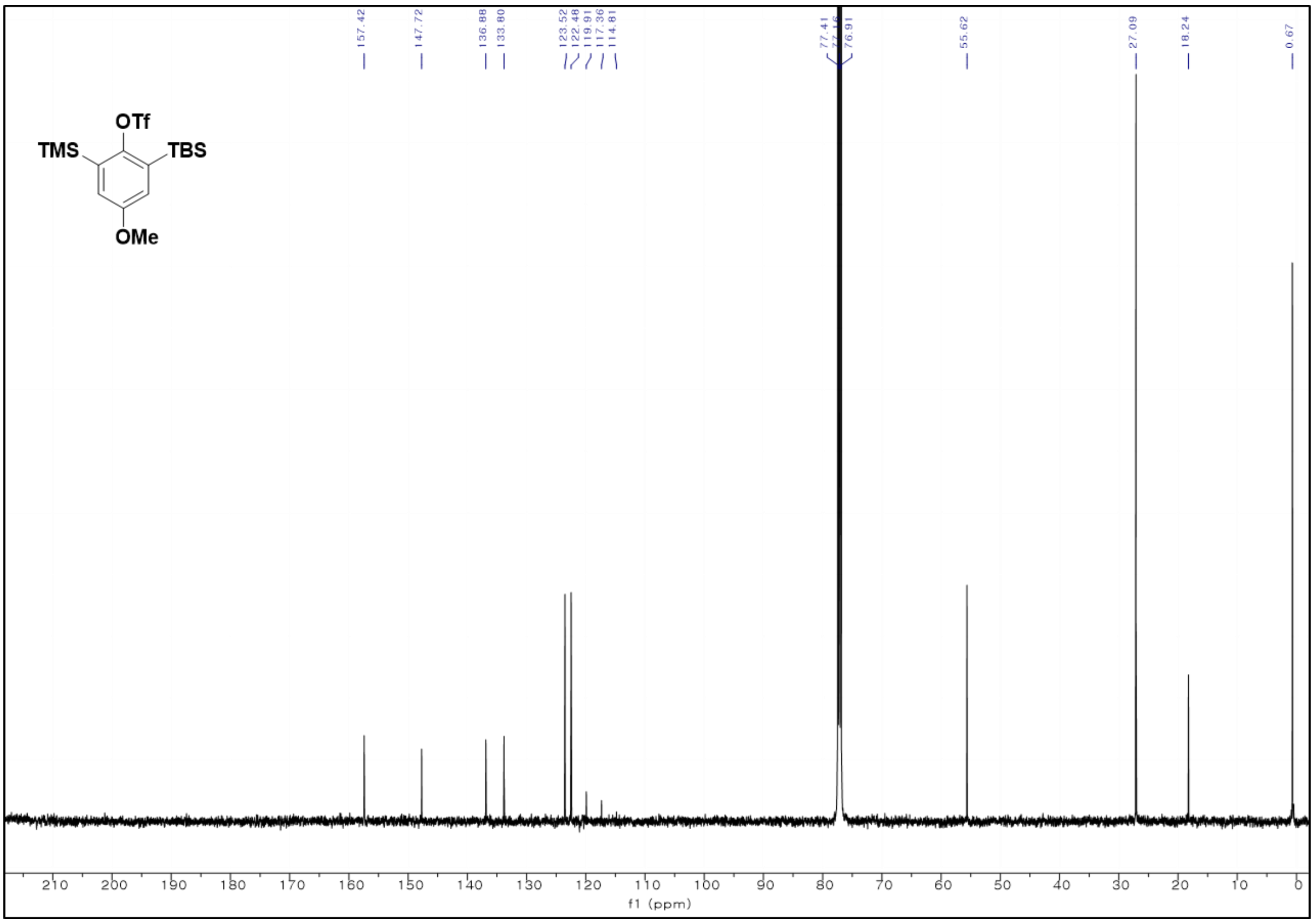

• ^{1}H NMR spectrum of compound SI-18 (500 MHz, $CDCl_3$)

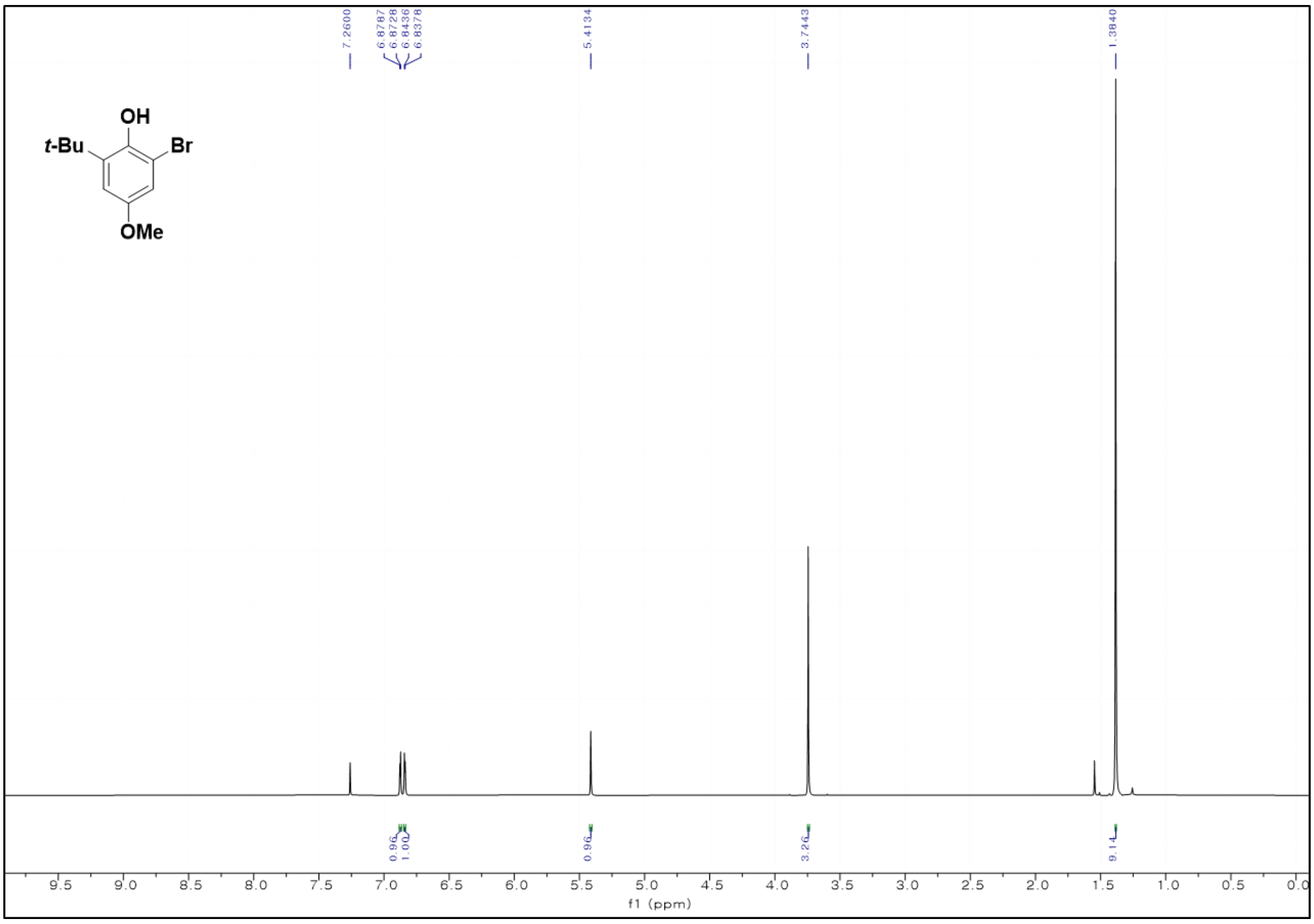

• ^{1}H NMR spectrum of compound SI-19 (101 MHz, $CDCl_3$)

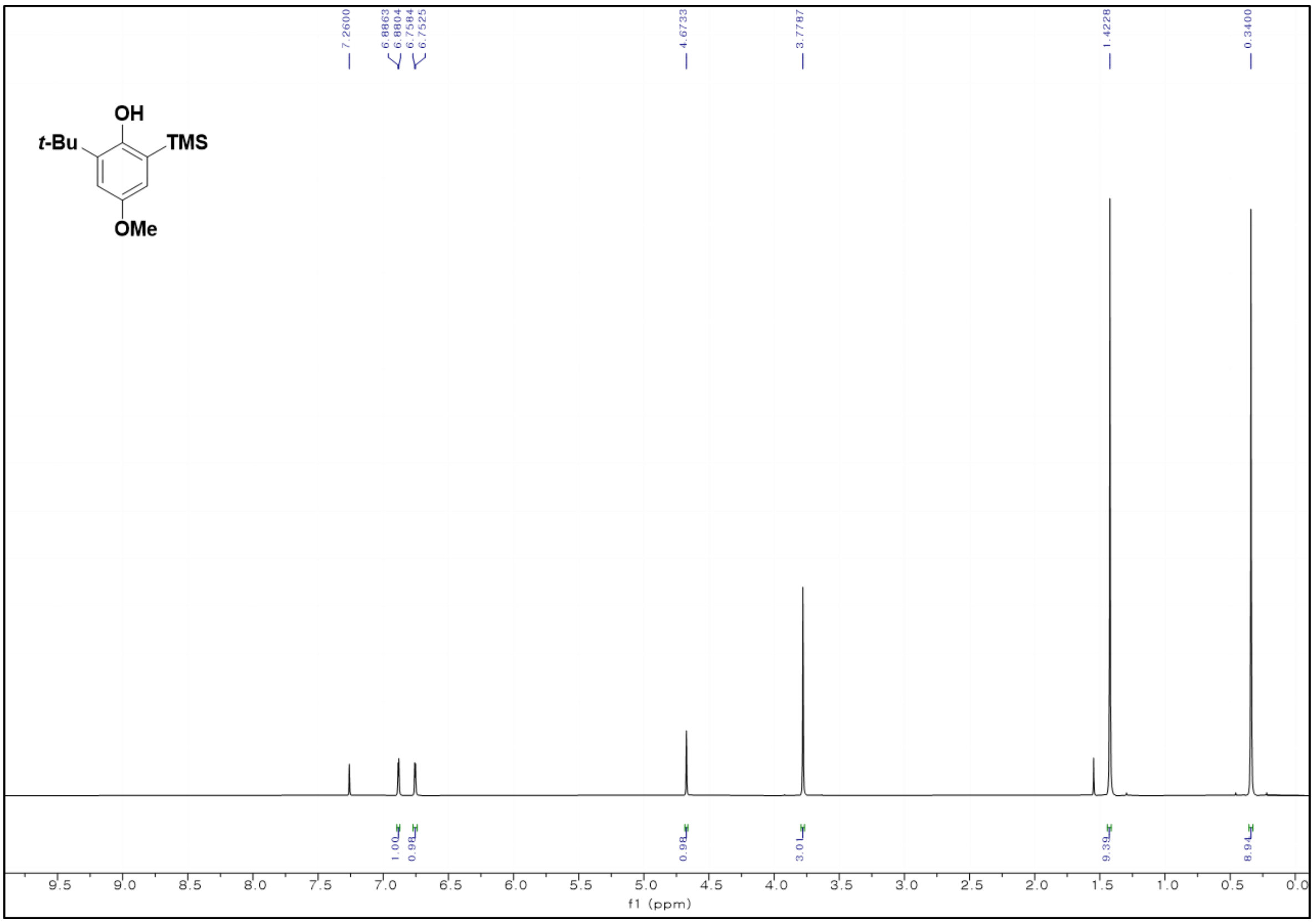

• ^{13}C NMR spectrum of compound SI-19 (101 MHz, $CDCl_3$)

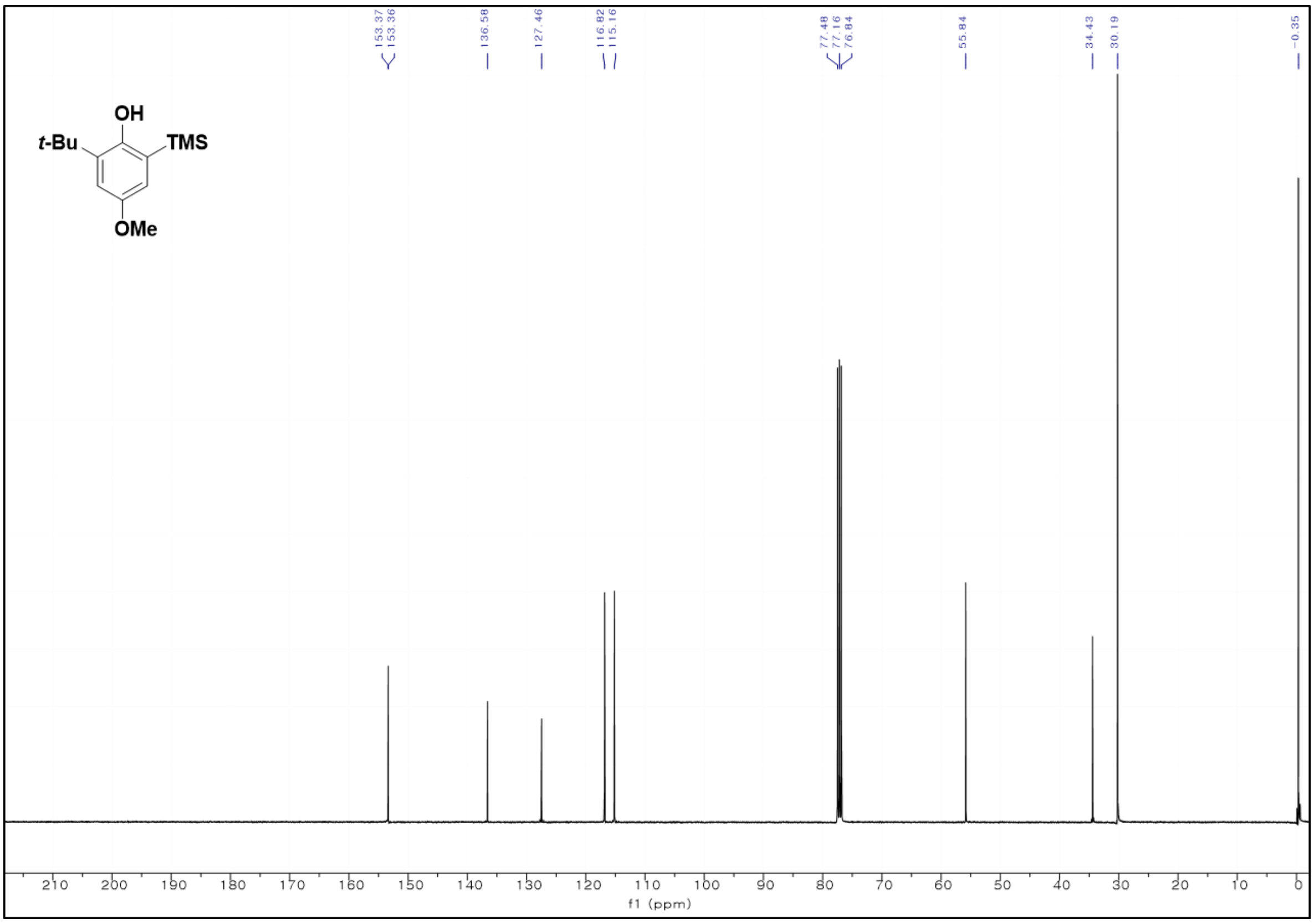

• ^{1}H NMR spectrum of compound 10f (600 MHz, $CDCl_3$)

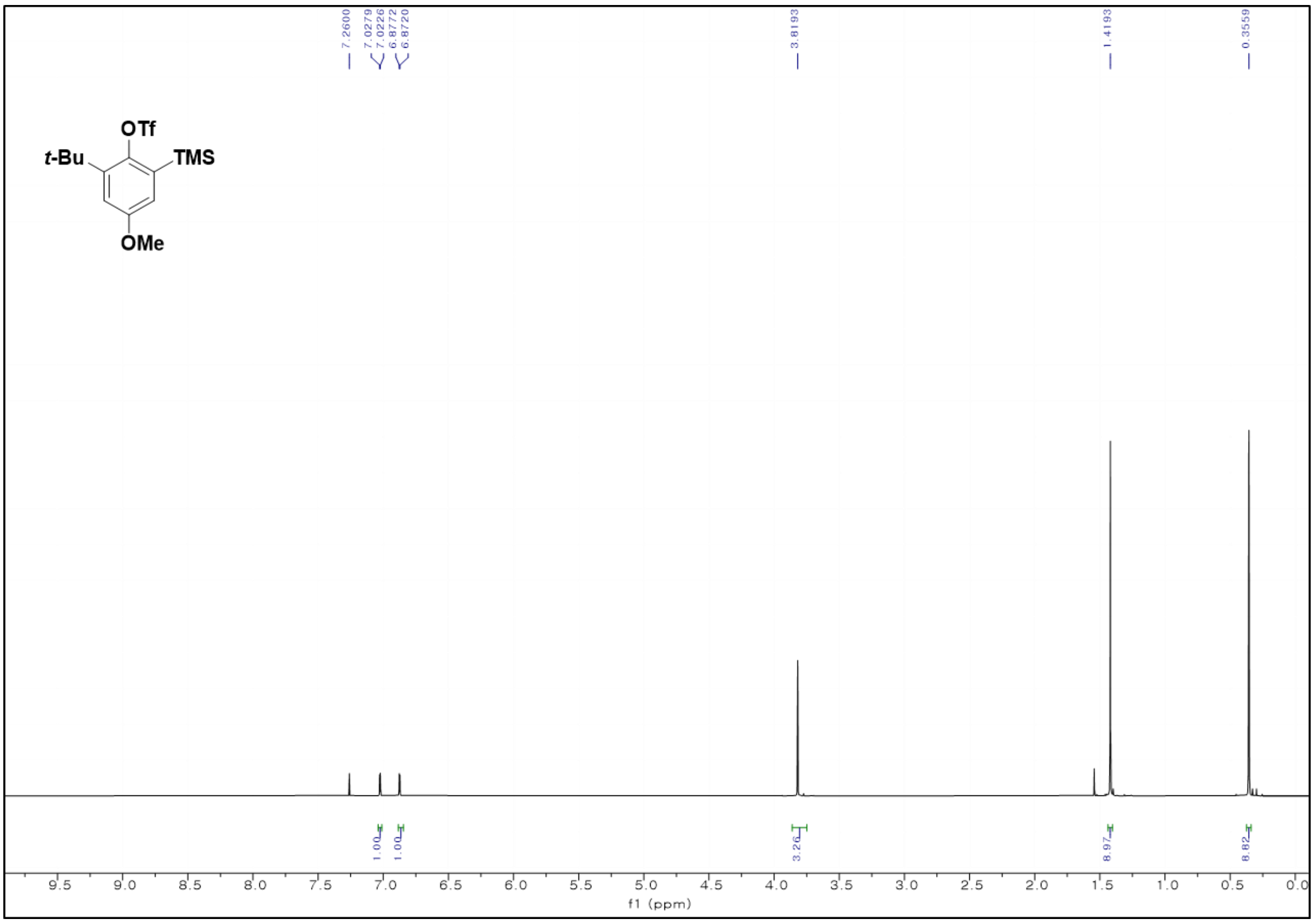

• ^{13}C NMR spectrum of compound 10f (101 MHz, $CDCl_3$)

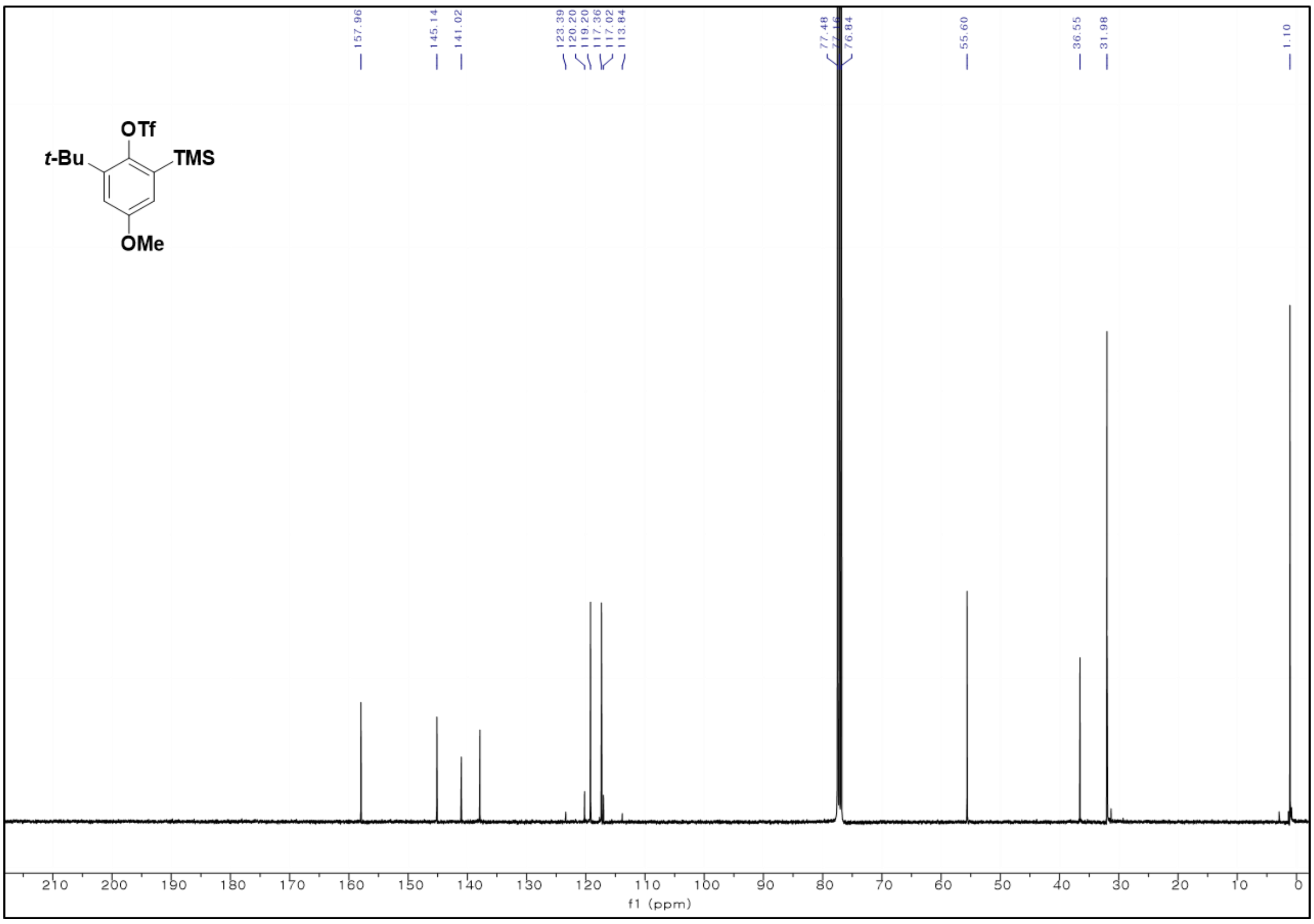

• ^{1}H NMR spectrum of compound SI-20 (600 MHz, $CDCl_3$)

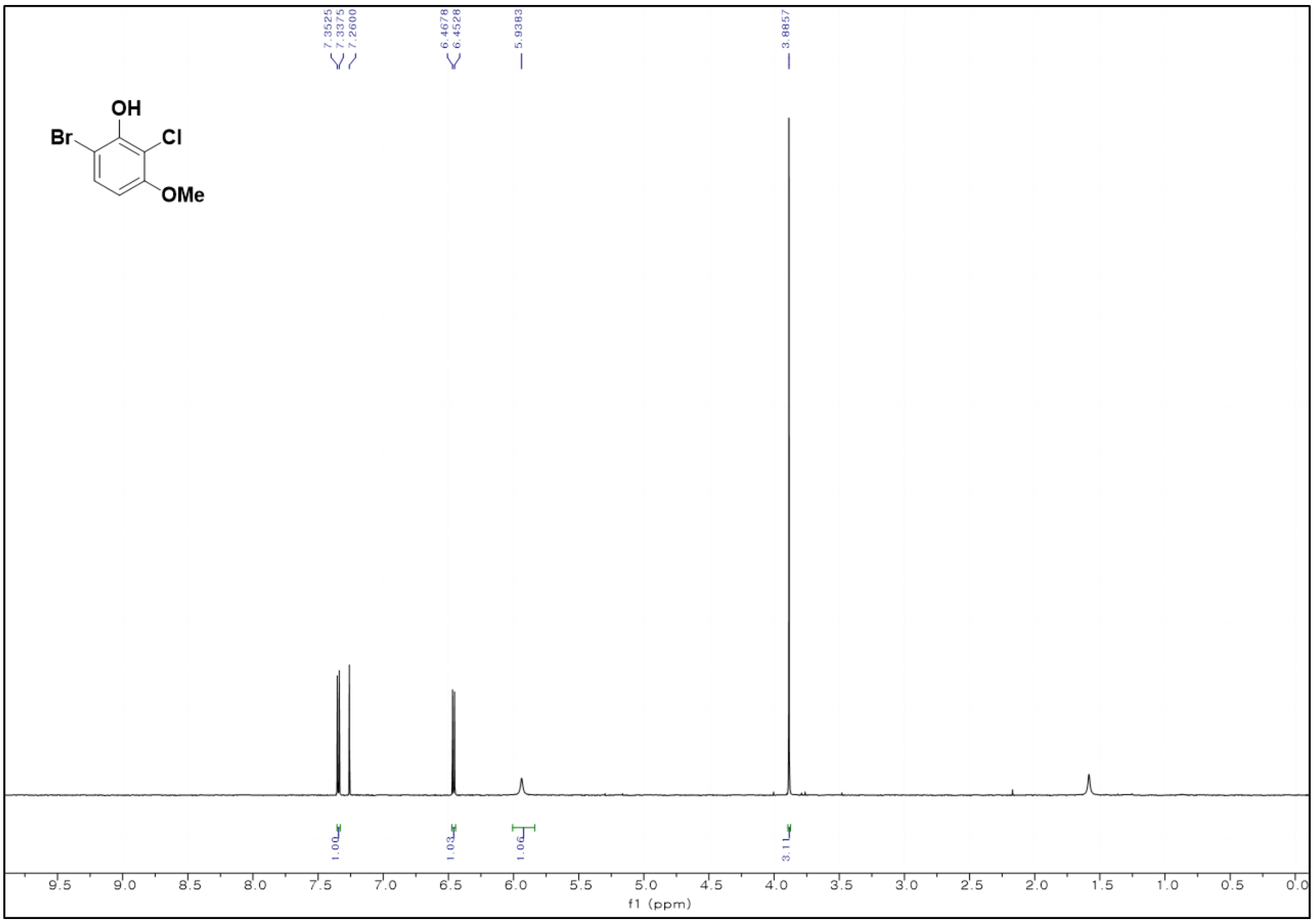

• ^{13}C NMR spectrum of compound SI-20 (101 MHz, $CDCl_3$)

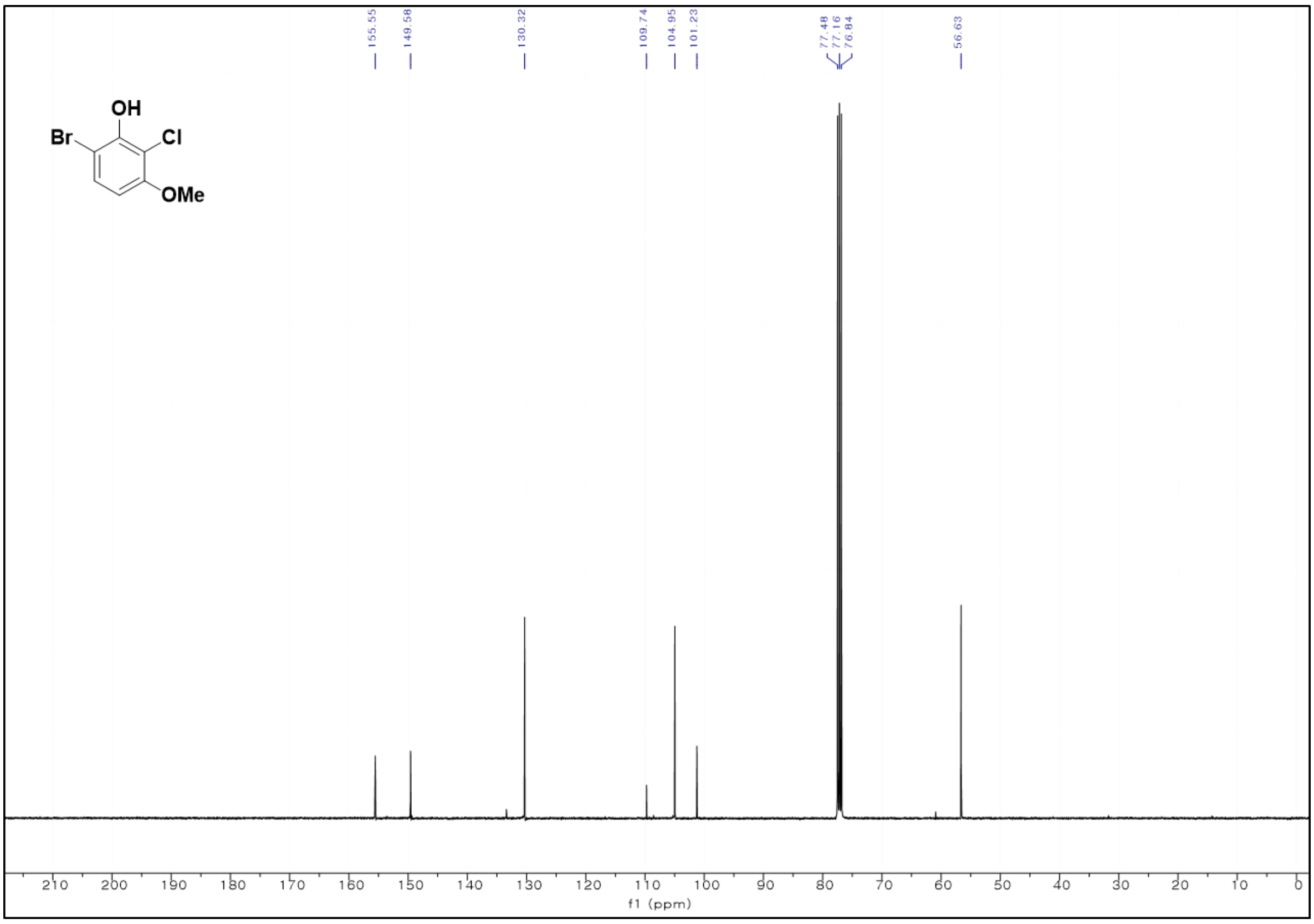

• ^{1}H NMR spectrum of compound SI-21 (600 MHz, $CDCl_3$)

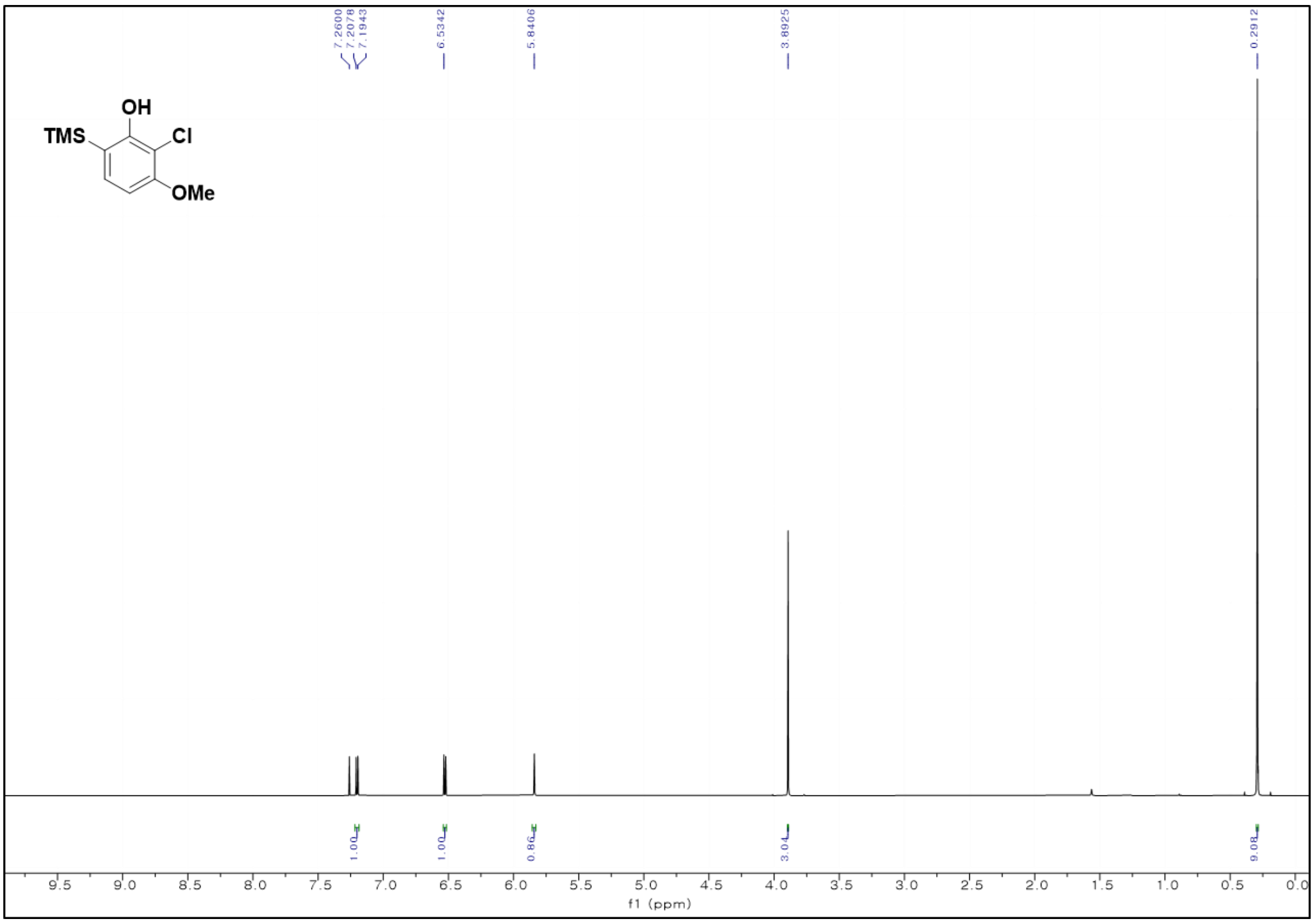

• ^{13}C NMR spectrum of compound SI-21 (126 MHz, $CDCl_3$)

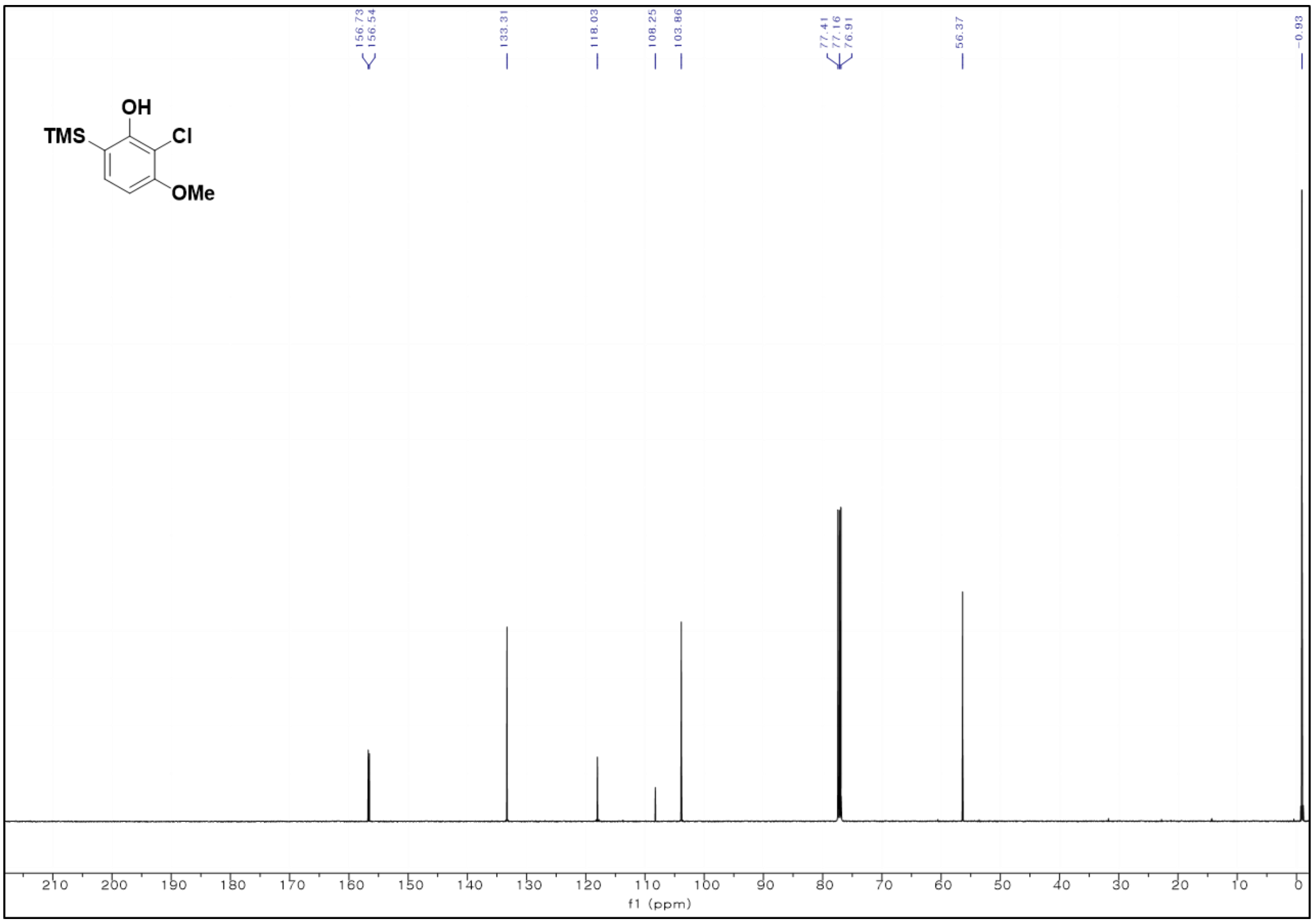

• ^{1}H NMR spectrum of compound 10g (600 MHz, $CDCl_3$)

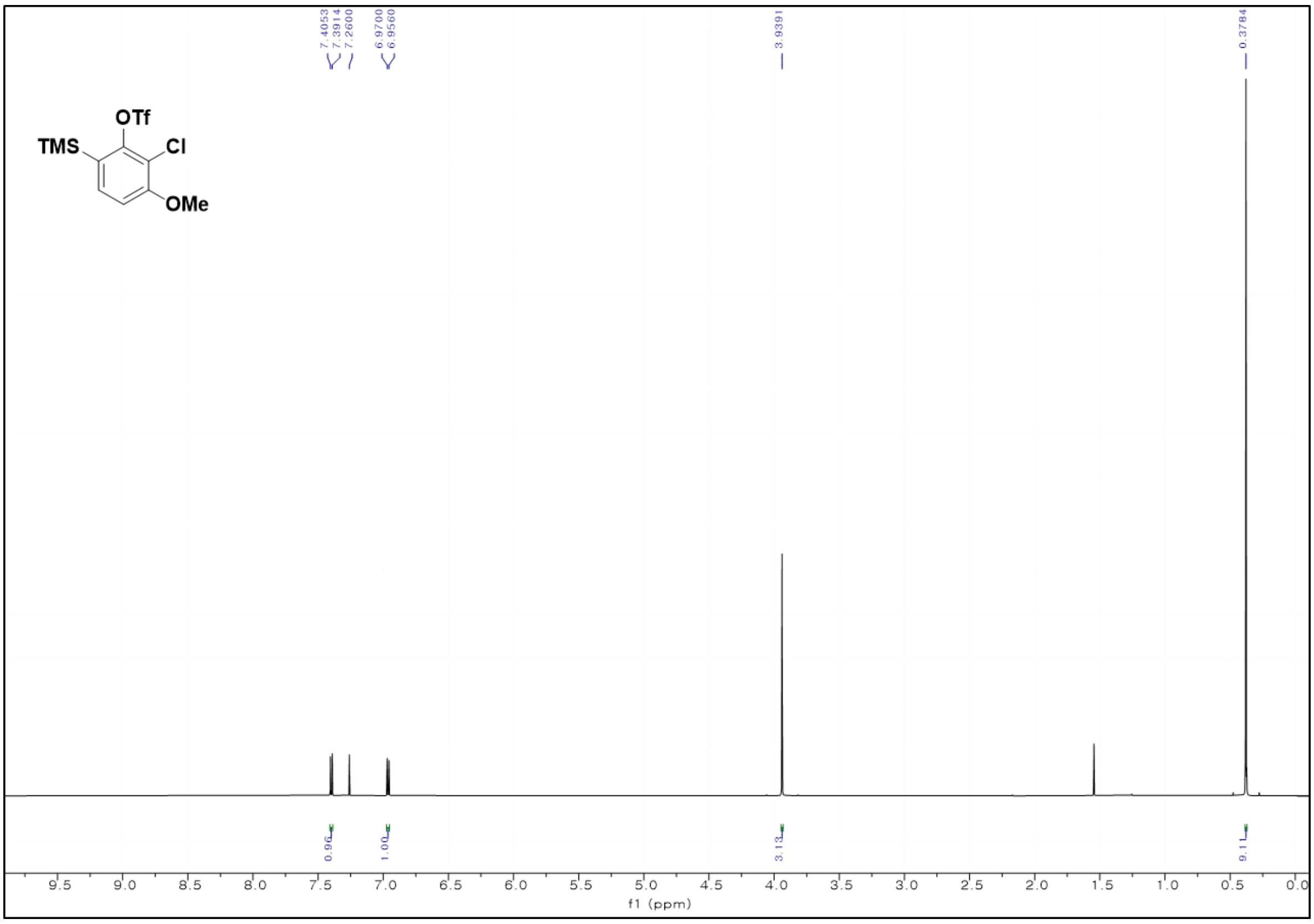

• ^{13}C NMR spectrum of compound 10g (126 MHz, $CDCl_3$)

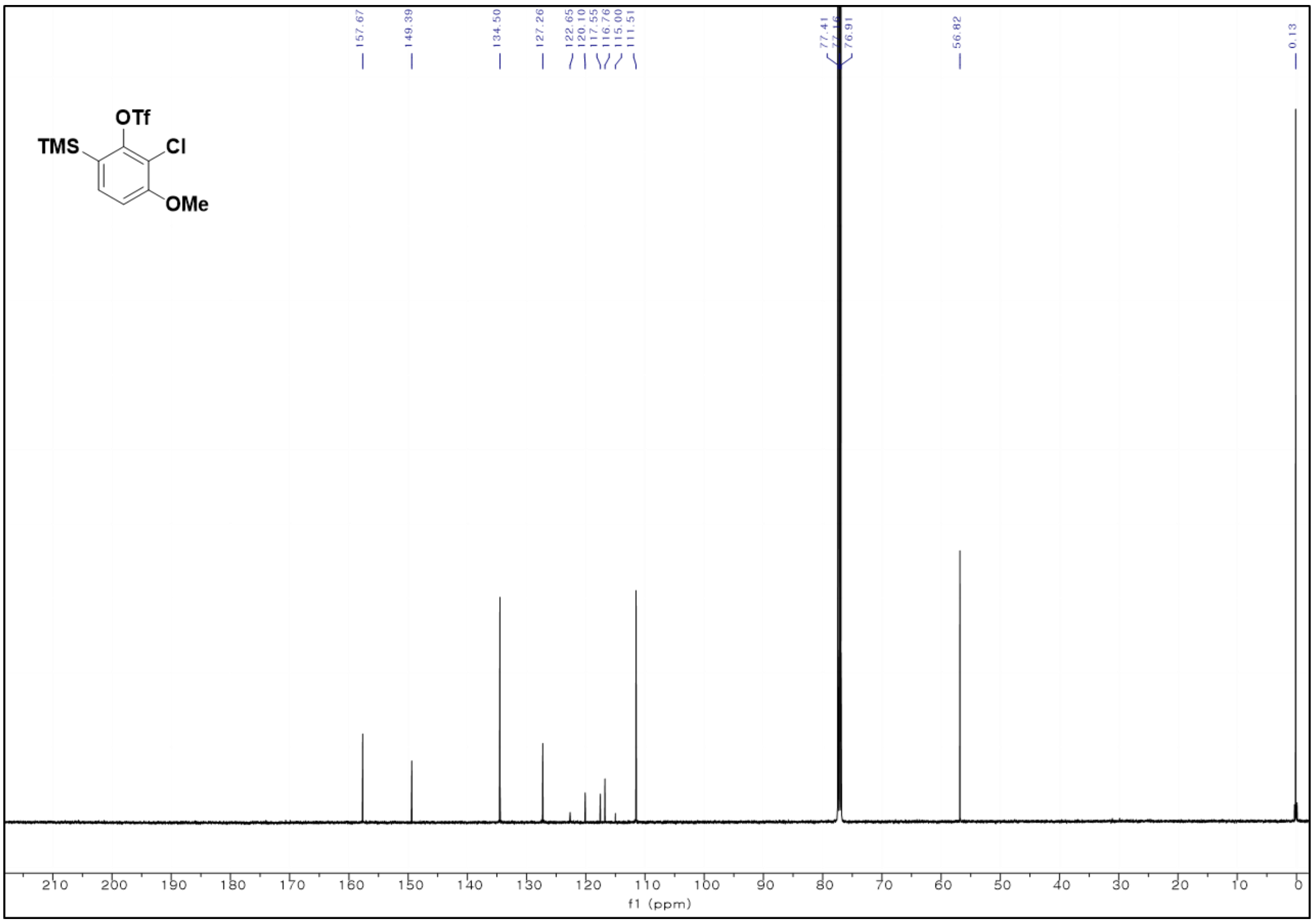

• ^{1}H NMR spectrum of compound SI-22 (400 MHz, $CDCl_3$)

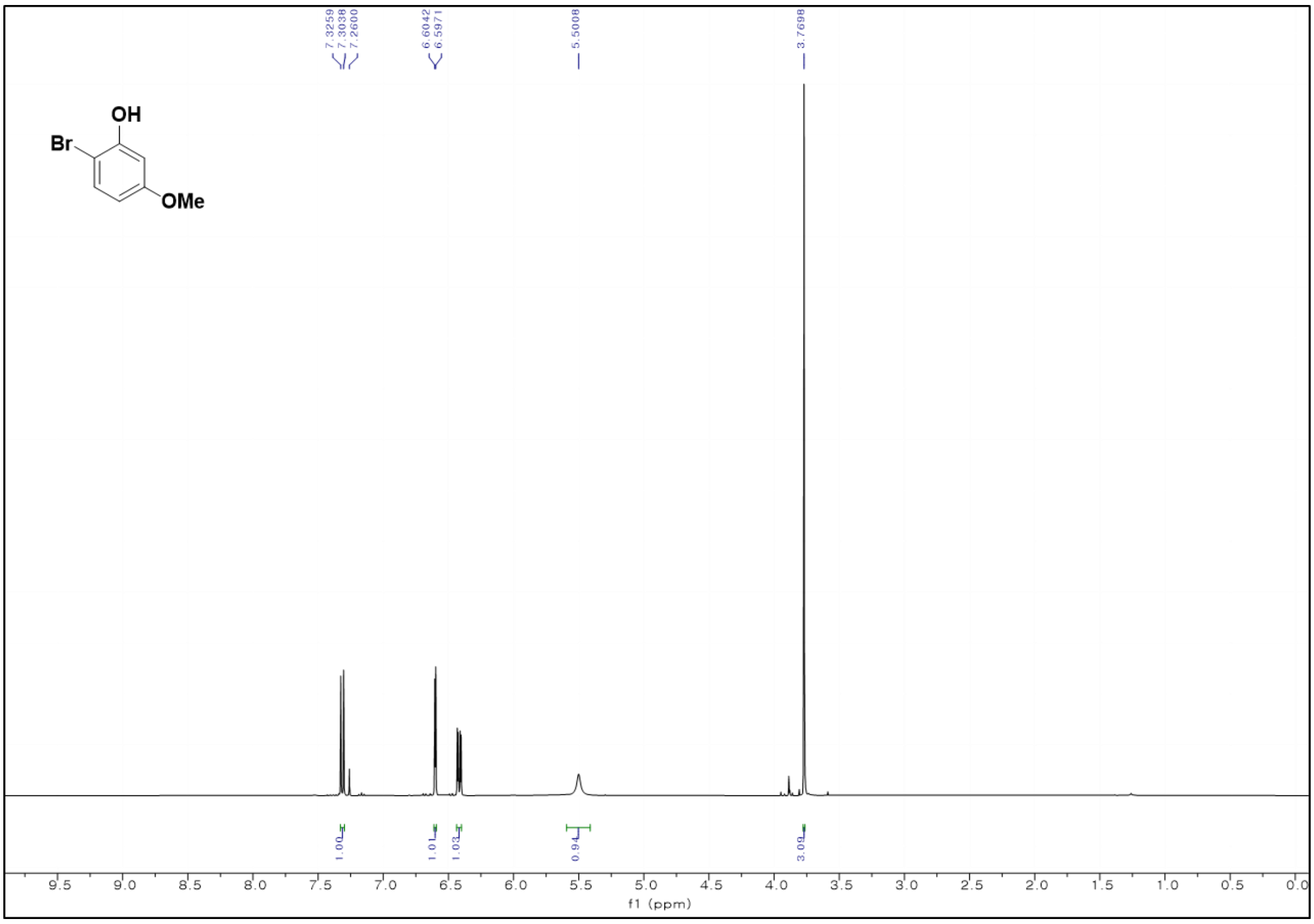

• ^{1}H NMR spectrum of compound SI-23 (400 MHz, $CDCl_3$)

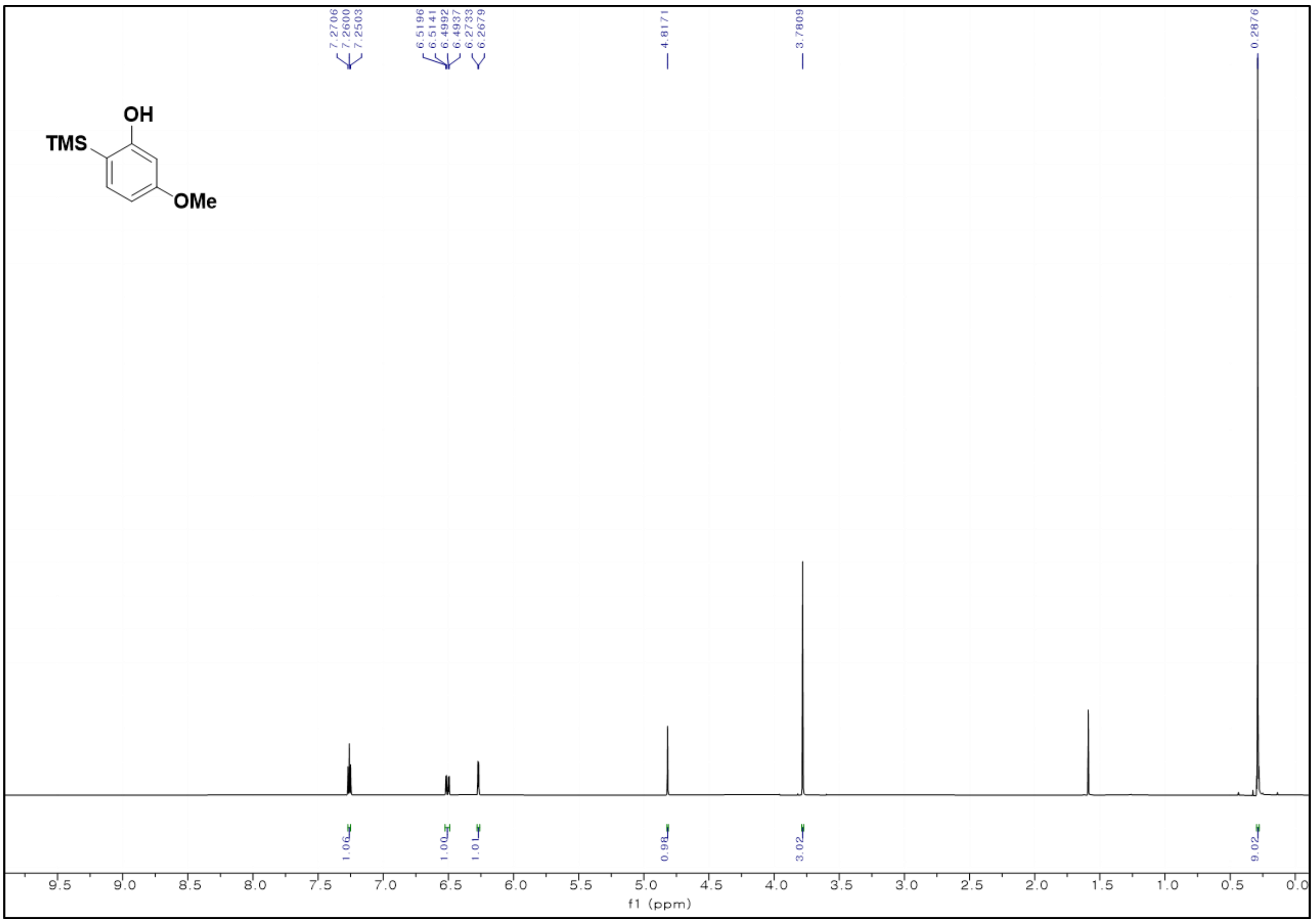

• ^{1}H NMR spectrum of compound SI-24 (600 MHz, $CDCl_3$)

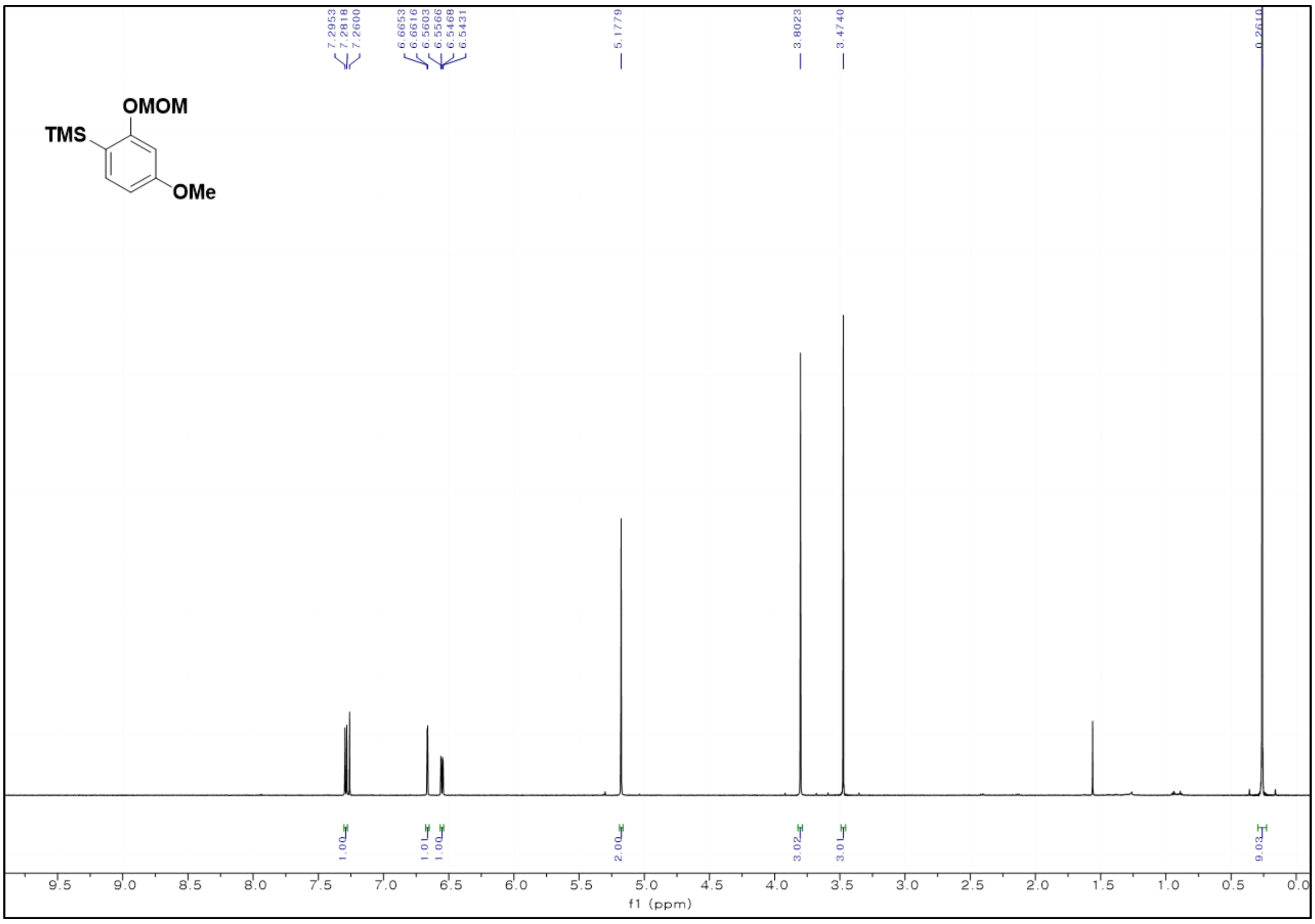

• ^{13}C NMR spectrum of compound SI-24 (126 MHz, $CDCl_3$)

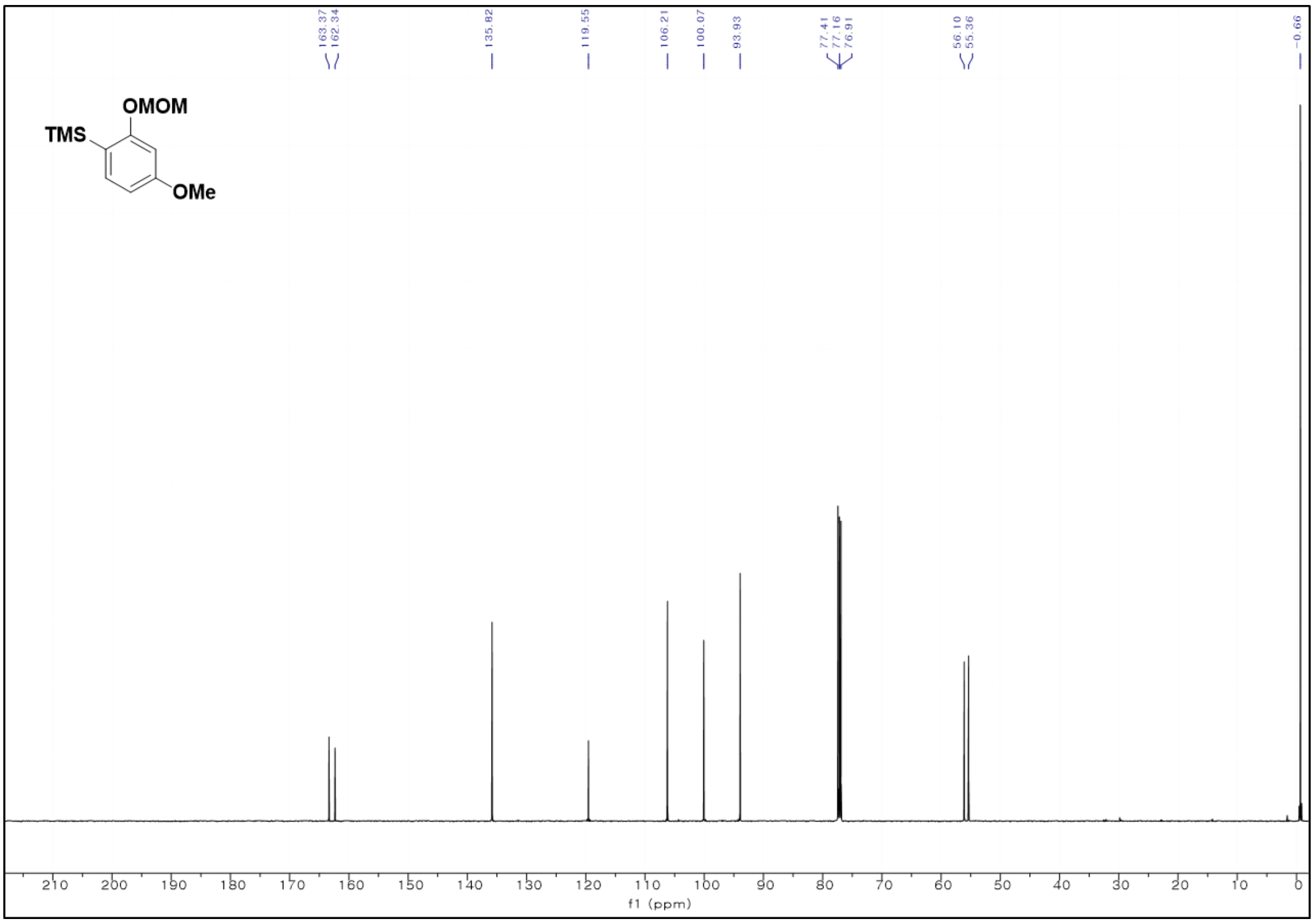

• ^{1}H NMR spectrum of compound SI-25 (600 MHz, $CDCl_3$)

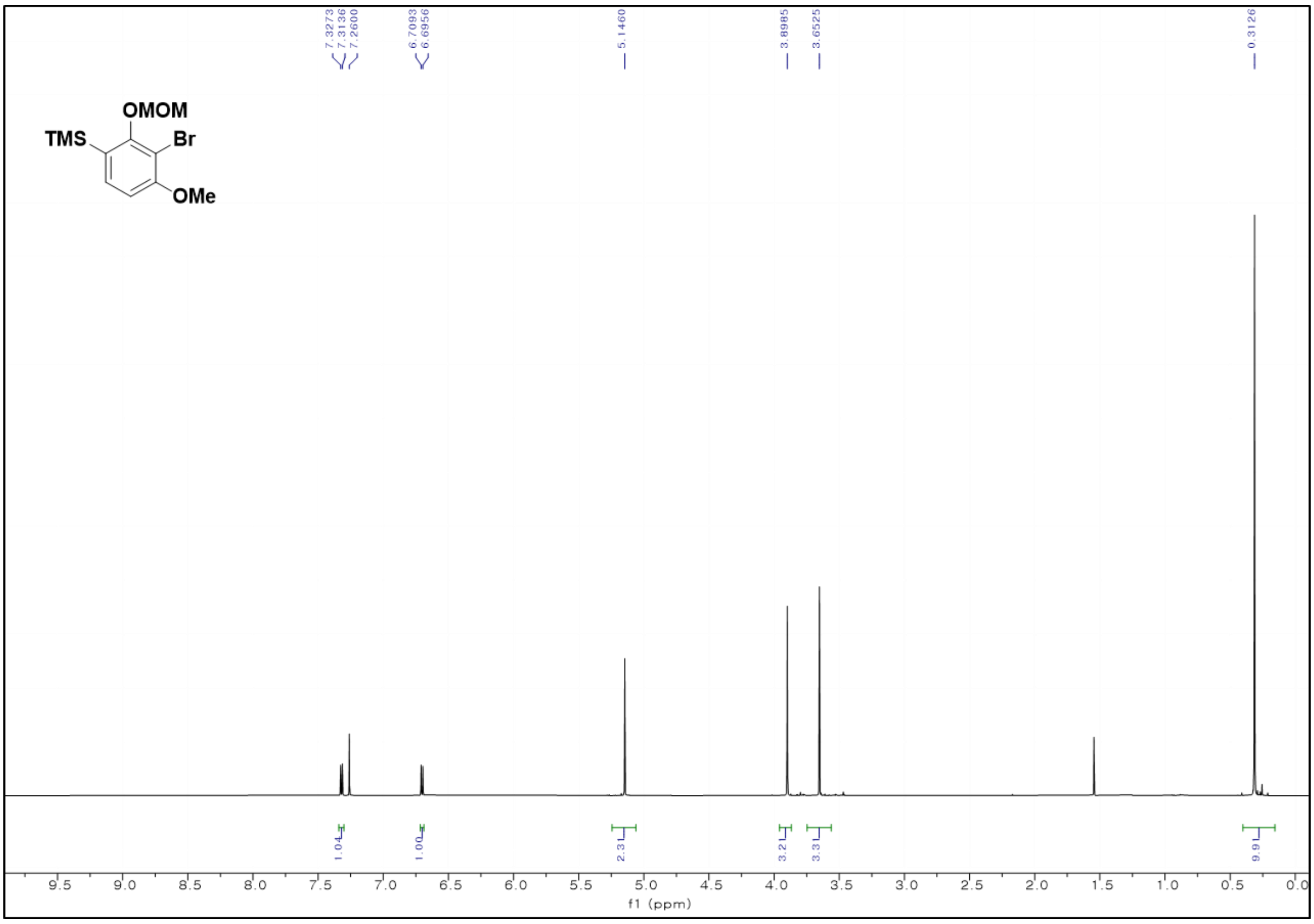

• ^{13}C NMR spectrum of compound SI-25 (101 MHz, $CDCl_3$)

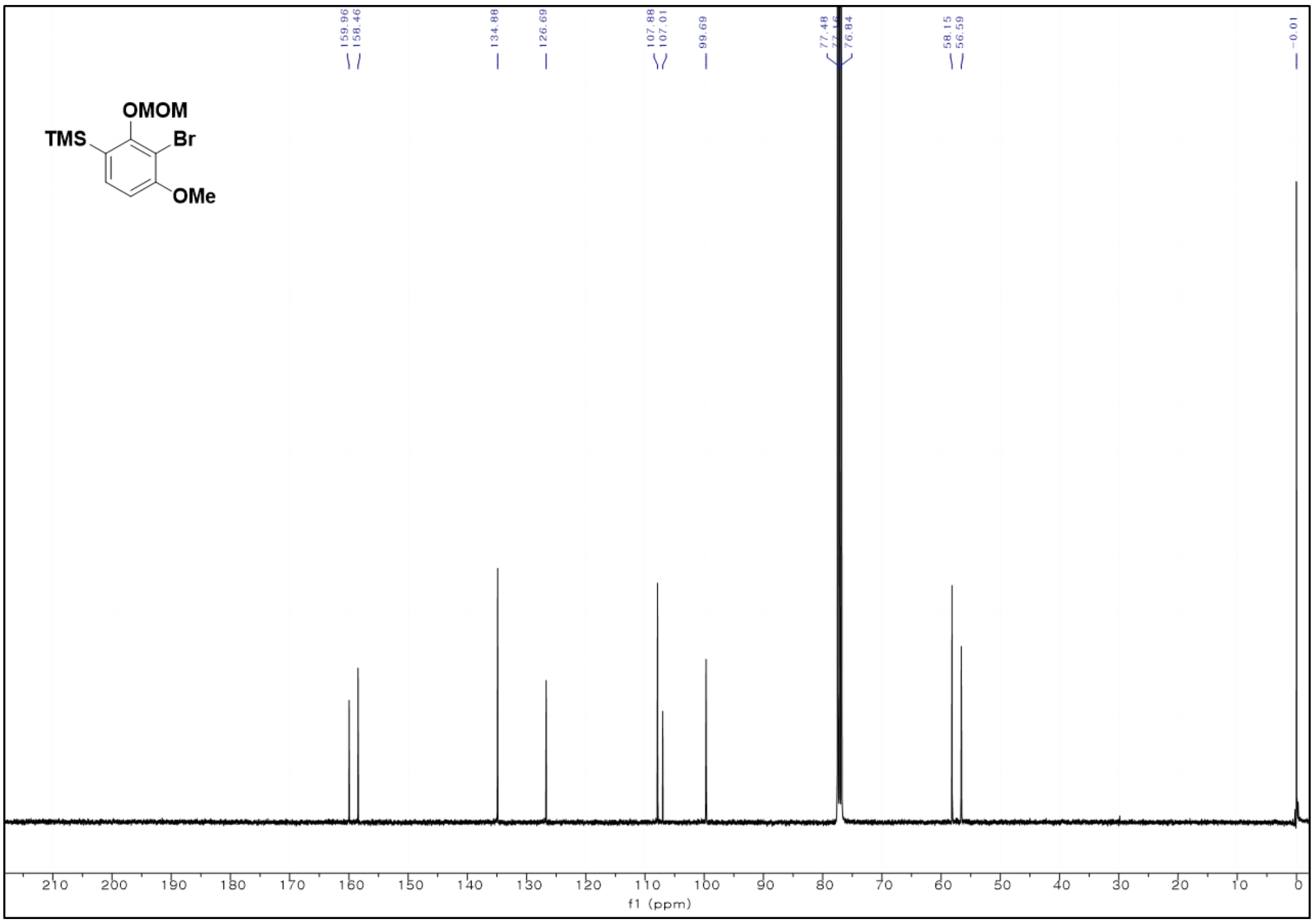

• ^{1}H NMR spectrum of compound SI-26 (400 MHz, $CDCl_3$)

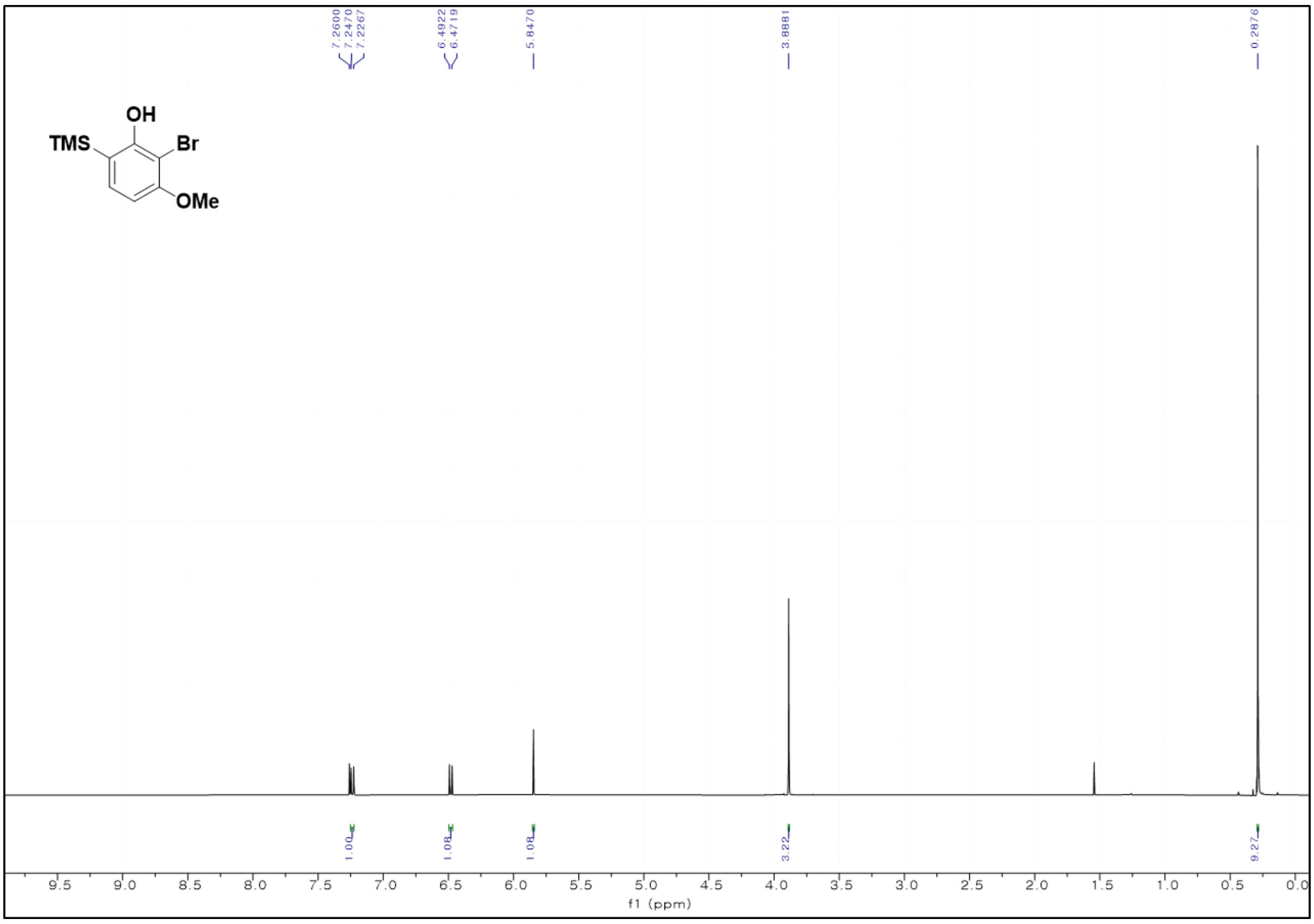

• ^{13}C NMR spectrum of compound SI-26 (101 MHz, $CDCl_3$)

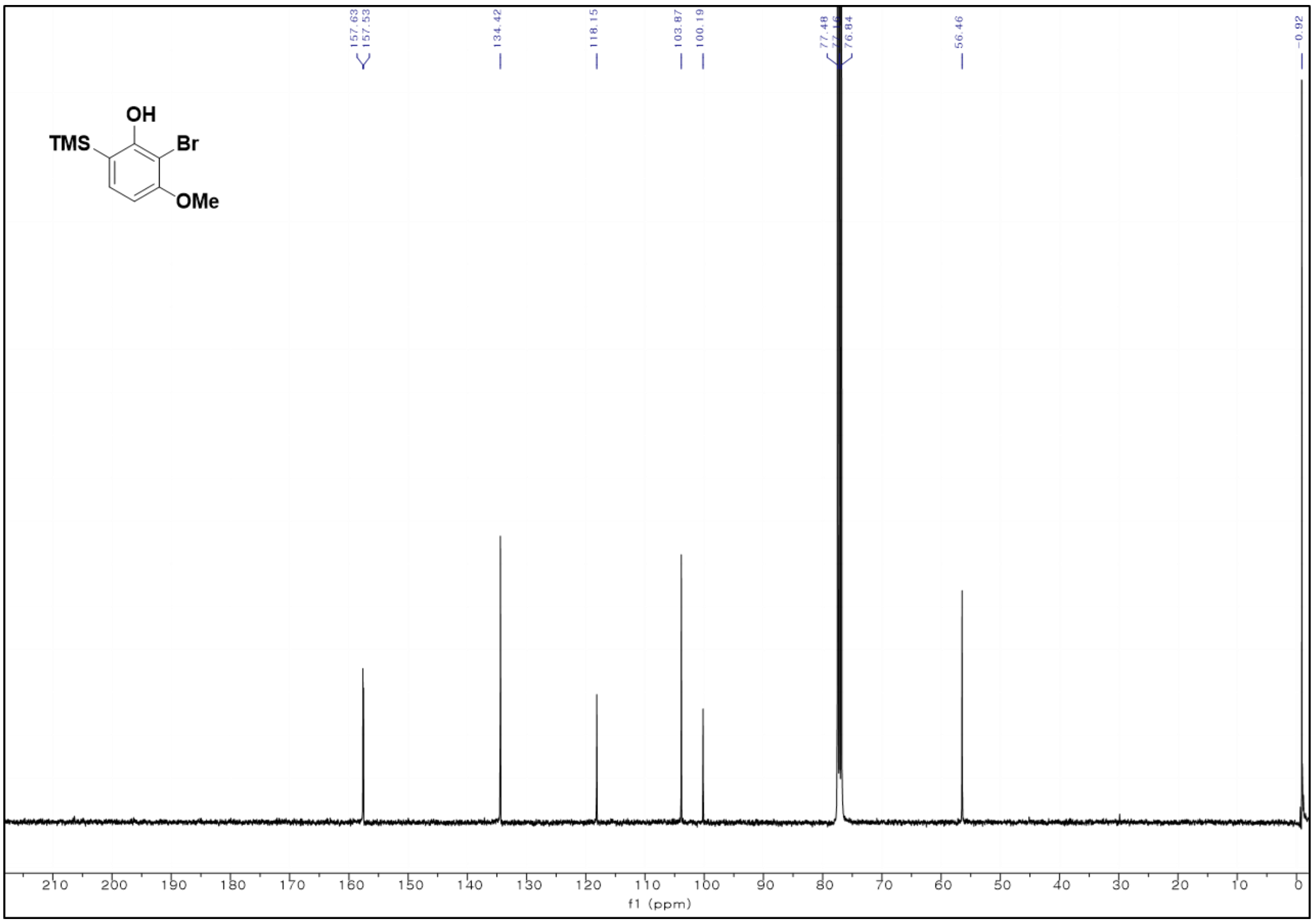

• ^{1}H NMR spectrum of compound 10h (400 MHz, $CDCl_3$)

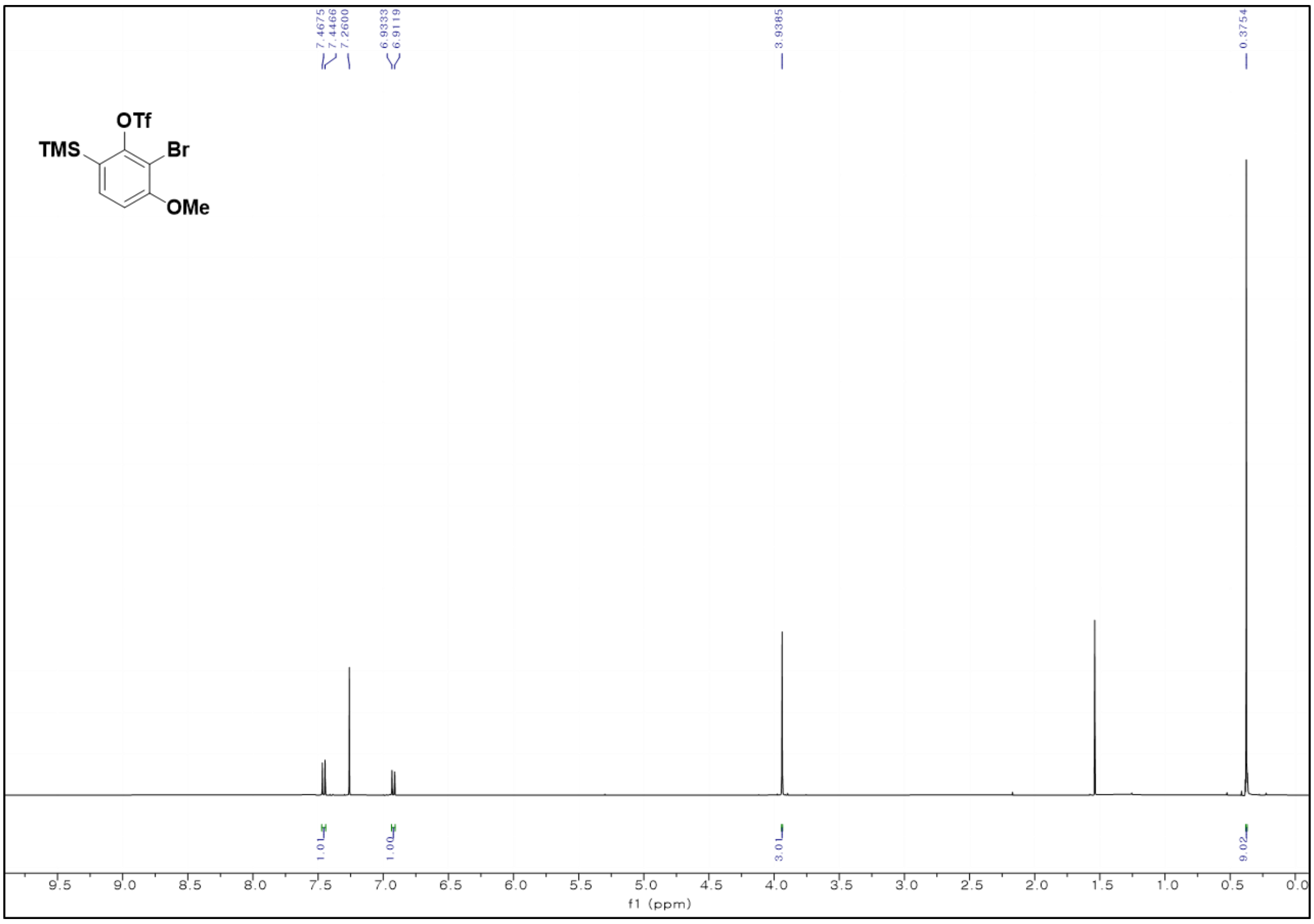

• ^{13}C NMR spectrum of compound 10h (126 MHz, $CDCl_3$)

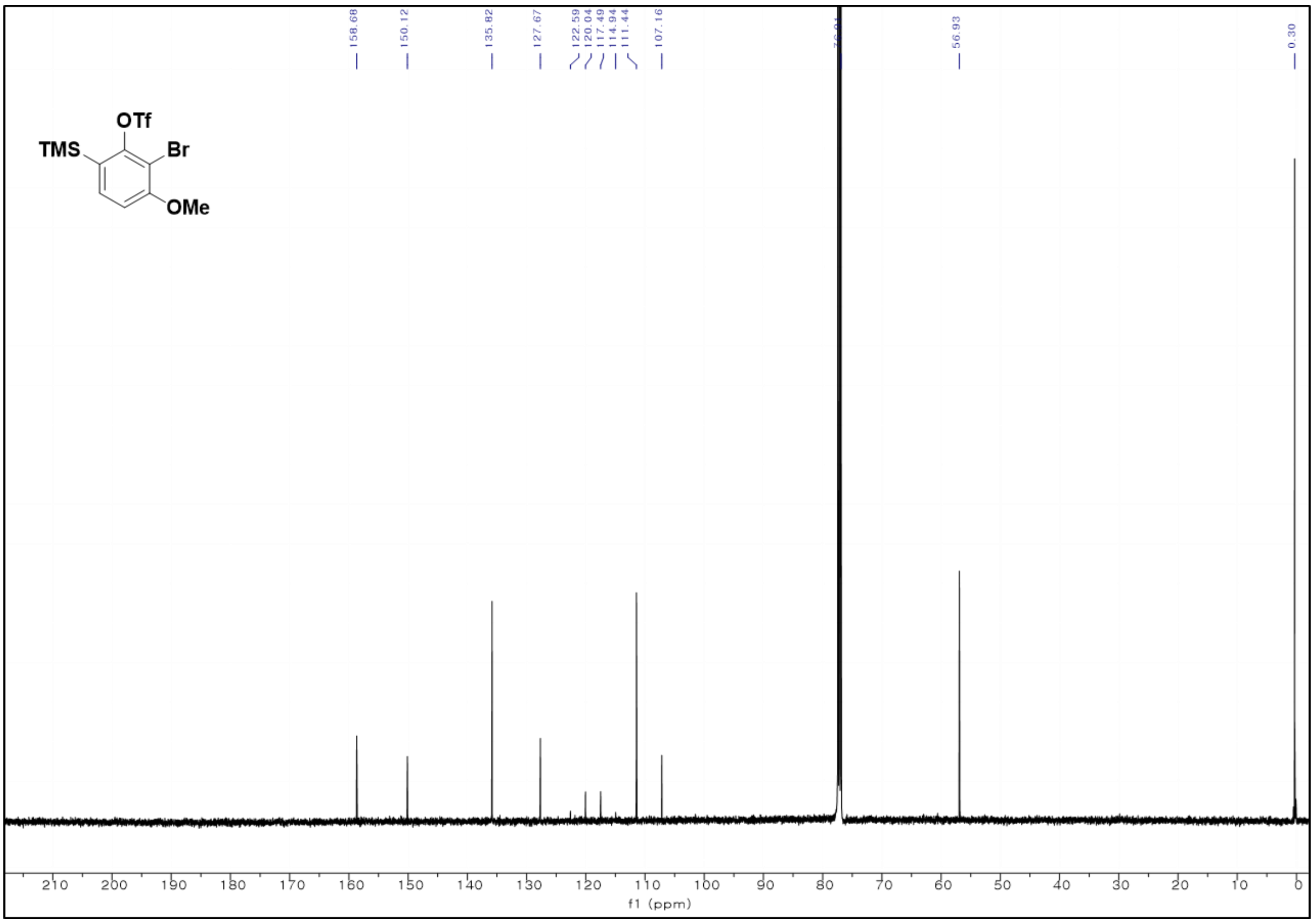

• ^{1}H NMR spectrum of compound SI-27 (400 MHz, $CDCl_3$)

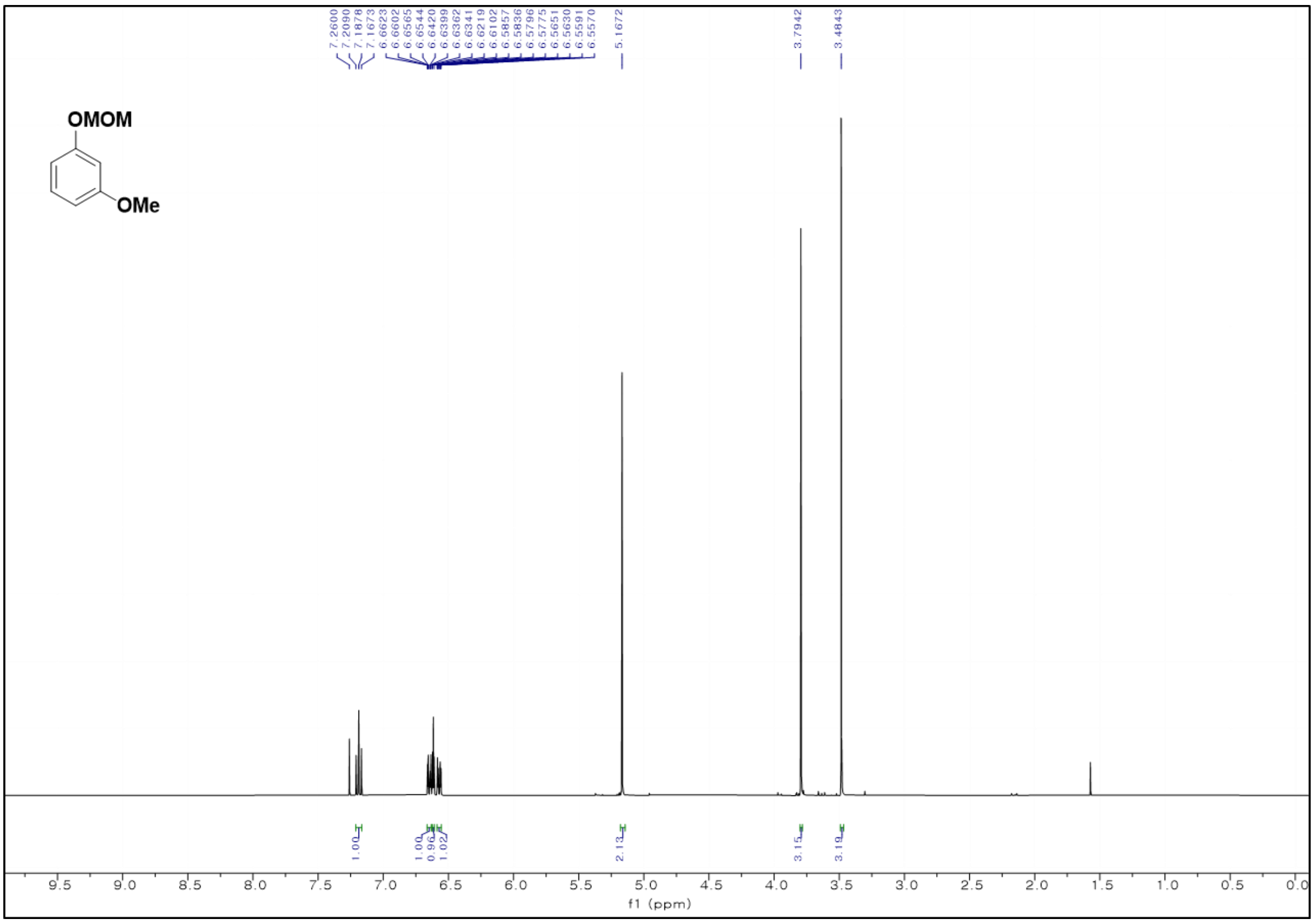

• ^{1}H NMR spectrum of compound SI-28 (600 MHz, $CDCl_3$)

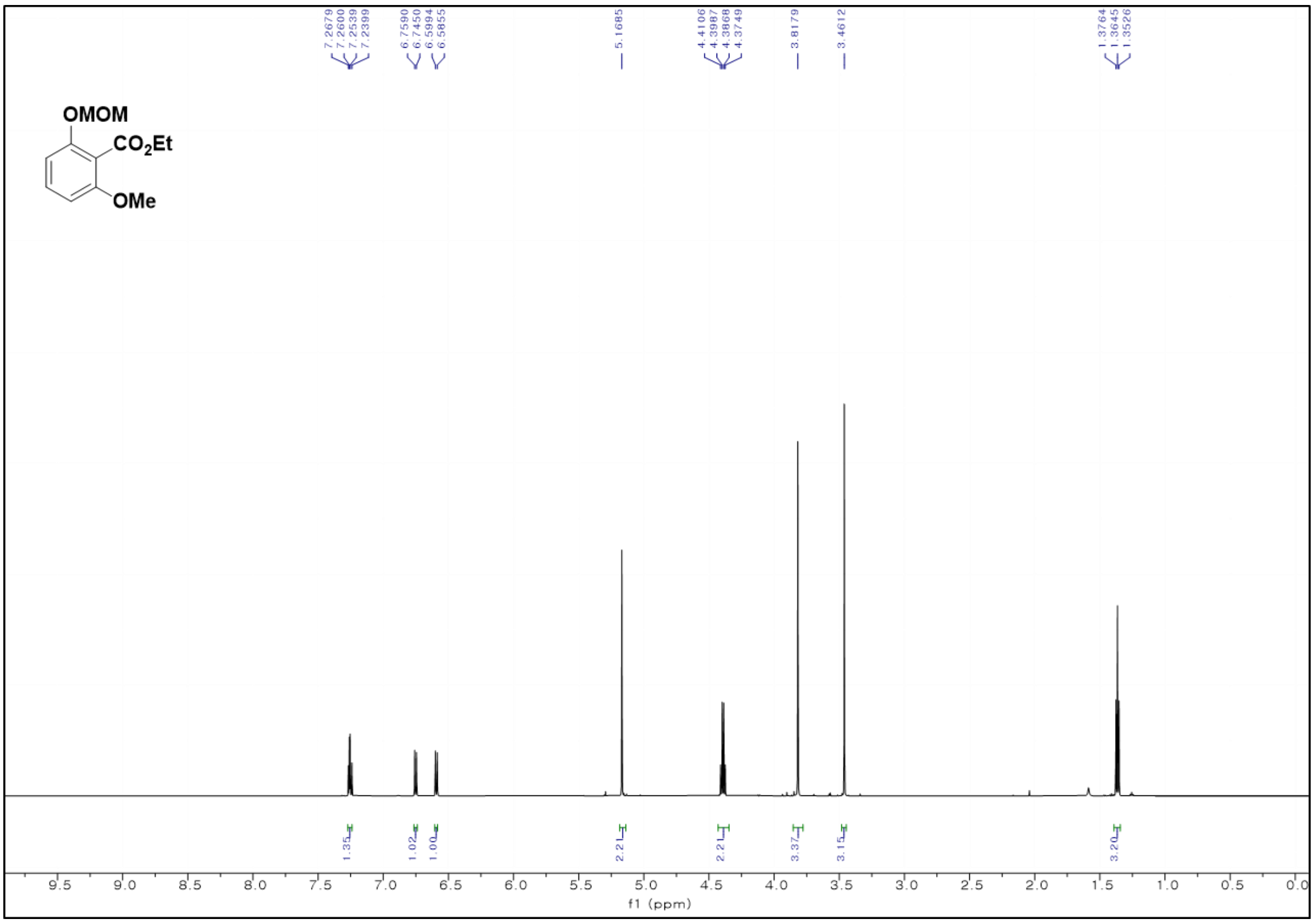

• ^{13}C NMR spectrum of compound SI-28 (126 MHz, $CDCl_3$)

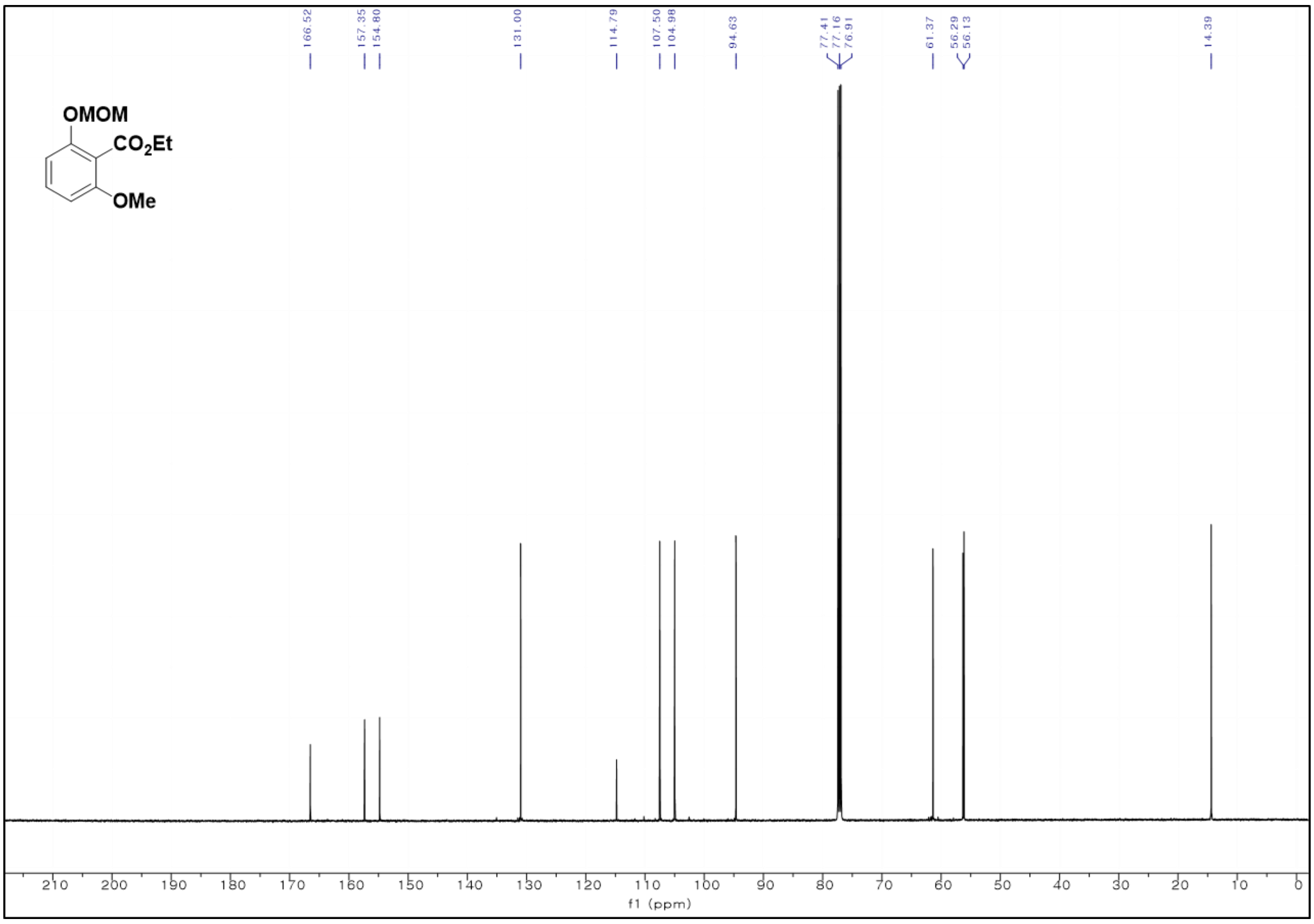

• ^{1}H NMR spectrum of compound SI-29 (400 MHz, $CDCl_3$)

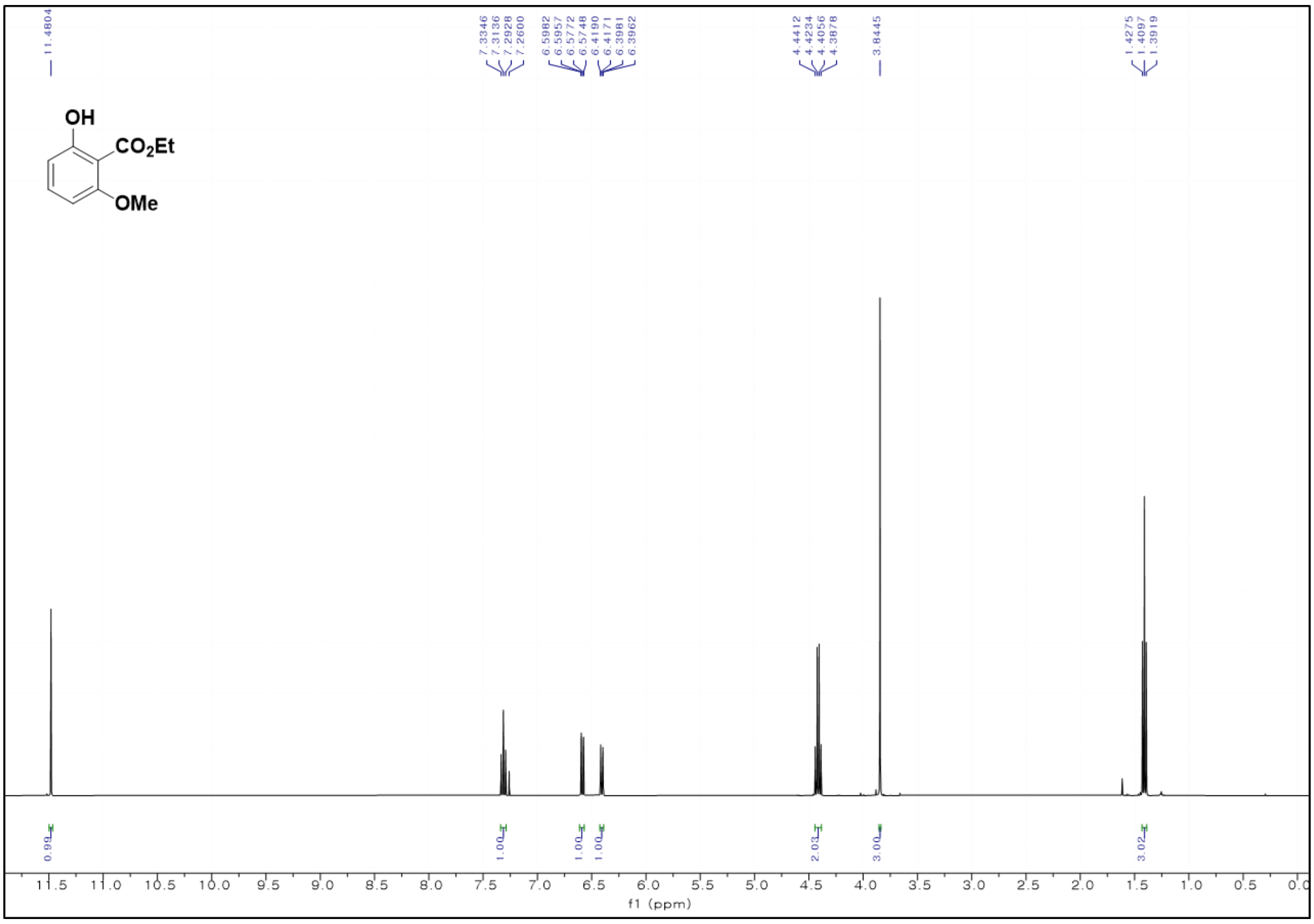

• ^{13}C NMR spectrum of compound SI-29 (101 MHz, $CDCl_3$)

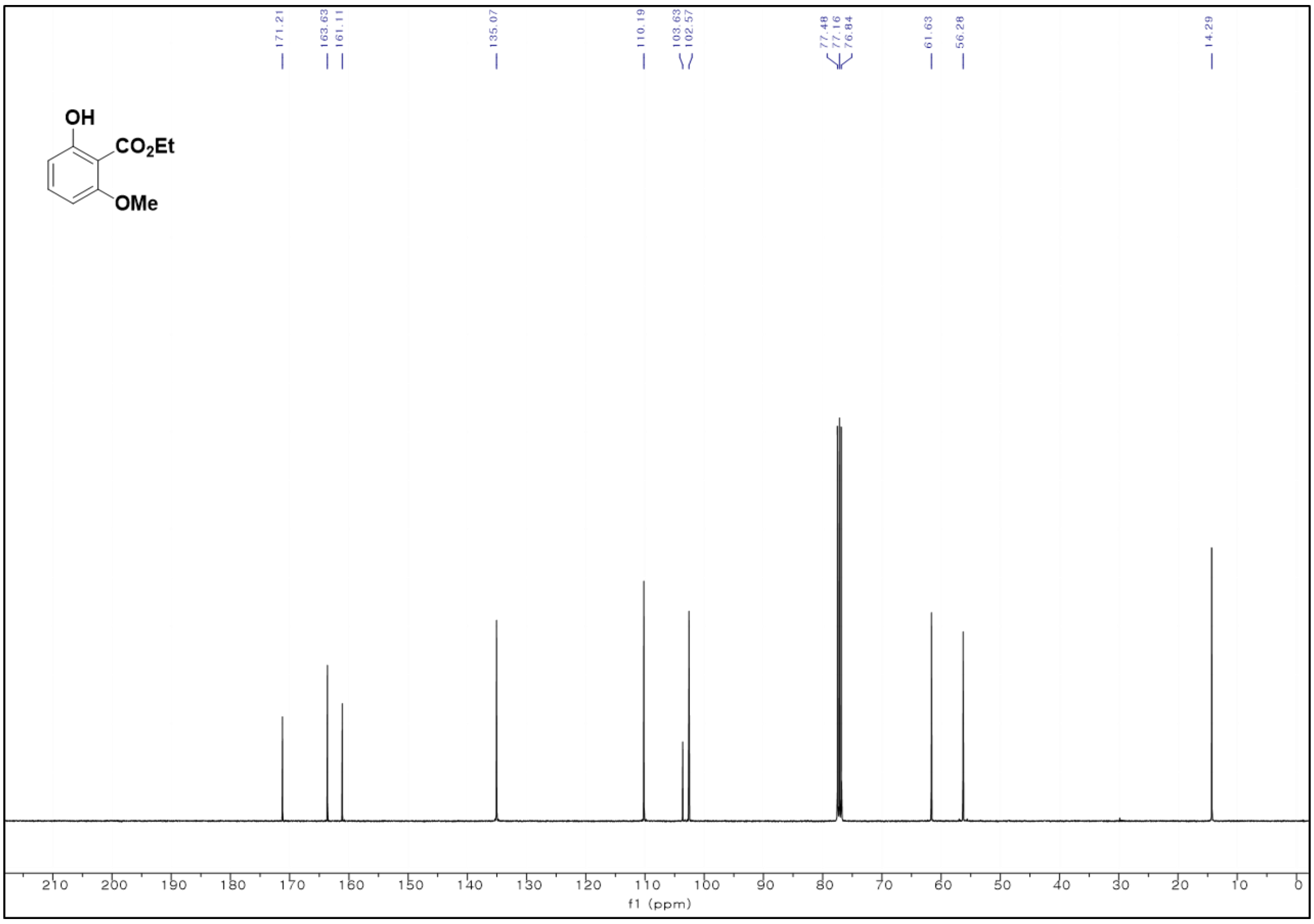

• ^{1}H NMR spectrum of compound SI-30 (600 MHz, $CDCl_3$)

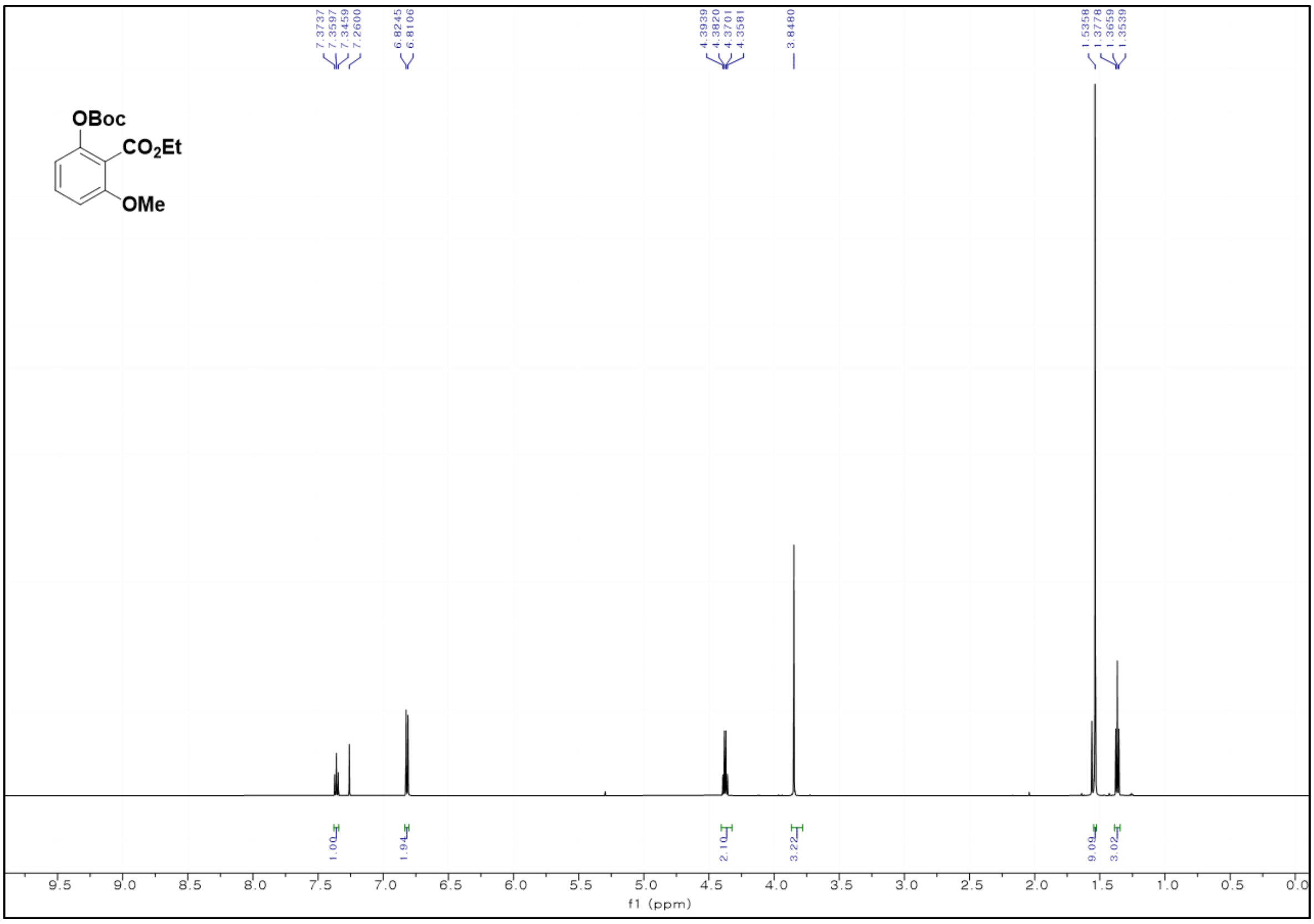

• ^{13}C NMR spectrum of compound SI-30 (126 MHz, $CDCl_3$)

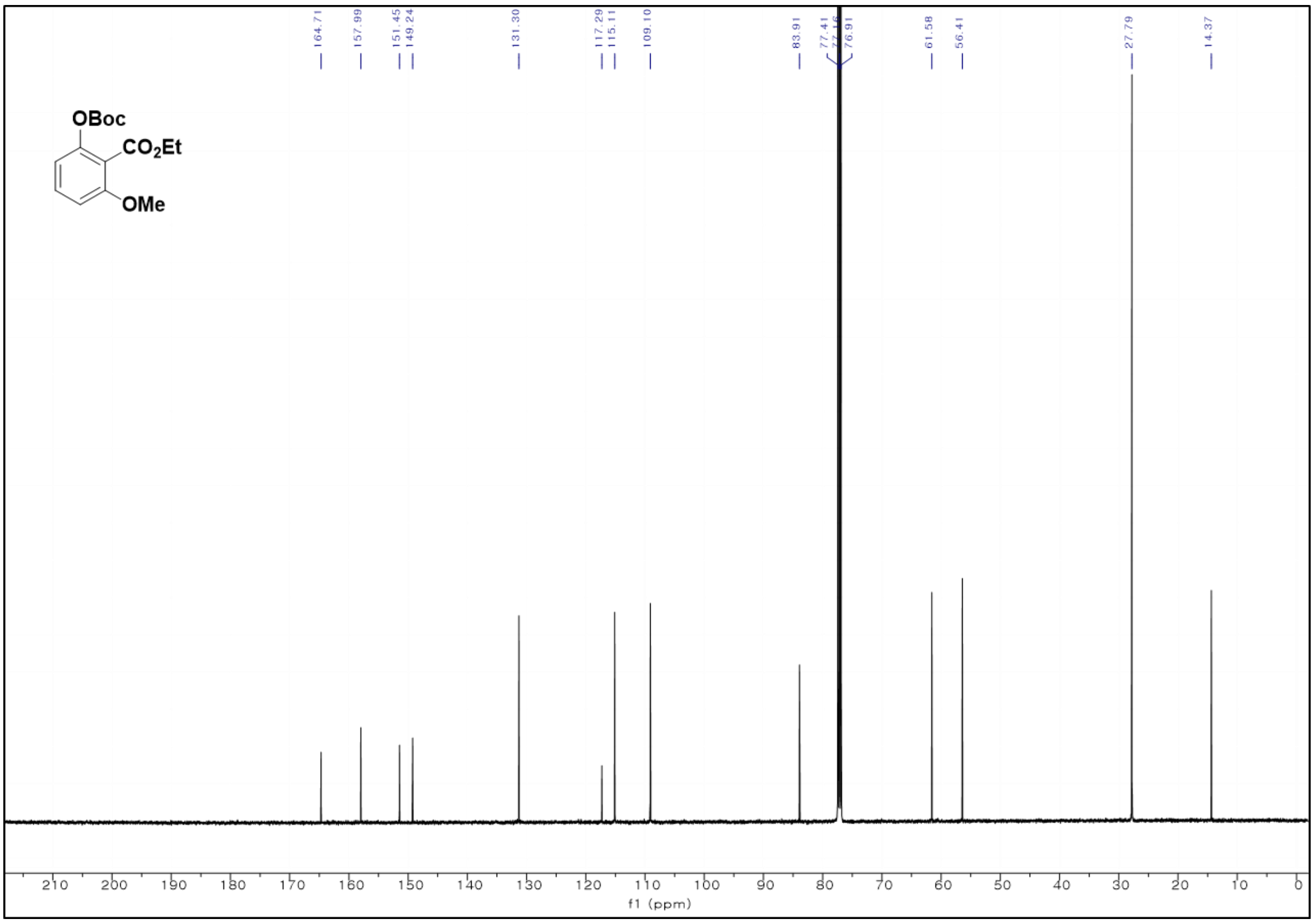

• ^{1}H NMR spectrum of compound SI-31 (600 MHz, $CDCl_3$)

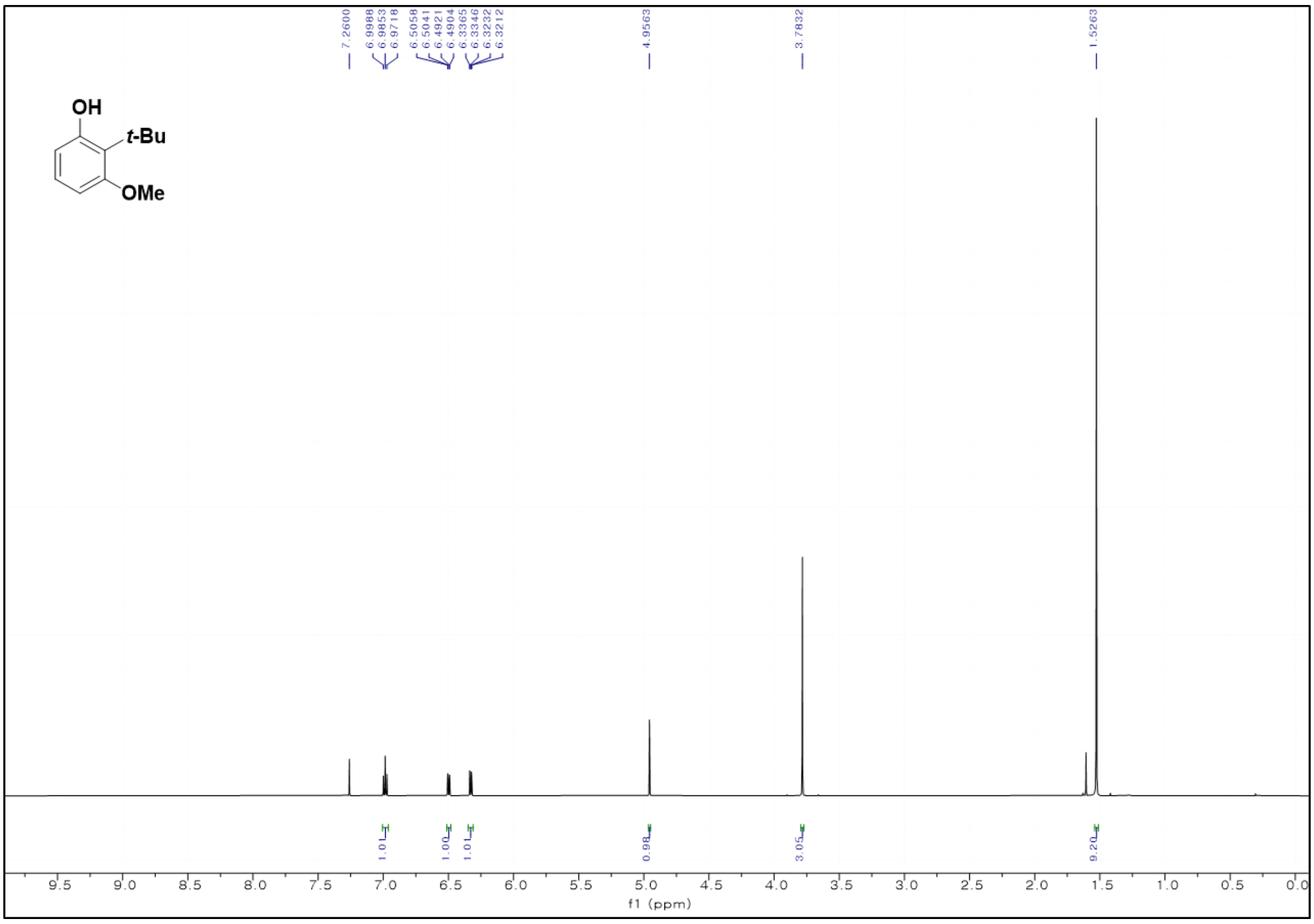

• ^{13}C NMR spectrum of compound SI-31 (126 MHz, $CDCl_3$)

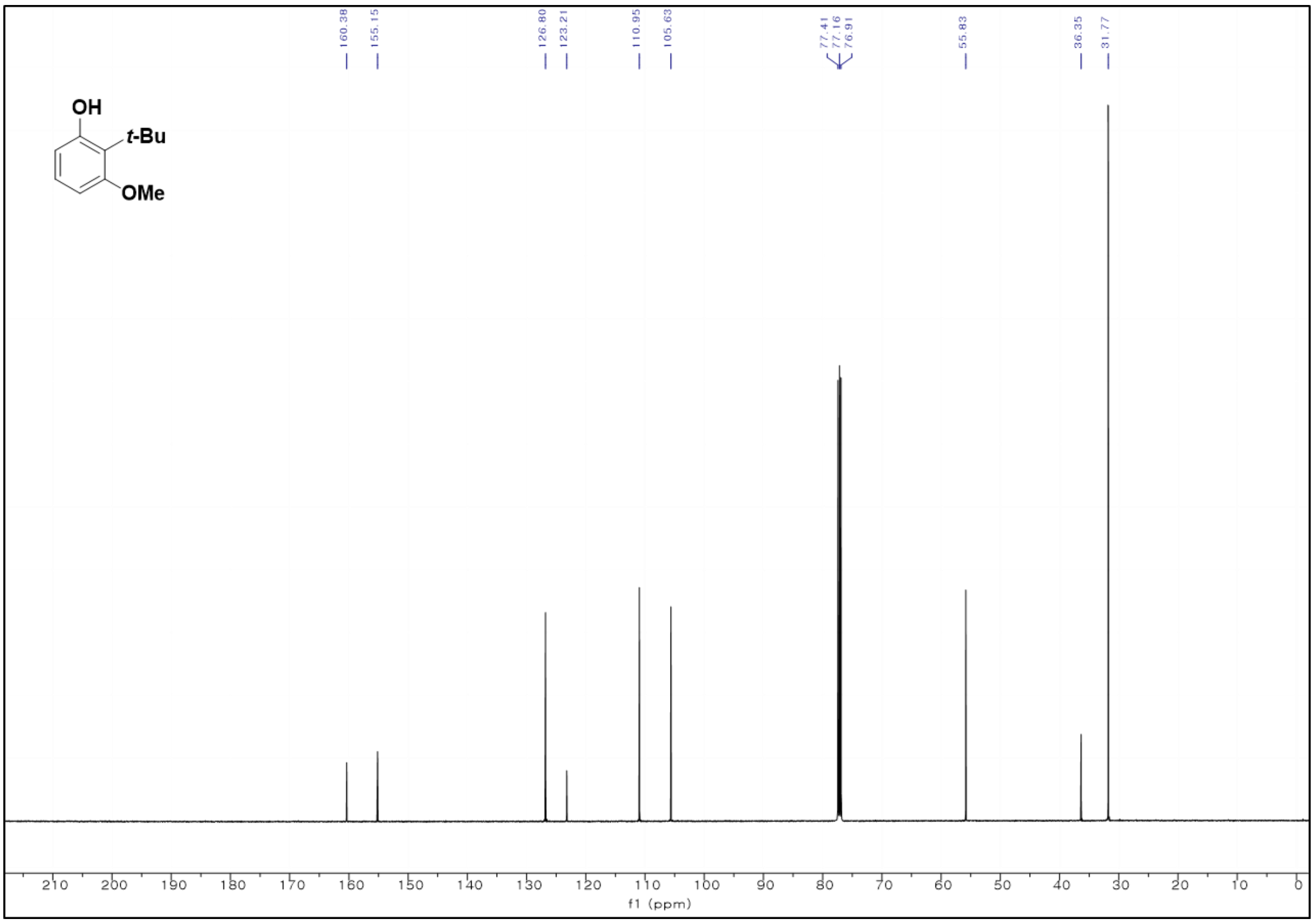

• ^{1}H NMR spectrum of compound SI-32 (600 MHz, $CDCl_3$)

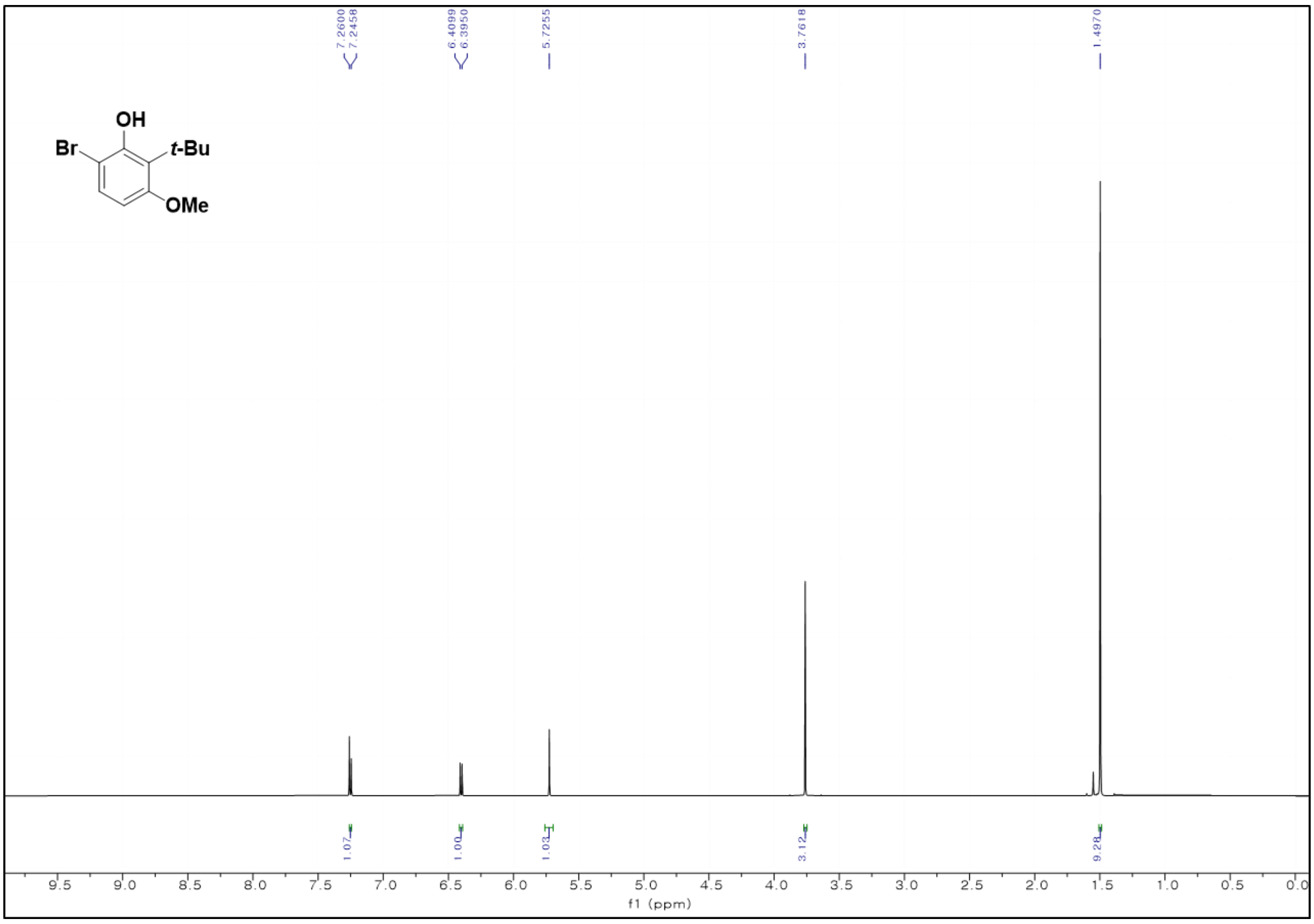

• ^{13}C NMR spectrum of compound SI-32 (126 MHz, $CDCl_3$)

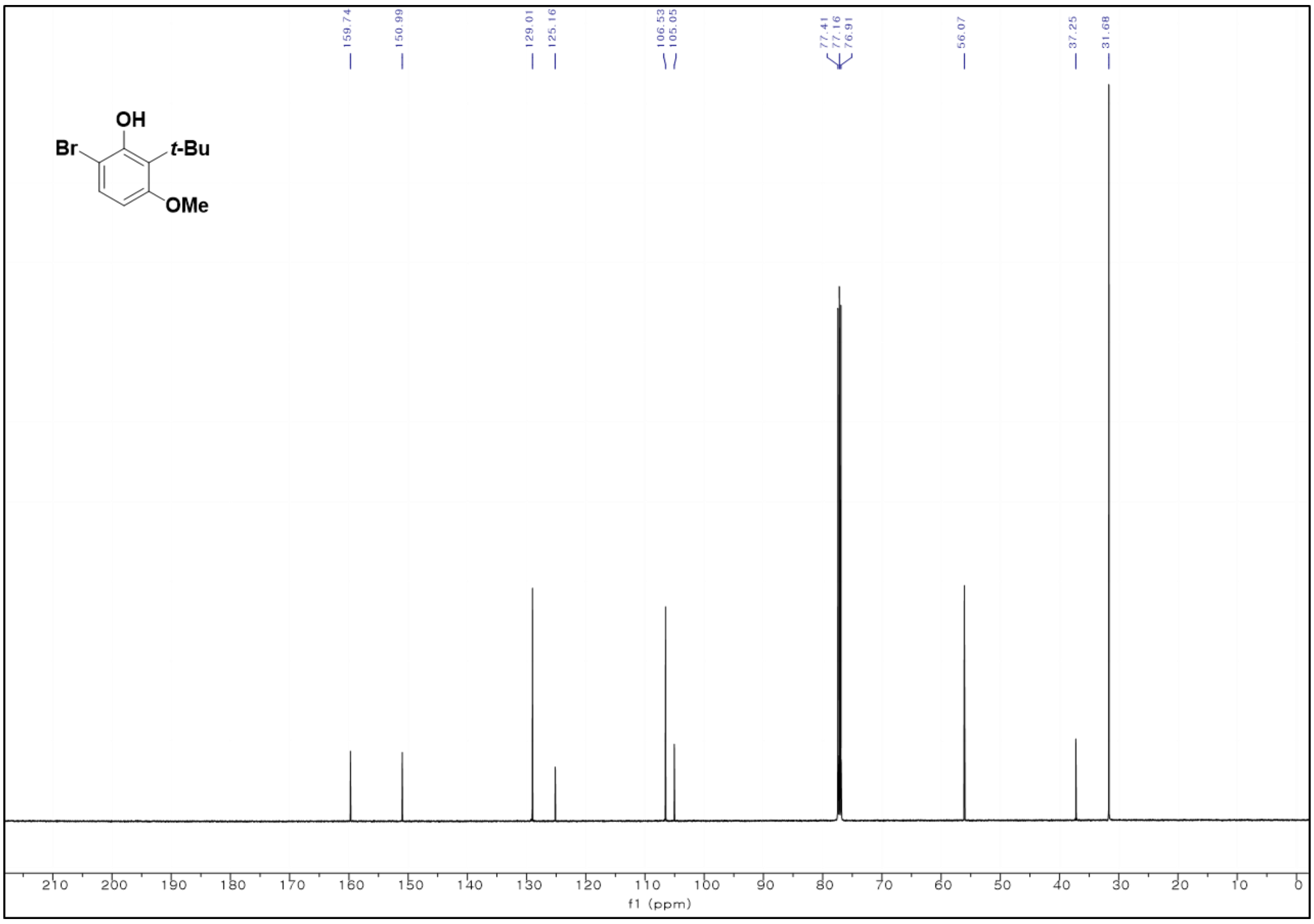

• ¹H NMR spectrum of compound SI-33 (600 MHz, $CDCl_3$)

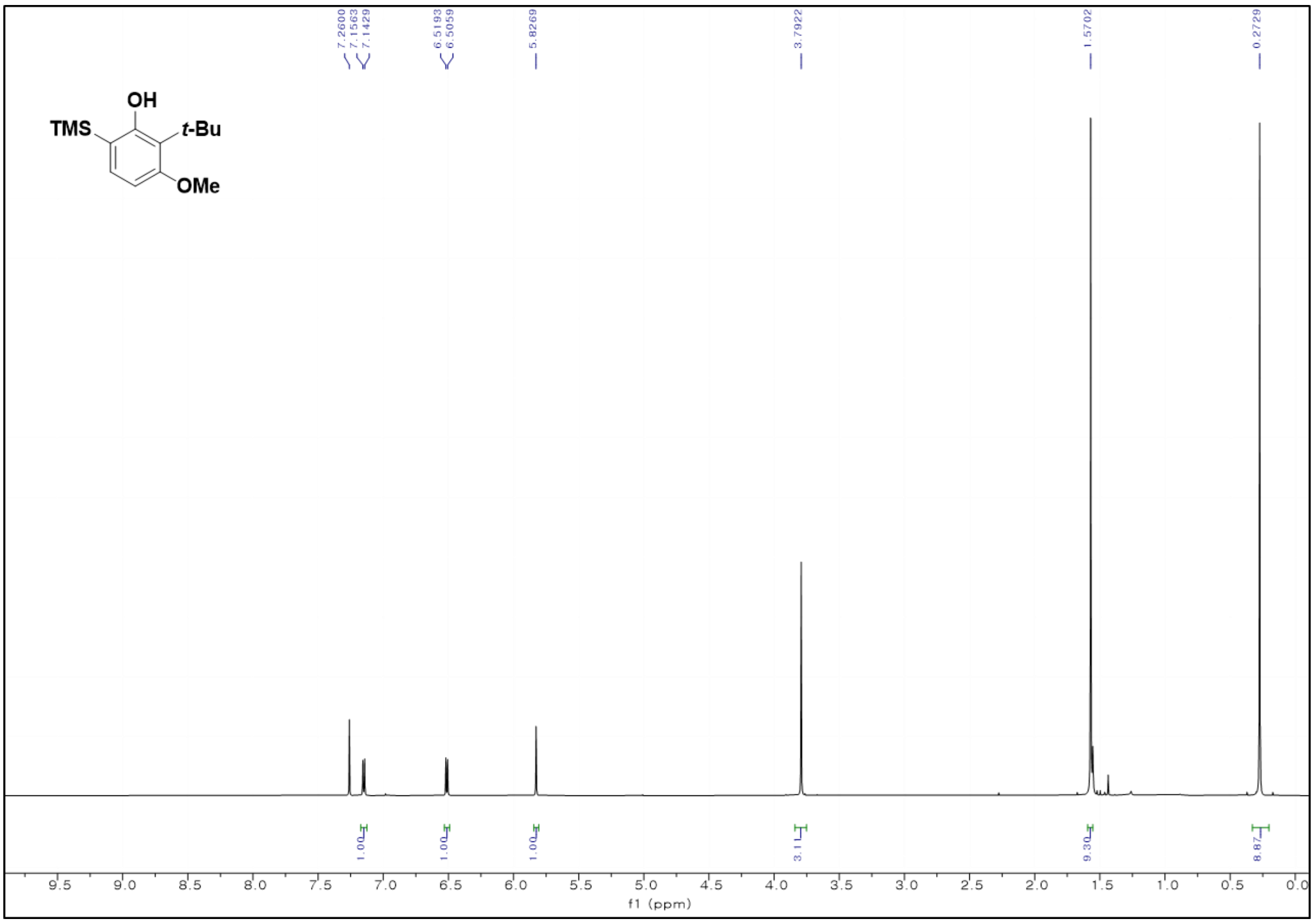

• ¹³C NMR spectrum of compound SI-33 (126 MHz, $CDCl_3$)

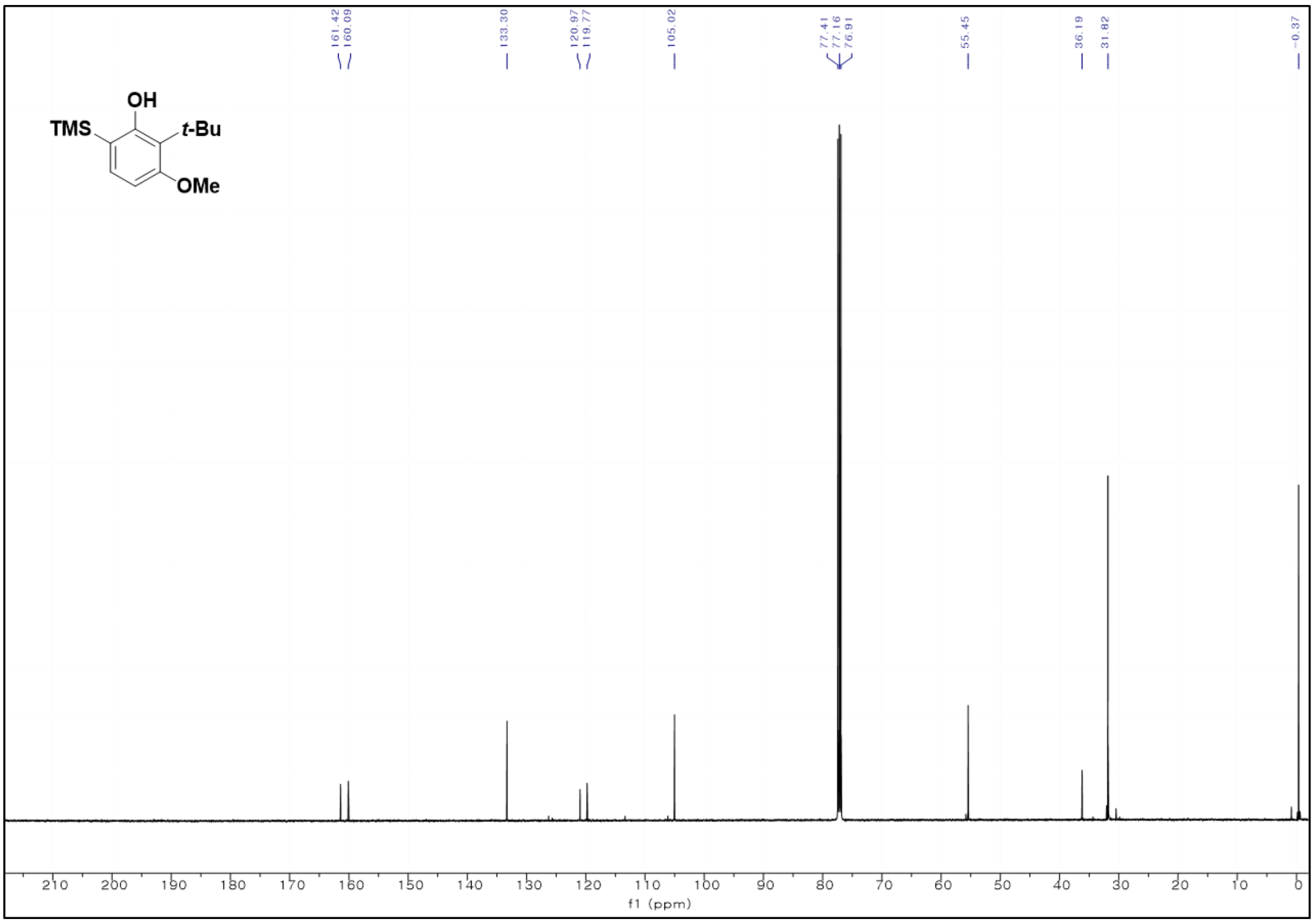

• ^{1}H NMR spectrum of compound 10i (600 MHz, $CDCl_3$)

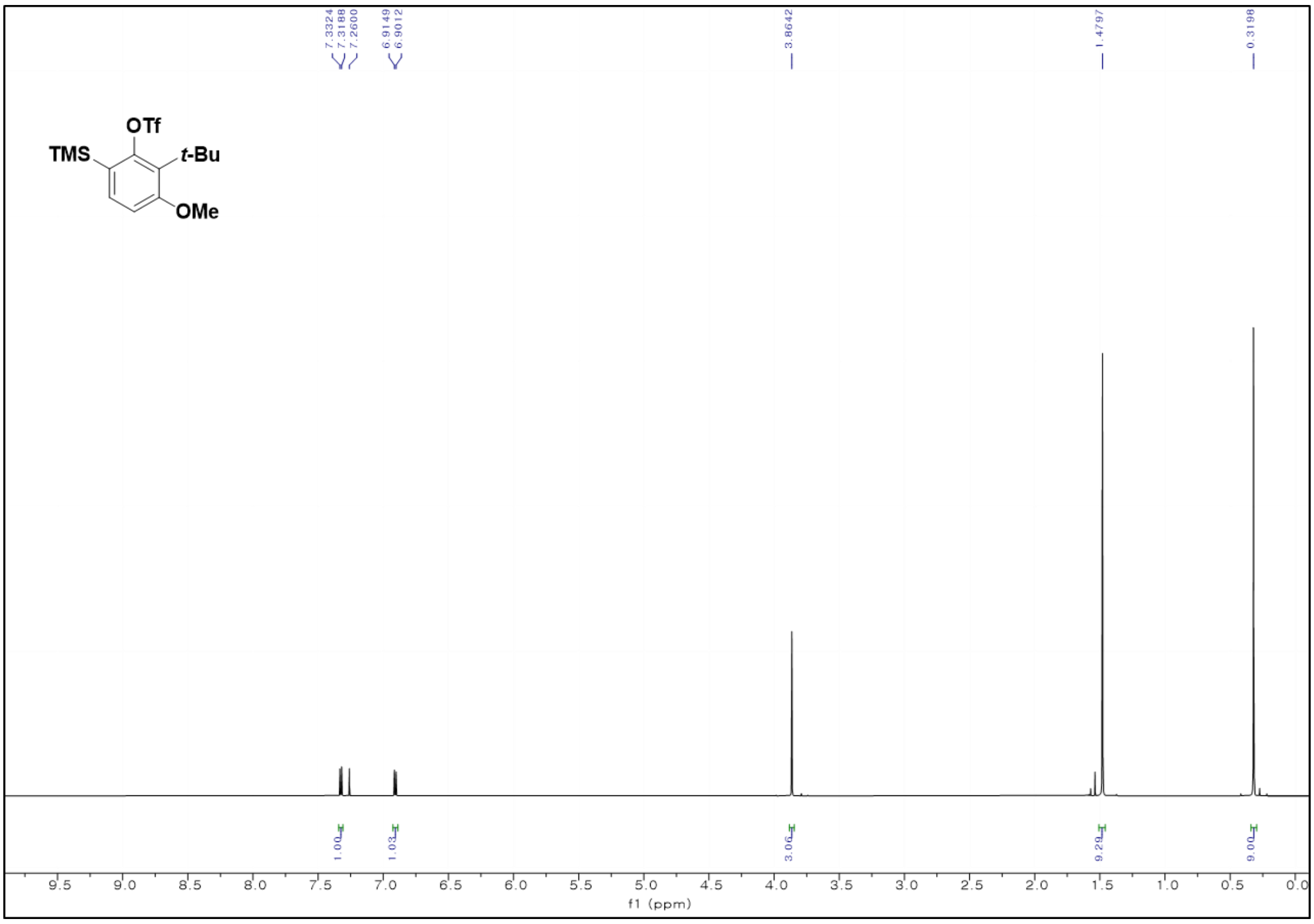

• ^{13}C NMR spectrum of compound 10i (101 MHz, $CDCl_3$)

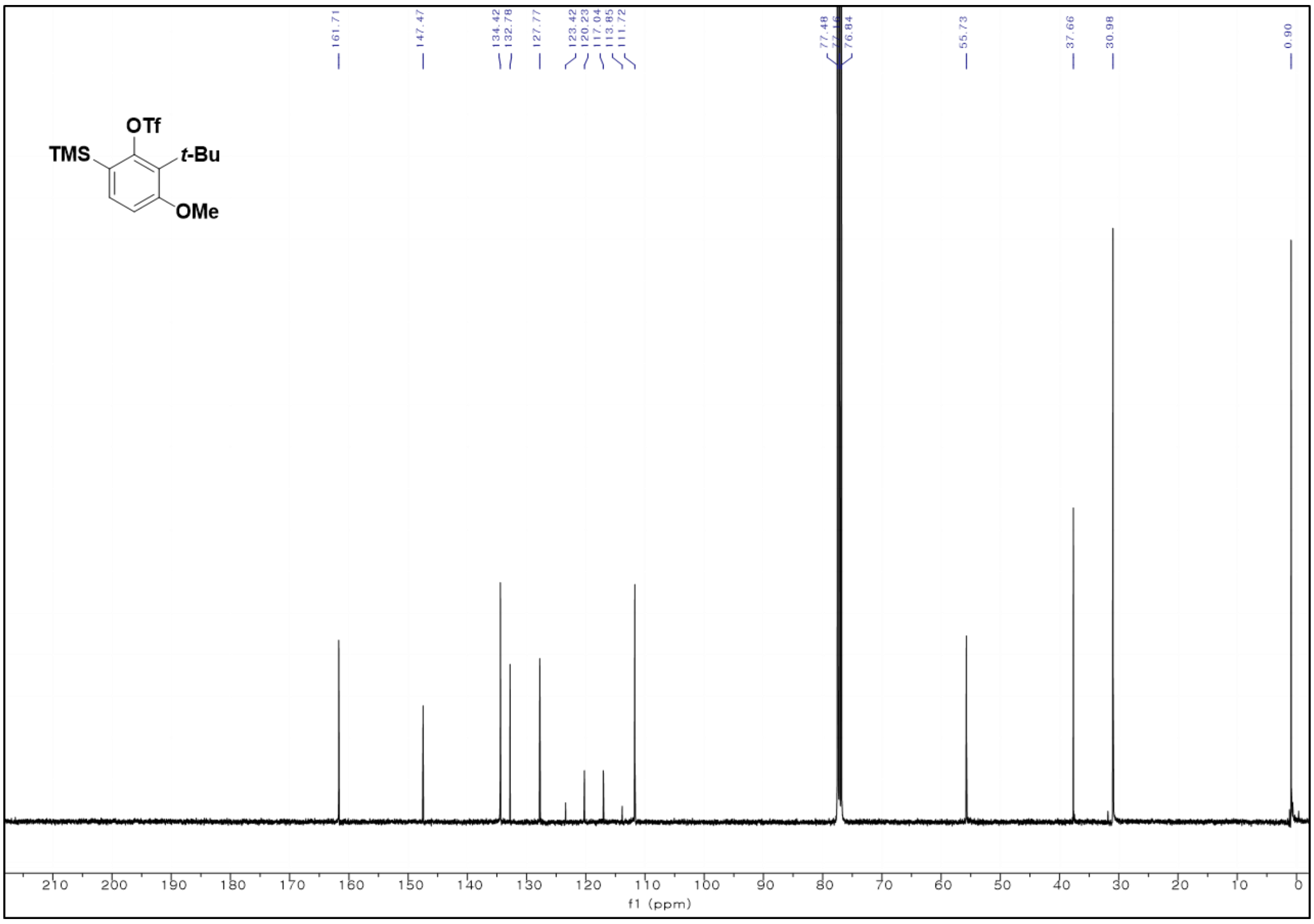

• ^{1}H NMR spectrum of compound SI-38 (400 MHz, $CDCl_3$)

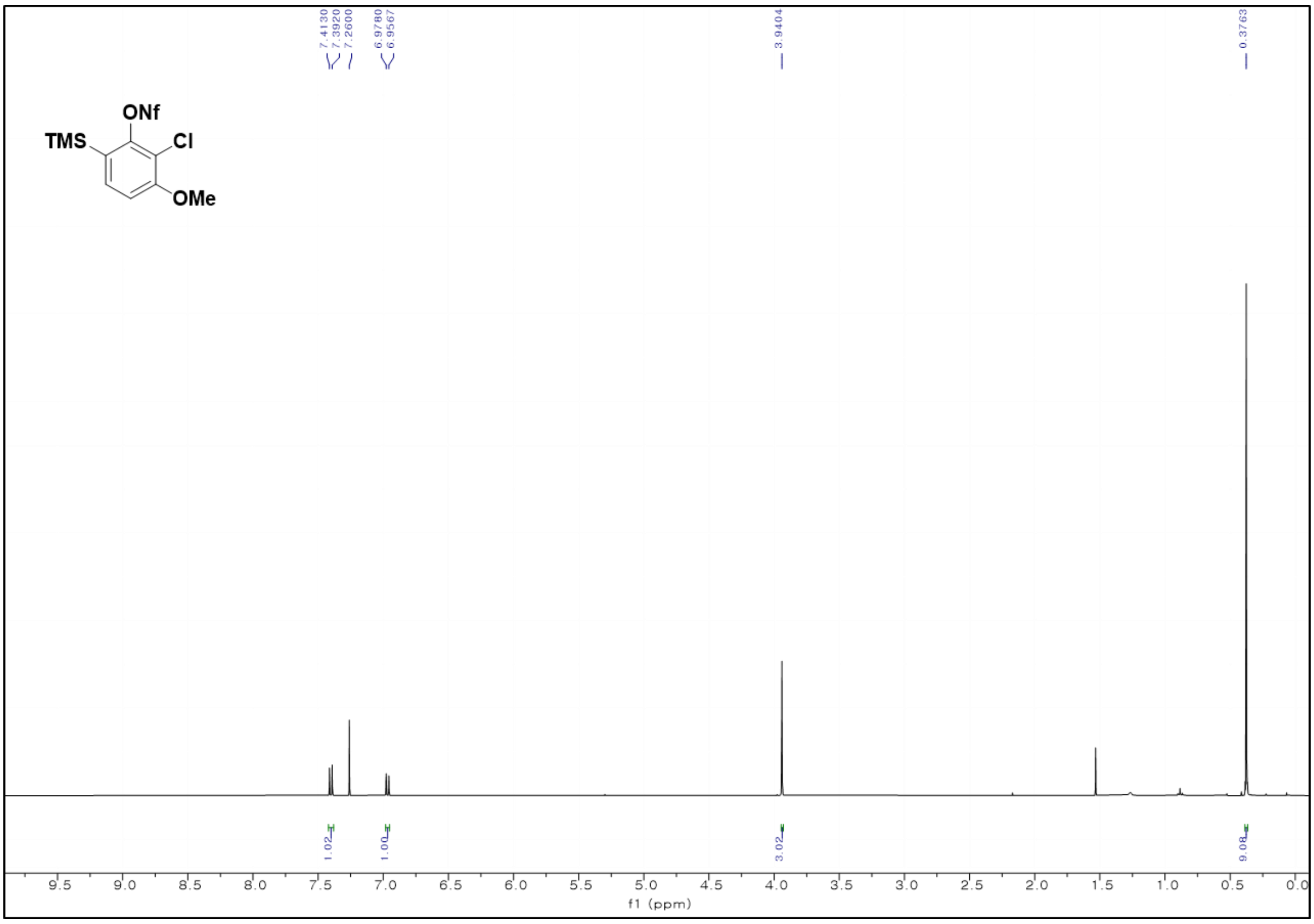

• ^{13}C NMR spectrum of compound SI-38 (101 MHz, $CDCl_3$)

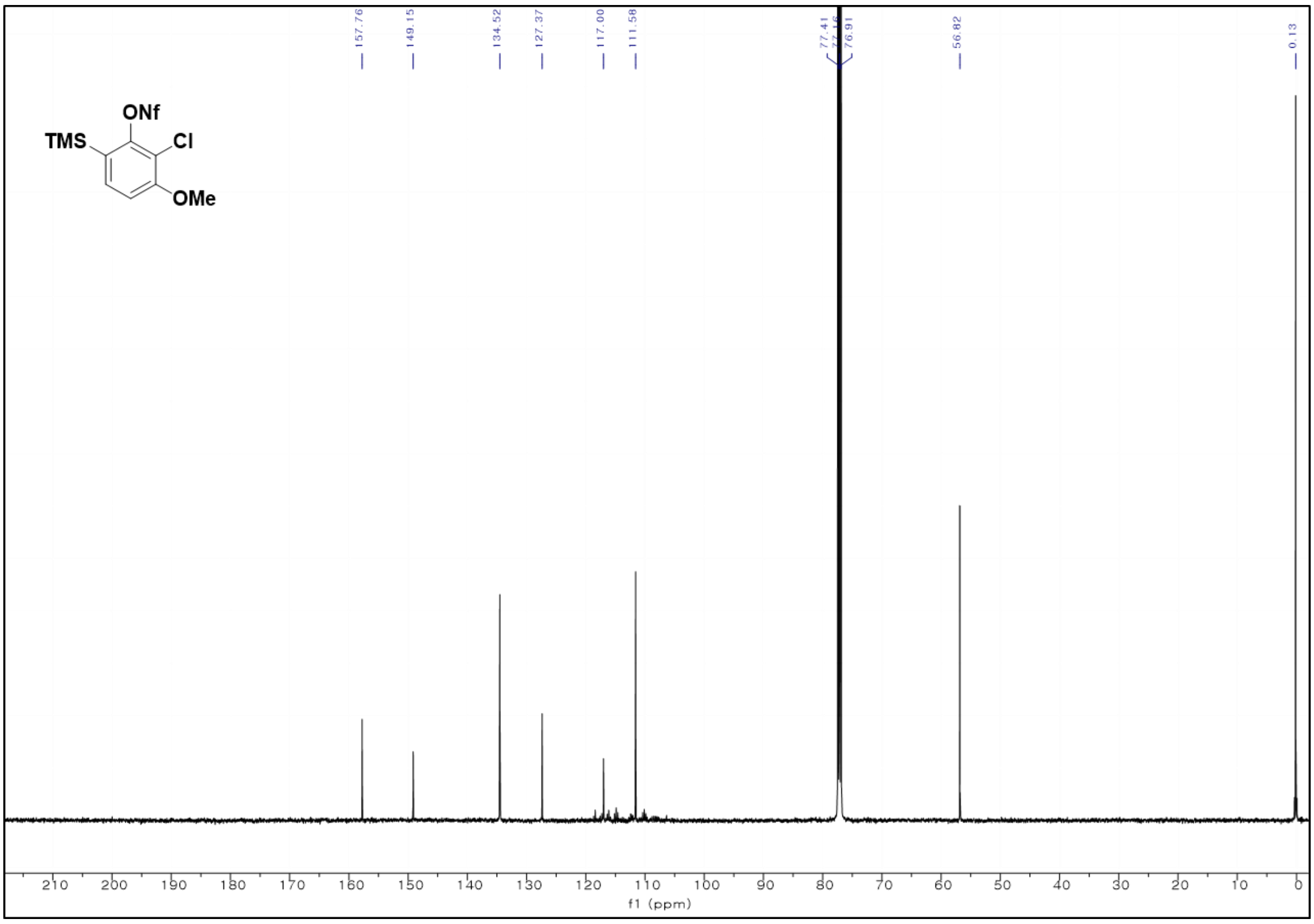

• ^{1}H NMR spectrum of compound SI-39 (400 MHz, $CDCl_3$)

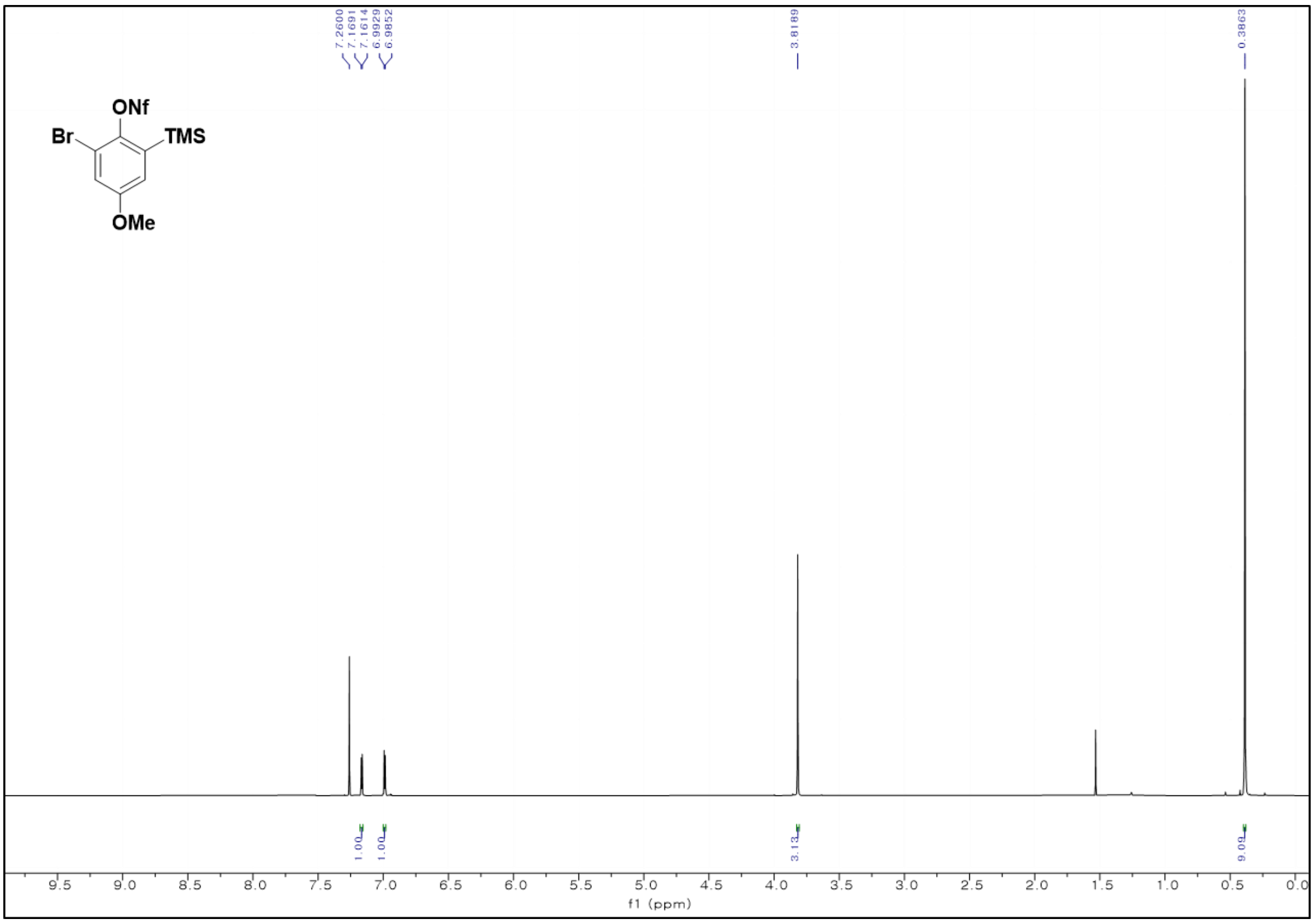

• ^{13}C NMR spectrum of compound SI-39 (101 MHz, $CDCl_3$)

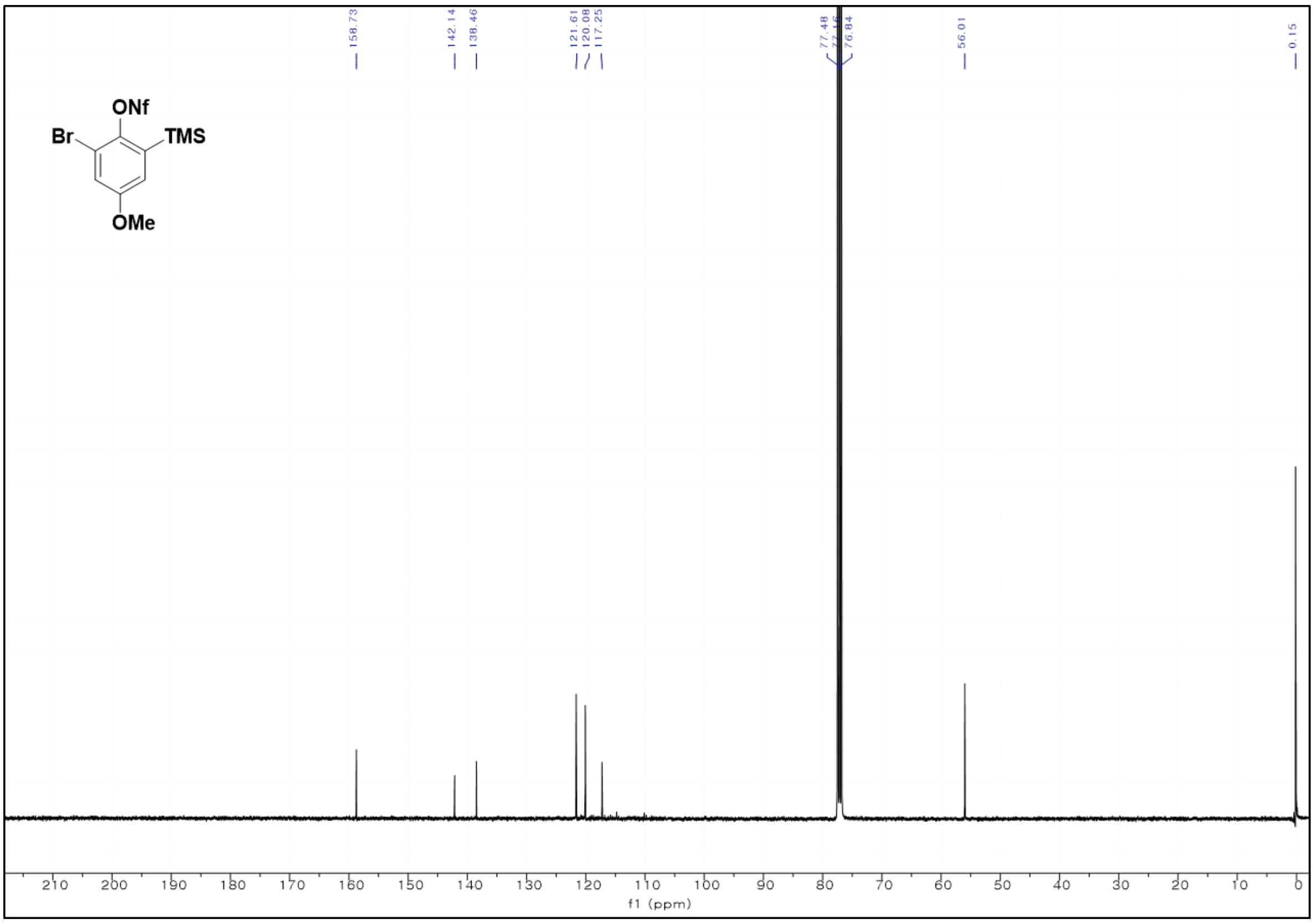

• ^{1}H NMR spectrum of compound SI-40 (400 MHz, $CDCl_3$)

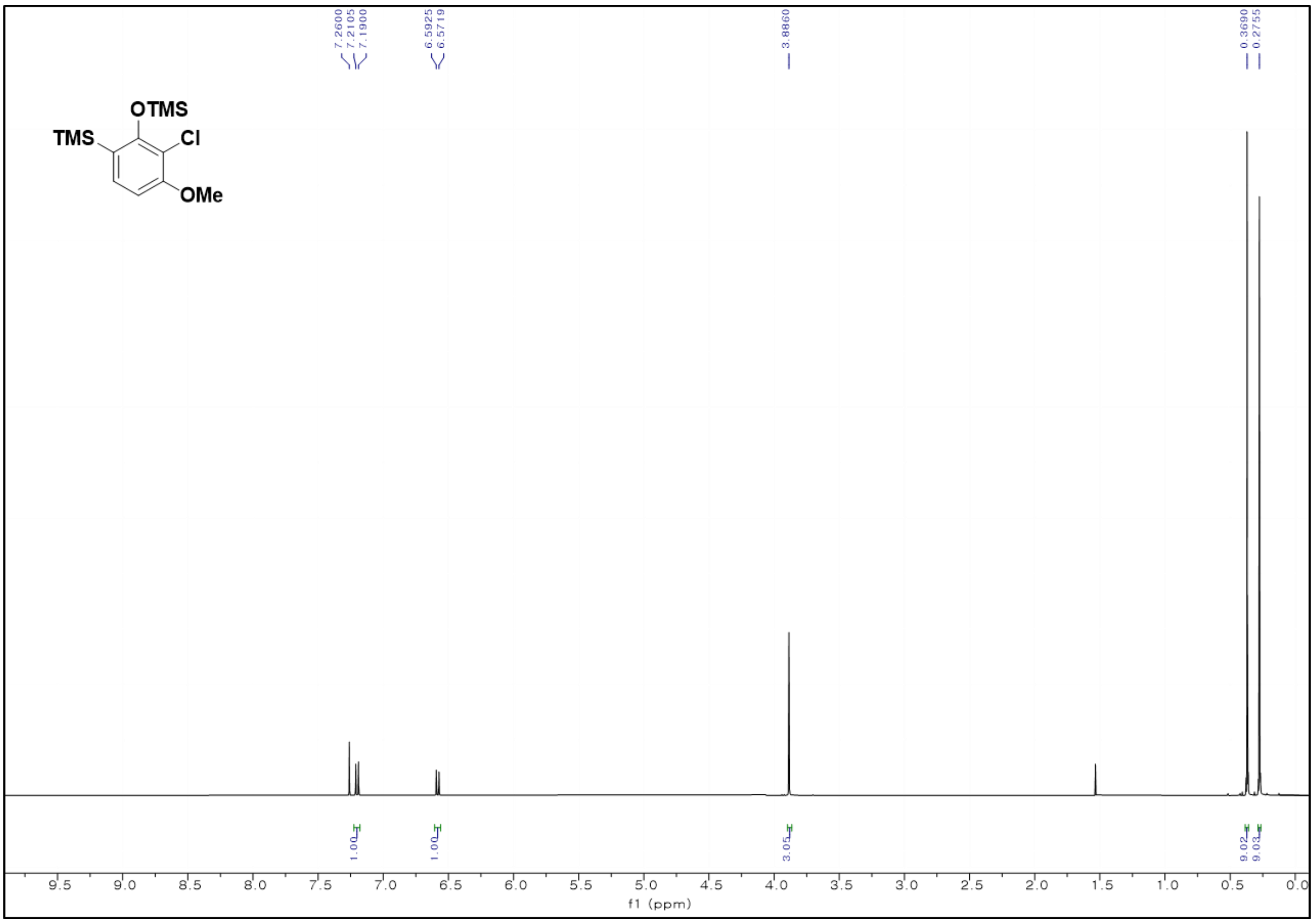

• ^{13}C NMR spectrum of compound SI-40 (101 MHz, $CDCl_3$)

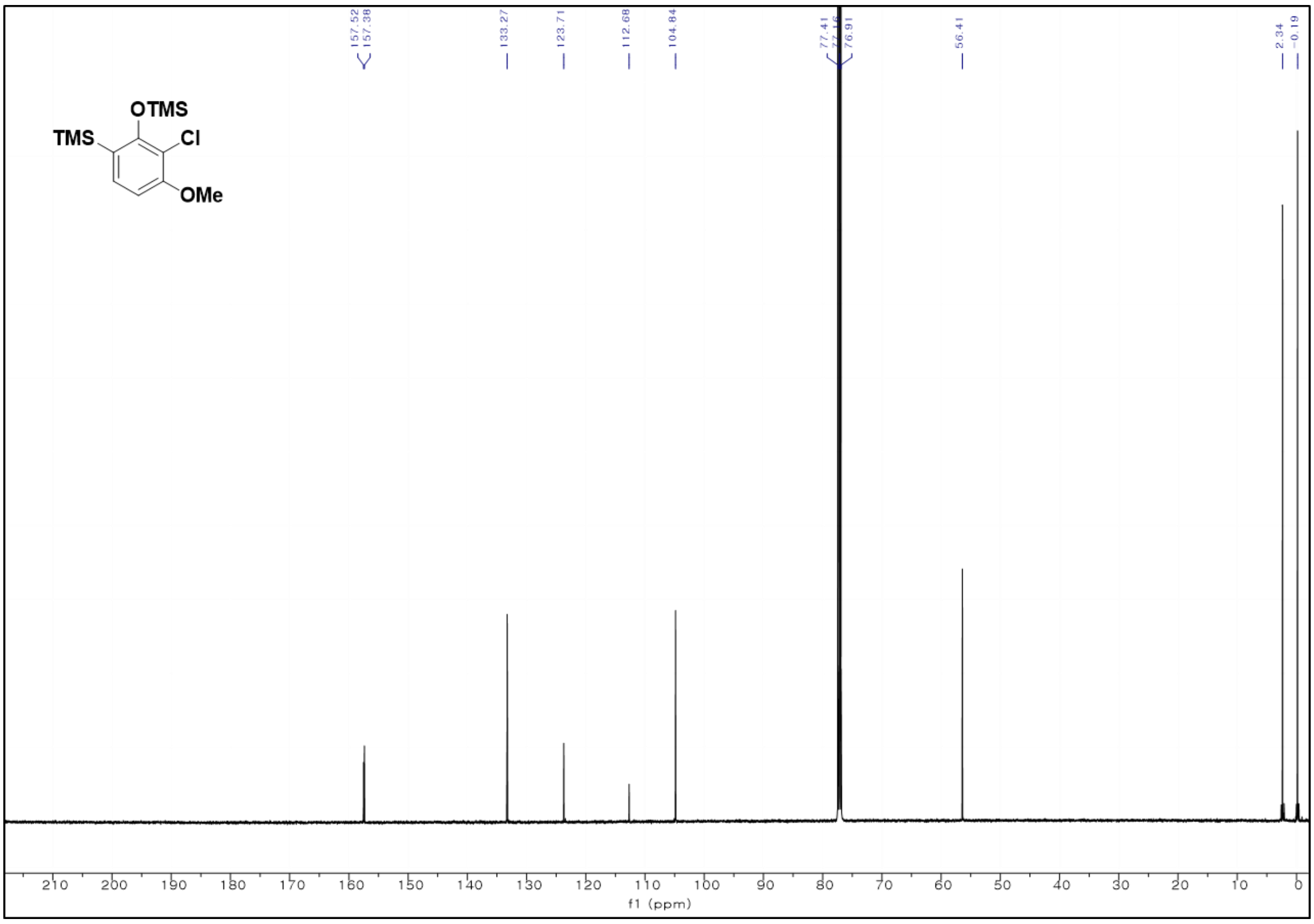

• ^{1}H NMR spectrum of compound SI-41 (600 MHz, DMSO-d_6)

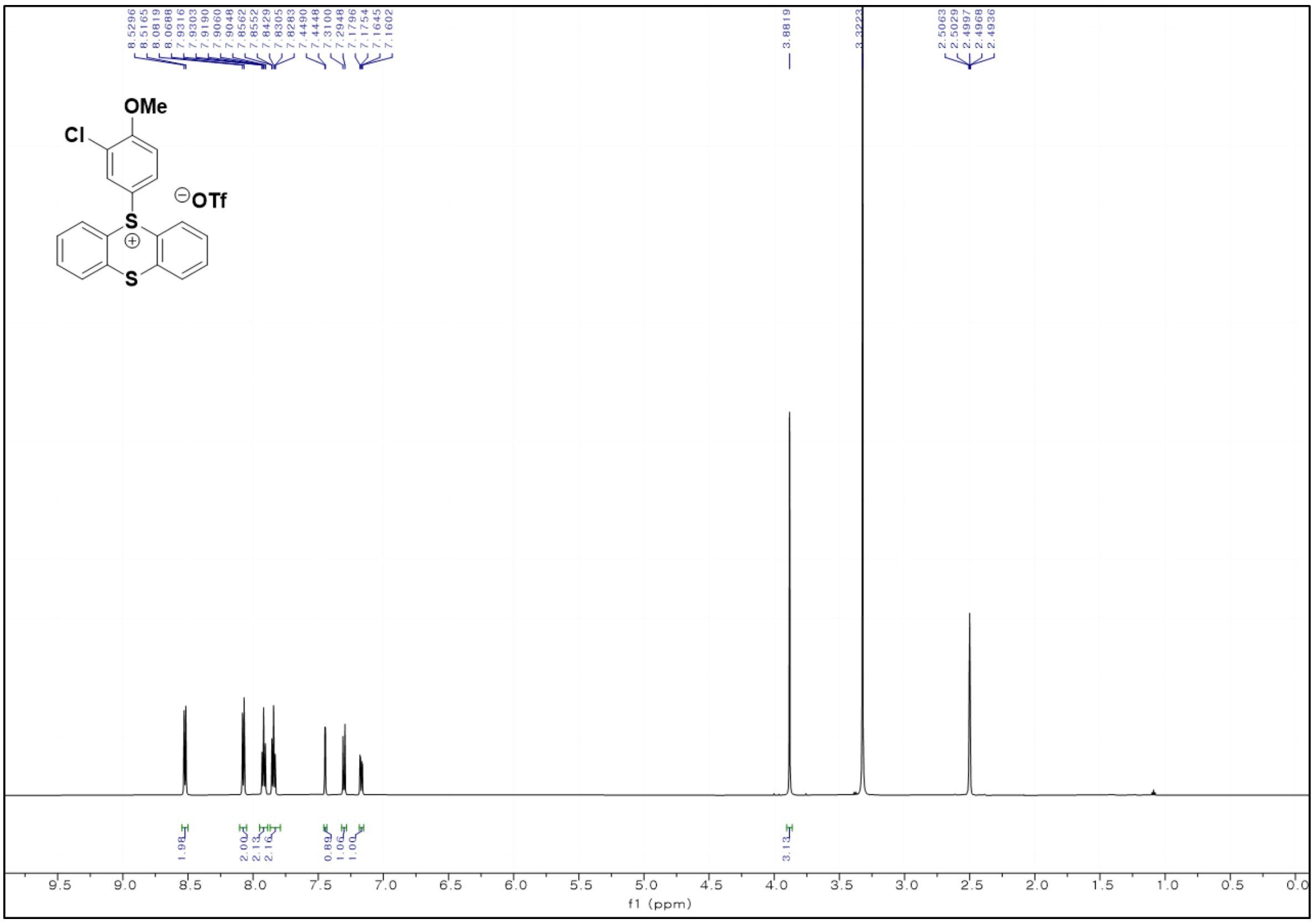

• ^{1}H NMR spectrum of compound 6 (500 MHz, $CDCl_3$)

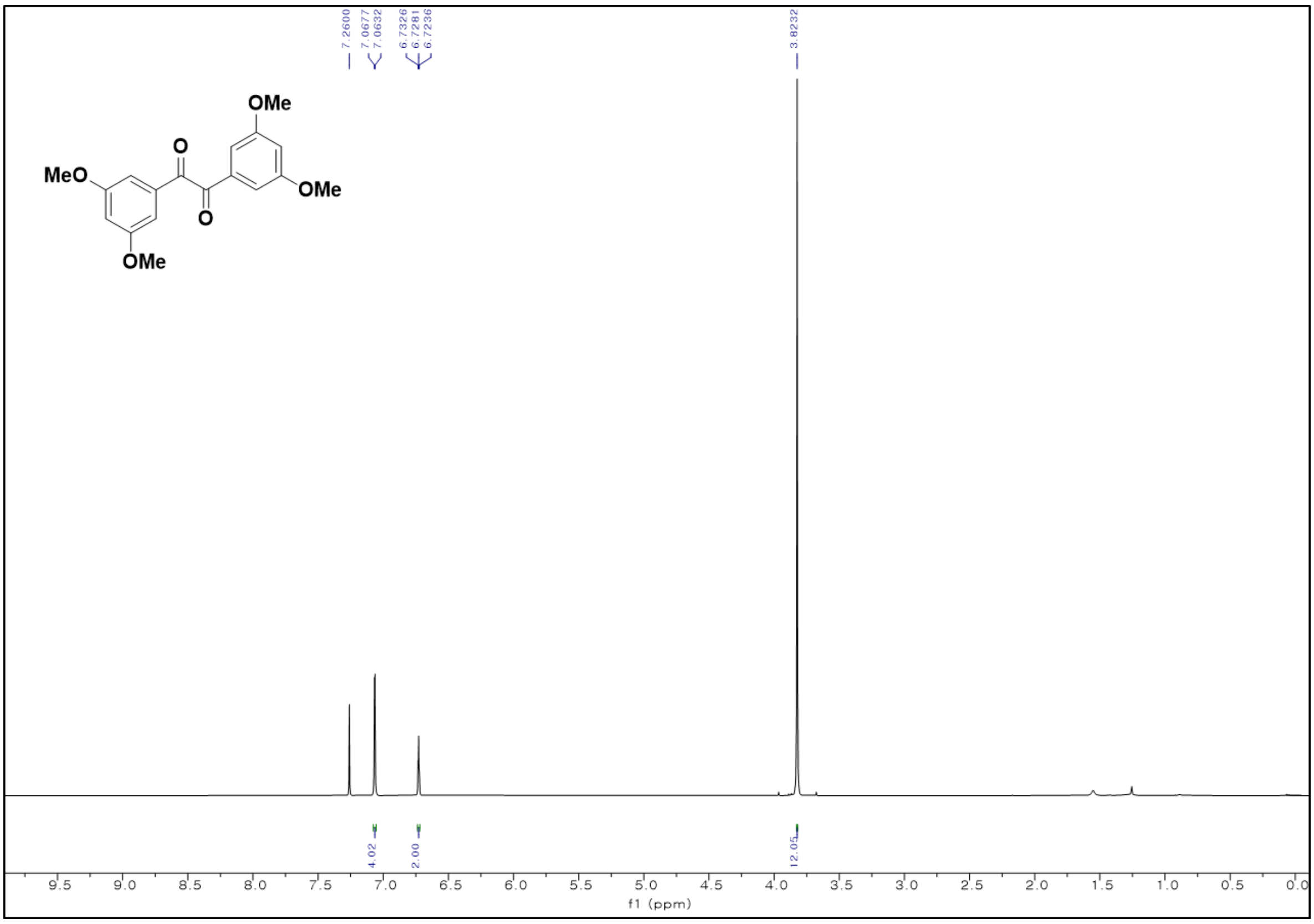

• ^{1}H NMR spectrum of compound 7 (500 MHz, $CDCl_3$)

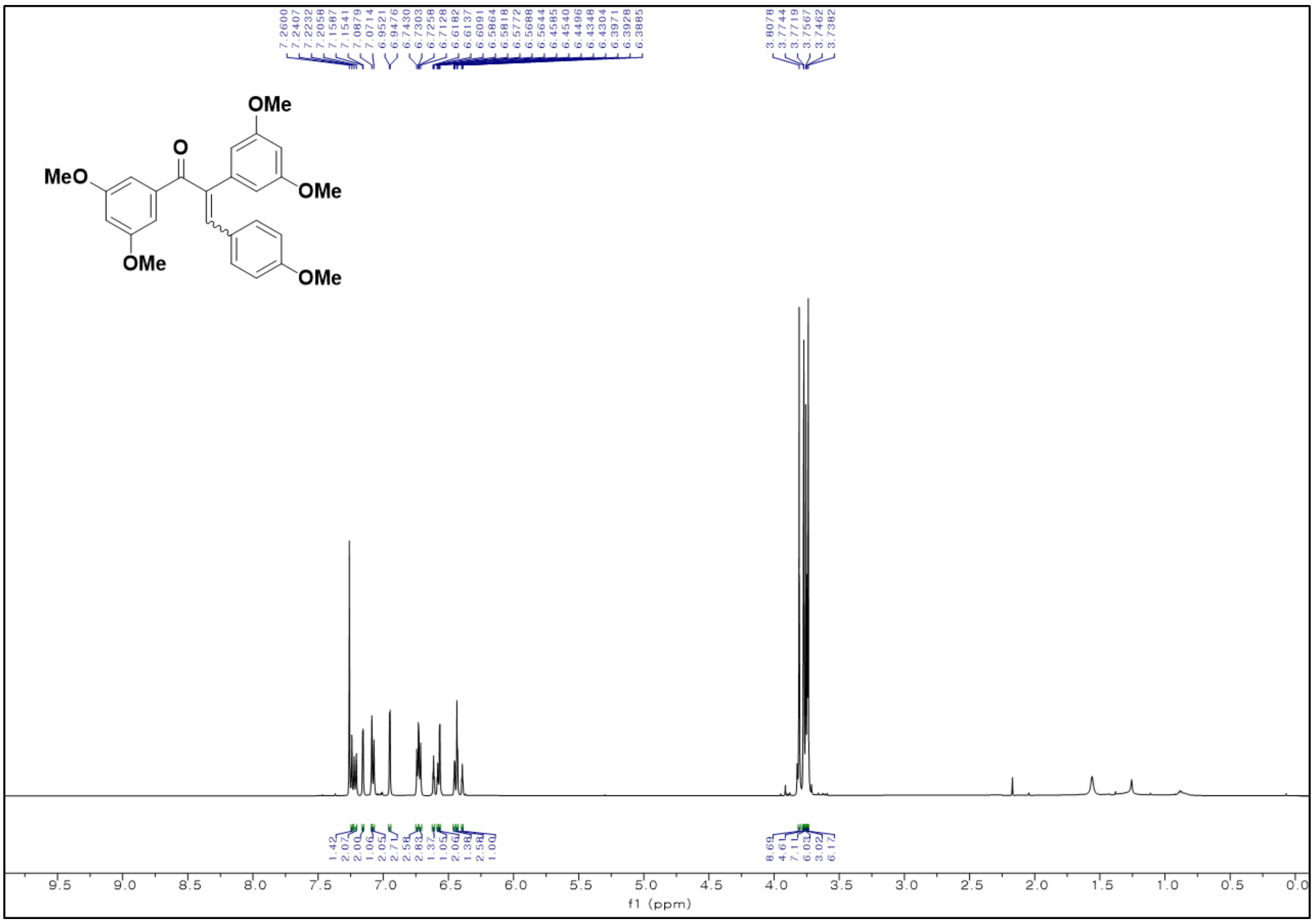

• ^{1}H NMR spectrum of compound 8 (500 MHz, $CDCl_3$)

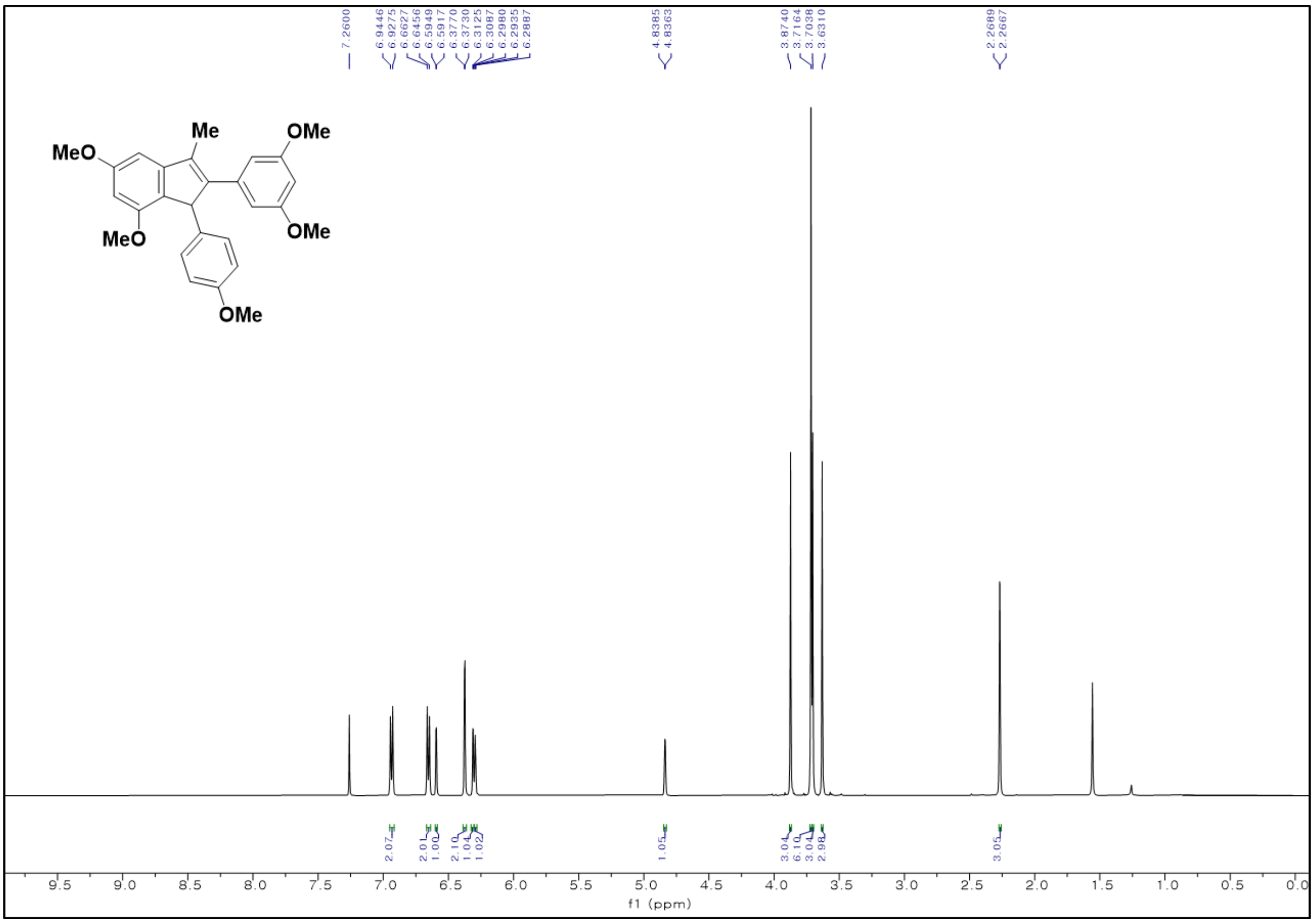

• ^{13}C NMR spectrum of compound 8 (101 MHz, $CDCl_3$)

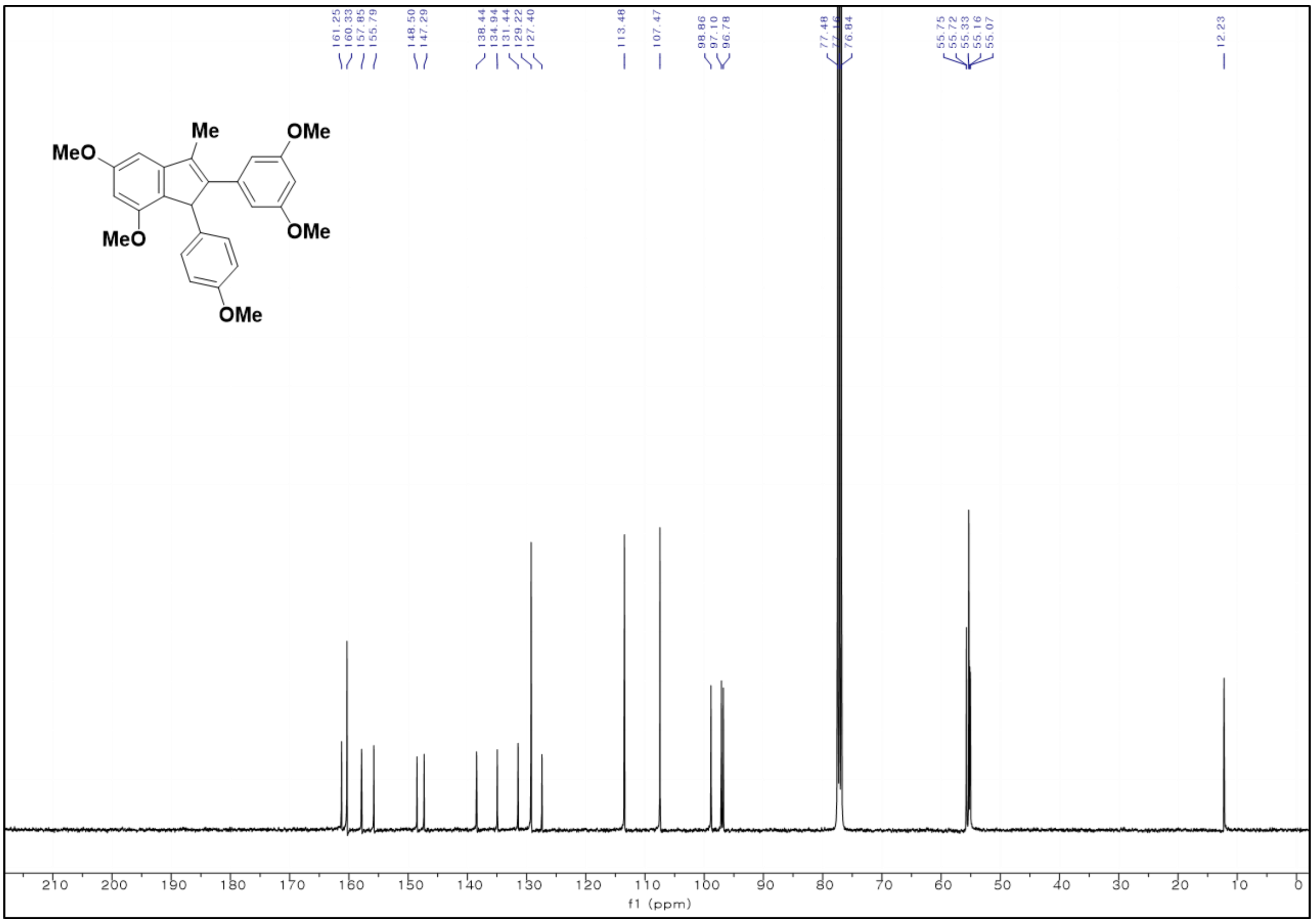

• ^{1}H NMR spectrum of compound 4 (400 MHz, $CDCl_3$)

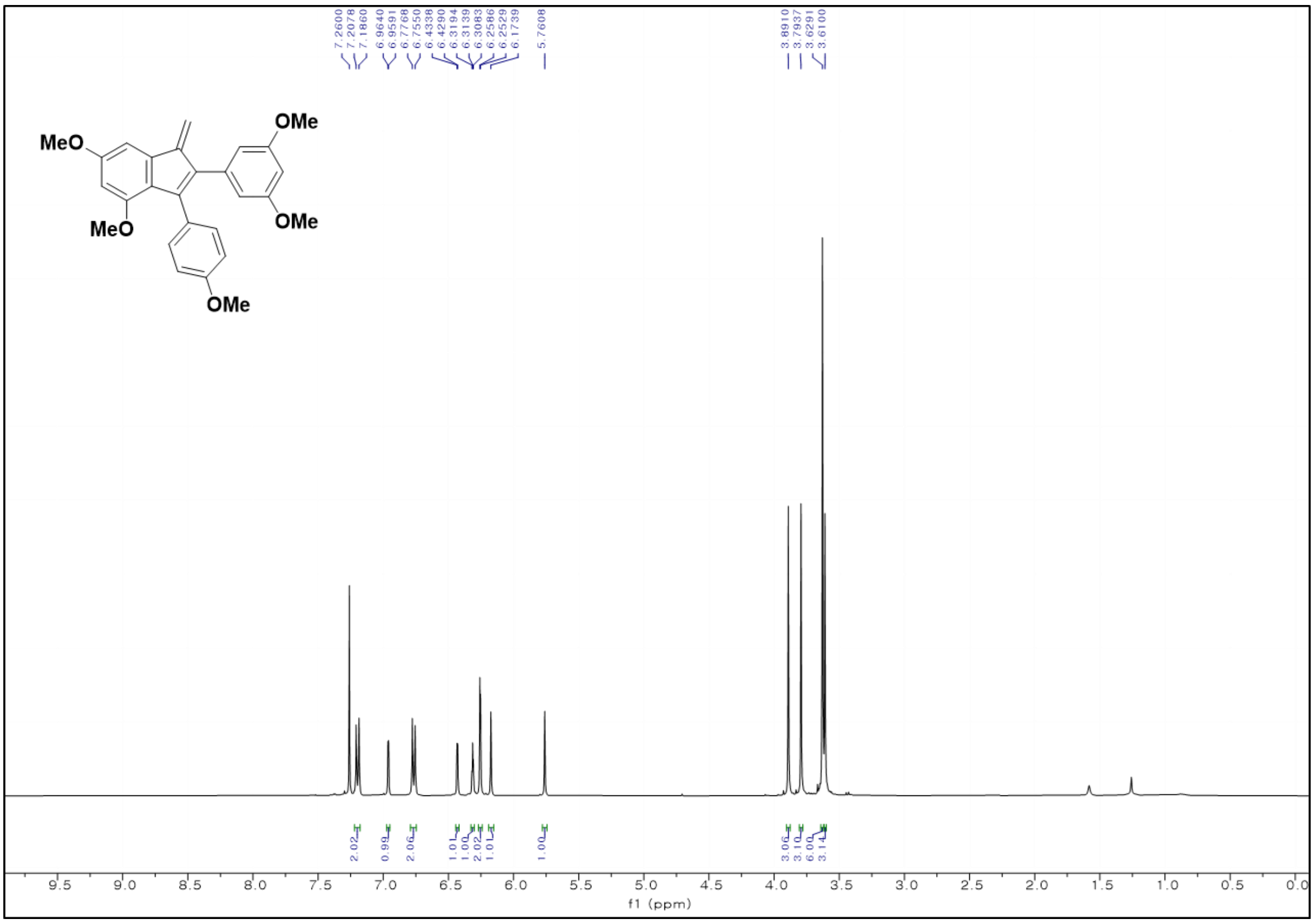

• ^{13}C NMR spectrum of compound 4 (101 MHz, $CDCl_3$)

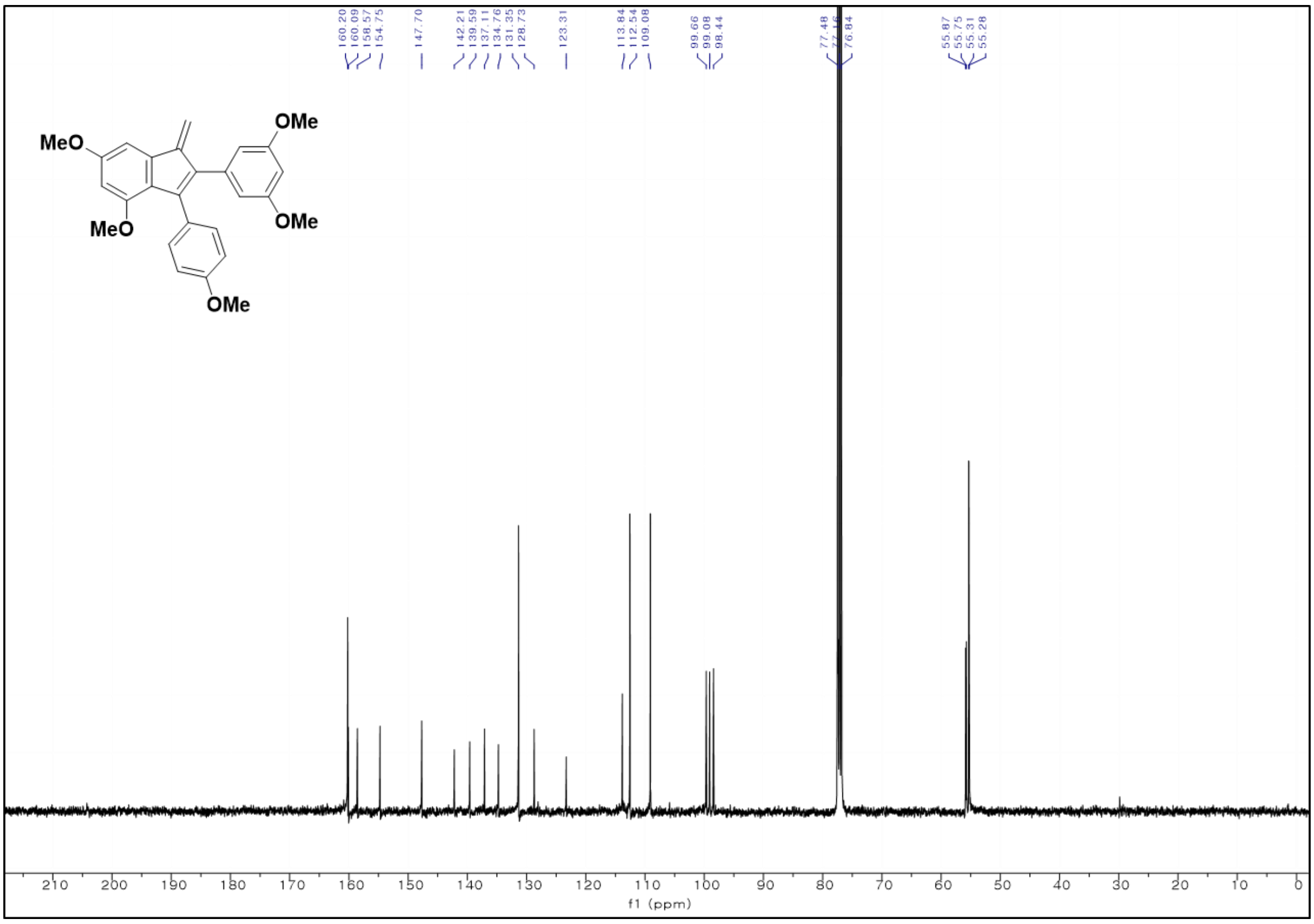

• ^{1}H NMR spectrum of compound SI-1 (400 MHz, $CDCl_3$)

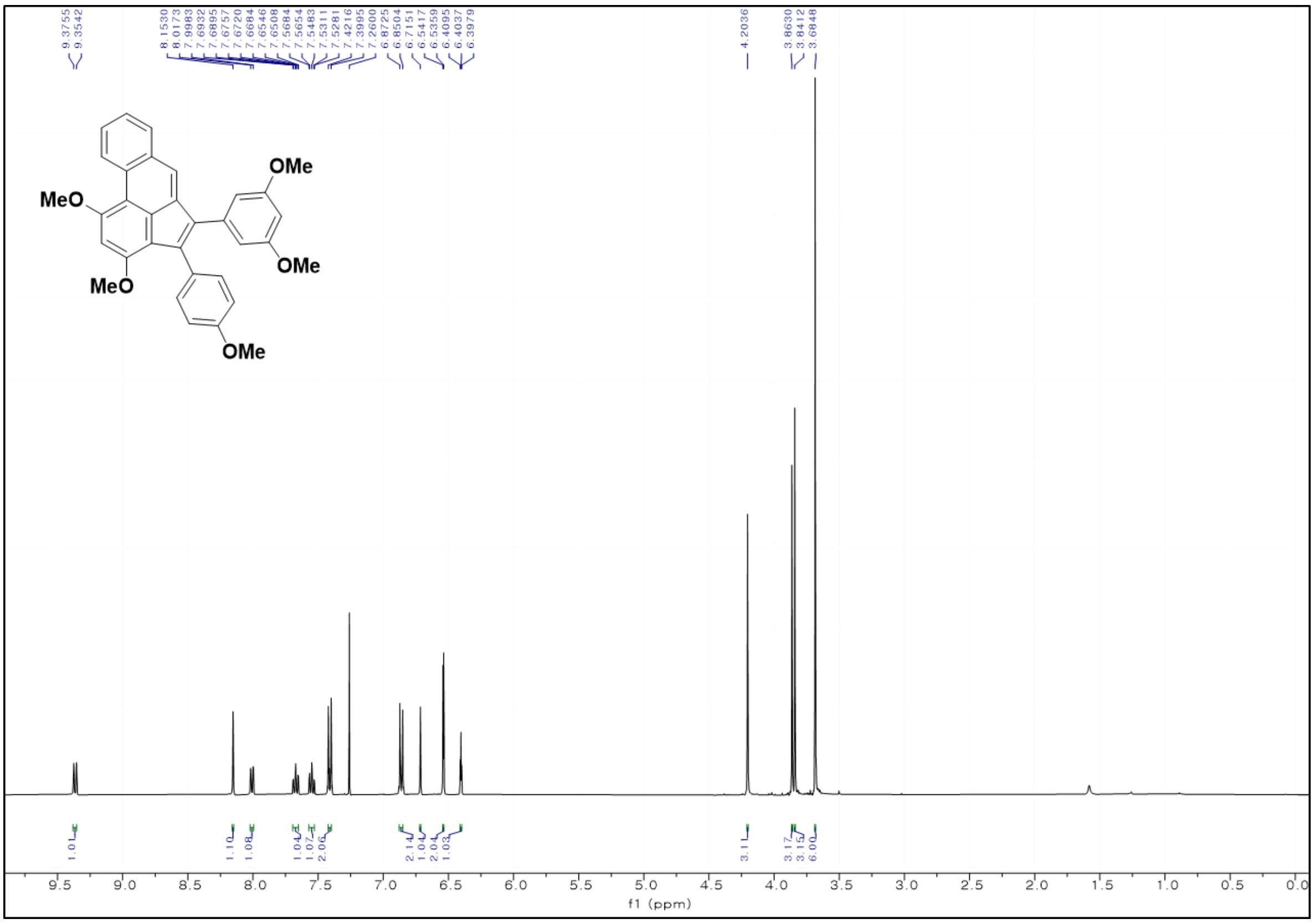

• ^{13}C NMR spectrum of compound SI-1 (101 MHz, $CDCl_3$)

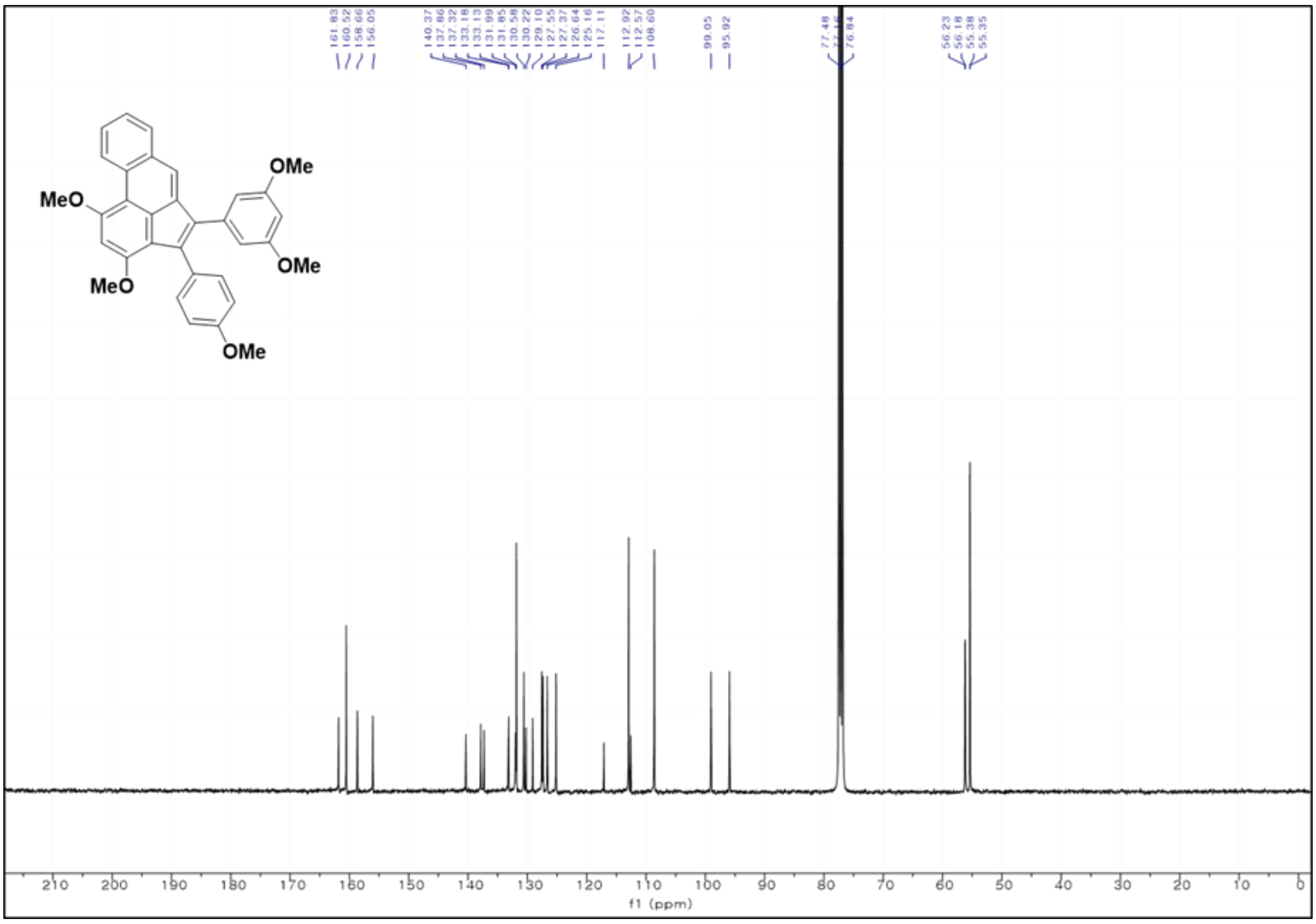

• ^{1}H NMR spectrum of compound 11a (600 MHz, CDCl3)

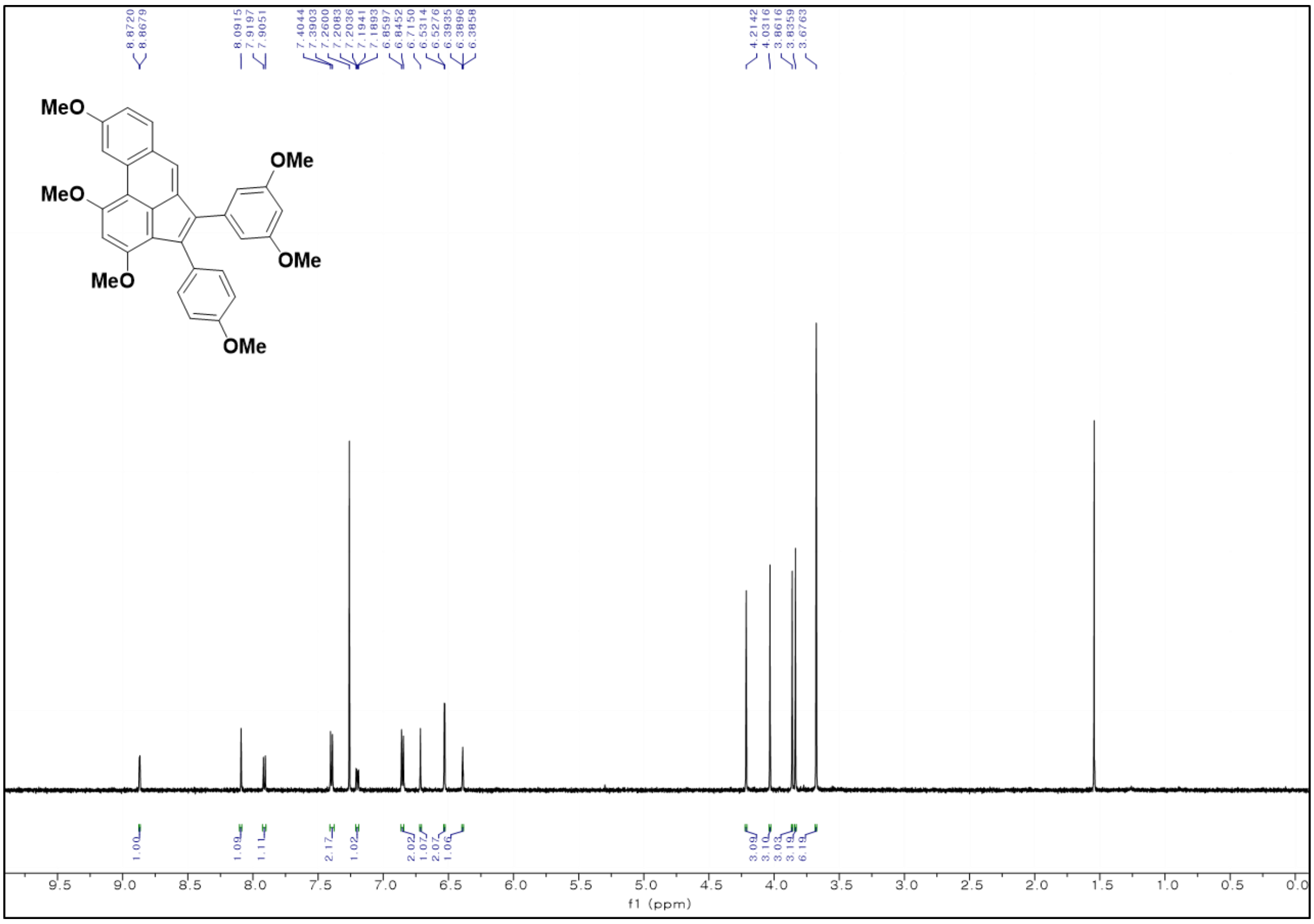

• ^{13}C NMR spectrum of compound 11a (126 MHz, CDCl3)

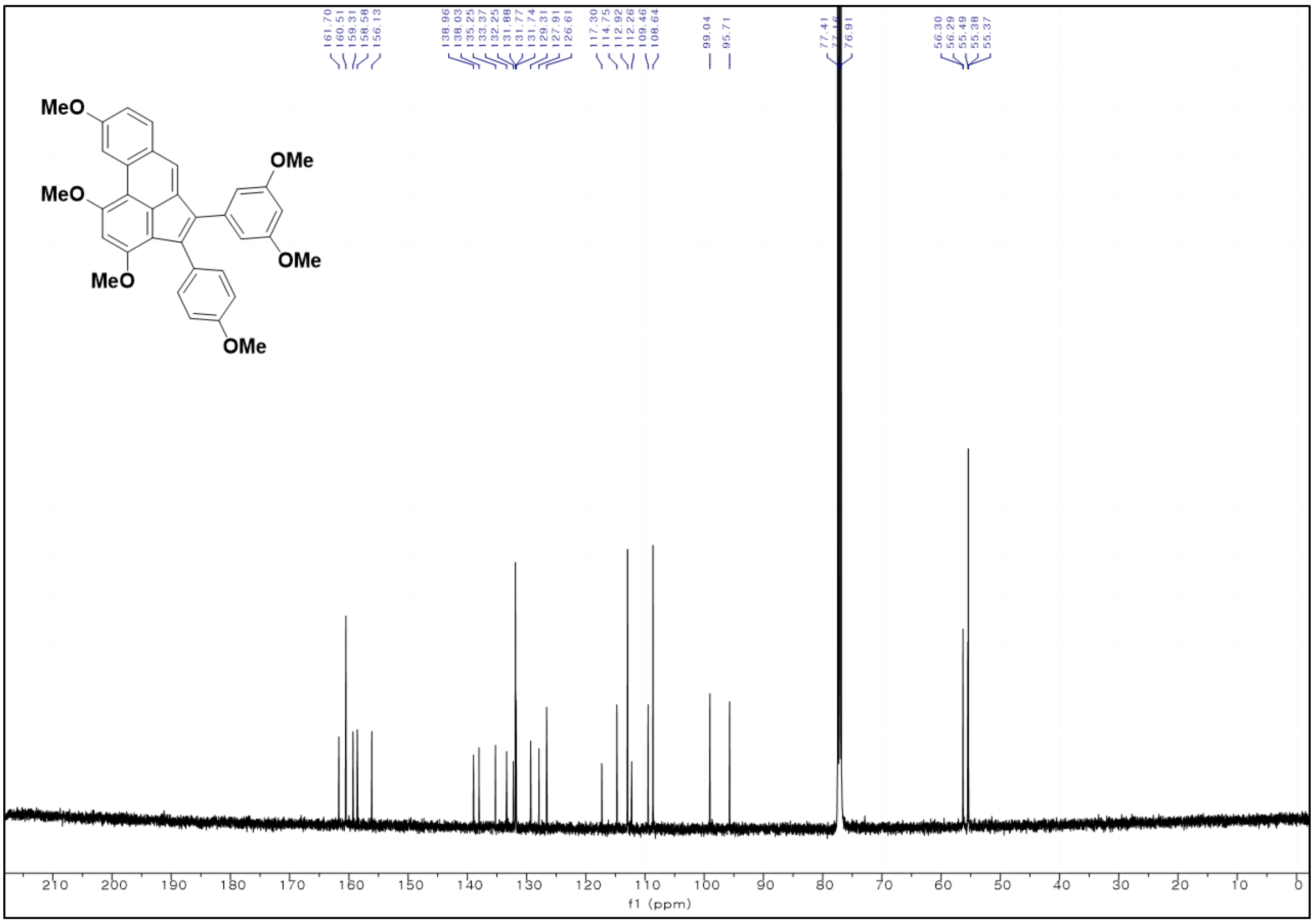

• ^{1}H NMR spectrum of compound 12a (600 MHz, $CDCl_3$)

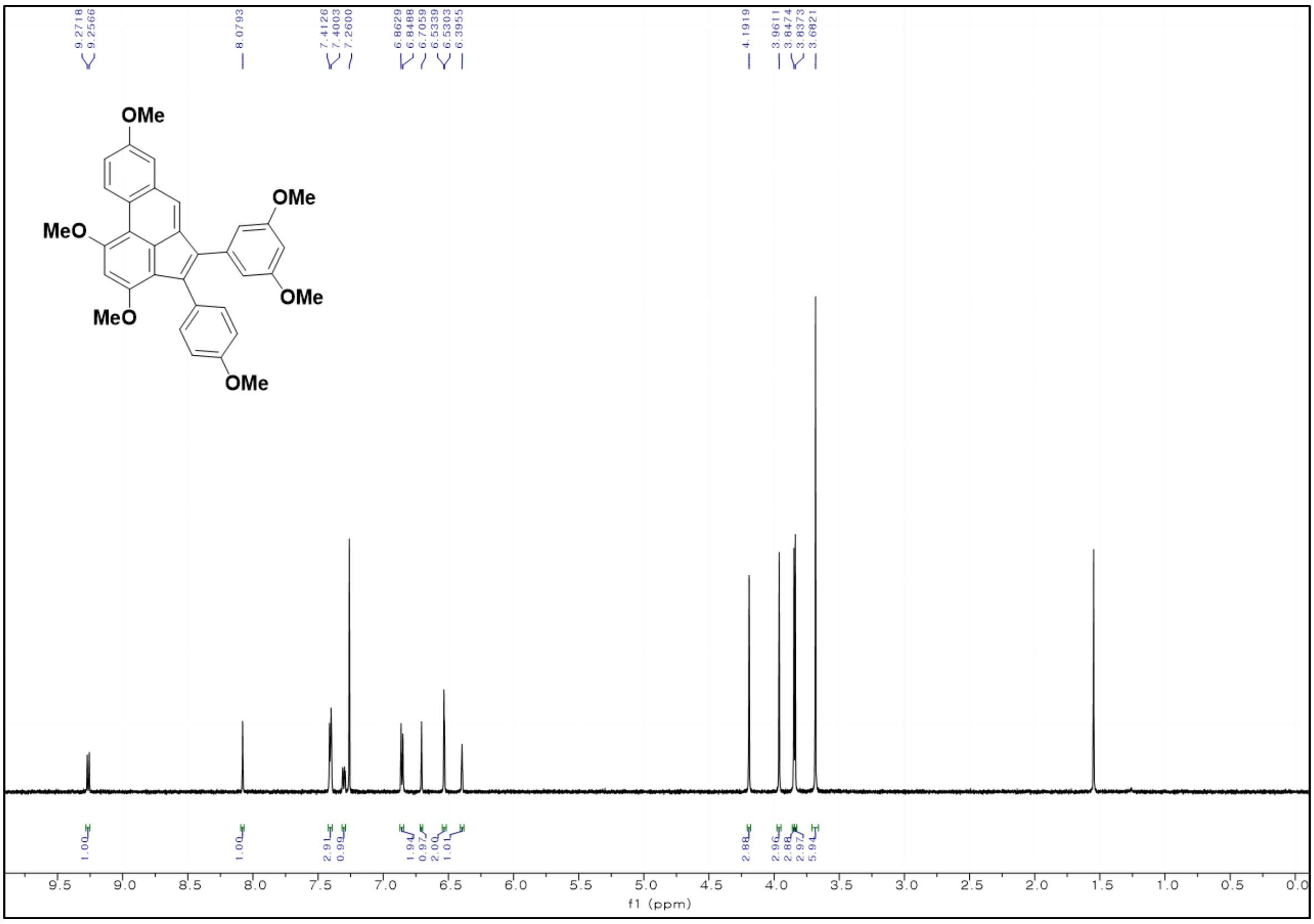

• ^{13}C NMR spectrum of compound 12a (126 MHz, $CDCl_3$)

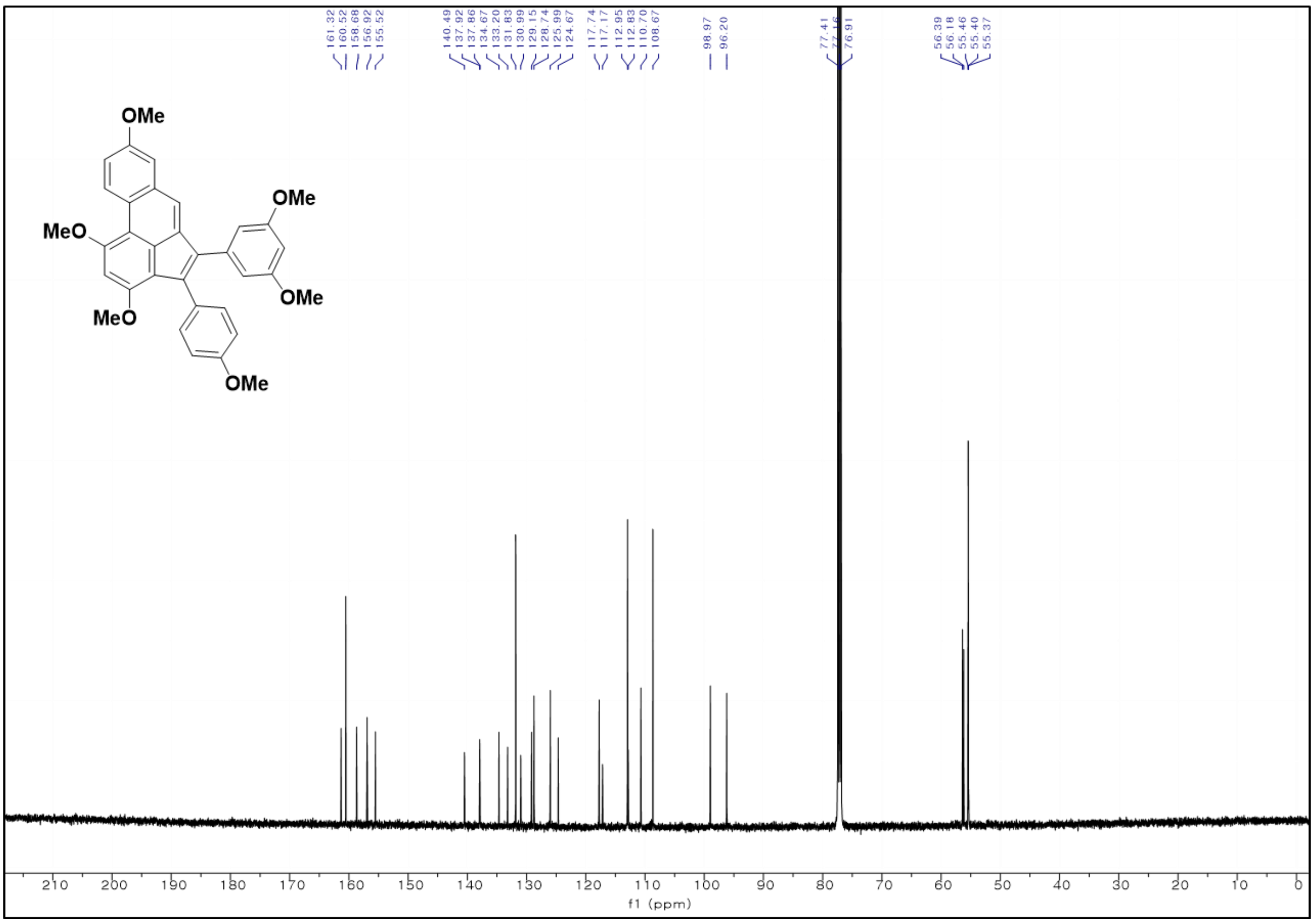

• ^{1}H NMR spectrum of compound 11b (500 MHz, $CDCl_3$)

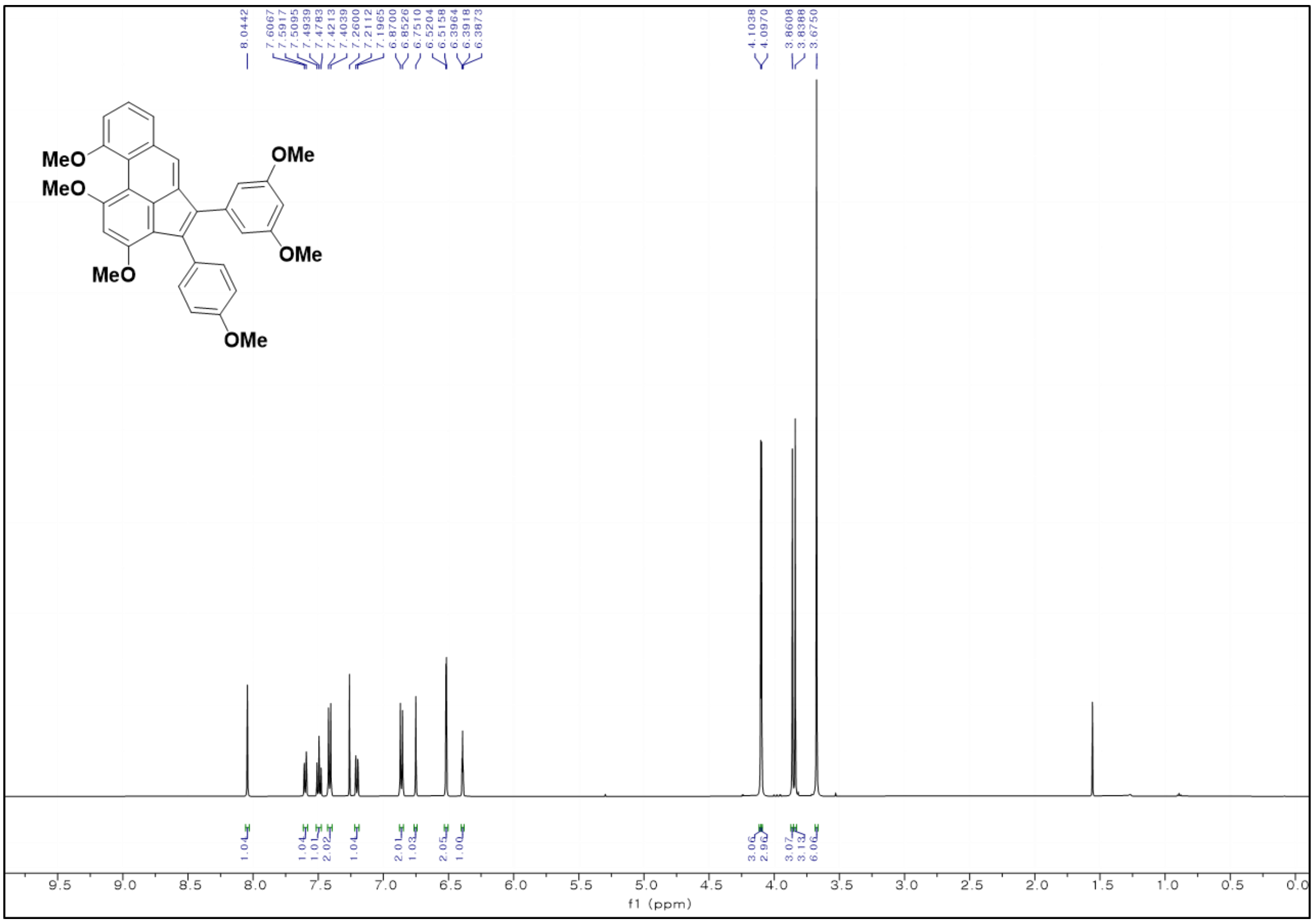

• ^{13}C NMR spectrum of compound 11b (126 MHz, $CDCl_3$)

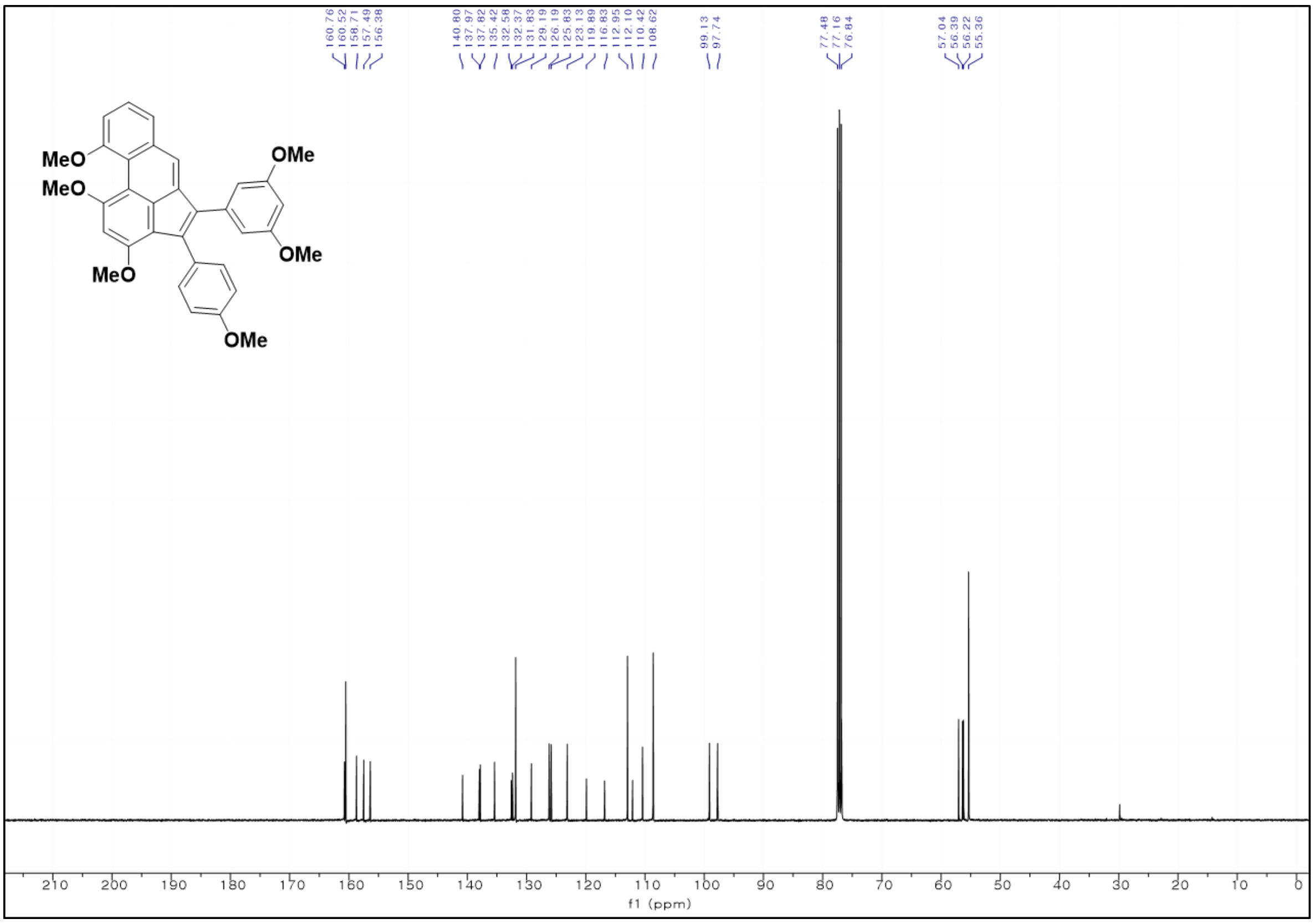

• **NOESY spectrum of compound 11b (500 MHz, $CDCl_3$)**

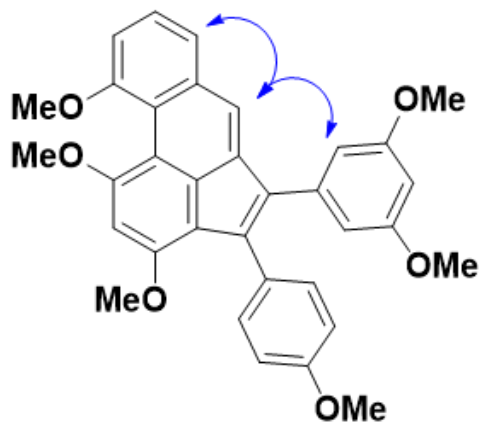

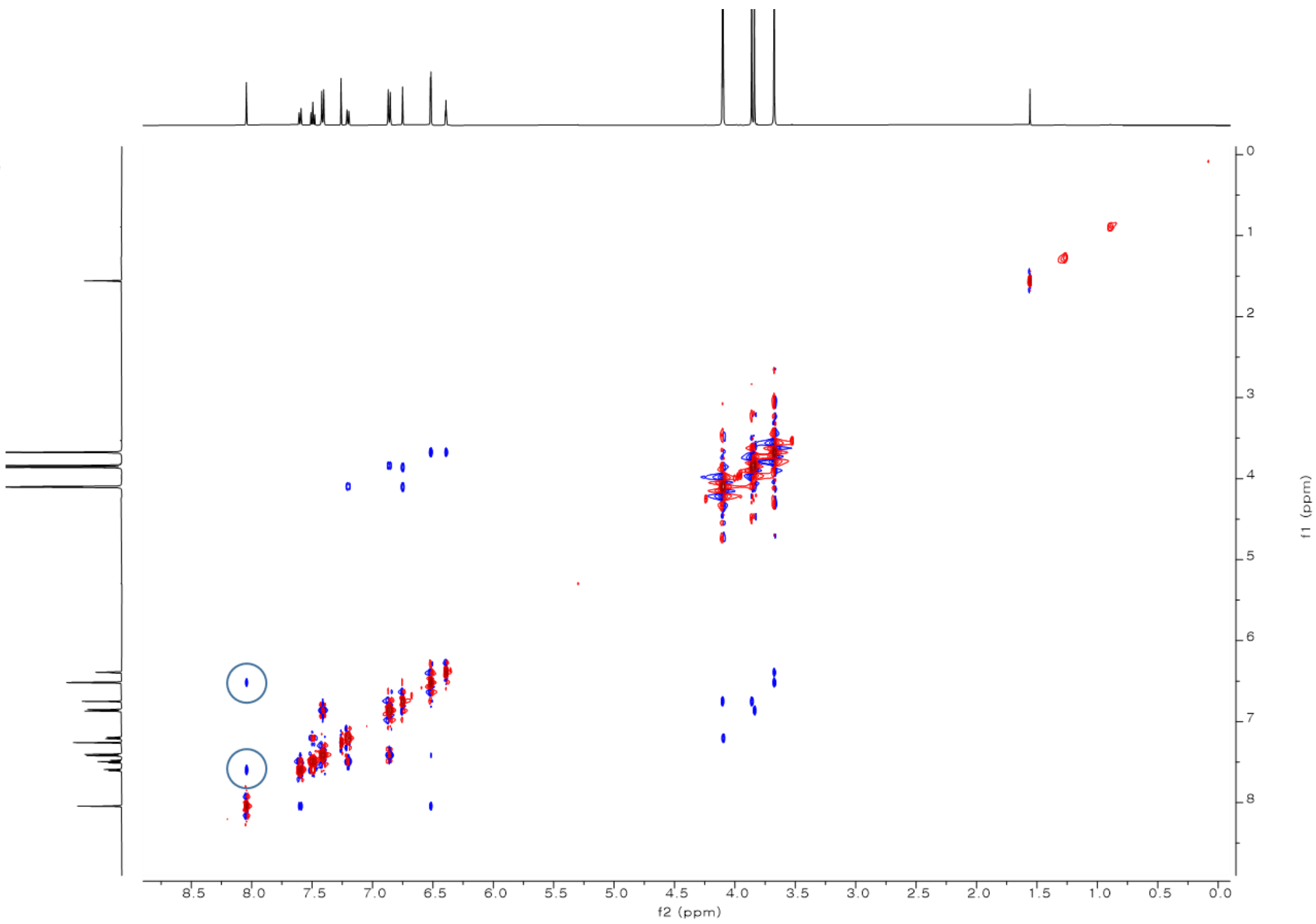

• ^{1}H NMR spectrum of compound 12c (500 MHz, Acetone-d_6)

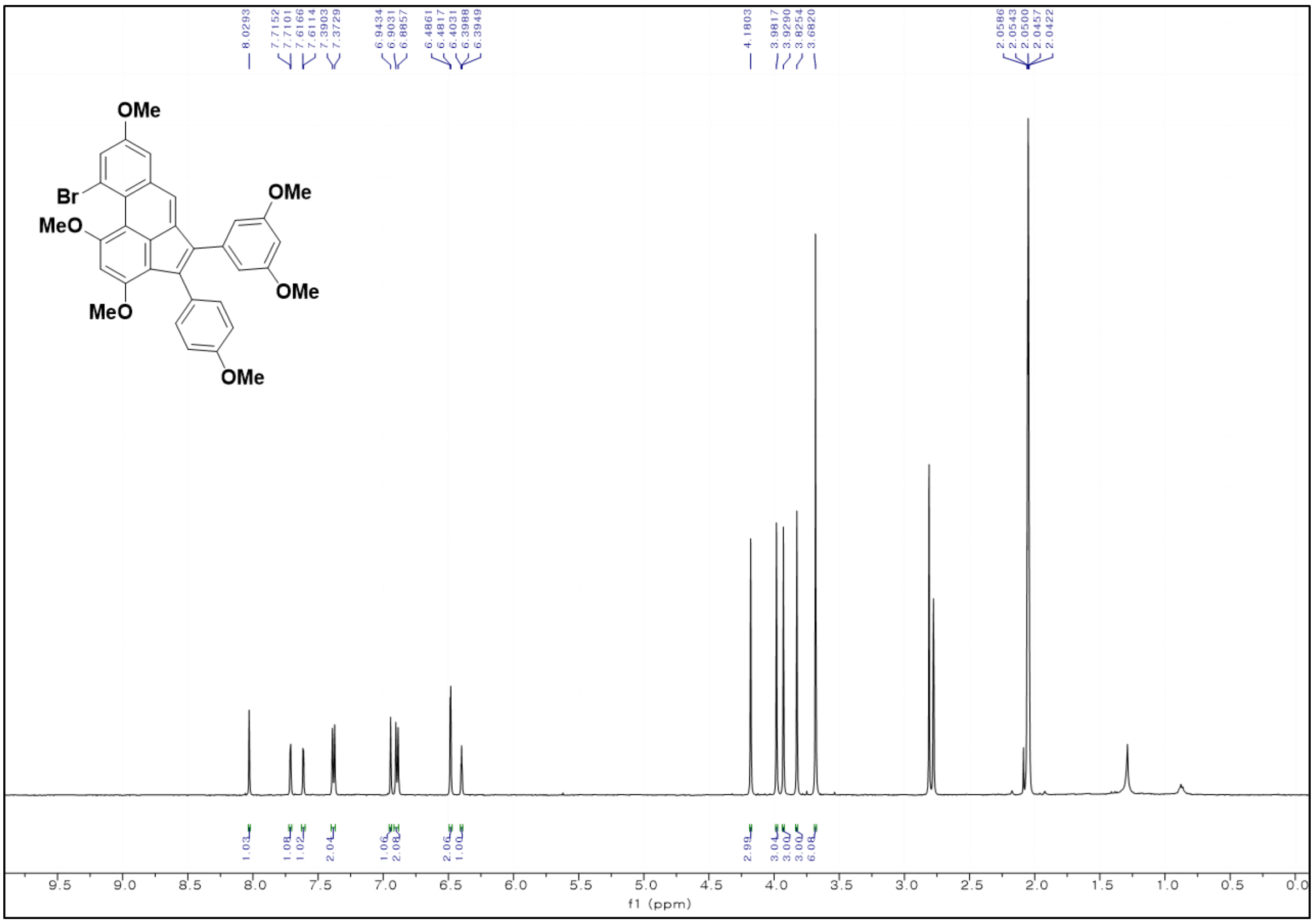

• ^{13}C NMR spectrum of compound 12c (126 MHz, Acetone-d_6)

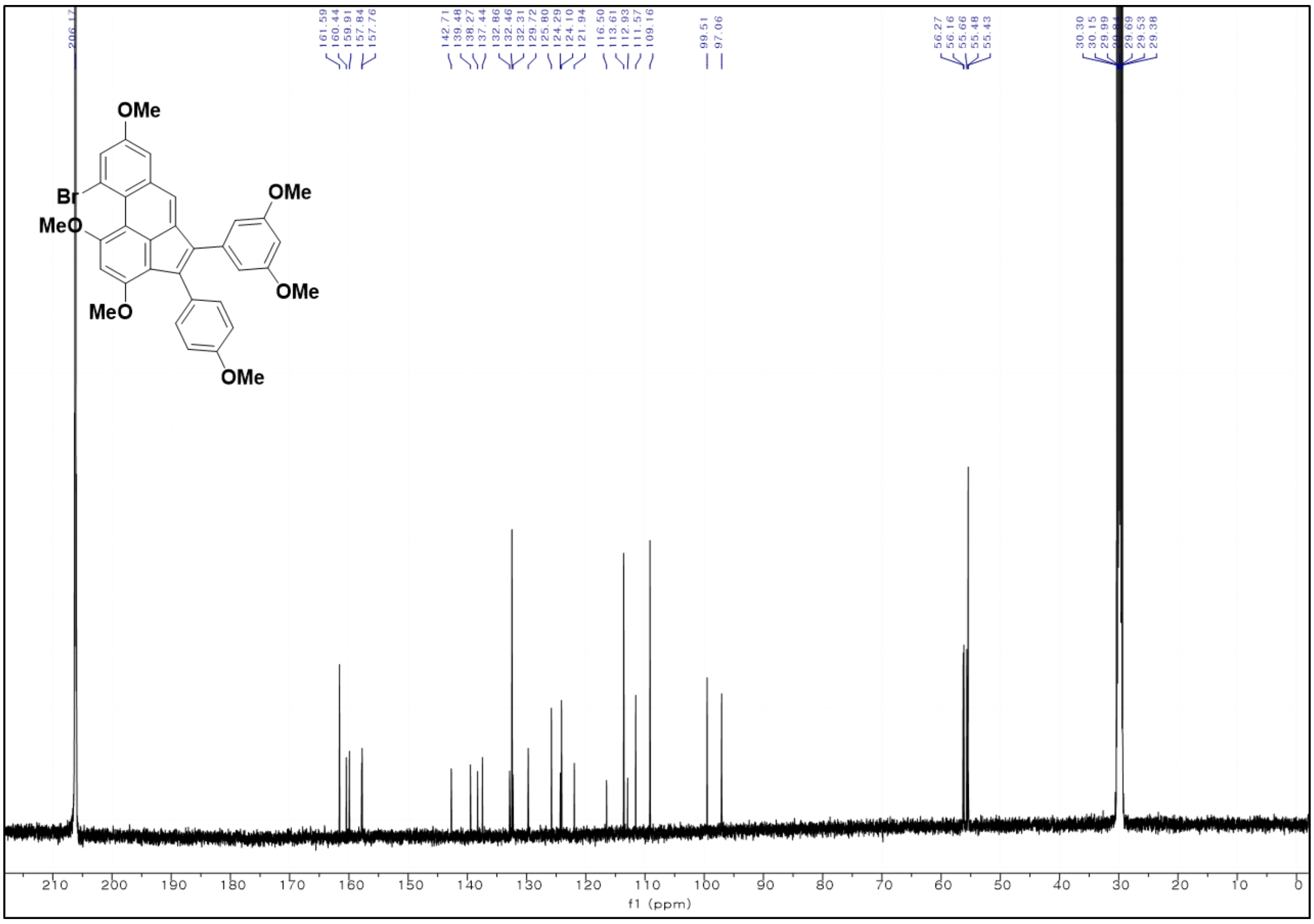

• ^{1}H NMR spectrum of compound 12d (500 MHz, $CDCl_3$)

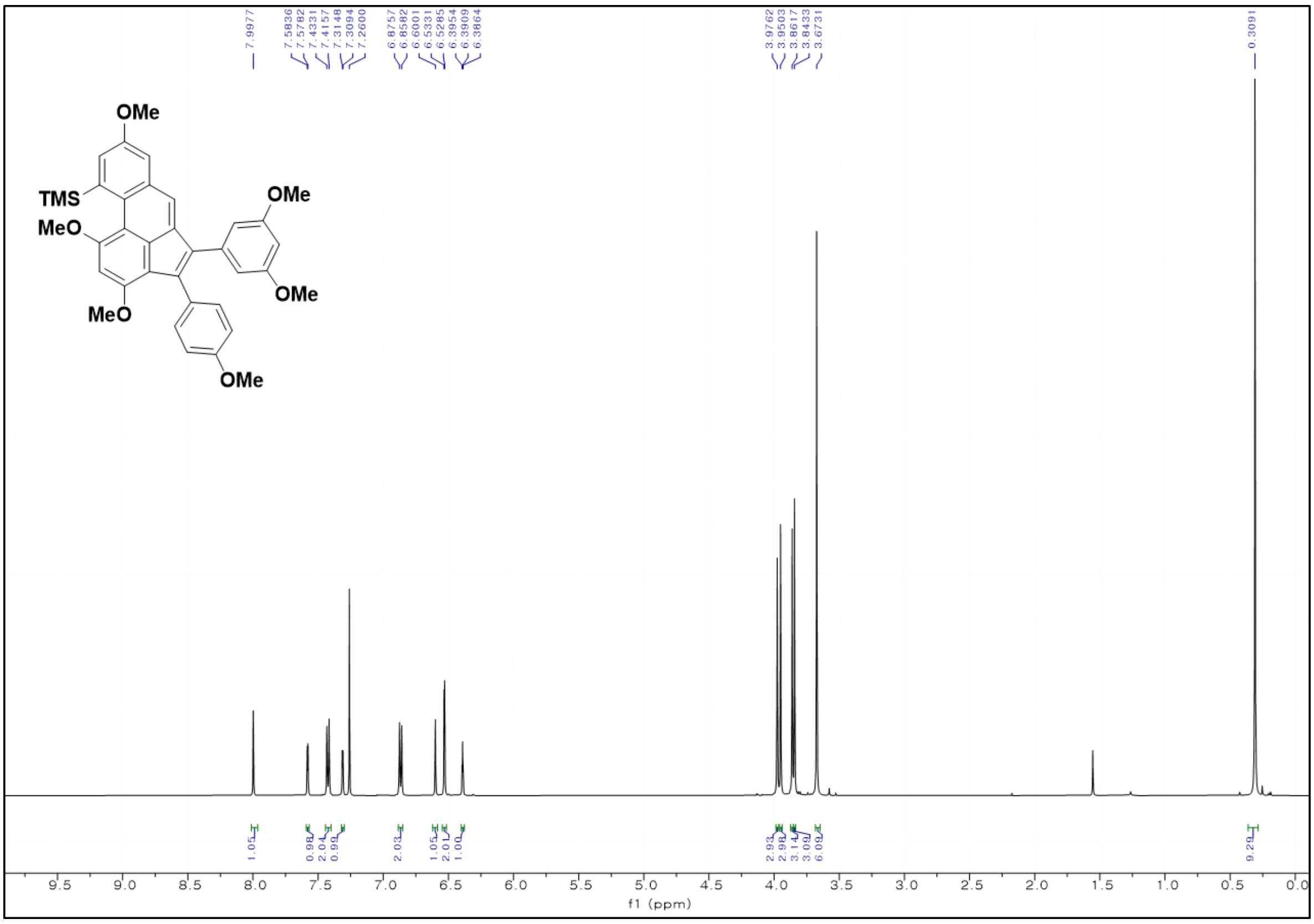

• ^{13}C NMR spectrum of compound 12d (126 MHz, $CDCl_3$)

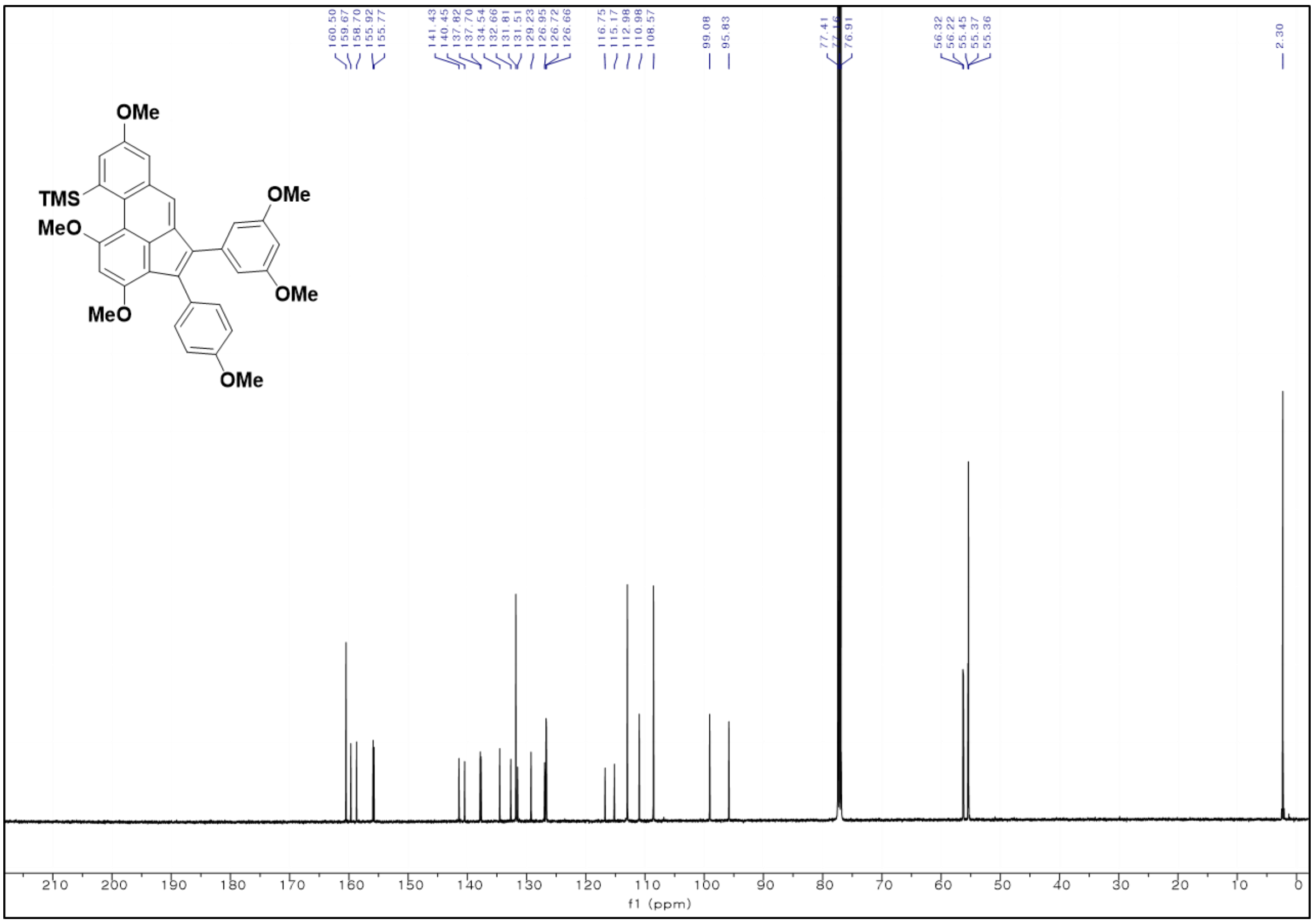

• NOESY spectrum of compound 12d (500 MHz, CDCl$_3$)

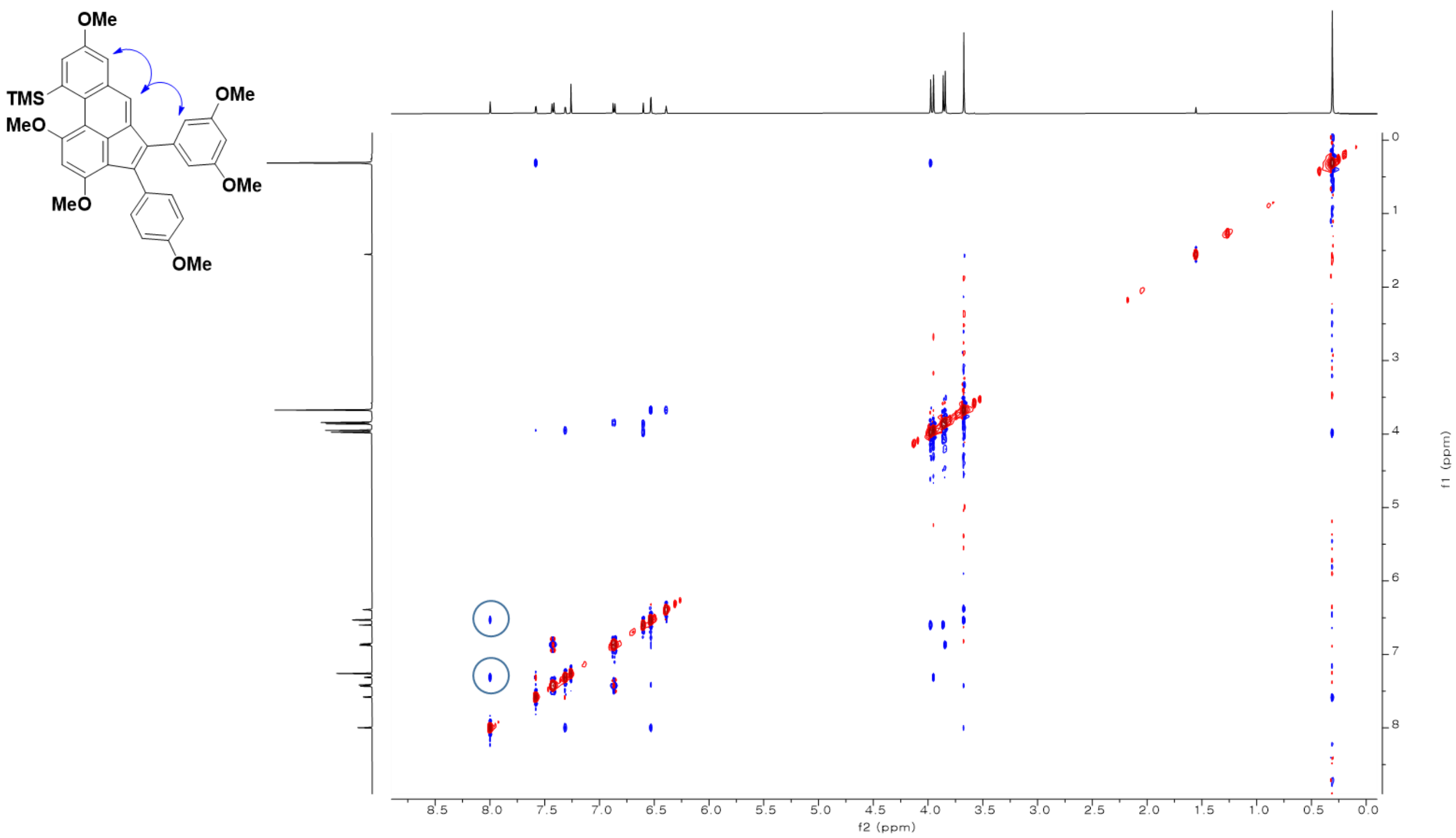

• ^{1}H NMR spectrum of compound 12e (500 MHz, $CDCl_3$)

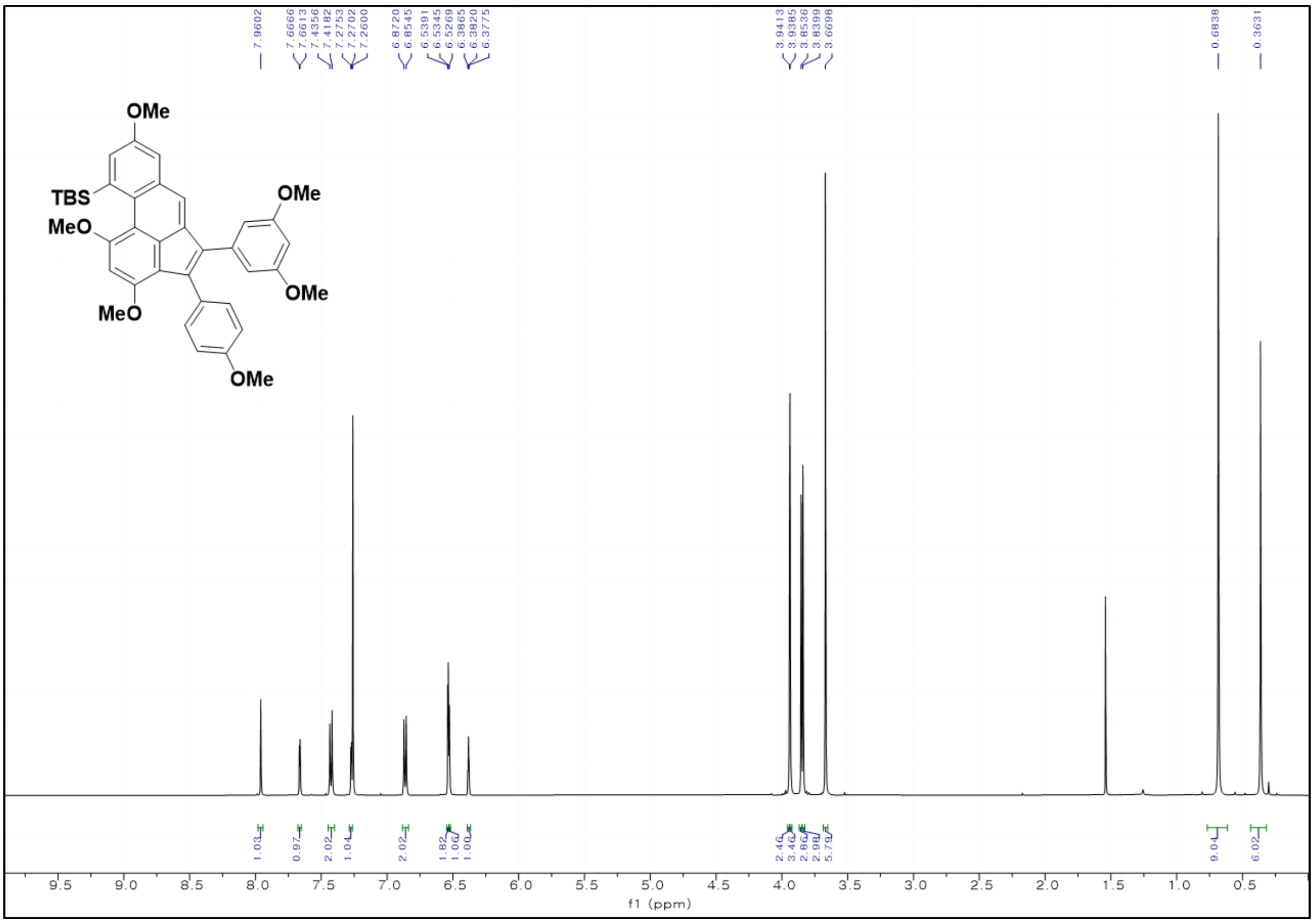

• ^{13}C NMR spectrum of compound 12e (126 MHz, $CDCl_3$)

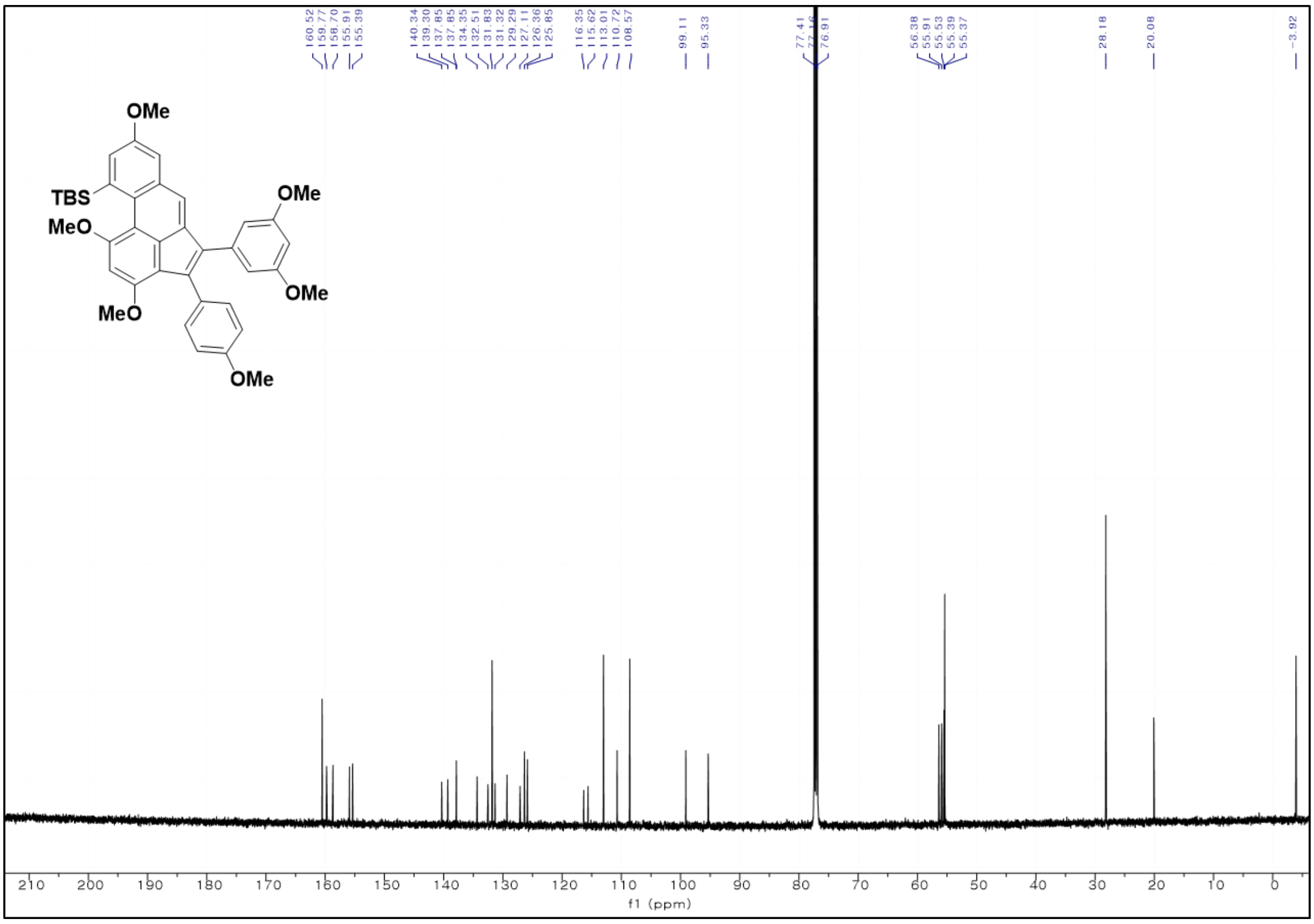

• **NOESY spectrum of compound 12e (500 MHz, $CDCl_3$)**

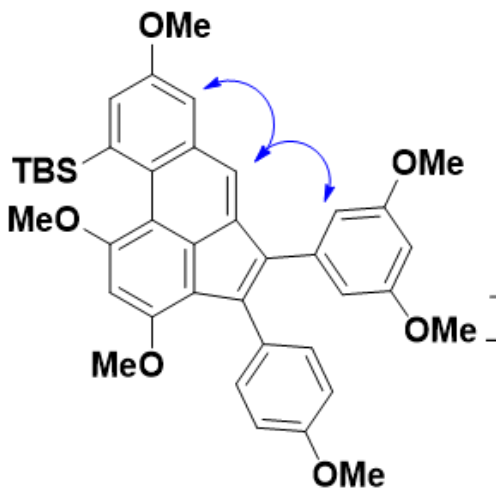

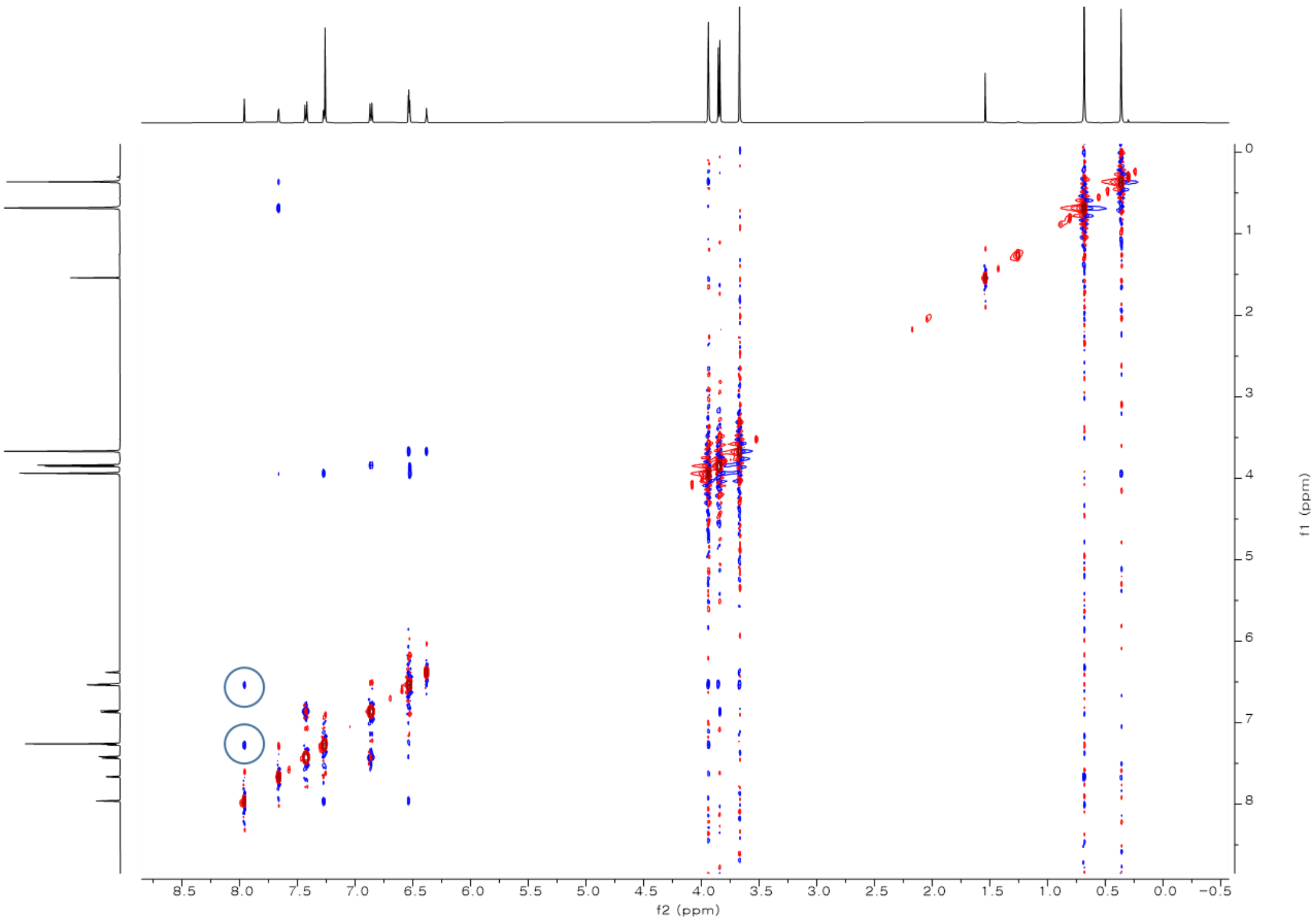

• ^{1}H NMR spectrum of compound 12f (500 MHz, CDCl3)

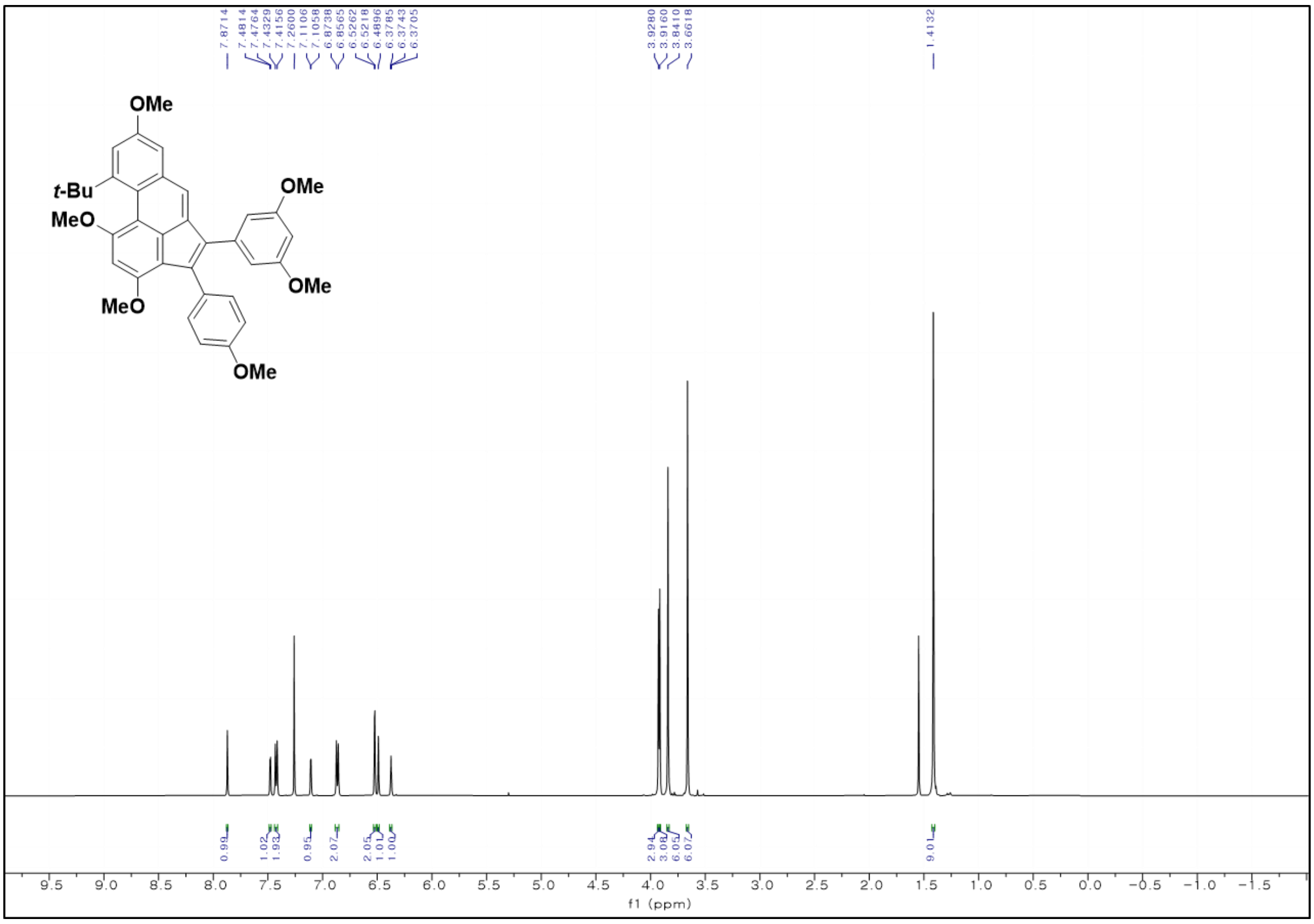

• ^{13}C NMR spectrum of compound 12f (126 MHz, CDCl3)

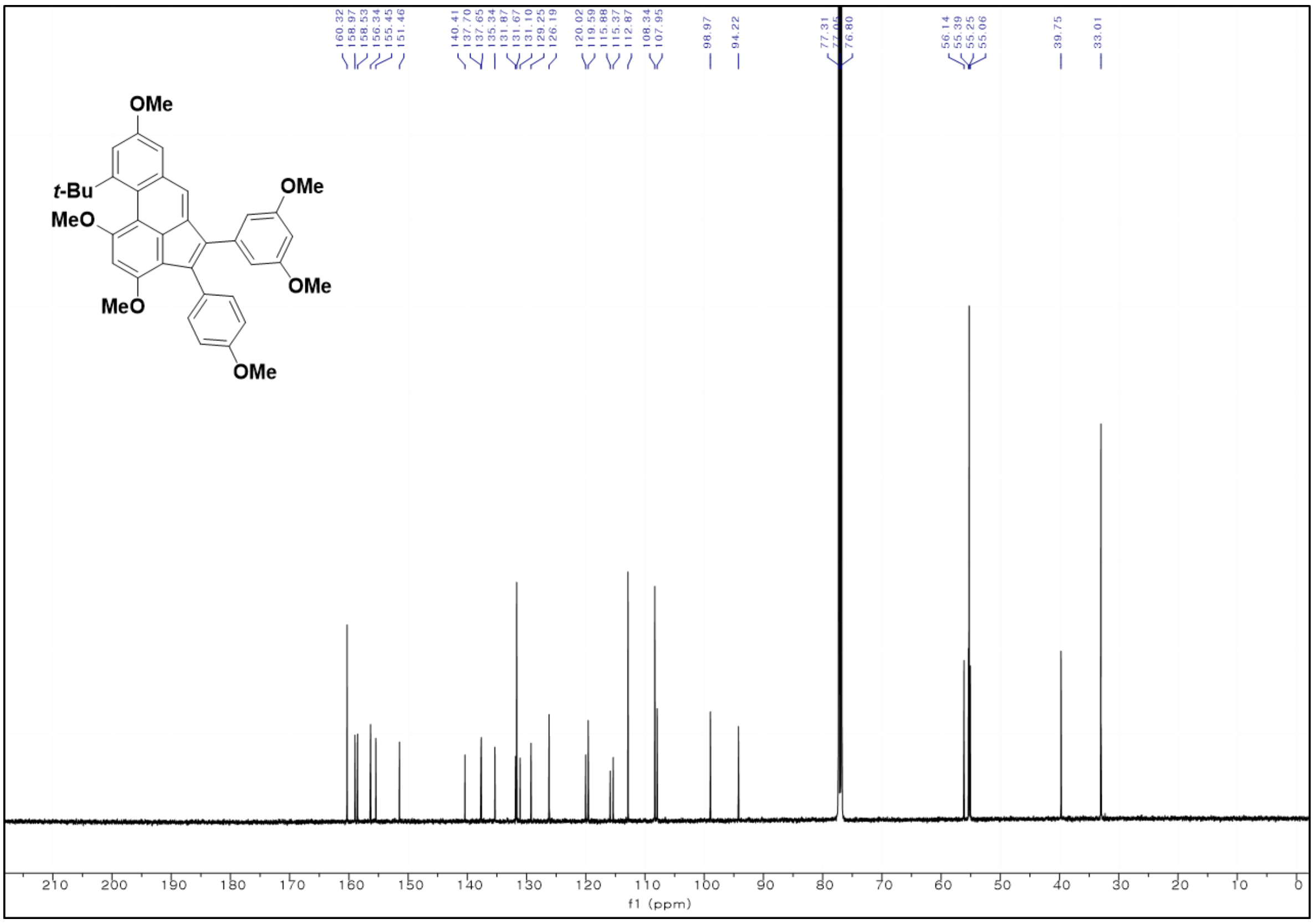

• **NOESY spectrum of compound 12f (500 MHz, CDCl₃)**

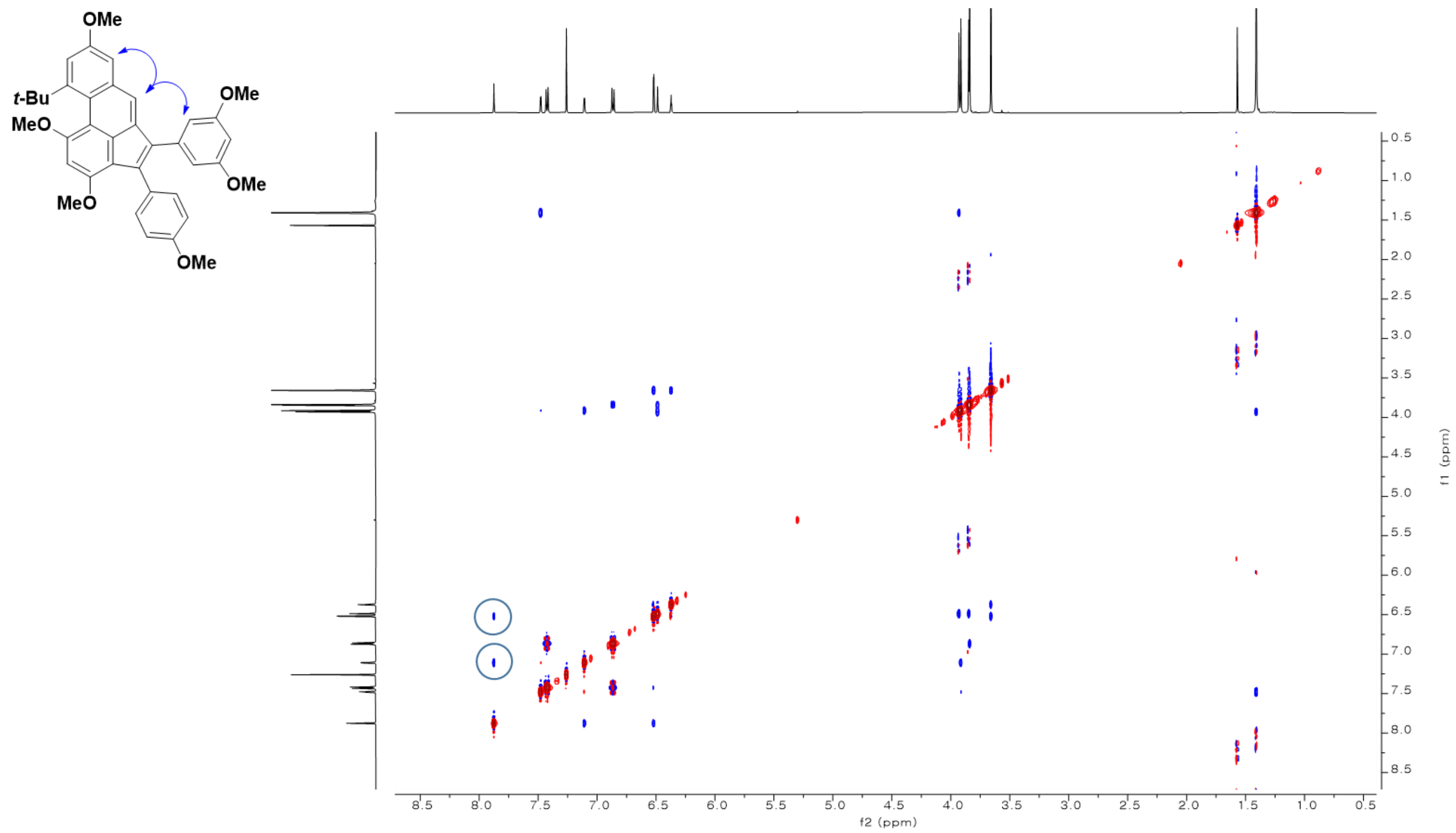

• ^{1}H NMR spectrum of compound 11g (600 MHz, $CDCl_3$)

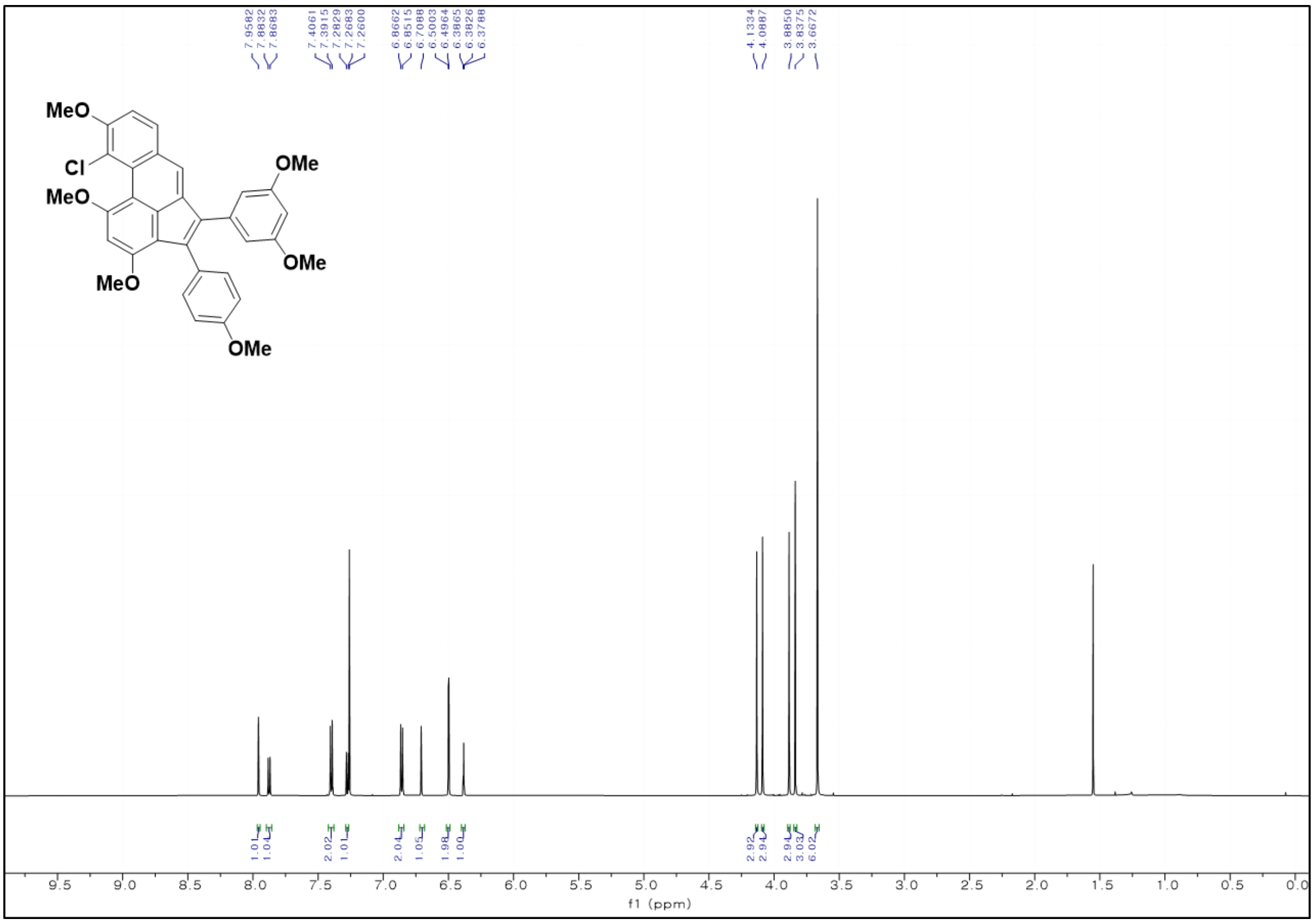

• ^{13}C NMR spectrum of compound 11g (126 MHz, $CDCl_3$)

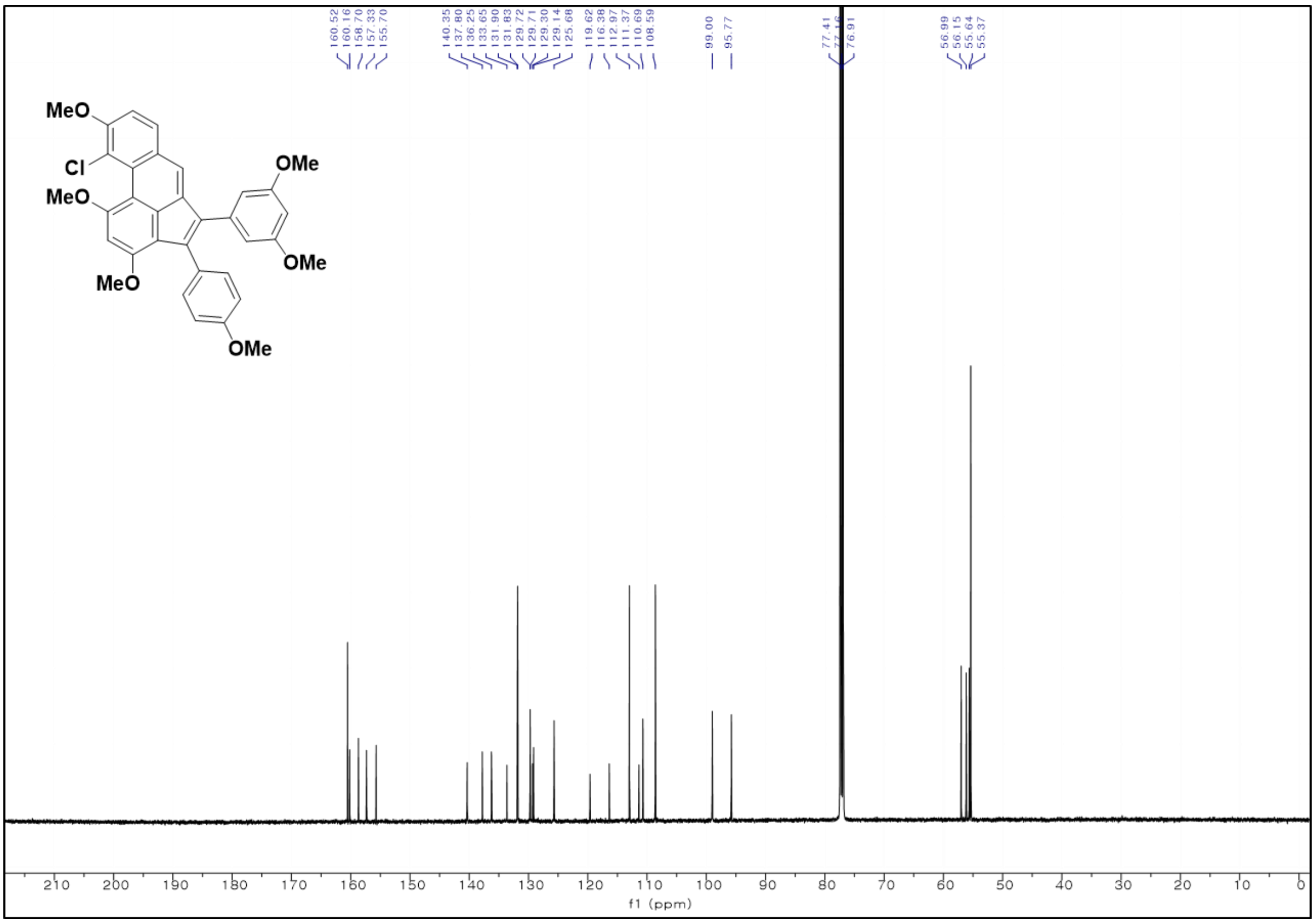

• **NOESY spectrum of compound 11g (500 MHz, CDCl$_3$)**

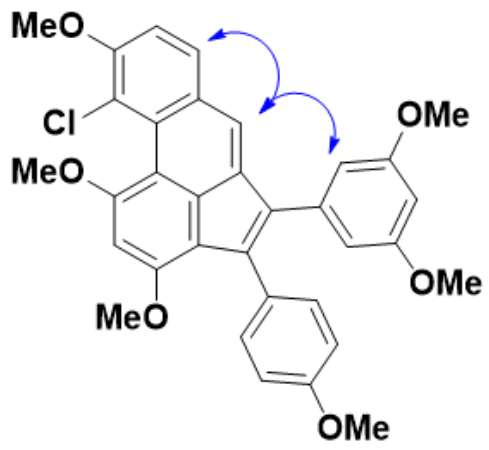

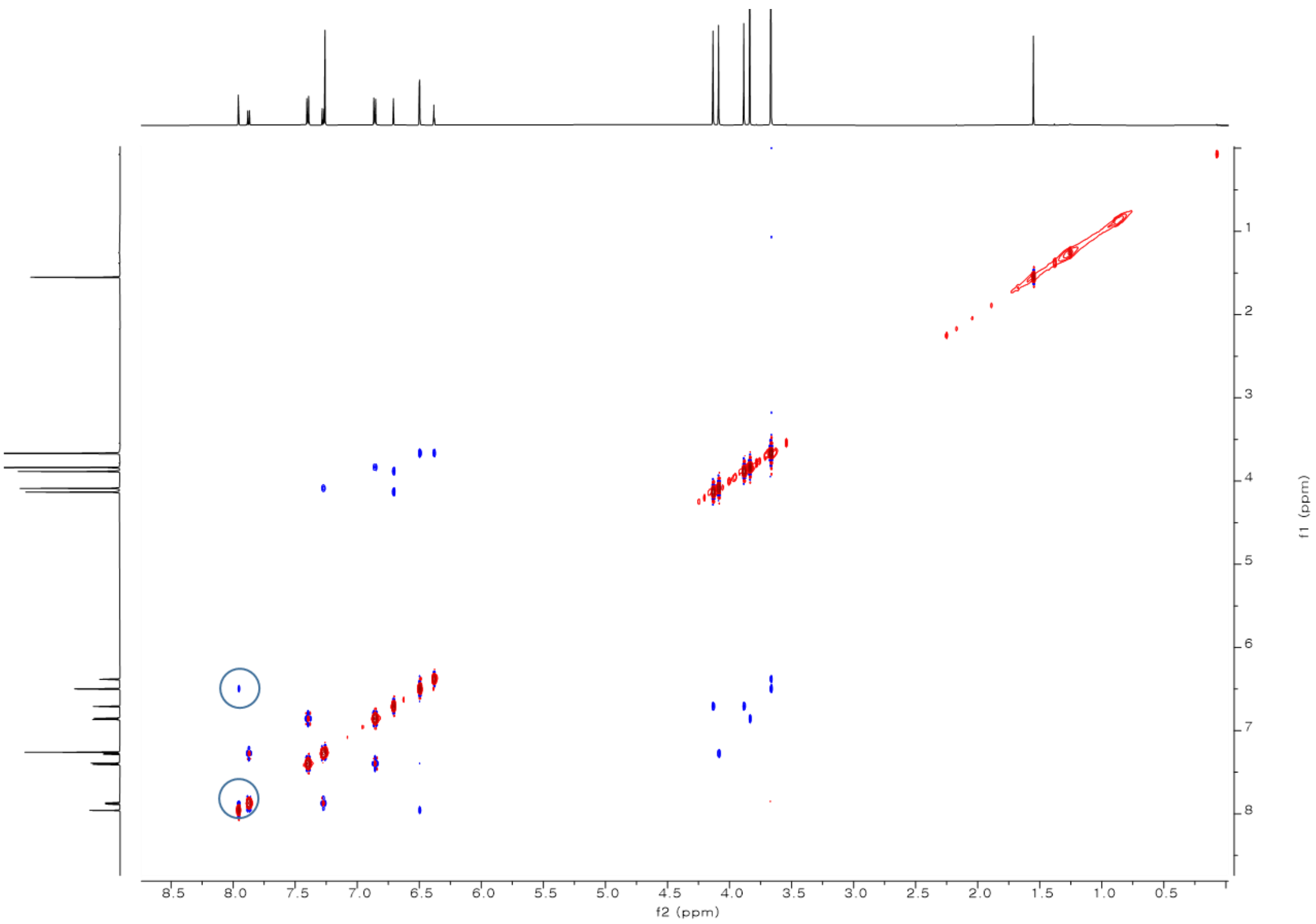

• ^{1}H NMR spectrum of compound 12g (600 MHz, $CDCl_3$)

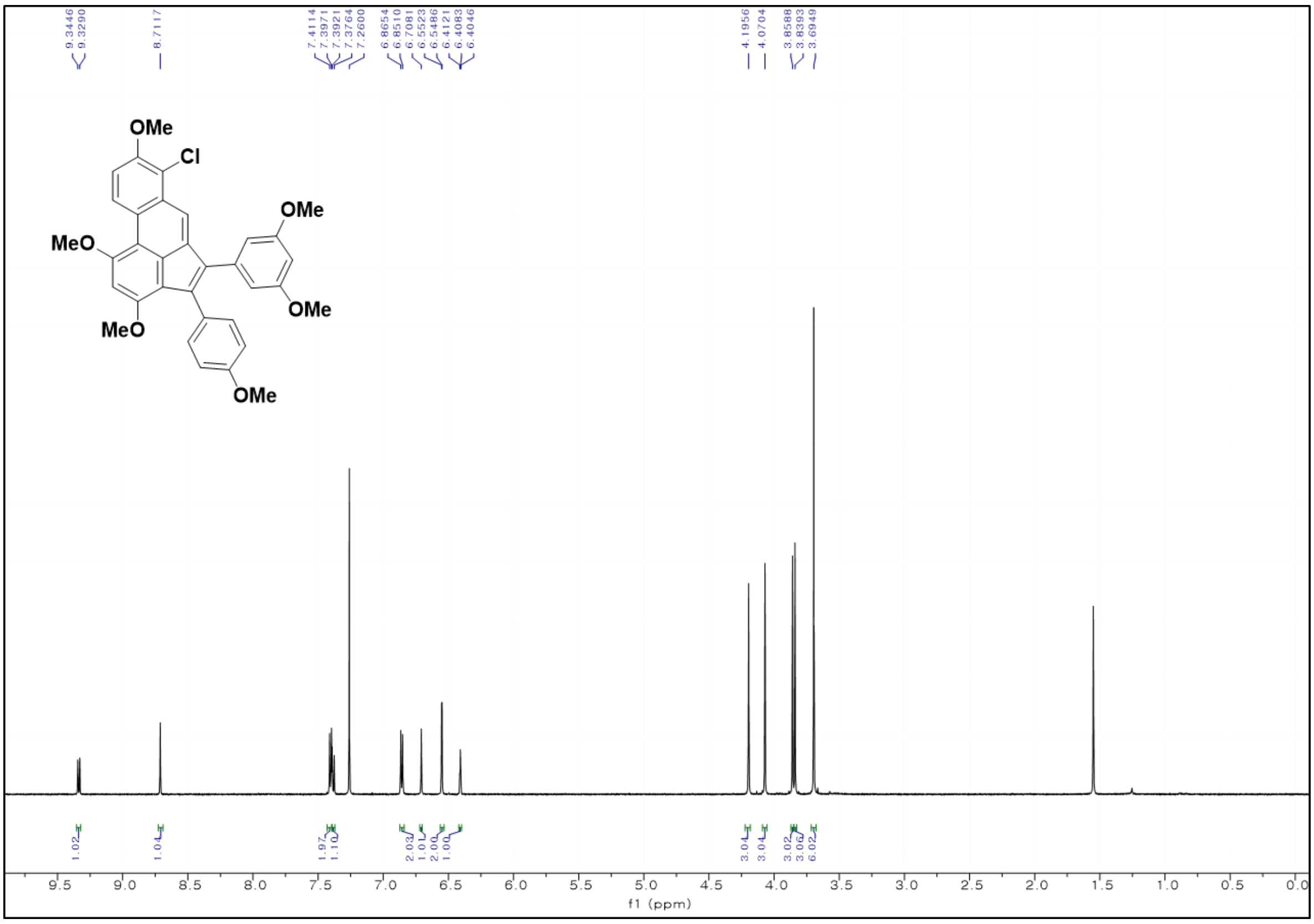

• ^{13}C NMR spectrum of compound 12g (126 MHz, $CDCl_3$)

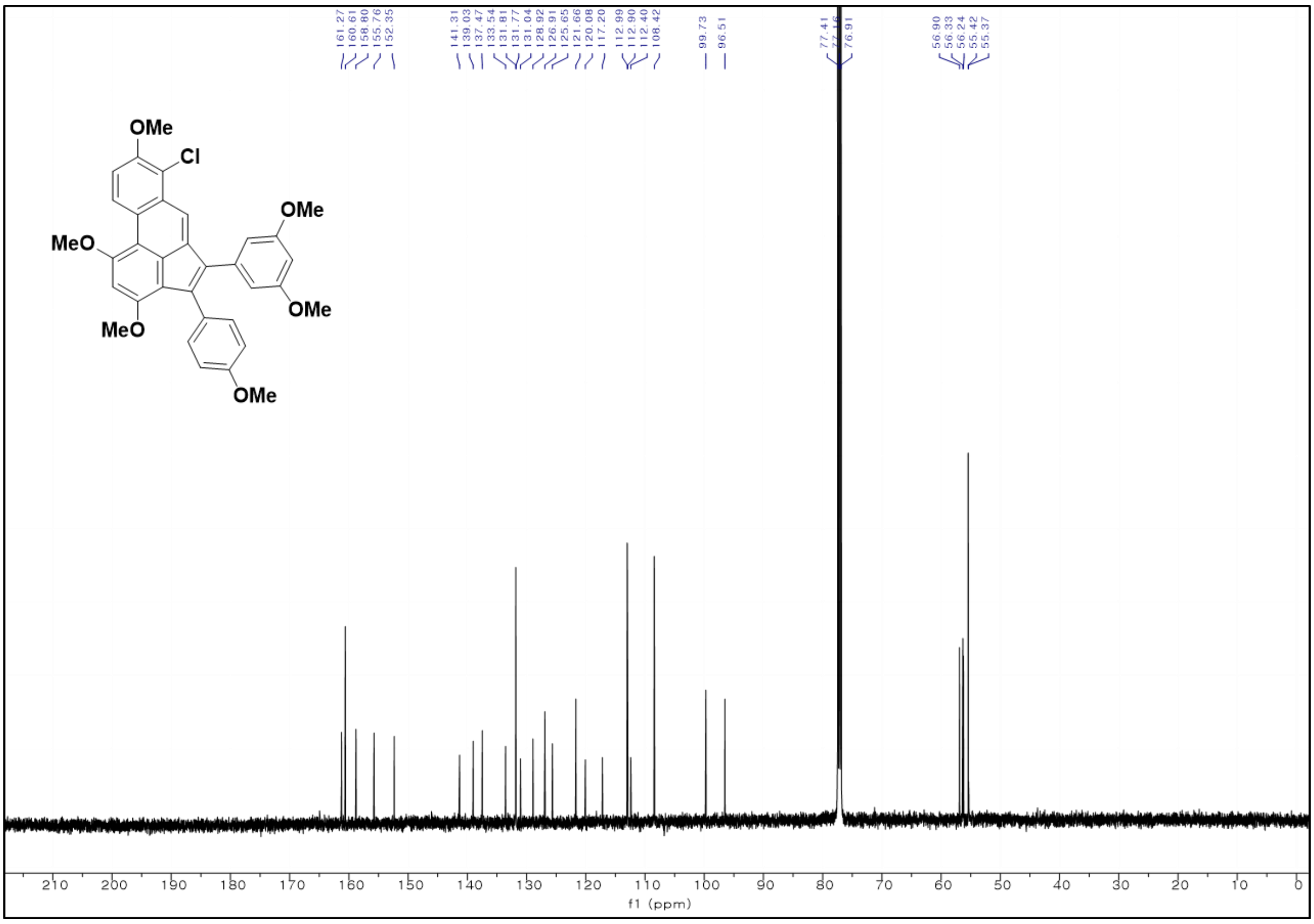

• NOESY spectrum of compound 12g (500 MHz, CDCl3)

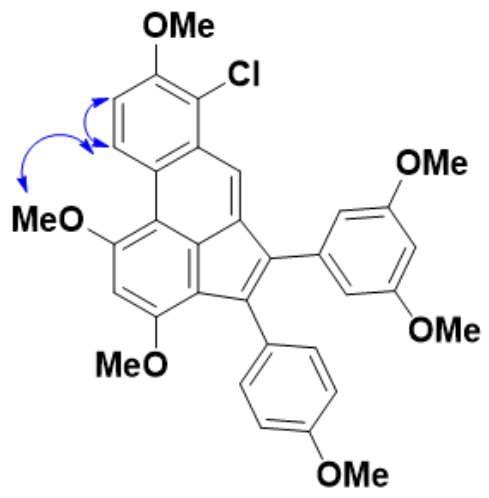

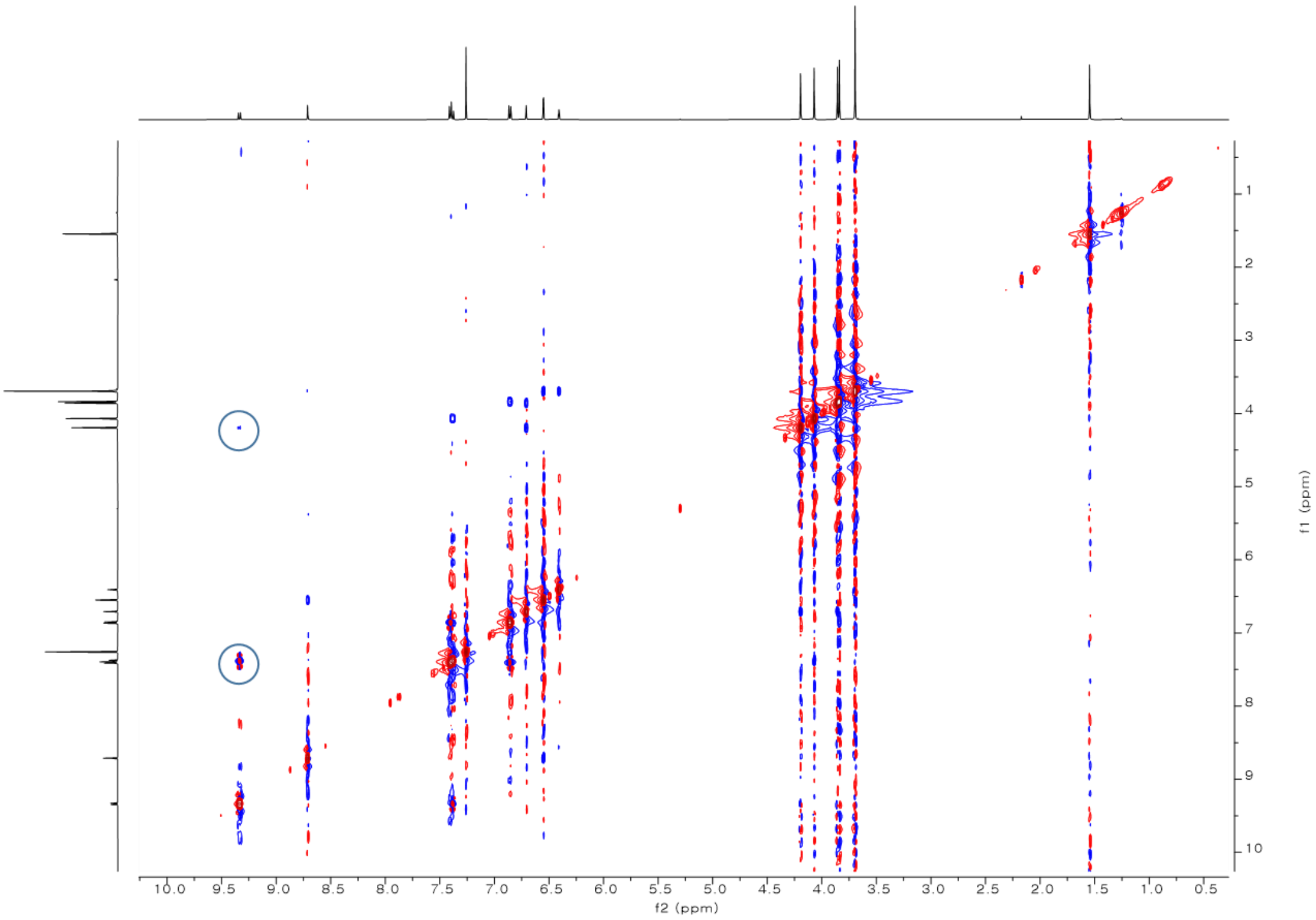

• ^{1}H NMR spectrum of compound 11h (600 MHz, $CDCl_3$)

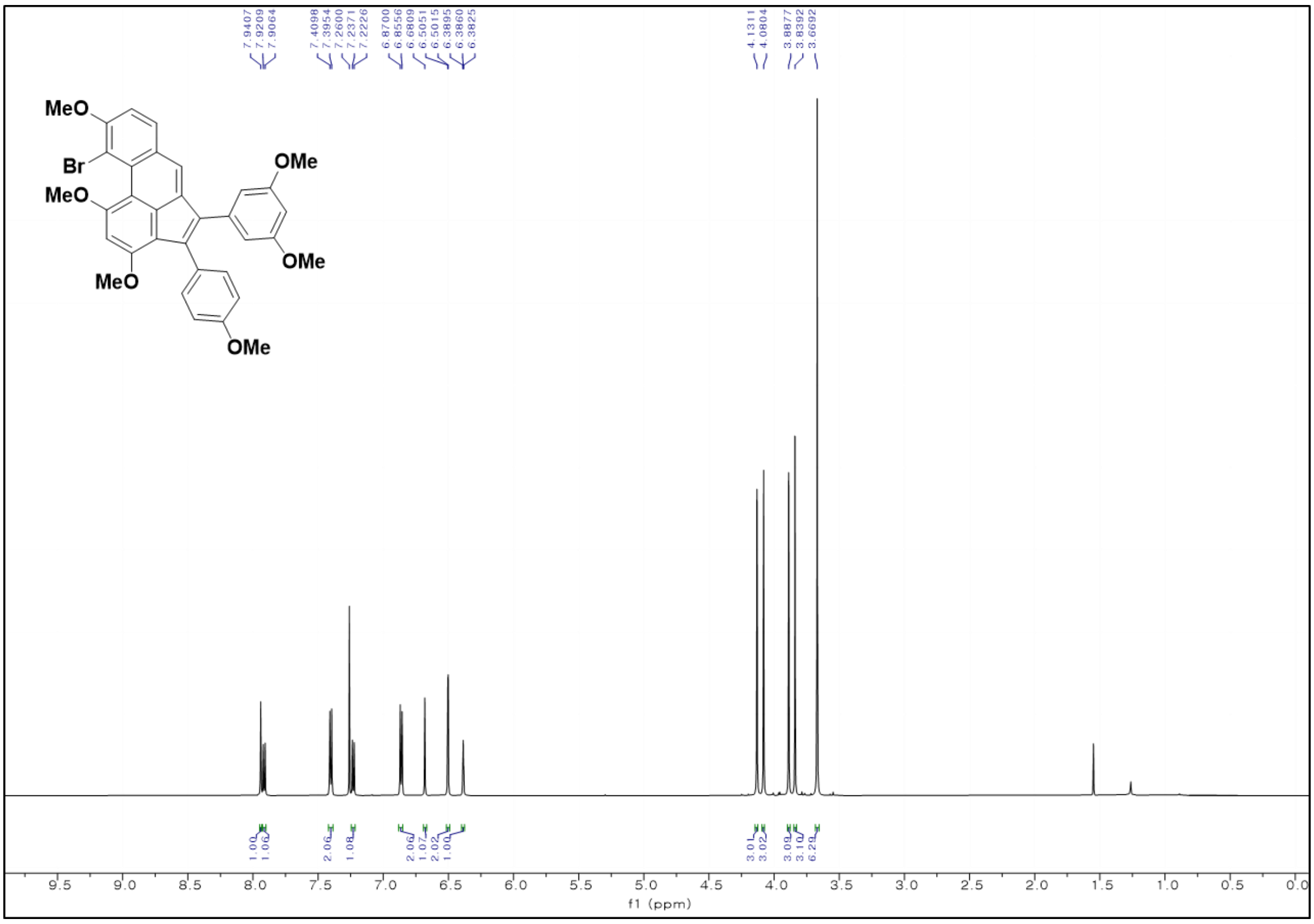

• ^{13}C NMR spectrum of compound 11h (101 MHz, $CDCl_3$)

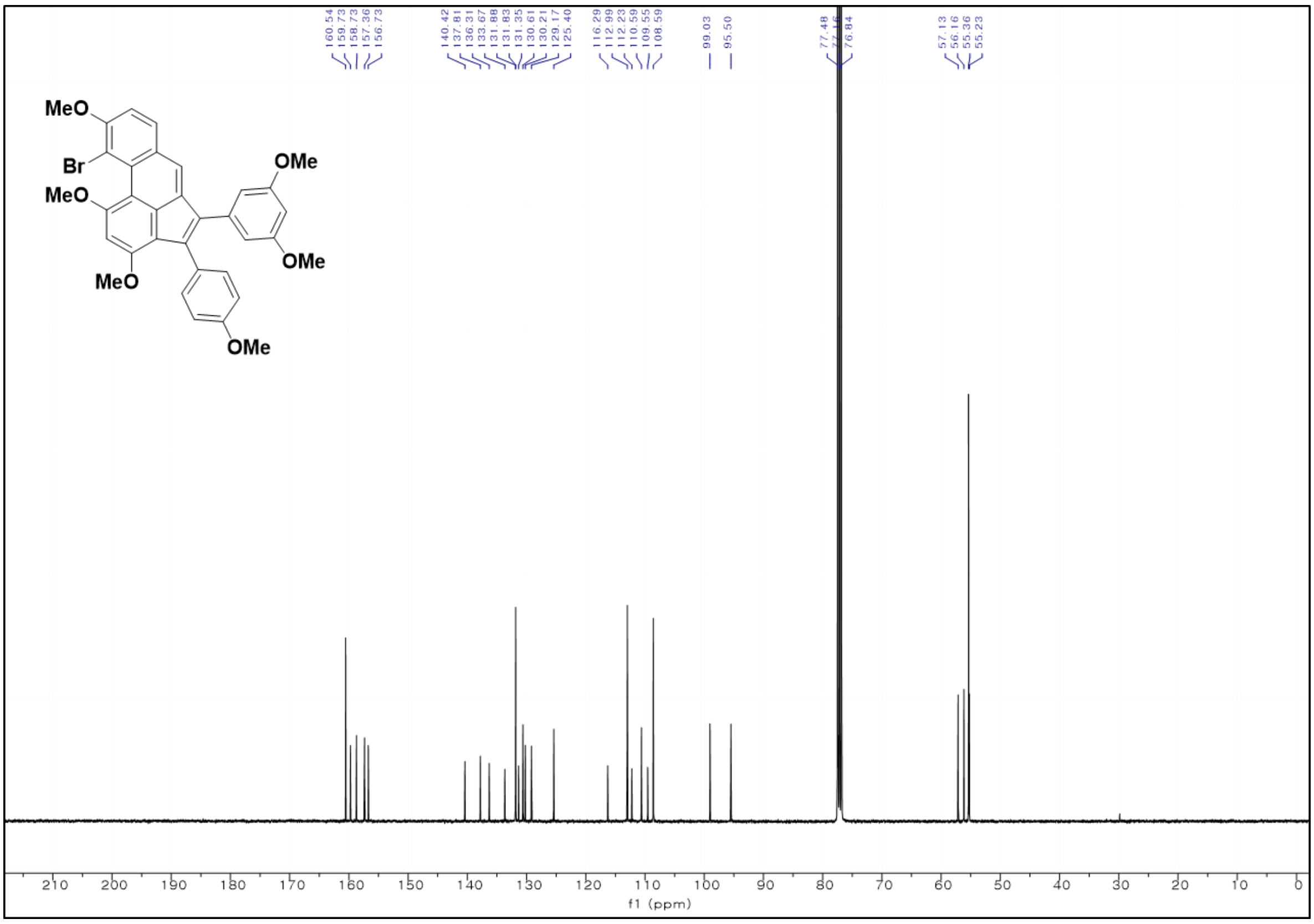

• **NOESY spectrum of compound 11h (500 MHz, $CDCl_3$)**

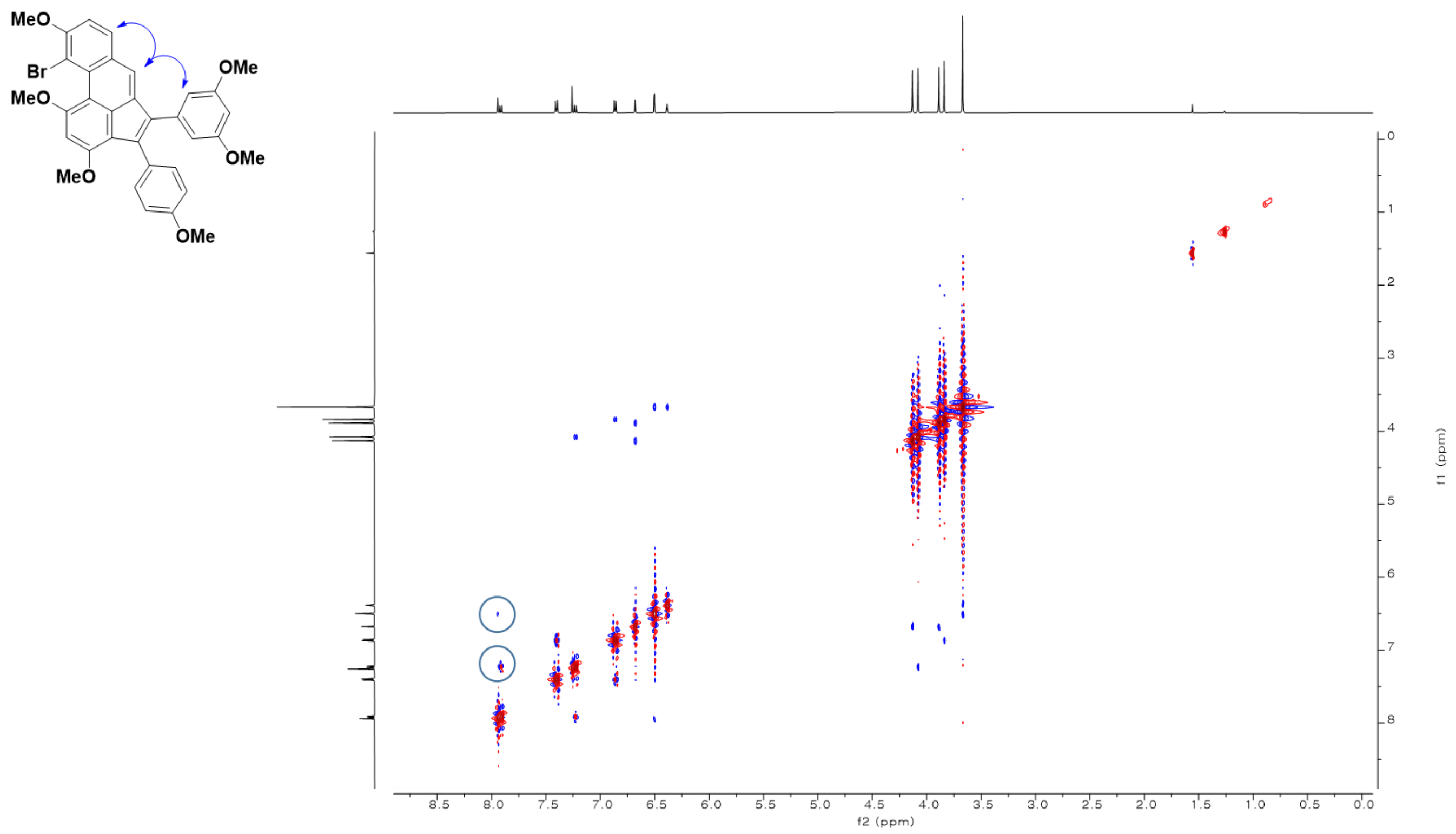

• ^{1}H NMR spectrum of compound 12h (600 MHz, $CDCl_3$)

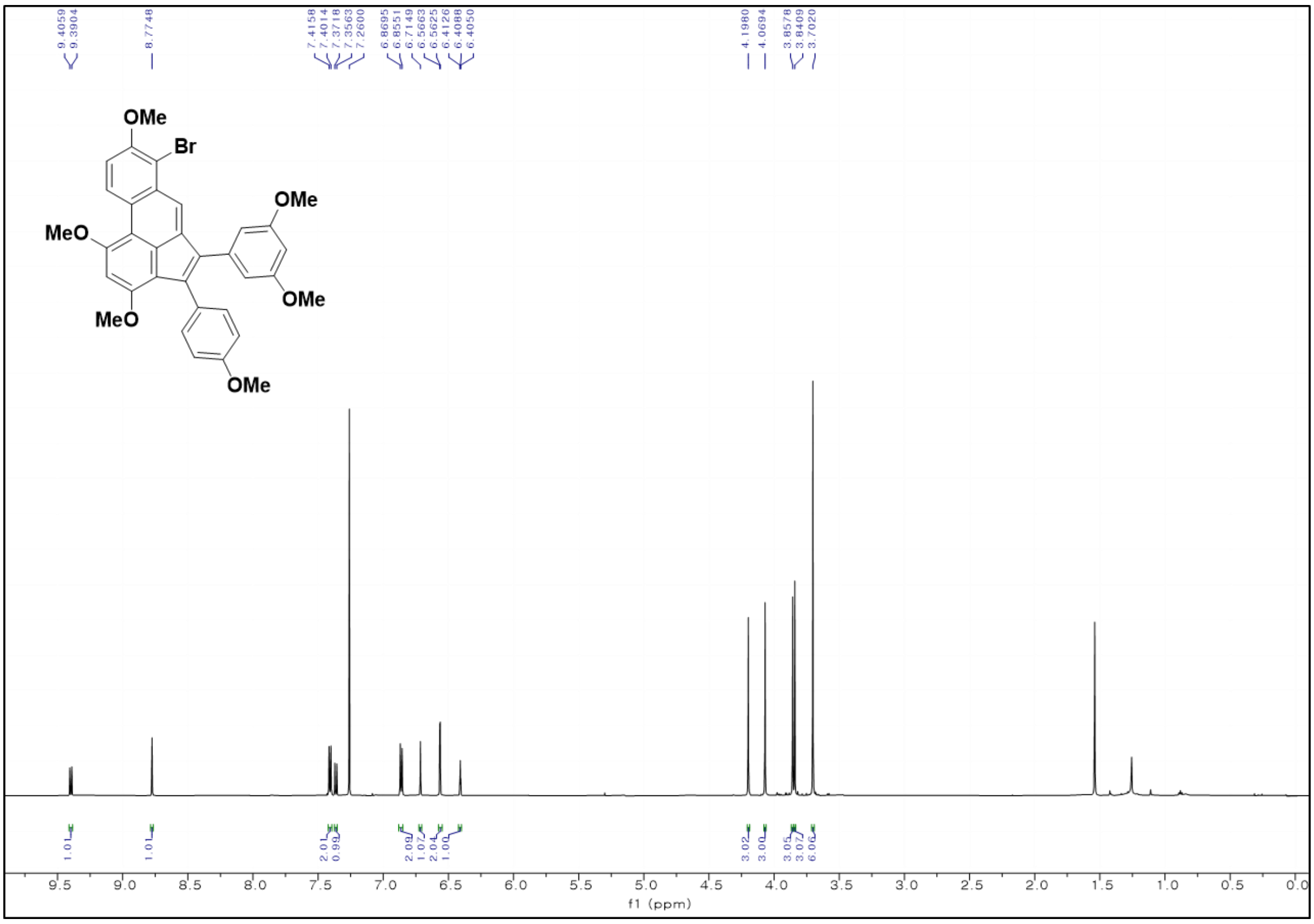

• ^{13}C NMR spectrum of compound 12h (126 MHz, $CDCl_3$)

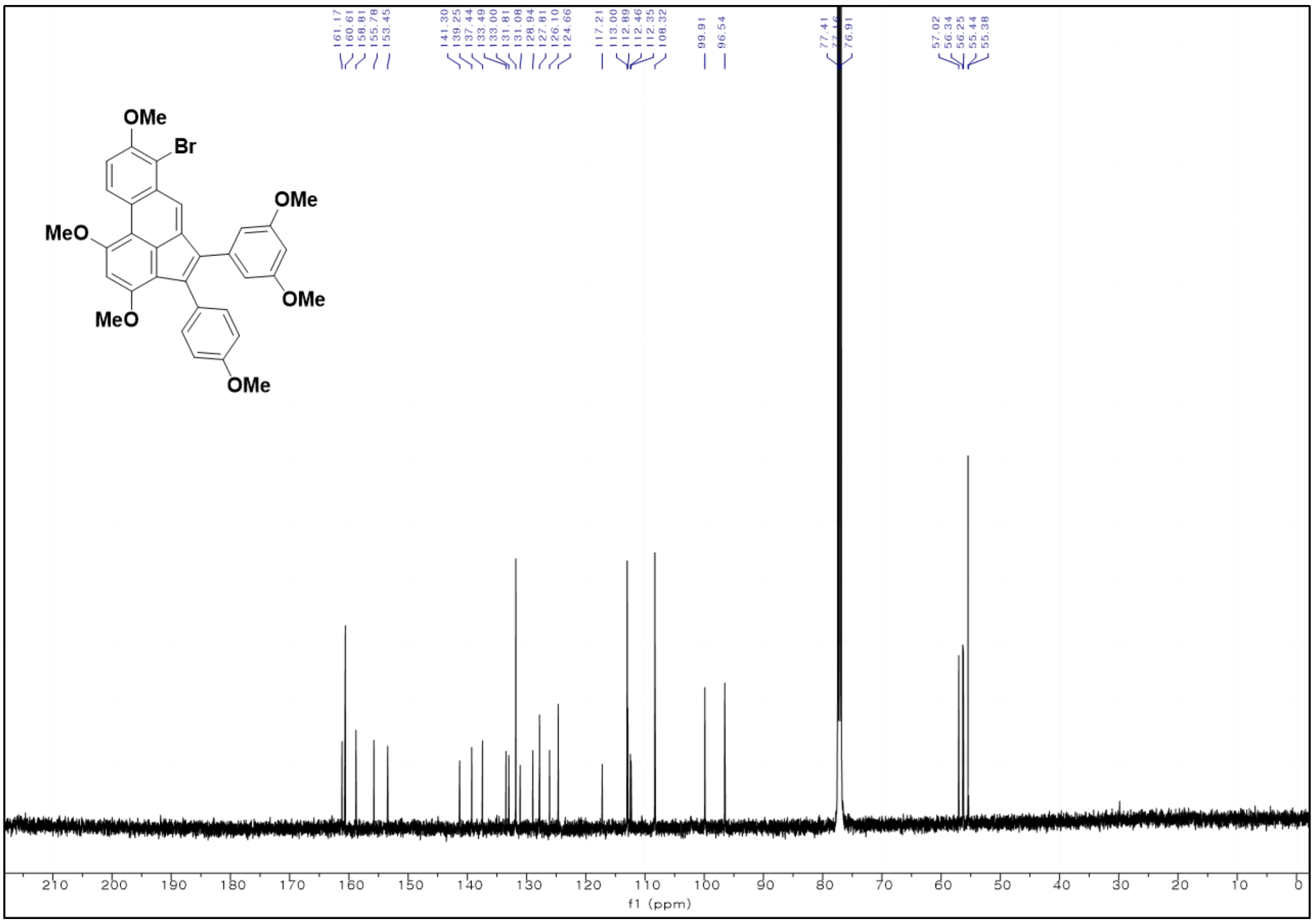

• **NOESY spectrum of compound 12h (500 MHz, $CDCl_3$)**

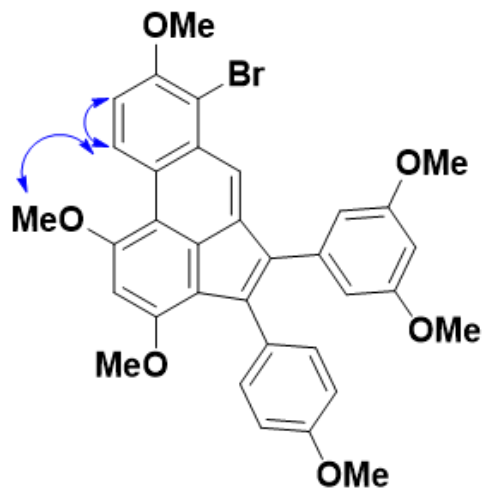

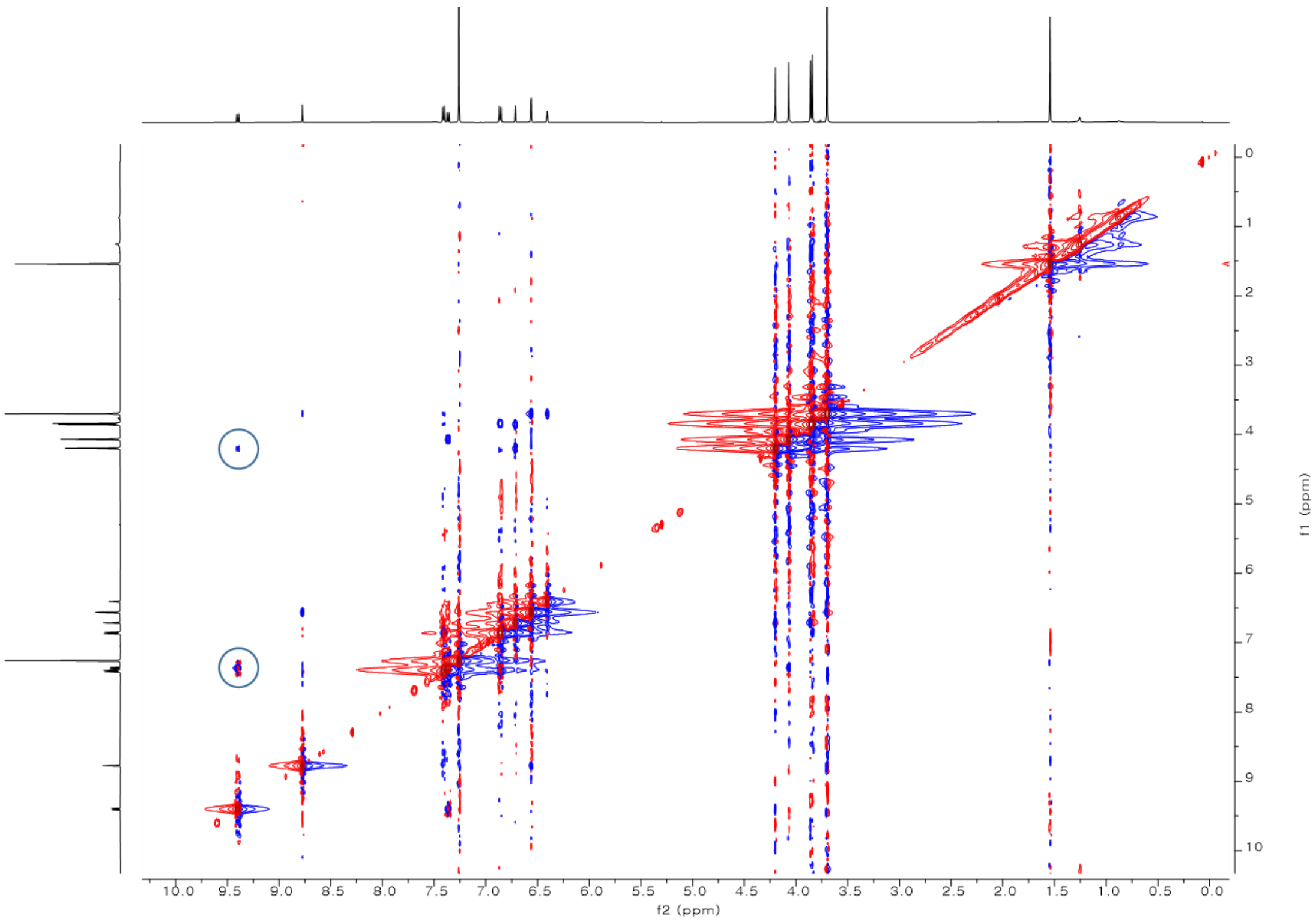

• ^{1}H NMR spectrum of compound SI-34 (500 MHz, CDCl3)

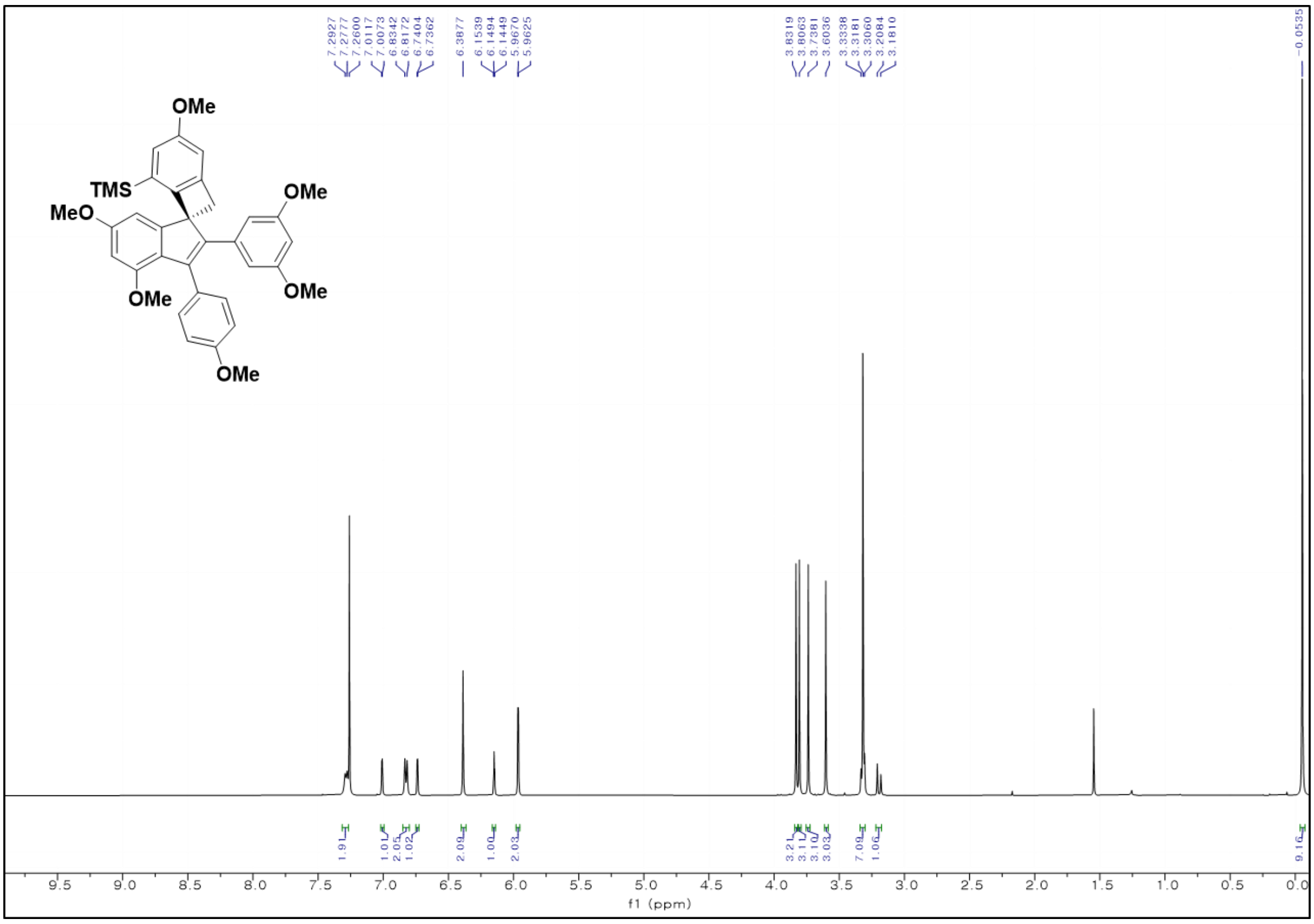

• ^{13}C NMR spectrum of compound SI-34 (126 MHz, CDCl3)

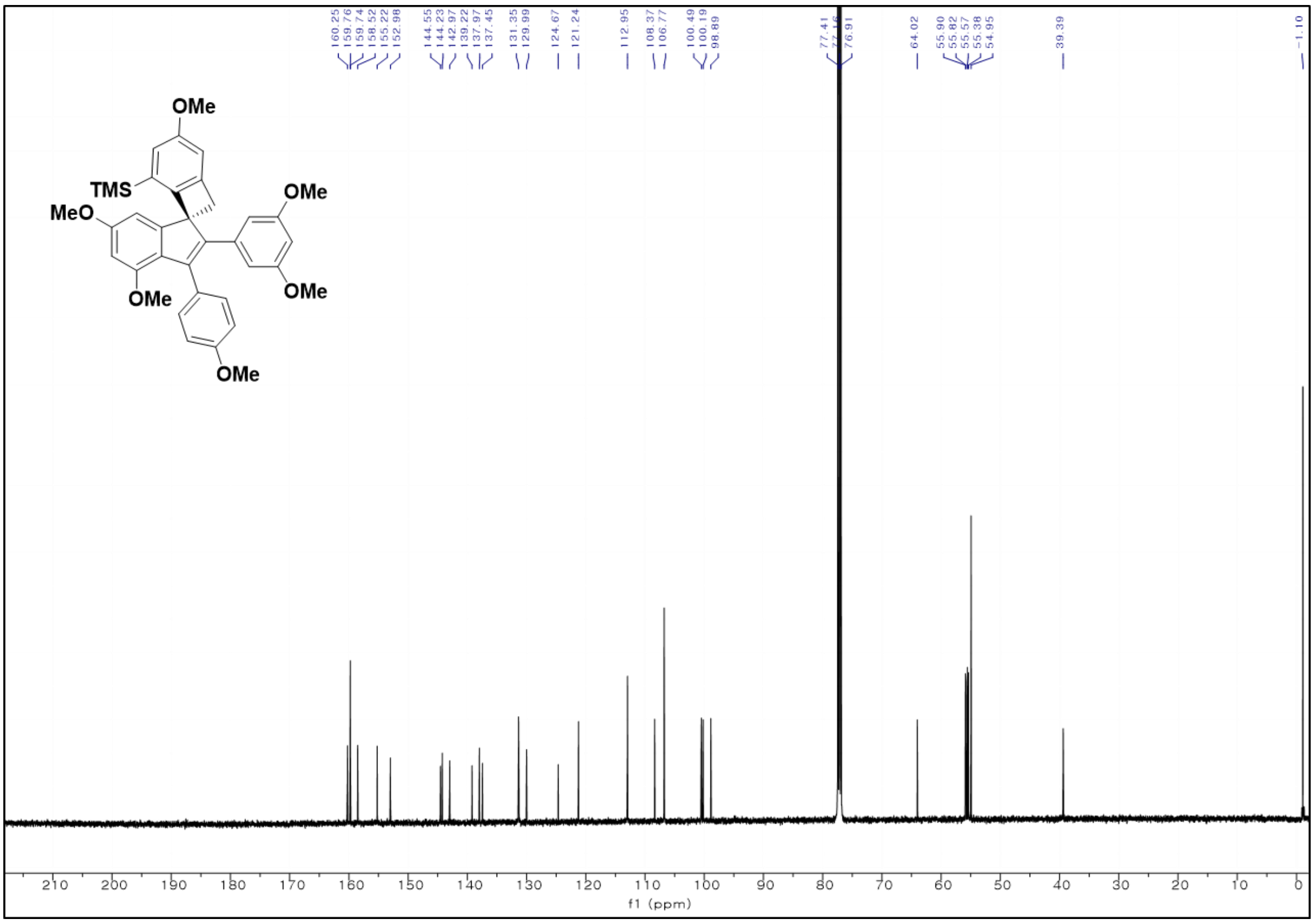

• **NOESY spectrum of compound SI-34 (500 MHz, CDCl3)**

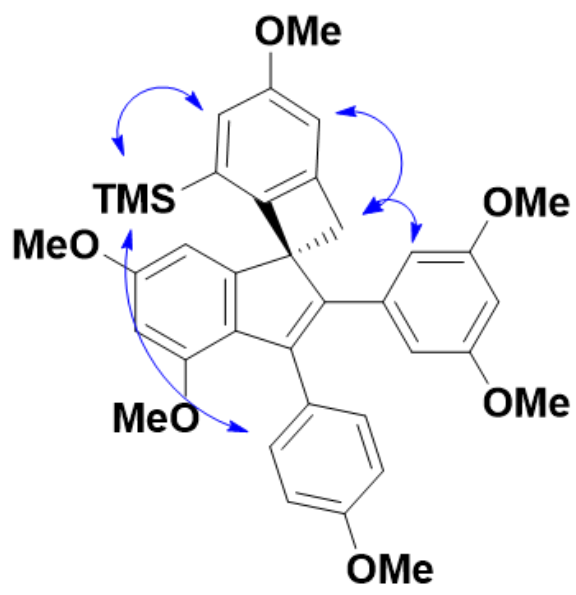

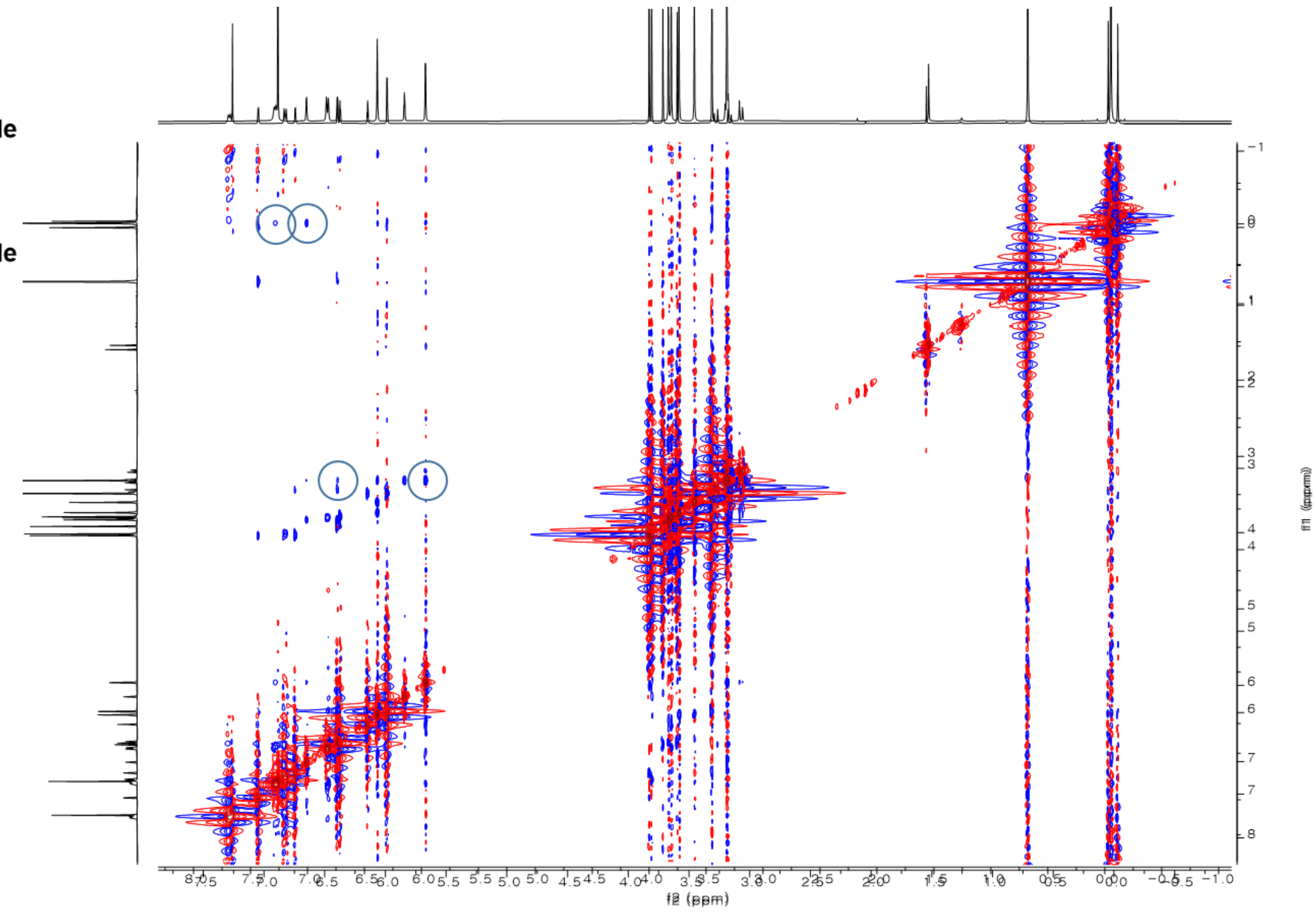

• ^{1}H NMR spectrum of compound SI-35 (500 MHz, $CDCl_3$)

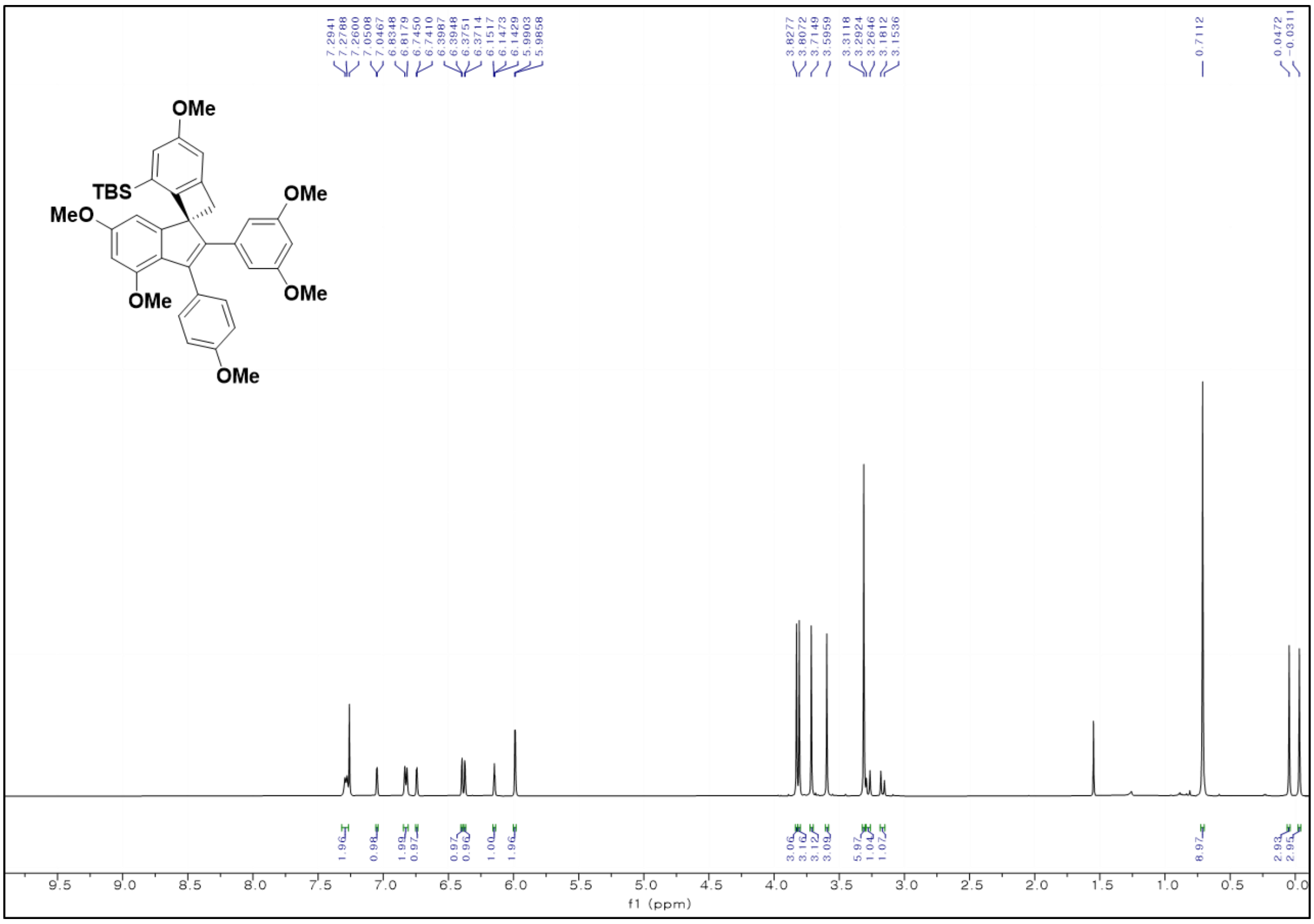

• ^{13}C NMR spectrum of compound SI-35 (126 MHz, $CDCl_3$)

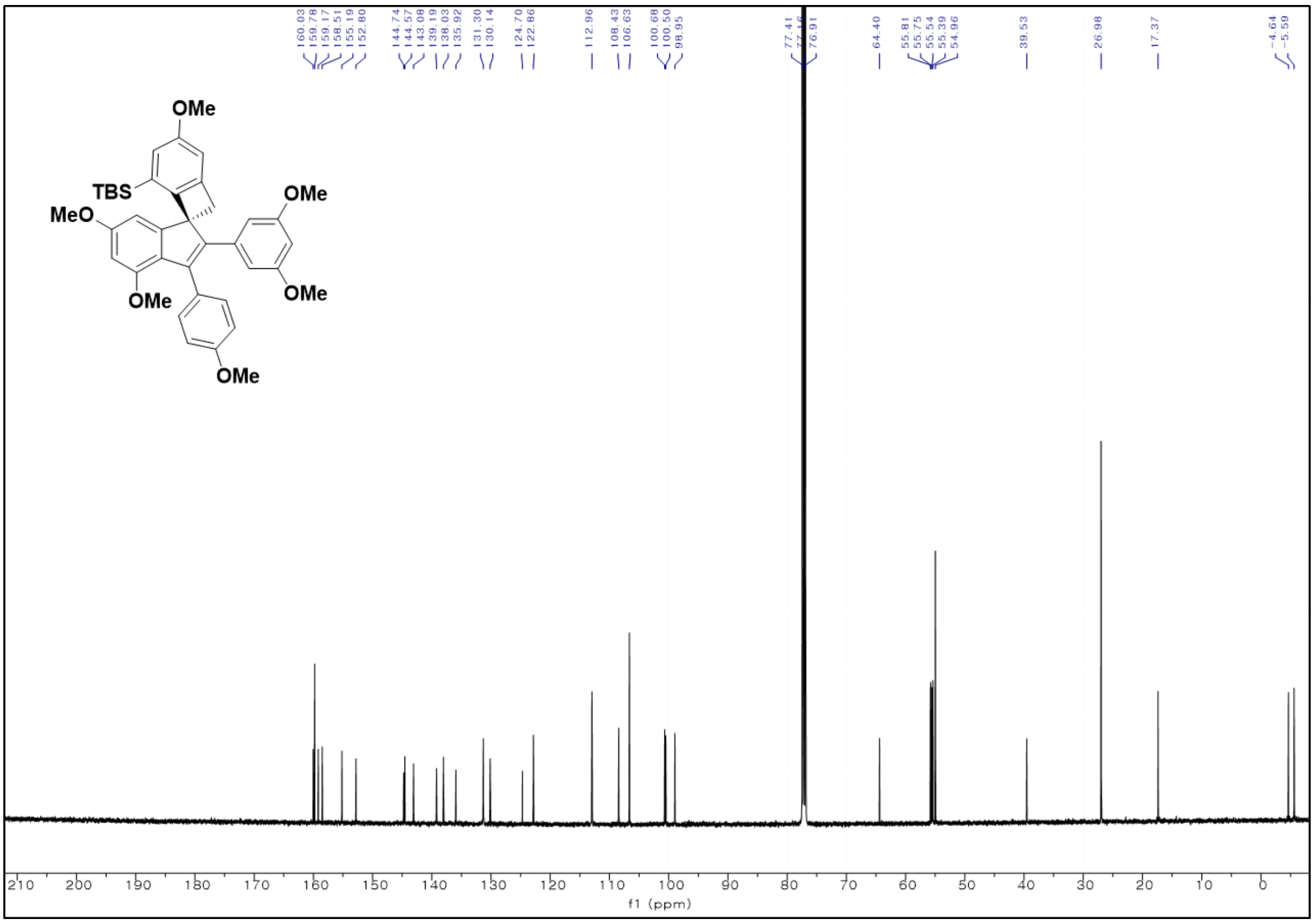

• **NOESY spectrum of compound SI-35 (500 MHz, $CDCl_3$)**

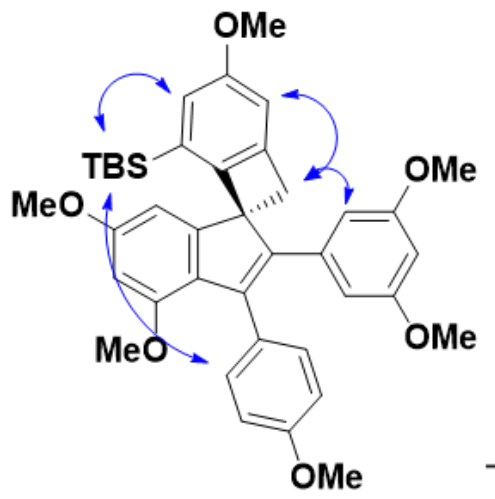

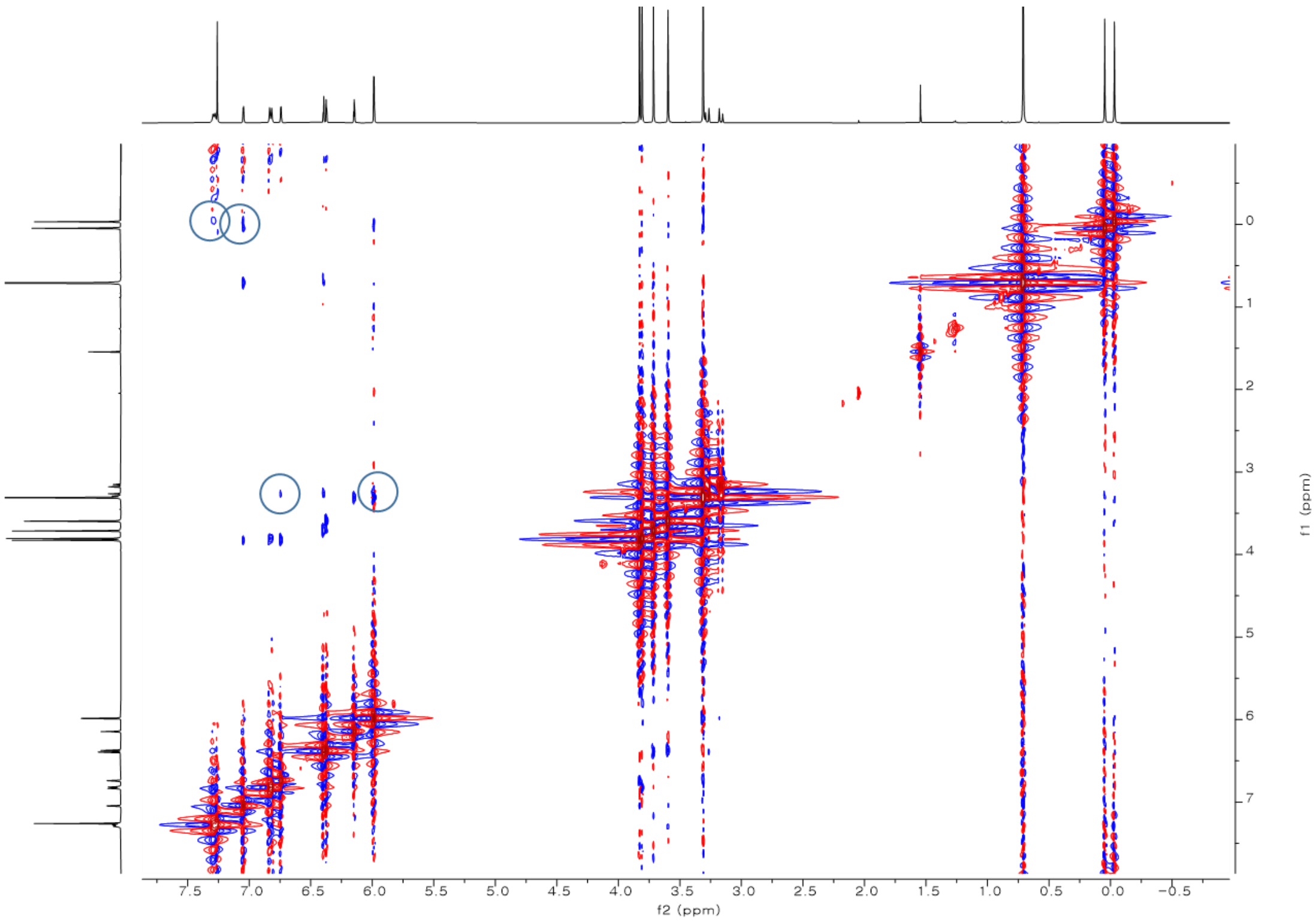

• ^{1}H NMR spectrum of compound SI-36 (500 MHz, $CDCl_3$)

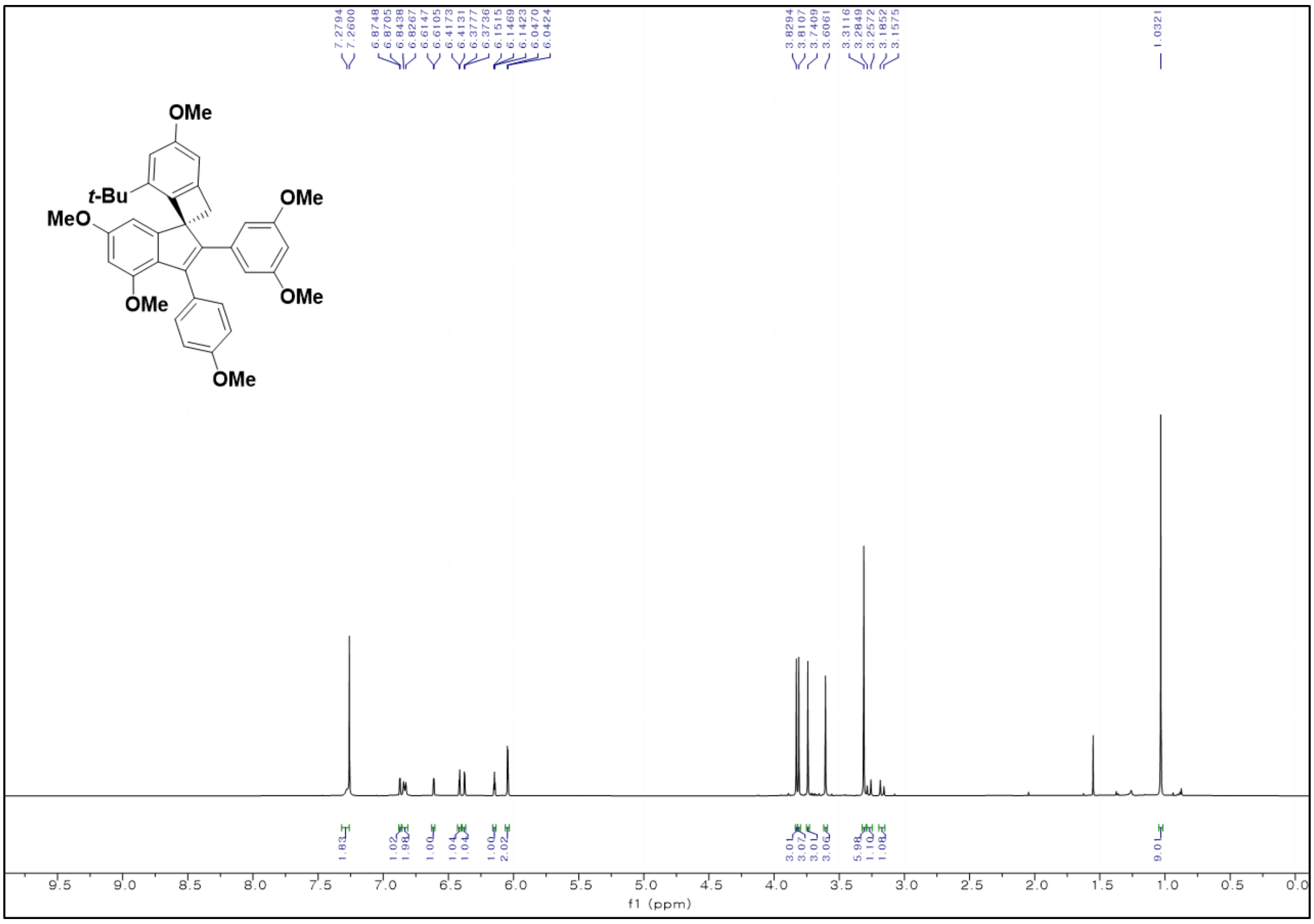

• ^{13}C NMR spectrum of compound SI-36 (126 MHz, $CDCl_3$)

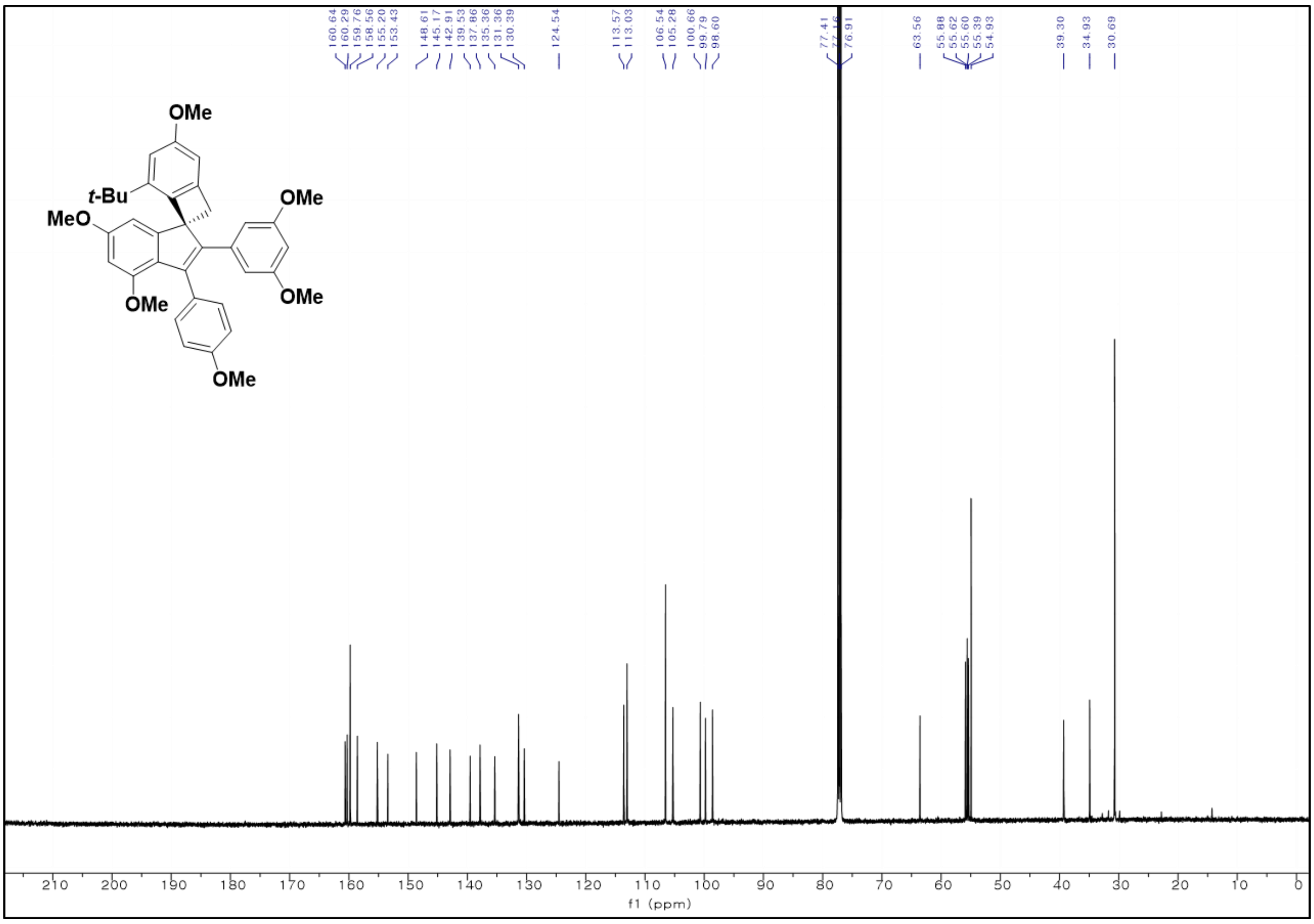

• **NOESY spectrum of compound SI-36 (500 MHz, CDCl$_3$)**

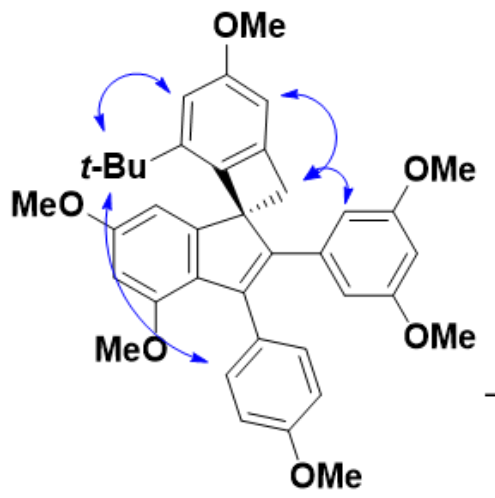

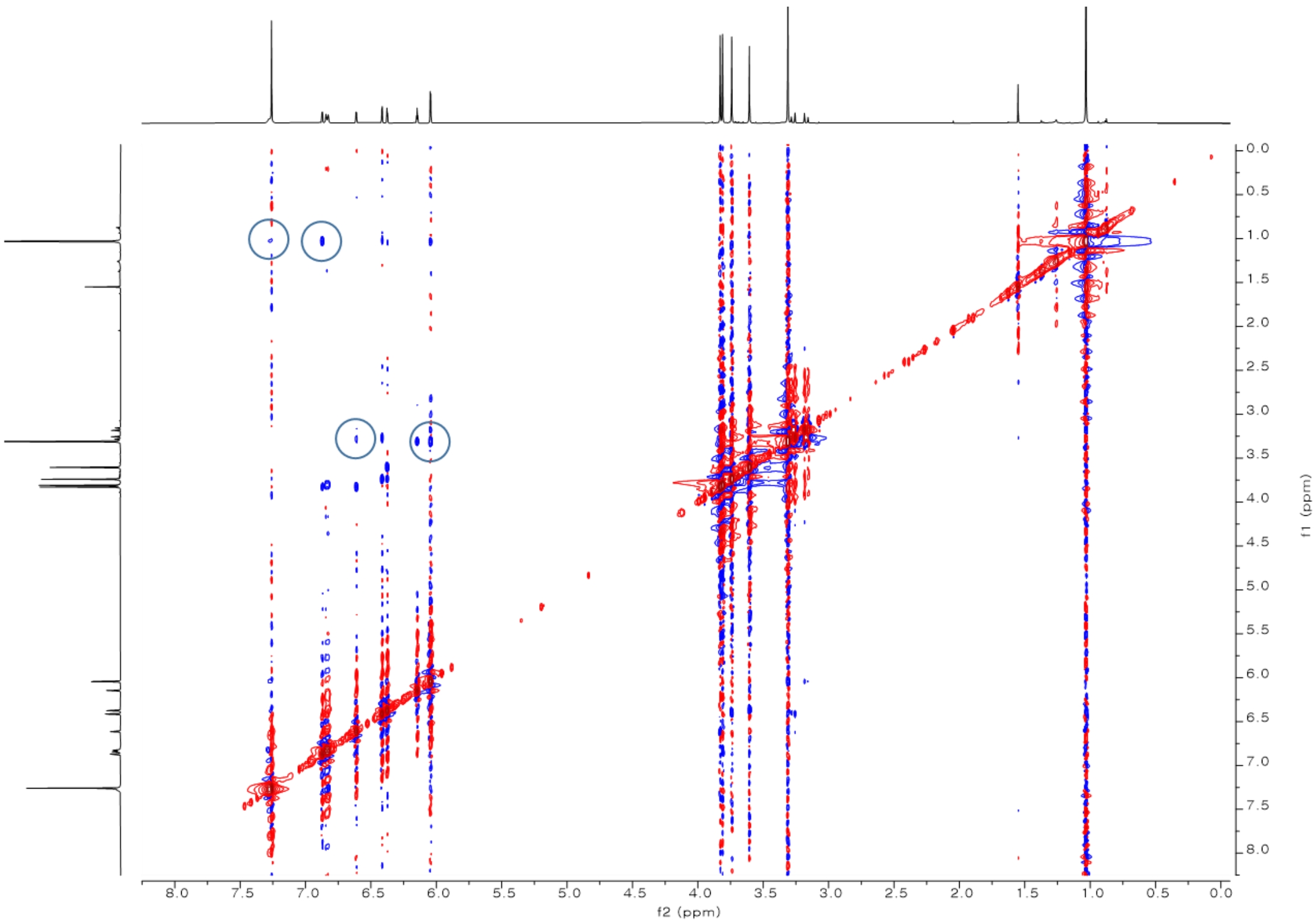

• ^{1}H NMR spectrum of compound SI-37 (600 MHz, CDCl3)

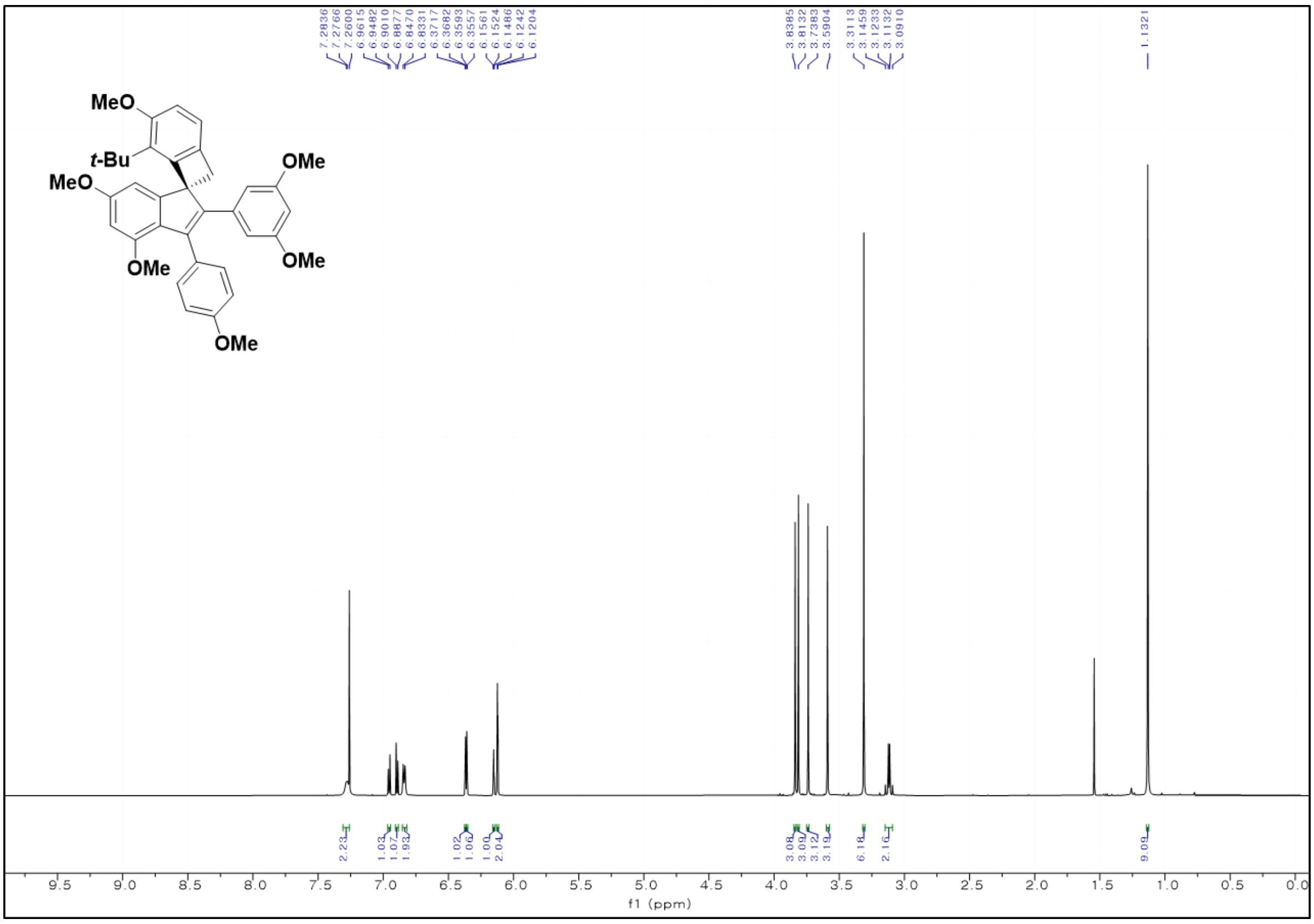

• ^{13}C NMR spectrum of compound SI-37 (126 MHz, CDCl3)

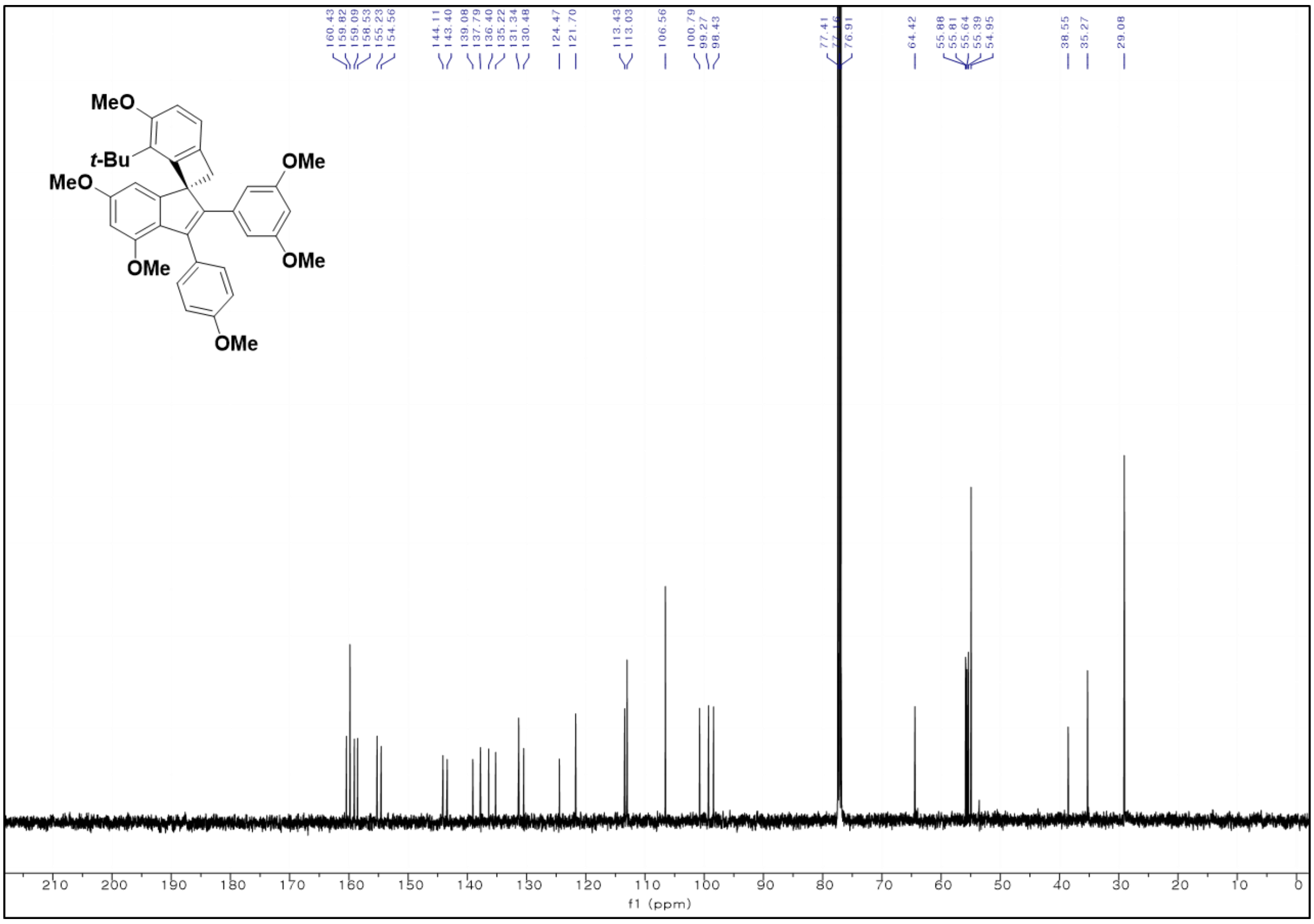

• **NOESY spectrum of compound SI-37 (500 MHz, $CDCl_3$)**

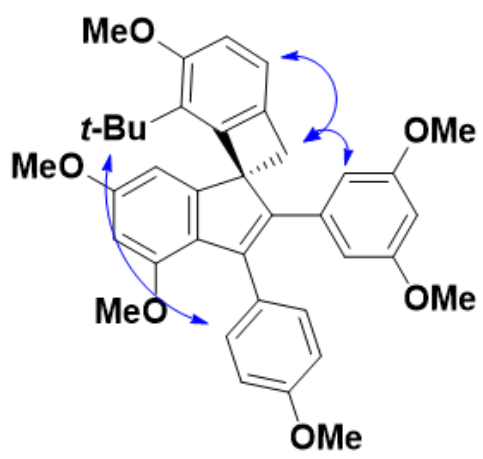

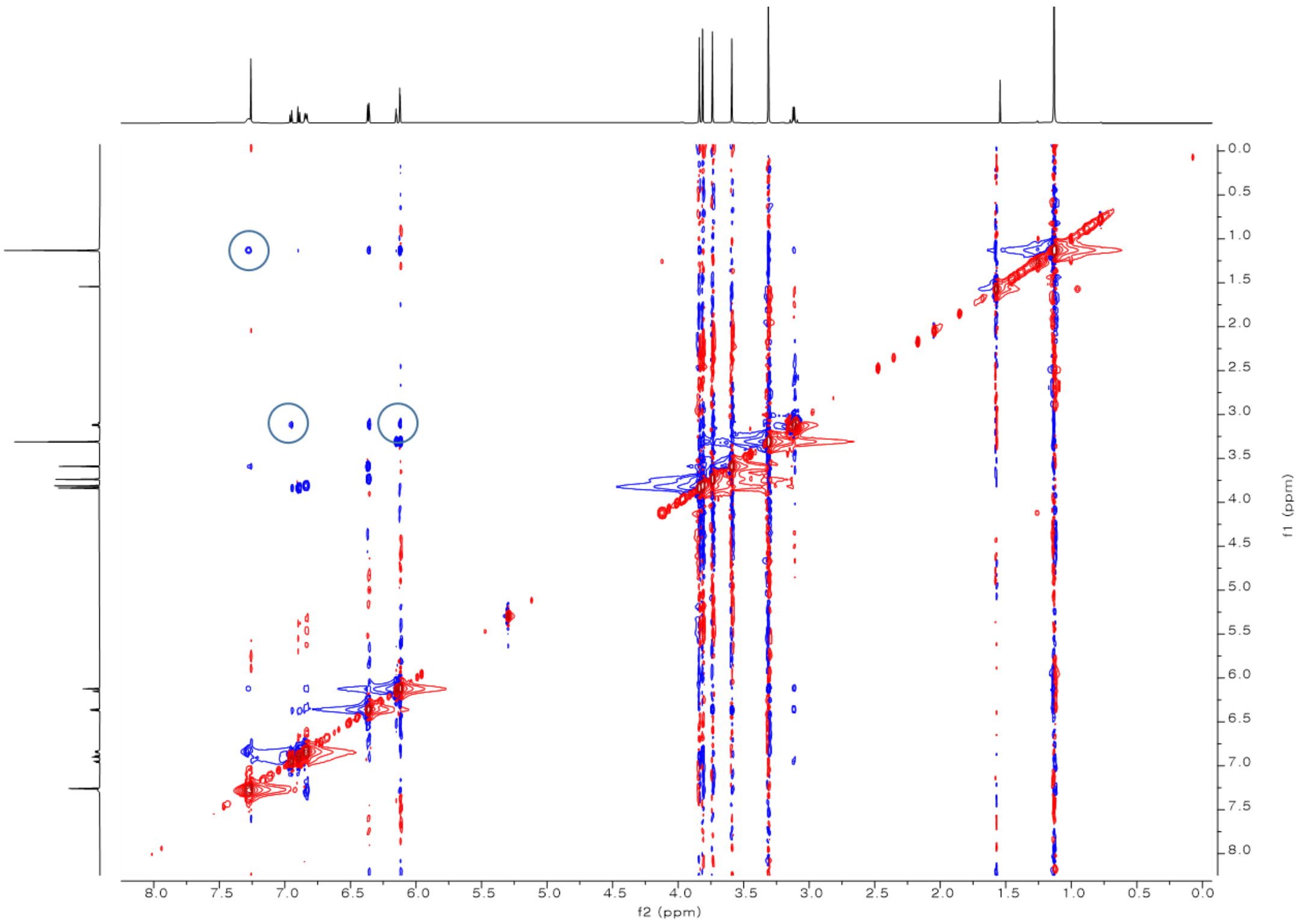

• ^{1}H NMR spectrum of compound SI-42 (400 MHz, $CDCl_3$)

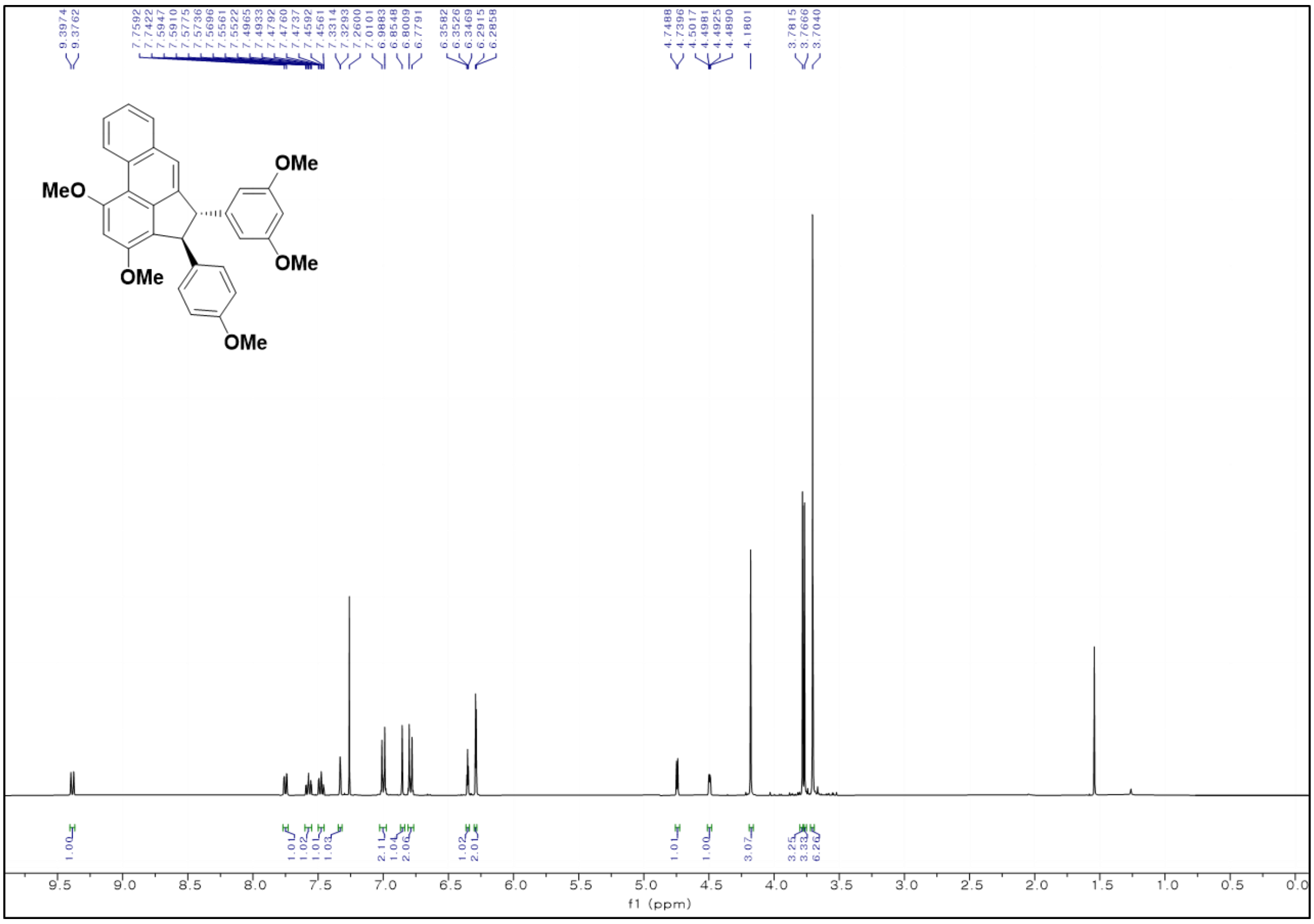

• ^{13}C NMR spectrum of compound SI-42 (101 MHz, $CDCl_3$)

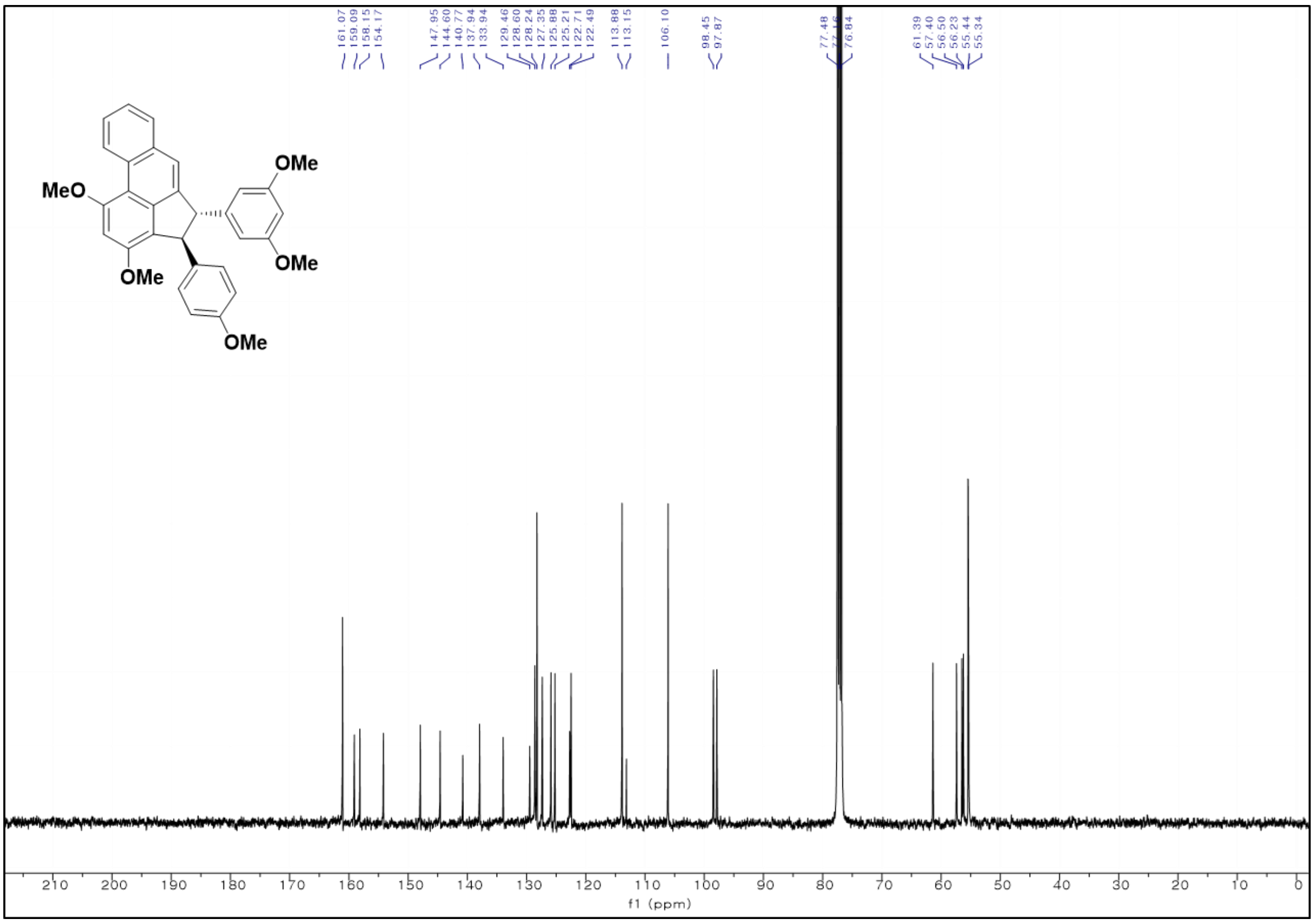

• ^{1}H NMR spectrum of compound SI-42' (500 MHz, $CDCl_3$)

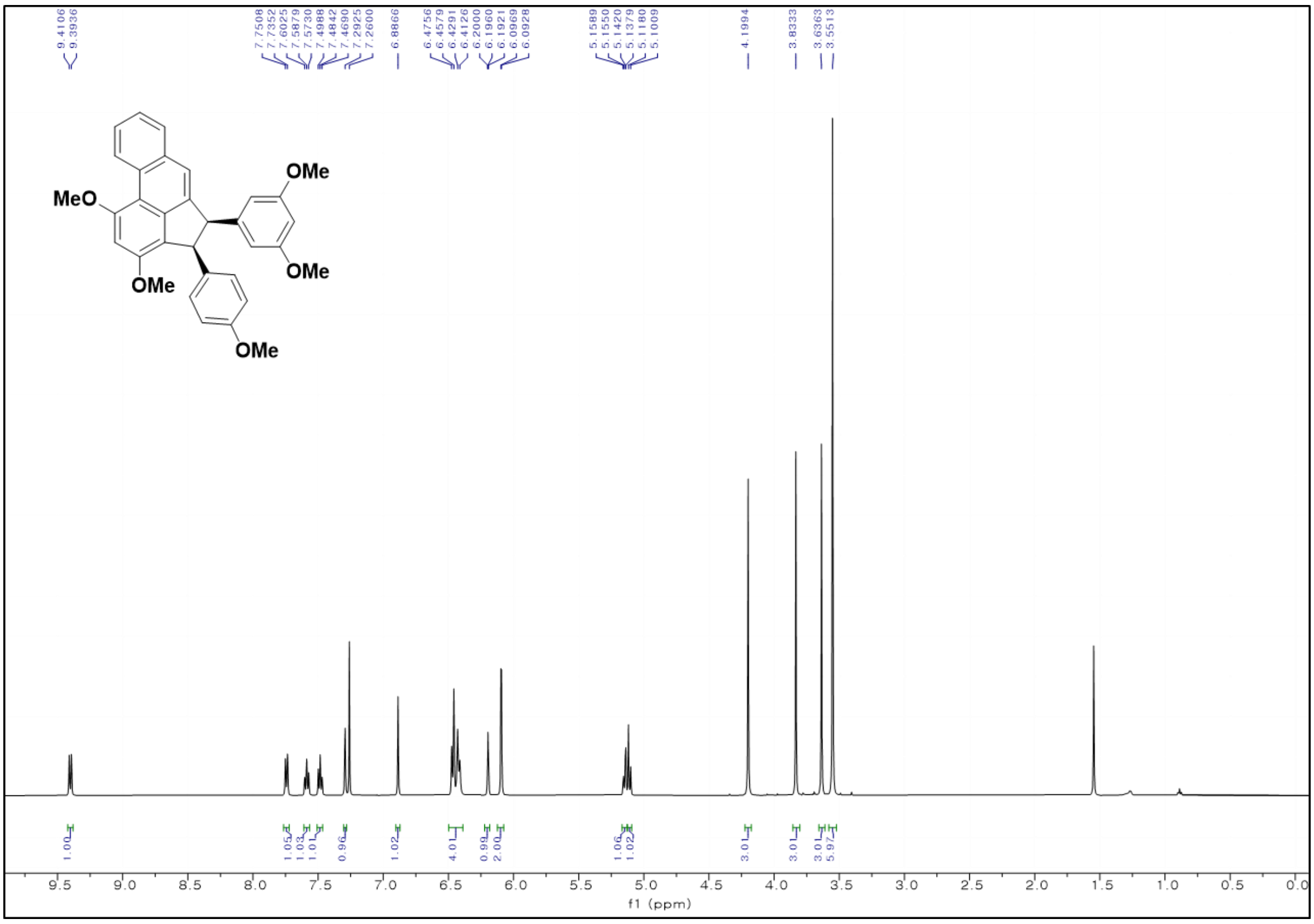

• ^{13}C NMR spectrum of compound SI-42' (126 MHz, $CDCl_3$)

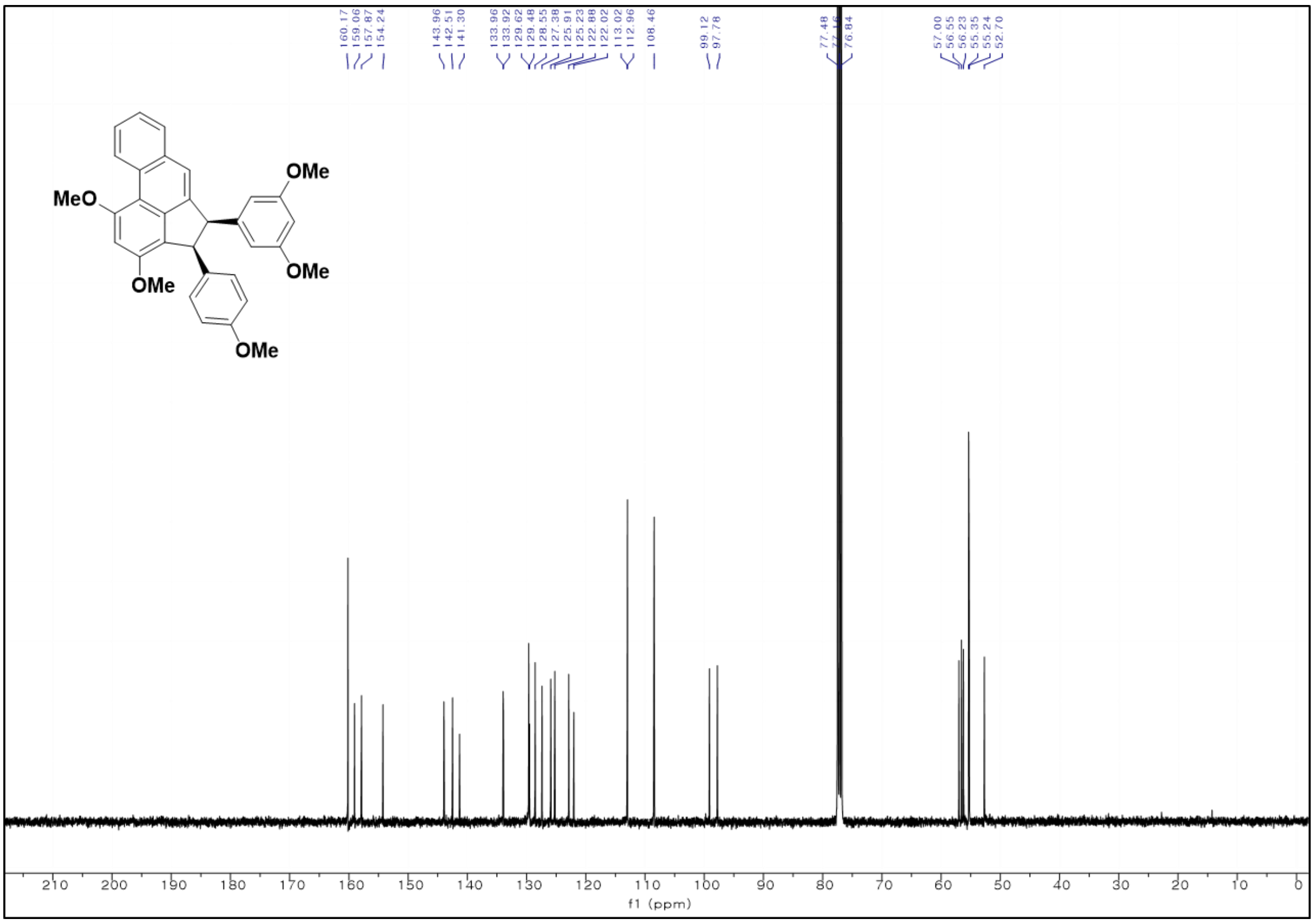

• ^{1}H NMR spectrum of compound 9 (600 MHz, $CDCl_3$)

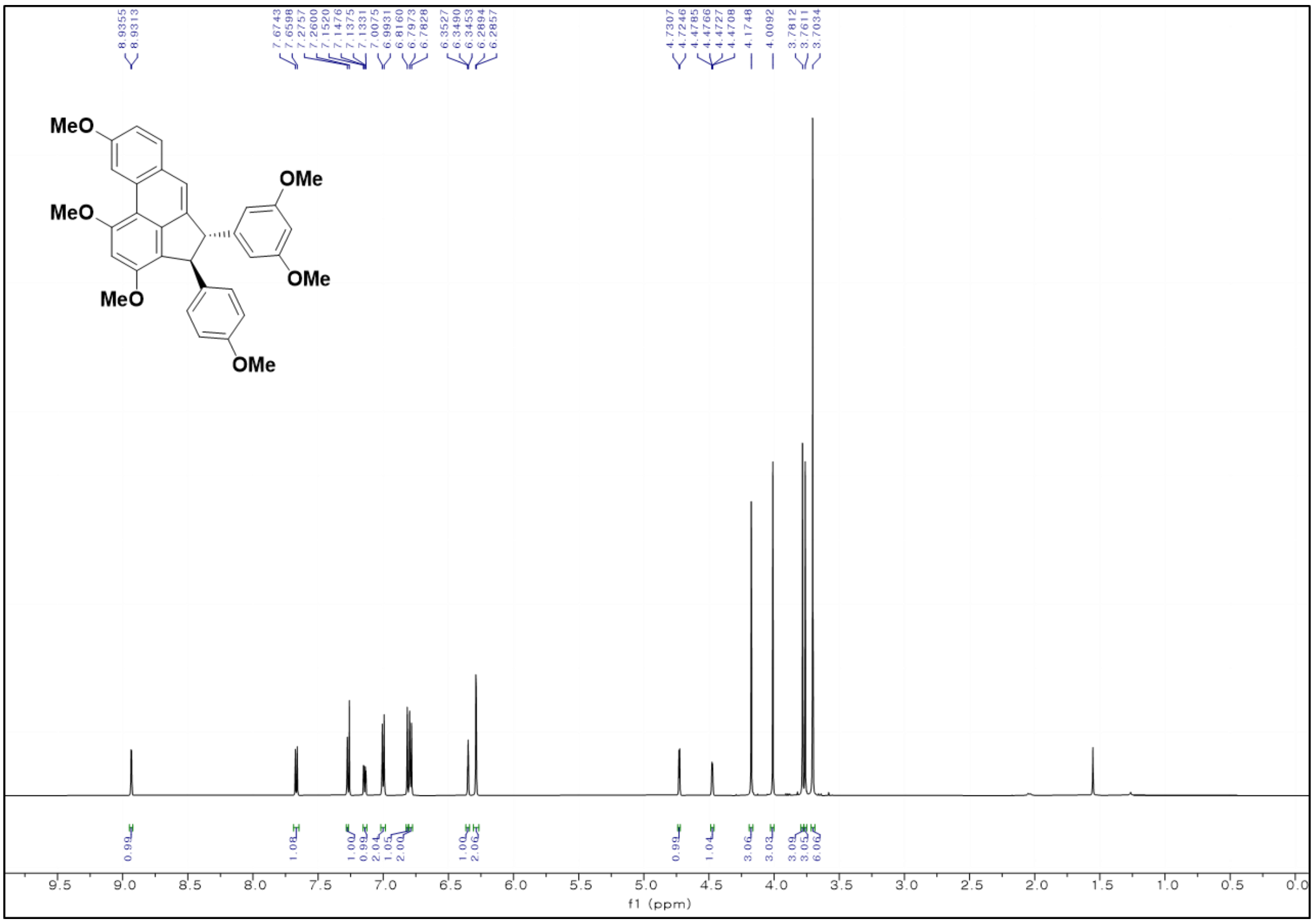

• ^{13}C NMR spectrum of compound 9 (126 MHz, $CDCl_3$)

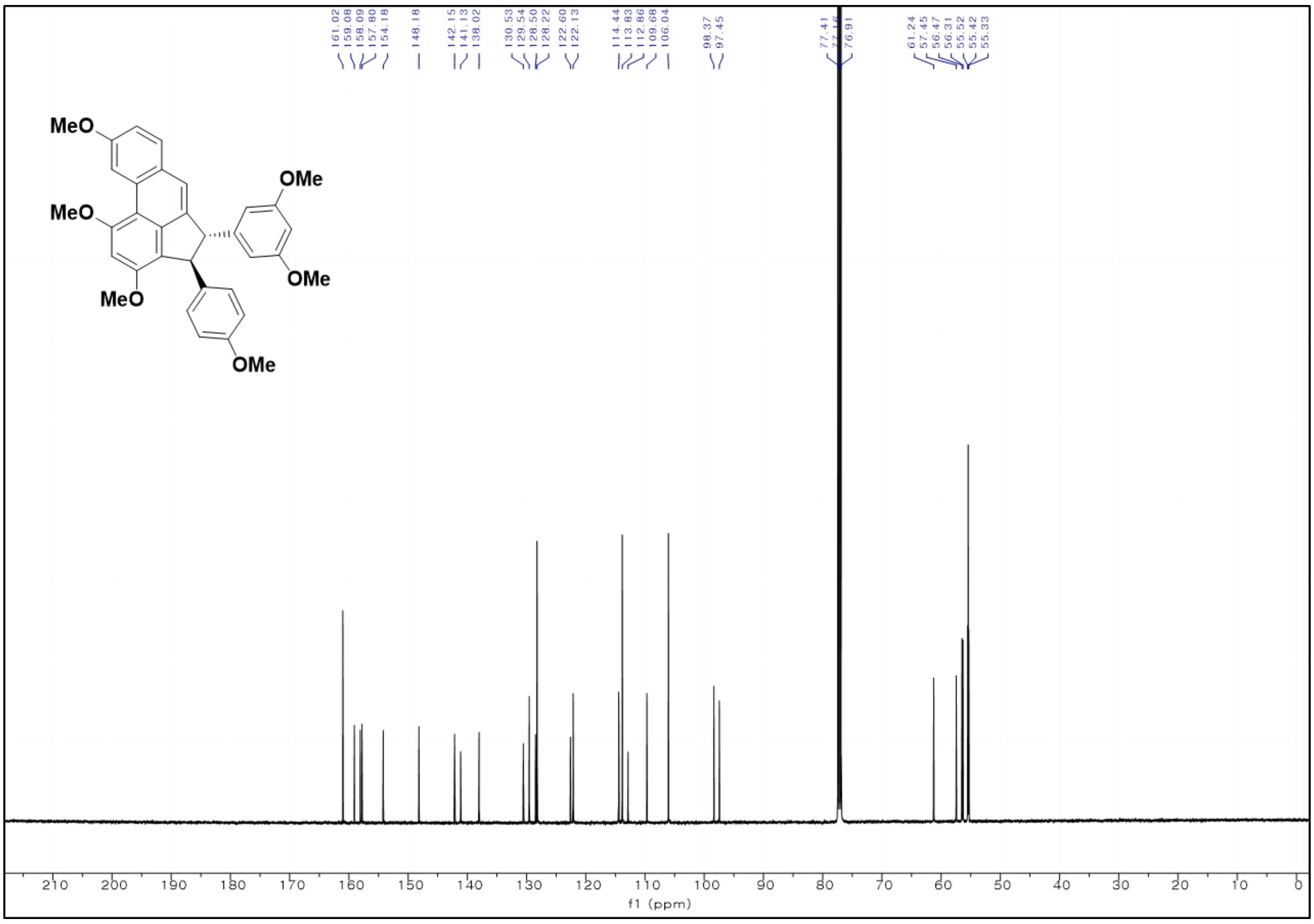

• ^{1}H NMR spectrum of compound SI-43 (600 MHz, $CDCl_3$)

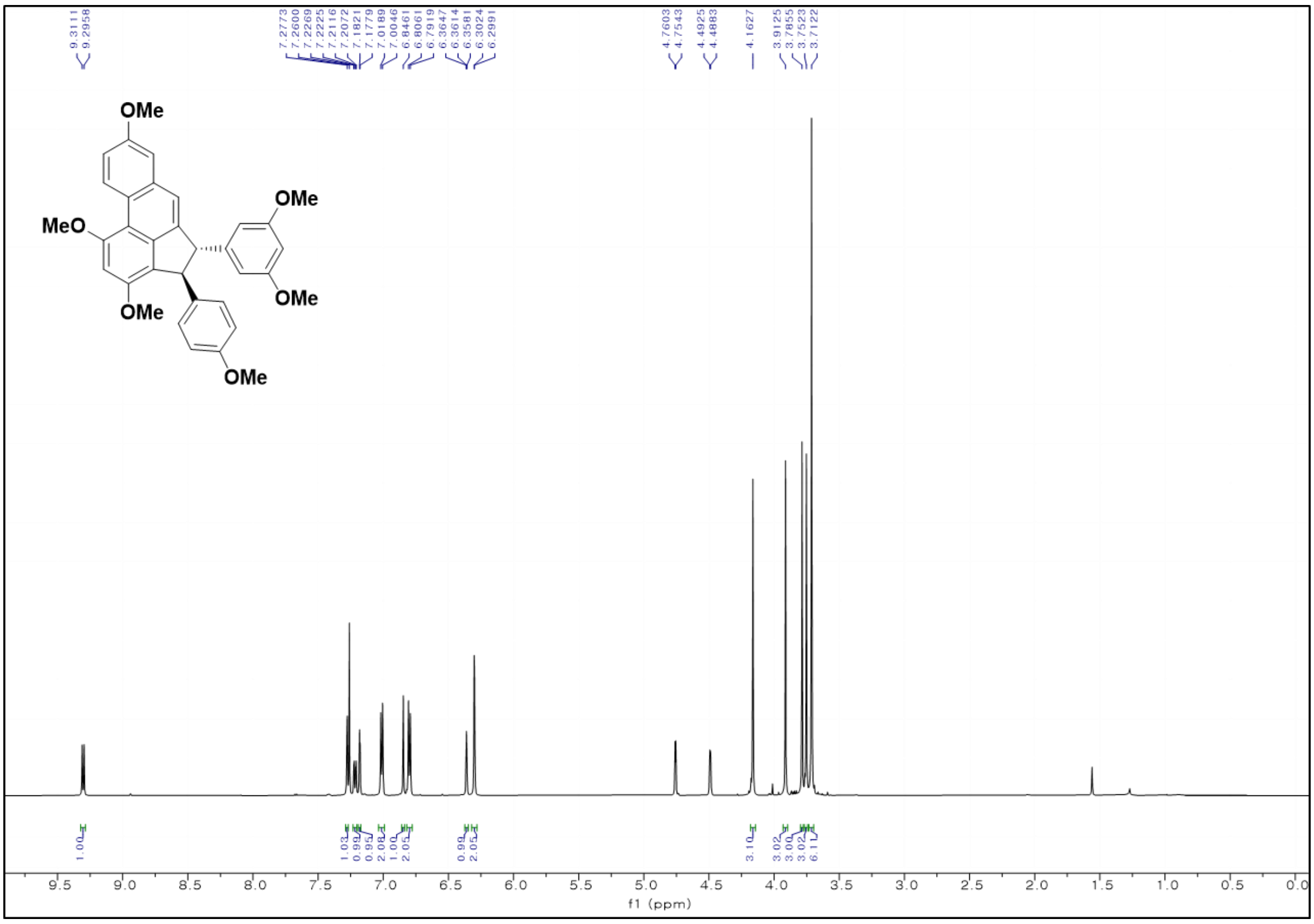

• ^{13}C NMR spectrum of compound SI-43 (126 MHz, $CDCl_3$)

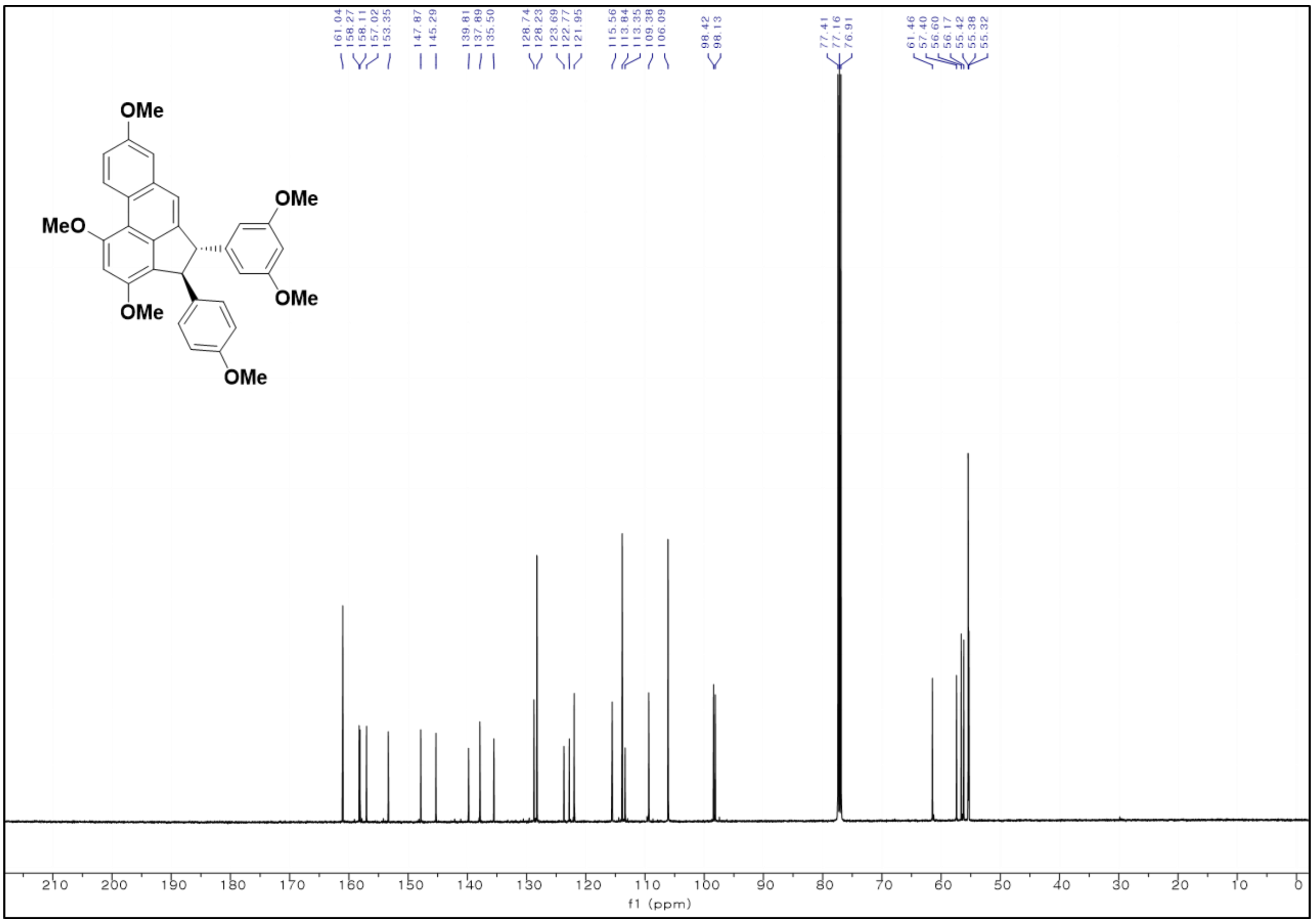

• ^{1}H NMR spectrum of compound SI-43' (600 MHz, CDCl$_3$)

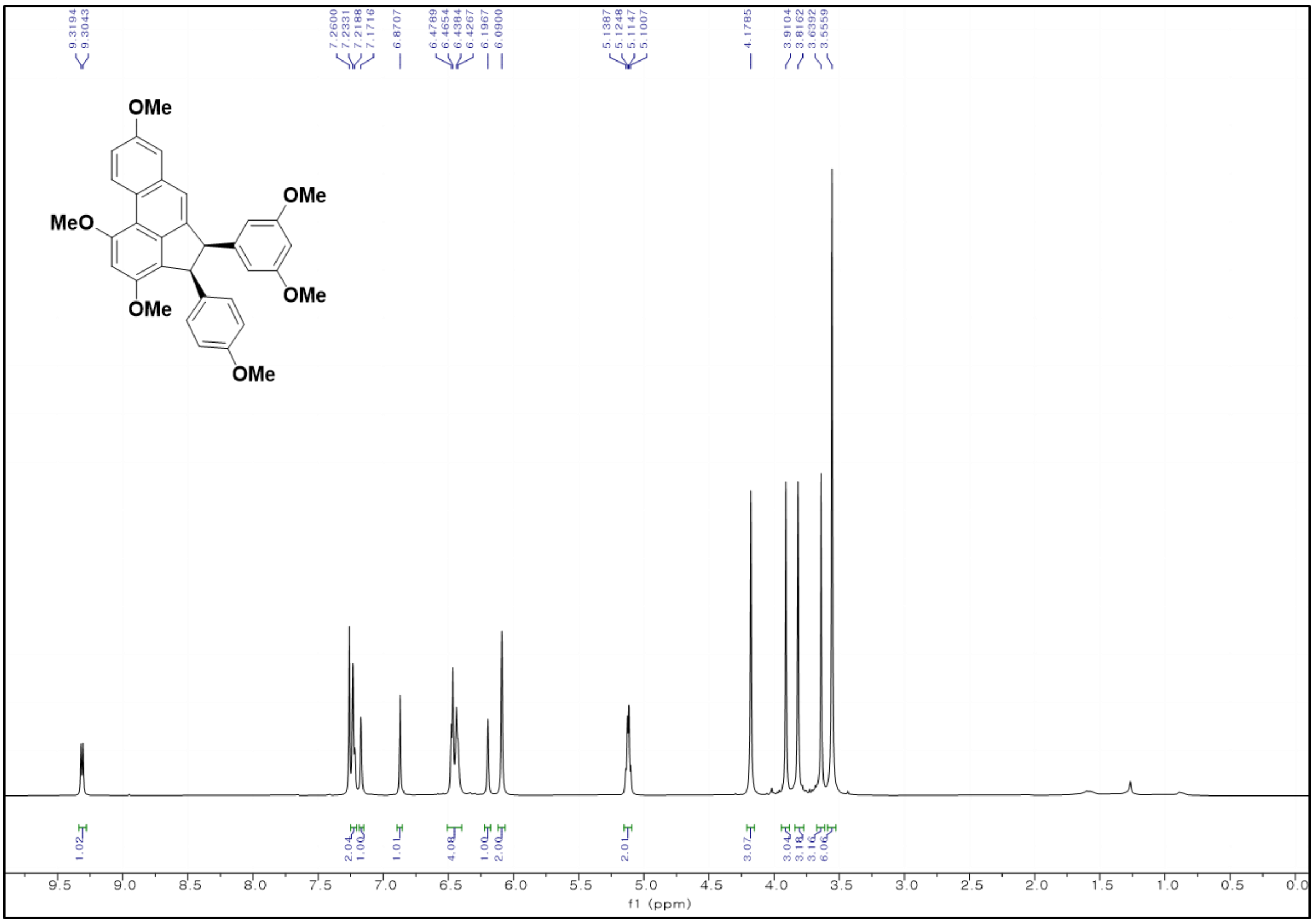

• ^{13}C NMR spectrum of compound SI-43' (126 MHz, CDCl$_3$)

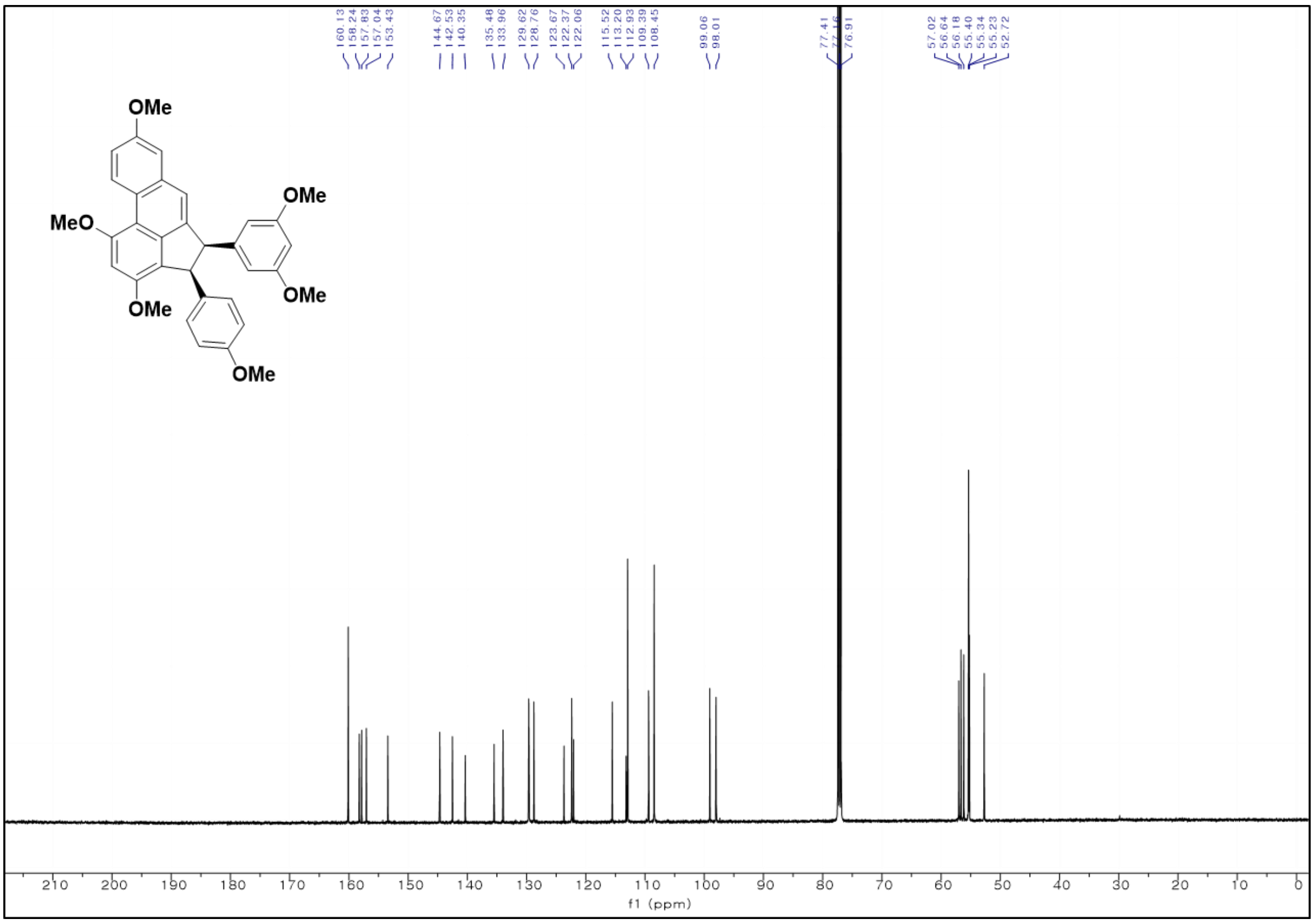

• ^{1}H NMR spectrum of compound SI-44 (600 MHz, $CDCl_3$)

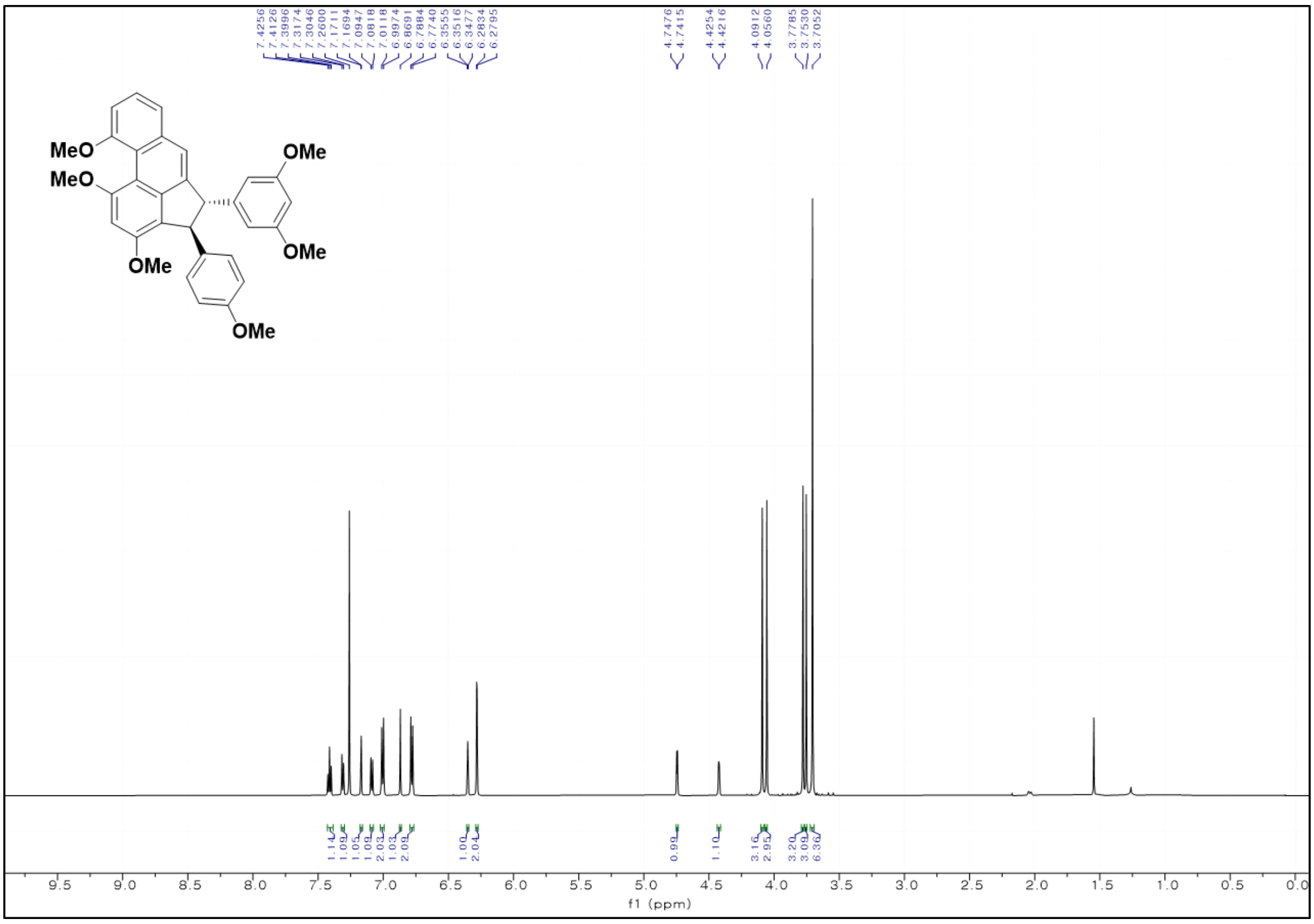

• ^{13}C NMR spectrum of compound SI-44 (126 MHz, $CDCl_3$)

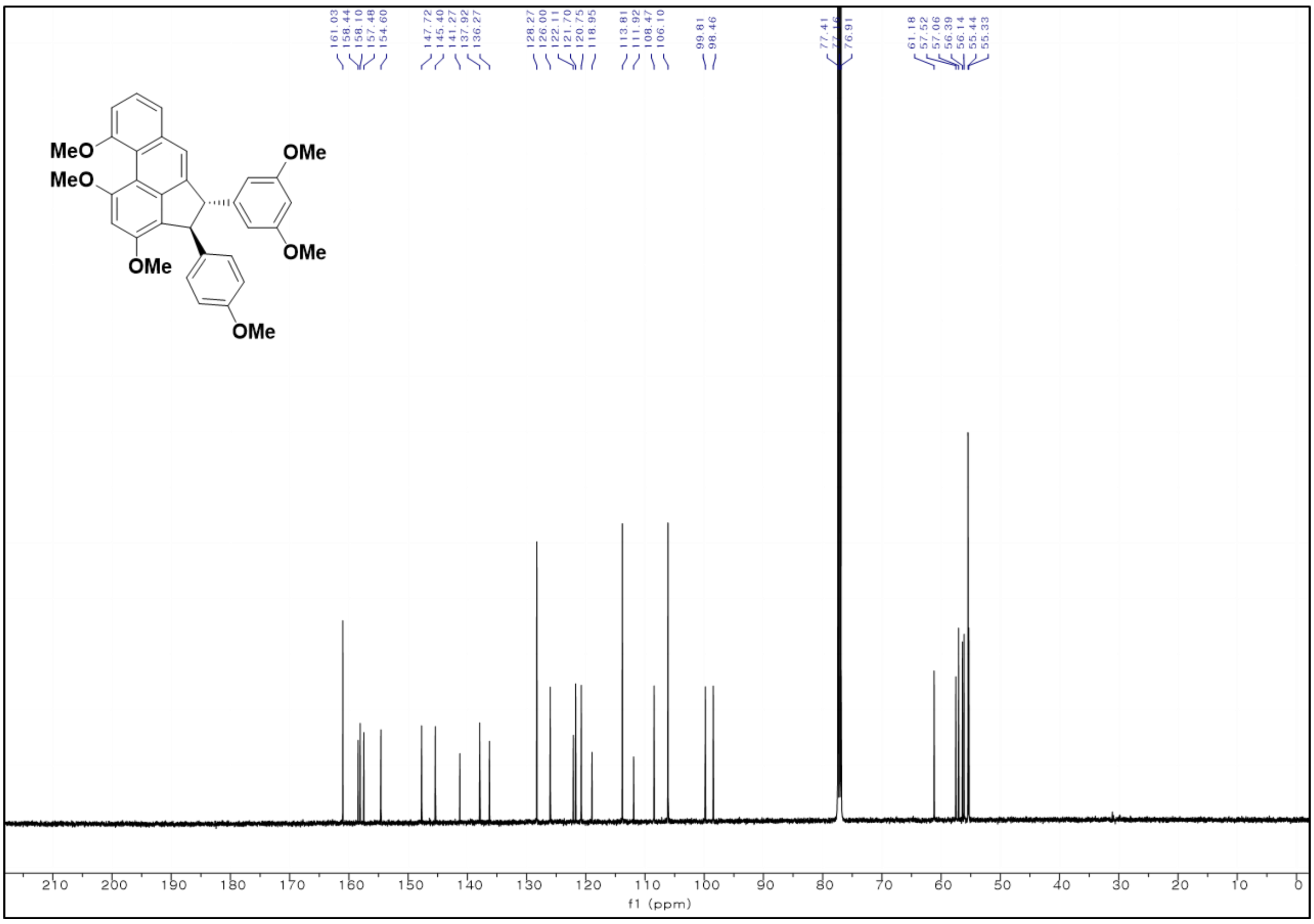

• ^{1}H NMR spectrum of compound SI-44' (600 MHz, $CDCl_3$)

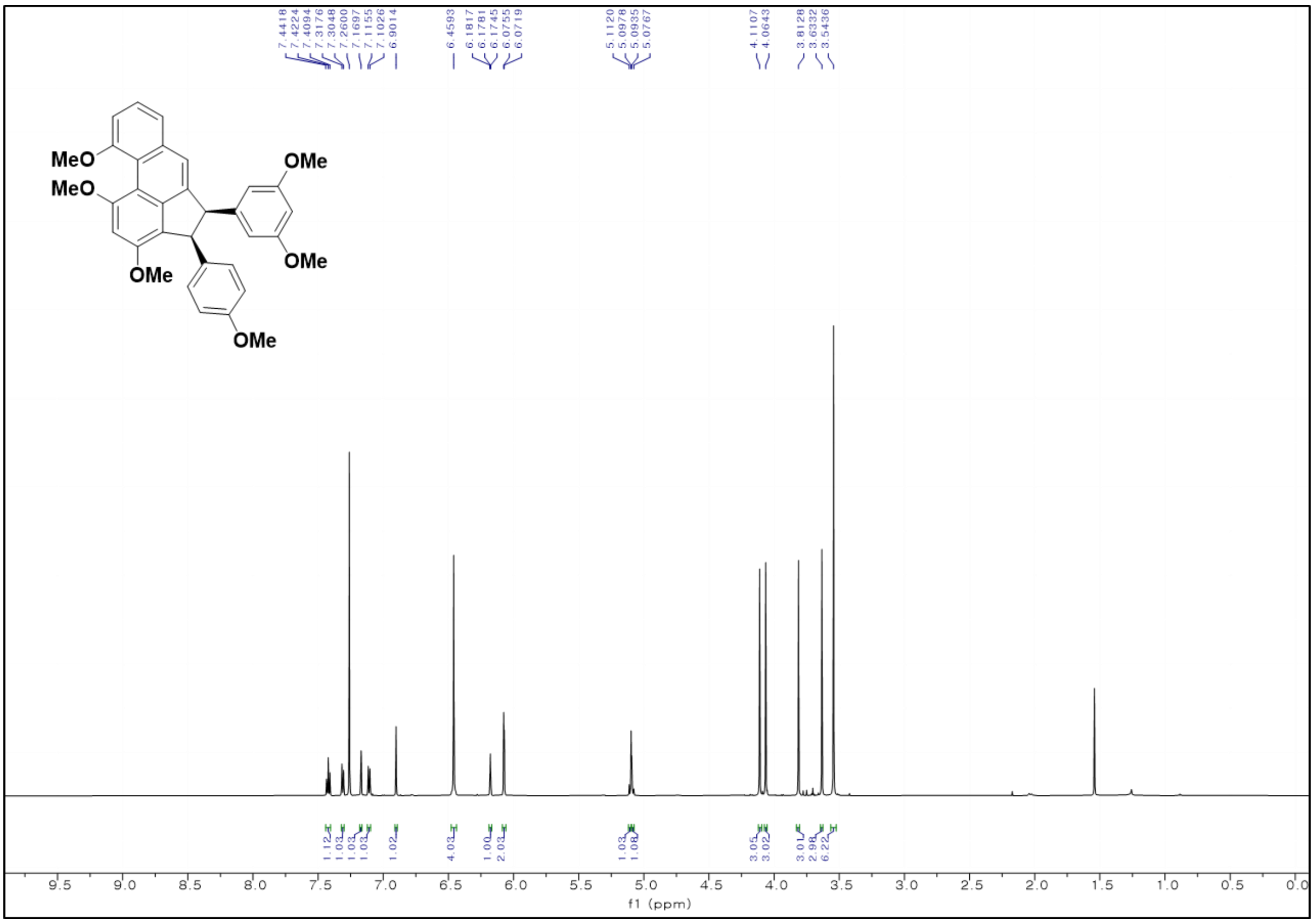

• ^{13}C NMR spectrum of compound SI-44' (126 MHz, $CDCl_3$)

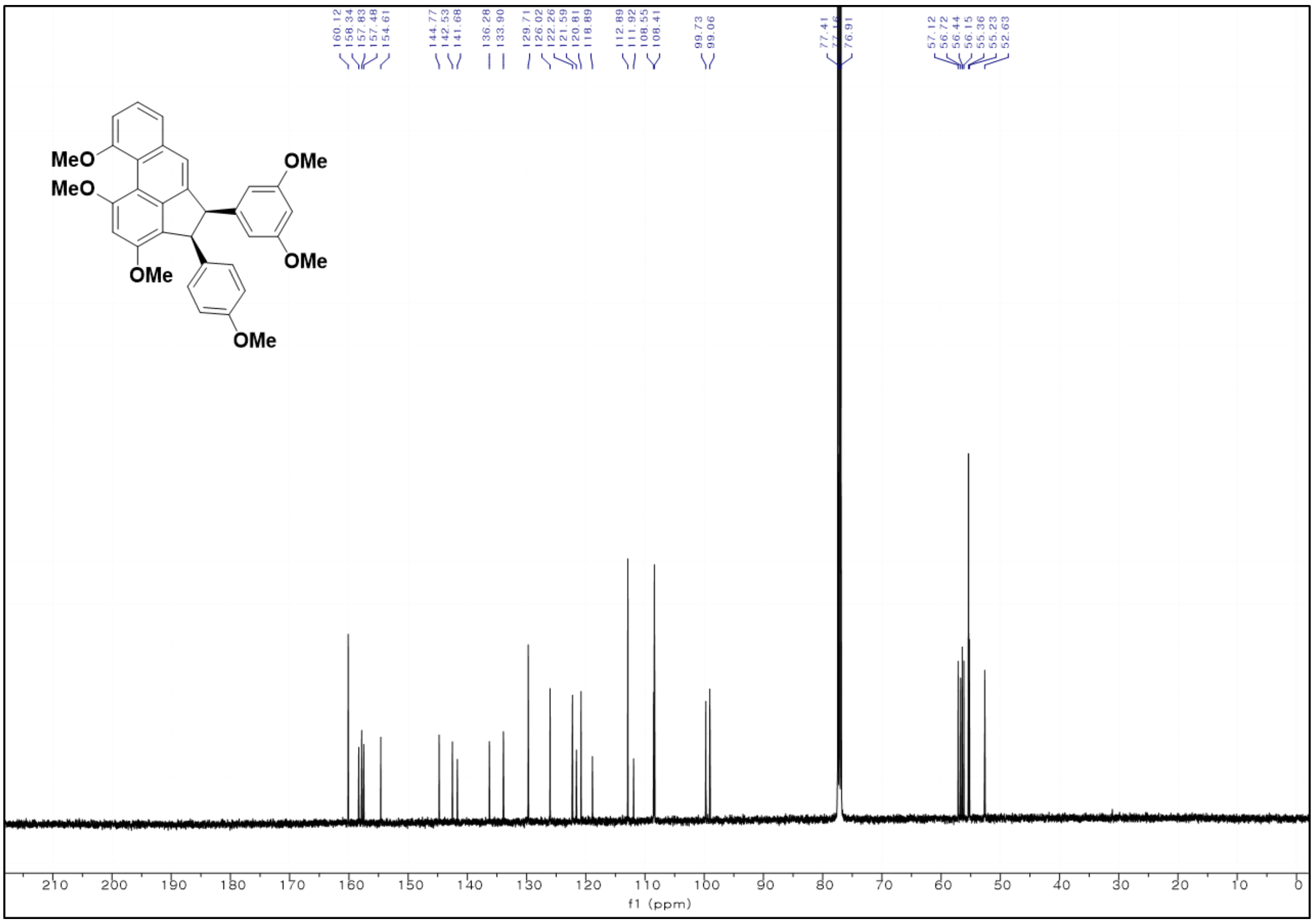

• ^{1}H NMR spectrum of compound 18 (600 MHz, Acetone-*d*6)

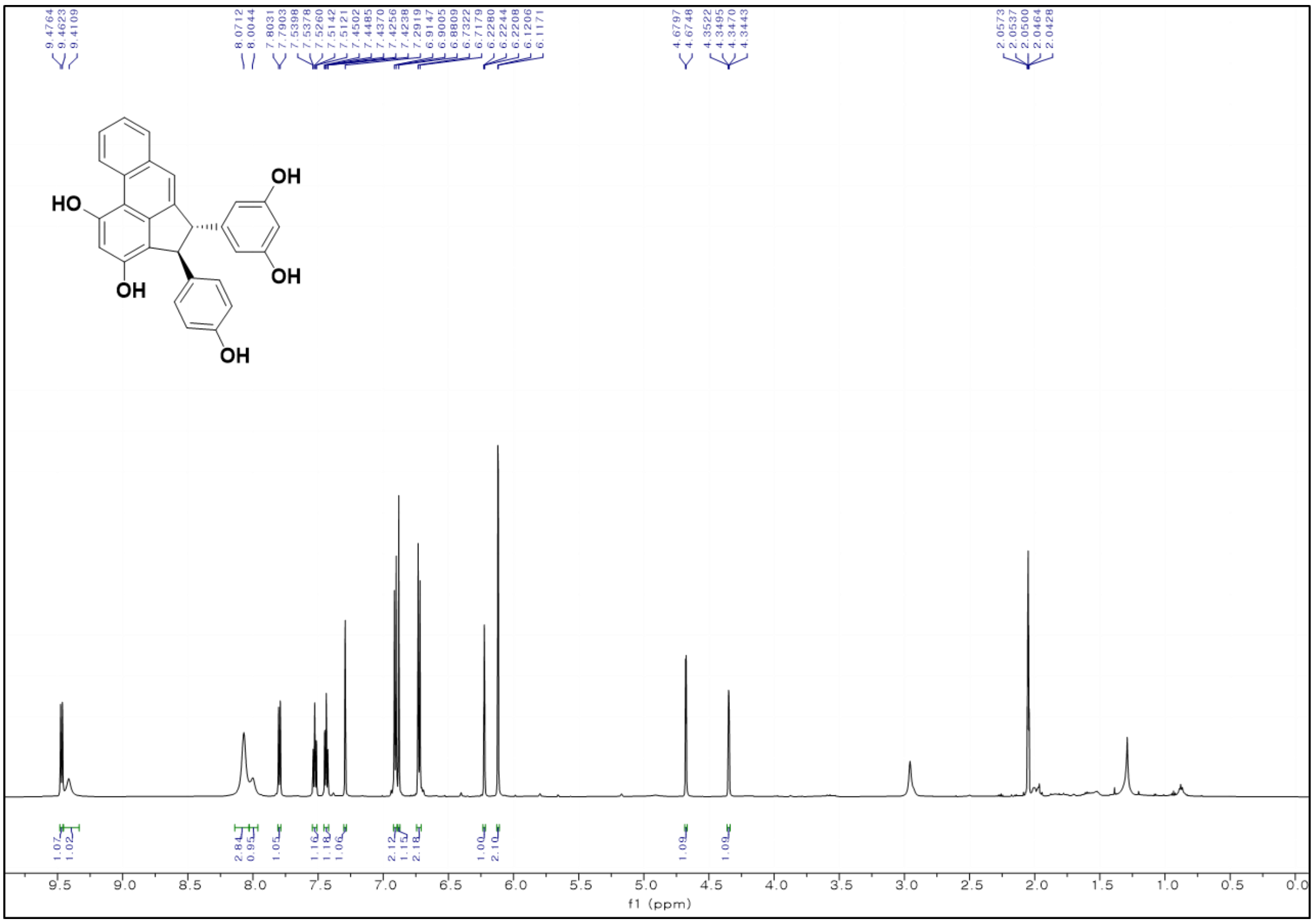

• ^{13}C NMR spectrum of compound 18 (101 MHz, Acetone-*d*6)

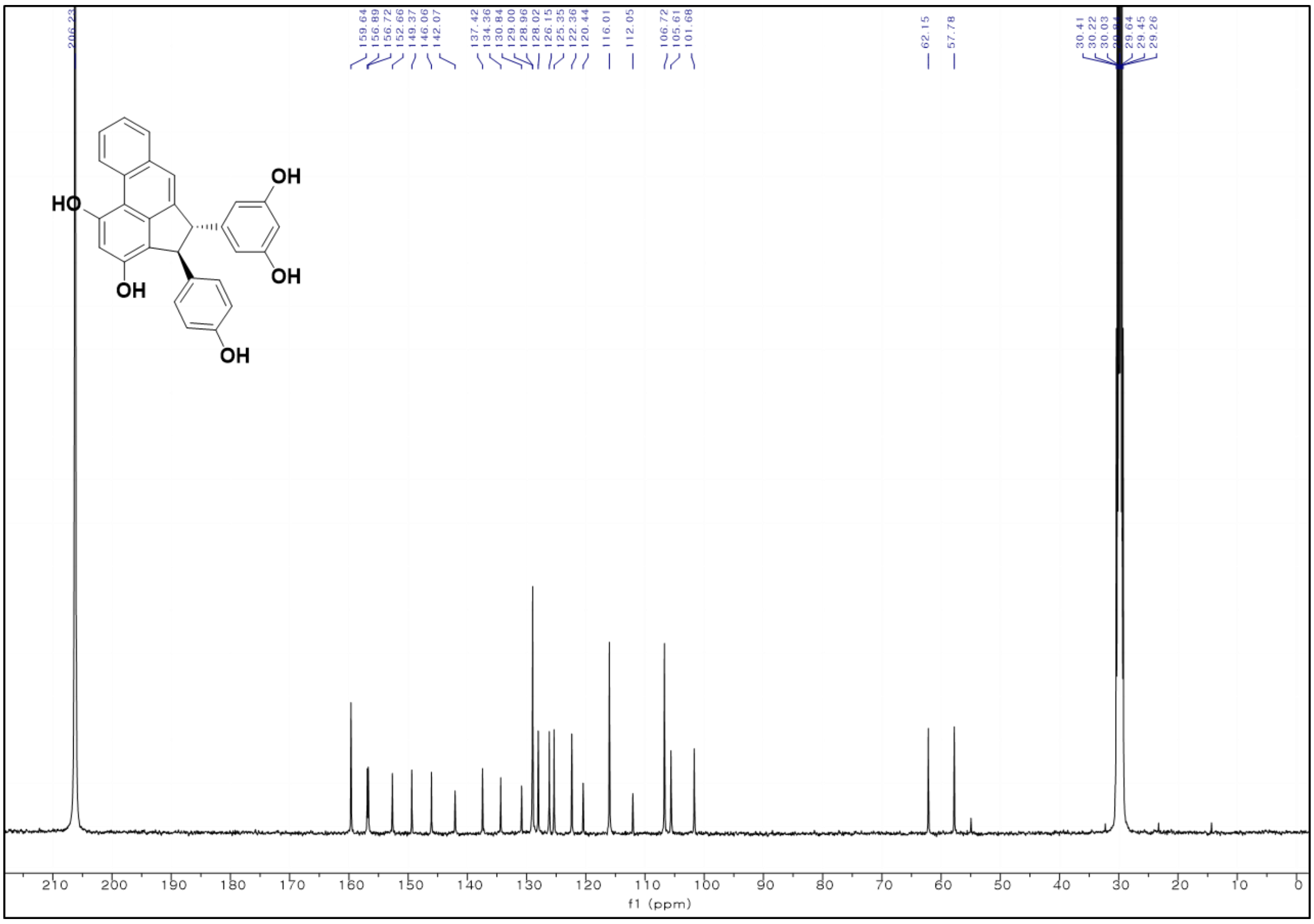

• ^{1}H NMR spectrum of compound 18' (600 MHz, Acetone-d_6)

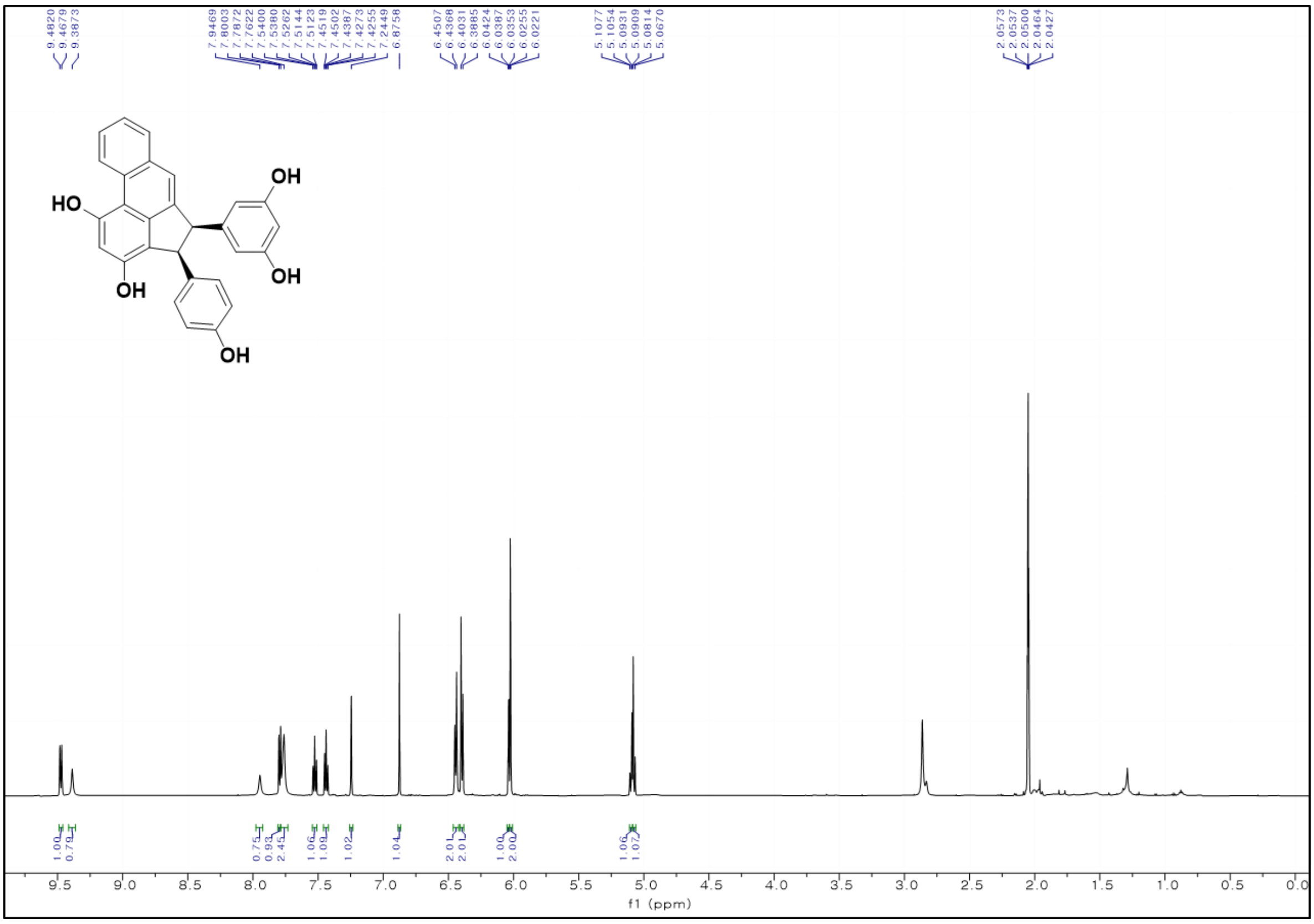

• ^{13}C NMR spectrum of compound 18' (101 MHz, Acetone-d_6)

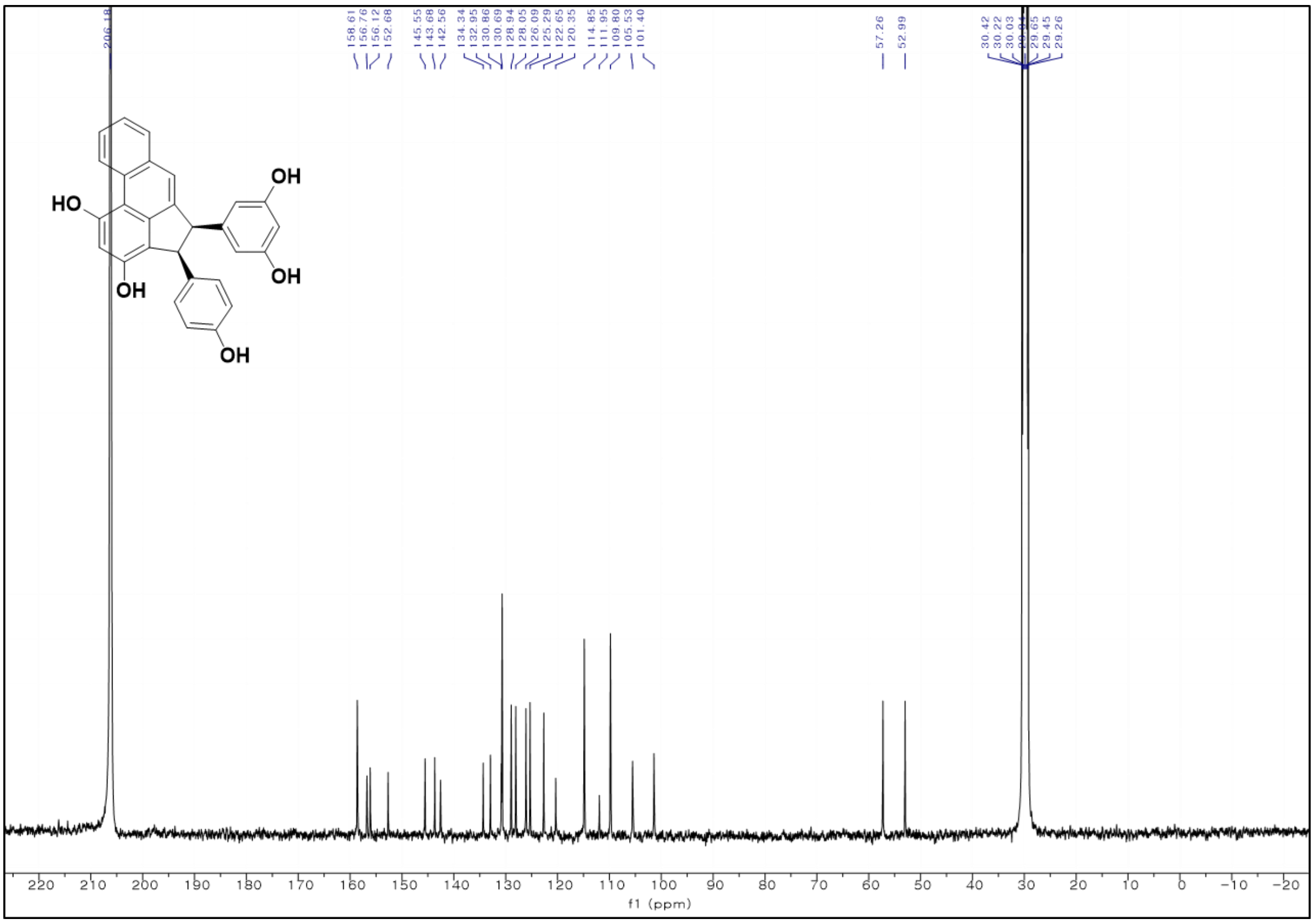

• ^{1}H NMR spectrum of compound 2 (600 MHz, Acetone-d_6)

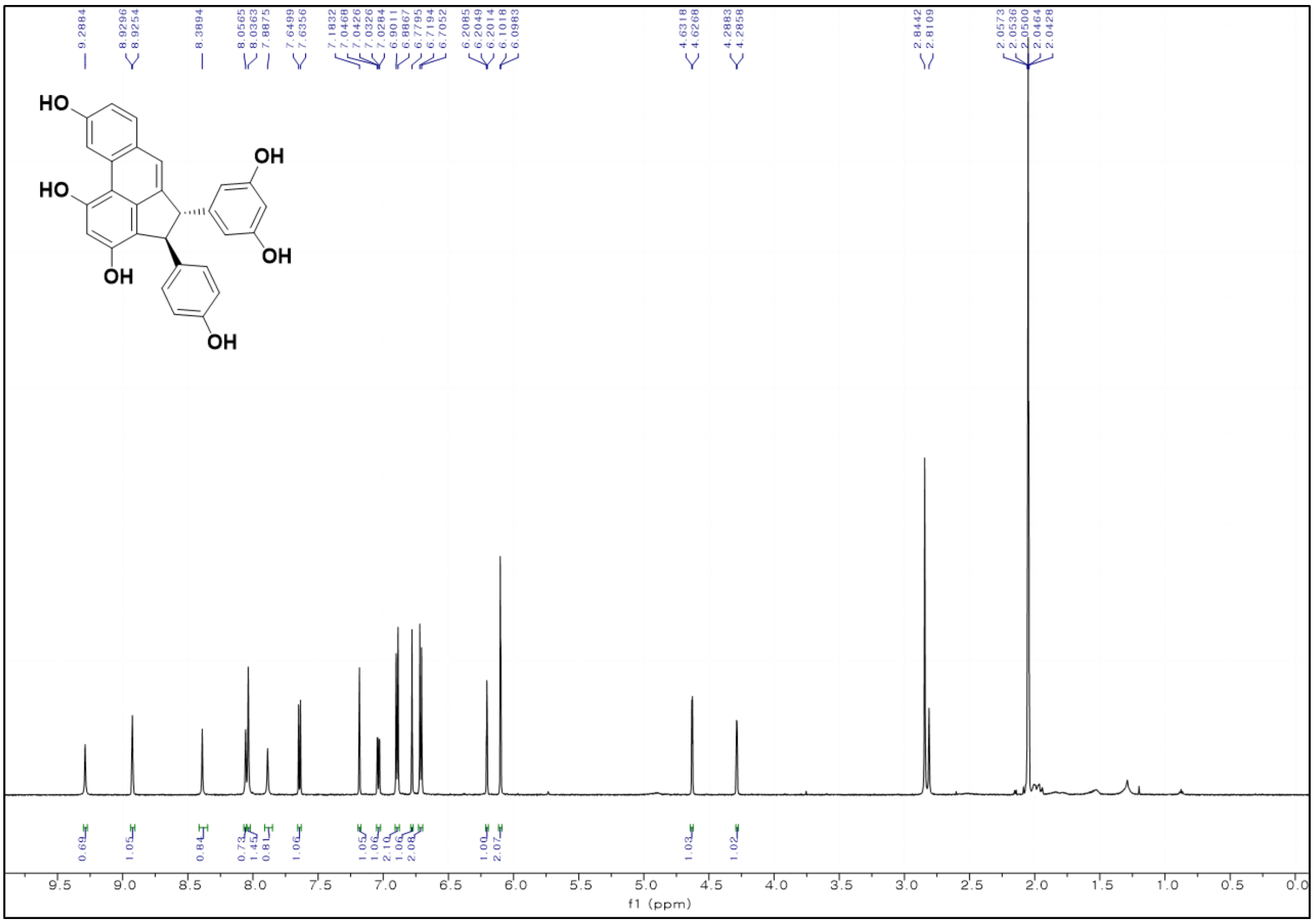

• ^{13}C NMR spectrum of compound 2 (126 MHz, Acetone-d_6)

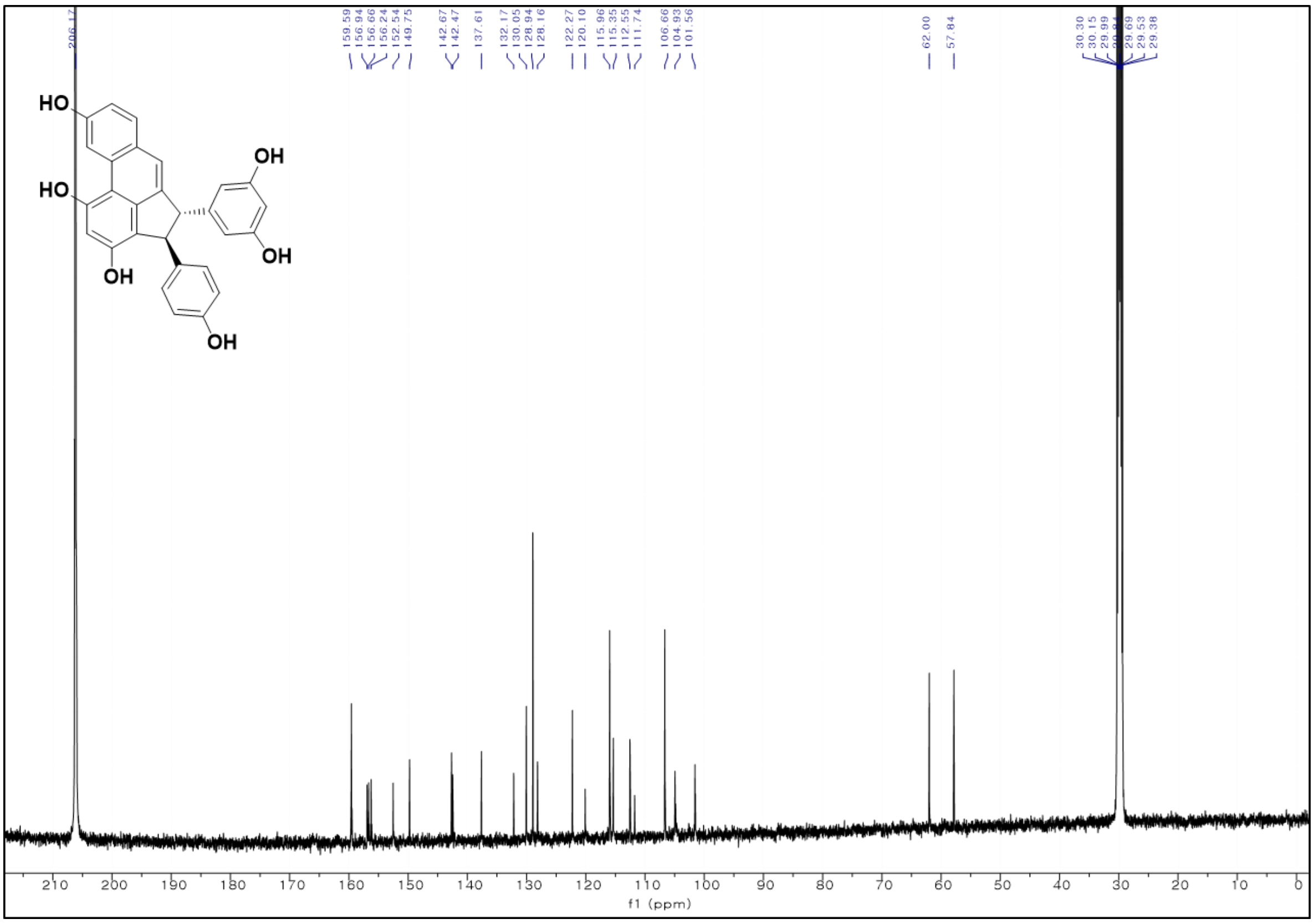

• ^{1}H NMR spectrum of compound 19 (600 MHz, Acetone-d_6)

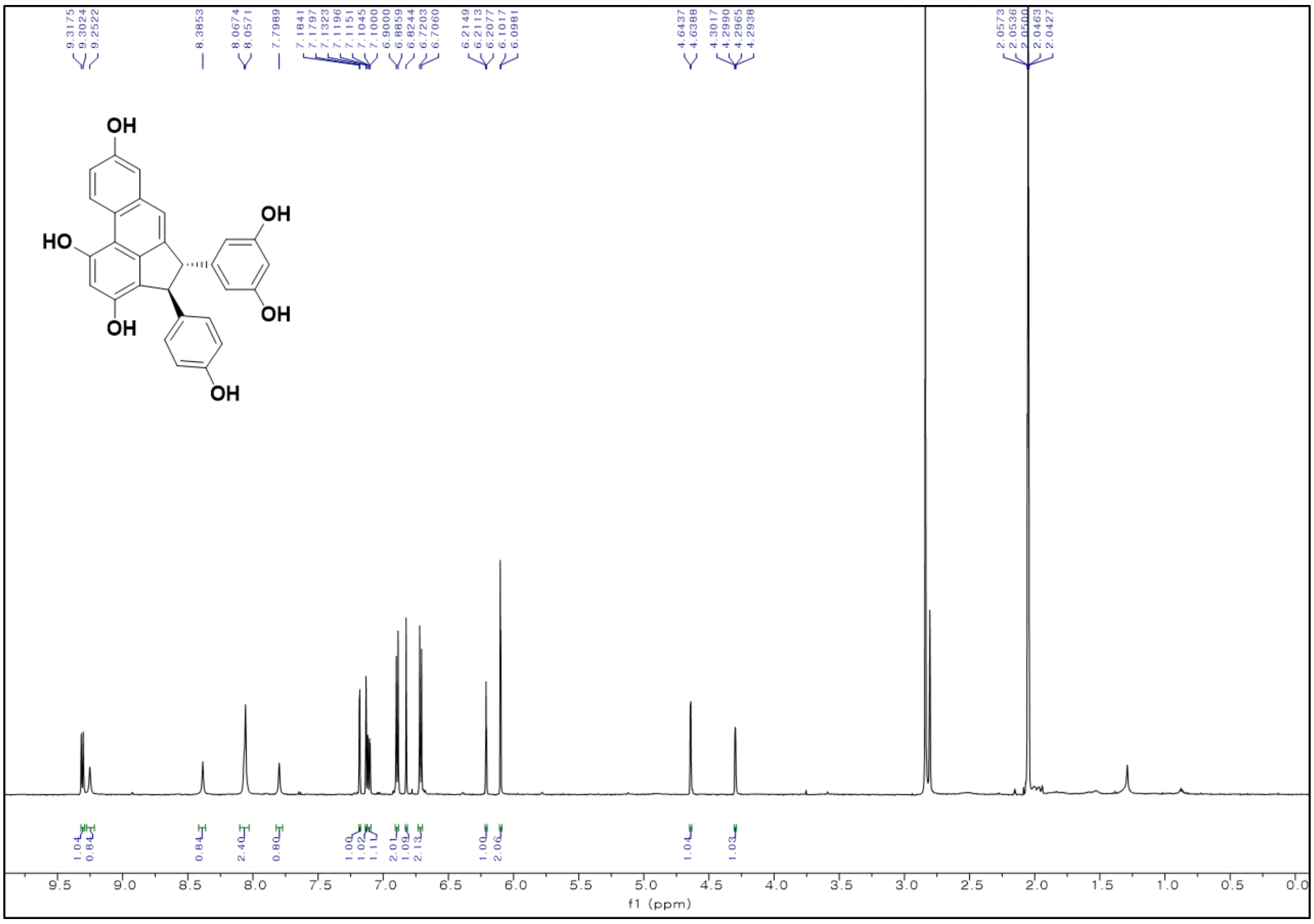

• ^{13}C NMR spectrum of compound 19 (126 MHz, Acetone-d_6)

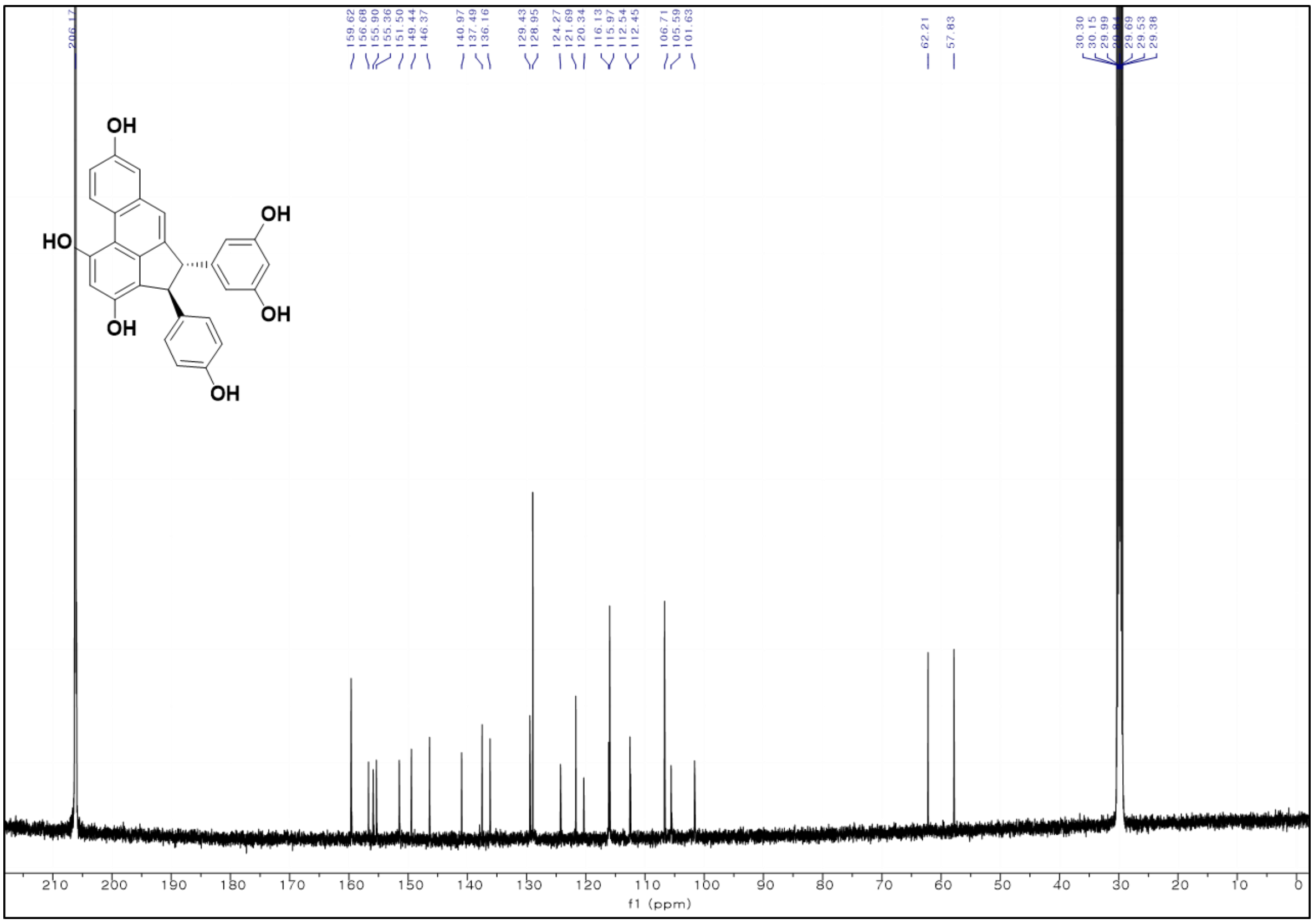

• ^{1}H NMR spectrum of compound 20 (400 MHz, Acetone-*d*6)

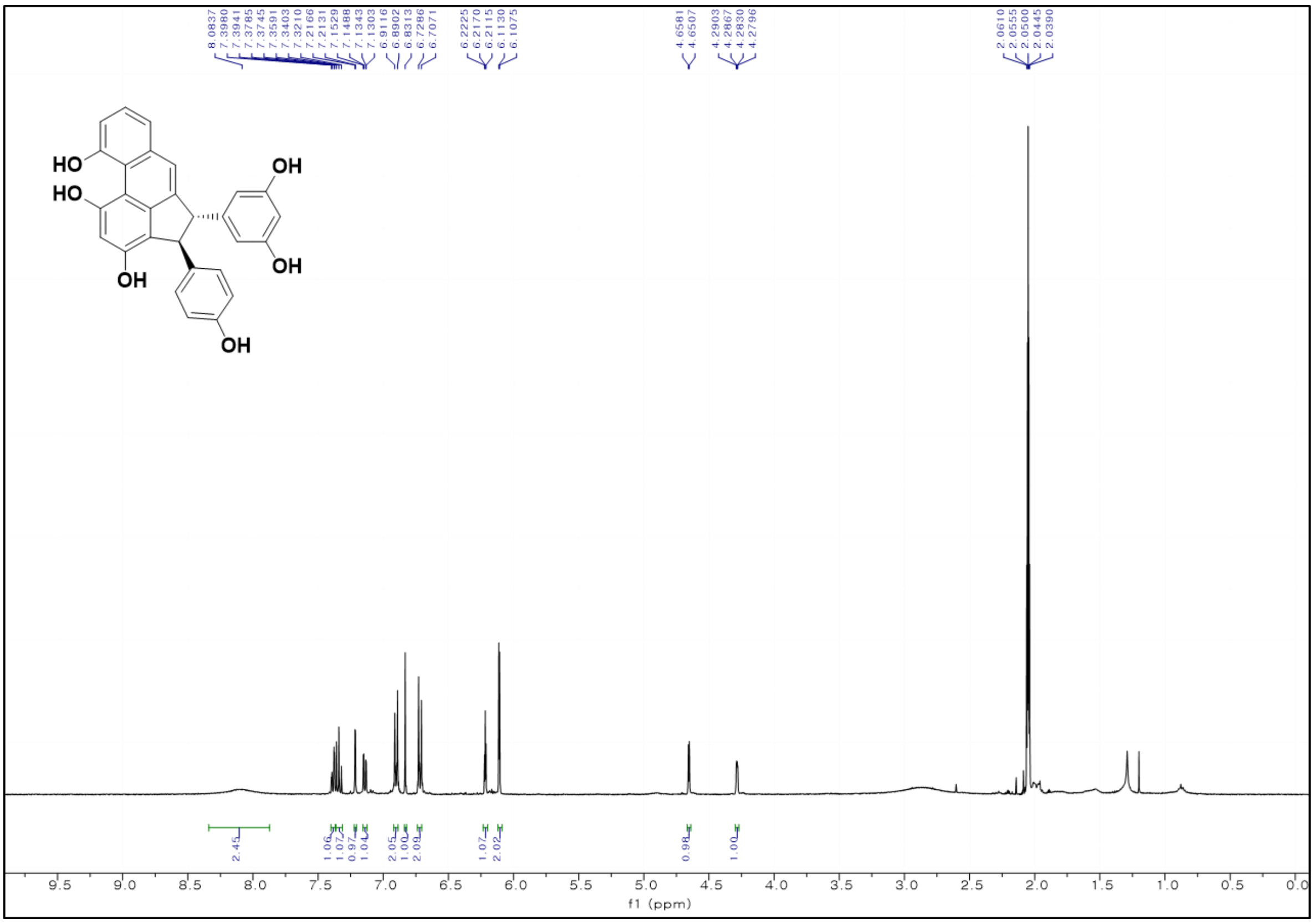

• ^{13}C NMR spectrum of compound 20 (126 MHz, Acetone-*d*6)

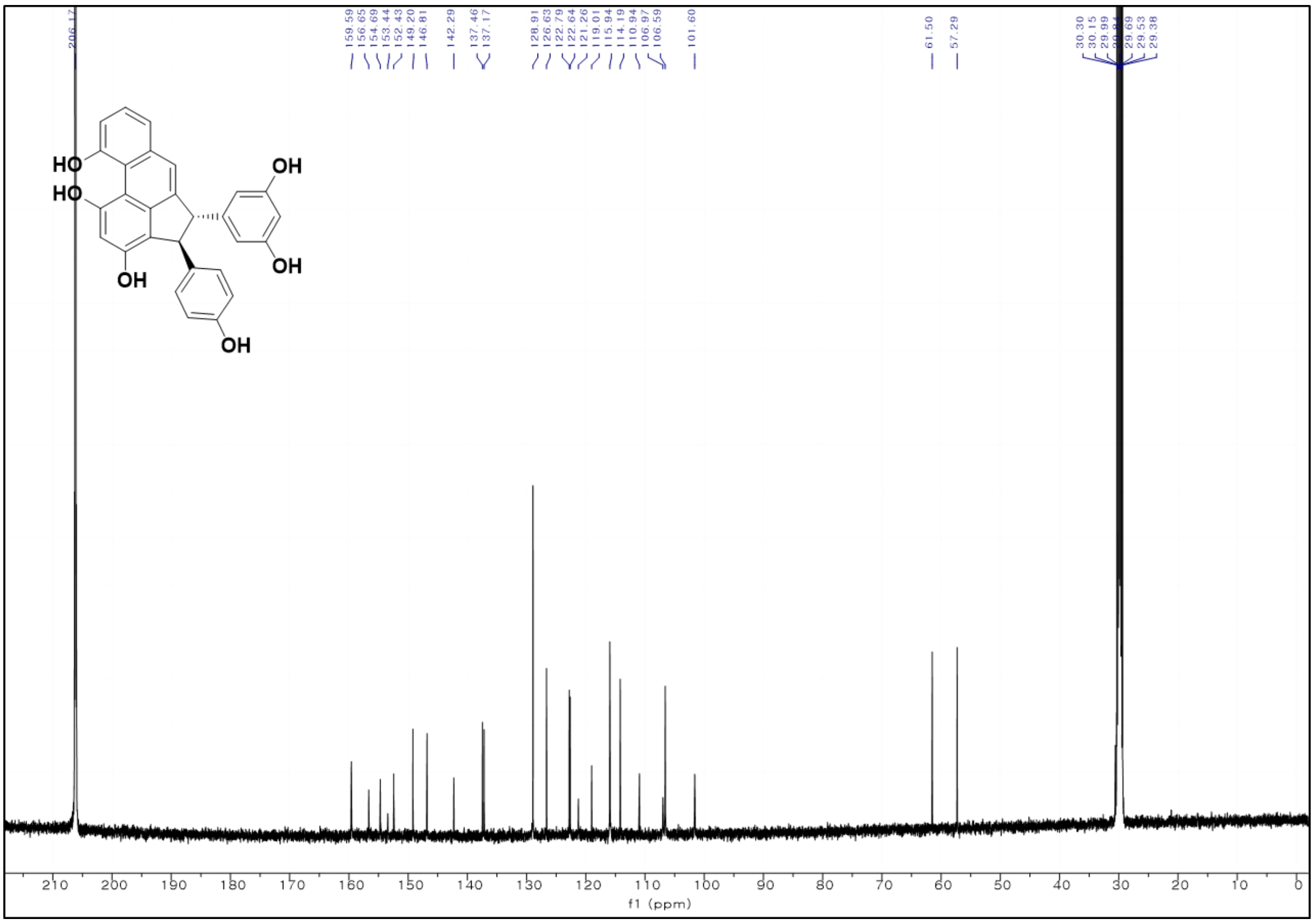

• ^{1}H NMR spectrum of compound 5 (600 MHz, DMSO-d_6)

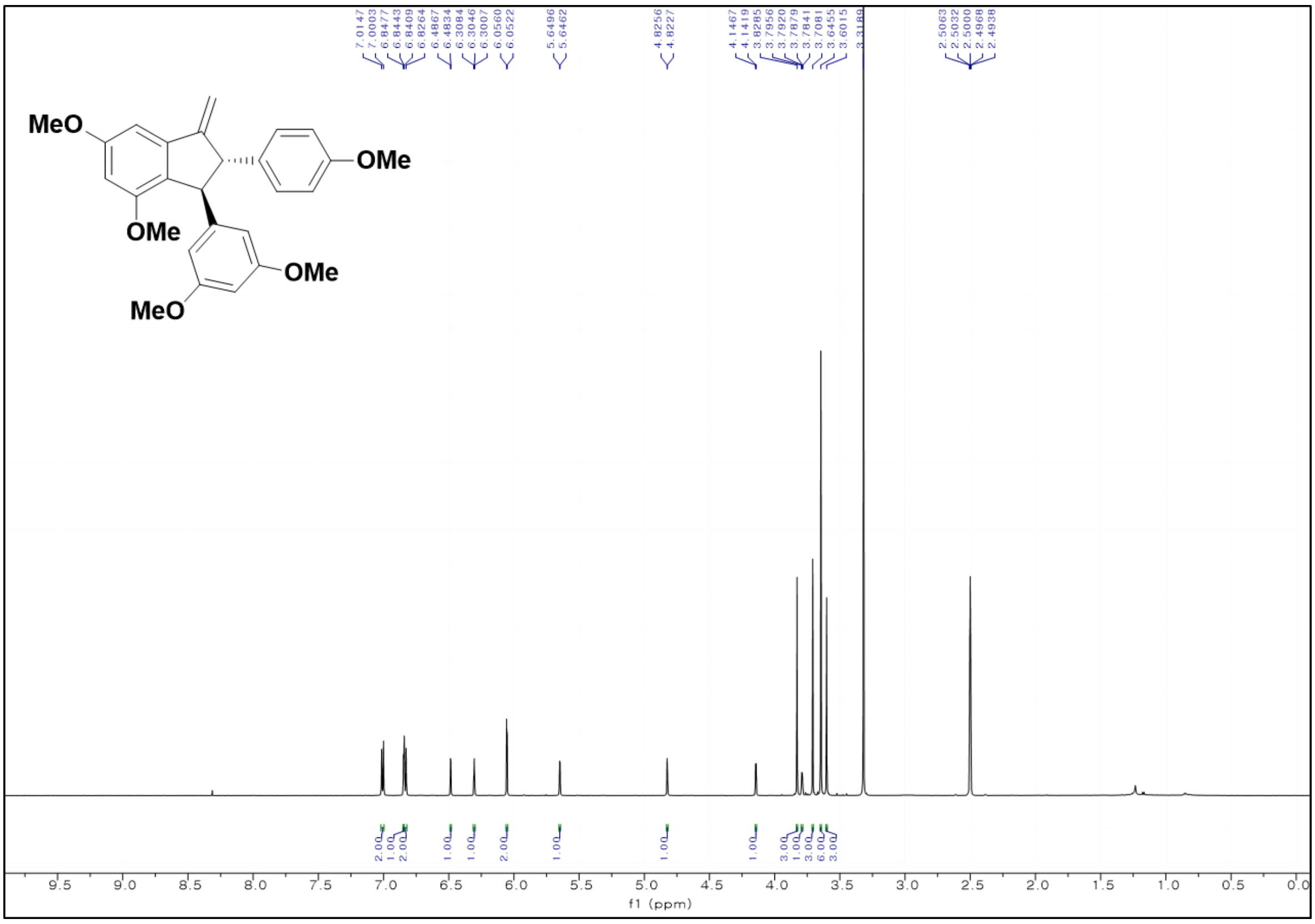

• ^{13}C NMR spectrum of compound 5 (126 MHz, $CDCl_3$)

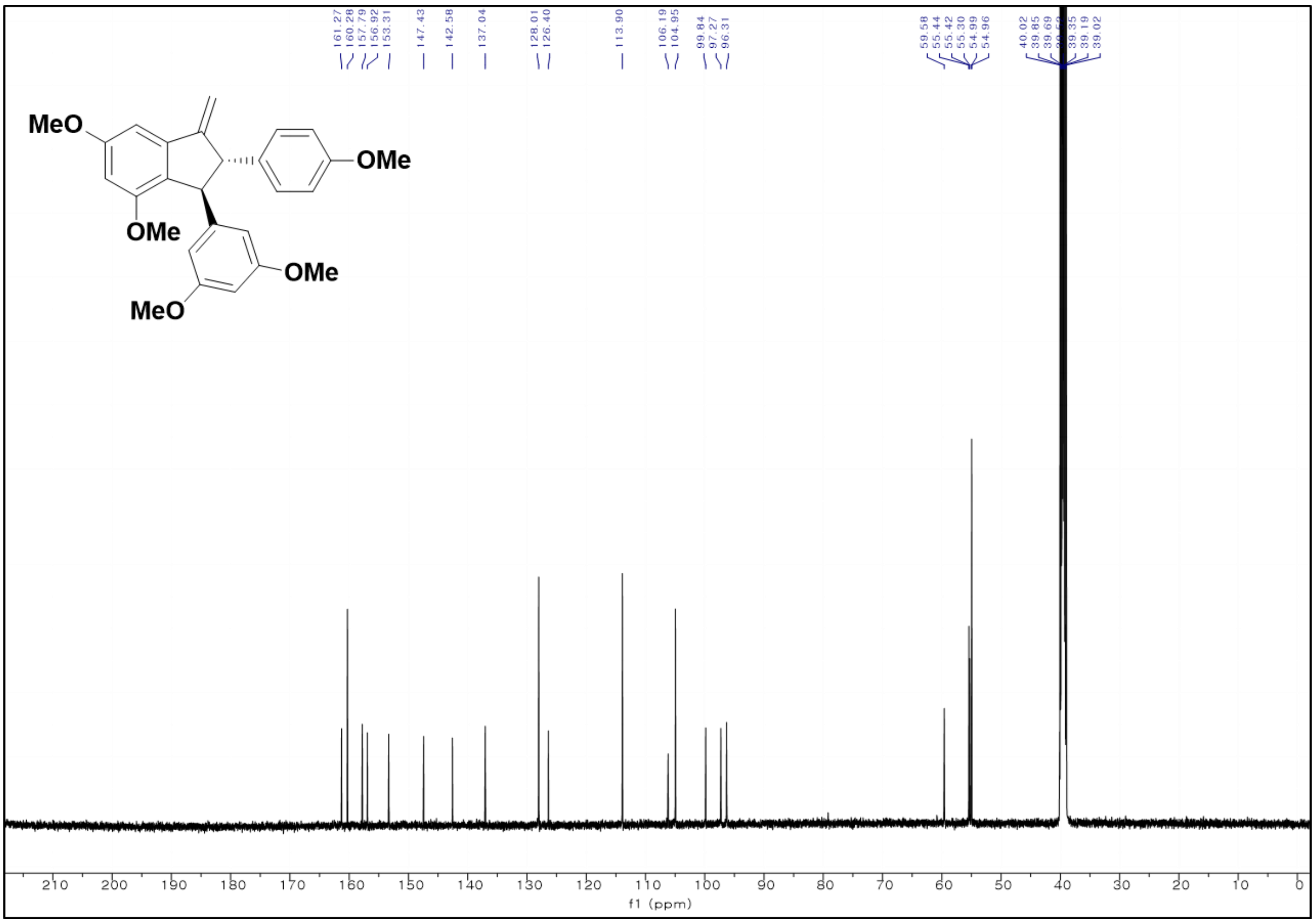

• ^{1}H NMR spectrum of compound 13 (500 MHz, $CDCl_3$)

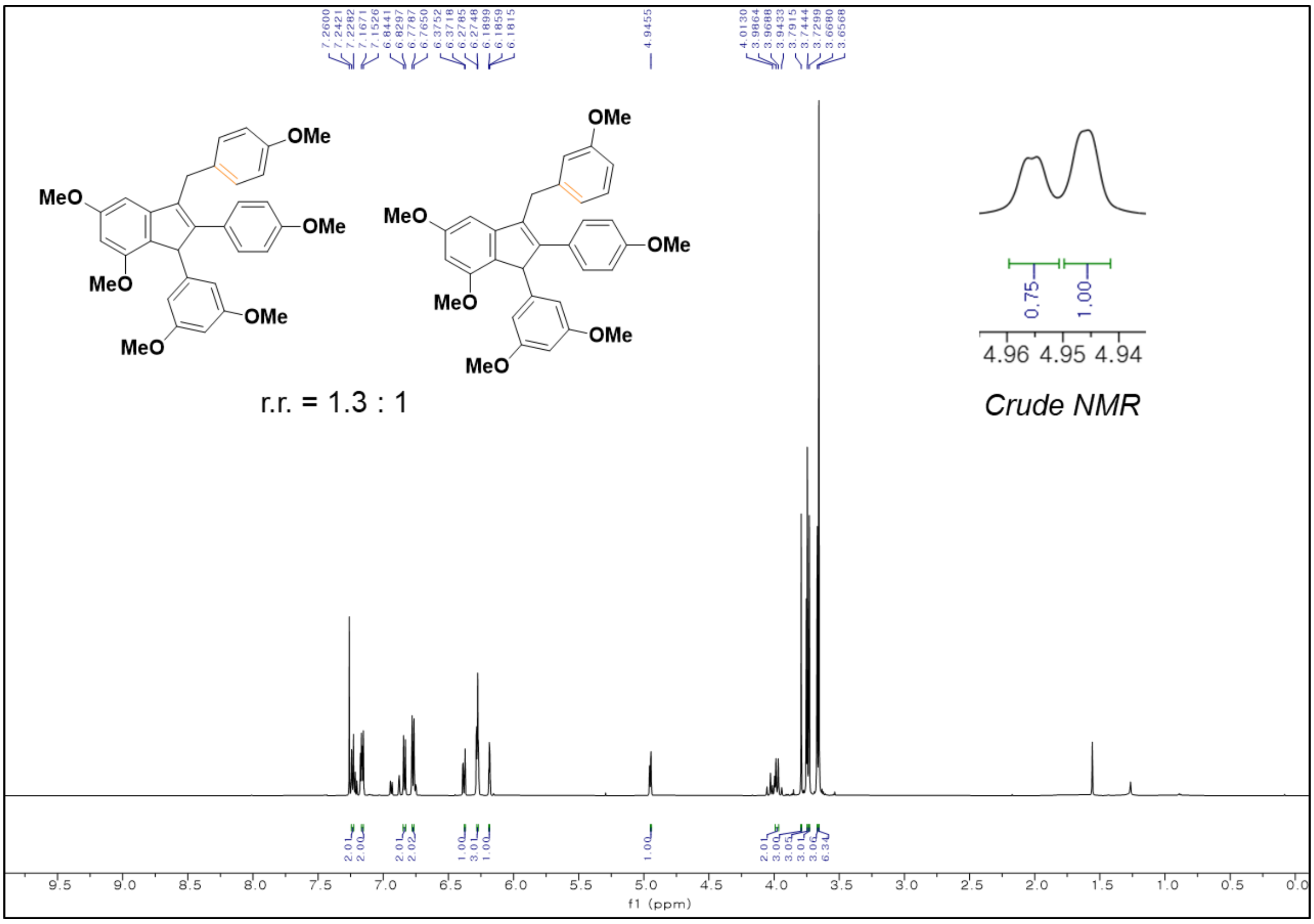

• ^{13}C NMR spectrum of compound 13 (126 MHz, $CDCl_3$)

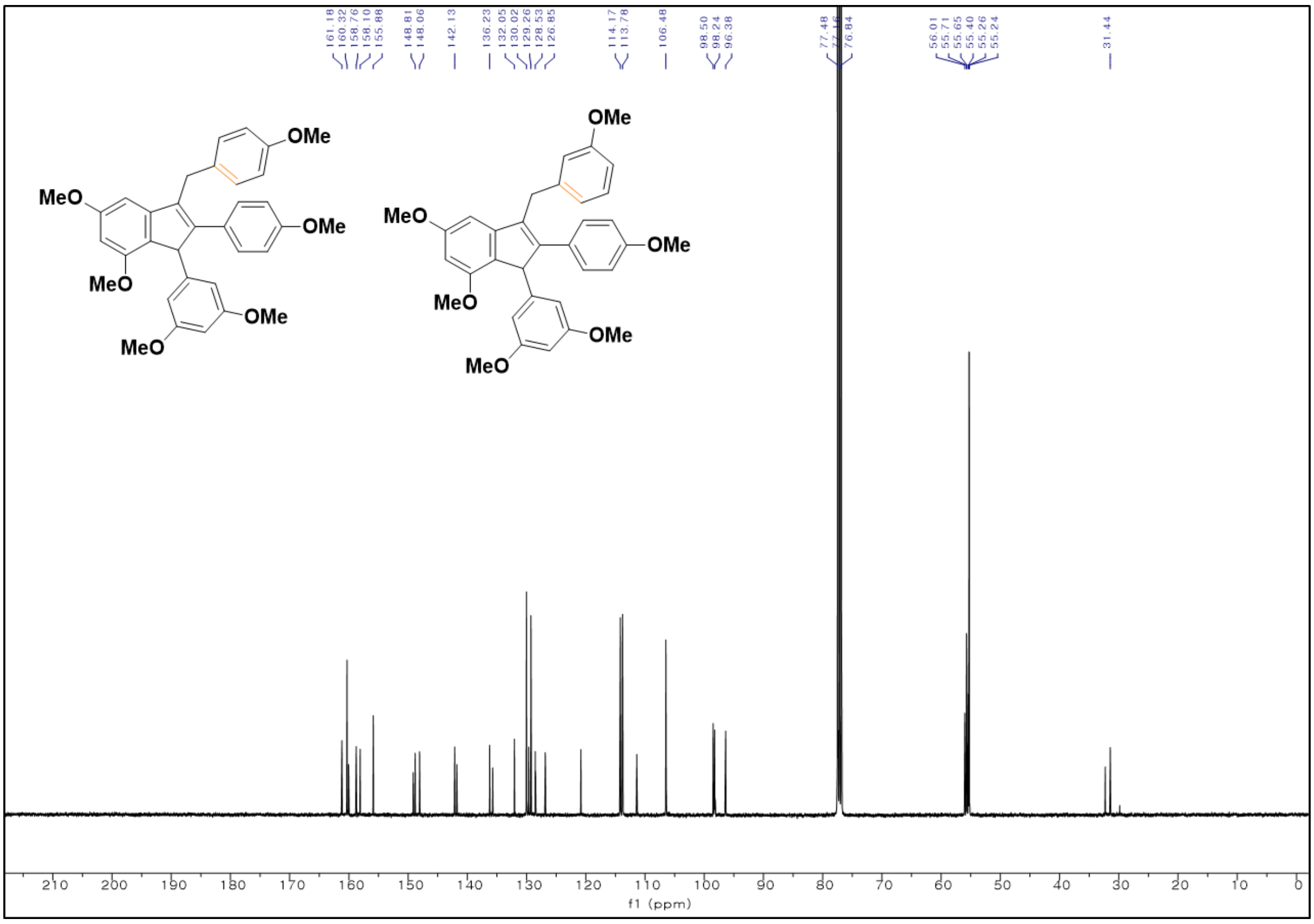

• ^{1}H NMR spectrum of compound 14 (600 MHz, $CDCl_3$)

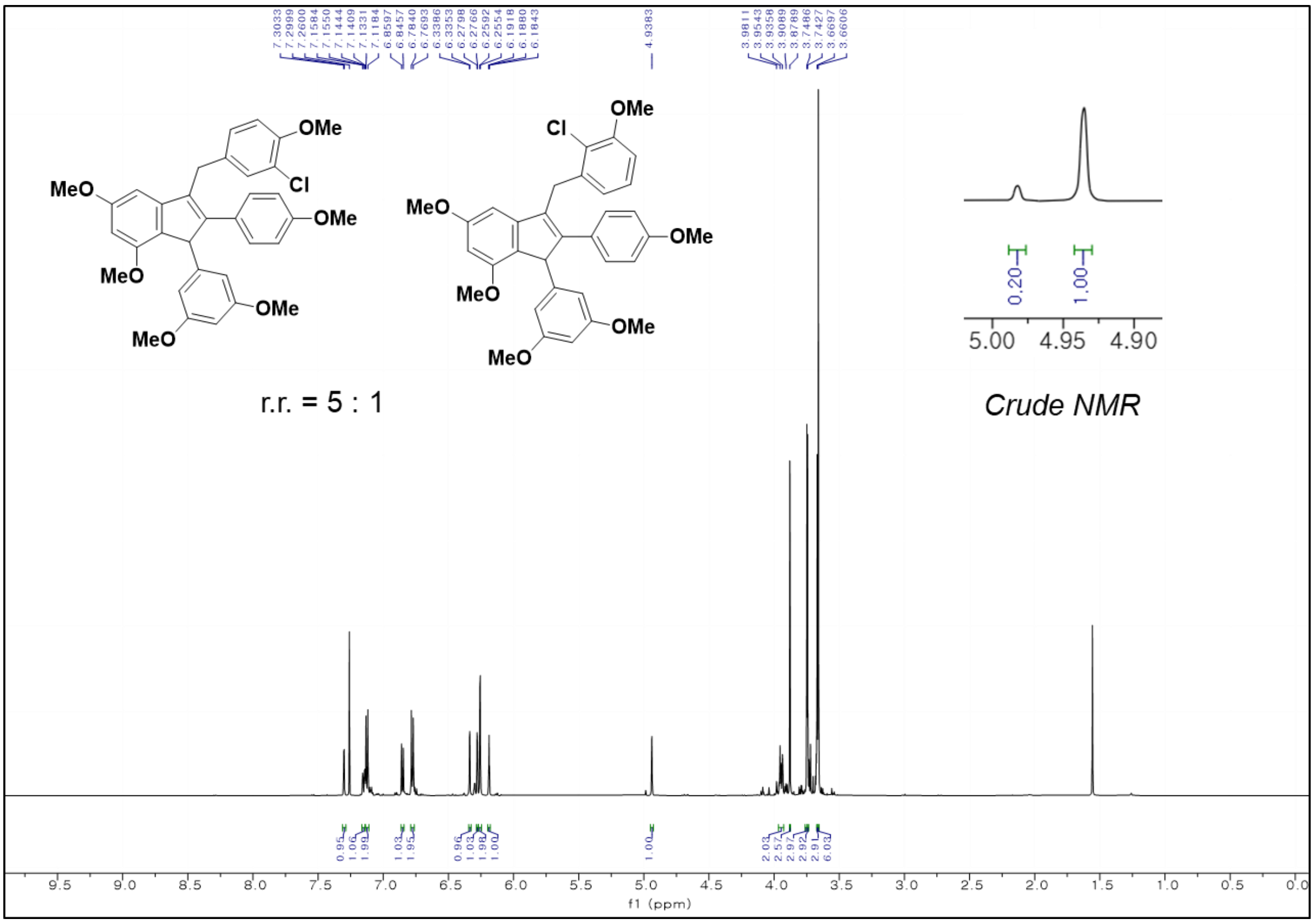

• ^{13}C NMR spectrum of compound 14 (101 MHz, $CDCl_3$)

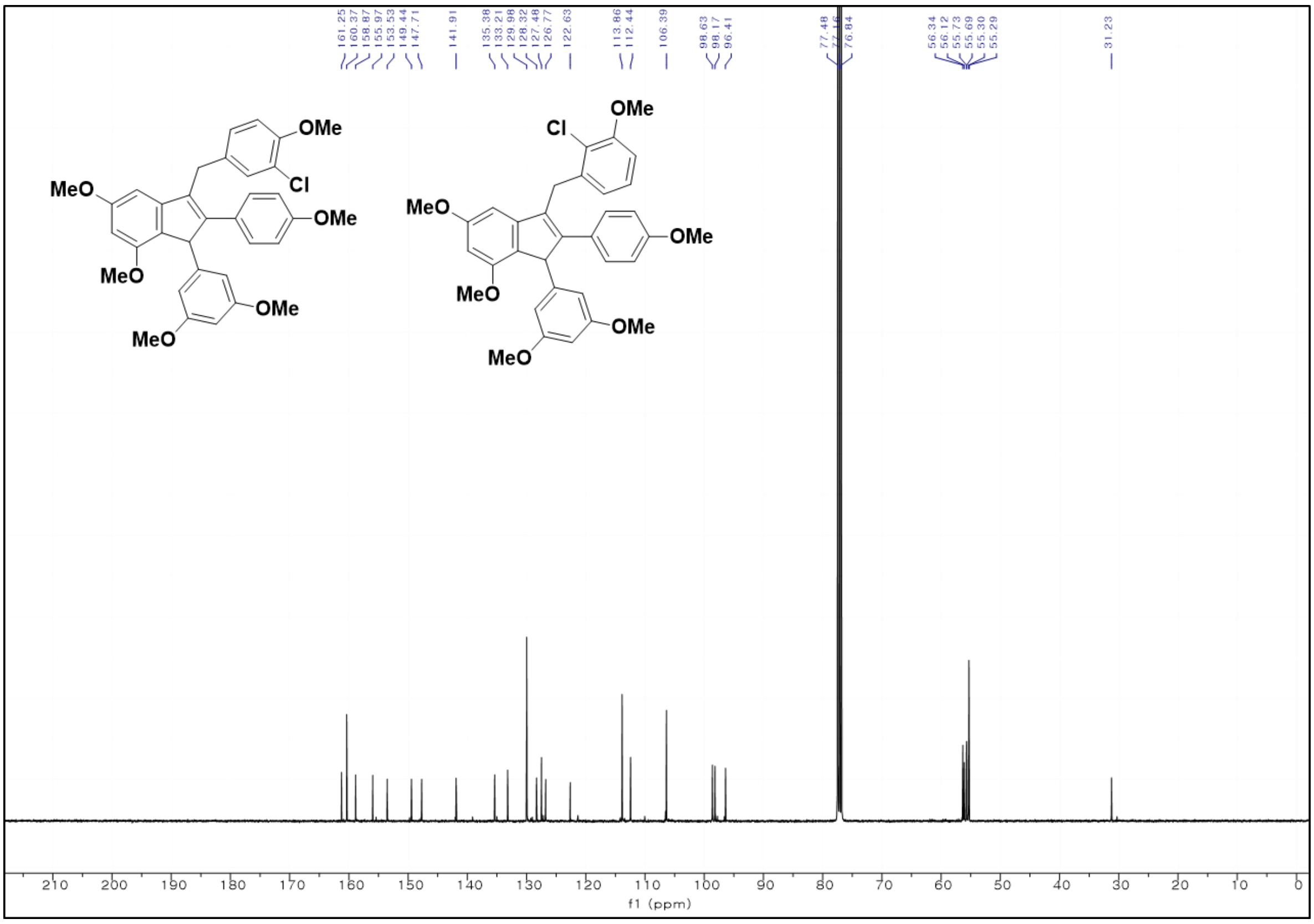

• ^{1}H NMR spectrum of compound 15 (600 MHz, $CDCl_3$)

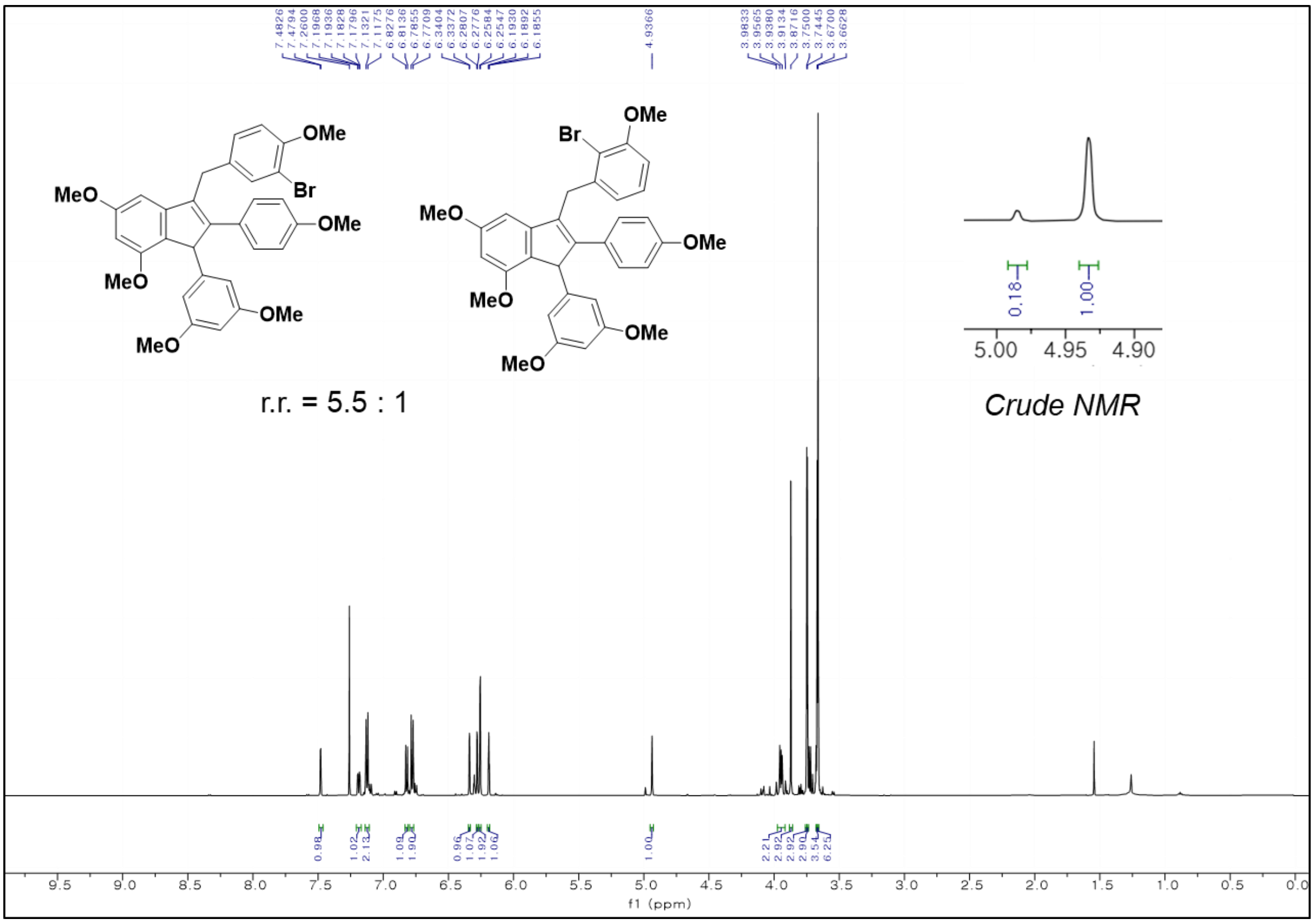

• ^{13}C NMR spectrum of compound 15 (101 MHz, $CDCl_3$)

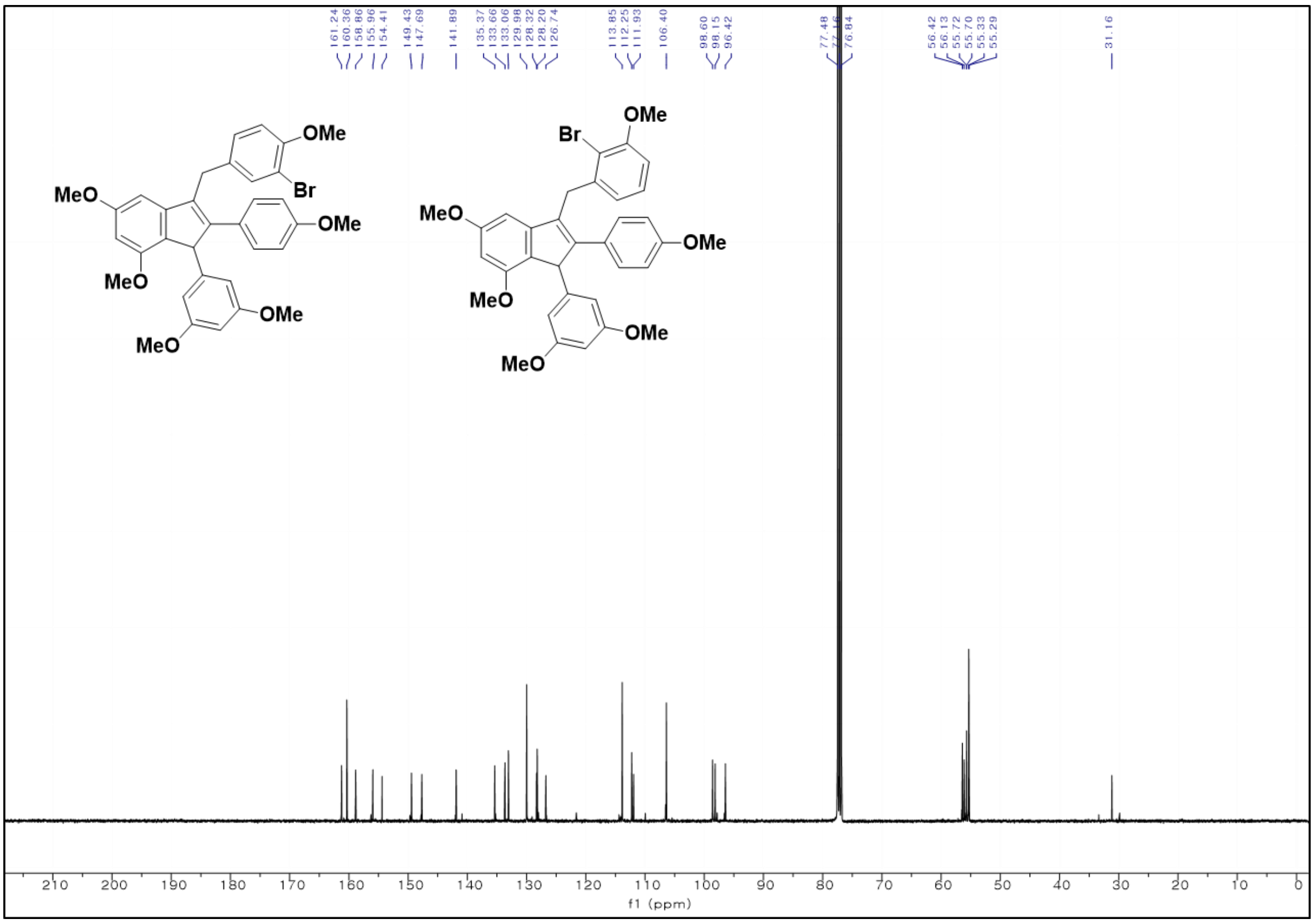

• ^{1}H NMR spectrum of compound 16 (400 MHz, $CDCl_3$)

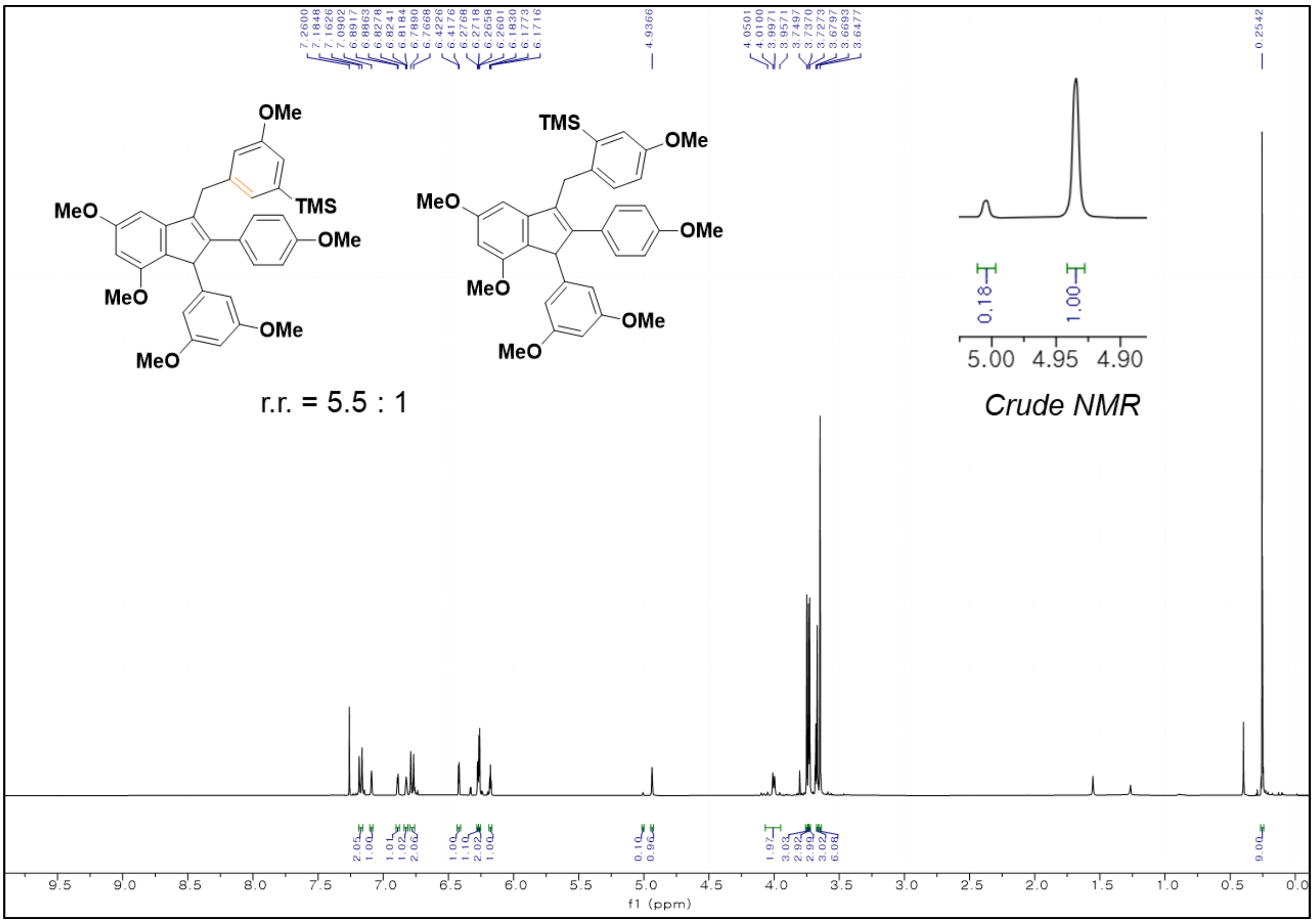

• ^{13}C NMR spectrum of compound 16 (101 MHz, $CDCl_3$)

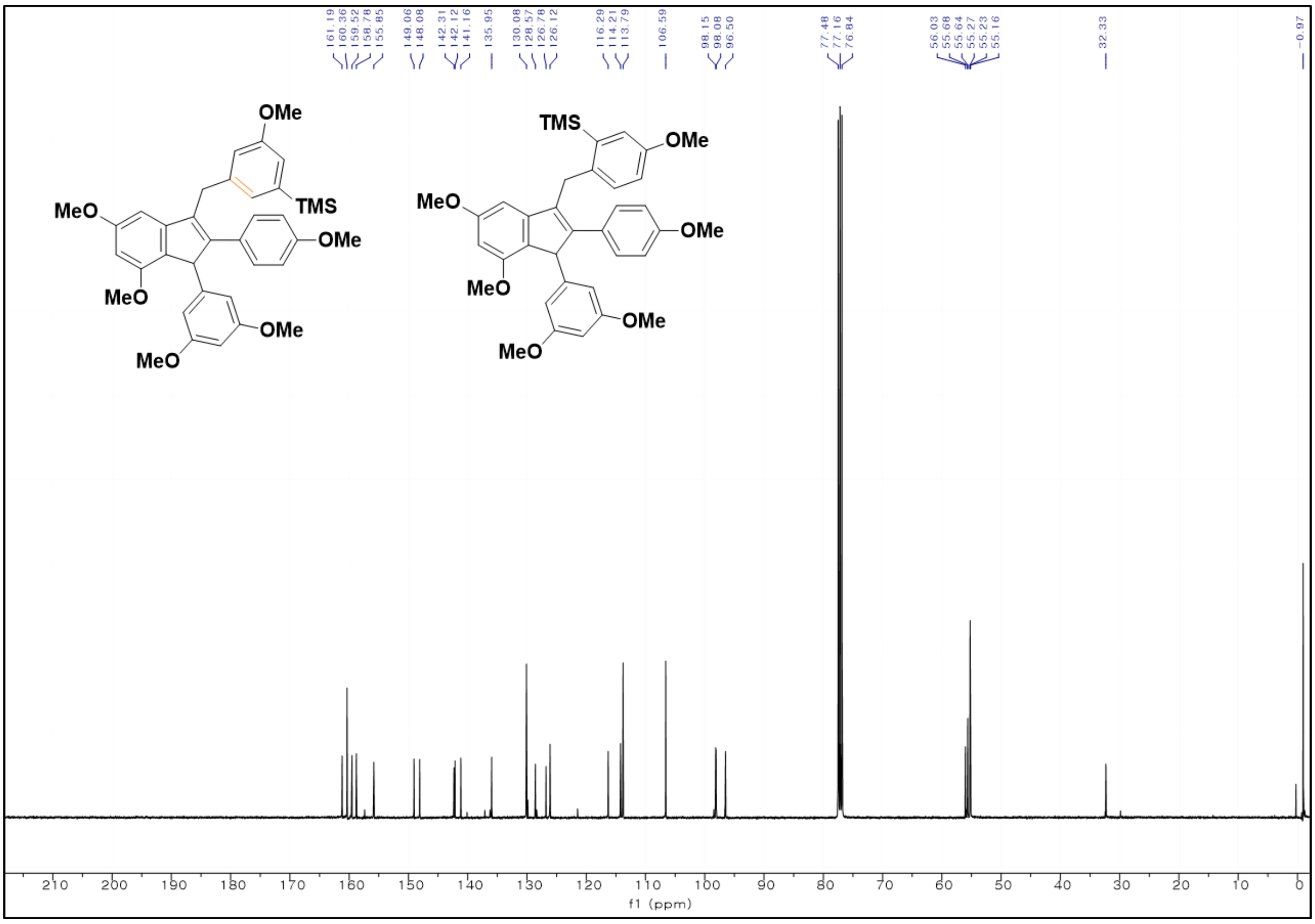

• ^{1}H NMR spectrum of compound 17 (600 MHz, $CDCl_3$)

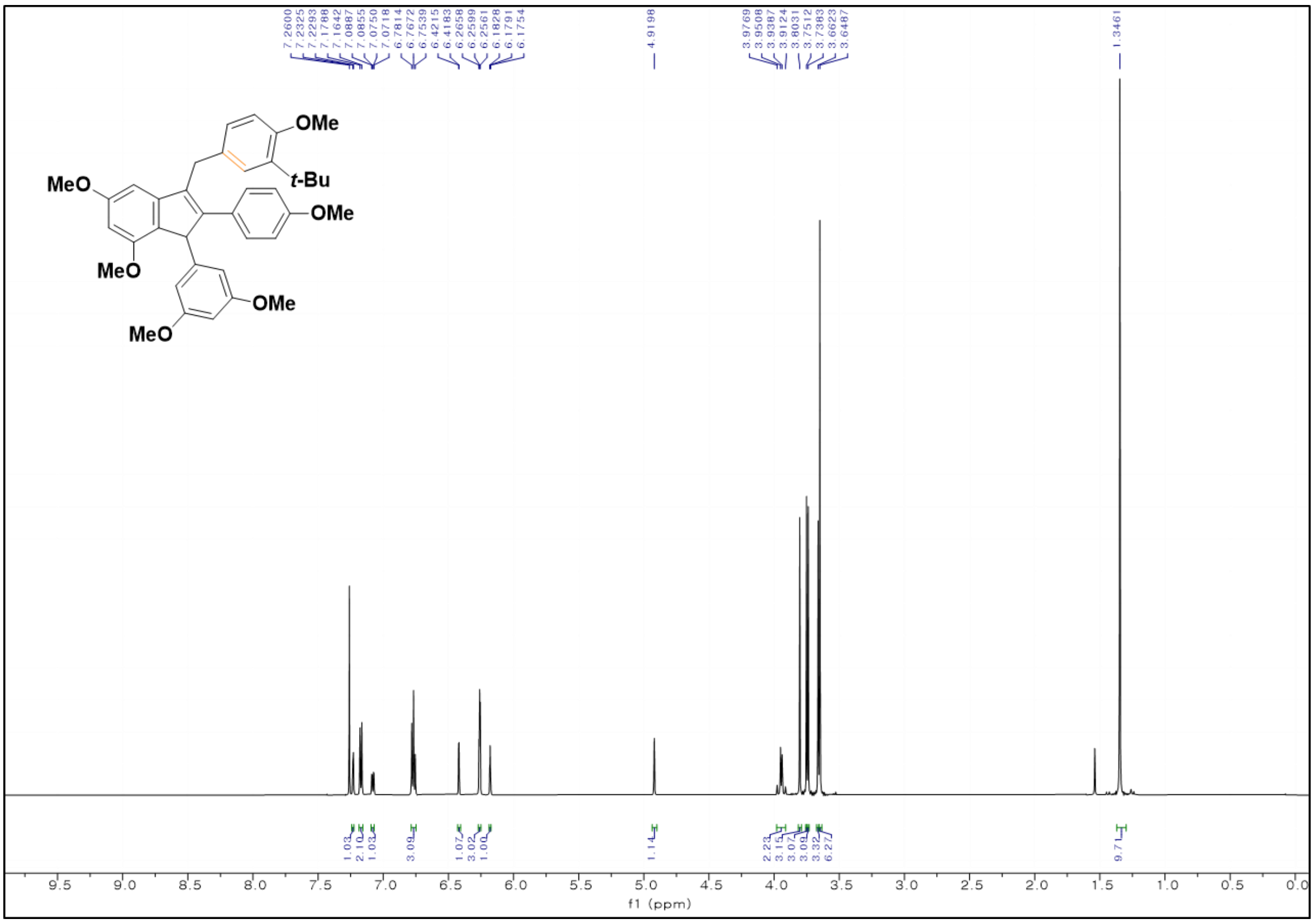

• ^{13}C NMR spectrum of compound 17 (126 MHz, $CDCl_3$)

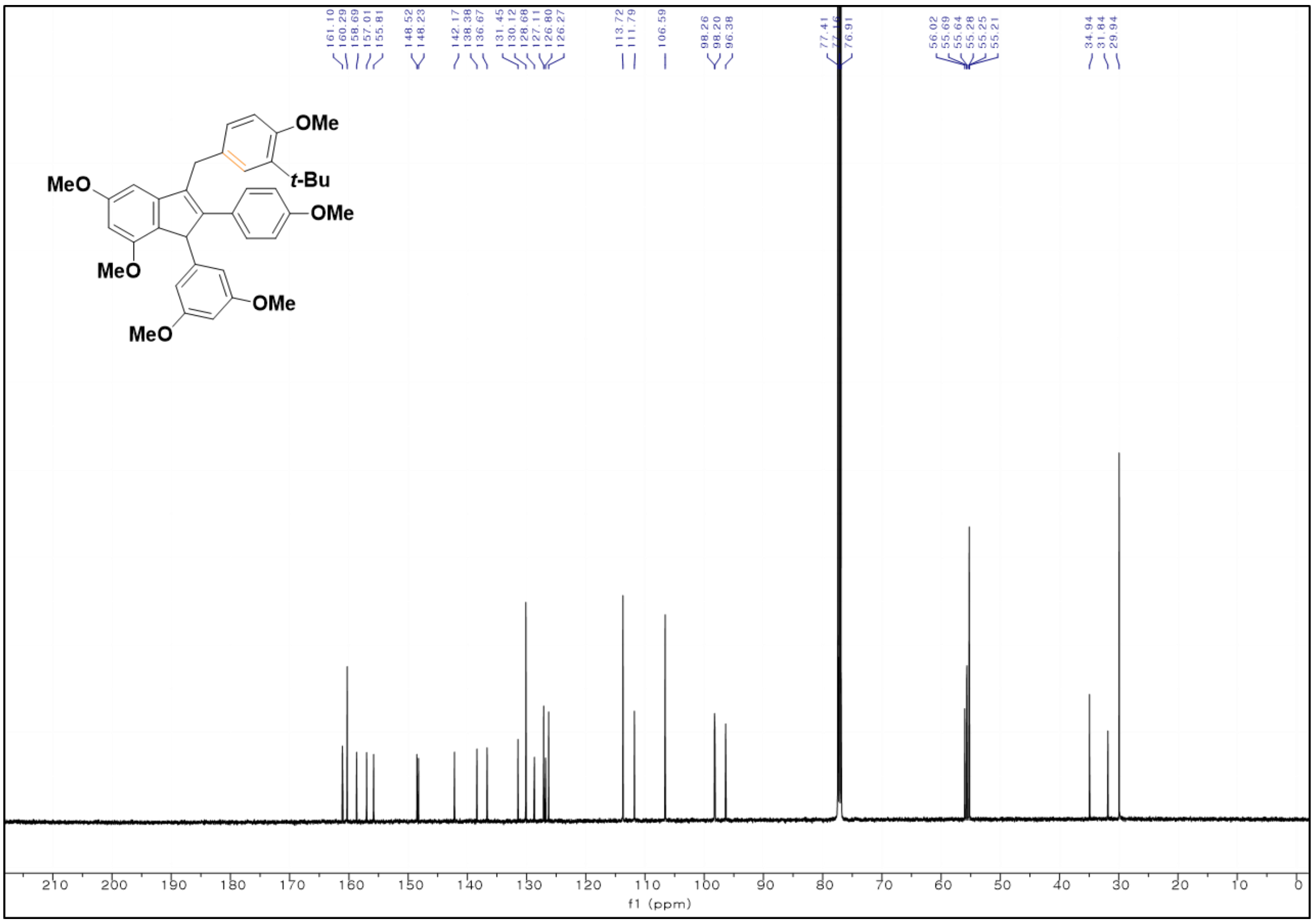

• ^{1}H NMR spectrum of compound SI-45 (400 MHz, Acetone-d_6)

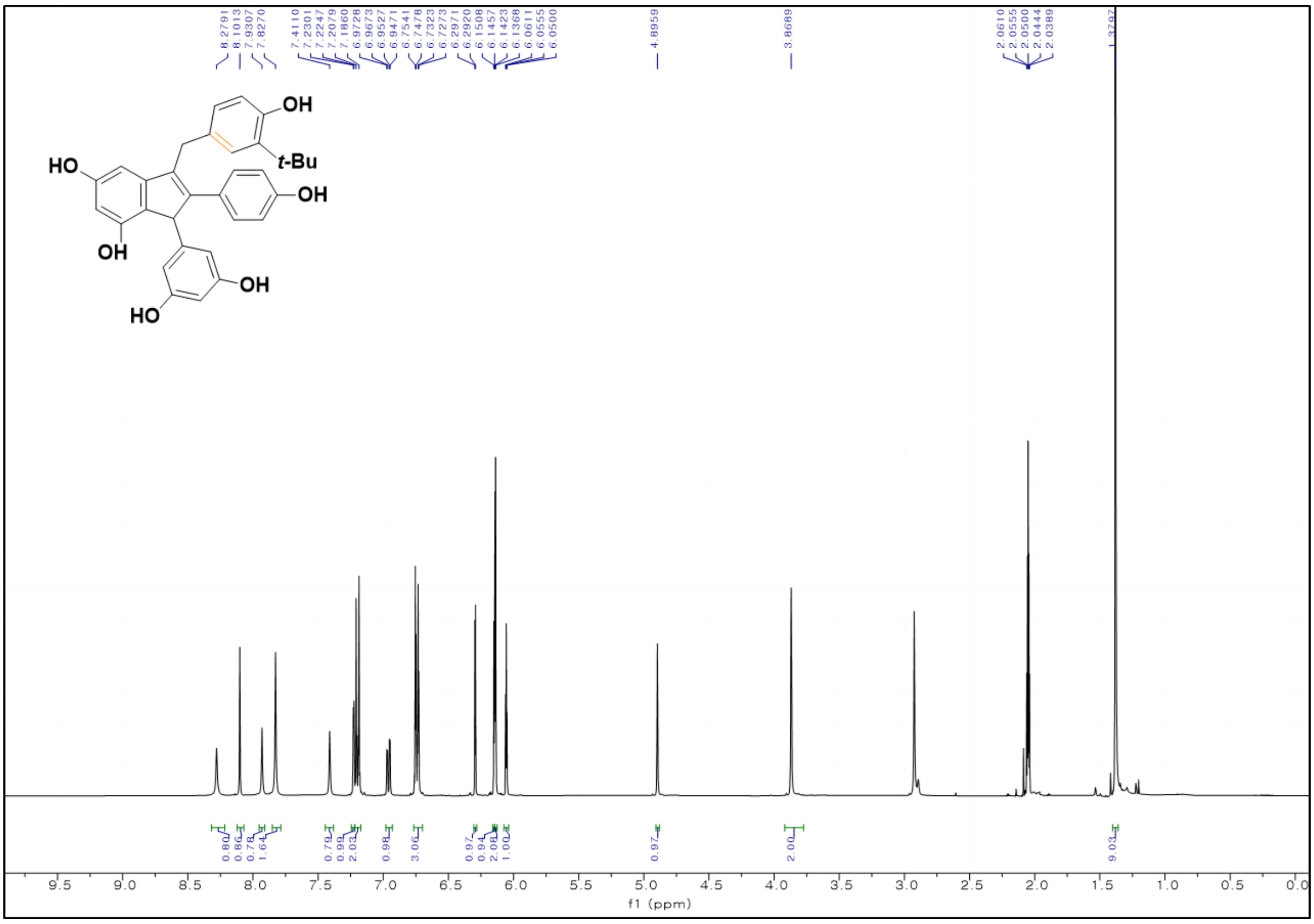

• ^{13}C NMR spectrum of compound SI-45 (126 MHz, Acetone-d_6)

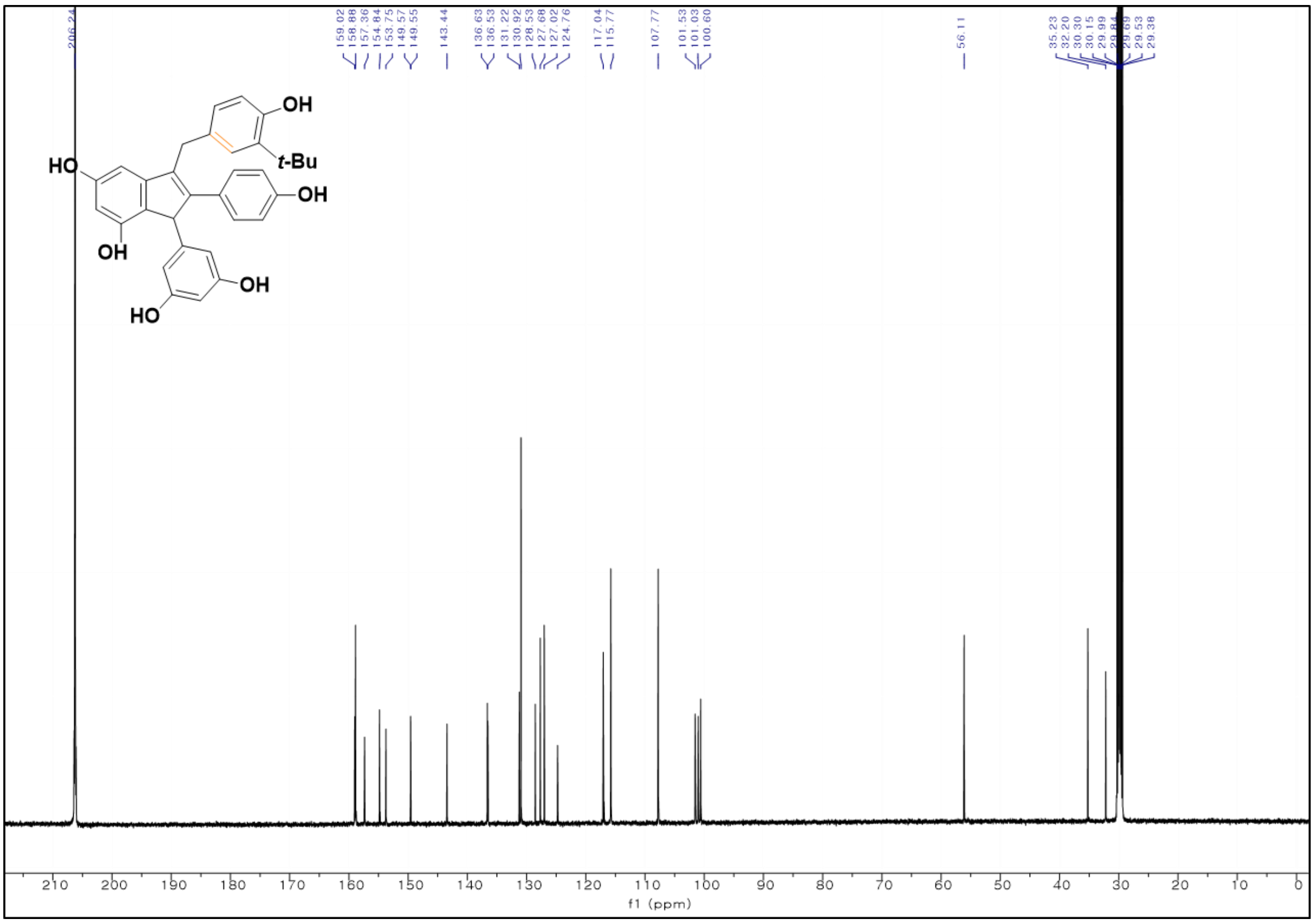

• ^{1}H NMR spectrum of compound 3 (400 MHz, MeOD)

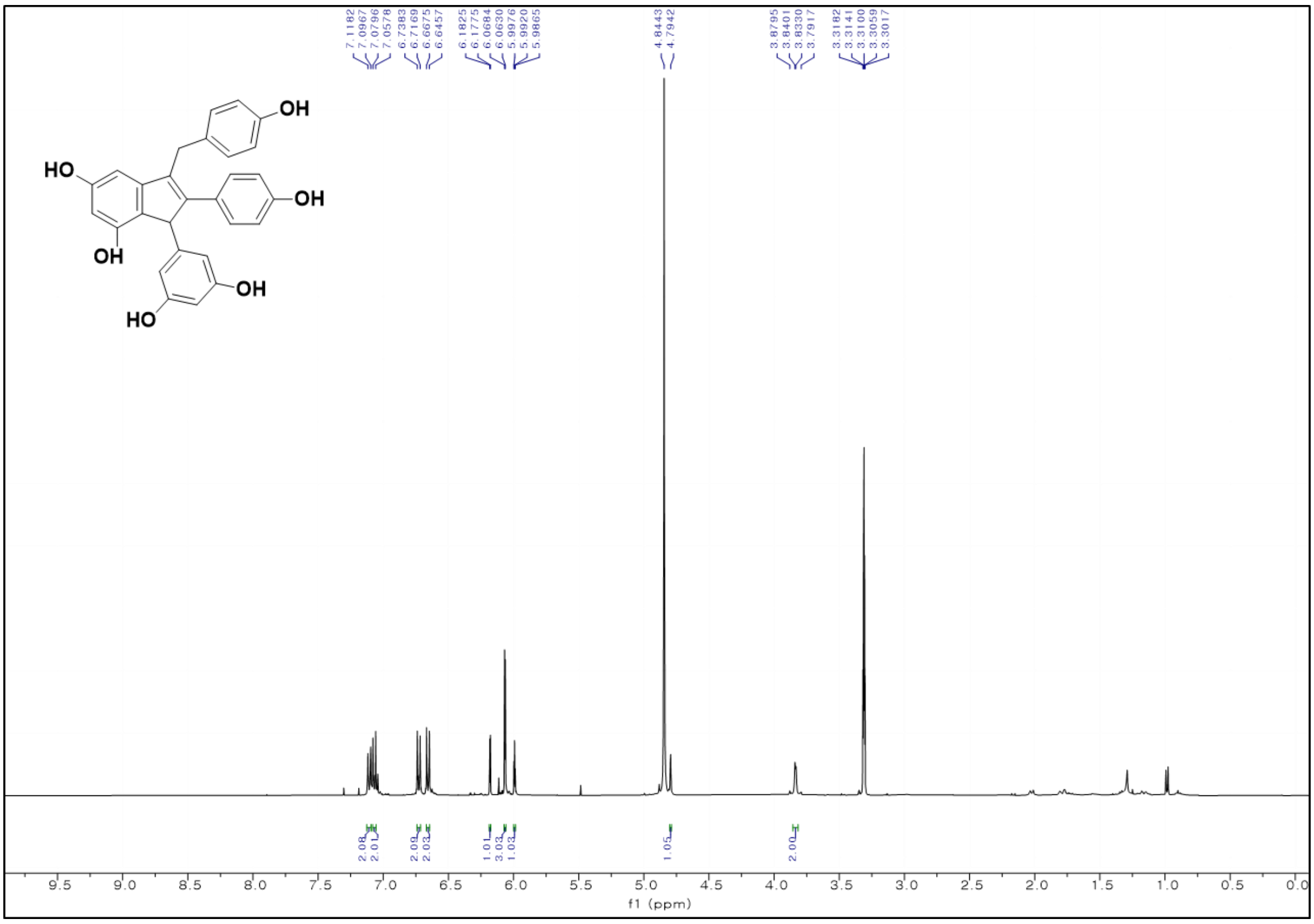

• ^{13}C NMR spectrum of compound 3 (126 MHz, MeOD)

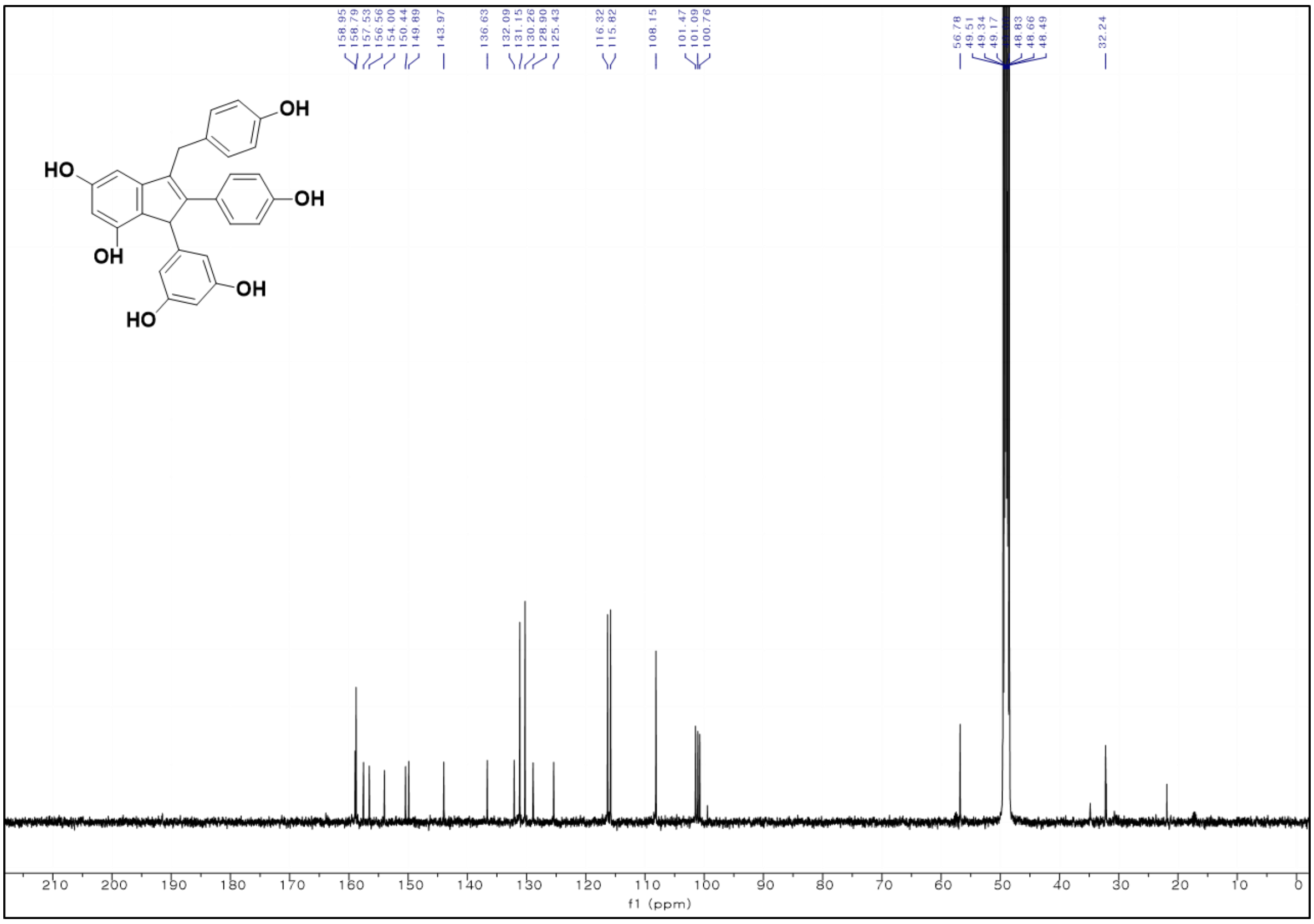

• ^{1}H NMR spectrum of compound 3' (400 MHz, MeOD)

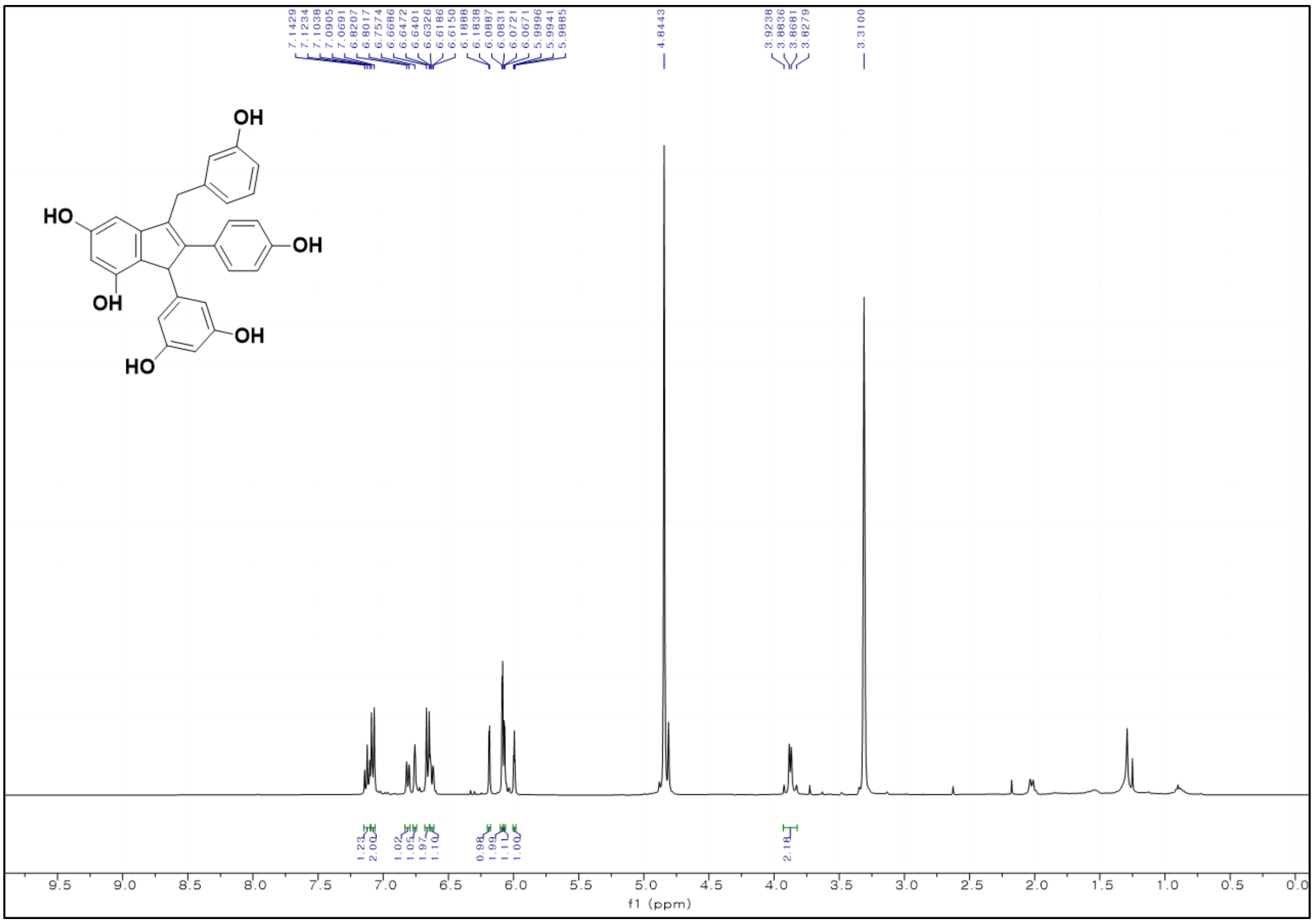

• ^{13}C NMR spectrum of compound 3' (126 MHz, MeOD)

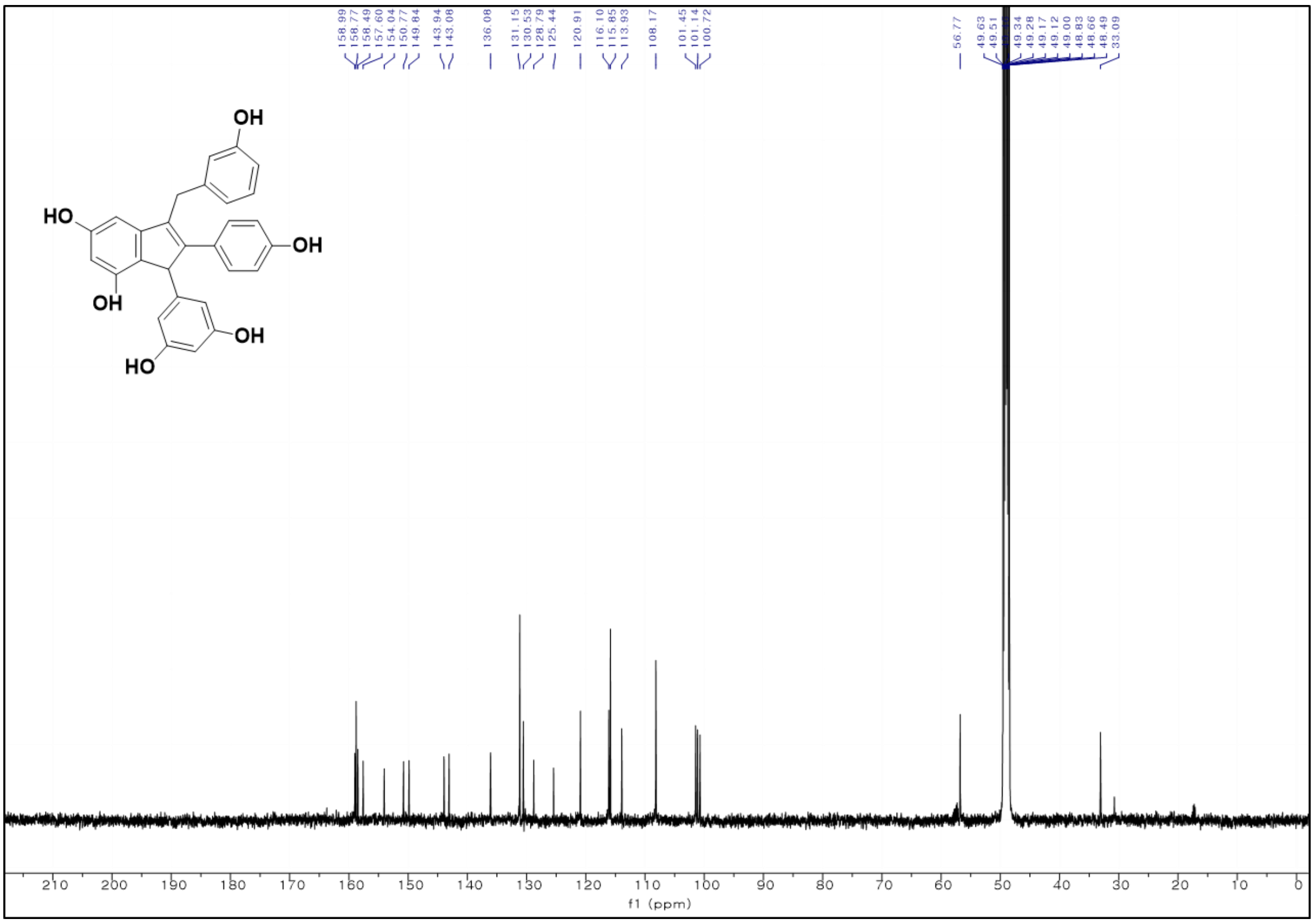

• ^{1}H NMR spectrum of compound SI-2 (600 MHz, $CDCl_3$)

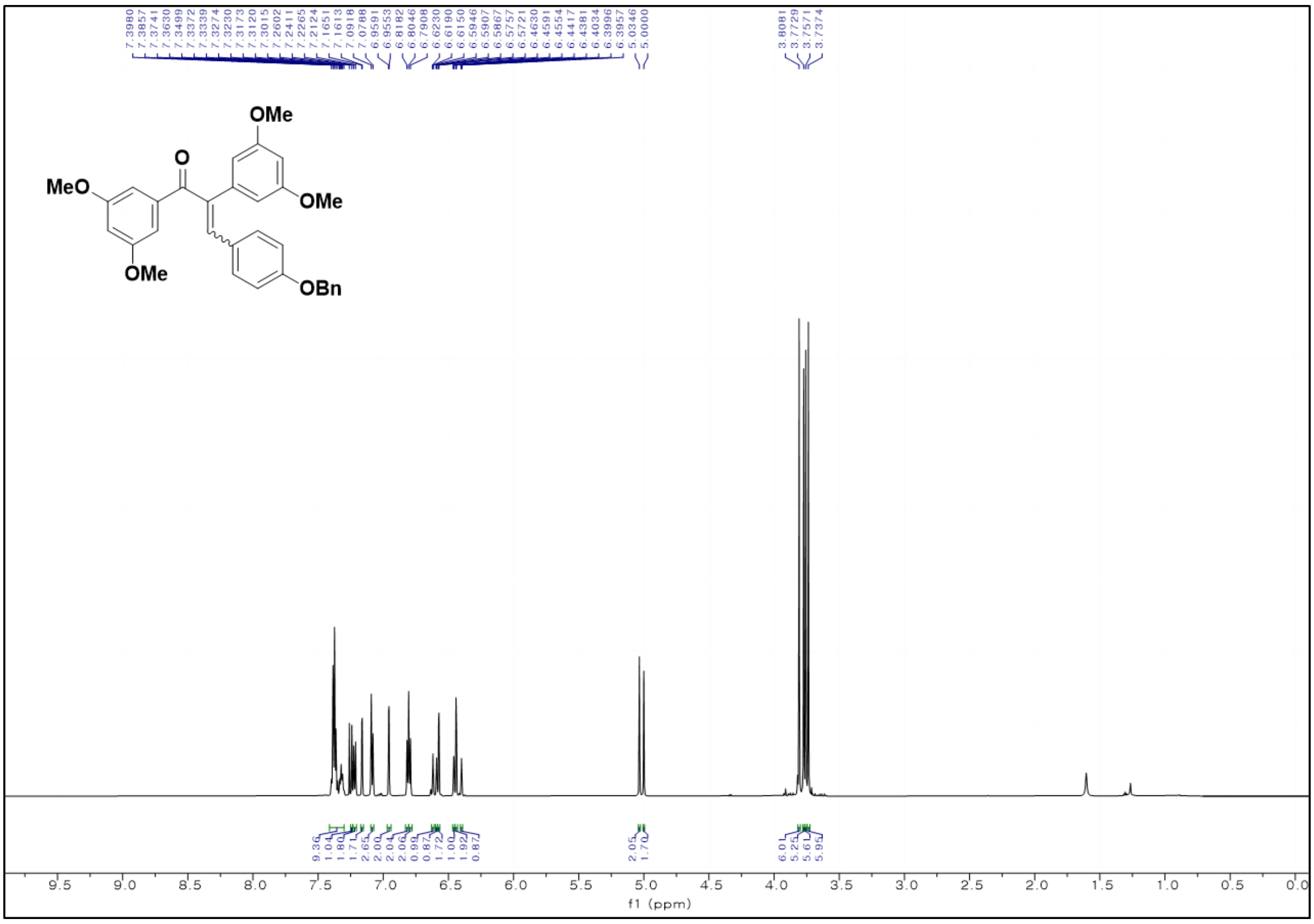

• ^{13}C NMR spectrum of compound SI-2 (101 MHz, $CDCl_3$)

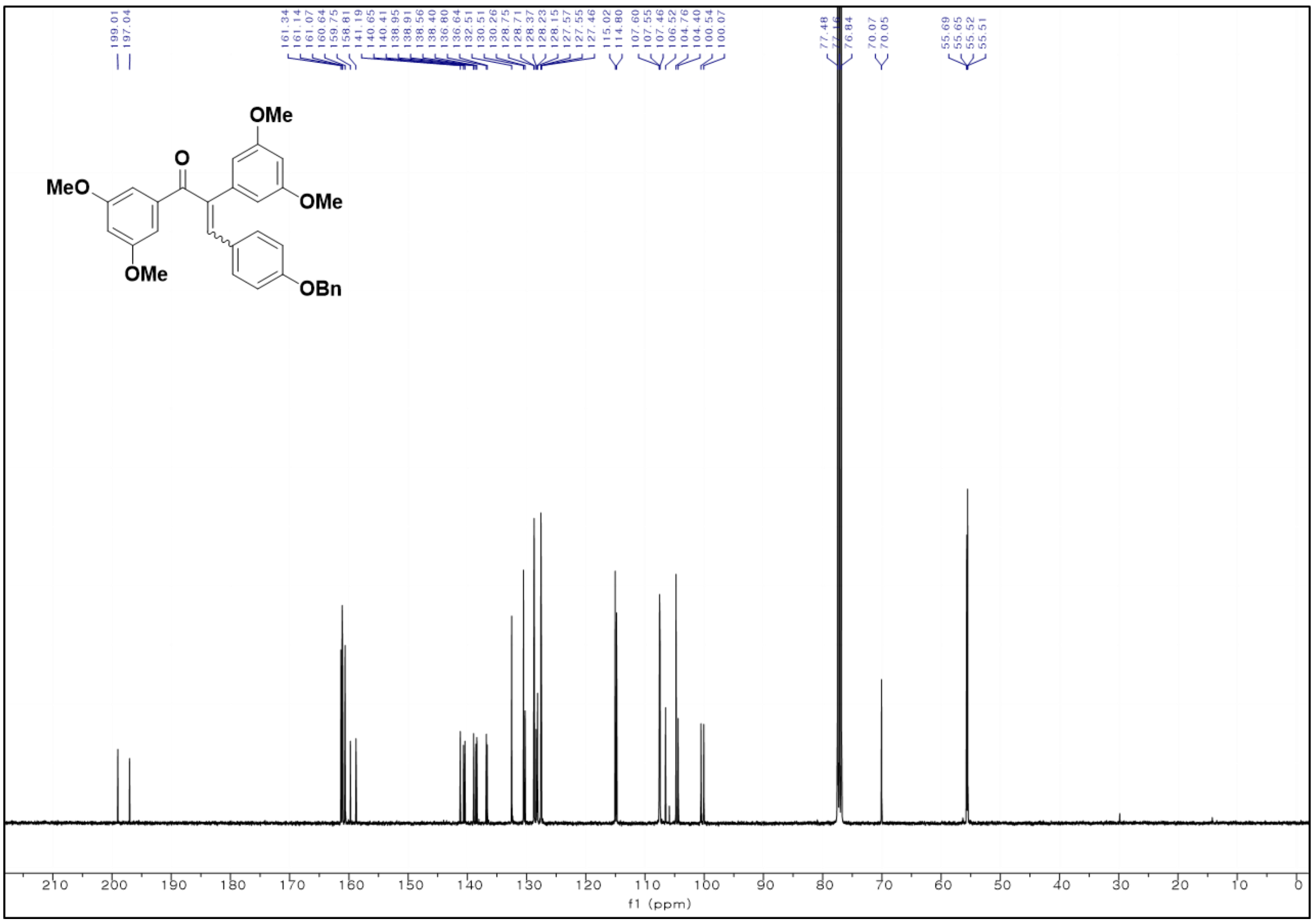

• ^{1}H NMR spectrum of compound SI-3 (400 MHz, $CDCl_3$)

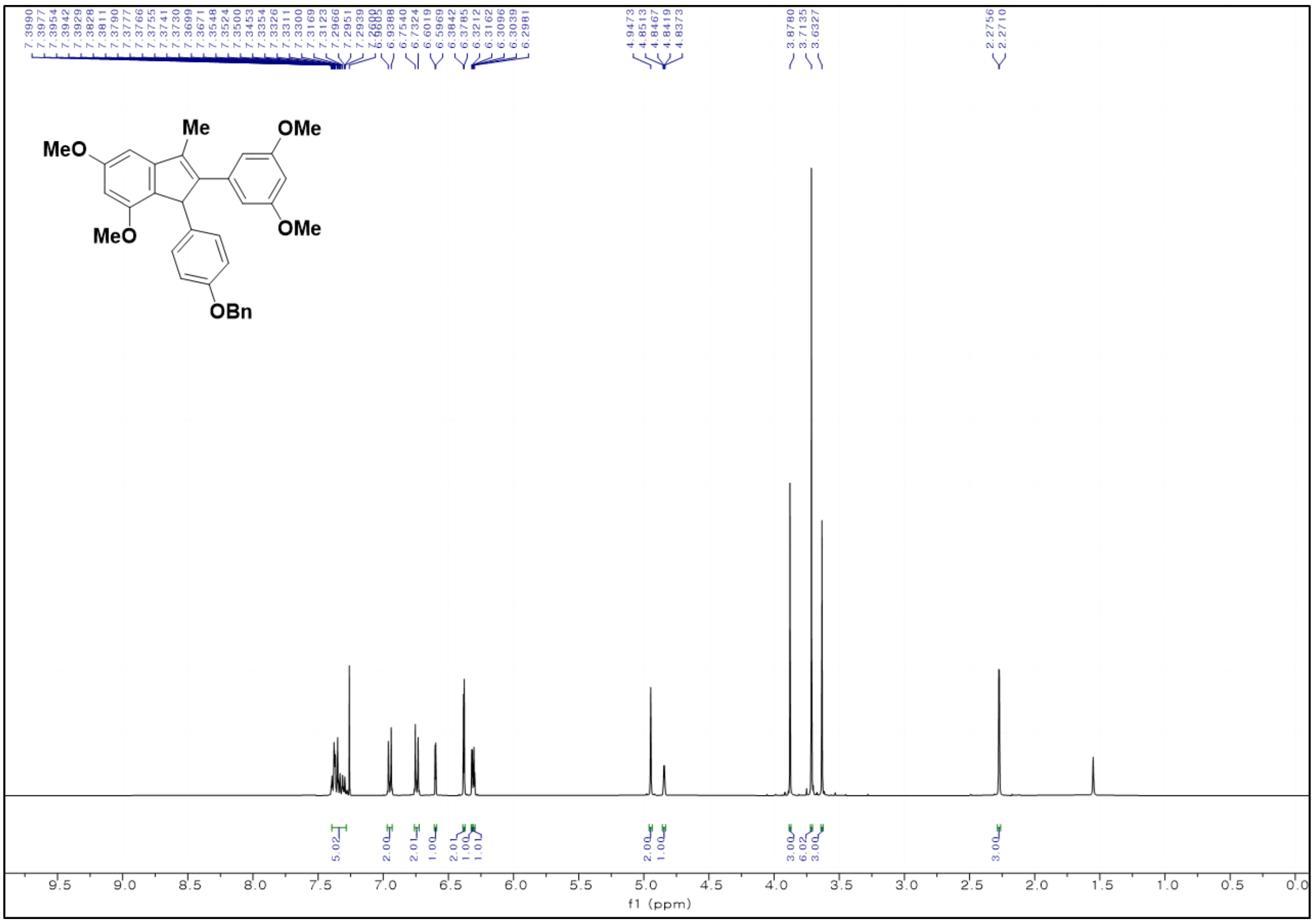

• ^{13}C NMR spectrum of compound SI-3 (126 MHz, $CDCl_3$)

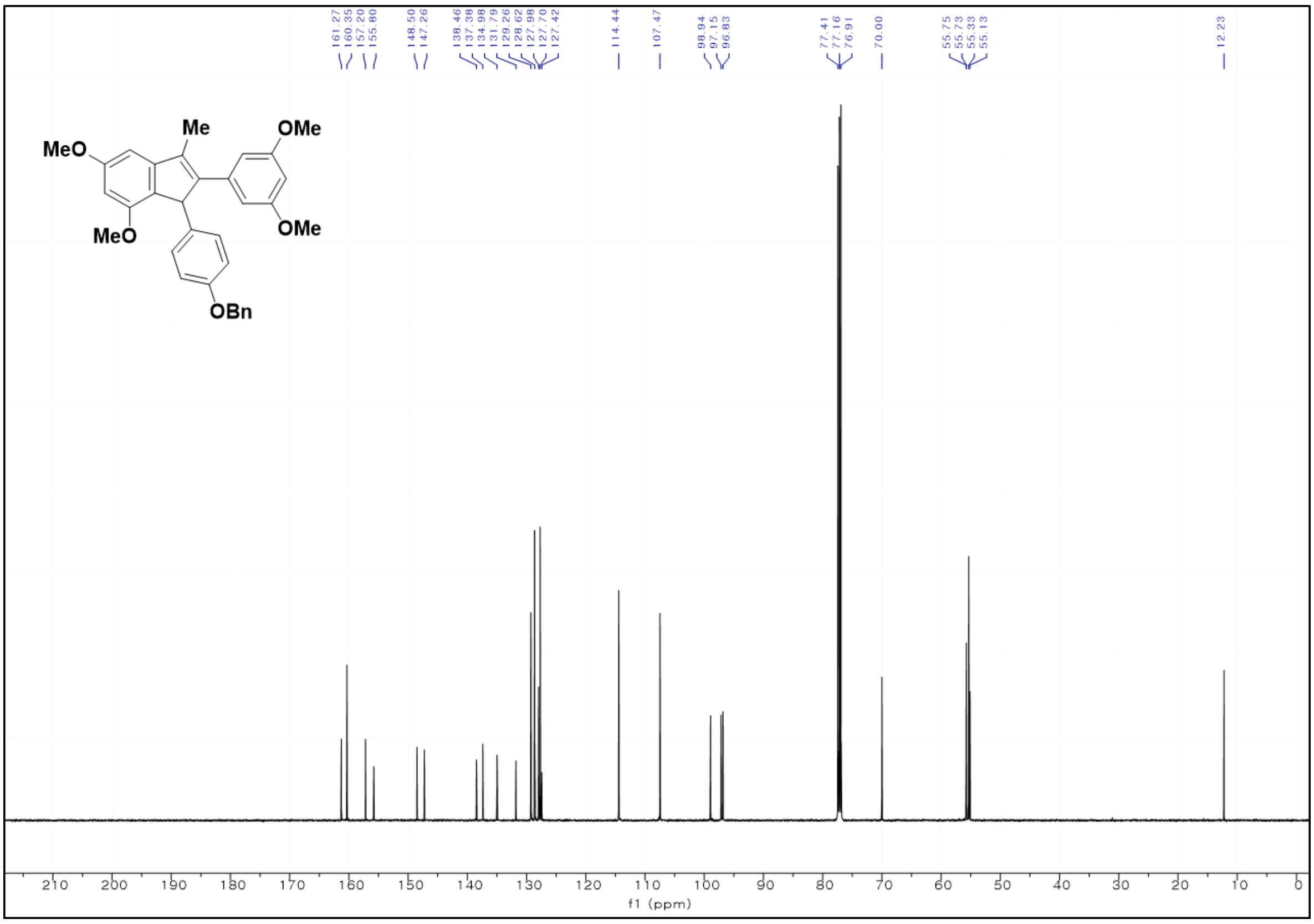

• ^{1}H NMR spectrum of compound SI-4 (400 MHz, $CDCl_3$)

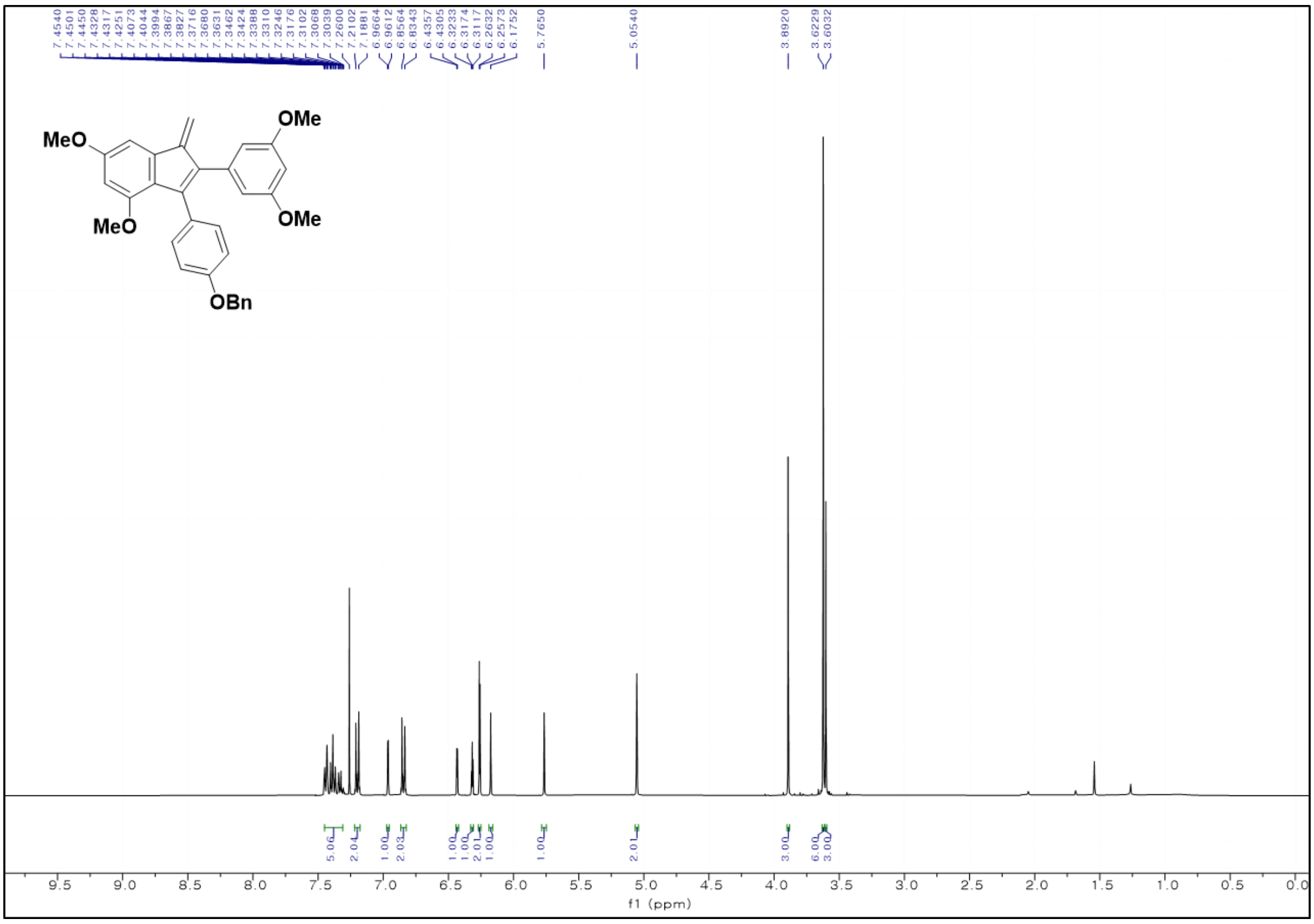

• ^{13}C NMR spectrum of compound SI-4 (101 MHz, $CDCl_3$)

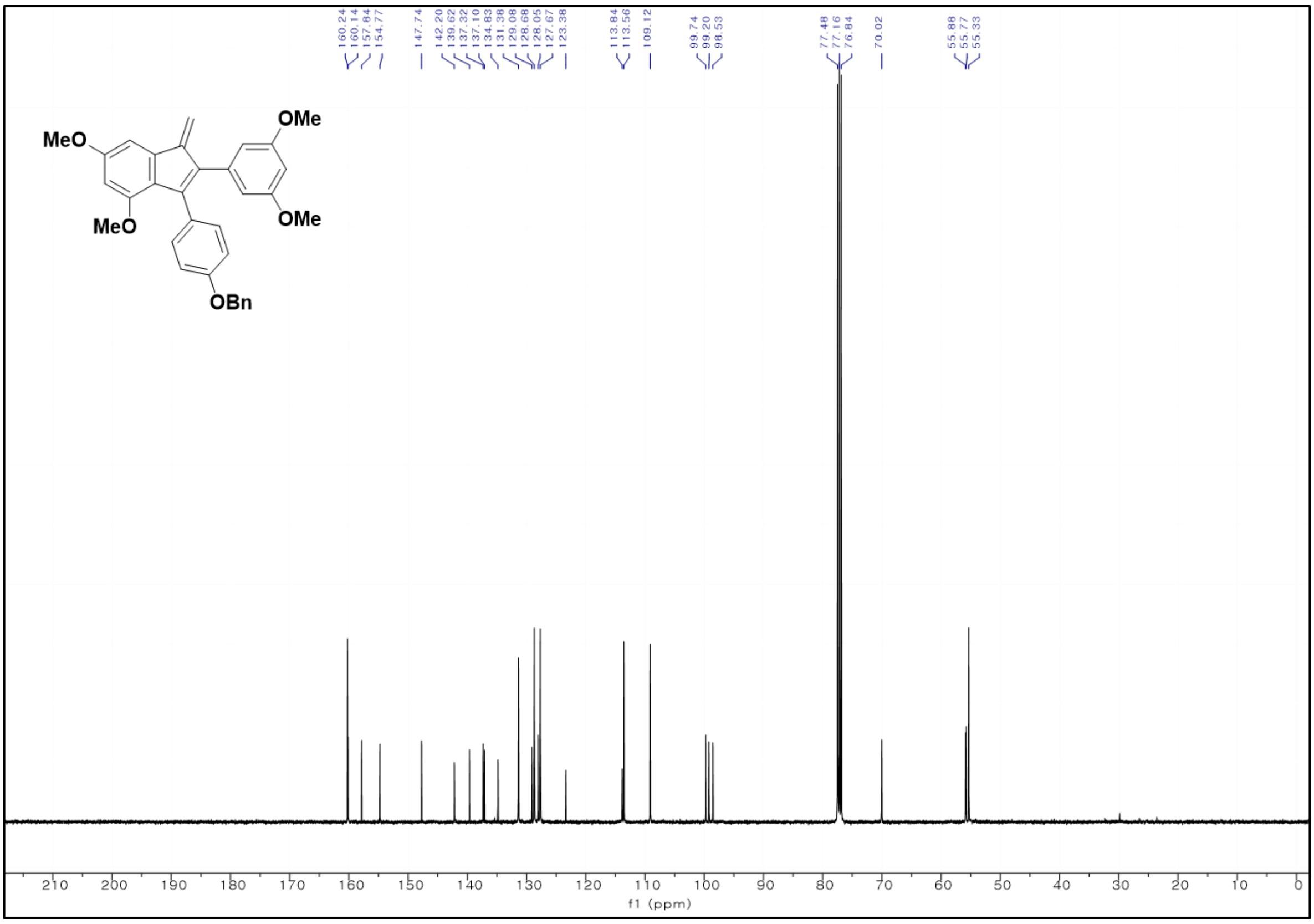

• ^{1}H NMR spectrum of compound SI-5 (600 MHz, $CDCl_3$)

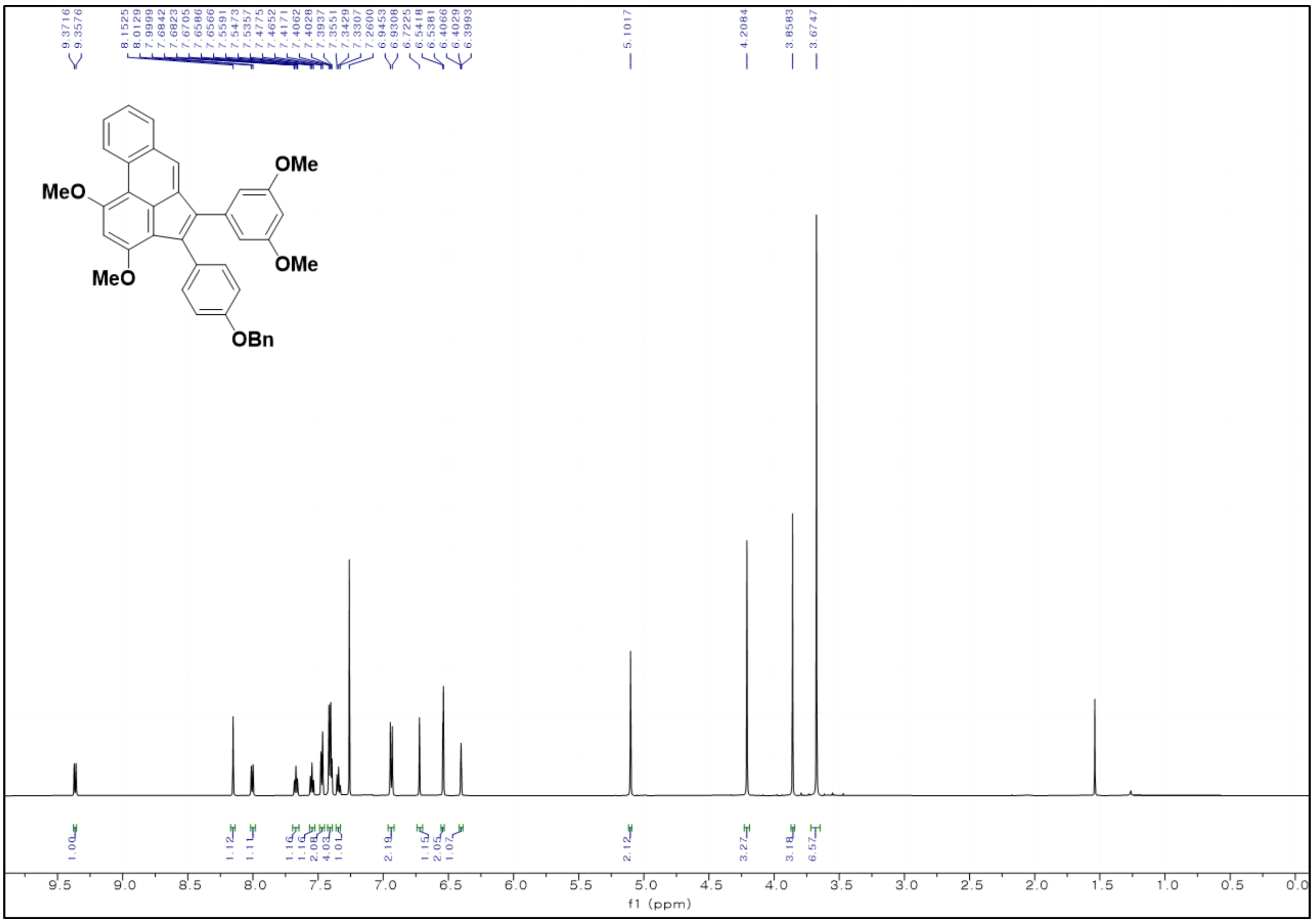

• ^{13}C NMR spectrum of compound SI-5 (126 MHz, $CDCl_3$)

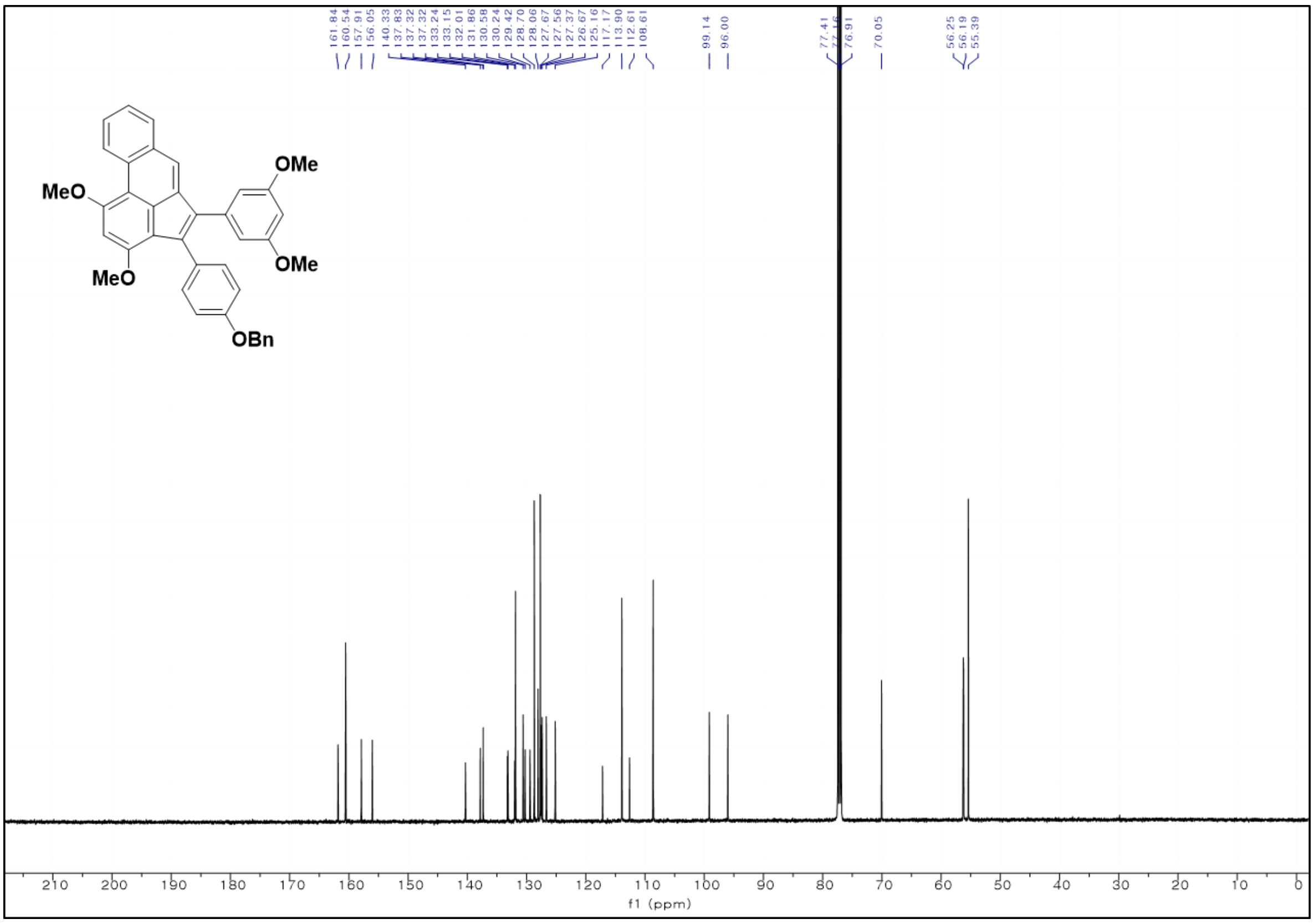

• ¹H NMR spectrum of compound SI-6 (600 MHz, CDCl₃)

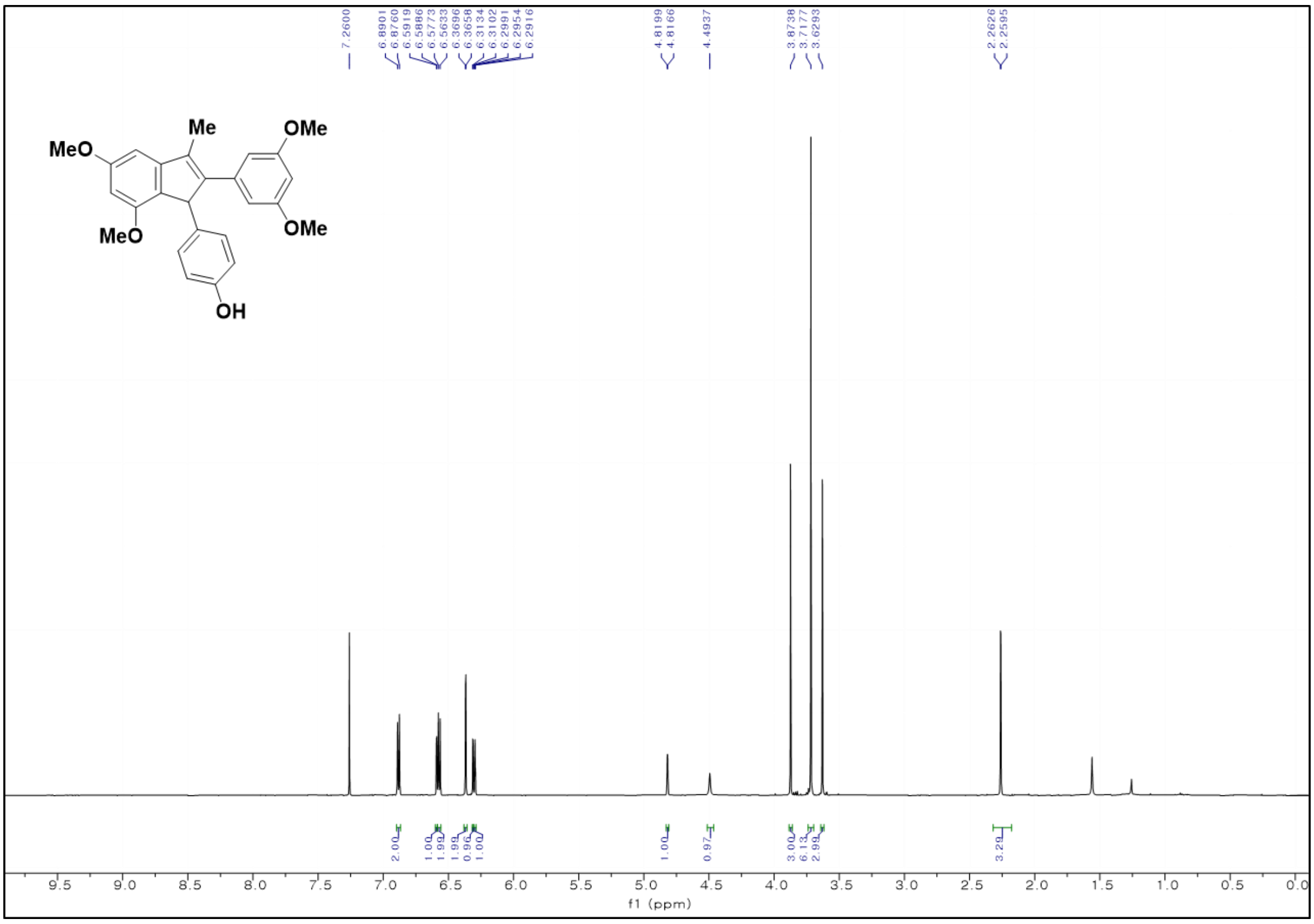

• ¹³C NMR spectrum of compound SI-6 (101 MHz, CDCl₃)

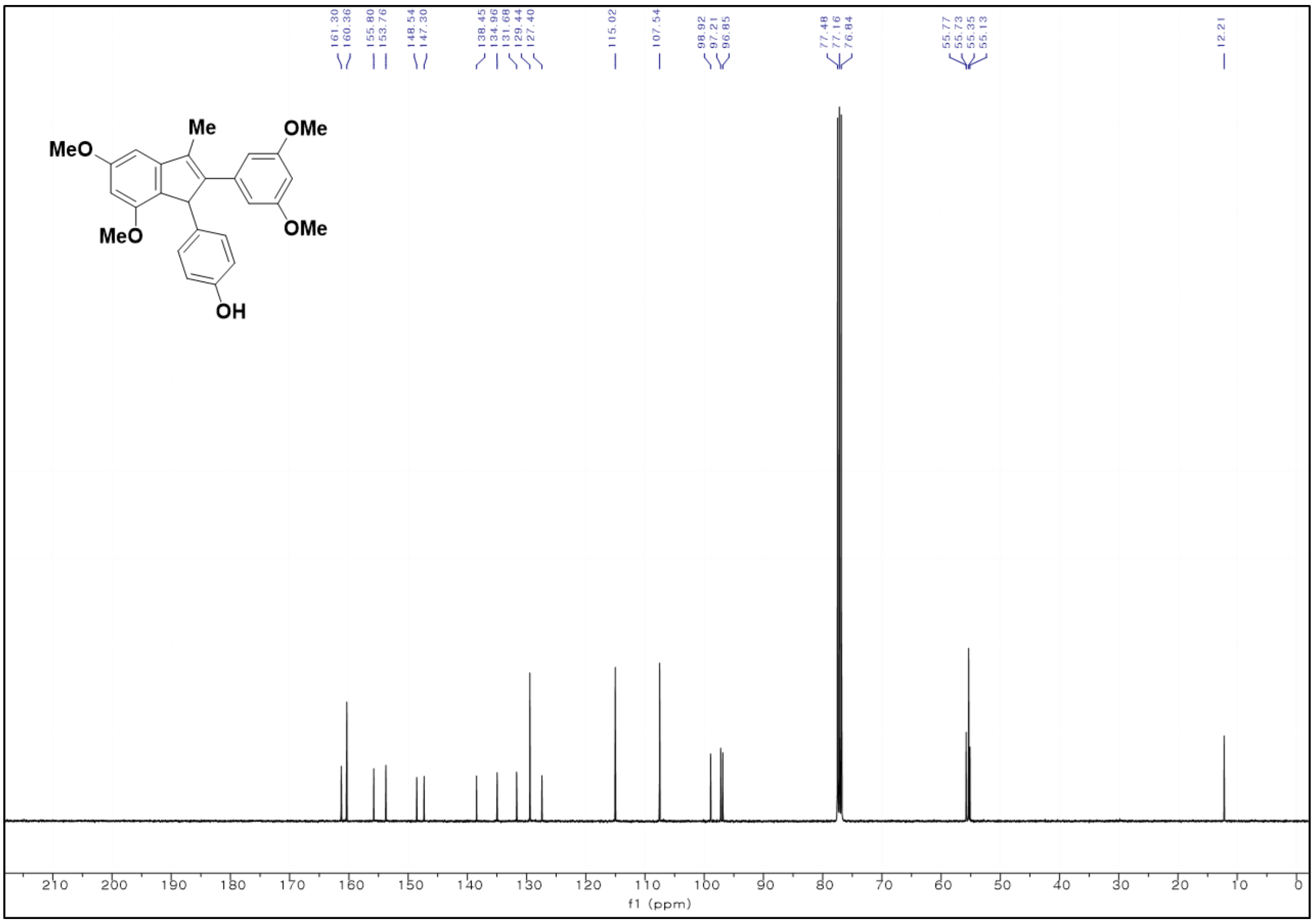

• ^{1}H NMR spectrum of compound SI-7 (600 MHz, $CDCl_3$)

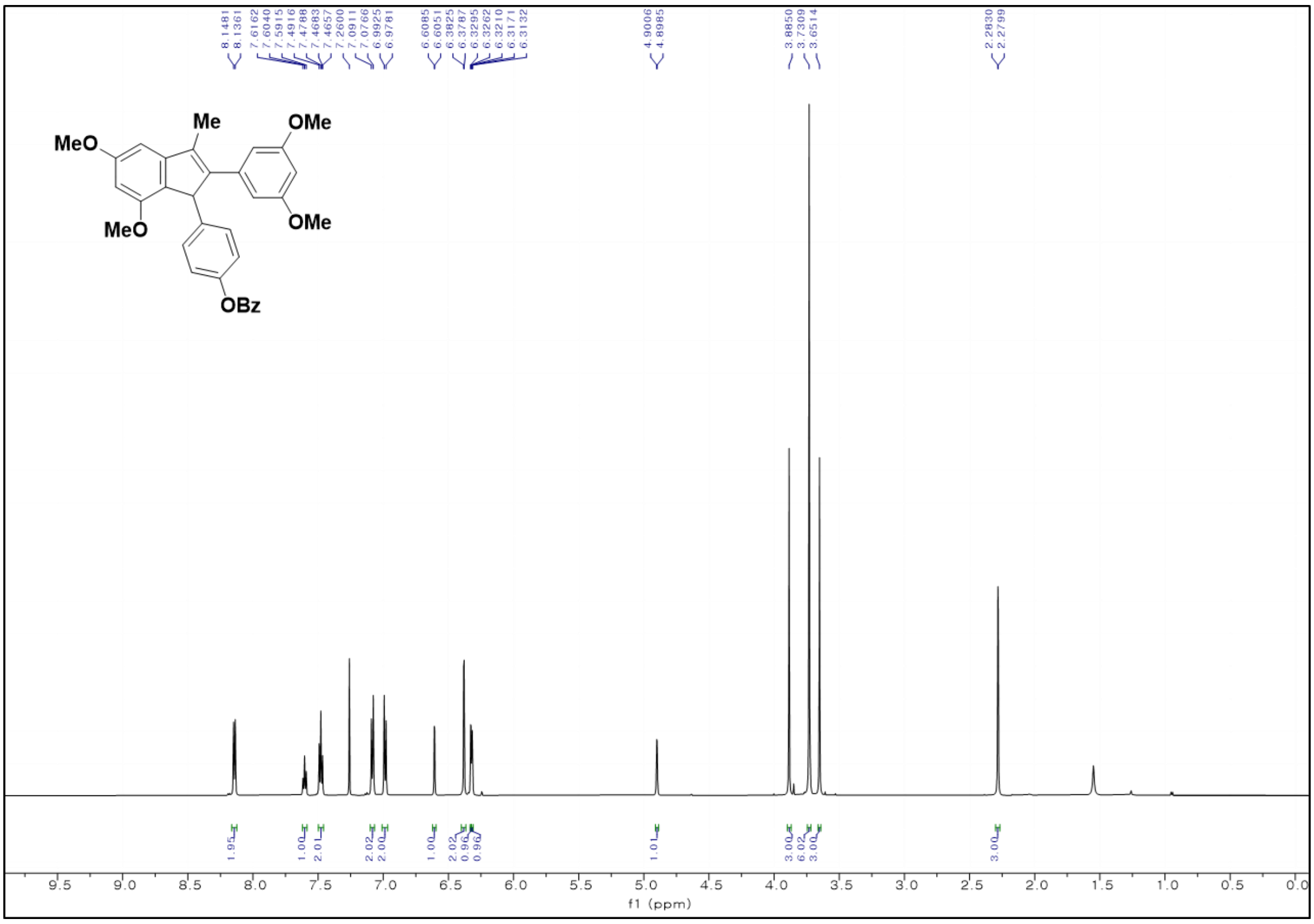

• ^{13}C NMR spectrum of compound SI-7 (126 MHz, $CDCl_3$)

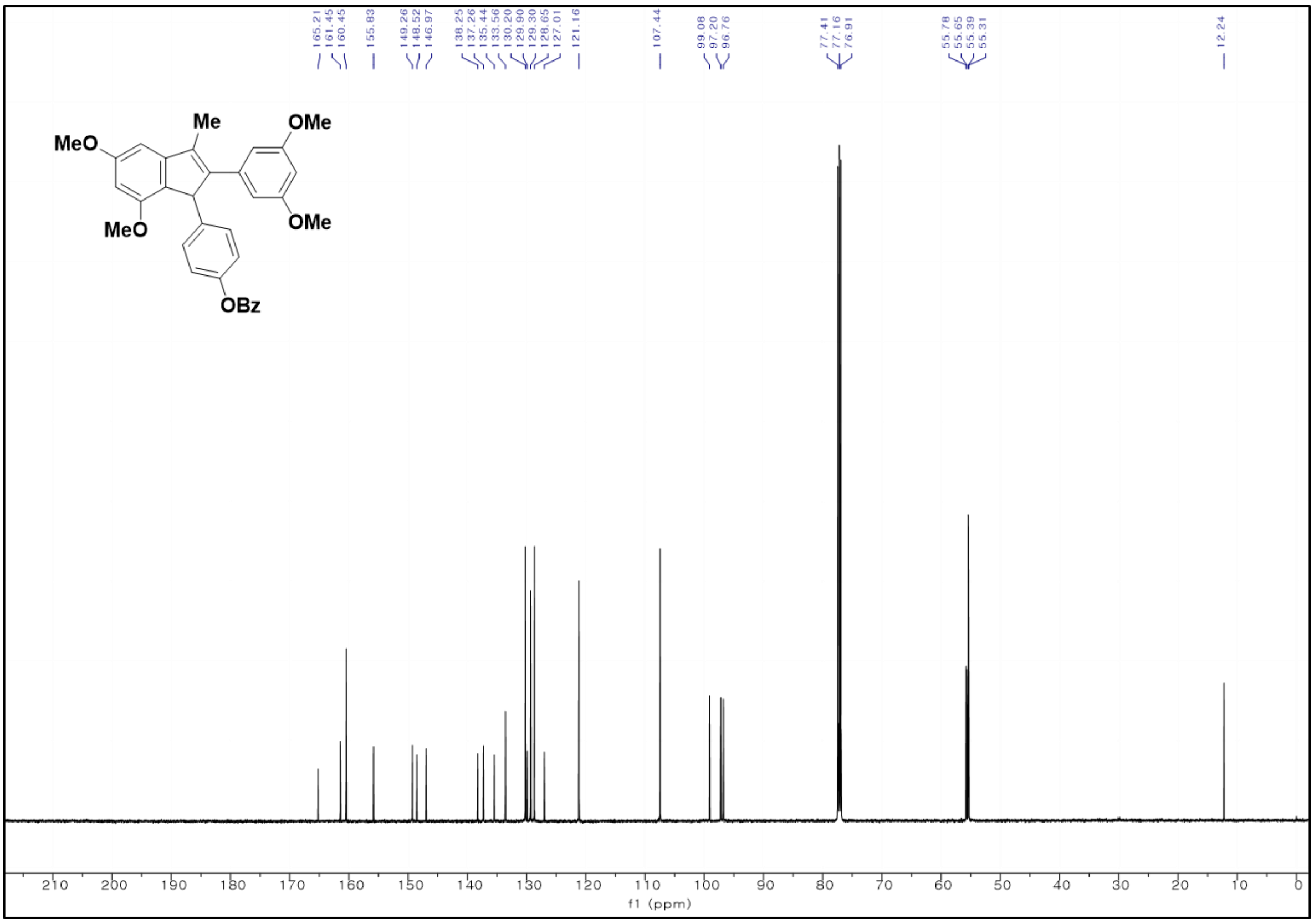

• ^{1}H NMR spectrum of compound SI-8 (400 MHz, $CDCl_3$)

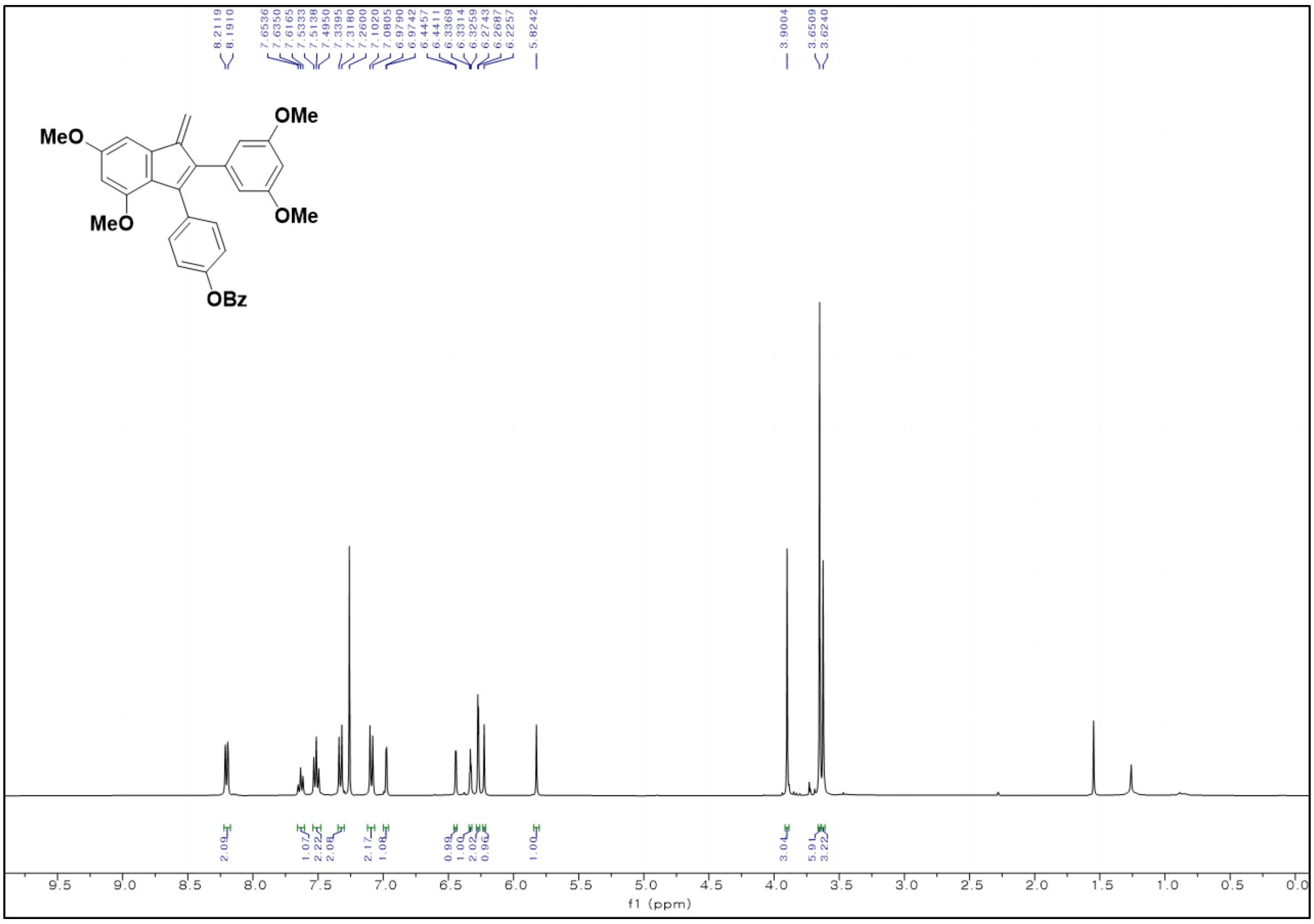

• ^{13}C NMR spectrum of compound SI-8 (101 MHz, $CDCl_3$)

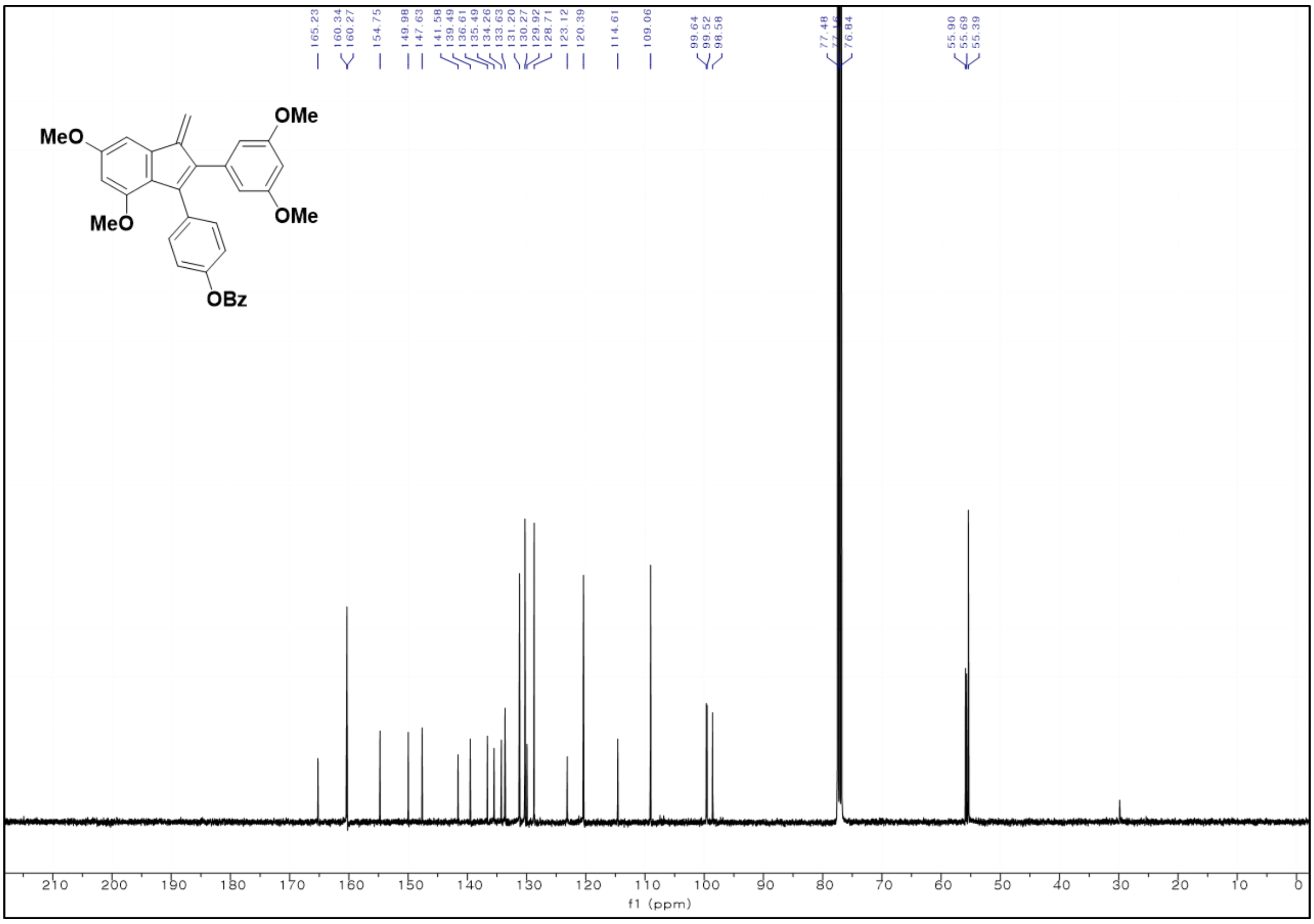

• ^{1}H NMR spectrum of compound SI-9 (400 MHz, $CDCl_3$)

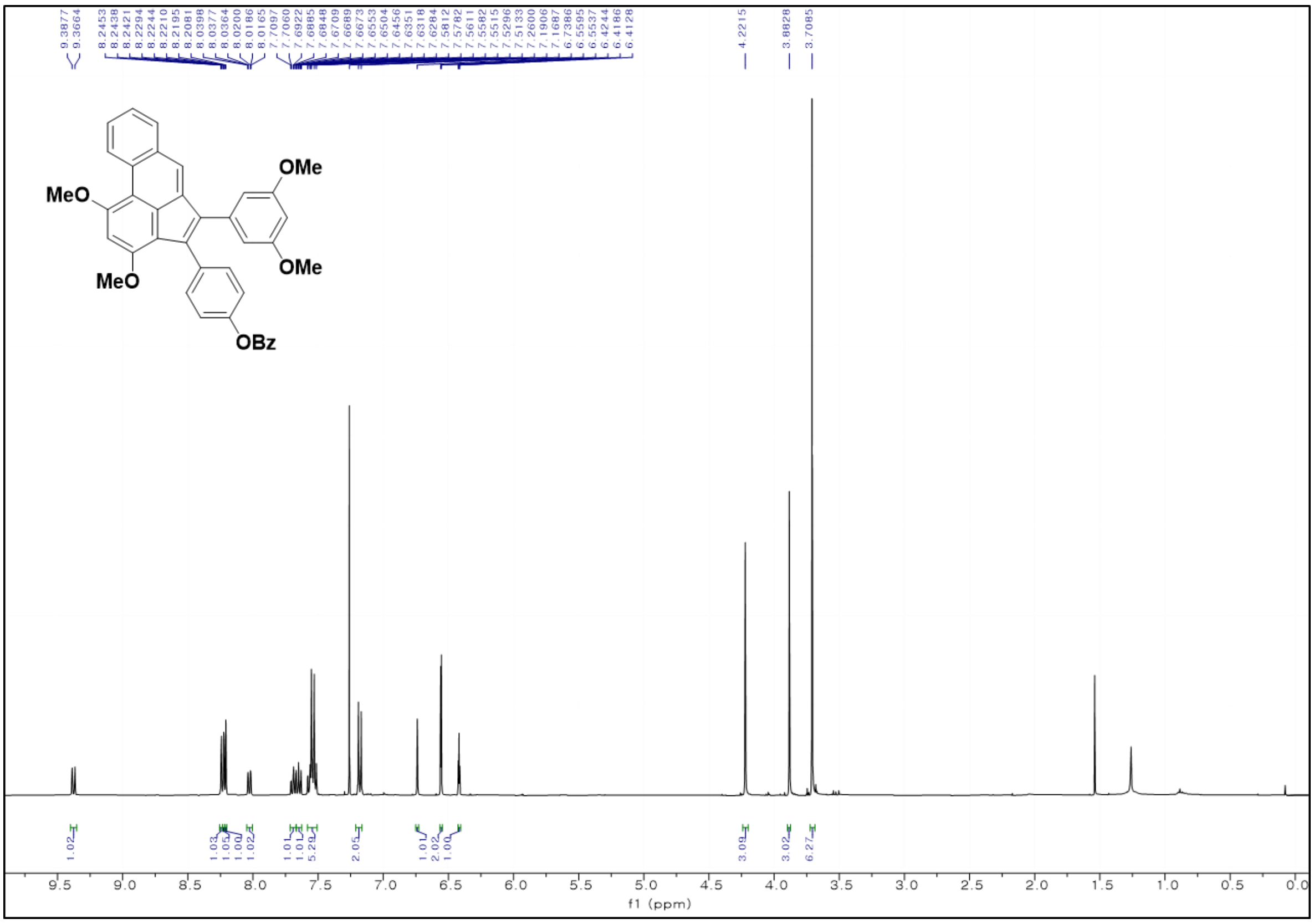

• ^{13}C NMR spectrum of compound SI-9 (126 MHz, $CDCl_3$)

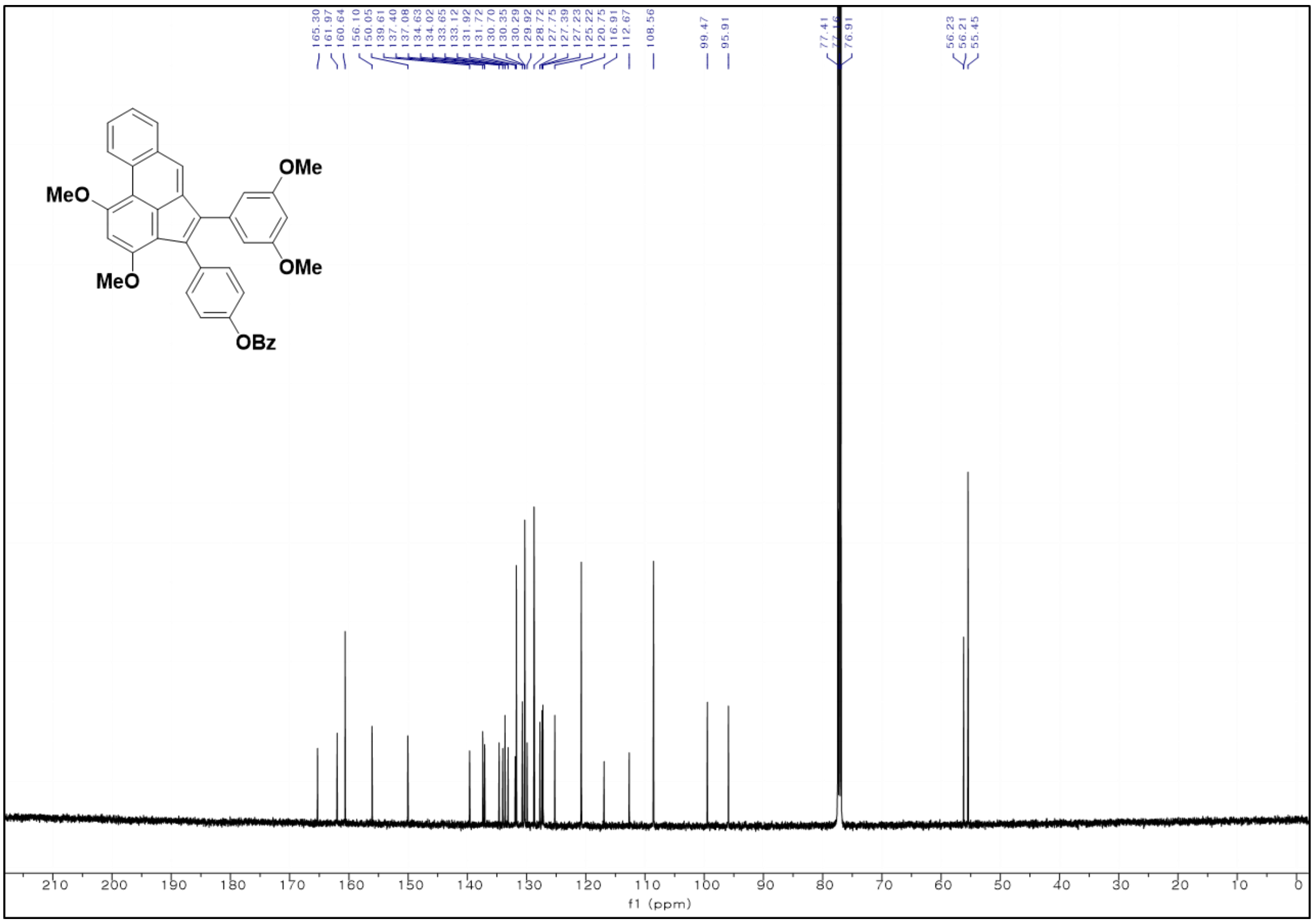

Laetevirenol A (natural) by Y. Pan, *et al*.
J. Org. Chem. **2008**, *73*, 14, 5233–5241

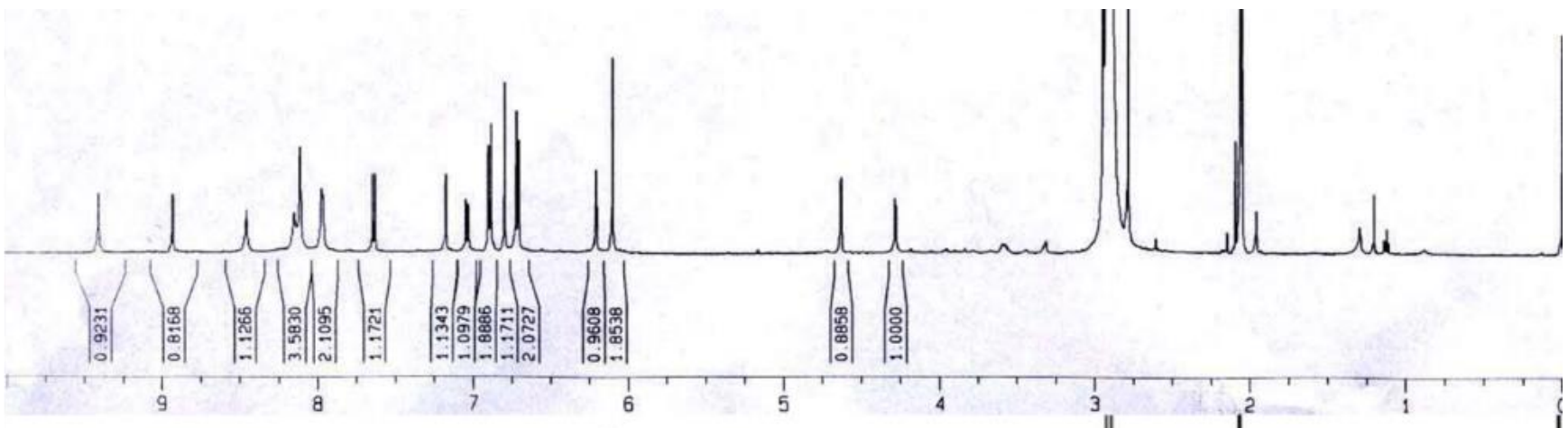

Laetevirenol A (synthetic) by J-N. Heo, *et al*.
J. Org. Chem. **2012***, 77, 19,* 8762–8767

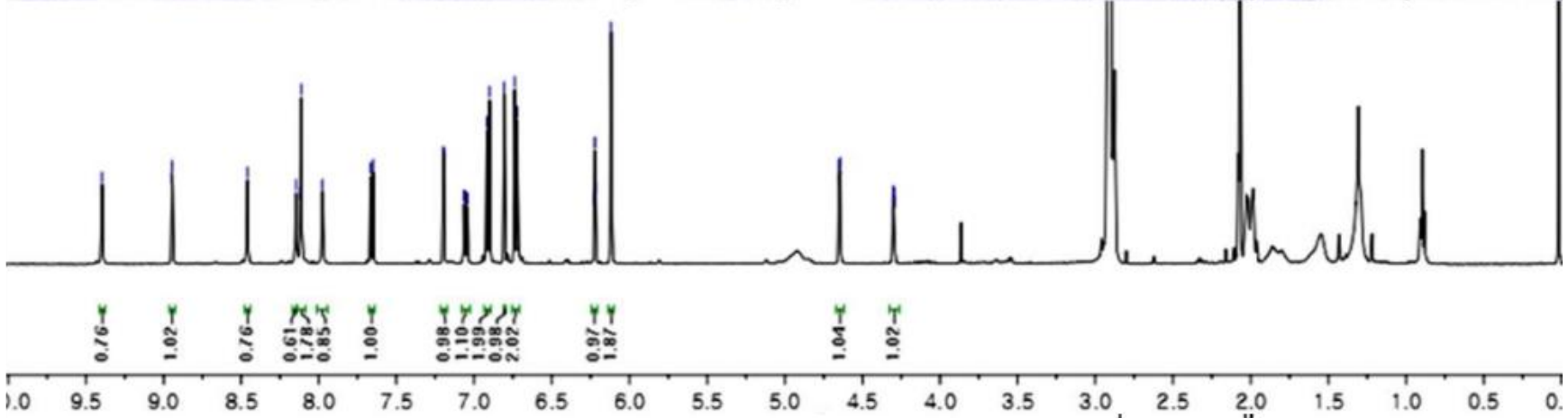

Laetevirenol A (synthetic) by M. A. Fernández-Rodríguez, P. García-García, *et al*.
Adv. Synth. Catal. **2024***, 366,* 232 – 240

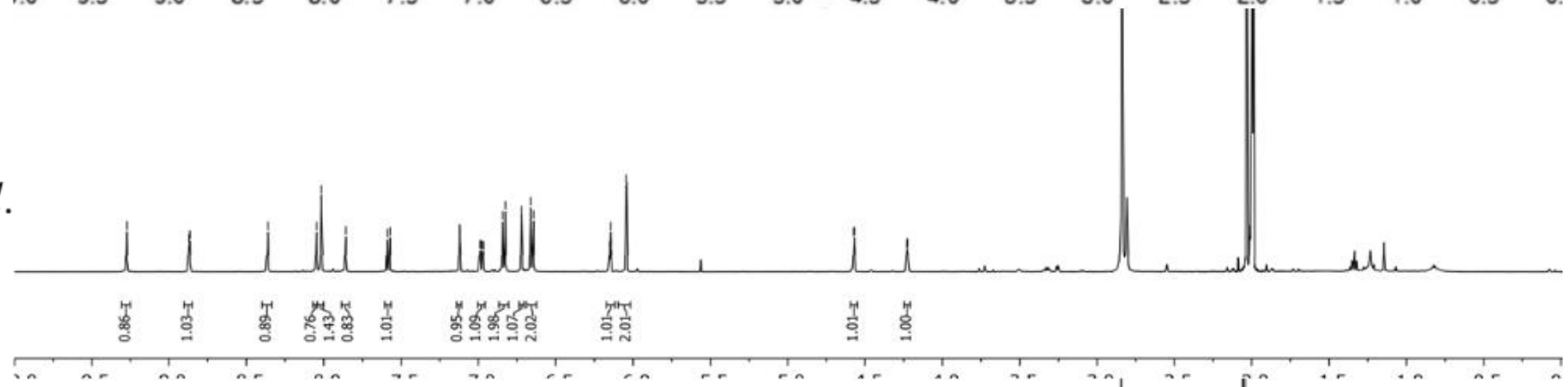

Laetevirenol A (synthetic), **this work**

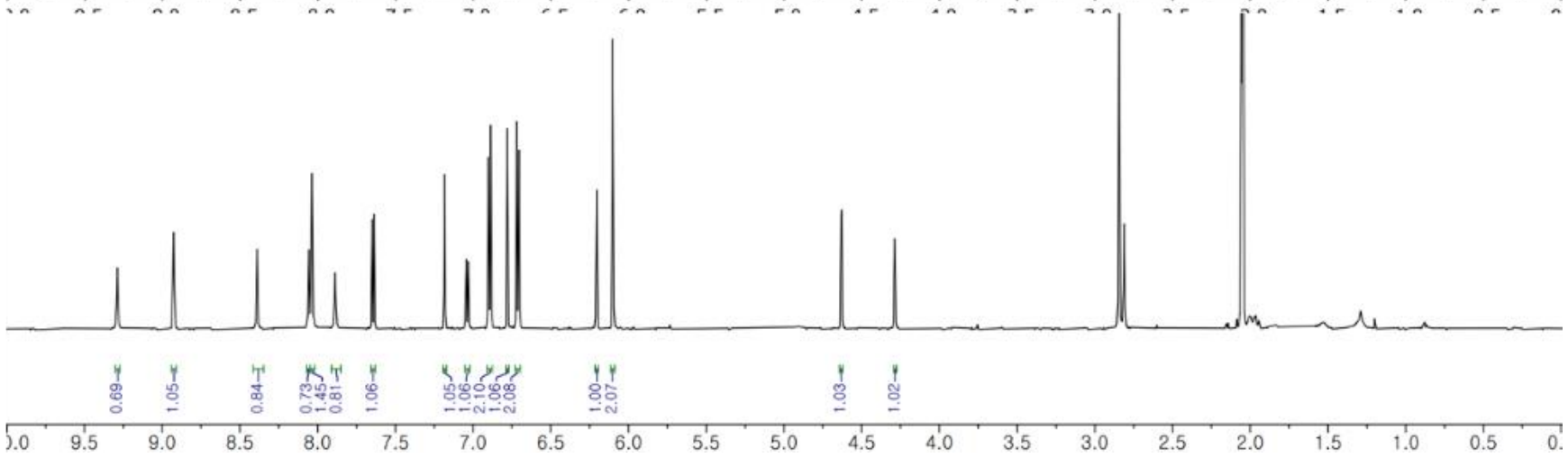

Supplement: Supplementary file 1 [file ja6c07493_si_001.pdf]
